# Efficacy of Topical Intervention for Recurrent Aphthous Stomatitis: A Network Meta-Analysis

**DOI:** 10.3390/medicina58060771

**Published:** 2022-06-07

**Authors:** Hao Liu, Lei Tan, Gege Fu, Ling Chen, Hua Tan

**Affiliations:** 1 College of Stomatology, Chongqing Medical University, Chongqing 400016, China; haoliu@hospital.cqmu.edu.cn; 2 College of Nursing, Chongqing Medical University, Chongqing 400016, China; tanleilei@stu.cqmu.edu.cn; 3 First Clinical College, Chongqing Medical University, Chongqing 400016, China; fugege@stu.cqmu.edu.cn; 4 The Center of Experimental Teaching Management, Chongqing Medical University, Chongqing 400016, China

**Keywords:** recurrent aphthous stomatitis, network meta-analysis, topical intervention

## Abstract

*Background and objectives:* To compare the efficacy and safety of topical interventions used for recurrent aphthous stomatitis. *Materials and Methods:* This network meta-analysis was conducted in accordance with the PRISMA statement. We searched four electronic databases, PubMed, Web of Science (WOS), Cochrane Central Register of Controlled Trials and Embase, for randomized controlled trials reporting efficacy and safety data on topical interventions for recurrent aphthous stomatitis. We performed a quality evaluation using a methodology based on the Cochrane Handbook. Two authors independently extracted data on healing effect, size reduction effect, symptom reduction effect, recurrence and safety assessment. Network meta-analysis was then performed using ADDIS and RevMan. *Results*: A total of 72 trials (5272 subjects) involving 29 topical interventions were included. Honey, lnsulin liposome gel, laser, amlexanox, glycyrrhiza and triamcinolone had better efficacy performance. Probiotics and chlorhexidine helped to prolong ulcer intervals and reduce recurrence. Doxycycline and penicillin had a high risk of adverse events. Hematologic evaluation showed no preference. The rank possibility of size-reducing effect and symptom-reducing effect supported the short-term effect of laser and the long-term effect of probiotics. *Conclusions*: We recommend the use of laser as a short-term intervention during the exacerbation phase of RAS and probiotics as a long-term intervention during the exacerbation and remission phases of RAS.

## 1. Introduction

Recurrent aphthous ulcer (RAU) or recurrent aphthous stomatitis (RAS) is a very common disease of the oral mucosa. The prevalence can range from 1.4% to 21.4% [1,2,3,4,5], according to retrospective population-based studies in different countries and regions. Research on the etiology and pathogenesis of RAS is extensive. Many views have suggested that the oral microbiota may be the culprit [6], with *Streptococcus* [7], *Helicobacter pylori* [8], *cytomegalovirus* [9] and a variety of obscure microorganisms [10] all thought to be potentially important members of the core microbiota responsible for ulcers. Systemic diseases can also have ulcers as an important phenotype [11], adding to the uncertainty of etiologic tracing. In conclusion, the etiology and pathogenesis of RAS are not fully understood, leading to the fact that specific treatments for RAU have not been identified in clinical and basic trials [12]. The current treatment for RAS is still mainly symptomatic, with the main objectives of relieving pain, promoting lesion healing and prolonging the interval period. Topical treatment is considered effective for the treatment of minor recurrent aphthous ulcer (MiRAU) and as an adjunct to the treatment of major recurrent aphthous ulcer (MaRAU). Topical medication, laser, cryotherapy and cautery are currently considered to be effective topical treatments [13]. Topical glucocorticoids are the first-line drug for topical application. They are used for the local treatment of RAS through their anti-inflammatory and immunosuppressive effects, such as dexamethasone and triamcinolone [14]. Tetracyclines and their derivatives (especially doxycycline) are also thought to inhibit ulcer formation and tissue destruction due to lesions. They act by inhibiting matrix metalloproteinases in the inflammatory pathway [15]. Amlexanox has also been repeatedly mentioned in current studies. It is thought to have anti-inflammatory and anti-allergic properties [16]. Biological and laser treatments are new therapeutic techniques for mucosal diseases that are now expected to have high expectations. Recent systematic analyses have affirmed the role of changes in oral flora in the progression of RAS [17], which also provides the basis for topical probiotic interventions. Laser therapy has had a positive effect in accelerating tissue repair and relieving pain [18], with satisfactory responses to the management of RAS. Some traditional treatments such as freezing [19] and cautery [20] have a positive effect on cell metabolism and tissue regeneration which are still heavily used in some areas. Low serum zinc levels have also been shown to be a risk factor for RAS in numerous studies [21]; hence, topical zinc supplementation is also recommended. In addition, some natural extracts such as curcumin [22], glycyrrhiza [23], honey [24], quercetin [25], chitosan [26], aloe [27], berberine gelatin [28], diosmectite [29], allicin [30] and other extracts have been shown in some studies to be promising topical interventions for ulcers.

RAS is a self-limiting disease that can vary in duration and status from one individual to another. Most cases of recurrent Aphthous stomatitis only last a few days and then tend to heal. However, the quality of daily life is severely affected by localized mucosal defects resulting in poor palatability, pain due to chemical-mechanical irritation and recurrent episodes [31]. To date, several authors have conducted traditional meta-analyses of local interventions for RAS [32,33,34]. Their focus has been on whether the interventions studied have a positive effect compared to an ineffective placebo. Such an approach to research dictates that only pairwise comparative estimates of effect can be derived, but the relative efficacy of more than two interventions cannot be compared simultaneously. Which local intervention is most efficacious for RAS remains controversial, and there is a lack of valid research evidence.

Based on a search of relevant databases, no comprehensive systematic evaluation and ranking of multiple local interventions for the treatment of RAS has been identified. Therefore, we reviewed existing studies and included multiple randomized controlled trials (RCTs) in a systematic evaluation study to assess the efficacy and safety of as many as 29 local interventions for the treatment of RAS. Our aim was to provide a reliable reference for the selection of more efficient topical treatment options for patients with RAS.

## 2. Materials and Methods

### 2.1. Statement

Network meta-analysis (NMA) is a new type of high-quality analysis method. It is based on the assumptions of homogeneity, transferability and consistency. It fulfils the purpose of allowing simultaneous comparisons between multiple interventions and also provides a possible ranking of the effectiveness of different interventions [35]. As a result, NMA is used in many studies to provide more efficient evidence and to select superior solutions. Our study was carried out in strict accordance with the criteria and requirements for conducting the NMA [36]. Our study was conducted in accordance with the relevant statements. The Addis, Revman, Endnote and other software used in the study complied with the relevant operational requirements. The study was registered in the PROSPERO International Prospective Register of Systematic Reviews in accordance with the relevant requirements prior to implementation (CRD42021251154).

### 2.2. Data Sources and Search Strategy

Four databases, PubMed, Web of Science (WOS), Cochrane Central Register of Controlled Trials, and Embase, were searched during the study. The search was conducted from the date of creation of the database to 1 October 2021. The search was carried out using the medical terms “Stomatitis, Aphthous”, “Oral Ulcer” and “Clobetasol”. Synonyms and abbreviations such as “Canker sore”, “Corticosteroid” and “LLLT*” were also used as keywords to broaden the search. The search process was carried out by two independent individuals, and Endnote literature management software was used to manage the search results. The search strategy is detailed in Tables S1–S5.

### 2.3. Selection Criteria

The RCTs included in the study met the following criteria:

(1) Clinical or histopathological examination confirms a diagnosis of recurrent aphthous ulcers, with ulcer-like lesions visible anywhere on the oral mucosa.
(2) Simple ulcerative lesions of any undetermined cause such as psychological, nutritional and immunological factors, rather than oral manifestations of systemic diseases such as leukoaraiosis, diabetes mellitus, etc., or specific ulcerative lesions due to trauma, radiotherapy, etc.
(3) The population enrolled received only local interventions or placebo during the trial and did not receive any other treatment that might alter the RAS prior to or during the trial, such as receiving systemic steroids or immunosuppressants.
(4) For studies in patients with multiple oral mucosal diseases, we extracted only RAS data. If this was not possible, we excluded the study.

### 2.4. Outcomes

In this study, clinical efficacy and safety were selected as outcome indicators. Clinical efficacy was assessed by healing efficacy, effect of size reduction and effect of symptom reduction. Healing efficacy was assessed by the time to healing, i.e., the time elapsed from the time the subject was enrolled for the local intervention to the time the ulcer-like lesion was completely healed. The effect of size reduction was assessed by the efficacy index (EI). In this case, EI was calculated by EI = ulcer reduction area (mm^2^)/ulcer baseline area (mm^2^). The cumulative reduction in ulcer size on different examination days over the duration of the trial was counted. The effect of symptom reduction was also assessed by the efficacy index (EI). In this case, EI was calculated by EI = reduction in pain score/baseline ulcer pain score. Individual subject’s VAS score or decile scale score of pain level on different examination days. The degree of cumulative pain relief was calculated (VAS score: 10 cm horizontal line, marked 0 = no pain to 10 = worst pain; Decile scale: 0 for no pain, 10 for most pain). Safety was evaluated by the number of adverse events and the blood levels of the intervening drug. The study also extracted an evaluation regarding the effect on RAS recurrence.

### 2.5. Data Collection and Risk of Bias Assessment

Two researchers independently read and screened the literature against the inclusion and exclusion criteria, evaluated the quality of the literature against the criteria and extracted the information. The above process was completed independently by the two researchers and cross-checked, with any disagreements being resolved by consultation with a third party or discussion. Information extracted included: the first author of included studies, time of publication, country, sample size, gender, age, interventions and outcome indicators. The quality of included studies was assessed using RevMan 5.3 software provided by the Cochrane Collaboration. The evaluation included: (1) random sequence generation; (2) allocation concealment; (3) blinding of participants and personnel; (4) blinding of outcome assessment; (5) incomplete outcome data; (6) selective reporting; (7) other bias. The potential publication bias of the included studies was analyzed using a funnel plotting approach.

### 2.6. Data Synthesis and Statistical Analysis

Traditional meta-analysis was performed using RevMan 5.3 and ADDIS 1.16. Risk difference (RD) was used as an effective indicator for dichotomous variables and mean difference (MD) was used as an effective indicator for continuous variables, and 95% confidence intervals (95% CI) were used for each effect size estimate. I2 was used to quantify the heterogeneity of the test results. If *p* ≥ 0.1 and I2 ≤ 50%, the heterogeneity among the test results was considered small, and a fixed-effects model was used for combining; if *p* < 0.1 and I2 > 50%, the heterogeneity among the test results was considered large, and a random-effects model was used for combining, and a subgroup analysis or case-by-case literature exclusion method was used for sensitivity analysis.

A random-effects network within a Bayesian framework model was constructed using ADDIS 1.16 [37]. Networks were constructed according to different outcome indicators in order to include as many local interventions as possible. Direct and indirect comparisons were made between the different interventions to ensure that the results obtained were based on comprehensiveness and completeness considerations. Statistically significant differences were considered at *p* < 0.05. Ranking probabilities were also estimated in ADDIS 1.16. The MD of each local intervention compared to an arbitrary control was calculated, and the number of iterations of the Markov chain was calculated to evaluate the degree of convergence of the model. Variance calculations and node splitting analyses were also performed to assess inconsistency in the network meta-analysis. Results were considered inconsistent if the random effects variance differed significantly from the inconsistency or if the difference between direct and indirect evidence was judged to be *p* < 0.05.

## 3. Results

### 3.1. Study Selection

**Figure 1:**
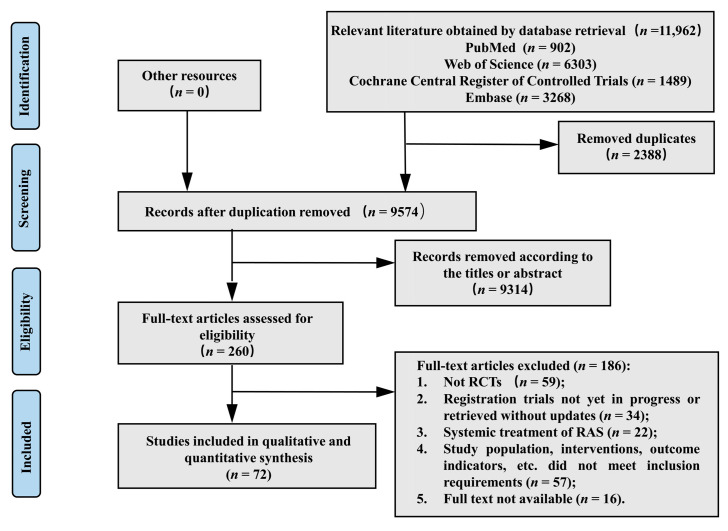
Flow chart of the study selection process.

A total of 11,962 records were identified by searching the four databases. After removing 2388 duplicate articles, we screened the titles and abstracts of 9574 articles. A total of 9314 articles that did not meet the inclusion criteria were excluded. A full-text review of 260 articles was conducted, and 186 of these were excluded, resulting in 72 eligible studies being used for qualitative and quantitative research (Figure 1).

### 3.2. Characteristics and Quality of Studies 

The 72 included studies are described in Table S6. They included three three-arm studies and two four-arm studies, in addition to one study that subgrouped the adult and child groups, which we also grouped in our study for comparison. The vast majority of subjects included were patients with definite RAS, most with definite minor RAS, and a few studies described the size of the subject’s ulcer, meeting the criteria for inclusion. Subjects were histologically and clinically confirmed, and all had definite symptoms at study entry. The minimum mean age was 6.82, and the oldest subject was 71 years. Most studies had more females than males. Treatment duration ranged from a few days to several months. Intervention forms could be loaded by a mucoadhesive matrix or presented as a paste, liquid, etc. Specific interventions and treatment plans are given in Table S6. The outcomes of the studies are summarized in Tables S7 and S8. 

**Figure 2:**
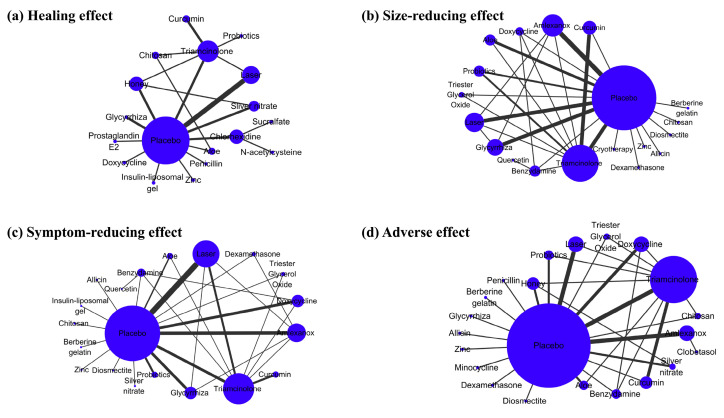
Network plots for the main outcomes considered in the review. (**a**) Healing effect; (**b**) size-reducing effect; (**c**) symptom-reducing effect; (**d**) adverse effect. Nodes and edges are weighted according to volume of studies, including that treatment or comparison.

In total, 69 studies were included to measure four evaluation items: (a) healing effect, data from 26 RCTs (1306 participants); (b) size-reducing effect, data from 37 RCTs (3587 participants); (c) symptom-reducing effect, data from 46 RCTs (4020 participants); (d) adverse effect, data from 36 RCTs (2787 participants). The network structure is shown in Figure 2. Three studies, because of incomplete data, only had the information about recurrence outcomes and were evaluated descriptively. In addition, 4 of the 72 studies described hematologic values, which we also evaluated descriptively only. The risk of bias estimates are shown in Figures S1 and S2. The majority of the studies showed a low risk of bias in terms of “incomplete outcome data”, “selective reporting” and “other bias”. Because some of the articles did not detail the specific processes for allocation, randomization, and measurement in the text, the risk estimates for “random sequence generation”, “allocation concealment” and “bling of outcome assessment” were judged as unclear risks. Some studies were single-blinded because of the limitations of interventions such as laser, cauterization and freezing, which did not completely shield participants, and therefore were considered high risk for “blinding of participants and personnel“. However, this does not mean that the quality of the included articles is too low. Using RevMan 5.3 software as an aid, we created funnel plots (Tables S3–S6).

### 3.3. Pairwise Meta-Analysis

Data from 14 RCTs (n = 820) with seven pairwise comparisons were pooled for healing effect (Chapter S1). Triamcinolone, laser, silver nitrate, and honey all showed a significant preference compared to placebo, while curcumin vs. triamcinolone and triamcinolone vs. placebo did not show a clear preference in the comparison. 

Data from 23 RCTs (n = 1807) with eight pairwise comparisons were pooled for size-reducing effect (Chapter S2). Triamcinolone, probiotics, glycyrrhiza and amlexanox were significantly better than placebo, while aloe vs. placebo, curcumin vs. triamcinolone, laser vs. placebo and probiotics vs. triamcinolone did not show a statistically significant difference. 

Data from 32 RCTs (n = 2940) with nine pairwise comparisons were pooled for symptom-reducing effect (Chapter S3). Triamcinolone, laser, glycyrrhiza, amlexanox and aloe were considered superior to placebo. Preference was given to laser over triamcinolone. Curcumin vs. triamcinolone, doxycycline vs. placebo, and probiotics vs. placebo were considered indistinguishable. 

Data from 22 RCTs (n = 1748) with nine pairwise comparisons were pooled for adverse effects (Chapter S4). There were no statistically significant differences found between any of them.

In addition, there is one point that needs to be added about the two outcome indicators, the size-reducing effect and symptom-reducing effect. Considering that the reporting time of the different studies was not far apart and did not meet the requirement to be divided into long-term and short-term comparisons, we performed pairwise comparisons only at the endpoint of treatment. However, more detailed and precise comparisons of daily changes were also performed, which are shown in Chapters S2 and S3.

### 3.4. Network Meta-Analysis

#### 3.4.1. Healing Effect

Data from 26 RCTs [20,24,26,38,39,40,41,42,43,44,45,46,47,48,49,50,51,52,53,54,55,56,57,58,59,60] with 20 pairwise comparisons among 18 interventions were pooled. The relative effect estimates for honey, insulin liposome gel and laser exhibited shorter healing times and better healing effects relative to placebo. (Estimates values are shown in Table 1). No significant differences were observed in the other comparisons (more details are shown in Chapter S1). From our analysis, we concluded that these 18 local interventions may have consistent or similar performance in terms of healing effect. Compared to placebo, the rank possibility (only the top five are listed in Table 2, more details are shown in Chapter S1) indicates that the best intervention is Insulin-liposomal gel (*p*-core, 0.24), followed by honey (*p*-core, 0.15), laser (*p*-core, 0.11), penicillin (*p*-core, 0.09) and aloe (*p*-core, 0.06).

#### 3.4.2. Size-Reducing Effect

Data from 37 RCTs [19,22,25,27,28,29,30,40,42,48,50,55,61,62,63,64,65,66,67,68,69,70,71,72,73,74,75,76,77,78,79,80,81,82,83,84] with 30 pairwise comparisons among 19 interventions were pooled. Network meta-analysis indicated that amlexanox, glycyrrhiza and triamcinolone were more effective than placebo (Table 1). No significant differences in size-reduction effect were shown for the other interventions (Chapter S2). Based on the rank possibility (Table 2, Chapter S2), the optimal solution is quercetin (*p*-core, 0.27). The other possible efficient interventions in priority order are dexamethasone (*p*-core, 0.14), amlexanox (*p*-core, 0.13), glycyrrhiza or laser (*p*-core, 0.08) and curcumin (*p*-core, 0.08).

**Chart 1:**
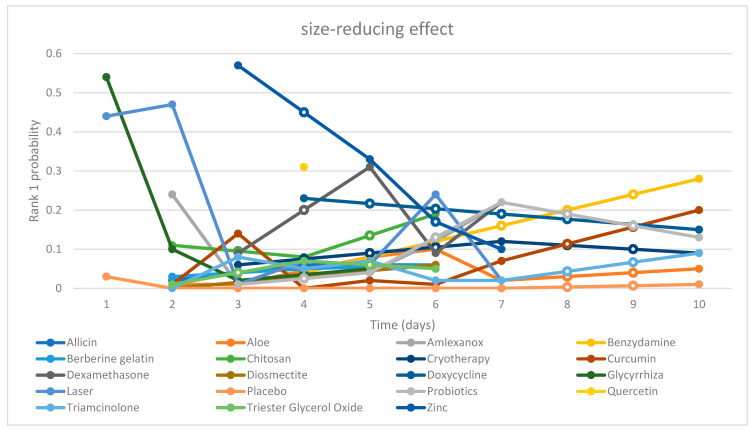
“Time-Rank 1 probability” folding line chart of side-reducing effect.

Most studies not only assessed efficacy at the end of the intervention but also performed many measurements during the treatment period. To take full advantage of these data to compare the change in ulcer size for each day of the treatment period, a more detailed ranking was performed (Chapter S2). This ranking uses days as the unit of time rather than the full treatment period. We extracted the probability that each intervention was first-ranked (*p*-core) and plotted it as a “Time-Rank 1 probability” folding line chart to more accurately reflect the effect of the intervention on ulcer size (Chart 1). We considered laser, glycyrrhiza and zinc to be the most potential to significantly reduce ulcer size in the short term.

#### 3.4.3. Symptom-Reducing Effect

Data from 46 RCTs [20,25,28,29,30,38,43,44,46,48,50,51,52,53,55,61,62,63,64,65,66,67,69,70,71,72,73,74,75,76,77,78,79,80,81,82,83,84,85,86,87,88,89,90,91] with 32 pairwise comparisons among 23 interventions were pooled. Amlexanox, laser and triamcinolone have a significant advantage over placebo (Table 1). No statistically significant differences were shown between the other interventions (Chapter S3). Rank possibility (Table 2, Chapter S3) is more in favor of insulin-liposomal gel (*p*-core, 0.24), followed by N-acetylcysteine or sucralfate (*p*-core, 0.12), curcumin (*p*-core, 0.08), laser (*p*-core, 0.09) and chlorhexidine (*p*-core, 0.07).

**Chart 2:**
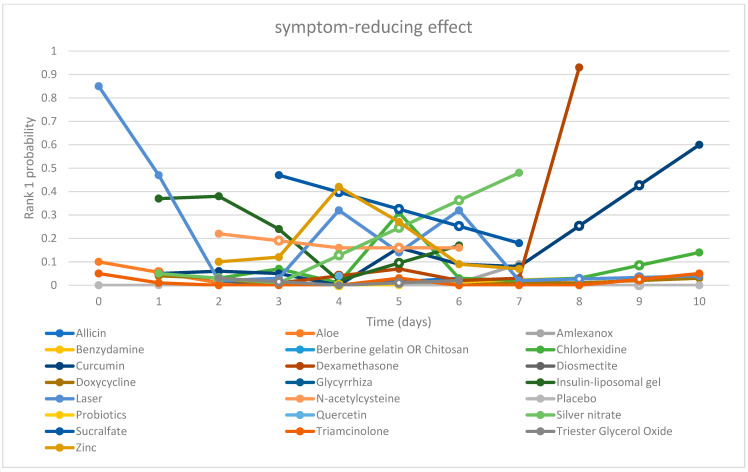
“Time-Rank 1 probability” folding line chart of symptom-reducing effect.

We also plotted the “Time-Rank 1 probability” folding line chart on the symptom reduction effect to reflect the improvement in pain per day during treatment (Chart 2). Laser, insulin-liposomal gel and sucralfate are considered as possible optimal solutions for short-term relief of ulcer-induced pain.

#### 3.4.4. Adverse Effect

Data from 36 RCTs [20,22,24,26,28,29,30,40,41,42,47,48,50,53,54,63,64,65,70,71,75,76,77,80,82,83,85,86,87,88,89,92,93,94,95,96] with 32 pairwise comparisons among 22 interventions were pooled. In terms of possible adverse events, triamcinolone performed better, with none occurring in 259 subjects, compared to amlexanox, chitosan, dexamethasone, doxycycline, penicillin and placebo. Compared to penicillin and doxycycline, dexamethasone also showed a significant improvement with 4 adverse events in 120 subjects, whereas penicillin and doxycycline showed weaknesses compared to placebo (Table 1). The other interventions were not significantly different (Chapter S4). In terms of performance on the rank possibility (Table 2, Chapter S4), triamcinolone (*p*-core, 0.15) was the optimal choice. The other interventions were berberine gelatin (*p*-core, 0.08); glycyrrhiza or laser (*p*-core, 0.07); aloe, honey or probiotics (*p*-core, 0.06); and curcumin, silver nitrate, triester glycerol oxide or zinc (*p*-core, 0.06). Possible adverse events are collated and shown in Chapter S4.

### 3.5. Other Outcome Indicators

#### 3.5.1. Hematologic Values

Four RCTs [30,77,80,82] included in the study reported on the blood levels of the intervention drug or blood laboratory findings (Table 3). There were no valuable results identified. Dexamethasone, aloe, allicin and amlexanox were not associated with hematologic safety hazards.

#### 3.5.2. Relapse

Four RCTs [58,61,97,98] describe considerations regarding RAS recurrence (Table 4). The results involved four interventions probiotics, chlorhexidine, benzydamine and triamcinolone, with outcome indicators including outbreak frequency, number of new ulcers and interval between ulcers. The investigators’ observations were that (a) probiotics helped to reduce the frequency of ulcer outbreaks in children without the same performance in adults; (b) chlorhexidine prolonged the interval between ulcers but did not significantly reduce the number of new ulcers during the trial period; (c) benzydamine was not helpful in reducing the number of new ulcers; (d) both media-based administration or administered in liquid form, the reduction in the number of new ulcers by Triamcinolone was not statistically significant.

### 3.6. Consistency and Sensitivity Analysis

We used ADDIS 1.16 software to help assess the consistency and inconsistency of the network meta-analysis. The consistency model, inconsistency model and node split model were adopted for full consideration. The results concluded that statistical heterogeneity within the study was non-existent, with no inconsistency observed in any of the four comparisons healing effect, size-reducing effect, symptom-reducing effect and adverse effect. The inconsistency between direct and indirect comparisons was achieved at the minimum possible level.

In terms of sensitivity analysis, both the method of rejecting literature piece by piece for sensitivity comparison and the method of subgroup analysis based on the time of outcome measurement were used. It can be considered that the optimization was achieved as much as possible.

### 3.7. Our Perspective

Efficacy and safety are two essential requirements for an intervention capable of being used. In this multi-study, multi-intervention study, we also used efficacy and safety as two important items of comparison. By comparison, we concluded that in terms of safety alone, all interventions included in the study were largely compliant, none exhibiting serious adverse events or hematologic effects. Therefore, efficacy was the first consideration in selecting the interventions. Considering healing promotion, pain reduction and relapse delay as evaluation indicators and, more importantly, the time dimension, we recommend laser as a short-term “shock therapy” intervention and probiotics as a long-term “conditioning” intervention.

## 4. Discussion

RAS has a self-healing property [99], but by the clinical characteristics of easy recurrence, local tissue loss, and pain [31], interventions are routinely used. However, because the etiology is not clear and the individual tendency is large, the treatment of RAS is limited to symptomatic treatment. Promoting healing, relieving pain and reducing recurrence are the goals of treatment [13]. In patients with minor RAS and major RAS not accompanied by systemic symptoms, local interventions are frequently used [100]. A wide variety of local interventions are available, many of which have demonstrated promising results in clinical trials relative to placebo. However, what is best and what better meets treatment expectations has not been determined. Therefore, there is value in assessing and comparing the efficacy of available topical interventions.

This is the first systematic evaluation and network meta-analysis on topical interventions for RAS, incorporating as many potential options as possible. In this study, both paired analyses and network comparisons were implemented. Descriptive analyses were also necessary for some outcome indicators with small sample sizes and different evaluation metrics. In total, 29 topical interventions were mentioned in this study, including placebo, allicin, aloe, amlexanox, benzydamine, berberine gelatin, chitosan chlorhexidine, clobetasol, cryotherapy, curcumin, dexamethasone, diosmectite, doxycycline, glycyrrhiza, honey, insulin-liposomal gel, laser, minocycline, N-acetylcysteine, penicillin, probiotics, prostaglandin E2, quercetin, silver nitrate, sucralfate, triamcinolone, triester glycerol oxide and zinc (clobetasol and minocycline were evaluated only in adverse events and were not involved in the primary outcome). To obtain as much data as possible, we searched four major databases, also supplemented by gray sources. We established clear inclusion and exclusion criteria, especially for the exclusion of traumatic ulcers, radiological ulcers and ulcers associated with systemic diseases. Seventy-two studies (5272 subjects) were included. We evaluated four efficacy and one safety outcome. The healing effect, size-reducing effect and symptom-reducing effect were the main bases of efficacy evaluation, and relapse was only analyzed descriptively. The safety aspect was more focused on adverse effects, and hematologic values were used as a complement. In addition, rank possibility based on *p*-score was the tool to help in the screening. 

As mentioned in the results, after combining the four efficacy evaluations and an additional safety evaluation, we propose our recommendation to clinicians: use laser as a short-term “shock therapy” during ulcer flare-ups, and use probiotics as a long-term “modifier” during the full ulcer cycle.

The laser, in our opinion, is an absolute landmark in clinical practice for the treatment of oral mucosal diseases. It is remarkable in common oral mucosal diseases and even precancerous lesions, such as oral mucositis due to radiotherapy and chemotherapy [101], oral submucosal fibrosis [102], oral lichen planus [103], oral leukoplakia [104] and burning mouth syndrome [105]. The effect of the laser on the oral mucosa has been interpreted as a stimulating biological effect [106]. Controlled laser light in a specific wavelength and power range is involved in local metabolic events through various physicochemical processes [107,108,109,110], thus exerting analgesic, anti-inflammatory and pro-repair effects without thermal damage. Certainly, the role in the treatment of RAS is gaining attention [111,112,113,114]. The types of lasers used in the current treatment are carbon dioxide laser, crystal laser, diode laser and low-level laser therapy (LLLT). In this evaluation, 14 RCTs with laser interventions were included. Five studies used diode lasers, six used CO2 lasers, one used Er, Cr: YSGG lasers, one used Nd: YAG lasers, and one used LLLT. The modality used, the wavelength of the laser and the power output were different. Whether the different types of laser interventions and the differences in the course and frequency have an impact on the efficacy is under-researched. Due to the scarcity of studies on individual kinds of lasers and the lack of treatment criteria, we grouped different kinds of laser modalities as laser in the study. In the evaluation of efficacy, lasers were considered superior in terms of healing effect, size reduction effect and symptom reduction effect during the full course of treatment. Moreover, in daily evaluations, the laser demonstrated unparalleled short-term explosive power. Laser is also unquestionable in terms of safety considerations. The latest retrospective evaluation was made by Valerie G. A. Suter and co-workers [111]. After reviewing 11 studies, they presented similar expectations for laser interventions in RAS and concerns regarding laser standardization. The advantages of the laser over other topical interventions are: (a) short treatment period: significant results with a single intervention or few interventions apart; (b) superior efficacy: excellent efficiency in promoting defect healing and reducing pain; and (c) reliable safety: several retrospective studies have found no significant safety concerns, including adverse events and hematologic testing. Therefore, laser is a worthwhile option for common cases, steroid-intolerant individuals, and patients with severe ulcers who are forced to undergo systemic treatment. Our recommendation is that laser is used as a short-term intervention during the exacerbation phase of ulcers to promote healing and reduce pain.

The oral cavity is a natural kingdom of microorganisms, and probiotics are one of the important components. The oral microbial community constructed with the participation of various probiotics is an indispensable and important member of oral microecology, which is involved in the balance with pathogenic bacteria through various potential links. This microscopic balance is considered to be essential for maintaining a healthy oral cavity [115,116]. Imbalance is dangerous and may lead to the occurrence of dental caries [117], periodontal disease [118], fungal disease [119], etc. Probiotics alone or supplemented with other drugs to modulate the composition and structure of the dominant flora in disease states, thus intervening in pathological states such as dental caries [120,121,122], periodontal disease [120,123,124] and breath odor [125,126] are supported by many studies. This also applies to RAS. Although the etiology of RAS is unclear, many microbiological and immunological studies have presented evidence for the involvement of microbial factors in the pathogenic process [10,127,128]. Probiotic therapy for RAS was thus pioneered and implemented in many practices [129]. In our study, probiotics were involved in the management of RAS in the form of topical administration. Four RCTs were performed with *Lactobacillus* and one with Bacillus Clausii probiotic. One was provided as a mouthwash with bacterial product (lactic acid) except for the others, which were solid tablets. The trial period was from 7 days to 90 days. In the early stage, probiotics did not show advantages in terms of healing effect, size reduction effect and symptom reduction effect. In the later stage (day 7), its efficacy in promoting healing began to appear. In the evaluation of recurrence, one study made a valuable contribution. Lotfy Aggour and co-workers [61] used L. acidophilus containing lozenges as an intervention agent for topical interventions in adult subjects and pediatric subjects compared to placebo. They counted the frequency of outbreaks in both groups over a 6-month follow-up period and concluded that the frequency was significantly lower in children using probiotics compared to controls, while the same effect was not seen in adults. More studies on recurrence are missing. The evaluation of safety is satisfactory. Possible mechanisms for the involvement of probiotics in RAS management are: (a) Competition mechanism: compared to pathogenic microorganisms in the oral cavity, probiotics have stronger surface adhesion as well as the ability to organize new aggregation and coaggregation processes [130,131,132]. This would lead to the loss of dominance of old pathogenic microorganisms and the establishment of new harmless or even beneficial biofilms. (b) Pro-repair effects: probiotics and their signaling molecules may help to reduce the levels of pro-inflammatory cytokines, collagenase, elastase and prostaglandin E2 through a series of mechanisms that promote the repair of local damage [133,134]. (c) Regulation of the microenvironment: metabolites and active molecules of probiotics, such as lactic acid, hydrogen peroxide and bacteriocins, and themselves may be involved in the regulation of the local physicochemical environment and immune environments, which facilitates the cessation of RAS progression and promotes tissue resistance and repair [131,133,135]. Based on these several effects, the microenvironment prone to RAS is regulated and is the main reason for the reduction of recurrent effects, and the limited ability to promote healing in the short term may be related to the insufficient number of metabolites and active substances. Our recommendation is that probiotics should be used as a long-term intervention in both the exacerbation and remission phases of ulcers to prolong the inter-episode interval and reduce recurrence.

There are some limitations to our study. First, RCTs on some interventions are scarce, which leads to the credibility of the study being compromised. Second, in our study, we combined different types of lasers, at different wavelengths, into one intervention category and therefore could not explore the differences between them. Studies and standards regarding this area are also lacking. Third, more studies on RAS recurrence are lacking. The evidence is weak for conclusions about probiotics based on non-direct evidence articles and a small number of clinical trials. High-quality studies that include more individuals and uniform criteria for evaluating outcomes are needed in the future.

## 5. Conclusions

This new consideration-based and comprehensive network meta-analysis concluded that most of the local interventions did not show significant differences in the efficacy evaluation and safety evaluation. Based on the currently available evidence, we recommend the use of laser as a short-term intervention to promote healing and reduce pain during the exacerbation phase of RAS and probiotics as a long-term intervention to prolong the inter-episode period and reduce recurrence during the exacerbation and remission phases of RAS. We call for more large-scale RCTs based on trustworthy standards.

## Figures and Tables

**Table 1:** Significantly different estimates for healing effect.

Healing Effect			
Comparison	Honey vs. placebo	Insulin-liposomal gel vs. placebo	Laser vs. placebo
Relative effect estimate	−3.55 (−5.90, −1.13)	−3.90 (−7.53, −0.23)	−3.08 (−4.81, −1.19)
Size-reducing Effect			
Comparison	Amlexanox vs. placebo	Glycyrrhiza vs. placebo	Triamcinolone vs. placebo
Relative effect estimate	35.29 (15.53, 54.72)	29.07 (3.58, 54.49)	25.83 (7.91, 45.48)
Symptom-Reducing Effect			
Comparison	Amlexanox vs. placebo	Laser vs. placebo	Triamcinolone vs. placebo
Relative effect estimate	23.26 (4.15, 42.15)	32.21 (16.39, 48.08)	28.45 (10.36, 46.76)
Adverse Effect			
Comparison	Triamcinolone vs. amlexanox	Triamcinolone vs. chitosan	Triamcinolone vs. dexamethasone
Relative effect estimate	0.00 (0.00, 0.00)	0.00 (0.00, 0.08)	0.00 (0.00, 0.00)
Comparison	Dexamethasone vs. penicillin	Placebo vs. doxycycline	Triamcinolone vs. doxycycline
Relative effect estimate	0.00 (0.00, 0.10)	0.00 (0.00, 0.06)	0.00 (0.00, 0.00)
Comparison	Triamcinolone vs. penicillin	Triamcinolone vs. placebo	Placebo vs. penicillin
Relative effect estimate	0.00 (0.00, 0.00)	0.00 (0.00, 0.00)	0.00 (0.00, 0.14)
Comparison	Dexamethasone vs. doxycycline		
Relative effect estimate	0.00 (0.00, 0.06)		

**Table 2:** Outcome *p*-cores for the best five interventions.

Healing Effect			Size-Reducing Effect			Symptom-Reducing Effect			Adverse Effect		
Rank	Treatment	*p*-Core	Rank	Treatment	*p*-Core	Rank	Treatment	*p*-Core	Rank	Treatment	*p*-Core
18	Insulin-liposomal gel	0.24	1	Quercetin	0.27	1	Insulin-liposomal gel	0.24	22	Triamcinolone	0.15
17	Honey	0.15	2	Dexamethasone	0.14	2	N-acetylcysteine or sucralfate	0.12	21	Berberine gelatin	0.08
16	Laser	0.11	3	Amlexanox	0.13	3	Curcumin	0.08	20	Glycyrrhiza or laser	0.07
15	Penicillin	0.09	4	Glycyrrhiza or laser	0.08	4	Laser	0.09	19	Aloe or honey or probiotics	0.06
14	Aloe	0.06	5	Curcumin	0.08	5	Chlorhexidine	0.07	18	Curcumin, silver nitrate, triester glycerol oxide or zinc	0.06

**Table 3:** Hematologic values.

Interventions	Total	Hematologic Values
Dexamethasone	114	Blood level < 0.502 ng/mL
Aloe	60	No significant differences between the blood test values before and after 7 days of application
Allicin	48	None of the hematologic values on day 6 were considered clinically abnormal
Amlexanox	108	None of the hematologic values were considered clinically abnormal

**Table 4:** Recurrence and statistical significance.

Interventions	Total	Relapse	Statistical Significance
Probiotics	Adult group: 30 Children group: 30	Adult group: (Outbreak frequency/6 months) Probiotics: 3.33 (0.64) Placebo: 3.65 (0.32) Children group: (Outbreak frequency/6 months) Probiotics: 2.65 (0.54) Placebo: 3.65 (0.62)	Adult group: No change in outbreak frequency was reported within the 6 months next to treatment (*p* > 0.05). Children group: A statistically significant decrease in outbreak frequency was reported for probiotics group within the 6 months next to treatment. The change was significantly different from placebo group (*p* < 0.05).
Chlorhexidine	38	Total ulcer numbers (6 weeks): Chlorhexidine: 7.54 ± 6.52 Placebo: 8.32 ± 5.52 Interval between ulcers (6 weeks): Chlorhexidine: 7.26 ± 8.61 Placebo:3.86 ± 2.05	Total ulcer numbers: NA Interval between ulcers: Chlorhexidine significantly increased the interval between successive ulcers (*p* < 0.05). For the total group, the increase was from 3.86 days with the placebo to 7.26 days with chlorhexidine.
Benzydamine	18	Number of new ulcers (3 months) Benzydamine: 7 (2–33) Placebo: 8 (2–20)	*p* = 0.07
Chlorhexidine	18	Number of new ulcers (3 months) Chlorhexidine: 6.5 (3–20) Placebo: 8 (2–20)	*p* = 0.27
Triamcinolone	26	No. of new ulcers (8 months) Placebo: 7.81 Triamcinolone acetonide in orabase: 7.00 Triamcinolone acetonide in watery base: 6.42	Although there was a slight reduction in the number of new ulcers during treatment with both steroid preparations, this was not statistically significant.

## Data Availability

All data generated or analyzed during this study were taken from the published study. The data are included in this article and supporting file.

## Supplementary Materials

The following supporting information can be downloaded at: https://www.mdpi.com/article/10.3390/medicina58060771/s1, Table S1: Search Strategy; Table S2–S5: Search terms of PubMed, Web of Science, Cochrane Central Register of Controlled Trials and EMBASE; Table S6: Characteristics of included studies; Table S7: Outcomes of included studies; Table S8–S10: Details information of studies for each outcome in network meta-analysis; Table S11: PRISMA NMA Checklist of Items; Chapter S1: Healing effect; Chapter S2: Size-reducing effect; Chapter S3: Symptom-reducing effect; Chapter S4: Safety outcomes; Chapter S5: Relapse; Chart S1: “Time-Rank 1 probability” folding line chart; Figure S1: Risk of bias graph; Figure S2: Risk of bias summary for individual studies; Figure S3: Funnel plot for healing effect; Figure S4: Funnel plot for size-reducing effect; Figure S5: Funnel plot for symptom-reducing effect; Figure S6: Funnel plot for adverse effect; Figure S7: Network structure of sensitivity analysis.
