# Supplementary material for: Efficacy of Topical Intervention for Recurrent Aphthous Stomatitis: A Network Meta-Analysis
Source: Medicina (Kaunas). 2022 Jun 7;58(6):771. doi: 10.3390/medicina58060771 (PMC9227309; doi:10.3390/medicina58060771)
Supplement: Supplementary file 1 [file medicina-58-00771-s001.zip › medicina-1747928-supplementary.pdf]

**Supporting information**

Efficacy of topical intervention for recurrent aphthous stomatitis: A network meta-analysis

Table S1 Search Strategy

Table S2-S5 Search terms of PubMed, Web of Science, Cochrane Central Register of Controlled Trials and EMBASE

Table S6 Characteristics of included studies

Table S7 Outcomes of included studies

Table S8-S10 Details information of studies for each outcome in network meta-analysis

Table S11 PRISMA NMA Checklist of Items

Chapter S1 Healing effect

Chapter S2 Size-reducing effect

Chapter S3 Symptom-reducing effect

Chapter S4 Safety outcomes

Chapter S5 Relapse

Chart S1 “Time-Rank 1 probability” folding line chart

FigureS1 Risk of bias graph

Figure S2 Risk of bias summary for individual studies

Figure S3 Funnel plot for healing effect

Figure S4 Funnel plot for size-reducing effect

Figure S5 Funnel plot for symptom-reducing effect

Figure S6 Funnel plot for adverse effect

Figure S7 Network structure of sensitivity analysis

**Table S1 Search Strategy**

| | |
|---|---|
| Review question | What is the efficacy and safety of various topical interventions in patients with recurrent aphthous stomatitis (all types) in relation to each other or in comparison to placebo? |
| Population | Clinical or histopathological examination confirms a diagnosis of recurrent aphthous ulcers, with ulcer-like lesions visible anywhere on the oral mucosa.<br>Simple ulcerative lesions of any undetermined cause such as psychological, nutritional and immunological factors, rather than oral manifestations of systemic diseases such as leukoaraiosis, diabetes mellitus, etc., or specific ulcerative lesions due to trauma, radiotherapy, etc.<br>The population enrolled received only local interventions or placebo during the trial and did not receive any other treatment that might alter the RAU prior to or during the trial, such as receiving systemic steroids or immunosuppressants.<br>For studies in patients with multiple oral mucosal diseases, we extracted only RAS data. If this was not possible, we excluded this study. |
| Subgroups | The following factors will be considered for Subgroups if heterogeneity is present:<br>Heterogeneity of single incorporated articles<br>Treatment course<br>Different RAS types<br>Drug dose<br>Forms of medication |
| Interventions | • Allicin<br>• Aloe<br>• Amlexanox<br>• Benzydamine<br>• Berberine gelatin<br>• Chitosan<br>• Chlorhexidine<br>• Clobetasol<br>• Cryotherapy<br>• Curcumin<br>• Dexamethasone<br>• Diosmectite<br>• Doxycycline<br>• Glycyrrhiza<br>• Honey<br>• Insulin-liposomal gel<br>• Laser<br>• Minocycline<br>• N-acetylcysteine<br>• Penicillin<br>• Probiotics<br>• Prostaglandin E2<br>• Quercetin |

| | |
|---|---|
| | • Silver nitrate<br>• Sucralfate<br>• Triamcinolone<br>• Triester Glycerol Oxide<br>• Zinc<br>(Note: all doses, forms and durations will be included) |
| Comparison | • Placebo<br>• Allicin<br>• Aloe<br>• Amlexanox<br>• Benzydamine<br>• Berberine gelatin<br>• Chitosan<br>• Chlorhexidine<br>• Clobetasol<br>• Cryotherapy<br>• Curcumin<br>• Dexamethasone<br>• Diosmectite<br>• Doxycycline<br>• Glycyrrhiza<br>• Honey<br>• Insulin-liposomal gel<br>• Laser<br>• Minocycline<br>• N-acetylcysteine<br>• Penicillin<br>• Probiotics<br>• Prostaglandin E2<br>• Quercetin<br>• Silver nitrate<br>• Sucralfate<br>• Triamcinolone<br>• Triester Glycerol Oxide<br>• Zinc |
| Outcomes | • Healing efficacy<br>Evaluated by healing time, the time elapsed between the start of the intervention and the healing of the ulcerated lesion.<br>• Effect of size reduction<br>Evaluated by the effectiveness index, EI=ulcer reduction area (mm2) / ulcer baseline area (mm2). The cumulative reduction in ulcer size at different examination days over the duration of the trial was counted.<br>• Effect of symptom reduction<br>Evaluated by the effectiveness index, EI=reduction in pain score / baseline ulcer pain |

| | |
|---|---|
| | score. Individual subject's VAS score or decile scale score of pain level on different examination days. The degree of cumulative pain relief was calculated.<br>VAS score: 10 cm horizontal line, marked 0 = no pain to 10 = worst pain.<br>Decile scale: 0 for no pain, 10 for most pain.<br>• Safety outcomes<br>Adverse effect<br>Hematologic values<br>• Relapse |
| Study design | RCTs |
| Databases | PubMed, Web of Science (WOS), Cochrane Central Register of Controlled Trials, Embase |

## Table S2 Final search strategy for PubMed

| #ID | Topic or intervention | | Query | Records |
|---|---|---|---|---|
| #1 | Recurrent Aphthous Stomatitis | | ("Stomatitis, Aphthous" [Mesh]) OR ("Oral Ulcer" [Mesh]) OR ((((((((Aphth* [Title/Abstract]) OR Canker sore [Title/Abstract]) OR Oral ulcer* [Title/Abstract]) OR Mouth ulcer* [Title/Abstract]) OR Recurrent aphthous ulcer* [Title/Abstract]) OR Recurrent aphthous stomatitis [Title/Abstract]) OR Aphthous stomatitis [Title/Abstract]) | 9988 |
| #2 | Clobetasol | | ("Clobetasol" [Mesh]) OR (Clobetasol [Title/Abstract]) OR (Corticosteroid [Title/Abstract]) OR (Clobetasol Propionate [Title/Abstract]) | 53558 |
| #3 | Betamethasone | | ("Betamethasone" [Mesh]) OR ("Betamethasone Valerate" [Mesh]) OR (Betamethasone [Title/Abstract]) OR (Betasolon [Title/Abstract]) OR ( Betamethasone Valerate [Title/Abstract]) | 9273 |
| #4 | Dexamethasone | | ("Dexamethasone" [Mesh]) OR (Dexamethasone [Title/Abstract]) OR (Dexamucobase [Title/Abstract]) | 75058 |
| #5 | Triamcinolone | | ("Triamcinolone" [Mesh]) OR ("Triamcinolone Acetonide" [Mesh]) OR (Triamcinolone [Title/Abstract]) OR (Triamcinolone Acetonide [Title/Abstract]) | 12218 |
| #6 | Minocycline | | ("Minocycline" [Mesh]) OR (Minocycline [Title/Abstract]) | 9428 |
| #7 | Doxycycline | | ("Doxycycline" [Mesh]) OR (Doxycycline [Title/Abstract]) | 18183 |
| #8 | Penicillin | | ("Penicillin G"[Mesh]) OR ("Penicillin"[Mesh]) (Penicillin [Title/Abstract]) OR (Penicillin G Potassium [Title/Abstract]) OR (Penicillin G [Title/Abstract]) | 105884 |
| #9 | Benzydamine | | ("Benzydamine" [Mesh]) OR (Benzydamine [Title/Abstract]) | 597 |
| #10 | Amlexanox | | Amlexanox[Title/Abstract] | 151 |
| #11 | Chlorhexidine | | ("Chlorhexidine" [Mesh]) OR (Chlorhexidine [Title/Abstract]) OR (Hibitane [Title/Abstract]) | 13254 |
| #12 | Hyaluronic Acid | | ("Hyaluronic Acid " [Mesh]) OR (Hyaluronic Acid [Title/Abstract]) | 31587 |
| #13 | Laser | CO2<br>Nd:YAG<br>diode | ("Laser Therapy"[Mesh]) OR ("Lasers"[Mesh]) OR (Low level laser therapy [Title/Abstract]) OR (Lasertherap* [Title/Abstract]) OR (LLLT* [Title/Abstract]) OR (Laser* [Title/Abstract]) | 308558 |
| #14 | Sodium lauryl sulfate | | (Sodium lauryl sulfate dentifrice[Title/Abstract]) OR (Sodium lauryl sulfate toothpaste[Title/Abstract]) | 104 |

| | | | |
|---|---|---|---|
| #15 | Probiotics | ("probiotics" [Mesh]) OR ("Lactobacillales" [Mesh]) OR ("Lactococcus" [Mesh]) OR ("Bifidobacterium" [Mesh]) OR (probiotic* [Title/Abstract]) OR (lactobacilli [Title/Abstract]) OR (Lactobacillales [Title/Abstract]) OR (Lactococcus [Title/Abstract]) OR (Bifidobacteri* [Title/Abstract]) | 166173 |
| #16 | Insulin-liposomal gel | Insulin-liposomal gel [Title/Abstract] | 1 |
| #17 | Salicept | Salicept [Title/Abstract] | 4 |
| #18 | HybenX | HybenX [Title/Abstract] | 17 |
| #19 | Cryotherapy | ("Cryotherapy" [Mesh]) OR (Cryotherapy [Title/Abstract]) | 31784 |
| #20 | Prostaglandin E2 | ("Dinoprostone" [Mesh]) OR (Prostaglandin E2 [Title/Abstract]) | 37890 |
| #21 | Curcumin | ("Curcumin" [Mesh]) OR (Curcumin [Title/Abstract]) | 17505 |
| #22 | Glycyrrhiza | ("Glycyrrhiza" [Mesh]) OR (Glycyrrhiza [Title/Abstract]) | 3809 |
| #23 | Quercetin | ("Quercetin" [Mesh]) OR (Quercetin [Title/Abstract]) | 21546 |
| #24 | Chitosan | ("Chitosan" [Mesh]) OR (Chitosan [Title/Abstract]) | 33883 |
| #25 | Aloe | ("Aloe" [Mesh]) OR (“acemannan” [Supplementary Concept]) OR (Aloe [Title/Abstract]) OR (acemannan [Title/Abstract]) | 3217 |
| #26 | Honey | ("Honey" [Mesh]) OR (Honey [Title/Abstract]) | 12937 |
| #27 | lavender oil | ("lavender oil" [Supplementary Concept]) OR (lavender oil[Title/Abstract]) | 442 |
| #28 | Berberine gelatin | Berberine gelatin [Title/Abstract] | 1 |
| #29 | Diosmectite | ("Smectite" [Supplementary Concept]) OR (Diosmectite [Title/Abstract]) | 309 |
| #30 | Allicin | ("allicin" [Supplementary Concept]) OR ( Allicin [Title/Abstract]) | 900 |
| #31 | N-acetylcysteine | (" Acetylcysteine" [Mesh]) OR (N-acetylcysteine [Title/Abstract]) | 19573 |
| #32 | Sucralfate | ("Sucralfate" [Mesh]) OR (Sucralfate [Title/Abstract]) | 2082 |
| #33 | Triester Glycerol Oxide | Triester Glycerol Oxide [Title/Abstract] | 5 |
| #34 | Cautery: Silver nitrate<br>Debacterol | ("Cautery" [Mesh]) OR (Silver nitrate [Title/Abstract]) OR (Debacterol [Title/Abstract]) OR ( Cautery [Title/Abstract]) | 20040 |
| #35 | Zinc | ("Zinc" [Mesh]) OR (Zinc [Title/Abstract]) | 146109 |
| #36 | Final query | #1 AND (#2 OR #3 OR #4 OR #5 OR #6 OR #7 OR #8 OR #9 OR #10 OR #11 OR #12 OR #13 OR #14 | 902 |

| | | | |
|---|---|---|---|
| | | OR #15 OR #16 OR #17 OR #18 OR #19 OR #20 OR #21 OR #22 OR #23 OR #24 OR #25 OR #26 OR #27 OR #28 OR #29 OR #30 OR #31 OR #32 OR #33 OR #34 OR #35 OR #36) | |

**Table S3 Final search strategy for Web of Science**

| #ID | Topic or intervention | Query | Records |
|---|---|---|---|
| #1 | Recurrent Aphthous Stomatitis | (TS=(Aphth*) OR TS=(Canker sore) OR TS=(Oral ulcer*) OR TS=(Mouth ulcer*) OR TS=( Recurrent aphthous ulcer*) OR TS=( Recurrent aphthous stomatitis) OR TS=( Aphthous stomatitis)) | 47159 |
| #2 | Clobetasol | (TS=(Clobetasol) OR TS=(Corticosteroid) OR TS=(Clobetasol Propionate)) | 163969 |
| #3 | Betamethasone | (TS=(Betamethasone) OR TS=(Betasolon) OR TS=( Betamethasone Valerate)) | 10781 |
| #4 | Dexamethasone | (TS=(Dexamethasone) OR TS=( Dexamucobase)) | 112334 |
| #5 | Triamcinolone | (TS=(Triamcinolone) OR TS=(Triamcinolone Acetonide)) | 18064 |
| #6 | Minocycline | (TS=(Minocycline)) | 13720 |
| #7 | Doxycycline | (TS=(Doxycycline)) | 28374 |
| #8 | Penicillin | (TS=(Penicillin) OR TS=( Penicillin G Potassium) OR TS=( Penicillin G)) | 143392 |
| #9 | Benzydamine | (TS=(Benzydamine)) | 803 |
| #10 | Amlexanox | (TS=(Amlexanox)) | 242 |
| #11 | Chlorhexidine | (TS=(Chlorhexidine) OR TS=(Hibitane)) | 18711 |
| #12 | Hyaluronic Acid | (TS=(Hyaluronic Acid)) | 46592 |
| #13 | Laser<br>CO2<br>Nd:YAG<br>diode | (TS=(Laser Therapy) OR TS=(Low level laser therapy) OR TS=(Lasertherap*) OR TS=(LLLT*) OR TS=(Laser*)) | 924745 |
| #14 | Sodium lauryl sulfate | (TS=(Sodium lauryl sulfate dentifrice) OR TS=( Sodium lauryl sulfate toothpaste)) | 161 |
| #15 | Probiotics | (TS=(probiotic*) OR TS=(lactobacilli) OR TS=(Lactobacillales) OR TS=(Lactococcus) OR TS=(Bifidobacteri*)) | 187354 |
| #16 | Insulin-liposomal gel | (TS=(Insulin-liposomal gel)) | 1 |
| #17 | Salicept | (TS=(Salicept)) | 11 |
| #18 | HybenX | (TS=(HybenX)) | 19 |
| #19 | Cryotherapy | (TS=(Cryotherapy)) | 15196 |
| #20 | Prostaglandin E2 | (TS=(Prostaglandin E2) OR TS=(Dinoprostone)) | 51029 |
| #21 | Curcumin | (TS=(Curcumin)) | 30010 |
| #22 | Glycyrrhiza | (TS=(Glycyrrhiza)) | 7474 |
| #23 | Quercetin | (TS=(Quercetin)) | 40563 |
| #24 | Chitosan | (TS=(Chitosan)) | 89698 |
| #25 | Aloe | (TS=( Aloe) OR TS=(acemannan)) | 7631 |
| #26 | Honey | (TS=(Honey)) | 43877 |
| #27 | lavender oil | (TS=( lavender oil)) | 2124 |
| #28 | Berberine gelatin | (TS=(Berberine gelatin)) | 27 |
| #29 | Diosmectite | (TS=(Diosmectite)) | 70 |

| | | | | |
|---|---|---|---|---|
| #30 | Allicin | | (TS=(Allicin)) | 1998 |
| #31 | N-acetylcysteine | | (TS=(N-acetylcysteine)) | 21589 |
| #32 | Sucralfate | | (TS=(Sucralfate)) | 2962 |
| #33 | Triester Glycerol Oxide | | (TS=(Triester Glycerol Oxide)) | 10 |
| #34 | Cautery | Silver nitrate | (TS=(Cautery) OR TS=(Silver nitrate) OR TS=(Debacterol)) | 21048 |
| | | Debacterol | | |
| #35 | Zinc | | (TS=(Zinc)) | 509430 |
| #36 | Final query | | #1 AND (#2 OR #3 OR #4 OR #5 OR #6 OR #7 OR #8 OR #9 OR #10 OR #11 OR #12 OR #13 OR #14 OR #15 OR #16 OR #17 OR #18 OR #19 OR #20 OR #21 OR #22 OR #23 OR #24 OR #25 OR #26 OR #27 OR #28 OR #29 OR #30 OR #31 OR #32 OR #33 OR #34 OR #35) | 6303 |

**Table S4 Final search strategy for Cochrane Central Register of Controlled Trials**

| #ID | Topic or intervention | Query | Records |
|---|---|---|---|
| #1 | Recurrent Aphthous Stomatitis | MeSH descriptor: [Stomatitis, Aphthous] explode all trees | 242 |
| #2 | | MeSH descriptor: [Oral Ulcer] explode all trees | 65 |
| #3 | | (Aphth*):ti,ab,kw | 774 |
| #4 | | (Canker sore):ti,ab,kw | 16 |
| #5 | | (Oral ulcer*):ti,ab,kw | 4827 |
| #6 | | (Mouth ulcer*):ti,ab,kw | 929 |
| #7 | | (Recurrent aphthous ulcer*):ti,ab,kw | 342 |
| #8 | | (Recurrent aphthous stomatitis):ti,ab,kw | 364 |
| #9 | | (Aphthous stomatitis):ti,ab,kw | 555 |
| #10 | | #1 OR #2 OR #3 OR #4 OR #5 OR #6 OR #7 OR #8 OR #9 | 5645 |
| #11 | Clobetasol | MeSH descriptor: [Clobetasol] explode all trees | 382 |
| #12 | | (Clobetasol):ti,ab,kw OR (Corticosteroid):ti,ab,kw OR (Clobetasol Propionate):ti,ab,kw | 23575 |
| #13 | | #11 OR #12 | 23575 |
| #14 | Betamethasone | MeSH descriptor: [Betamethasone] explode all trees | 1492 |
| #15 | | MeSH descriptor: [Betamethasone Valerate] explode all trees | 352 |
| #16 | | (Betamethasone):ti,ab,kw OR (Betasolon):ti,ab,kw OR (Betamethasone Valerate):ti,ab,kw | 2570 |
| #17 | | #14 OR #15 OR #16 | 2846 |
| #18 | Dexamethasone | MeSH descriptor: [Dexamethasone] explode all trees | 4794 |
| #19 | | (Dexamethasone):ti,ab,kw OR (Dexamucobase):ti,ab,kw | 12687 |
| #20 | | #18 OR #19 | 12708 |
| #21 | Triamcinolone | MeSH descriptor: [Triamcinolone] explode all trees | 1448 |
| #22 | | MeSH descriptor: [Triamcinolone Acetonide] explode all trees | 1138 |
| #23 | | (Triamcinolone):ti,ab,kw OR (Triamcinolone Acetonide):ti,ab,kw | 3320 |
| #24 | | #21 OR #22 OR #23 | 3320 |
| #25 | Minocycline | MeSH descriptor: [Minocycline] explode all trees | 544 |
| #26 | | (Minocycline):ti,ab,kw | 1126 |
| #27 | | #25 OR #26 | 1126 |
| #28 | Doxycycline | MeSH descriptor: [Doxycycline] explode all trees | 1089 |
| #29 | | (Doxycycline):ti,ab,kw | 2223 |
| #30 | | #28 OR #29 | 2223 |
| #31 | Penicillin | MeSH descriptor: [Penicillin G] explode all trees | 4719 |

| | | | | |
|---|---|---|---|---|
| #32 | | | (Penicillin):ti,ab,kw OR (Penicillin G Potassium):ti,ab,kw OR (penicillin G):ti,ab,kw | 3529 |
| #33 | | | #31 OR #32 | 7005 |
| #34 | Benzydamine | | MeSH descriptor: [Benzydamine] explode all trees | 104 |
| #35 | | | (Benzydamine):ti,ab,kw | 258 |
| #36 | | | #34 OR #35 | 258 |
| #37 | Amlexanox | | (Amlexanox):ti,ab,kw | 27 |
| #38 | Chlorhexidine | | MeSH descriptor: [Chlorhexidine] explode all trees | 2296 |
| #39 | | | (Chlorhexidine):ti,ab,kw OR (Hibitane):ti,ab,kw | 5022 |
| #40 | | | #38 OR #39 | 5022 |
| #41 | Hyaluronic Acid | | MeSH descriptor: [Hyaluronic Acid] explode all trees | 1722 |
| #42 | | | (Hyaluronic Acid):ti,ab,kw | 3421 |
| #43 | | | #41 OR #42 | 3421 |
| #44 | Laser | $CO_2$、Nd:YAG、diode | MeSH descriptor: [Laser Therapy] explode all trees | 4309 |
| #45 | | | MeSH descriptor: [Lasers] explode all trees | 2308 |
| #46 | | | (Low level laser therapy):ti,ab,kw OR (Lasertherap*):ti,ab,kw OR (LLLT*):ti,ab,kw OR (Laser*):ti,ab,kw | 20404 |
| #47 | | | #44 OR #45 OR #46 | 20509 |
| #48 | Sodium lauryl sulfate | | (Sodium lauryl sulfate dentifrice):ti,ab,kw OR (Sodium lauryl sulfate toothpaste):ti,ab,kw | 52 |
| #49 | Probiotics | | MeSH descriptor: [Probiotics] explode all trees | 2208 |
| #50 | | | MeSH descriptor: [Lactobacillales] explode all trees | 3480 |
| #51 | | | MeSH descriptor: [Lactococcus] explode all trees | 16 |
| #52 | | | MeSH descriptor: [Bifidobacterium] explode all trees | 759 |
| #53 | | | (probiotic*):ti,ab,kw OR (lactobacilli):ti,ab,kw OR (Lactobacillales):ti,ab,kw OR (Lactococcus):ti,ab,kw OR (Bifidobacteri*):ti,ab,kw | 9131 |
| #54 | | | #49 OR #50 OR #51 OR #52 OR #53 | 11104 |
| #55 | Insulin-liposomal gel | | (Insulin-liposomal gel):ti,ab,kw | 1 |
| #56 | Salicept | | (Salicept):ti,ab,kw | 2 |
| #57 | HybenX | | (HybenX):ti,ab,kw | 9 |
| #58 | Cryotherapy | | MeSH descriptor: [Cryotherapy] explode all trees | 1669 |
| #59 | | | (Cryotherapy):ti,ab,kw | 2291 |
| #60 | | | #58 OR #59 | 3231 |
| #61 | Prostaglandin E2 | | MeSH descriptor: [Dinoprostone] explode all trees | 1164 |
| #62 | | | (Prostaglandin E2):ti,ab,kw | 1994 |
| #63 | | | #61 OR #62 | 2495 |
| #64 | Curcumin | | MeSH descriptor: [Curcumin] explode all trees | 437 |
| #65 | | | (Curcumin):ti,ab,kw | 1277 |

| | | | |
|---|---|---|---|
| #66 | | #64 OR #65 | 1277 |
| #67 | Glycyrrhiza | MeSH descriptor: [Glycyrrhiza] explode all trees | 105 |
| #68 | | (Glycyrrhiza):ti,ab,kw | 351 |
| #69 | | #67 OR #68 | 351 |
| #70 | Quercetin | MeSH descriptor: [Quercetin] explode all trees | 205 |
| #71 | | (Quercetin):ti,ab,kw | 518 |
| #72 | | #70 OR #71 | 518 |
| #73 | Chitosan | MeSH descriptor: [Quercetin] explode all trees | 205 |
| #74 | | (Chitosan):ti,ab,kw | 480 |
| #75 | | #73 OR #74 | 685 |
| #76 | Aloe | MeSH descriptor: [Aloe] explode all trees | 681 |
| #77 | | (Aloe):ti,ab,kw OR (acemannan):ti,ab,kw | 81 |
| #78 | | #76 OR #77 | 681 |
| #79 | Honey | MeSH descriptor: [Honey] explode all trees | 163 |
| #80 | | (honey):ti,ab,kw | 1129 |
| #81 | | #79 OR #80 | 1129 |
| #82 | Lavender oil | (lavender oil):ti,ab,kw | 524 |
| #83 | Berberine gelatin | (Berberine gelatin):ti,ab,kw | 1 |
| #84 | Diosmectite | (Diosmectite):ti,ab,kw | 30 |
| #85 | Allicin | (Allicin):ti,ab,kw | 50 |
| #86 | N-acetylcysteine | MeSH descriptor: [Acetylcysteine] explode all trees | 1155 |
| #87 | | (N-acetylcysteine):ti,ab,kw | 1804 |
| #88 | | #86 OR #87 | 2144 |
| #89 | Sucralfate | MeSH descriptor: [Sucralfate] explode all trees | 398 |
| #90 | | (Sucralfate):ti,ab,kw | 798 |
| #91 | | #89 OR #90 | 798 |
| #92 | Triester Glycerol Oxide | (Triester Glycerol Oxide):ti,ab,kw | 6 |
| #93 | Cautery<br>Silver nitrate、Debacterol | MeSH descriptor: [Cautery] explode all trees | 805 |
| #94 | | (Cautery):ti,ab,kw OR (Silver nitrate):ti,ab,kw OR (Debacterol):ti,ab,kw | 627 |
| #95 | | #93 OR #94 | 1309 |
| #96 | Zinc | MeSH descriptor: [Zinc] explode all trees | 1677 |
| #97 | | (Zinc):ti,ab,kw | 8083 |
| #98 | | #96 OR #97 | 8083 |
| #99 | Final query | #10 AND (#13 OR #17 OR #20 OR #24 OR #27 OR #30 OR #33 OR #36 OR #37 OR #40 OR #43 OR #47 OR #48 OR #54 OR #55 OR # 56 OR #57 OR #60 OR #63 OR #66 OR #69 OR #72 OR #75 OR #78 OR #81 OR #82 OR #83 OR #84 OR #85 OR #88 OR #91 OR #92 OR #95 OR #98) | 1560 |
| | Cochrane Reviews | | 69 |
| | Trials | | 1489 |

**Table S5 Final search strategy for Embase**

| #ID | Topic or intervention | Query | Records |
|---|---|---|---|
| #1 | Recurrent Aphthous Stomatitis | 'mouth ulcer'/exp OR 'aphthous stomatitis'/exp | 19837 |
| #2 | | aphth*:ti,ab,kw OR 'canker sore':ti,ab,kw OR 'oral ulcer*':ti,ab,kw OR 'mouth ulcer*':ti,ab,kw OR 'recurrent aphthous ulcer*':ti,ab,kw OR 'recurrent aphthous stomatitis':ti,ab,kw OR 'aphthous stomatitis':ti,ab,kw | 11455 |
| #3 | | #1 OR #2 | 23211 |
| #4 | Clobetasol | 'clobetasol'/exp | 3164 |
| #5 | | (Clobetasol):ti,ab,kw OR (Corticosteroid):ti,ab,kw OR (Clobetasol Propionate):ti,ab,kw | 78877 |
| #6 | | #4 AND #5 | 81186 |
| #7 | Betamethasone | 'betamethasone'/exp OR 'betamethasone valerate'/exp | 21527 |
| #8 | | betamethasone:ti,ab,kw OR betasolon:ti,ab,kw OR 'betamethasone valerate':ti,ab,kw | 7514 |
| #9 | | #7 OR #8 | 23136 |
| #10 | Dexamethasone | 'dexamethasone'/exp | 164002 |
| #11 | | dexamethasone:ti,ab,kw OR dexamucobase:ti,ab,kw | 83821 |
| #12 | | #10 OR #11 | 175940 |
| #13 | Triamcinolone | 'triamcinolone'/exp OR 'triamcinolone acetonide'/exp | 30533 |
| #14 | | triamcinolone:ti,ab,kw OR 'triamcinolone acetonide':ti,ab,kw | 11046 |
| #15 | | #13 OR #14 | 31894 |
| #16 | Minocycline | 'minocycline'/exp | 25639 |
| #17 | | minocycline:ti,ab,kw | 9918 |
| #18 | | #16 OR #17 | 26613 |
| #19 | Doxycycline | 'doxycycline'/exp | 57207 |
| #20 | | doxycycline:ti,ab,kw | 21704 |
| #21 | | #19 OR #20 | 59886 |
| #22 | Penicillin | 'penicillin g'/exp | 83154 |
| #23 | | penicillin:ti,ab,kw OR 'penicillin g potassium':ti,ab,kw OR 'penicillin g':ti,ab,kw | 62134 |
| #24 | | #22 OR #23 | 121598 |
| #25 | Benzydamine | 'benzydamine'/exp | 1564 |
| #26 | | benzydamine:ti,ab,kw | 707 |
| #27 | | #25 OR #26 | 1710 |
| #28 | Amlexanox | amlexanox:ti,ab,kw | 211 |
| #29 | Chlorhexidine | 'chlorhexidine'/exp | 18967 |
| #30 | | chlorhexidine:ti,ab,kw OR hibitane:ti,ab,kw | 13927 |

| | | | | |
|---|---|---|---|---|
| #31 | | | #29 OR #30 | 23175 |
| #32 | Hyaluronic Acid | | 'hyaluronic acid'/exp | 46079 |
| #33 | | | 'hyaluronic acid':ti,ab,kw | 26672 |
| #34 | | | #32 OR #33 | 50728 |
| #35 | Laser | CO2、Nd:YAG、diode | 'low level laser therapy'/exp OR 'laser'/exp | 172779 |
| #36 | | | 'low level laser therapy':ti,ab,kw OR lasertherap*:ti,ab,kw OR lllt*:ti,ab,kw OR laser*:ti,ab,kw | 304449 |
| #37 | | | #35 OR #36 | 335329 |
| #38 | Sodium lauryl sulfate | | (Sodium lauryl sulfate dentifrice):ti,ab,kw OR (Sodium lauryl sulfate toothpaste):ti,ab,kw | 0 |
| #39 | Probiotics | | 'probiotic agent'/exp OR 'lactobacillales'/exp OR 'lactococcus'/exp OR 'bifidobacterium'/exp | 272586 |
| #40 | | | probiotic*:ti,ab,kw OR lactobacilli:ti,ab,kw OR lactobacillales:ti,ab,kw OR lactococcus:ti,ab,kw OR bifidobacteri*:ti,ab,kw | 59391 |
| #41 | | | #39 OR #40 | 279556 |
| #42 | Insulin-liposomal gel | | 'insulin-liposomal gel':ti,ab,kw | 1 |
| #43 | Salicept | | salicept:ti,ab,kw | 6 |
| #44 | HybenX | | hybenx:ti,ab,kw | 13 |
| #45 | Cryotherapy | | 'cryotherapy'/exp | 37910 |
| #46 | | | cryotherapy:ti,ab,kw | 11654 |
| #47 | | | #45 OR #46 | 39784 |
| #48 | Prostaglandin E2 | | 'prostaglandin e2'/exp | 57910 |
| #49 | | | 'prostaglandin e2':ti,ab,kw | 10531 |
| #50 | | | #48 OR #49 | 60453 |
| #51 | Curcumin | | 'curcumin'/exp | 28319 |
| #52 | | | curcumin:ti,ab,kw | 21431 |
| #53 | | | #51 OR #52 | 29950 |
| #54 | Glycyrrhiza | | 'glycyrrhiza'/exp | 6231 |
| #55 | | | glycyrrhiza:ti,ab,kw | 2858 |
| #56 | | | #54 OR #55 | 7108 |
| #57 | Quercetin | | 'quercetin'/exp | 34523 |
| #58 | | | quercetin:ti,ab,kw | 25917 |
| #59 | | | #57 OR #58 | 40873 |
| #60 | Chitosan | | 'chitosan'/exp | 36676 |
| #61 | | | chitosan:ti,ab,kw | 39460 |
| #62 | | | #60 OR #61 | 44767 |
| #63 | Aloe | | 'aloe'/exp OR 'acemannan'/exp | 4031 |
| #64 | | | aloe:ti,ab,kw OR acemannan:ti,ab,kw | 4626 |
| #65 | | | #63 OR #64 | 5954 |
| #66 | Honey | | 'honey'/exp | 8250 |
| #67 | | | honey:ti,ab,kw | 14894 |

| | | | |
|---|---|---|---|
| #68 | | #66 OR #67 | 16060 |
| #69 | Lavender oil | 'lavender oil'/exp | 1259 |
| #70 | | 'lavender oil':ti,ab,kw | 456 |
| #71 | | #69 OR #70 | 1337 |
| #72 | Berberine gelatin | 'berberine gelatin':ti,ab,kw | 1 |
| #73 | Diosmectite | 'diosmectite'/exp | 44 |
| #74 | | diosmectite:ti,ab,kw | 53 |
| #75 | | #73 OR #74 | 76 |
| #76 | Allicin | 'allicin'/exp | 1573 |
| #77 | | allicin:ti,ab,kw | 1144 |
| #78 | | #76 OR #77 | 1755 |
| #79 | N-acetylcysteine | 'acetylcysteine'/exp | 38564 |
| #80 | | 'n acetylcysteine':ti,ab,kw | 15862 |
| #81 | | #79 OR #80 | 40501 |
| #82 | Sucralfate | 'sucralfate'/exp | 7121 |
| #83 | | sucralfate:ti,ab,kw | 2542 |
| #84 | | #82 OR #83 | 7373 |
| #85 | Triester Glycerol Oxide | 'triester glycerol oxide':ti,ab,kw | 9 |
| #86 | Cautery / Silver nitrate、Debacterol | 'cauterization'/exp | 15544 |
| #87 | | cautery:ti,ab,kw OR 'silver nitrate':ti,ab,kw OR debacterol:ti,ab,kw | 9652 |
| #88 | | #86 OR #87 | 22264 |
| #89 | Zinc | 'zinc'/exp | 125700 |
| #90 | | zinc:ti,ab,kw | 148781 |
| #91 | | #89 OR #90 | 206697 |
| #92 | Final query | #6 OR #9 OR #12 OR #15 OR #18 OR #21 OR #24 OR #27 OR #28 OR #31 OR #34 OR #37 OR #38 OR #41 OR #42 OR #43 OR #44 OR #47 OR #50 OR #53 OR #56 OR #59 OR #62 OR #65 OR #68 OR #71 OR #72 OR #75 OR #78 OR #81 OR #84 OR #85 OR #88 OR #91 | 1604834 |
| #93 | | #3 AND #92 | 3268 |

## Table S6 Characteristics of included studies

| Author Year | Country | Type of study | Type of RAS | Patients | | Comparison | |
|---|---|---|---|---|---|---|---|
| | | | | Male/Female | Age | Intervention (N) | Treatment Course |
| Huo<br>2021 | China | RCT | minor aphthous ulcer | 8/17<br>9/17 | Under 40:16 Above 40:9<br>Under 40:13 Above 40:13 | diode laser 810 nm (25)<br>triamcinolone acetonide 0.1% (26) | once daily for continuous 3 days<br>three times a day until the lesion was healed |
| Aggour (audlt)<br>2021 | Egypt | RCT | minor RAS | 11/19<br>10/20 | 28.82±8.44<br>29.38±9.06 | ChocBalls (L. acidophilus containing lozenges) (30)<br>Oracure oral gel (30) | melt in the mouth slowly twice daily for 5 days<br>melt in the mouth slowly twice daily for 5 days |
| Aggour (child)<br>2021 | Egypt | RCT | minor RAS | 12/18<br>14/16 | 6.82±2.44<br>7.18±1.96 | ChocBalls (L. acidophilus containing lozenges) (30)<br>Oracure oral gel (30) | melt in the mouth slowly twice daily for 5 days<br>melt in the mouth slowly twice daily for 5 days |
| Shi<br>2020 | China | RCT | NA | 8/12<br>4/11 | 30.5±26.6<br>26.07±56.44 | aloe vera fermentation gel (20)<br>chitosan gel (15) | apply a layer of gel on the surface of the ulcer every day after each meal (three times each day) until the ulcer disappeared<br>apply a layer of gel on the surface of the ulcer every day after each meal (three times each day) until the ulcer disappeared |
| Kavita<br>2020 | India | RCT | NA | NA | 18-48 | 0.1% topical triamcinolone acetonide (30)<br>5% topical amlexanox (30) | apply paste on the ulcers three to four times per day for a minimum of 10 min<br>apply paste on the ulcers three to four times per day for a minimum of 10 min |
| Shao<br>2020 | China | RCT | NA | 15/19<br>17/15 | 31.62±8.45<br>28.72±6.23 | film containing chitosan (34)<br>polyvinyl alcohol film (32) | twice a day (after breakfast and after dinner). The lasting time of the film is about 1 h. Avoid drinking or eating for 1 h after using the film<br>twice a day (after breakfast and after dinner). The lasting time of the film is about 1 h. Avoid drinking or eating for 2 h after using the film |

| | | | | | | | |
|---|---|---|---|---|---|---|---|
| Pedersen | Denmark | RCT | minor or major | 2/8 | 22.7 (18-28) | test lozenges contained a mix of two probiotic strains, Lactobacillus reuteri DSM17938 and ATCC PTA 5289 (10) | take two lozenges per day (morning and evening) for 90 days |
| 2020 | | | | 5/5 | 24.8 (22-30) | placebo lozenges without bacteria (10) | take two lozenges per day (morning and evening) for 90 days |
| Ghorbani | Iran | RCT | minor aphthous ulcers | 10/13 | 38.66±21.60 | mucoadhesive tablet with zinc sulfate (23) | 3 times a day. The duration of the intervention was 7 days |
| 2020 | | | | 9/14 | 41.28±24.37 | placebo without zinc sulfate (23) | 3 times a day. The duration of the intervention was 7 days |
| Ibrahim | Egypt | RCT | NA | 6/14 | 36.60±11.50 | Kenalog in Orabase contained triamcinolone acetonide (20) | two times per day, once in the morning and the other at bed time, after meals for 1 or 2 weeks according to patient's clinical response |
| 2020 | | | | 8/12 | 35.10±13.00 | lactic acid 5% mouth wash (20) | three times daily before meals and leave the solution in their mouth for 3-5 minutes, then spit it. They used the mouth wash for 1-2 weeks according to patient's clinical response |
| Kia | Iran | RCT | NA | 20/9 | 43.72±9.62 | 5% curcumin (29) | three times a day for 10 days |
| 2020 | | | | 16/13 | 45.05±8.90 | 0.1% triamcinolone (29) | three times a day for 10 days |
| Owlia | Iran | RCT | acute-onset oral aphthae with diameter ≥5 mm | 12/13 | 29.92 (15-45) | Penicillin (25) | four times a day for a week |
| 2020 | | | | 14/11 | 31.32 (15-45) | placebo (25) | four times a day for a week |
| Seyyedi | Iran | RCT | Minor aphthous stomatitis | 2/13 | 34.54±14.93 | pulsed carbon dioxide laser (5) | NA |
| 2020 | | | | | | continuous carbon dioxide laser (5) | NA |
| | | | | | | 0.1% triamcinolone acetonide (5) | NA |
| Raman | India | RCT | minor aphthous ulcer | 11/19 | 21.3 | curcumin (30) | three times daily until your pain completely |

| | | | | | | | |
|---|---|---|---|---|---|---|---|
| 2020 | | | | 8/22 | 21.6 | triamcinolone acetonide (30) | subsides<br>three times daily until your pain completely subsides |
| Bardellini 2020 | Italia | RCT | MiRAS | 12/18 | 8.9±2.2 | diode laser (30) | Laser therapy was administered on day 1 (T0) for three consecutive days |
| | | | | 11/19 | 8.4±2.1 | Sham treatment (30) | Laser therapy was administered on day 1 (T0) for three consecutive days |
| Nirmala 2019 | India | RCT | NA | NA | NA | Bacillus Clausii probiotic for local application (20) | twice daily for 1 week |
| | | | | | | triamicinolone paste for local application (20) | twice daily |
| Soliman 2019 | Egypt | RCT | MiRAS | 6/4 | 30.1±6.9 | diode laser treatment (10) | performed in the first visit only |
| | | | | 7/3 | 28.1±5.8 | placebo (10) | rinse a diluted sodium bicarbonate oral 4 times/day |
| Tavangar 2019 | Iran | RCT | minor RAS | 24/36 | 18-50 | triamcinolone muco-adhesive paste (20) | three times a day, until the ulcer healed completely |
| | | | | | | Placebo (20) | three times a day, until the ulcer healed completely |
| Halboub 2019 | Saudi Arabia | RCT | Minor:51 Major:7 | 16/22 | 30.13±16.62 | N-acetyl cysteine (NAC) (200 mg dissolved in water, n = 38) | mouthwashes for 30 seconds |
| | | | | 9/11 | 25.50±6.86 | 0.12% chlorhexidine digluconate (CHX, n = 20) | mouthwashes for 30 seconds |
| El-Wakeel 2019 | Egypt | RCT | minor aphthous ulcers | 19/21 | 28.9 | insulin-liposomal gel (40) | once daily for 6 days |
| | | | | 20/20 | | placebo gel (40) | once daily for 6 days |
| Rahmani 2018 | Iran | RCT | minor recurrent aphthous stomatitis | 8/12 | 35.15±9.8 | 0.5% Chitosan mouthwash (20) | keep 5 cc mouthwash for 4 minutes in the mouth and then spit it out 3 times a day after each meal |
| | | | | | | 0.5% Triamcinolone mouthwash (20) | keep 5 cc mouthwash for 4 minutes in the mouth and then spit it out 3 times a day after each meal |

| | | | | | | | |
|---|---|---|---|---|---|---|---|
| Sharma 2018 | India | RCT | minor aphthous ulcers | NA | 26.5 | 0.1%Triamcinolone acetonide (10) | 4 times a day, preferably following oral hygiene after breakfast, lunch, dinner and at bedtime |
| | | | | | 26.7 | 20% Benzocaine gel (10) | 4 times a day, preferably following oral hygiene after breakfast, lunch, dinner and at bedtime |
| | | | | | 28.7 | 100 mg Doxycycline hyclate (10) | The powdered doxycycline hyclate was mixed with denture adhesive and normal saline with 20:2:1 ratio and was directly placed over the ulcer using a plastic instrument |
| | | | | | 28.4 | placebo (10) | 4 times a day, preferably following oral hygiene after breakfast, lunch, dinner and at bedtime |
| Ofluoglu 2017 | Turkey | RCT | NA | 78/82 | 38.76±13.03 | TA pomade contains 0.1% triamcinolon acetonide (53) | rinse their mouth with tap water prior to the administration of the agent and apply the agent to the ulcer 4 times per day (after meals and before bed time) for 7 days |
| | | | | | | TGO gel contains 92.67% triester glycerol oxide (56) | rinse their mouth with tap water prior to the administration of the agent and apply the agent to the ulcer 4 times per day (after meals and before bed time) for 7 days |
| | | | | | | placebo gel (51) | rinse their mouth with tap water prior to the administration of the agent and apply the agent to the ulcer 4 times per day (after meals and before bed time) for 7 days |
| Zeini 2017 | Egypt | RCT | minor RAS | 8/6 | 34.6 | CO2 laser (14) | single session treatment |
| | | | | 4/6 | 30.2 | placebo (10) | sham laser |
| Rodríguez-Archilla | Spain | RCT | minor | 8/17 | 33±11 | cauterization with silver nitrate (25) | single treatment |

| | | | | | | | |
|---|---|---|---|---|---|---|---|
| 2017 | | | | 8/17 | 31±14 | propolis (25) | Spray 3 times a day and avoid application after eating or drinking |
| | | | | 11/14 | 30±11 | placebo (25) | Spray 3 times a day and avoid application after eating or drinking |
| Pandya | India | RCT | minor aphthous ulcers | 14/6 | 25.1±13.79 | a gel containing approximately 2% quercetin (20) | applied topically on the ulcers TID for total of 7 days and were restrained from eating, drinking, or rinsing the mouth for 30 minutes after each application |
| 2017 | | | | 12/8 | 27±13.29 | 0.15% benzydamine hydrochloride mouthwash (20) | rinsed for 30 seconds and then expelled out, approximately 15 minutes before meals TID for 7 days |
| Yilmaz | Turkey | RCT | minor RAS | 24/16 | 26±9.2 | Er,Cr:YSGG laser (40) | only at the first visit |
| 2017 | | | | | | placebo (40) | only at the first visit |
| Nasry | Egypt | RCT | NA | 23/37 | 28.5 | herbal combination of Acacia nilotica and Licorice (15) | apply the medication on the lesion q.i.d. after drying it with a small sterile cotton pad and refrain from eating at least for 30 min after the drug application |
| 2016 | | | | | | Amlexanox 5% (15) | apply the medication on the lesion q.i.d. after drying it with a small sterile cotton pad and refrain from eating at least for 30 min after the drug application |
| | | | | | | diode laser (15) | single treatment, 2nd or a 3rd laser pass was occasionally needed to decrease the pain |
| | | | | | | placebo adhesive tablet (15) | apply the medication on the lesion q.i.d. after drying it with a small sterile cotton pad and refrain from eating at least for 30 min after the |

| | | | | | | | |
|---|---|---|---|---|---|---|---|
| | | | | | | | drug application |
| Abbasi 2016 | Iran | RCT | minor aphthous ulcers | 18/22 | 36.2±3.36 | Adcortyl 0.1% (20) | four times daily for 7 days |
| | | | | | | 5% Amlexanox (20) | four times daily for 7 days |
| Andishe Tadbir 2015 | Iran | RCT | minor aphthous stomata | 5/10 | 28.93±7.29 | placebo (Orabase alone) (15) | four times a day, after eating and performing oral hygiene and to continue till complete resolution |
| | | | | 9/5 | 30.86±4.79 | triamcinolone in Orabase (14) | four times a day, after eating and performing oral hygiene and to continue till complete resolution |
| Raeesi 2015 | Iran | RCT | major ulcer were excluded | 2/18 | 26.8 | paste containing 5% of licorice extract (20) | Three times after meal and one time before sleeping for 5 days |
| | | | | 4/16 | 24.45 | paste without licorice extract (20) | Three times after meal and one time before sleeping for 5 days |
| Aggarwal 2014 | India | RCT | NA | 18/12 | NA | diode laser (30) | single treatment |
| | | | | | | Sham treatment (30) | single treatment |
| Mansour 2014 | Saudi Arabia | RCT | minor RAS | 38/52 | 31.7±8.4 | mucoadhesive gel with aloe vera as active ingredient (30) | apply the drug to the ulcer four times a day (after meals and before bedtime) for 5 days using finger or cotton tip applicator and to refrain from eating and drinking for 30 min after application |
| | | | | | | plain mucoadhesive gel (placebo) (30) | apply the drug to the ulcer four times a day (after meals and before bedtime) for 5 days using finger or cotton tip applicator and to refrain from eating and drinking for 30 min after application |
| Deshmukh 2014 | India | RCT | minor RAS | 31/29 | 32.51±11.80 | Curcumin gel (30) | apply the gel three times a day on each ulcer after meals and not to consume food or water for half an hour after application of the gel |
| | | | | | | Triamcinolone Acetonide gel (30) | apply the gel three times a day on each ulcer |

| | | | | | | | |
|---|---|---|---|---|---|---|---|
| | | | | | | | after meals and not to consume food or water for half an hour after application of the gel |
| El-Haddad | Kingdom of Saudi Arabia | RCT | minor RAS | 54/126 | <20y: 7<br>20-29y: 41<br>≥30y: 19 | topical commercial honey (67) | three times a day (after meals) for 8 days |
| 2014 | | | | | <20y: 6<br>20-29y: 21<br>≥30y: 30 | 0.1% triamcinolone acetonide (57) | three times a day (after meals) for 8 days |
| | | | | | <20y: 8<br>20-29y: 31<br>≥30y: 17 | Orabase (56) | three times a day (after meals) for 8 days |
| Albrektson | Sweden | RCT | minor RAS | 5/15 | 22.5±16.7 | low-level laser therapy (LLLT) (20) | 3 occasions, with a 1-day interval. |
| 2014 | | | | 11/9 | 27.5±25.0 | Placebo without any laser power (20) | 3 occasions, with a 1-day interval. |
| Soylu | Turkey | RCT | NA | 19/16 | 25.1 15-40 | Silver nitrate sticks (35) | r nitrate stick was gently held over the ulcer for a few seconds until the lesion turned to white, after the procedure, the oral cavity was rinsed with water for 5 min. |
| 2014 | | | | 30/17 | 23.5 16-35 | placebo sticks (30) | the empty sticks were held over the ulcer, after the procedure, the oral cavity was rinsed with water for 5 min. |
| Soylu and Okuyucu | Turkey | RCT | recurrent oral aphthous ulcers less than 1 cm | 17/18 | 38.31±5.40 | Sucralfate suspension (35) | 4 times a day 5 mL as an oral rinse for 1 to 2 minutes after routine mouth care and before sleep for one week |
| 2014 | | | | 19/16 | 38.97±5.14 | chlorhexidine oral rinse (35) | 4 times a day as an oral rinse for 1 to 2 minutes after routine mouth care and before sleep for one week |

| | | | | | | | |
|---|---|---|---|---|---|---|---|
| Bhat | India | RCT | less than 5 mm | 37/13 | 27 | 5% Lexanox oral paste (50) | 4 times a day, squeeze out approximately ¼ inch (0.5 cm) of the paste from the tube on a clean fingertip and apply to the site of the ulcer |
| 2013 | | | | 26/14 | | placebo paste (50) | 4 times a day, squeeze out approximately ¼ inch (0.5 cm) of the paste from the tube on a clean fingertip and apply to the site of the ulcer |
| Bhalang | Thailand | RCT | MiRAU | NA | NA | 0.1% triamcinolone acetonide (60) | 3 times/day for 7 days |
| 2013 | | | | | | 0.5% acemannan extracted from Aloe vera (60) | 3 times/day for 7 days |
| | | | | | | placebo (60) | 3 times/day for 7 days |
| Halim | Malaysia | RCT | size < 10 mm | NA | NA | turmeric (10) | twice a day for five consecutive days |
| 2013 | | | | | | triamcinolone (10) | twice a day for five consecutive days |
| Jiang and Yang | China | RCT | MiRAS | 11/24 | 33.03±3.28 | Diosmectite paste (35) | apply the paste to the appointed ulcer 4 times per day (after meals and before bedtime) for 5 days (day 1 to day 5) |
| 2013 | | | | 11/19 | 34.50±4.85 | placebo paste (30) | apply the paste to the appointed ulcer 4 times per day (after meals and before bedtime) for 5 days (day 1 to day 5) |
| Jiang and Zhu | China | RCT | MiRAS | 14/28 | 30.86±7.53 | gelatin containing berberine (42) | 4 times per day for 5 days |
| 2013 | | | | 10/32 | 31.19±6.00 | vehicle (42) | 4 times per day for 5 days |
| Prasad | India | RCT | miRAU | NA | 27.48±6.82 | CO2 laser (25) | single |
| 2013 | | | | | | placebo (25) | single |
| Vijayabala | India | RCT | minor RAS | 30/20 | 25.14 16-65 | powdered doxycycline hyclate tablet topically whereas (25) | A single application of the medicament or placebo was done during the patient's initial visit |
| 2013 | | | | | | powdered placebo tablet (25) | A single application of the medicament or placebo was done during the patient's initial visit |
| Sattayut | Thailand | RCT | minor RAS | NA | 21 19-23 | CO2 laser (7) | single, 5 seconds |

| | | | | | | | |
|---|---|---|---|---|---|---|---|
| 2013 | | | | | 23 18-39 | sham laser (7) | single, 5 seconds |
| Jiang | China | RCT | 10 mm- 2 mm | 36/62 | 37 | 5 mg allicin adhesive tablets (48) | apply 1 adhesive tablet to the appointed ulcer 4 times a day (after meals and before bedtime) for 5 days |
| 2012 | | | | | | vehicle only (48) | apply 1 adhesive tablet to the appointed ulcer 4 times a day (after meals and before bedtime) for 5 days |
| Manifar | Iran | RCT | not greater than 6 mm | 15/13 | 36.1±9.6 | 2% curcumin (28) | twice per day for a two week |
| 2012 | | | | 17/12 | 33.3 ±9.5 | placebo gel (29) | twice per day for a two week |
| Liu | China | RCT | <10 mm | 50/70 | 32.29±11.25 | dexamethasone ointment (120) | 3 times a day (after meals) for 5 days |
| 2012 | | | | 52/68 | 31.67±12.04 | placebo (120) | 3 times a day (after meals) for 5 days |
| Babaee | Iran | RCT | minor RAS | 10/10 | 27.95±7.96 | Aloe vera gel (20) | thraee-times a day for at least ten days |
| 2012 | | | | 8/12 | 29.25±7.48 | placebo (20) | thraee-times a day for at least ten days |
| Zand | Iran | RCT | minor aphthous ulcers | 1/9 | 35.6 22-56 | non-ablative CO2 laser therapy (NACLT) (10) | single |
| 2012 | | | | | | placebo (10) | single |
| Galal | Egypt | RCT | NA | NA | 18-35 | Licorice (10) | four times on the lesions |
| 2012 | | | | | | control (10) | four times on the lesions |
| Trinchieri | Italy | RCT | minor aphthous stomatitis | 7/8 | 8-36 | lozenge containing Lactobacillus brevis CD2 (15) | 4 times daily for a total period of 7 days |
| 2011 | | | | 8/7 | 7-35 | placebo (15) | 4 times daily for a total period of 7 days |
| Meng | China | RCT | Minor Recurrent aphthous ulceration | 44/64 | 29.82±10.44 | amlexanox adhesive pellicles (108) | 4 times a day (after meals and before bedtime) for 5 days |
| 2009 | | | | 37/71 | 30.18±11.06 | placebo (105) | 4 times a day (after meals and before bedtime) for 5 days |

| | | | | | | | |
|---|---|---|---|---|---|---|---|
| Tezel | Turkey | RCT | NA | 4/6 | 29.7±7.4 | (triamcinolone acetonide 0.1% | three times a day for 1 wk (10) |
| 2009 | | | | 3/7 | 34.3±7.7 | Nd:YAG laser | single (10) |
| Skulason | Iceland | RCT | minor recurrent oral ulceration | 7/18 | NA | Doxy-Gel, a gel containing a low dose of doxycycline (25) | four times a day |
| 2009 | | | | 10/14 | | placebo (24) | four times a day |
| Zand | Iran | RCT | miRAS | 2/13 | 37.9±10.9 | CO2 laser (15) | single |
| 2009 | | | | | | placebo (15) | single |
| Moghadamnia | Iran | RCT | minor RAS | 10/5 | 26.27±4.28 | bioadhesive with licorice (15) | q.i.d. for 5 days |
| 2009 | | | | | | bioadhesive without licorice (15) | q.i.d. for 5 days |
| Gorsky | Israel | RCT | RAS minor | 9/9 | 37 (16-66) | 0.2% aqueous solution of minocycline as a mouthwash (18) | four times a day, avoiding any food or drink for 1 hour after rinsing. Therapy continued up to 10 days following the onset of the lesion or until resolution of pain |
| 2008 | | | | 4/11 | 38 (17-71) | aqueous placebo mouthwash (15) | four times a day, avoiding any food or drink for 1 hour after rinsing. Therapy continued up to 10 days following the onset of the lesion or until resolution of pain |
| Rodríguez | Colombia | RCT | NA | 14/34 | 30.6±9.0 | 0.05% clobetasol propionate oral paste (48) | s four times per day for 5 days |
| 2007 | | | | 30/18 | 34.5±13.7 | 5% amlexanox oral paste (48) | s four times per day for 5 days |
| Arikan | Turkey | RCT | NA | 11/9 | 30.9 (18-47) | cryotherapy (20) | NA |
| 2006 | | | | | | hydrogel (20) | NA |
| Liu | China | RCT | minor aphthous ulcerations | 101/111 | 34 | amlexanox tablets (104) | 4 times a day (after meals and before bedtime) for 5 days |
| 2006 | | | | | | vehicle tablets (108) | 4 times a day (after meals and before bedtime) for 5 days |
| Garnick | USA | RCT | aphthous stomatitis | 35 (19-56) | NA | Carrier gel (4) | NA |

| | | | | | | | |
|---|---|---|---|---|---|---|---|
| | | | minor | | | | |
| 1998 | | | | | | Aloe vera (3) | NA |
| Khandwala 1 | USA | RCT | minor ulcers | NA | NA | Vehicle (512) | four times a day until ulcers healed or for the duration of the study |
| 1997 | | | | | | 5% Amlexanox (579) | four times a day until ulcers healed or for the duration of the study |
| Ylikontiola | Finland | RCT | minor aphthae | 4/27 | woman: 38 (23-63) man:32 (28-45) | doxymycine (15) | Medications were covered by isobutyl cyanoacrylate (Iso-Dent). Application was made only once during the recurrent aphthous ulcer episode |
| 1997 | | | | | | calcii gluconase as placebo (16) | Medications were covered by isobutyl cyanoacrylate (Iso-Dent). Application was made only once during the recurrent aphthous ulcer episode |
| Greer Jr | USA | RCT | NA | NA | 18-70 | 5% amlexanox paste (18) | twice daily for 3 days and once on day 4 |
| 1993 | | | | | | vehicle paste (14) | twice daily for 3 days and once on day 4 |
| Miles | USA | RCT | NA | NA | NA | 0.025% triamcinolone acetonide (6) | The patient returned to the clinic each time a new lesion appeared for reapplication of their assigned treatment |
| 1993 | | | | | | 0.12% chlorhexidine digluconate (6) | The patient returned to the clinic each time a new lesion appeared for reapplication of their assigned treatment |
| Taylor | UK | RCT | minor aphthous ulceration | 7/11 | 32.6 (20-57) | PGE2 gel (18) | twice daily for 10 days or untel healing |
| 1993 | | | | 6/9 | | placebo (15) | twice daily for 10 days or untel healing |
| Hunter | UK | RCT | minor aphthous | 12/29 | 27.4 (17-61) | 0.2% chlorhexidine gluconate (38) | 3 times daily for 6 weeks |

| | | | | | | | |
|---|---|---|---|---|---|---|---|
| 1987 | | | ulceration | | | placebo mouthwash (38) | 3 times daily for 6 weeks |
| Matthews 1987 | UK | RCT | minor RAS | NA | NA | Benzydamine hydrochloride 0.15% mouthwash (18) | 2 minutes every 4 hours for 3 mouths |
| | | | | | | 0.2% aqueous chlorhexidine gluconate mouthwash (18) | 2 minutes every 4 hours for 3 mouths |
| | | | | | | Placebo (18) | 2 minutes every 4 hours for 3 mouths |
| Yeoman | UK | RCT | minor aphthous ulcers | 9/12 | 35 (17-63) | betamethasone valerate aerosol (10) | NA |
| 1978 | | | | | | placebo aerosol (10) | NA |
| Addy | UK | RCT | NA | NA | NA | Chlorhexidine gel (20) | 3 times a day after meals for 35 days |
| 1976 | | | | | | Placebo gel (20) | 3 times a day after meals for 35 days |
| Addy | UK | RCT | NA | 11/15 | 17-60 | Asringent (Zine Sulphate and Zine Chloride 0.5ml) (12) | 3 times daily after meals for 5 weeks |
| 1974 | | | | | | Control (12) | 3 times daily after meals for 5 weeks |
| Browne | UK | RCT | severe recurrent aphthous stomatitis | 9/17 | 29.6 (10-53) | Orabase (26) | three times a day |
| 1968 | | | | | | 0.1% triamcinolone acetonide in orabase (26) | three times a day |

## Table S7 Outcomes of included studies

| Author Year | Clinical response assessment<br>size/healing time | Symptom assessment<br>VAS/NRS/0-10 | Adverse effect | Hematologic values | Relapse |
|---|---|---|---|---|---|
| Huo<br>2021 | healing time | VAS | NA | NA | NA |
| Aggour (audlt)<br>2021 | Size | VAS | NA | NA | Outbreak frequency/6 months<br>Probiotics: 3.33 (0.64)<br>Placebo: 3.65 (0.32) |
| Aggour (child)<br>2021 | Size | VAS | NA | NA | Outbreak frequency/6 months<br>Probiotics: 2.65 (0.54)<br>Placebo: 3.65 (0.62) |
| Shi<br>2020 | healing time | NA | NA | NA | NA |
| Kavita<br>2020 | Size | VAS | NA | NA | NA |
| Shao<br>2020 | Size | VAS | No adverse effects were observed during the study | NA | NA |
| Pedersen<br>2020 | NA | VAS | No side effects or adverse events were reported by the patients during the intervention period | NA | NA |
| Ghorbani<br>2020 | Size | VAS | No adverse effect was observed in the two groups during the study | NA | NA |
| Ibrahim<br>2020 | Size and healing time | NA | No side effects were reported in both groups | NA | NA |
| Kia<br>2020 | Size | VAS | No burning sensation, the abnormal change in the mucosa, or taste sense malfunction was observed | NA | NA |

| | | | | | |
|---|---|---|---|---|---|
| Owlia 2020 | Size and healing time | NA | three patients just complained of minor burning sensation after application of drug | NA | NA |
| Seyyedi 2020 | Size | NA | NA | NA | NA |
| Raman 2020 | Size and healing time | NA | There were no major adverse effects reported in both the groups during the study period | NA | NA |
| Bardellini 2020 | Size | VAS | NA | NA | NA |
| Nirmala 2019 | Size | NA | NA | NA | NA |
| Soliman 2019 | Size | VAS | NA | NA | NA |
| Tavangar 2019 | Size and healing time | VAS | NA | | NA |
| Halboub 2019 | Size and healing time | VAS | NA | NA | NA |
| El-Wakeel 2019 | healing time | VAS | NA | NA | NA |
| Rahmani 2018 | Size and healing time | VAS | one patient (5%) felt mild throat sore after applying Chitosan mouthwash and bad taste 20 minutes | NA | NA |
| Sharma 2018 | Size | VAS | None of the patients reported any allergy to any therapy | NA | NA |
| Ofluoglu 2017 | Size | VAS | All patients tolerated the agents and no side effect were reported during the study | NA | NA |
| Zeini 2017 | healing time | VAS | NA | NA | NA |

| | | | | | |
|---|---|---|---|---|---|
| Rodríguez-Archilla 2017 | healing time | NA | No patient reported adverse effects related to the treatment received | NA | NA |
| Pandya 2017 | Size | VAS | NA | NA | NA |
| Yilmaz 2017 | NA | VAS | No complications or adverse reactions were observed | NA | NA |
| Nasry 2016 | Size | VAS | NA | NA | NA |
| Abbasi 2016 | Size | VAS | NA | NA | NA |
| Andishe Tadbir 2015 | Size and healing time | VAS | NA | NA | NA |
| Raeesi 2015 | Size and healing time | VAS | without side effect | NA | NA |
| Aggarwal 2014 | healing time | NA | NA | NA | NA |
| Mansour 2014 | Size | VAS | No adverse effects were reported regarding all used mucoadhesive gels | NA | NA |
| Deshmukh 2014 | Size and healing time | VAS | No patient suffered from any allergic reaction to the gels used in the study for treatment of minor RAS | NA | NA |
| El-Haddad 2014 | Size and healing time | VAS | No systemic side effects | NA | NA |
| Albrektson 2014 | NA | VAS | Twenty patients in each group completed the study, with no reported adverse events | NA | NA |
| Soylu 2014 | healing time | VAS | No side effects were recorded in either of the groups. | NA | NA |

| | | | | | |
|---|---|---|---|---|---|
| Soylu and Okuyucu 2014 | healing time | VAS | No side effects were recorded in either of the groups | NA | NA |
| Bhat 2013 | Size | VAS | 8 patients belonging to the amlexanox group who complained of adverse events during the study. 3 patients complained of a transient "stinging" at the application site, which was considered to be mild in severity and probably related to the studied medications. 2 patients complained of a metallic taste in the oral cavity soon after application of the paste. 3 other patients complained of a "cooling" sensation at the application site following which they felt slight discomfort for a few minutes. None of these 8 cases discontinued the trial due to the adverse events | NA | NA |
| Bhalang 2013 | Size | VAS | No subjects exhibited allergic reactions or side-effects to acemannan | There were no significant differences between the blood test values before and after 7 days of acemannan application | NA |
| Halim 2013 | Size | VAS | NA | NA | NA |
| Jiang and Yang 2013 | Size | VAS | no obvious adverse side effects | NA | NA |
| Jiang and Zhu 2013 | Size | VAS | without obvious side effects | NA | NA |
| Prasad 2013 | healing time | 0-10 score | NA | NA | NA |
| Vijayabala 2013 | healing time | VAS | 8 report transient bitter sensation | NA | NA |
| Sattayut | Size | VAS | NA | NA | NA |

| | | | | | |
|---|---|---|---|---|---|
| 2013 | | | | | |
| Jiang 2012 | Size | VAS | No minor or major adverse side effects | None of the hematologic values at day 6 were considered clinically abnormal | NA |
| Manifar 2012 | Size | NA | No adverse drug reaction to a treatment was reported | NA | NA |
| Liu 2012 | Size and healing time | NRS | Twelve cases (4 in the treatment group and 8 in the control group) reported slight adverse reactions | the concentration of dexamethasone in the serum after using the dexamethasone ointment was <0.502 ng/mL | NA |
| Babaee 2012 | Size | VAS | NA | NA | NA |
| Zand 2012 | healing time | NA | The patients reported no warmth in their lesions during laser treatment | NA | NA |
| Galal 2012 | Size | VAS | NA | NA | NA |
| Trinchieri 2011 | Size | 0-10 score | complete absence of side effects | NA | NA |
| Meng 2009 | Size | VAS | None of the patients in the study was observed or reported to have any adverse reactions | None of the hematologic values were considered clinically abnormal | NA |
| Tezel 2009 | NA | VAS | there were no reported adverse events | NA | NA |
| Skulason 2009 | NA | 0-10 score | One subject reported a short burning sensation from the ulcer after application of the gel | NA | NA |
| Zand 2009 | NA | VAS | None of the patients reported pain or warmth in their lesions during laser therapy | NA | NA |

| | | | | | |
|---|---|---|---|---|---|
| Moghadamnia 2009 | Size and healing time | VAS | NA | NA | NA |
| Gorsky 2008 | NA | VAS | no tooth discoloration or other side effects were reported | NA | NA |
| Rodríguez 2007 | NA | VAS | There were no adverse effects reported in any of the treatment groups by the patients or the examiners | NA | NA |
| Arikan 2006 | Size | NA | NA | NA | NA |
| Liu 2006 | Size | VAS | There were 2 adverse events reported in the study, One was in the amlexanox group presented as local,transient "stinging", Another happened in the vehicle-control group as edema of the fingers. | NA | NA |
| Garnick 1998 | healing time | VAS | NA | NA | NA |
| Khandwala 1997 | Size and healing time | VAS | NA | NA | NA |
| Ylikontiola 1997 | NA | VAS | NA | NA | NA |
| Greer Jr 1993 | Size | NA | None of the patients in the study was observed to have or reported any adverse experience | NA | NA |
| Miles 1993 | healing time | VAS | NA | NA | NA |
| Taylor 1993 | healing time | VAS | NA | NA | NA |
| Hunter 1987 | healing time | NA | NA | NA | Total ulcer numbers (6 weeks): Chlorhexidine: 7.54±6.52 Placebo: 8.32±5.52 |

| | | | | | |
|---|---|---|---|---|---|
| | | | | | Interval between ulcers (6 weeks):<br>Chlorhexidine: 7.26±8.61<br>Placebo:3.86±2.05 |
| Matthews<br>1987 | NA | NA | NA | NA | No. of new ulcers (3 months)<br>Benzydamine: 7 (2-33)<br>Chlorhexidine: 6.5 (3-20)<br>Placebo: 8 (2-20) |
| Yeoman<br>1978 | healing time | NA | NA | NA | NA |
| Addy<br>1976 | healing time | NA | NA | NA | NA |
| Addy<br>1974 | healing time | NA | NA | NA | NA |
| Browne<br>1968 | healing time | NA | NA | NA | No. of new ulcers (8 months)<br>Placebo: 7.81<br>Triamcinolone acetonide in orabase : 7.00<br>Triamcinolone acetonide in watery base : 6.42 |

# Table S8 Details information of studies for each outcome in network meta-analysis (1)

| Author Year | Treatment | Size-reducing effect ( judged by Effectiveness index, EI=ulcer reduction area (mm2) / ulcer baseline area (mm2) ) | | | | | | | | | | | | | | | | | | |
|---|---|---|---|---|---|---|---|---|---|---|---|---|---|---|---|---|---|---|---|---|
| | | Total | Mean (%) | SD | days | Mean (%) | SD | days | Mean (%) | SD | days | Mean (%) | SD | days | Mean (%) | SD | days | Mean (%) | SD | days |
| Huo | Laser | NA | | | | | | | | | | | | | | | | | | |
| 2021 | Triamcinolone | NA | | | | | | | | | | | | | | | | | | |
| Aggour (audlt) | Probiotics | 30 | 44.55 | 20.79 | 3 | 70.03 | 15.17 | 5 | | | | | | | | | | | | |
| 2021 | Placebo | 30 | 31.66 | 24.86 | 3 | 62.76 | 18.24 | 5 | | | | | | | | | | | | |
| Aggour (child) | Probiotics | 30 | 51.64 | 18.66 | 3 | 76.85 | 14.13 | 5 | | | | | | | | | | | | |
| 2021 | Placebo | 30 | 37.98 | 27.13 | 3 | 62.45 | 22.68 | 5 | | | | | | | | | | | | |
| Shi | Aloe | NA | | | | | | | | | | | | | | | | | | |
| 2020 | Chitosan | NA | | | | | | | | | | | | | | | | | | |
| Kavita | Triamcinolone | 30 | 33.70 | 3.31 | 3 | 90.83 | 2.07 | 5 | | | | | | | | | | | | |
| 2020 | Amlexanox | 30 | 22.95 | 8.03 | 3 | 82.80 | 6.89 | 5 | | | | | | | | | | | | |
| Shao | Chitosan | 34 | 1.12 | 85.85 | 2 | 20.07 | 85.85 | 4 | 56.26 | 80.35 | 6 | | | | | | | | | |
| 2020 | Placebo | 32 | -11.35 | 131.35 | 2 | 1.20 | 150.42 | 4 | 23.11 | 152.56 | 6 | | | | | | | | | |
| Pedersen | Probiotics | NA | | | | | | | | | | | | | | | | | | |
| 2020 | Placebo | NA | | | | | | | | | | | | | | | | | | |
| Ghorbani | Zinc | 23 | 78.23 | 28.31 | 3 | 98.51 | 31.60 | 5 | 100.00 | 0.00 | 7 | | | | | | | | | |
| 2020 | Placebo | 23 | -7.03 | 40.95 | 3 | 55.65 | 37.23 | 5 | 93.96 | 39.44 | 7 | | | | | | | | | |
| Ibrahim | Triamcinolone | 20 | 89.54 | 55.10 | 7 | 98.93 | 58.48 | 14 | | | | | | | | | | | | |
| 2020 | Probiotics | 20 | 97.95 | 52.23 | 7 | 100.00 | 0.00 | 14 | | | | | | | | | | | | |
| Kia | Curcumin | 29 | 16.44 | 22.50 | 1 | 50.49 | 20.96 | 4 | 74.90 | 20.17 | 7 | 90.03 | 19.89 | 10 | | | | | | |
| 2020 | Triamcinolone | 29 | 17.78 | 17.59 | 1 | 52.77 | 16.34 | 4 | 75.61 | 15.46 | 7 | 91.68 | 15.12 | 10 | | | | | | |
| Owlia | Penicillin | 25 | 59.58 | NA | 3 | 91.23 | NA | 6 | 100.00 | NA | 8 | | | | | | | | | |
| 2020 | Placebo | 25 | -31.18 | NA | 3 | -4.79 | NA | 6 | -8.28 | NA | 8 | | | | | | | | | |

| | | | | | | | | | | | | | | | | | | | | |
|---|---|---|---|---|---|---|---|---|---|---|---|---|---|---|---|---|---|---|---|---|
| Seyyedi | Laser | 5 | 7.99 | 40.44 | 3 | 66.30 | 40.73 | 5 | 92.16 | 41.76 | 7 | | | | | | | | | |
| 2020 | Laser | 5 | 9.97 | 53.60 | 3 | 87.10 | 46.40 | 5 | 97.65 | 50.67 | 7 | | | | | | | | | |
| | Triamcinolone | 5 | 37.65 | 29.28 | 3 | 95.59 | 20.19 | 5 | 100.00 | 0.00 | 7 | | | | | | | | | |
| Raman | Curcumin | 30 | -72.75 | 61.24 | 2 | -190.45 | 99.72 | 3 | -254.49 | 150.84 | 4 | -151.97 | 140.45 | 5 | -62.08 | 125.00 | 6 | 71.91 | 36.80 | 7 |
| 2020 | Triamcinolone | 30 | -67.84 | 44.59 | 2 | -175.08 | 60.66 | 3 | -144.26 | 44.59 | 4 | -57.05 | 82 | 5 | 16.72 | 44 | 6 | 28.19 | 59 | 7 |
| Bardellini | Laser | 30 | 68.61 | 20.05 | 4 | 87.62 | 21.12 | 7 | | | | | | | | | | | | |
| 2020 | Placebo | 30 | 42.92 | 24.72 | 4 | 74.52 | 26.15 | 7 | | | | | | | | | | | | |
| Nirmala | Probiotics | 20 | 58.15 | 44.05 | 5 | 83.70 | 49.78 | 10 | | | | | | | | | | | | |
| 2019 | Triamcinolone | 20 | 41.41 | 42.73 | 5 | 95.59 | 44.05 | 10 | | | | | | | | | | | | |
| Soliman | Laser | 10 | 62.80 | 41.92 | 4 | 97.41 | 44.97 | 6 | | | | | | | | | | | | |
| 2019 | Placebo | 10 | 4.92 | 50.82 | 4 | 52.46 | 45.25 | 6 | | | | | | | | | | | | |
| Tavangar | Triamcinolone | 20 | 37.50 | NA | 3 | 82.29 | NA | 5 | | | | | | | | | | | | |
| 2019 | Placebo | 20 | 11.11 | NA | 3 | 55.56 | NA | 5 | | | | | | | | | | | | |
| Halboub | N-acetylcysteine | 38 | 3.08 | 5.57 | 2 | 25.20 | 22.12 | 4 | 59.52 | 49.6. | 6 | | | | | | | | | |
| 2019 | Chlorhexidine | 20 | 1.14 | 2.79 | 2 | 20.44 | 17.60 | 4 | 47.21 | 33.71 | 6 | | | | | | | | | |
| El-Wakeel | Insulin-liposomal gel | NA | | | | | | | | | | | | | | | | | | |
| 2019 | Placebo | NA | | | | | | | | | | | | | | | | | | |
| Rahmani | Chitosan | 20 | -77.49 | NA | 3 | 24.22 | NA | 5 | 76.92 | NA | 7 | 100.00 | NA | 10 | | | | | | |
| 2018 | Triamcinolone | 20 | -19.13 | NA | 3 | 33.33 | NA | 5 | 89.28 | NA | 7 | 100.00 | NA | 10 | | | | | | |
| Sharma | Triamcinolone | 10 | 82.76 | 30.39 | 4 | 100.00 | 0.20 | 8 | 100.00 | 0.00 | 10 | | | | | | | | | |
| 2018 | Benzydamine | 10 | 51.60 | 28.90 | 4 | 98.80 | 30.52 | 8 | 99.58 | 31.13 | 10 | | | | | | | | | |
| | Doxycycline | 10 | 73.79 | 23.79 | 4 | 99.86 | 27.13 | 8 | 100.00 | 0.00 | 10 | | | | | | | | | |
| | Placebo | 10 | -21.52 | 35.26 | 4 | 65.24 | 31.41 | 8 | 78.73 | 32.50 | 10 | | | | | | | | | |
| Ofluoglu | Triamcinolone | 53 | 14.51 | 5.06 | 2 | 37.78 | 8.09 | 4 | 70.11 | 9.46 | 6 | | | | | | | | | |
| 2017 | Triester Glycerol Oxide | 56 | 16.71 | 4.19 | 2 | 46.39 | 8.28 | 4 | 77.80 | 8.08 | 6 | | | | | | | | | |

| | | | | | | | | | | | |
|---|---|---|---|---|---|---|---|---|---|---|---|
| | Placebo | 51 | 12.61 | 5.86 | 2 | 34.88 | 8.64 | 4 | 65.94 | 8.88 | 6 |
| Zeini | Laser | NA | | | | | | | | | |
| 2017 | Placebo | NA | | | | | | | | | |
| Rodríguez-Archilla | Silver nitrate | NA | | | | | | | | | |
| 2017 | Honey | NA | | | | | | | | | |
| | Placebo | NA | | | | | | | | | |
| Pandya | Quercetin | 20 | 44.25 | 20.96 | 2 | 75.40 | 20.64 | 4 | 95.40 | 21.17 | 7 |
| 2017 | Benzydamine | 20 | 22.22 | 27.04 | 2 | 53.54 | 25.89 | 4 | 80.13 | 25.69 | 7 |
| Yilmaz | Laser | NA | | | | | | | | | |
| 2017 | Placebo | NA | | | | | | | | | |
| Nasry | Glycyrrhiza | 15 | 42.00 | 11.50 | 2 | 63.00 | 20.50 | 5 | | | |
| 2016 | Amlexanox | 15 | 48.10 | 16.50 | 2 | 77.80 | 28.70 | 5 | | | |
| | Laser | 15 | 52.70 | 19.80 | 2 | 85.10 | 22.00 | 5 | | | |
| | Placebo | 15 | 7.10 | 5.30 | 2 | 10.00 | 7.30 | 5 | | | |
| Abbasi | Dexamethasone | 20 | 53.46 | 1.01 | 3 | 80.77 | 22.69 | 5 | 100.00 | 0.00 | 7 |
| 2016 | Amlexanox | 20 | 48.31 | 44.92 | 3 | 71.69 | 39.08 | 5 | 92.31 | 43.69 | 7 |
| Andishe Tadbir | Placebo | 15 | -23.6 | 32.34 | 3 | 61.71 | 31.64 | 6 | | | |
| 2015 | Triamcinolone | 14 | 78.46 | 38.14 | 3 | 99.41 | 40.51 | 6 | | | |
| Raeesi | Glycyrrhiza | 20 | 36.65 | 33.65 | 1 | 96.14 | 37.11 | 3 | 99.95 | 38.26 | 5 |
| 2015 | Placebo | 20 | 6.87 | 26.74 | 1 | 70.10 | 24.90 | 3 | 91.73 | 26.45 | 5 |
| Aggarwal | Laser | NA | | | | | | | | | |
| 2014 | Placebo | NA | | | | | | | | | |
| Mansour | Aloe | 30 | 35.17 | NA | 4 | 59.48 | 24.64 | 6 | | | |
| 2014 | Placebo | 30 | 16.80 | NA | 4 | 31.48 | 31.48 | 6 | | | |
| Deshmukh | Curcumin | 30 | 93.03 | 25.49 | 3 | 99.96 | 28.40 | 5 | 100.00 | 0.00 | 7 |
| 2014 | Triamcinolone | 30 | 91.16 | 37.67 | 3 | 99.90 | 41.12 | 5 | 100.00 | 0.00 | 7 |

| | | | | | | | | | | | | | | | | | | | | |
|---|---|---|---|---|---|---|---|---|---|---|---|---|---|---|---|---|---|---|---|---|
| El-Haddad | Honey | 67 | 85.38 | NA | 1 | 98.95 | NA | 2 | 99.98 | NA | 3 | 100.00 | NA | 4 | 100.00 | NA | 5 | 100.00 | NA | 6 |
| 2014 | Triamcinolone | 57 | 24.36 | NA | 1 | 51.75 | NA | 2 | 72.33 | NA | 3 | 90.67 | NA | 4 | 97.24 | NA | 5 | 99.72 | NA | 6 |
| | Placebo | 56 | 23.42 | NA | 1 | 47.99 | NA | 2 | 64.35 | NA | 3 | 78.56 | NA | 4 | 89.50 | NA | 5 | 96.23 | NA | 6 |
| | | | 100.00 | NA | 7 | 100.00 | NA | 8 | | | | | | | | | | | | |
| | | | 99.97 | NA | 7 | 100.00 | NA | 8 | | | | | | | | | | | | |
| | | | 99.12 | NA | 7 | 99.95 | NA | 8 | | | | | | | | | | | | |
| Albrektson | Laser | NA | | | | | | | | | | | | | | | | | | |
| 2014 | Placebo | NA | | | | | | | | | | | | | | | | | | |
| Soylu | Silver nitrate | NA | | | | | | | | | | | | | | | | | | |
| 2014 | Placebo | NA | | | | | | | | | | | | | | | | | | |
| Soylu and | Sucralfate | NA | | | | | | | | | | | | | | | | | | |
| Okuyucu | | | | | | | | | | | | | | | | | | | | |
| 2014 | Chlorhexidine | NA | | | | | | | | | | | | | | | | | | |
| Bhat | Amlexanox | 50 | 47.01 | 49.91 | 4 | 93.10 | 53.54 | 6 | | | | | | | | | | | | |
| 2013 | Placebo | 50 | -27.92 | 37.67 | 4 | 12.05 | 34.99 | 6 | | | | | | | | | | | | |
| Bhalang | Triamcinolone | 60 | 10.00 | 10.00 | 2 | 45.00 | 10.34 | 5 | 70.00 | 10.00 | 7 | | | | | | | | | |
| 2013 | Aloe | 60 | 10.00 | 9.43 | 2 | 30.00 | 8.85 | 5 | 60.00 | 9.08 | 7 | | | | | | | | | |
| | Placebo | 60 | 5.00 | 10.00 | 2 | 10.00 | 10.00 | 5 | 30.00 | 10.00 | 7 | | | | | | | | | |
| Halim | Curcumin | 10 | 48.31 | 76.03 | 5 | | | | | | | | | | | | | | | |
| 2013 | Triamcinolone | 10 | 69.72 | 84.51 | 5 | | | | | | | | | | | | | | | |
| Jiang and Yang | Diosmectite | 35 | 1.65 | 20.82 | 2 | 28.81 | 21.40 | 4 | 70.37 | 18.60 | 6 | | | | | | | | | |
| 2013 | Placebo | 30 | 4.78 | 18.13 | 2 | 27.09 | 18.69 | 4 | 54.98 | 14.22 | 6 | | | | | | | | | |
| Jiang and Zhu | Berberine gelatin | 42 | 35.63 | 18.28 | 2 | 54.07 | 11.98 | 4 | 70.56 | 11.84 | 6 | | | | | | | | | |
| 2013 | Placebo | 42 | 14.14 | 20.74 | 2 | 37.66 | 18.33 | 4 | 54.35 | 16.40 | 6 | | | | | | | | | |
| Prasad | Laser | NA | | | | | | | | | | | | | | | | | | |
| 2013 | Placebo | NA | | | | | | | | | | | | | | | | | | |

| | | | | | | | | | | | | | | | | | |
|---|---|---|---|---|---|---|---|---|---|---|---|---|---|---|---|---|---|
| Vijayabala | Doxycycline | NA | | | | | | | | | | | | | | | |
| 2013 | Placebo | NA | | | | | | | | | | | | | | | |
| Sattayut | Laser | 7 | -18.75 | 216.25 | 1 | -31.25 | 198.75 | 2 | -75.00 | 124.75 | 3 | -12.5 | 86 | 5 | 50.00 | 72 | 7 |
| 2013 | Placebo | 7 | -31.58 | 47.79 | 1 | -26.32 | 48.63 | 2 | 5.26 | 97.89 | 3 | 47.37 | 78 | 5 | 78.95 | 55 | 7 |
| Jiang | Allicin | 48 | 28.20 | 32.68 | 2 | 57.30 | 34.30 | 4 | 72.50 | 38.72 | 6 | | | | | | |
| 2012 | Placebo | 48 | 9.80 | 31.66 | 2 | 36.50 | 39.66 | 4 | 55.10 | 35.32 | 6 | | | | | | |
| Manifar | Curcumin | 28 | 68.58 | 14.99 | 4 | 97.47 | 16.31 | 7 | | | | | | | | | |
| 2012 | Placebo | 29 | 59.04 | 14.06 | 4 | 95.52 | 13.91 | 7 | | | | | | | | | |
| Liu | Dexamethasone | 114 | 88.70 | 78.47 | 6±2 | | | | | | | | | | | | |
| 2012 | Placebo | 117 | 56.64 | 92.06 | 6±2 | | | | | | | | | | | | |
| Babaee | Aloe | 20 | 22.91 | 24.50 | 3 | 73.90 | 22.51 | 7 | 100.00 | 0.00 | 10 | | | | | | |
| 2012 | Placebo | 20 | 37.75 | 28.49 | 3 | 94.92 | 28.49 | 7 | 100.00 | 0.00 | 10 | | | | | | |
| Zand | Laser | NA | | | | | | | | | | | | | | | |
| 2012 | Placebo | NA | | | | | | | | | | | | | | | |
| Galal | Glycyrrhiza | 10 | 66.25 | 22.31 | 2 | 87.65 | 23.08 | 5 | | | | | | | | | |
| 2012 | Placebo | 10 | 13.78 | 16.56 | 2 | 19.00 | 16.79 | 5 | | | | | | | | | |
| Trinchieri | Probiotics | 15 | 99.93 | NA | 7 | | | | | | | | | | | | |
| 2011 | Placebo | 15 | 80.11 | NA | 7 | | | | | | | | | | | | |
| Meng | Amlexanox | 108 | 42.08 | 82.22 | 4 | 60.21 | 83.63 | 6 | | | | | | | | | |
| 2009 | Placebo | 105 | 19.67 | 78.39 | 4 | 41.28 | 91.51 | 6 | | | | | | | | | |
| Tezel | Triamcinolone | NA | | | | | | | | | | | | | | | |
| 2009 | Laser | NA | | | | | | | | | | | | | | | |
| Skulason | Doxycycline | NA | | | | | | | | | | | | | | | |
| 2009 | Placebo | NA | | | | | | | | | | | | | | | |
| Zand | Laser | NA | | | | | | | | | | | | | | | |
| 2009 | Placebo | NA | | | | | | | | | | | | | | | |

| | | | | | | | | | | | |
|---|---|---|---|---|---|---|---|---|---|---|---|
| Moghadamnia | Glycyrrhiza | 15 | 18.41 | 30.32 | 3 | 42.24 | 35.38 | 5 | | | |
| 2009 | Placebo | 15 | 18.02 | 33.05 | 3 | 33.91 | 41.63 | 5 | | | |
| Gorsky | Minocycline | NA | | | | | | | | | |
| 2008 | Placebo | NA | | | | | | | | | |
| Rodríguez | Clobetasol | NA | | | | | | | | | |
| 2007 | Amlexanox | NA | | | | | | | | | |
| Arikan | Cryotherapy | 10 | 43.75 | 38.40 | 3 | 89.44 | 39.66 | 7 | 100.00 | 0.00 | 10 |
| 2006 | Placebo | 10 | 20.99 | 42.19 | 3 | 80.25 | 39.74 | 7 | 98.77 | 43.26 | 10 |
| Liu | Amlexanox | 104 | 39.37 | 76.95 | 4 | 67.37 | 76.65 | 6 | | | |
| 2006 | Placebo | 108 | 6.44 | 97.34 | 4 | 37.68 | 92.02 | 6 | | | |
| Garnick | Placebo | NA | | | | | | | | | |
| 1998 | Aloe | NA | | | | | | | | | |
| Khandwala | Placebo | 512 | -0.03 | 0.06 | 3 | 0.84 | 0.50 | 4 | 4.54 | 1.14 | 5 |
| 1997 | Amlexanox | 579 | 2.98 | 0.67 | 3 | 10.35 | 2.10 | 4 | 26.66 | 2.56 | 5 |
| Ylikontiola | Doxycycline | NA | | | | | | | | | |
| 1997 | Placebo | NA | | | | | | | | | |
| Greer Jr | Amlexanox | 18 | 4.61 | NA | 2 | 15.70 | NA | 3 | 31.06 | NA | 4 |
| 1993 | Placebo | 14 | -10.91 | NA | 2 | -17.11 | NA | 3 | 8.56 | NA | 4 |
| Miles | Triamcinolone | NA | | | | | | | | | |
| 1993 | Chlorhexidine | NA | | | | | | | | | |
| Taylor | Prostaglandin E2 | NA | | | | | | | | | |
| 1993 | Placebo | NA | | | | | | | | | |
| Hunter | Chlorhexidine | NA | | | | | | | | | |
| 1987 | Placebo | NA | | | | | | | | | |
| Matthews | Benzydamine | NA | | | | | | | | | |
| 1987 | Chlorhexidine | NA | | | | | | | | | |

| | | |
|---|---|---|
| Yeoman | Betamethasone | NA |
| 1978 | Placebo | NA |
| Addy | Chlorhexidine | NA |
| 1976 | Placebo | NA |
| Addy | Zinc | NA |
| 1974 | Placebo | NA |
| Browne | Placebo | NA |
| 1968 | Triamcinolone | NA |

## Table S9 Details information of studies for each outcome in network meta-analysis (2)

| Author Year | Treatment | Symptom-reducing effect ( judged by Effectiveness index, EI=reduction in pain score / baseline ulcer pain score ) | | | | | | | | | | | | | | | | | | |
|---|---|---|---|---|---|---|---|---|---|---|---|---|---|---|---|---|---|---|---|---|
| | | Total | Mean (%) | SD | days | Mean (%) | SD | days | Mean (%) | SD | days | Mean (%) | SD | days | Mean (%) | SD | days | Mean (%) | SD | days |
| Huo | Laser | 25 | 60.16 | 10.94 | 1 | 88.28 | 10.74 | 3 | 100.00 | 0.00 | 7 | | | | | | | | | |
| 2021 | Triamcinolone | 26 | 14.76 | 7.56 | 1 | 47.28 | 7.91 | 3 | 94.55 | 7.03 | 7 | | | | | | | | | |
| Aggour (audlt) | Probiotics | 30 | 50.30 | 18.85 | 3 | 55.77 | 26.13 | 5 | | | | | | | | | | | | |
| 2021 | Placebo | 30 | 40.67 | 25.82 | 3 | 44.51 | 26.03 | 5 | | | | | | | | | | | | |
| Aggour (child) | Probiotics | 30 | 37.90 | 20.97 | 3 | 55.26 | 25.62 | 5 | | | | | | | | | | | | |
| 2021 | Placebo | 30 | 32.15 | 23.68 | 3 | 51.73 | 22.56 | 5 | | | | | | | | | | | | |
| Shi | Aloe | NA | | | | | | | | | | | | | | | | | | |
| 2020 | Chitosan | NA | | | | | | | | | | | | | | | | | | |
| Kavita | Triamcinolone | 30 | 44.64 | 3.04 | 3 | 80.36 | 1.79 | 5 | | | | | | | | | | | | |
| 2020 | Amlexanox | 30 | 7.14 | 8.57 | 3 | 38.10 | 6.19 | 5 | | | | | | | | | | | | |
| Shao | Chitosan | 34 | 12.95 | 50.52 | 2 | 47.67 | 51.81 | 4 | 80.31 | 45.85 | 6 | | | | | | | | | |
| 2020 | Placebo | 32 | 1.03 | 50.90 | 2 | 37.73 | 56.33 | 4 | 71.32 | 51.42 | 6 | | | | | | | | | |
| Pedersen | Probiotics | 10 | 56.30 | 73.90 | 90 | | | | | | | | | | | | | | | |
| 2020 | Placebo | 10 | 65.00 | 99.10 | 90 | | | | | | | | | | | | | | | |
| Ghorbani | Zinc | 23 | 38.98 | 26.89 | 2 | 77.30 | 25.79 | 3 | 93.09 | 24.57 | 4 | 100.00 | 25.85 | 5 | 100.00 | 0.00 | 6 | 100.00 | 0.00 | 7 |
| 2020 | Placebo | 23 | 3.87 | 23.78 | 2 | 16.19 | 25.25 | 3 | 28.65 | 25.25 | 4 | 47.28 | 26.55 | 5 | 65.76 | 24.69 | 6 | 86.53 | 21.54 | 7 |
| Ibrahim | Triamcinolone | NA | | | | | | | | | | | | | | | | | | |
| 2020 | Probiotics | NA | | | | | | | | | | | | | | | | | | |
| Kia | Curcumin | 29 | 5.45 | 22.73 | 1 | 38.18 | 30.55 | 4 | 63.64 | 28.00 | 7 | 81.82 | 29.27 | 10 | | | | | | |
| 2020 | Triamcinolone | 29 | 7.14 | 25.89 | 1 | 37.50 | 30.54 | 4 | 51.79 | 31.79 | 7 | 73.21 | 37.32 | 10 | | | | | | |
| Owlia | Penicillin | NA | | | | | | | | | | | | | | | | | | |
| 2020 | Placebo | NA | | | | | | | | | | | | | | | | | | |

| | | | | | | | | | | | | | | | | | | | | |
|---|---|---|---|---|---|---|---|---|---|---|---|---|---|---|---|---|---|---|---|---|
| Seyyedi | Laser | 5 | 77.05 | 11.40 | 0 | 73.70 | 6.70 | 0.5 | 77.05 | 6.70 | 1 | 56.95 | 15.58 | 2 | 73.70 | 18.09 | 3 | | | |
| 2020 | Laser | 5 | 79.72 | 16.98 | 0 | 58.93 | 16.12 | 0.5 | 58.93 | 16.12 | 1 | 69.32 | 18.20 | 2 | 83.19 | 6.59 | 3 | | | |
| | Triamcinolone | 5 | 36.22 | 9.86 | 0 | 48.29 | 20.72 | 0.5 | 52.31 | 19.72 | 1 | 68.41 | 28.17 | 2 | 80.48 | 26.16 | 3 | | | |
| Raman | Curcumin | NA | | | | | | | | | | | | | | | | | | |
| 2020 | Triamcinolone | NA | | | | | | | | | | | | | | | | | | |
| Bardellini | Laser | 30 | 75.00 | 41.50 | 4 | 75.00 | 46.00 | 7 | | | | | | | | | | | | |
| 2020 | Placebo | 30 | 25.00 | 45.75 | 4 | 75.00 | 45.00 | 7 | | | | | | | | | | | | |
| Nirmala | Probiotics | NA | | | | | | | | | | | | | | | | | | |
| 2019 | Triamcinolone | NA | | | | | | | | | | | | | | | | | | |
| Soliman | Laser | 10 | 50.00 | 87.00 | 2 | 59.00 | 86.50 | 3 | 91.82 | 18.34 | 4 | 100.00 | 0.00 | 6 | | | | | | |
| 2019 | Placebo | 10 | 0.00 | 0.00 | 2 | 8.00 | 14.00 | 3 | 8.00 | 13.98 | 4 | 48.00 | 31.55 | 6 | | | | | | |
| Tavangar | Triamcinolone | 20 | 0.00 | NA | 1 | 5.00 | NA | 2 | 25.00 | NA | 3 | 45.00 | NA | 4 | 70.00 | NA | 5 | 100.00 | NA | 6 |
| 2019 | Placebo | 20 | 0.00 | NA | 1 | 0.00 | NA | 2 | 5.00 | NA | 3 | 30.00 | NA | 4 | 50.00 | NA | 5 | 90.00 | NA | 6 |
| | | | 100.00 | NA | 7 | | | | | | | | | | | | | | | |
| | | | 100.00 | NA | 7 | | | | | | | | | | | | | | | |
| Halboub | N-acetylcysteine | 38 | 47.47 | 32.28 | 2 | 78.64 | 40.66 | 4 | 89.87 | 37.34 | 6 | | | | | | | | | |
| 2019 | Chlorhexidine | 20 | 31.82 | 35.27 | 2 | 62.73 | 49.82 | 4 | 80.91 | 39.27 | 6 | | | | | | | | | |
| El-Wakeel | Insulin-liposomal gel | 40 | 62.50 | 24.50 | 1 | 75.00 | 24.50 | 2 | 100.00 | 0.00 | 3 | 100.00 | 0.00 | 4 | 100.00 | 0.00 | 6 | | | |
| 2019 | Placebo | 40 | 0.00 | 0.00 | 1 | 12.50 | 18.50 | 2 | 25.00 | 18.50 | 3 | 62.50 | 27.75 | 4 | 50.00 | 18.50 | 6 | | | |
| Rahmani | Chitosan | 20 | -17.15 | NA | 3 | 57.85 | NA | 5 | 80.81 | NA | 7 | 100.00 | NA | 10 | | | | | | |
| 2018 | Triamcinolone | 20 | 39.73 | NA | 3 | 88.36 | NA | 5 | 97.26 | NA | 7 | 100.00 | NA | 10 | | | | | | |
| Sharma | Triamcinolone | 10 | 59.72 | 16.25 | 4 | 88.89 | 9.44 | 8 | 100.00 | 0.00 | 10 | | | | | | | | | |
| 2018 | Benzydamine | 10 | 49.18 | 18.69 | 4 | 100.00 | 10.98 | 8 | 100.00 | 0.00 | 10 | | | | | | | | | |
| | Doxycycline | 10 | 64.29 | 16.86 | 4 | 100.00 | 9.14 | 8 | 100.00 | 0.00 | 10 | | | | | | | | | |
| | Placebo | 10 | 38.81 | 17.46 | 4 | 100.00 | 11.04 | 8 | 88.06 | 9.55 | 10 | | | | | | | | | |
| Ofluoglu | Triamcinolone | 53 | 14.34 | 5.42 | 2 | 32.61 | 6.43 | 4 | 63.96 | 10.59 | 6 | | | | | | | | | |

| | | | | | | | | | | | | | | | | | | | | |
|---|---|---|---|---|---|---|---|---|---|---|---|---|---|---|---|---|---|---|---|---|
| 2017 | Triester Glycerol Oxide | 56 | 23.18 | 5.47 | 2 | 42.85 | 9.19 | 4 | 72.86 | 11.30 | 6 | | | | | | | | | |
| | Placebo | 51 | 9.03 | 6.76 | 2 | 28.63 | 8.12 | 4 | 61.21 | 10.40 | 6 | | | | | | | | | |
| Zeini | Laser | 14 | 1.71 | 3.02 | 0 | | | | | | | | | | | | | | | |
| 2017 | Placebo | 10 | 1.00 | 2.30 | 0 | | | | | | | | | | | | | | | |
| Rodríguez-Archilla | Silver nitrate | NA | | | | | | | | | | | | | | | | | | |
| 2017 | Honey | NA | | | | | | | | | | | | | | | | | | |
| | Placebo | NA | | | | | | | | | | | | | | | | | | |
| Pandya | Quercetin | 20 | 45.79 | 33.27 | 2 | 85.98 | 30.65 | 4 | 98.13 | 31.03 | 7 | | | | | | | | | |
| 2017 | Benzydamine | 20 | 34.34 | 36.57 | 2 | 67.68 | 39.39 | 4 | 90.91 | 30.51 | 7 | | | | | | | | | |
| Yilmaz | Laser | 40 | 97.59 | 22.89 | 0 | 90.36 | 22.53 | 1 | 95.18 | 24.22 | 3 | 98.80 | 23.73 | 7 | 100.00 | 0.00 | 10 | | | |
| 2017 | Placebo | 40 | 3.7. | 27.90 | 0 | 8.64 | 26.67 | 1 | 41.98 | 26.17 | 3 | 86.42 | 26.17 | 7 | 100.00 | 0.00 | 10 | | | |
| Nasry | Glycyrrhiza | 15 | 22.20 | 10.50 | 2 | 43.40 | 15.80 | 5 | | | | | | | | | | | | |
| 2016 | Amlexanox | 15 | 29.80 | 11.30 | 2 | 61.90 | 24.50 | 5 | | | | | | | | | | | | |
| | Laser | 15 | 43.30 | 20.00 | 2 | 67.80 | 21.50 | 5 | | | | | | | | | | | | |
| | Placebo | 15 | 4.10 | 0.50 | 2 | 6.20 | 1.60 | 5 | | | | | | | | | | | | |
| Abbasi | Dexamethasone | 20 | 61.83 | 33.02 | 3 | 88.36 | 33.97 | 5 | 100.00 | 0.00 | 7 | | | | | | | | | |
| 2016 | Amlexanox | 20 | 65.73 | 36.89 | 3 | 80.52 | 37.21 | 5 | 100.00 | 0.00 | 7 | | | | | | | | | |
| Andishe Tadbir | Placebo | 15 | -17.63 | 48.68 | 3 | 36.84 | 48.95 | 6 | | | | | | | | | | | | |
| 2015 | Triamcinolone | 14 | 58.70 | 28.26 | 3 | 89.61 | 28.50 | 6 | | | | | | | | | | | | |
| Raeesi | Glycyrrhiza | 20 | 55.43 | 11.88 | 1 | 92.96 | 10.26 | 3 | 100.00 | 0.00 | 5 | | | | | | | | | |
| 2015 | Placebo | 20 | 17.42 | 13.53 | 1 | 41.37 | 13.37 | 3 | 62.21 | 13.69 | 5 | | | | | | | | | |
| Aggarwal | Laser | NA | | | | | | | | | | | | | | | | | | |
| 2014 | Placebo | NA | | | | | | | | | | | | | | | | | | |
| Mansour | Aloe | 30 | 56.10 | NA | 4 | 76.45 | 45.61 | 6 | | | | | | | | | | | | |
| 2014 | Placebo | 30 | 30.74 | NA | 4 | 63.20 | 56.71 | 6 | | | | | | | | | | | | |
| Deshmukh | Curcumin | 30 | 29.79 | 32.34 | 1 | 55.32 | 33.19 | 2 | 76.60 | 30.21 | 3 | 91.49 | 28.30 | 4 | 98.72 | 30.64 | 5 | 100.00 | 0.00 | 6 |

| | | | | | | | | | | | | | | | | | | | | |
|---|---|---|---|---|---|---|---|---|---|---|---|---|---|---|---|---|---|---|---|---|
| 2014 | Triamcinolone | 30 | 26.67 | 34.89 | 1 | 53.33 | 35.78 | 2 | 75.56 | 33.11 | 3 | 88.89 | 28.67 | 4 | 95.56 | 27.56 | 5 | 98.67 | 29.33 | 6 |
| | | | 100.00 | 0.00 | 7 | | | | | | | | | | | | | | | |
| | | | 100.00 | 0.00 | 7 | | | | | | | | | | | | | | | |
| El-Haddad | Honey | 67 | 99.33 | NA | 1 | 100.00 | NA | 2 | 100.00 | NA | 3 | 100.00 | NA | 4 | 100.00 | NA | 5 | 100.00 | NA | 6 |
| 2014 | Triamcinolone | 57 | 25.81 | NA | 1 | 56.86 | NA | 2 | 90.79 | NA | 3 | 100.00 | NA | 4 | 100.00 | NA | 5 | 100.00 | NA | 6 |
| | Placebo | 56 | 3.57 | NA | 1 | 13.01 | NA | 2 | 23.53 | NA | 3 | 64.35 | NA | 4 | 83.78 | NA | 5 | 85.92 | NA | 6 |
| | | | 100.00 | NA | 7 | 100.00 | NA | 8 | | | | | | | | | | | | |
| | | | 100.00 | NA | 7 | 100.00 | NA | 8 | | | | | | | | | | | | |
| | | | 95.19 | NA | 7 | 100.00 | NA | 8 | | | | | | | | | | | | |
| Albrektson | Laser | 20 | 33.65 | NA | 1 | 62.81 | NA | 2 | | | | | | | | | | | | |
| 2014 | Placebo | 20 | 1.22 | NA | 1 | 6.85 | NA | 2 | | | | | | | | | | | | |
| Soylu | Silver nitrate | 35 | 23.65 | 9.39 | 1 | 55.80 | 8.84 | 3 | 94.70 | 8.18 | 7 | | | | | | | | | |
| 2014 | Placebo | 30 | 7.61 | 7.61 | 1 | 35.22 | 8.70 | 3 | 68.48 | 8.70 | 7 | | | | | | | | | |
| Soylu and Okuyucu | Sucralfate | 35 | 27.95 | 8.64 | 1 | 50.39 | 9.99 | 3 | 94.95 | 8.31 | 7 | | | | | | | | | |
| 2014 | Chlorhexidine | 35 | 16.00 | 9.06 | 1 | 31.32 | 9.73 | 3 | 88.81 | 11.86 | 7 | | | | | | | | | |
| Bhat | Amlexanox | 50 | 60.72 | 16.80 | 4 | 89.66 | 12.02 | 6 | | | | | | | | | | | | |
| 2013 | Placebo | 50 | 9.31 | 15.82 | 4 | 22.07 | 17.69 | 6 | | | | | | | | | | | | |
| Bhalang | Triamcinolone | 60 | 12.43 | 3.93 | 0 | 29.72 | 3.62 | 2 | 49.49 | 3.80 | 3 | 64.97 | 4.14 | 4 | 78.19 | 3.62 | 5 | 87.80 | 3.73 | 6 |
| 2013 | Aloe | 60 | 14.80 | 3.73 | 0 | 24.97 | 3.73 | 2 | 32.09 | 4.14 | 3 | 44.41 | 3.93 | 4 | 56.61 | 3.62 | 5 | 69.15 | 3.93 | 6 |
| | Placebo | 60 | 10.62 | 4.14 | 0 | 18.98 | 4.14 | 2 | 29.15 | 4.30 | 3 | 40.23 | 3.93 | 4 | 51.53 | 3.93 | 5 | 64.07 | 4.14 | 6 |
| | | | 93.11 | 4.14 | 7 | | | | | | | | | | | | | | | |
| | | | 82.37 | 3.93 | 7 | | | | | | | | | | | | | | | |
| | | | 75.48 | 3.93 | 7 | | | | | | | | | | | | | | | |
| Halim | Curcumin | 10 | 80.70 | 36.32 | 5 | | | | | | | | | | | | | | | |
| 2013 | Triamcinolone | 10 | 81.82 | 72.04 | 5 | | | | | | | | | | | | | | | |
| Jiang and Yang | Diosmectite | 35 | 9.75 | 16.10 | 2 | 24.39 | 21.99 | 3 | 42.68 | 23.74 | 4 | 51.22 | 17.20 | 5 | 56.10 | 16.13 | 6 | | | |

| | | | | | | | | | | | | | | | | | | | | |
|---|---|---|---|---|---|---|---|---|---|---|---|---|---|---|---|---|---|---|---|---|
| 2013 | Placebo | 30 | 5.13 | 21.92 | 2 | 14.10 | 23.08 | 3 | 24.36 | 25.77 | 4 | 35.90 | 22.69 | 5 | 38.46 | 21.28 | 6 | | | |
| Jiang and Zhu | Berberine gelatin | 42 | 16.10 | 16.71 | 2 | 44.57 | 17.20 | 4 | 76.65 | 16.83 | 6 | | | | | | | | | |
| 2013 | Placebo | 42 | 6.56 | 12.57 | 2 | 22.60 | 15.09 | 4 | 60.87 | 13.53 | 6 | | | | | | | | | |
| Prasad | Laser | 25 | 91.98 | 7.90 | 0 | 96.70 | 7.55 | 1 | | | | | | | | | | | | |
| 2013 | Placebo | 25 | 1.49 | 9.65 | 0 | 8.17 | 9.78 | 1 | | | | | | | | | | | | |
| Vijayabala | Doxycycline | 25 | 42.19 | 29.69 | 1 | 64.06 | 28.13 | 2 | 85.94 | 29.69 | 3 | 93.75 | 34.38 | 4 | 98.44 | 37.50 | 5 | 98.44 | 37.50 | 6 |
| 2013 | Placebo | 25 | 11.86 | 16.95 | 1 | 23.73 | 25.42 | 2 | 47.46 | 37.29 | 3 | 67.80 | 35.59 | 4 | 84.75 | 32.20 | 5 | 96.61 | 35.59 | 6 |
| | | | 98.44 | 35.94 | 7 | 100.00 | 3 | 8 | 100.00 | 0 | 9 | 100.00 | 0 | 10 | | | | | | |
| | | | 100.00 | 0.00 | 7 | 100.00 | 0 | 8 | 100.00 | 0 | 9 | 100.00 | 0 | 10 | | | | | | |
| Sattayut | Laser | 7 | 20.51 | 35.67 | 0 | 22.08 | 39.37 | 1 | 49.73 | 39.07 | 2 | | | | | | | | | |
| 2013 | Placebo | 7 | 41.99 | 38.61 | 0 | 25.37 | 35.16 | 1 | 38.47 | 41.04 | 2 | | | | | | | | | |
| Jiang | Allicin | 48 | 20.10 | 22.79 | 2 | 56.30 | 17.83 | 4 | 75.70 | 16.62 | 6 | | | | | | | | | |
| 2012 | Placebo | 48 | 13.90 | 29.37 | 2 | 33.60 | 28.42 | 4 | 52.00 | 23.22 | 6 | | | | | | | | | |
| Manifar | Curcumin | NA | | | | | | | | | | | | | | | | | | |
| 2012 | Placebo | NA | | | | | | | | | | | | | | | | | | |
| Liu | Dexamethasone | 114 | 95.09 | 33.16 | 6±2 | | | | | | | | | | | | | | | |
| 2012 | Placebo | 117 | 82.19 | 37.27 | 6±2 | | | | | | | | | | | | | | | |
| Babaee | Aloe | 20 | 19.33 | NA | 2 | 44.15 | NA | 3 | 64.36 | NA | 4 | 74.47 | NA | 5 | 79.61 | NA | 6 | 82.62 | NA | 7 |
| 2012 | Placebo | 20 | 13.11 | NA | 2 | 34.65 | NA | 3 | 44.34 | NA | 4 | 59.61 | NA | 5 | 69.66 | NA | 6 | 77.20 | NA | 7 |
| Zand | Laser | NA | | | | | | | | | | | | | | | | | | |
| 2012 | Placebo | NA | | | | | | | | | | | | | | | | | | |
| Galal | Glycyrrhiza | 10 | 14.60 | 10.20 | 2 | 22.90 | 40.30 | 5 | | | | | | | | | | | | |
| 2012 | Placebo | 10 | 4.00 | 0.70 | 2 | 6.00 | 1.20 | 5 | | | | | | | | | | | | |
| Trinchieri | Probiotics | 15 | 96.82 | NA | 7 | | | | | | | | | | | | | | | |
| 2011 | Placebo | 15 | 47.14 | NA | 7 | | | | | | | | | | | | | | | |
| Meng | Amlexanox | 108 | 56.12 | 43.88 | 4 | 80.97 | 43.31 | 6 | | | | | | | | | | | | |

| | | | | | | | | | | | | | | | | | | | | |
|---|---|---|---|---|---|---|---|---|---|---|---|---|---|---|---|---|---|---|---|---|
| 2009 | Placebo | 105 | 38.75 | 45.20 | 4 | 66.61 | 43.91 | 6 | | | | | | | | | | | | |
| Tezel | Triamcinolone | 10 | 19.82 | 9.33 | 1 | 51.94 | 8.81 | 4 | 93.01 | 8.29 | 7 | | | | | | | | | |
| 2009 | Laser | 10 | 82.97 | 9.78 | 1 | 97.71 | 8.77 | 4 | 100.00 | 0.00 | 7 | | | | | | | | | |
| Skulason | Doxycycline | 25 | 23.53 | 48.04 | 2 | 43.14 | 53.33 | 3 | | | | | | | | | | | | |
| 2009 | Placebo | 24 | 9.43 | 48.11 | 2 | 24.53 | 51.32 | 3 | | | | | | | | | | | | |
| Zand | Laser | 15 | 98.87 | 19.03 | 0 | 92.74 | 19.35 | 4h | 90.32 | 18.55 | 8h | 90 | 18.87 | 12h | 92.10 | 19.03 | 24h | 91.61 | 18.55 | 48h |
| 2009 | Placebo | 15 | 3.33 | 21.48 | 0 | -0.37 | 21.30 | 4h | 0.00 | 21.48 | 8h | 0.37 | 21.30 | 12h | -3.15 | 20.93 | 24h | 12.96 | 20.93 | 48h |
| | | | 95.97 | 19.19 | 72h | 96.29 | 19.35 | 96h | | | | | | | | | | | | |
| | | | 25.19 | 21.11 | 72h | 42.04 | 20.93 | 96h | | | | | | | | | | | | |
| Moghadamnia | Glycyrrhiza | 15 | -86.44 | 24.29 | 1 | -97.74 | 23.16 | 2 | -40.11 | 24.29 | 3 | 18.64 | 23.16 | 4 | 57.06 | 23.16 | 5 | | | |
| 2009 | Placebo | 15 | -52.78 | 17.06 | 1 | -42.46 | 16.67 | 2 | -15.08 | 16.67 | 3 | 25.40 | 17.06 | 4 | 48.81 | 17.06 | 5 | | | |
| Gorsky | Minocycline | 18 | 20.41 | NA | 1 | 55.51 | NA | 2 | 69.39 | NA | 3 | 73.47 | NA | 4 | 79.59 | NA | 5 | 79.59 | NA | 6 |
| 2008 | Placebo | 15 | -2.78 | NA | 1 | -38.89 | NA | 2 | -55.56 | NA | 3 | -66.67 | NA | 4 | -66.67 | NA | 5 | -61.11 | NA | 6 |
| | | | 79.59 | NA | 7 | 79.59 | NA | 8 | 79.59 | NA | 9 | 79.59 | NA | 10 | | | | | | |
| | | | -50 | NA | 7 | -22.22 | NA | 8 | -8.33 | NA | 9 | 16.67 | NA | 10 | | | | | | |
| Rodríguez | Clobetasol | 48 | 50.29 | NA | 2 | 84.35 | NA | 5 | | | | | | | | | | | | |
| 2007 | Amlexanox | 48 | 45.95 | NA | 2 | 74.16 | NA | 5 | | | | | | | | | | | | |
| Arikan | Cryotherapy | NA | | | | | | | | | | | | | | | | | | |
| 2006 | Placebo | NA | | | | | | | | | | | | | | | | | | |
| Liu | Amlexanox | 104 | 58.00 | 44.89 | 4 | 90.00 | 39.33 | 6 | | | | | | | | | | | | |
| 2006 | Placebo | 108 | 43.00 | 48.77 | 4 | 76.54 | 43.21 | 6 | | | | | | | | | | | | |
| Garnick | Placebo | NA | | | | | | | | | | | | | | | | | | |
| 1998 | Aloe | NA | | | | | | | | | | | | | | | | | | |
| Khandwala | Placebo | 512 | 36.63 | 3.02 | 3 | 49.88 | 3.25 | 4 | 65.66 | 3.48 | 5 | | | | | | | | | |
| 1997 | Amlexanox | 579 | 42.08 | 2.72 | 3 | 66.09 | 3.22 | 4 | 81.93 | 2.97 | 5 | | | | | | | | | |
| Ylikontiola | Doxycycline | 15 | 38.00 | 14.40 | 1 | 61.60 | 10.00 | 2 | 76.60 | 5.80 | 3 | 82.60 | 3.60 | 4 | 84.40 | 5.20 | 5 | 90.00 | 5.20 | 6 |

| | | | | | | | | | | | | | | | | | | | | |
|---|---|---|---|---|---|---|---|---|---|---|---|---|---|---|---|---|---|---|---|---|
| 1997 | Placebo | 16 | 16.00 | 13.00 | 1 | 22.00 | 9.20 | 2 | 27.40 | 10.40 | 3 | 39.40 | 10.40 | 4 | 57.80 | 9.20 | 5 | 71.20 | 8.20 | 6 |
| | | | 95.20 | 3.00 | 7 | 94.80 | 2.60 | 8 | 98.40 | 1.60 | 9 | 99.60 | 0.60 | 10 | | | | | | |
| | | | 80.40 | 7.00 | 7 | 94.40 | 4.40 | 8 | 98.80 | 1.20 | 9 | 100.00 | 0.00 | 10 | | | | | | |
| Greer Jr | Amlexanox | NA | | | | | | | | | | | | | | | | | | |
| 1993 | Placebo | NA | | | | | | | | | | | | | | | | | | |
| Miles | Triamcinolone | 6 | 32.79 | 21.31 | 2 | 48.91 | 21.86 | 3 | 74.59 | 21.31 | 4 | 77.60 | 21.04 | 5 | 86.34 | 21.58 | 6 | 95.36 | 22.95 | 7 |
| 1993 | Chlorhexidine | 6 | 38.67 | 20.26 | 2 | 78.27 | 18.23 | 3 | 84.71 | 18.23 | 4 | 86.56 | 18.05 | 5 | 90.24 | 18.05 | 6 | 90.42 | 18.05 | 7 |
| | | | 98.36 | 23.77 | 8 | 99.73 | 24.32 | 9 | 100.00 | 0 | 10 | | | | | | | | | |
| | | | 100.00 | 0.00 | 8 | 100.00 | 0.00 | 9 | 100.00 | 0 | 10 | | | | | | | | | |
| Taylor | Prostaglandin E2 | 18 | -4.86 | NA | 2 | -0.4 | NA | 3 | -10.53 | NA | 4 | 19.84 | NA | 5 | 50.61 | NA | 6 | 68.42 | NA | 7 |
| 1993 | Placebo | 15 | -16.67 | NA | 2 | -14.78 | NA | 3 | 10.38 | NA | 4 | 22.96 | NA | 5 | 42.45 | NA | 6 | 63.52 | NA | 7 |
| | | | 78.95 | NA | 8 | 87.85 | NA | 9 | 89.96 | NA | 10 | | | | | | | | | |
| | | | 64.47 | NA | 8 | 83.96 | NA | 9 | 88.36 | NA | 10 | | | | | | | | | |
| Hunter | Chlorhexidine | NA | | | | | | | | | | | | | | | | | | |
| 1987 | Placebo | NA | | | | | | | | | | | | | | | | | | |
| Matthews | Benzydamine | NA | | | | | | | | | | | | | | | | | | |
| 1987 | Chlorhexidine | NA | | | | | | | | | | | | | | | | | | |
| Yeoman | Betamethasone | NA | | | | | | | | | | | | | | | | | | |
| 1978 | Placebo | NA | | | | | | | | | | | | | | | | | | |
| Addy | Chlorhexidine | NA | | | | | | | | | | | | | | | | | | |
| 1976 | Placebo | NA | | | | | | | | | | | | | | | | | | |
| Addy | Zinc | NA | | | | | | | | | | | | | | | | | | |
| 1974 | Placebo | NA | | | | | | | | | | | | | | | | | | |
| Browne | Placebo | NA | | | | | | | | | | | | | | | | | | |
| 1968 | Triamcinolone | NA | | | | | | | | | | | | | | | | | | |

## Table S10 Details information of studies for each outcome in network meta-analysis (3)

| Author Year | Treatment | Healing effect (days) | | | Adverse event occurrence | |
|---|---|---|---|---|---|---|
| | | Mean | SD | Total | Events | Total |
| Huo | Laser | 6.60 | 0.29 | 25 | NA | |
| 2021 | Triamcinolone | 7.77 | 0.52 | 26 | NA | |
| Aggour (audlt) | Probiotics | NA | | | NA | |
| 2021 | Placebo | NA | | | NA | |
| Aggour (child) | Probiotics | NA | | | NA | |
| 2021 | Placebo | NA | | | NA | |
| Shi | Aloe | 7.40 | 1.85 | 20 | NA | |
| 2020 | Chitosan | 7.93 | 1.84 | 15 | NA | |
| Kavita | Triamcinolone | NA | | | NA | |
| 2020 | Amlexanox | NA | | | NA | |
| Shao | Chitosan | NA | | | 0 | 34 |
| 2020 | Placebo | NA | | | 0 | 32 |
| Pedersen | Probiotics | NA | | | 0 | 10 |
| 2020 | Placebo | NA | | | 0 | 10 |
| Ghorbani | Zinc | NA | | | 0 | 23 |
| 2020 | Placebo | NA | | | 0 | 23 |
| Ibrahim | Triamcinolone | 7.75 | 2.88 | 20 | 0 | 20 |
| 2020 | Probiotics | 5.40 | 1.40 | 20 | 0 | 20 |
| Kia | Curcumin | NA | | | 0 | 29 |
| 2020 | Triamcinolone | NA | | | 0 | 29 |
| Owlia | Penicillin | 2.79 | 0.16 | 14 | 3 | 25 |
| 2020 | Placebo | 5.90 | 0.93 | 12 | 0 | 25 |

| | | | | | | |
|---|---|---|---|---|---|---|
| Seyyedi | Laser | NA | | | NA | |
| 2020 | Laser | NA | | | NA | |
| | Triamcinolone | NA | | | NA | |
| Raman | Curcumin | 7.21 | 0.08 | 30 | 0 | 30 |
| 2020 | Triamcinolone | 6.77 | 0.08 | 30 | 0 | 30 |
| Bardellini | Laser | NA | | | NA | |
| 2020 | Placebo | NA | | | NA | |
| Nirmala | Probiotics | NA | | | NA | |
| 2019 | Triamcinolone | NA | | | NA | |
| Soliman | Laser | NA | | | NA | |
| 2019 | Placebo | NA | | | NA | |
| Tavangar | Triamcinolone | 5.75 | 1.08 | 20 | NA | |
| 2019 | Placebo | 6.50 | 1.05 | 20 | NA | |
| Halboub | N-acetylcysteine | 4.87 | 2.46 | 38 | NA | |
| 2019 | Chlorhexidine | 5.40 | 2.82 | 20 | NA | |
| El-Wakeel | Insulin-liposomal gel | 4.20 | 0.60 | 40 | NA | |
| 2019 | Placebo | 8.10 | 1.00 | 40 | NA | |
| Rahmani | Chitosan | 6.20 | 1.82 | 20 | 1 | 20 |
| 2018 | Triamcinolone | 5.15 | 1.42 | 20 | 0 | 20 |
| Sharma | Triamcinolone | NA | | | 0 | 10 |
| 2018 | Benzydamine | NA | | | 0 | 10 |
| | Doxycycline | NA | | | 0 | 10 |
| | Placebo | NA | | | 0 | 10 |
| Ofluoglu | Triamcinolone | NA | | | 0 | 53 |
| 2017 | Triester Glycerol Oxide | NA | | | 0 | 56 |
| | Placebo | NA | | | 0 | 51 |

| | | | | | | |
|---|---|---|---|---|---|---|
| Zeini | Laser | 7.21 | 4.20 | 14 | NA | |
| 2017 | Placebo | 6.40 | 1.83 | 10 | NA | |
| Rodríguez-Archilla | Silver nitrate | 7.32 | 0.99 | 25 | 0 | 25 |
| 2017 | Honey | 6.80 | 0.71 | 25 | 0 | 25 |
| | Placebo | 8.96 | 0.89 | 25 | 0 | 25 |
| Pandya | Quercetin | NA | | | NA | |
| 2017 | Benzydamine | NA | | | NA | |
| Yilmaz | Laser | NA | | | 0 | 40 |
| 2017 | Placebo | NA | | | 0 | 40 |
| Nasry | Glycyrrhiza | NA | | | NA | |
| 2016 | Amlexanox | NA | | | NA | |
| | Laser | NA | | | NA | |
| | Placebo | NA | | | NA | |
| Abbasi | Dexamethasone | NA | | | NA | |
| 2016 | Amlexanox | NA | | | NA | |
| Andishe Tadbir | Placebo | 6.58 | NA | 15 | NA | |
| 2015 | Triamcinolone | 4.22 | NA | 14 | NA | |
| Raeesi | Glycyrrhiza | 4.20 | 1.30 | 20 | 0 | 20 |
| 2015 | Placebo | 7.45 | 1.30 | 20 | 0 | 20 |
| Aggarwal | Laser | 3.05 | 1.10 | 30 | NA | |
| 2014 | Placebo | 8.90 | 2.45 | 30 | NA | |
| Mansour | Aloe | NA | | | 0 | 30 |
| 2014 | Placebo | NA | | | 0 | 30 |
| Deshmukh | Curcumin | 3.90 | 1.18 | 30 | 0 | 30 |
| 2014 | Triamcinolone | 4.10 | 1.18 | 30 | 0 | 30 |
| El-Haddad | Honey | 2.73 | 0.57 | 67 | 0 | 67 |

| | | | | | | |
|---|---|---|---|---|---|---|
| 2014 | Triamcinolone | 5.91 | 0.91 | 57 | 0 | 57 |
| | Placebo | 7.14 | 0.92 | 56 | 0 | 56 |
| Albrektson | Laser | NA | | | 0 | 20 |
| 2014 | Placebo | NA | | | 0 | 20 |
| Soylu | Silver nitrate | 2.70 | 1.48 | 35 | 0 | 35 |
| 2014 | Placebo | 5.50 | 2.22 | 30 | 0 | 30 |
| Soylu and Okuyucu | Sucralfate | 1.97 | 1.56 | 23 | 0 | 35 |
| 2014 | Chlorhexidine | 2.80 | 3.00 | 19 | 0 | 35 |
| Bhat | Amlexanox | NA | | | 8 | 50 |
| 2013 | Placebo | NA | | | 0 | 50 |
| Bhalang | Triamcinolone | NA | | | 0 | 60 |
| 2013 | Aloe | NA | | | 0 | 60 |
| | Placebo | NA | | | 0 | 60 |
| Halim | Curcumin | NA | | | NA | |
| 2013 | Triamcinolone | NA | | | NA | |
| Jiang and Yang | Diosmectite | NA | | | 0 | 35 |
| 2013 | Placebo | NA | | | 0 | 30 |
| Jiang and Zhu | Berberine gelatin | NA | | | 0 | 42 |
| 2013 | Placebo | NA | | | 0 | 42 |
| Prasad | Laser | 4.08 | 0.81 | 25 | NA | |
| 2013 | Placebo | 7.84 | 0.90 | 25 | NA | |
| Vijayabala | Doxycycline | 3.70 | 1.30 | 25 | 8 | 25 |
| 2013 | Placebo | 5.30 | 1.20 | 25 | 0 | 25 |
| Sattayut | Laser | NA | | | NA | |
| 2013 | Placebo | NA | | | NA | |
| Jiang | Allicin | NA | | | 0 | 48 |

| | | | | | | |
|---|---|---|---|---|---|---|
| 2012 | Placebo | NA | | | 0 | 48 |
| Manifar | Curcumin | NA | | | 0 | 28 |
| 2012 | Placebo | NA | | | 0 | 29 |
| Liu | Dexamethasone | 6.00 | NA | 114 | 4 | 120 |
| 2012 | Placebo | 7.00 | NA | 117 | 8 | 120 |
| Babaee | Aloe | NA | | | NA | |
| 2012 | Placebo | NA | | | NA | |
| Zand | Laser | 4.80 | 2.40 | 10 | 0 | 10 |
| 2012 | Placebo | 7.60 | 2.50 | 10 | 0 | 10 |
| Galal | Glycyrrhiza | NA | | | NA | |
| 2012 | Placebo | NA | | | NA | |
| Trinchieri | Probiotics | NA | | | 0 | 15 |
| 2011 | Placebo | NA | | | 0 | 15 |
| Meng | Amlexanox | NA | | | 0 | 108 |
| 2009 | Placebo | NA | | | 0 | 105 |
| Tezel | Triamcinolone | NA | | | 0 | 10 |
| 2009 | Laser | NA | | | 0 | 10 |
| Skulason | Doxycycline | NA | | | 1 | 25 |
| 2009 | Placebo | NA | | | 0 | 24 |
| Zand | Laser | NA | | | 0 | 15 |
| 2009 | Placebo | NA | | | 0 | 15 |
| Moghadamnia | Glycyrrhiza | 8.46 | 1.55 | 15 | NA | |
| 2009 | Placebo | 8.60 | 1.68 | 15 | NA | |
| Gorsky | Minocycline | NA | | | 0 | 18 |
| 2008 | Placebo | NA | | | 0 | 15 |
| Rodríguez | Clobetasol | NA | | | 0 | 48 |

| | | | | | | |
|---|---|---|---|---|---|---|
| 2007 | Amlexanox | NA | | | 0 | 48 |
| Arikan | Cryotherapy | NA | | | NA | |
| 2006 | Placebo | NA | | | NA | |
| Liu | Amlexanox | NA | | | 1 | 104 |
| 2006 | Placebo | NA | | | 1 | 108 |
| Garnick | Placebo | 9.01 | 1.32 | 4 | NA | |
| 1998 | Aloe | 6.38 | 0.73 | 3 | NA | |
| Khandwala | Placebo | 4.90 | NA | 512 | NA | |
| 1997 | Amlexanox | 5.60 | NA | 579 | NA | |
| Ylikontiola | Doxycycline | NA | | | NA | |
| 1997 | Placebo | NA | | | NA | |
| Greer Jr | Amlexanox | NA | | | 0 | 18 |
| 1993 | Placebo | NA | | | 0 | 14 |
| Miles | Triamcinolone | 5.93 | NA | 6 | NA | |
| 1993 | Chlorhexidine | 5.38 | NA | 6 | NA | |
| Taylor | Prostaglandin E2 | 7.78 | 2.44 | 18 | NA | |
| 1993 | Placebo | 8.27 | 1.87 | 15 | NA | |
| Hunter | Chlorhexidine | 5.02 | 2.41 | 38 | NA | |
| 1987 | Placebo | 5.78 | 2.74 | 38 | NA | |
| Matthews | Benzydamine | NA | | | NA | |
| 1987 | Chlorhexidine | NA | | | NA | |
| Yeoman | Betamethasone | 8.50 | NA | 10 | NA | |
| 1978 | Placebo | 15.00 | NA | 10 | NA | |
| Addy | Chlorhexidine | 4.80 | 2.90 | 20 | NA | |
| 1976 | Placebo | 7.80 | 2.90 | 20 | NA | |
| Addy | Zinc | 21.80 | 11.50 | 12 | NA | |

| | | | | | |
|---|---|---|---|---|---|
| 1974 | Placebo | 16.80 | 6.20 | 12 | NA |
| Browne | Placebo | 8.21 | NA | 26 | NA |
| 1968 | Triamcinolone | 7.53 | NA | 26 | NA |

## Chapter S1 Healing effect

## 1.Consistency Model

### Summary estimates for Healing effect

The estimate values for healing effect are given as mean difference (MD) and 95% confidence interval (CI). The table shows the pooled estimates based on the network meta-analysis during the treatment period for each study.

| | | | | | | | | | |
|---|---|---|---|---|---|---|---|---|---|
| Aloe | 1.15 (-2.06, 4.38) | 0.21 (-4.14, 4.50) | 0.79 (-3.48, 5.19) | 0.38 (-4.64, 5.29) | 0.20 (-3.88, 4.46) | -1.56 (-5.60, 2.45) | -1.93 (-6.87, 2.94) | -1.10 (-4.70, 2.63) | -0.36 (-6.18, 5.29) |
| -1.15 (-4.38, 2.06) | Chitosan | -0.94 (-5.43, 3.33) | -0.36 (-4.43, 3.80) | -0.79 (-6.01, 4.21) | -0.93 (-5.30, 3.34) | -2.70 (-6.76, 1.25) | -3.08 (-8.15, 1.92) | -2.24 (-5.96, 1.57) | -1.47 (-7.32, 4.24) |
| -0.21 (-4.50, 4.14) | 0.94 (-3.33, 5.43) | Chlorhexidine | 0.63 (-3.62, 4.87) | 0.18 (-4.45, 4.76) | 0.01 (-3.87, 3.94) | -1.74 (-5.37, 1.95) | -2.12 (-6.75, 2.49) | -1.30 (-4.43, 2.14) | -0.55 (-4.36, 3.35) |
| -0.79 (-5.19, 3.48) | 0.36 (-3.80, 4.43) | -0.63 (-4.87, 3.62) | Curcumin | -0.43 (-5.49, 4.45) | -0.61 (-4.75, 3.64) | -2.36 (-6.18, 1.49) | -2.75 (-7.78, 2.06) | -1.92 (-5.25, 1.57) | -1.13 (-6.99, 4.46) |
| -0.38 (-5.29, 4.64) | 0.79 (-4.21, 6.01) | -0.18 (-4.76, 4.45) | 0.43 (-4.45, 5.49) | Doxycycline | -0.16 (-4.73, 4.45) | -1.95 (-6.28, 2.51) | -2.29 (-7.47, 2.94) | -1.47 (-5.41, 2.81) | -0.67 (-6.72, 5.38) |
| -0.20 (-4.46, 3.88) | 0.93 (-3.34, 5.30) | -0.01 (-3.94, 3.87) | 0.61 (-3.64, 4.75) | 0.16 (-4.45, 4.73) | Glycyrrhiza | -1.77 (-5.38, 1.84) | -2.14 (-6.74, 2.50) | -1.31 (-4.43, 1.94) | -0.57 (-6.14, 4.91) |
| 1.56 (-2.45, 5.60) | 2.70 (-1.25, 6.76) | 1.74 (-1.95, 5.37) | 2.36 (-1.49, 6.18) | 1.95 (-2.51, 6.28) | 1.77 (-1.84, 5.38) | Honey | -0.37 (-4.82, 3.92) | 0.46 (-2.32, 3.32) | 1.24 (-4.02, 6.50) |
| 1.93 (-2.94, 6.87) | 3.08 (-1.92, 8.15) | 2.12 (-2.49, 6.75) | 2.75 (-2.06, 7.78) | 2.29 (-2.94, 7.47) | 2.14 (-2.50, 6.74) | 0.37 (-3.92, 4.82) | Insulin-liposomal gel | 0.82 (-3.11, 4.93) | 1.58 (-4.51, 7.69) |
| 1.10 (-2.63, 4.70) | 2.24 (-1.57, 5.96) | 1.30 (-2.14, 4.43) | 1.92 (-1.57, 5.25) | 1.47 (-2.81, 5.41) | 1.31 (-1.94, 4.43) | -0.46 (-3.32, 2.32) | -0.82 (-4.93, 3.11) | Laser | 0.77 (-4.38, 5.84) |
| 0.36 (-5.29, 6.18) | 1.47 (-4.24, 7.32) | 0.55 (-3.35, 4.36) | 1.13 (-4.46, 6.99) | 0.67 (-5.38, 6.72) | 0.57 (-4.91, 6.14) | -1.24 (-6.50, 4.02) | -1.58 (-7.69, 4.51) | -0.77 (-5.84, 4.38) | N-acetylcysteine |
| 1.12 (-3.82, 6.23) | 2.27 (-2.72, 7.35) | 1.31 (-3.30, 5.97) | 1.95 (-2.97, 6.92) | 1.51 (-3.82, 6.78) | 1.33 (-3.13, 5.97) | -0.45 (-4.63, 4.00) | -0.78 (-5.92, 4.55) | 0.01 (-3.88, 4.28) | 0.78 (-5.31, 6.83) |
| -1.96 (-5.26, 1.29) | -0.85 (-4.23, 2.65) | -1.78 (-4.55, 0.96) | -1.16 (-4.38, 2.07) | -1.60 (-5.39, 2.06) | -1.75 (-4.38, 0.95) | -3.55 (-5.90, -1.13) | -3.90 (-7.53, -0.23) | -3.08 (-4.81, -1.19) | -2.31 (-7.06, 2.40) |
| 1.70 (-3.59, 6.82) | 2.82 (-2.30, 7.67) | 1.88 (-3.37, 6.94) | 2.47 (-2.23, 7.04) | 2.02 (-3.97, 7.74) | 1.89 (-3.38, 6.76) | 0.12 (-4.71, 4.75) | -0.26 (-6.18, 5.33) | 0.57 (-4.06, 5.03) | 1.38 (-5.27, 7.60) |
| -1.47 (-6.57, 3.69) | -0.34 (-5.49, 5.04) | -1.31 (-6.05, 3.49) | -0.65 (-5.68, 4.54) | -1.11 (-6.61, 4.20) | -1.28 (-6.00, 3.52) | -3.04 (-7.53, 1.59) | -3.43 (-8.70, 1.96) | -2.60 (-6.76, 1.85) | -1.80 (-7.90, 4.26) |
| 0.54 (-3.51, 4.72) | 1.72 (-2.48, 5.90) | 0.74 (-3.06, 4.41) | 1.35 (-2.59, 5.44) | 0.89 (-3.57, 5.42) | 0.75 (-2.98, 4.50) | -1.02 (-3.92, 1.93) | -1.37 (-5.98, 3.04) | -0.55 (-3.56, 2.54) | 0.21 (-5.02, 5.52) |
| 0.67 (-5.08, 6.39) | 1.81 (-3.99, 7.76) | 0.84 (-3.10, 4.76) | 1.48 (-4.27, 7.29) | 1.01 (-5.18, 7.04) | 0.91 (-4.67, 6.33) | -0.89 (-6.24, 4.47) | -1.25 (-7.35, 4.75) | -0.46 (-5.53, 4.78) | 0.29 (-5.17, 5.84) |
| -0.69 (-4.20, 2.70) | 0.44 (-2.75, 3.60) | -0.51 (-3.97, 2.88) | 0.11 (-2.45, 2.64) | -0.33 (-4.66, 3.92) | -0.51 (-3.77, 2.84) | -2.25 (-5.07, 0.47) | -2.63 (-6.94, 1.52) | -1.80 (-4.03, 0.52) | -1.02 (-6.29, 4.13) |
| -7.24 (-15.78, 1.88) | -6.08 (-14.87, 3.27) | -7.01 (-15.71, 1.92) | -6.39 (-15.05, 2.90) | -6.88 (-15.68, 2.53) | -7.00 (-15.57, 2.03) | -8.82 (-17.19, 0.14) | -9.16 (-18.13, 0.15) | -8.32 (-16.60, 0.64) | -7.49 (-17.12, 2.25) |

| | | | | | | | |
|---|---|---|---|---|---|---|---|
| -1.12 (-6.23, 3.82) | 1.96 (-1.29, 5.26) | -1.70 (-6.82, 3.59) | 1.47 (-3.69, 6.57) | -0.54 (-4.72, 3.51) | -0.67 (-6.39, 5.08) | 0.69 (-2.70, 4.20) | 7.24 (-1.88, 15.78) |
| -2.27 (-7.35, 2.72) | 0.85 (-2.65, 4.23) | -2.82 (-7.67, 2.30) | 0.34 (-5.04, 5.49) | -1.72 (-5.90, 2.48) | -1.81 (-7.76, 3.99) | -0.44 (-3.60, 2.75) | 6.08 (-3.27, 14.87) |
| -1.31 (-5.97, 3.30) | 1.78 (-0.96, 4.55) | -1.88 (-6.94, 3.37) | 1.31 (-3.49, 6.05) | -0.74 (-4.41, 3.06) | -0.84 (-4.76, 3.10) | 0.51 (-2.88, 3.97) | 7.01 (-1.92, 15.71) |
| -1.95 (-6.92, 2.97) | 1.16 (-2.07, 4.38) | -2.47 (-7.04, 2.23) | 0.65 (-4.54, 5.68) | -1.35 (-5.44, 2.59) | -1.48 (-7.29, 4.27) | -0.11 (-2.64, 2.45) | 6.39 (-2.90, 15.05) |
| -1.51 (-6.78, 3.82) | 1.60 (-2.06, 5.39) | -2.02 (-7.74, 3.97) | 1.11 (-4.20, 6.61) | -0.89 (-5.42, 3.57) | -1.01 (-7.04, 5.18) | 0.33 (-3.92, 4.66) | 6.88 (-2.53, 15.68) |
| -1.33 (-5.97, 3.13) | 1.75 (-0.95, 4.38) | -1.89 (-6.76, 3.38) | 1.28 (-3.52, 6.00) | -0.75 (-4.50, 2.98) | -0.91 (-6.33, 4.67) | 0.51 (-2.84, 3.77) | 7.00 (-2.03, 15.57) |
| 0.45 (-4.00, 4.63) | 3.55 (1.13, 5.90) | -0.12 (-4.75, 4.71) | 3.04 (-1.59, 7.53) | 1.02 (-1.93, 3.92) | 0.89 (-4.47, 6.24) | 2.25 (-0.47, 5.07) | 8.82 (-0.14, 17.19) |
| 0.78 (-4.55, 5.92) | 3.90 (0.23, 7.53) | 0.26 (-5.33, 6.18) | 3.43 (-1.96, 8.70) | 1.37 (-3.04, 5.98) | 1.25 (-4.75, 7.35) | 2.63 (-1.52, 6.94) | 9.16 (-0.15, 18.13) |
| -0.01 (-4.28, 3.88) | 3.08 (1.19, 4.81) | -0.57 (-5.03, 4.06) | 2.60 (-1.85, 6.76) | 0.55 (-2.54, 3.56) | 0.46 (-4.78, 5.53) | 1.80 (-0.52, 4.03) | 8.32 (-0.64, 16.60) |
| -0.78 (-6.83, 5.31) | 2.31 (-2.40, 7.06) | -1.38 (-7.60, 5.27) | 1.80 (-4.26, 7.90) | -0.21 (-5.52, 5.02) | -0.29 (-5.84, 5.17) | 1.02 (-4.13, 6.29) | 7.49 (-2.25, 17.12) |
| Penicillin | 3.09 (-0.59, 6.84) | -0.55 (-6.18, 5.34) | 2.63 (-2.93, 7.93) | 0.58 (-3.91, 5.01) | 0.47 (-5.53, 6.50) | 1.84 (-2.31, 6.11) | 8.34 (-1.08, 17.23) |
| -3.09 (-6.84, 0.59) | Placebo | -3.65 (-7.85, 0.89) | -0.47 (-4.45, 3.37) | -2.53 (-5.05, 0.08) | -2.63 (-7.39, 2.20) | -1.28 (-3.26, 0.77) | 5.23 (-3.48, 13.40) |
| 0.55 (-5.34, 6.18) | 3.65 (-0.89, 7.85) | Probiotics | 3.11 (-2.90, 8.88) | 1.11 (-4.06, 6.09) | 1.01 (-5.54, 7.36) | 2.38 (-1.61, 6.18) | 8.88 (-1.03, 18.07) |
| -2.63 (-7.93, 2.93) | 0.47 (-3.37, 4.45) | -3.11 (-8.88, 2.90) | Prostaglandin E2 | -2.03 (-6.67, 2.76) | -2.16 (-8.41, 4.01) | -0.78 (-5.12, 3.72) | 5.71 (-3.54, 14.75) |
| -0.58 (-5.01, 3.91) | 2.53 (-0.08, 5.05) | -1.11 (-6.09, 4.06) | 2.03 (-2.76, 6.67) | Silver nitrate | -0.09 (-5.57, 5.37) | 1.23 (-1.87, 4.41) | 7.82 (-1.24, 16.25) |
| -0.47 (-6.50, 5.53) | 2.63 (-2.20, 7.39) | -1.01 (-7.36, 5.54) | 2.16 (-4.01, 8.41) | 0.09 (-5.37, 5.57) | Sucralfate | 1.39 (-3.86, 6.65) | 7.89 (-2.04, 17.34) |
| -1.84 (-6.11, 2.31) | 1.28 (-0.77, 3.26) | -2.38 (-6.18, 1.61) | 0.78 (-3.72, 5.12) | -1.23 (-4.41, 1.87) | -1.39 (-6.65, 3.86) | Triamcinolone | 6.49 (-2.44, 14.86) |
| -8.34 (-17.23, 1.08) | -5.23 (-13.40, 3.48) | -8.88 (-18.07, 1.03) | -5.71 (-14.75, 3.54) | -7.82 (-16.25, 1.24) | -7.89 (-17.34, 2.04) | -6.49 (-14.86, 2.44) | Zinc |

# Rank probability

**Rank 1 is worst, rank N is best.**

| Drug | Rank 1 | Rank 2 | Rank 3 | Rank 4 | Rank 5 | Rank 6 | Rank 7 | Rank 8 | Rank 9 | Rank 10 | Rank 11 | Rank 12 | Rank 13 | Rank 14 | Rank 15 | Rank 16 | Rank 17 | Rank 18 |
|---|---|---|---|---|---|---|---|---|---|---|---|---|---|---|---|---|---|---|
| Aloe | 0 | 0.03 | 0.05 | 0.05 | 0.06 | 0.07 | 0.07 | 0.08 | 0.08 | 0.08 | 0.08 | 0.08 | 0.07 | 0.06 | 0.05 | 0.05 | 0.04 | 0.02 |
| Chitosan | 0.03 | 0.14 | 0.11 | 0.11 | 0.1 | 0.09 | 0.08 | 0.07 | 0.06 | 0.05 | 0.04 | 0.04 | 0.03 | 0.02 | 0.02 | 0.01 | 0.01 | 0 |
| Chlorhexidine | 0 | 0.02 | 0.04 | 0.06 | 0.07 | 0.08 | 0.09 | 0.09 | 0.1 | 0.09 | 0.09 | 0.08 | 0.06 | 0.05 | 0.04 | 0.02 | 0.01 | 0 |
| Curcumin | 0.02 | 0.08 | 0.09 | 0.09 | 0.09 | 0.1 | 0.1 | 0.08 | 0.07 | 0.06 | 0.05 | 0.04 | 0.04 | 0.03 | 0.02 | 0.01 | 0.01 | 0 |
| Doxycycline | 0.02 | 0.08 | 0.07 | 0.07 | 0.07 | 0.07 | 0.07 | 0.07 | 0.07 | 0.07 | 0.06 | 0.06 | 0.05 | 0.05 | 0.04 | 0.04 | 0.03 | 0.02 |
| Glycyrrhiza | 0.01 | 0.03 | 0.04 | 0.06 | 0.07 | 0.08 | 0.09 | 0.09 | 0.09 | 0.09 | 0.08 | 0.07 | 0.06 | 0.05 | 0.04 | 0.03 | 0.02 | 0.01 |
| Honey | 0 | 0 | 0 | 0 | 0 | 0.01 | 0.01 | 0.02 | 0.03 | 0.04 | 0.06 | 0.07 | 0.09 | 0.12 | 0.14 | 0.17 | 0.15 | 0.09 |
| Insulin-liposomal gel | 0 | 0.01 | 0.01 | 0.01 | 0.01 | 0.02 | 0.02 | 0.02 | 0.03 | 0.04 | 0.04 | 0.05 | 0.06 | 0.07 | 0.09 | 0.12 | 0.17 | 0.24 |
| Laser | 0 | 0 | 0 | 0 | 0 | 0.01 | 0.02 | 0.03 | 0.04 | 0.06 | 0.08 | 0.11 | 0.14 | 0.15 | 0.15 | 0.11 | 0.07 | 0.03 |
| N-acetylcysteine | 0.02 | 0.06 | 0.05 | 0.05 | 0.05 | 0.05 | 0.05 | 0.05 | 0.05 | 0.06 | 0.05 | 0.06 | 0.05 | 0.06 | 0.06 | 0.07 | 0.08 | 0.09 |
| Penicillin | 0 | 0.01 | 0.02 | 0.02 | 0.03 | 0.03 | 0.04 | 0.04 | 0.04 | 0.05 | 0.06 | 0.07 | 0.08 | 0.08 | 0.09 | 0.1 | 0.12 | 0.11 |
| Placebo | 0.02 | 0.2 | 0.28 | 0.23 | 0.14 | 0.08 | 0.03 | 0.02 | 0.01 | 0 | 0 | 0 | 0 | 0 | 0 | 0 | 0 | 0 |
| Probiotics | 0 | 0.01 | 0.02 | 0.01 | 0.02 | 0.02 | 0.03 | 0.03 | 0.04 | 0.04 | 0.05 | 0.05 | 0.06 | 0.06 | 0.08 | 0.1 | 0.14 | 0.23 |
| Prostaglandin E2 | 0.05 | 0.22 | 0.12 | 0.09 | 0.08 | 0.07 | 0.06 | 0.05 | 0.05 | 0.04 | 0.04 | 0.03 | 0.02 | 0.02 | 0.02 | 0.02 | 0.01 | 0.01 |
| Silver nitrate | 0 | 0.01 | 0.01 | 0.02 | 0.03 | 0.04 | 0.05 | 0.07 | 0.08 | 0.09 | 0.09 | 0.11 | 0.11 | 0.1 | 0.08 | 0.06 | 0.04 | 0.02 |
| Sucralfate | 0.01 | 0.05 | 0.04 | 0.04 | 0.04 | 0.04 | 0.04 | 0.05 | 0.05 | 0.05 | 0.06 | 0.05 | 0.06 | 0.06 | 0.07 | 0.07 | 0.1 | 0.12 |
| Triamcinolone | 0 | 0.01 | 0.04 | 0.08 | 0.12 | 0.14 | 0.15 | 0.13 | 0.11 | 0.08 | 0.06 | 0.03 | 0.02 | 0.01 | 0 | 0 | 0 | 0 |
| Zinc | 0.81 | 0.05 | 0.02 | 0.02 | 0.01 | 0.01 | 0.01 | 0.01 | 0.01 | 0.01 | 0.01 | 0.01 | 0 | 0 | 0.01 | 0 | 0.01 | 0.01 |

## Consistency check

| Parameter | Median (95% CI) |
|---|---|
| Random Effects Standard Deviation | 1.66 (1.01, 2.96) |

## Convergence Diagnostics

Convergence is assessed using the Brooks-Gelman-Rubin method. This method compares within-chain and between-chain variance to calculate the Potential Scale Reduction Factor (PSRF). A PSRF close to one indicates approximate convergence has been reached.

| Parameter | PSRF |
|---|---|
| d.Aloe.Chitosan | 1.00 |
| d.Chlorhexidine.Nacetylcysteine | 1.00 |
| d.Chlorhexidine.Sucralfate | 1.00 |
| d.Placebo.Aloe | 1.00 |
| d.Placebo.Chlorhexidine | 1.00 |
| d.Placebo.Doxycycline | 1.00 |
| d.Placebo.Glycyrrhiza | 1.00 |
| d.Placebo.Honey | 1.00 |
| d.Placebo.Insulinliposomalgel | 1.00 |
| d.Placebo.Laser | 1.00 |
| d.Placebo.Penicillin | 1.00 |
| d.Placebo.ProstaglandinE2 | 1.00 |
| d,Placebo.Silvernitrate | 1.00 |
| d.Placebo.Triamcinolone | 1.00 |
| d.Placebo.Zinc | 1.00 |
| d.Triamcinolone.Curcumin | 1.00 |
| d.Triamcinolone.Probiotics | 1.00 |
| sd.d | 1.00 |

| | |
|---|---|
| Number of chains | : 4 |
| Tuning iterations | : 20,000 |
| Simulation iterations | : 50,000 |
| Thinning interval | : 10 |
| Inference samples | : 10,000 |
| Variance scaling factor | : 2.5 |

## 2.Inconsistency Model

# Summary estimates for Healing effect

The estimate values for healing effect are given as mean difference (MD) and 95% confidence interval (CI). The table shows the pooled estimates based on the network meta-analysis during the treatment period for each study.

| | | | | | | | | | |
|---|---|---|---|---|---|---|---|---|---|
| Aloe | 1.01 (-2.48, 4.58) | -0.16 (-5.29, 4.88) | 0.62 (-4.33, 5.44) | 0.13 (-5.71, 5.88) | -0.04 (-5.19, 4.80) | -1.80 (-6.56, 2.80) | -2.21 (-8.00, 3.27) | -1.49 (-6.10, 3.13) | -0.63 (-7.18, 5.97) |
| -1.01 (-4.58, 2.48) | Chitosan | -1.17 (-6.08, 3.81) | -0.44 (-4.95, 3.95) | -0.88 (-6.57, 4.47) | -1.12 (-5.94, 3.65) | -2.82 (-7.32, 1.45) | -3.25 (-8.70, 2.17) | -2.54 (-6.76, 1.81) | -1.66 (-8.04, 4.76) |
| 0.16 (-4.88, 5.29) | 1.17 (-3.81, 6.08) | Chlorhexidine | 0.74 (-3.96, 5.56) | 0.25 (-4.67, 5.10) | 0.04 (-3.99, 4.10) | -1.68 (-5.60, 2.27) | -2.11 (-6.95, 2.89) | -1.37 (-4.94, 2.37) | -0.50 (-4.47, 3.64) |
| -0.62 (-5.44, 4.33) | 0.44 (-3.95, 4.95) | -0.74 (-5.56, 3.96) | Curcumin | -0.48 (-6.01, 4.91) | -0.70 (-5.18, 3.99) | -2.39 (-6.55, 1.70) | -2.83 (-8.25, 2.57) | -2.09 (-6.01, 1.88) | -1.21 (-7.62, 4.97) |
| -0.13 (-5.88, 5.71) | 0.88 (-4.47, 6.57) | -0.25 (-5.10, 4.67) | 0.48 (-4.91, 6.01) | Doxycycline | -0.19 (-4.95, 4.73) | -1.94 (-6.58, 2.79) | -2.35 (-7.71, 3.25) | -1.63 (-5.91, 2.97) | -0.75 (-7.05, 5.68) |
| 0.04 (-4.80, 5.19) | 1.12 (-3.65, 5.94) | -0.04 (-4.10, 3.99) | 0.70 (-3.99, 5.18) | 0.19 (-4.73, 4.95) | Glycyrrhiza | -1.73 (-5.51, 2.04) | -2.18 (-6.81, 2.58) | -1.42 (-4.84, 2.01) | -0.52 (-6.24, 5.17) |
| 1.80 (-2.80, 6.56) | 2.82 (-1.45, 7.32) | 1.68 (-2.27, 5.60) | 2.39 (-1.70, 6.55) | 1.94 (-2.79, 6.58) | 1.73 (-2.04, 5.51) | Honey | -0.42 (-5.11, 4.27) | 0.32 (-2.74, 3.59) | 1.24 (-4.52, 6.85) |
| 2.21 (-3.27, 8.00) | 3.25 (-2.17, 8.70) | 2.11 (-2.89, 6.95) | 2.83 (-2.57, 8.25) | 2.35 (-3.25, 7.71) | 2.18 (-2.58, 6.81) | 0.42 (-4.27, 5.11) | Insulin-liposomal gel | 0.73 (-3.55, 5.11) | 1.61 (-4.74, 8.01) |
| 1.49 (-3.13, 6.10) | 2.54 (-1.81, 6.76) | 1.37 (-2.37, 4.94) | 2.09 (-1.88, 6.01) | 1.63 (-2.97, 5.91) | 1.42 (-2.01, 4.84) | -0.32 (-3.59, 2.74) | -0.73 (-5.11, 3.55) | Laser | 0.89 (-4.64, 6.24) |
| 0.63 (-5.97, 7.18) | 1.66 (-4.76, 8.04) | 0.50 (-3.64, 4.47) | 1.21 (-4.97, 7.62) | 0.75 (-5.68, 7.05) | 0.52 (-5.17, 6.24) | -1.24 (-6.85, 4.52) | -1.61 (-8.01, 4.74) | -0.89 (-6.24, 4.64) | N-acetylcysteine |
| 1.44 (-4.20, 7.28) | 2.49 (-2.97, 8.09) | 1.33 (-3.47, 6.25) | 2.07 (-3.35, 7.47) | 1.61 (-3.91, 7.06) | 1.40 (-3.32, 6.20) | -0.34 (-4.97, 4.22) | -0.79 (-6.21, 4.75) | -0.04 (-4.22, 4.43) | 0.84 (-5.51, 7.12) |
| -2.05 (-5.66, 1.47) | -0.64 (-4.50, 3.29) | -1.80 (-4.70, 1.17) | -1.06 (-4.74, 2.72) | -1.54 (-5.48, 2.33) | -1.74 (-4.54, 1.09) | -3.48 (-6.00, -0.85) | -3.90 (-7.65, -0.07) | -3.16 (-5.05, -1.09) | -2.25 (-7.30, 2.73) |
| 1.87 (-3.96, 7.77) | 2.93 (-2.49, 8.33) | 1.76 (-3.74, 7.53) | 2.49 (-2.33, 7.47) | 2.02 (-4.25, 8.24) | 1.80 (-3.61, 7.68) | 0.09 (-4.95, 5.30) | -0.35 (-6.58, 5.89) | 0.39 (-4.51, 5.67) | 1.26 (-5.59, 8.35) |
| -1.18 (-6.94, 4.79) | -0.14 (-5.85, 5.61) | -1.28 (-6.38, 3.74) | -0.58 (-6.08, 4.86) | -1.05 (-6.71, 4.56) | -1.23 (-6.14, 3.76) | -2.97 (-7.87, 1.87) | -3.43 (-9.00, 2.24) | -2.67 (-7.17, 2.01) | -1.81 (-8.29, 4.73) |
| 0.90 (-4.07, 6.28) | 1.94 (-2.94, 6.95) | 0.81 (-3.41, 5.02) | 1.52 (-3.14, 6.34) | 1.03 (-4.03, 5.99) | 0.85 (-3.37, 5.01) | -1.03 (-4.27, 2.26) | -1.32 (-6.18, 3.72) | -0.58 (-4.15, 3.15) | 0.30 (-5.63, 6.20) |
| 0.97 (-5.58, 7.65) | 2.01 (-4.55, 8.46) | 0.83 (-3.37, 5.04) | 1.60 (-4.93, 7.75) | 1.09 (-5.48, 7.55) | 0.87 (-4.97, 6.76) | -0.85 (-6.70, 4.87) | -1.28 (-7.87, 5.15) | -0.55 (-6.17, 5.07) | 0.34 (-5.51, 6.14) |
| -0.47 (-4.47, 3.66) | 0.56 (-2.90, 4.01) | -0.60 (-4.46, 3.35) | 0.15 (-2.63, 2.89) | -0.34 (-5.14, 4.24) | -0.53 (-4.21, 3.22) | -2.26 (-5.35, 0.76) | -2.69 (-7.26, 1.88) | -1.66 (-4.45, 1.36) | -1.08 (-6.78, 4.59) |
| -6.82 (-16.07, 2.44) | -5.79 (-14.87, 3.19) | -6.95 (-15.84, 1.71) | -6.22 (-15.20, 2.77) | -6.70 (-15.91, 2.23) | -6.87 (-15.65, 1.76) | -8.62 (-17.39, -0.01) | -9.10 (-18.19, -0.05) | -8.31 (-16.94, 0.06) | -7.44 (-17.34, 2.22) |

| | | | | | | | |
|---|---|---|---|---|---|---|---|
| -1.44 (-7.28, 4.20) | 2.05 (-1.47, 5.66) | -1.87 (-7.77, 3.96) | 1.18 (-4.79, 6.94) | -0.90 (-6.28, 4.07) | -0.97 (-7.65, 5.58) | 0.47 (-3.66, 4.47) | 6.82 (-2.44, 16.07) |
| -2.49 (-8.09, 2.97) | 0.64 (-3.29, 4.50) | -2.93 (-8.33, 2.49) | 0.14 (-5.61, 5.85) | -1.94 (-6.95, 2.94) | -2.01 (-8.46, 4.55) | -0.56 (-4.01, 2.90) | 5.79 (-3.19, 14.87) |
| -1.33 (-6.25, 3.47) | 1.80 (-1.17, 4.70) | -1.76 (-7.53, 3.74) | 1.28 (-3.74, 6.38) | -0.81 (-5.02, 3.41) | -0.83 (-5.04, 3.37) | 0.60 (-3.35, 4.46) | 6.95 (-1.71, 15.84) |
| -2.07 (-7.47, 3.35) | 1.06 (-2.72, 4.74) | -2.49 (-7.47, 2.33) | 0.58 (-4.86, 6.08) | -1.52 (-6.34, 3.14) | -1.60 (-7.75, 4.93) | -0.15 (-2.89, 2.63) | 6.22 (-2.77, 15.20) |
| -1.61 (-7.06, 3.91) | 1.54 (-2.33, 5.48) | -2.02 (-8.24, 4.25) | 1.05 (-4.56, 6.71) | -1.03 (-5.99, 4.03) | -1.09 (-7.55, 5.48) | 0.34 (-4.24, 5.14) | 6.70 (-2.23, 15.91) |
| -1.40 (-6.20, 3.32) | 1.74 (-1.09, 4.54) | -1.80 (-7.68, 3.61) | 1.23 (-3.76, 6.14) | -0.85 (-5.01, 3.37) | -0.87 (-6.76, 4.97) | 0.53 (-3.22, 4.21) | 6.87 (-1.76, 15.65) |
| 0.34 (-4.22, 4.97) | 3.48 (0.85, 6.00) | -0.09 (-5.30, 4.95) | 2.97 (-1.87, 7.87) | 1.03 (-2.26, 4.27) | 0.85 (-4.87, 6.70) | 2.26 (-0.76, 5.35) | 8.62 (0.01, 17.39) |
| 0.79 (-4.75, 6.21) | 3.90 (0.07, 7.65) | 0.35 (-5.89, 6.58) | 3.43 (-2.24, 9.00) | 1.32 (-3.72, 6.18) | 1.28 (-5.15, 7.87) | 2.69 (-1.88, 7.26) | 9.10 (0.05, 18.19) |
| 0.04 (-4.43, 4.22) | 3.16 (1.09, 5.05) | -0.39 (-5.67, 4.51) | 2.67 (-2.01, 7.17) | 0.58 (-3.15, 4.15) | 0.55 (-5.07, 6.17) | 1.66 (-1.36, 4.45) | 8.31 (-0.06, 16.94) |
| -0.84 (-7.12, 5.51) | 2.25 (-2.73, 7.30) | -1.26 (-8.35, 5.59) | 1.81 (-4.73, 8.29) | -0.30 (-6.20, 5.63) | -0.34 (-6.14, 5.51) | 1.08 (-4.59, 6.78) | 7.44 (-2.22, 17.34) |
| Penicillin | 3.13 (-0.73, 7.08) | -0.43 (-6.74, 5.76) | 2.62 (-3.02, 8.24) | 0.53 (-4.36, 5.56) | 0.51 (-5.92, 7.07) | 1.94 (-2.73, 6.65) | 8.32 (-0.74, 17.43) |
| -3.13 (-7.08, 0.73) | Placebo | -3.55 (-8.51, 1.21) | -0.51 (-4.55, 3.63) | -2.59 (-5.64, 0.48) | -2.62 (-7.70, 2.69) | -1.07 (-3.76, 1.93) | 5.13 (-2.97, 13.59) |
| 0.43 (-5.76, 6.74) | 3.55 (-1.21, 8.51) | Probiotics | 3.09 (-3.28, 9.52) | 0.96 (-4.51, 6.63) | 0.94 (-6.02, 8.11) | 2.35 (-1.73, 6.49) | 8.72 (-0.63, 18.29) |
| -2.62 (-8.24, 3.02) | 0.51 (-3.63, 4.55) | -3.09 (-9.52, 3.28) | Prostaglandin E2 | -2.11 (-7.19, 3.13) | -2.12 (-8.71, 4.56) | -0.69 (-5.45, 3.98) | 5.59 (-3.31, 15.02) |
| -0.53 (-5.56, 4.36) | 2.59 (-0.48, 5.64) | -0.96 (-6.63, 4.51) | 2.11 (-3.13, 7.19) | Silver nitrate | -0.02 (-6.06, 5.97) | 1.38 (-2.49, 5.29) | 7.75 (-0.99, 16.60) |
| -0.51 (-7.07, 5.92) | 2.62 (-2.69, 7.70) | -0.94 (-8.11, 6.02) | 2.12 (-4.56, 8.71) | 0.02 (-5.97, 6.06) | Sucralfate | 1.46 (-4.55, 7.00) | 7.71 (-1.86, 17.68) |
| -1.94 (-6.65, 2.73) | 1.07 (-1.93, 3.76) | -2.35 (-6.49, 1.73) | 0.69 (-3.98, 5.45) | -1.38 (-5.29, 2.49) | -1.46 (-7.00, 4.55) | Triamcinolone | 6.33 (-2.30, 15.11) |
| -8.32 (-17.43, 0.74) | -5.13 (-13.59, 2.97) | -8.72 (-18.29, 0.63) | -5.59 (-15.02, 3.31) | -7.75 (-16.60, 0.99) | -7.71 (-17.68, 1.86) | -6.33 (-15.11, 2.30) | Zinc |

## Inconsistency Factors

| Cycle | Median (95% CI) |
|---|---|
| Aloe, Chitosan, Honey, Placebo, Triamcinolone | -0.13 (-4.40, 2.70) |
| Honey, Laser, Placebo, Triamcinolone | -0.12 (-3.53, 2.49) |
| Honey, Placebo, Silver nitrate | -0.05 (-3.19, 2.66) |
| Honey, Placebo, Triamcinolone | 0.07 (-2.51, 3.12) |

## Variance Calculation

| Parameter | Median (95% CI) |
|---|---|
| Random Effects Standard Deviation | 1.74 (1.04, 3.19) |
| Inconsistency Standard Deviation | 1.25 (0.06, 4.94) |

## Convergence Diagnostics

Convergence is assessed using the Brooks-Gelman-Rubin method. This method compares within-chain and between-chain variance to calculate the Potential Scale Reduction Factor (PSRF). A PSRF close to one indicates approximate convergence has been reached.

| Parameter | PSRF |
|---|---|
| d.Aloe.Chitosan | 1.00 |
| d.Chitosan.Triamcinolone | 1.00 |
| d.Chlorhexidine.Nacetylcysteine | 1.00 |
| d.Chlorhexidine.Sucralfate | 1.00 |
| d.Honey.Placebo | 1.00 |
| d.Placebo.Chlorhexidine | 1.00 |
| d.Placebo.Doxycycline | 1.00 |
| d.Placebo.Glycyrrhiza | 1.00 |
| d.Placebo.Insulinliposomalgel | 1.00 |
| d.Placebo.Laser | 1.00 |
| d.Placebo.Penicilin | 1.00 |
| d.Placebo.ProstaglandinE2 | 1.00 |
| d.Placebo.Silvernitrate | 1.00 |
| d.Placebo.Zinc | 1.00 |
| d.Triamcinolone.Curcumin | 1.00 |
| d.Triamcinolone.Honey | 1.00 |
| d.Triamcinolone.Probiotics | 1.00 |
| w.Aloe.Chitosan.Triamcinolone.Honey.Placebo | 1.00 |
| w.Honey.Placebo.Laser.Triamcinolone | 1.00 |
| w.Honey.Placebo.Sivernitrate | 1.00 |
| w.Honey.Placebo.Triamcinolone | 1.00 |
| sd.d | 1.00 |
| sd.w | 1.00 |

Number of chains : 4
Tuning iterations : 20,000
Simulation iterations : 50,000
Thinning interval : 10
Inference samples : 10,000
Variance scaling factor : 2.5

## 3. Node-splitting analysis

### Node-splitting analysis of inconsistency

Inconsistency between direct and indirect estimates was estimated in the Node-Splitting Model. When P values is above 0.05 in a comparison, there is evidence of statistical inconsistency.

| Name | Direct Effect | Indirect Effect | Overall | P-Value |
|---|---|---|---|---|
| Aloe, Chitosan | 0.53 (-3.52, 4.64) | 2.55 (-3.37, 8.80) | 1.15 (-2.06, 4.38) | 0.55 |
| Aloe, Placebo | 2.63 (-1.46, 6.74) | 0.61 (-5.66, 6.72) | 1.96 (-1.29, 5.26) | 0.56 |
| Chitosan, Triamcinolone | -1.12 (-5.09, 2.87) | 0.98 (-5.10, 7.03) | -0.44 (-3.60, 2.75) | 0.55 |
| Honey, Silver nitrate | 0.52 (-3.45, 4.52) | 0.64 (-4.23, 5.67) | 1.02 (-1.93, 3.92) | 0.96 |
| Honey, Triamcinolone | 3.17 (-0.67, 7.09) | 1.73 (-2.02, 5.56) | 2.25 (-0.47, 5.07) | 0.55 |
| Laser, Placebo | 3.28 (1.01, 5.26) | 2.20 (-2.39, 6.89) | 3.08 (1.19, 4.81) | 0.64 |
| Laser, Triamcinolone | 1.11 (-2.81, 4.98) | 2.26 (-1.19, 5.37) | 1.80 (-0.52, 4.03) | 0.62 |
| Placebo, Triamcinolone | -1.02 (-3.67, 1.63) | -2.41 (-6.00, 1.23) | -1.28 (-3.26, 0.77) | 0.49 |

## 4. Network structure

### Network structure of sensitivity analysis

26 RCTs involving 18 local interventions were included in the sensitivity analysis considering the healing effect.

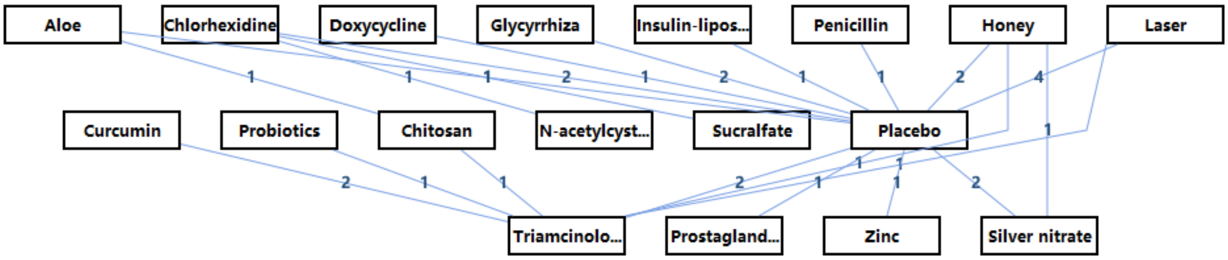

## 5. Pairwise meta-analysis

### Pairwise meta-analysis of healing effect

Healing effect were measured by mean difference (MD) and 95% confidence interval (CI).

| | Aloe | | | Chitosan | | | | Mean Difference |
|---|---|---|---|---|---|---|---|---|
| Study or Subgroup | Mean | SD | Total | Mean | SD | Total | Weight | IV, Random, 95% CI |
| Shi 2020 | 7.4 | 1.85 | 20 | 7.93 | 1.84 | 15 | 100.0% | -0.53 [-1.76, 0.70] |
| **Total (95% CI)** | | | **20** | | | **15** | **100.0%** | **-0.53 [-1.76, 0.70]** |

Heterogeneity: Not applicable
Test for overall effect: Z = 0.84 (P = 0.40)

Mean Difference
IV, Random, 95% CI

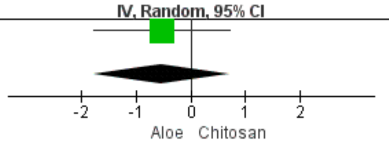


| | Aloe | | | Placebo | | | | Mean Difference |
|---|---|---|---|---|---|---|---|---|
| Study or Subgroup | Mean | SD | Total | Mean | SD | Total | Weight | IV, Random, 95% CI |
| Garnick 1998 | 6.38 | 0.73 | 3 | 9.01 | 1.32 | 4 | 100.0% | -2.63 [-4.16, -1.10] |
| **Total (95% CI)** | | | **3** | | | **4** | **100.0%** | **-2.63 [-4.16, -1.10]** |

Heterogeneity: Not applicable
Test for overall effect: Z = 3.36 (P = 0.0008)

Mean Difference
IV, Random, 95% CI

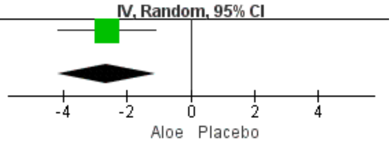


| | Chitosan | | | Triamcinolone | | | | Mean Difference |
|---|---|---|---|---|---|---|---|---|
| Study or Subgroup | Mean | SD | Total | Mean | SD | Total | Weight | IV, Random, 95% CI |
| Rahmani 2018 | 6.2 | 1.82 | 20 | 5.15 | 1.42 | 20 | 100.0% | 1.05 [0.04, 2.06] |
| **Total (95% CI)** | | | **20** | | | **20** | **100.0%** | **1.05 [0.04, 2.06]** |

Heterogeneity: Not applicable
Test for overall effect: Z = 2.03 (P = 0.04)

Mean Difference
IV, Random, 95% CI

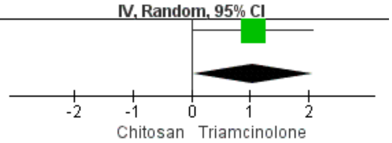


| | Chlorhexidine | | | N-acetylcysteine | | | | Mean Difference |
|---|---|---|---|---|---|---|---|---|
| Study or Subgroup | Mean | SD | Total | Mean | SD | Total | Weight | IV, Random, 95% CI |
| Halboub 2019 | 5.4 | 2.82 | 20 | 4.87 | 2.46 | 38 | 100.0% | 0.53 [-0.93, 1.99] |
| **Total (95% CI)** | | | **20** | | | **38** | **100.0%** | **0.53 [-0.93, 1.99]** |

Heterogeneity: Not applicable
Test for overall effect: Z = 0.71 (P = 0.48)

Mean Difference
IV, Random, 95% CI

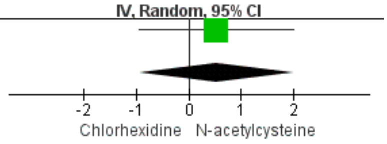


| | Chlorhexidine | | | Placebo | | | | Mean Difference |
|---|---|---|---|---|---|---|---|---|
| Study or Subgroup | Mean | SD | Total | Mean | SD | Total | Weight | IV, Random, 95% CI |
| Addy 1976 | 4.8 | 2.9 | 20 | 7.8 | 2.9 | 20 | 45.1% | -3.00 [-4.80, -1.20] |
| Hunter 1987 | 5.02 | 2.41 | 38 | 5.78 | 2.74 | 38 | 54.9% | -0.76 [-1.92, 0.40] |
| **Total (95% CI)** | | | **58** | | | **58** | **100.0%** | **-1.77 [-3.96, 0.41]** |

Heterogeneity: $Tau^2 = 1.91$; $Chi^2 = 4.21$, df = 1 (P = 0.04); $I^2 = 76\%$
Test for overall effect: Z = 1.59 (P = 0.11)

Mean Difference
IV, Random, 95% CI

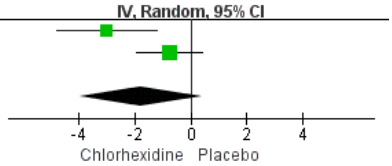


| | Chlorhexidine | | | Sucralfate | | | | Mean Difference |
|---|---|---|---|---|---|---|---|---|
| Study or Subgroup | Mean | SD | Total | Mean | SD | Total | Weight | IV, Random, 95% CI |
| Soylu and Okuyucu 2014 | 2.8 | 3 | 19 | 1.97 | 1.56 | 23 | 100.0% | 0.83 [-0.66, 2.32] |
| **Total (95% CI)** | | | **19** | | | **23** | **100.0%** | **0.83 [-0.66, 2.32]** |

Heterogeneity: Not applicable
Test for overall effect: Z = 1.09 (P = 0.28)

Mean Difference
IV, Random, 95% CI

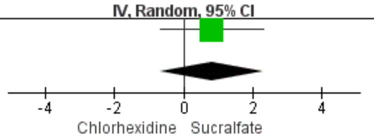


| | Curcumin | | | Triamcinolone | | | | Mean Difference |
|---|---|---|---|---|---|---|---|---|
| Study or Subgroup | Mean | SD | Total | Mean | SD | Total | Weight | IV, Random, 95% CI |
| Deshmukh 2014 | 3.9 | 1.18 | 30 | 4.1 | 1.18 | 30 | 38.7% | -0.20 [-0.80, 0.40] |
| Raman 2020 | 7.21 | 0.08 | 30 | 6.77 | 0.08 | 30 | 61.3% | 0.44 [0.40, 0.48] |
| **Total (95% CI)** | | | **60** | | | **60** | **100.0%** | **0.19 [-0.42, 0.80]** |

Heterogeneity: $Tau^2 = 0.16$; $Chi^2 = 4.39$, df = 1 (P = 0.04); $I^2 = 77\%$
Test for overall effect: Z = 0.62 (P = 0.54)

Mean Difference
IV, Random, 95% CI

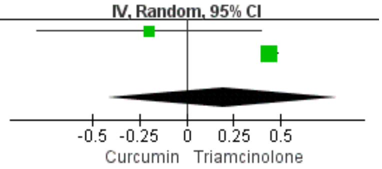


| | Doxycycline | | | Placebo | | | | Mean Difference |
|---|---|---|---|---|---|---|---|---|
| Study or Subgroup | Mean | SD | Total | Mean | SD | Total | Weight | IV, Random, 95% CI |
| Vijayabala 2013 | 3.7 | 1.3 | 25 | 5.3 | 1.2 | 25 | 100.0% | -1.60 [-2.29, -0.91] |
| **Total (95% CI)** | | | **25** | | | **25** | **100.0%** | **-1.60 [-2.29, -0.91]** |

Heterogeneity: Not applicable
Test for overall effect: Z = 4.52 (P < 0.00001)

Mean Difference
IV, Random, 95% CI

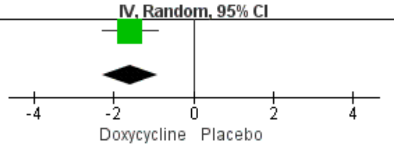

| Study or Subgroup | Honey Mean | Honey SD | Honey Total | Placebo Mean | Placebo SD | Placebo Total | Weight | Mean Difference IV, Random, 95% CI |
|---|---|---|---|---|---|---|---|---|
| El-Haddad 2014 | 2.73 | 0.57 | 67 | 7.14 | 0.92 | 56 | 50.3% | -4.41 [-4.69, -4.13] |
| Rodríguez-Archilla 2017 | 6.8 | 0.71 | 25 | 8.96 | 0.89 | 25 | 49.7% | -2.16 [-2.61, -1.71] |
| **Total (95% CI)** | | | **92** | | | **81** | **100.0%** | **-3.29 [-5.50, -1.09]** |

Heterogeneity: Tau² = 2.50; Chi² = 70.50, df = 1 (P < 0.00001); I² = 99%
Test for overall effect: Z = 2.93 (P = 0.003)

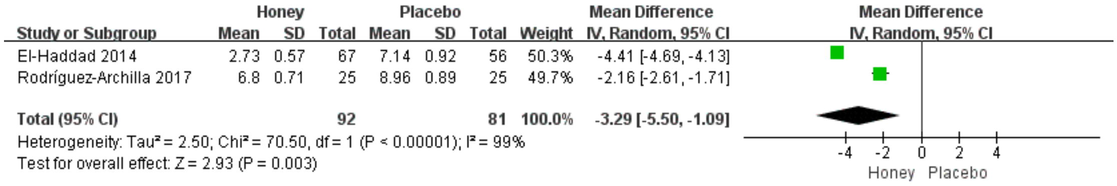


| Study or Subgroup | Honey Mean | Honey SD | Honey Total | Sliver nitrate Mean | Sliver nitrate SD | Sliver nitrate Total | Weight | Mean Difference IV, Random, 95% CI |
|---|---|---|---|---|---|---|---|---|
| Rodríguez-Archilla 2017 | 6.8 | 0.71 | 25 | 7.32 | 0.99 | 25 | 100.0% | -0.52 [-1.00, -0.04] |
| **Total (95% CI)** | | | **25** | | | **25** | **100.0%** | **-0.52 [-1.00, -0.04]** |

Heterogeneity: Not applicable
Test for overall effect: Z = 2.13 (P = 0.03)

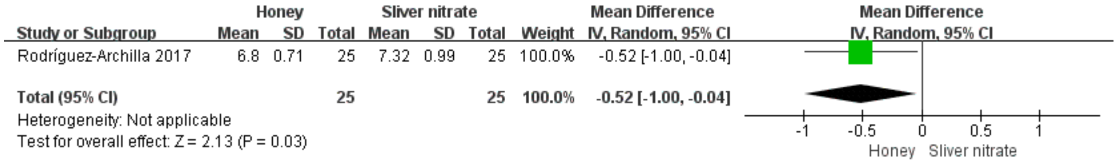


| Study or Subgroup | Honey Mean | Honey SD | Honey Total | Triamcinolone Mean | Triamcinolone SD | Triamcinolone Total | Weight | Mean Difference IV, Random, 95% CI |
|---|---|---|---|---|---|---|---|---|
| El-Haddad 2014 | 2.73 | 0.57 | 67 | 5.91 | 0.91 | 57 | 100.0% | -3.18 [-3.45, -2.91] |
| **Total (95% CI)** | | | **67** | | | **57** | **100.0%** | **-3.18 [-3.45, -2.91]** |

Heterogeneity: Not applicable
Test for overall effect: Z = 22.84 (P < 0.00001)

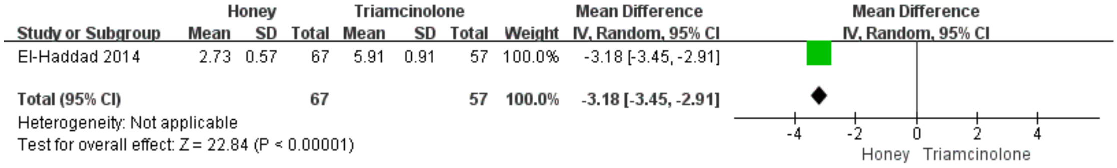


| Study or Subgroup | Insulin-liposomal gel Mean | Insulin-liposomal gel SD | Insulin-liposomal gel Total | Placebo Mean | Placebo SD | Placebo Total | Weight | Mean Difference IV, Random, 95% CI |
|---|---|---|---|---|---|---|---|---|
| El-Wakeel 2019 | 4.2 | 0.6 | 40 | 8.1 | 1 | 40 | 100.0% | -3.90 [-4.26, -3.54] |
| **Total (95% CI)** | | | **40** | | | **40** | **100.0%** | **-3.90 [-4.26, -3.54]** |

Heterogeneity: Not applicable
Test for overall effect: Z = 21.15 (P < 0.00001)

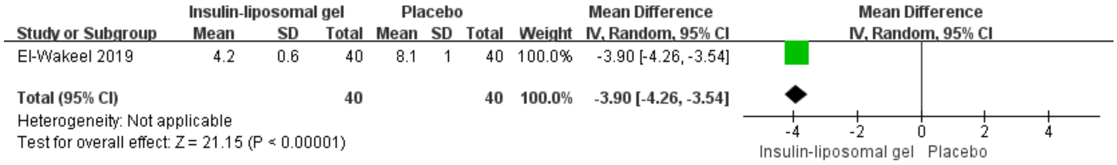


| Study or Subgroup | Laser Mean | Laser SD | Laser Total | Placebo Mean | Placebo SD | Placebo Total | Weight | Mean Difference IV, Random, 95% CI |
|---|---|---|---|---|---|---|---|---|
| Aggarwal 2014 | 3.05 | 1.1 | 30 | 8.9 | 2.45 | 30 | 28.2% | -5.85 [-6.81, -4.89] |
| Prasad 2013 | 4.08 | 0.81 | 25 | 7.84 | 0.9 | 25 | 29.8% | -3.76 [-4.23, -3.29] |
| Zand 2012 | 4.8 | 2.4 | 10 | 7.6 | 2.5 | 10 | 21.9% | -2.80 [-4.95, -0.65] |
| Zeini 2017 | 7.21 | 4.2 | 14 | 6.4 | 1.83 | 10 | 20.1% | 0.81 [-1.67, 3.29] |
| **Total (95% CI)** | | | **79** | | | **75** | **100.0%** | **-3.22 [-5.12, -1.32]** |

Heterogeneity: Tau² = 3.09; Chi² = 30.92, df = 3 (P < 0.00001); I² = 90%
Test for overall effect: Z = 3.33 (P = 0.0009)

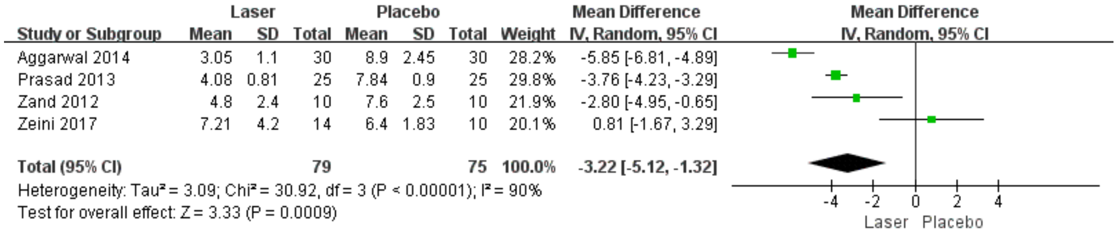


| Study or Subgroup | Laser Mean | Laser SD | Laser Total | Triamcinolone Mean | Triamcinolone SD | Triamcinolone Total | Weight | Mean Difference IV, Random, 95% CI |
|---|---|---|---|---|---|---|---|---|
| Huo 2021 | 6.6 | 0.29 | 25 | 7.77 | 0.52 | 26 | 100.0% | -1.17 [-1.40, -0.94] |
| **Total (95% CI)** | | | **25** | | | **26** | **100.0%** | **-1.17 [-1.40, -0.94]** |

Heterogeneity: Not applicable
Test for overall effect: Z = 9.97 (P < 0.00001)

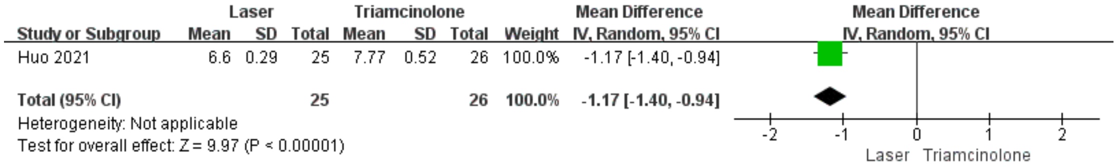


| Study or Subgroup | Penicillin Mean | Penicillin SD | Penicillin Total | Placebo Mean | Placebo SD | Placebo Total | Weight | Mean Difference IV, Random, 95% CI |
|---|---|---|---|---|---|---|---|---|
| Owlia 2020 | 2.79 | 0.16 | 14 | 5.9 | 0.93 | 12 | 100.0% | -3.11 [-3.64, -2.58] |
| **Total (95% CI)** | | | **14** | | | **12** | **100.0%** | **-3.11 [-3.64, -2.58]** |

Heterogeneity: Not applicable
Test for overall effect: Z = 11.44 (P < 0.00001)

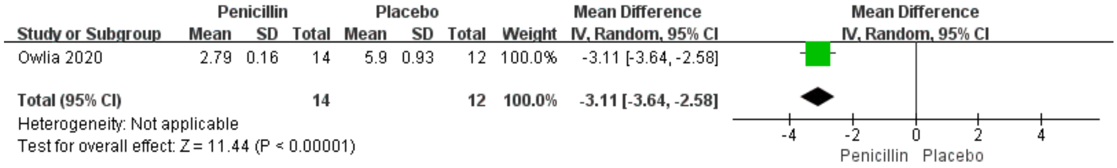


| Study or Subgroup | Probiotics Mean | Probiotics SD | Probiotics Total | Triamcinolone Mean | Triamcinolone SD | Triamcinolone Total | Weight | Mean Difference IV, Random, 95% CI |
|---|---|---|---|---|---|---|---|---|
| Ibrahim 2020 | 5.4 | 1.4 | 20 | 7.75 | 2.88 | 20 | 100.0% | -2.35 [-3.75, -0.95] |
| **Total (95% CI)** | | | **20** | | | **20** | **100.0%** | **-2.35 [-3.75, -0.95]** |

Heterogeneity: Not applicable
Test for overall effect: Z = 3.28 (P = 0.001)

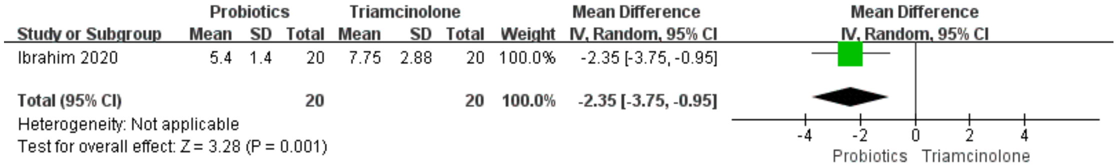

| | Prostaglandin E2 | | | Placebo | | | | Mean Difference |
|---|---|---|---|---|---|---|---|---|
| Study or Subgroup | Mean | SD | Total | Mean | SD | Total | Weight | IV, Random, 95% CI |
| Taylor 1993 | 7.78 | 2.44 | 18 | 8.27 | 1.87 | 15 | 100.0% | -0.49 [-1.96, 0.98] |
| **Total (95% CI)** | | | **18** | | | **15** | **100.0%** | **-0.49 [-1.96, 0.98]** |
| Heterogeneity: Not applicable | | | | | | | | |
| Test for overall effect: Z = 0.65 (P = 0.51) | | | | | | | | |

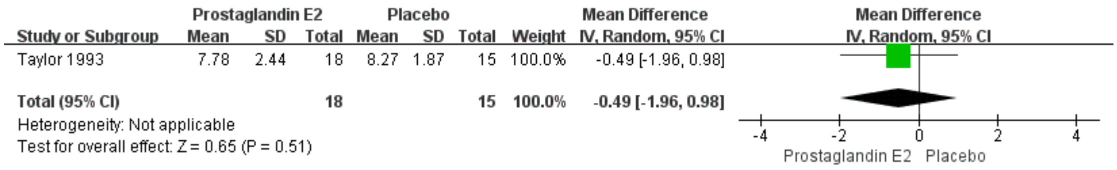


| | Silver nitrate | | | Placebo | | | | Mean Difference |
|---|---|---|---|---|---|---|---|---|
| Study or Subgroup | Mean | SD | Total | Mean | SD | Total | Weight | IV, Random, 95% CI |
| Rodríguez-Archilla 2017 | 7.32 | 0.99 | 25 | 8.96 | 0.89 | 25 | 55.8% | -1.64 [-2.16, -1.12] |
| Soylu 2014 | 2.7 | 1.48 | 35 | 5.5 | 2.22 | 30 | 44.2% | -2.80 [-3.73, -1.87] |
| **Total (95% CI)** | | | **60** | | | **55** | **100.0%** | **-2.15 [-3.28, -1.02]** |
| Heterogeneity: Tau² = 0.52; Chi² = 4.52, df = 1 (P = 0.03); I² = 78% | | | | | | | | |
| Test for overall effect: Z = 3.74 (P = 0.0002) | | | | | | | | |

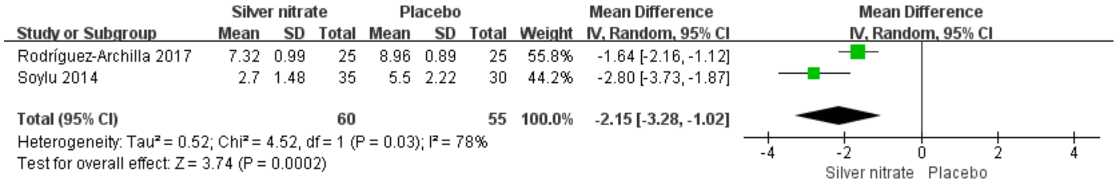


| | Triamcinolone | | | Placebo | | | | Mean Difference |
|---|---|---|---|---|---|---|---|---|
| Study or Subgroup | Mean | SD | Total | Mean | SD | Total | Weight | IV, Random, 95% CI |
| El-Haddad 2014 | 5.91 | 0.91 | 57 | 7.14 | 0.92 | 56 | 68.2% | -1.23 [-1.57, -0.89] |
| Tavangar 2019 | 5.75 | 1.08 | 20 | 6.5 | 1.05 | 20 | 31.8% | -0.75 [-1.41, -0.09] |
| **Total (95% CI)** | | | **77** | | | **76** | **100.0%** | **-1.08 [-1.52, -0.64]** |
| Heterogeneity: Tau² = 0.04; Chi² = 1.61, df = 1 (P = 0.20); I² = 38% | | | | | | | | |
| Test for overall effect: Z = 4.82 (P < 0.00001) | | | | | | | | |

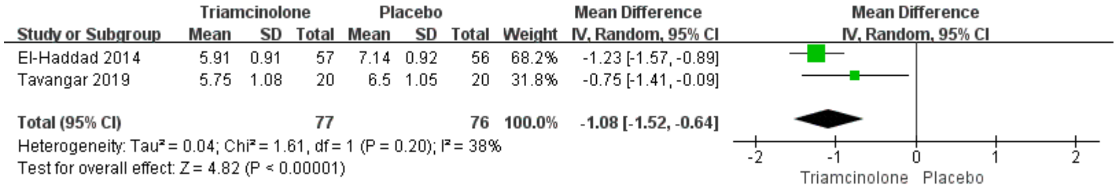


| | Zinc | | | Placebo | | | | Mean Difference |
|---|---|---|---|---|---|---|---|---|
| Study or Subgroup | Mean | SD | Total | Mean | SD | Total | Weight | IV, Random, 95% CI |
| Addy 1974 | 21.8 | 11.5 | 12 | 16.8 | 6.2 | 12 | 100.0% | 5.00 [-2.39, 12.39] |
| **Total (95% CI)** | | | **12** | | | **12** | **100.0%** | **5.00 [-2.39, 12.39]** |
| Heterogeneity: Not applicable | | | | | | | | |
| Test for overall effect: Z = 1.33 (P = 0.18) | | | | | | | | |

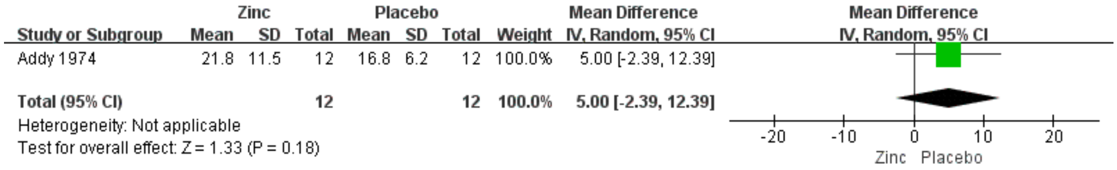

| Comparison | NO. of included study | Heterogeneity ($I^2$) | Combined effect [MD (95%CI)] |
|---|---|---|---|
| Aloe vs | | | |
| Placebo | 1 | NA | -2.63 (-4.16, -1.10) |
| Chitosan | 1 | NA | -0.53 (-1.76, 0.70) |
| Chitosan vs | | | |
| Triamcinolone | 1 | NA | 1.05 (0.04, 2.06) |
| Chlorhexidine vs | | | |
| Placebo | 2 | 76% | -1.77 (-3.96, 0.41) |
| Sucralfate | 1 | NA | 0.83 (-0.66, 2.32) |
| N-acetylcysteine | 1 | NA | 0.53 (-0.93, 1.99) |
| Curcumin vs | | | |
| Triamcinolone | 2 | 77% | 0.19 (-0.42, 0.80) |
| Doxycycline vs | | | |
| Placebo | 1 | NA | -1.60 (-2.29, -0.91) |
| Glycyrrhiza vs | | | |
| Placebo | 2 | 95% | -1.72 (-4.77, 1.32) |
| Honey vs | | | |
| Placebo | 2 | 99% | -3.29 (-5.50, -1.09) |
| Triamcinolone | 1 | NA | -3.18 (-3.45, -2.91) |
| Sliver nitrate | 1 | NA | -0.52 (-1.00, -0.04) |
| Insulin-liposomal gel vs | | | |
| Placebo | 1 | NA | -3.90 (-4.26, -3.54) |
| Laser vs | | | |
| Placebo | 4 | 90% | -3.22 (-5.12, -1.32) |
| Triamcinolone | 1 | NA | -1.17 (-1.40, -0.94) |
| Penicillin vs | | | |
| Placebo | 1 | NA | -3.11 (-3.64, -2.58) |
| Probiotics vs | | | |
| Triamcinolone | 1 | NA | -2.35 (-3.75, -0.95) |
| Prostaglandin E2 vs | | | |
| Placebo | 1 | NA | -0.49 (-1.96, 0.98) |
| Sliver nitrate vs | | | |
| Placebo | 2 | 78% | -2.15 (-3.28, -1.02) |
| Triamcinolone vs | | | |
| Placebo | 2 | 38% | -1.08 (-1.52, -0.64) |
| Zinc vs | | | |
| Placebo | 1 | NA | 5.00 (-2.39, 12.39) |

MD, mean difference; CI, confidence interval; NA, not applicable.

## Chapter S2 Size-reducing effect

## 1.Consistency Model

### Summary estimates for Size-reducing effect

The estimate values for size-reducing effect are given as mean difference (MD) and 95% confidence interval (CI). The table shows the pooled estimates based on the network meta-analysis during the treatment period for each study.

| | | | | | | | | | |
|---|---|---|---|---|---|---|---|---|---|
| Allicin | -0.03 (-57.26, 57.97) | 17.88 (-36.40, 72.91) | 7.17 (-65.84, 76.66) | -1.23 (-72.44, 68.54) | 15.67 (-75.07, 105.97) | -29.80 (-101.72, 43.30) | 9.18 (-47.48, 67.26) | 19.40 (-44.75, 82.51) | -1.83 (-72.19, 69.65) |
| 0.03 (-57.97, 57.26) | Aloe | 17.79 (-16.04, 50.64) | 6.70 (-50.76, 61.35) | -1.27 (-58.06, 54.29) | 15.01 (-62.84, 95.68) | -29.71 (-87.27, 29.15) | 9.32 (-28.39, 47.25) | 19.65 (-28.00, 65.65) | -1.21 (-58.77, 53.84) |
| -17.88 (-72.91, 36.40) | -17.79 (-50.64, 16.04) | Amlexanox | -11.00 (-64.44, 41.05) | -19.10 (-71.55, 33.01) | -2.77 (-79.47, 75.75) | -47.40 (-102.47, 9.24) | -8.85 (-41.13, 24.48) | 1.26 (-35.76, 38.69) | -19.26 (-72.62, 32.21) |
| -7.17 (-76.66, 65.84) | -6.70 (-61.35, 50.76) | 11.00 (-41.05, 64.44) | Benzydamine | -7.90 (-76.61, 63.20) | 8.49 (-80.50, 100.94) | -36.37 (-106.70, 36.92) | 2.65 (-52.23, 58.94) | 12.94 (-49.24, 75.73) | -8.24 (-77.25, 63.11) |
| 1.23 (-68.54, 72.44) | 1.27 (-54.29, 58.06) | 19.10 (-33.01, 71.55) | 7.90 (-63.20, 76.61) | Berberine gelatin | 16.72 (-70.83, 107.87) | -28.49 (-98.74, 44.52) | 10.67 (-45.76, 68.56) | 20.52 (-41.00, 82.58) | -0.45 (-69.16, 69.34) |
| -15.67 (-105.97, 75.07) | -15.01 (-95.68, 62.84) | 2.77 (-75.75, 79.47) | -8.49 (-100.94, 80.50) | -16.72 (-107.87, 70.83) | Chitosan | -45.29 (-135.67, 46.81) | -6.10 (-87.56, 73.60) | 4.17 (-80.39, 86.73) | -16.54 (-107.13, 72.53) |
| 29.80 (-43.30, 101.72) | 29.71 (-29.15, 87.27) | 47.40 (-9.24, 102.47) | 36.37 (-36.92, 106.70) | 28.49 (-44.52, 98.74) | 45.29 (-46.81, 135.67) | Cryotherapy | 38.75 (-19.39, 97.28) | 49.17 (-14.20, 109.45) | 28.28 (-44.82, 97.76) |
| -9.18 (-67.26, 47.48) | -9.32 (-47.25, 28.39) | 8.85 (-24.48, 41.13) | -2.65 (-58.94, 52.23) | -10.67 (-68.56, 45.76) | 6.10 (-73.60, 87.56) | -38.75 (-97.28, 19.39) | Curcumin | 10.05 (-37.03, 56.45) | -10.53 (-67.41, 44.51) |
| -19.40 (-82.51, 44.75) | -19.65 (-65.65, 28.00) | -1.26 (-38.69, 35.76) | -12.94 (-75.73, 49.24) | -20.52 (-82.58, 41.00) | -4.17 (-86.73, 80.39) | -49.17 (-109.45, 14.20) | -10.05 (-56.45, 37.03) | Dexamethasone | -21.23 (-81.51, 40.77) |
| 1.83 (-69.65, 72.19) | 1.21 (-53.84, 58.77) | 19.26 (-32.21, 72.62) | 8.24 (-63.11, 77.25) | 0.45 (-69.34, 69.16) | 16.54 (-72.53, 107.13) | -28.28 (-97.76, 44.82) | 10.53 (-44.51, 67.41) | 21.23 (-40.77, 81.51) | Diosmectite |
| -6.87 (-72.82, 61.31) | -7.41 (-57.15, 46.00) | 10.42 (-35.54, 59.21) | -0.60 (-52.36, 52.29) | -8.58 (-73.71, 58.65) | 7.99 (-76.67, 96.50) | -36.67 (-105.40, 34.67) | 1.79 (-46.73, 54.19) | 11.92 (-44.60, 70.89) | -8.70 (-74.11, 56.76) |
| -11.60 (-68.85, 45.73) | -12.06 (-47.94, 25.67) | 5.83 (-23.74, 37.06) | -5.04 (-61.23, 49.17) | -13.43 (-69.33, 41.77) | 3.40 (-75.63, 83.83) | -41.42 (-98.62, 18.48) | -2.65 (-39.55, 35.11) | 7.39 (-37.22, 52.40) | -13.49 (-68.04, 42.64) |
| -10.02 (-66.29, 51.38) | -10.43 (-46.21, 31.07) | 7.47 (-22.72, 41.98) | -3.58 (-57.20, 51.59) | -11.63 (-66.41, 48.12) | 5.04 (-74.15, 87.39) | -39.94 (-96.48, 22.29) | -1.30 (-36.36, 41.11) | 8.85 (-34.43, 58.31) | -11.87 (-66.00, 47.10) |
| 17.22 (-33.32, 67.83) | 17.23 (-9.23, 45.09) | 35.29 (15.53, 54.72) | 23.99 (-26.19, 73.07) | 16.12 (-32.96, 64.10) | 32.51 (-42.22, 107.67) | -12.25 (-63.50, 40.49) | 26.41 (-1.26, 54.91) | 36.62 (-1.39, 74.85) | 15.97 (-33.50, 63.58) |
| 2.25 (-56.96, 60.28) | 2.16 (-35.90, 39.89) | 20.01 (-13.33, 52.44) | 8.80 (-48.03, 63.31) | 0.68 (-55.89, 56.08) | 17.01 (-62.51, 99.12) | -27.49 (-84.85, 31.68) | 11.23 (-25.47, 47.35) | 21.62 (-25.03, 67.18) | 0.82 (-55.85, 55.23) |
| -21.38 (-109.18, 66.60) | -21.72 (-96.44, 57.46) | -3.90 (-75.60, 72.00) | -14.18 (-66.32, 36.53) | -22.48 (-107.41, 64.74) | -6.42 (-109.67, 101.42) | -51.12 (-139.16, 38.60) | -12.42 (-86.55, 65.13) | -1.97 (-83.33, 81.20) | -23.31 (-110.56, 65.25) |
| -8.47 (-63.29, 44.61) | -8.38 (-40.18, 21.59) | 9.29 (-16.84, 33.32) | -2.15 (-54.59, 47.05) | -9.71 (-63.45, 41.50) | 6.19 (-71.31, 85.16) | -38.42 (-92.95, 16.63) | 0.59 (-24.02, 24.70) | 10.77 (-31.50, 52.17) | -10.33 (-63.31, 41.22) |
| -5.64 (-71.49, 60.98) | -5.12 (-55.35, 45.43) | 12.50 (-33.97, 57.97) | 1.12 (-64.27, 65.66) | -6.54 (-73.34, 57.64) | 10.30 (-77.01, 97.23) | -34.93 (-103.23, 32.98) | 3.78 (-44.44, 53.68) | 13.82 (-42.48, 70.77) | -7.19 (-71.97, 58.25) |
| 5.73 (-67.81, 77.73) | 5.43 (-52.30, 64.10) | 23.68 (-32.84, 78.05) | 11.95 (-61.04, 84.36) | 4.75 (-68.54, 74.30) | 20.72 (-67.66, 111.97) | -24.90 (-99.17, 52.62) | 14.51 (-43.67, 72.64) | 25.16 (-42.26, 89.87) | 3.81 (-67.65, 75.79) |

| | | | | | | | | |
|---|---|---|---|---|---|---|---|---|
| 6.87 (-61.31, 72.82) | 11.60 (-45.73, 68.85) | 10.02 (-51.38, 66.29) | -17.22 (-67.83, 33.32) | -2.25 (-60.28, 56.96) | 21.38 (-66.60, 109.18) | 8.47 (-44.61, 63.29) | 5.64 (-60.98, 71.49) | -5.73 (-77.73, 67.81) |
| 7.41 (-46.00, 57.15) | 12.06 (-25.67, 47.94) | 10.43 (-31.07, 46.21) | -17.23 (-45.09, 9.23) | -2.16 (-39.89, 35.90) | 21.72 (-57.46, 96.44) | 8.38 (-21.59, 40.18) | 5.12 (-45.43, 55.35) | -5.43 (-64.10, 52.30) |
| -10.42 (-59.21, 35.54) | -5.83 (-37.06, 23.74) | -7.47 (-41.98, 22.72) | -35.29 (-54.72, -15.53) | -20.01 (-52.44, 13.33) | 3.90 (-72.00, 75.60) | -9.29 (-33.32, 16.84) | -12.50 (-57.97, 33.97) | -23.68 (-78.05, 32.84) |
| 0.60 (-52.29, 52.36) | 5.04 (-49.17, 61.23) | 3.58 (-51.59, 57.20) | -23.99 (-73.07, 26.19) | -8.80 (-63.31, 48.03) | 14.18 (-36.53, 66.32) | 2.15 (-47.05, 54.59) | -1.12 (-65.66, 64.27) | -11.95 (-84.36, 61.04) |
| 8.58 (-58.65, 73.71) | 13.43 (-41.77, 69.33) | 11.63 (-48.12, 66.41) | -16.12 (-64.10, 32.96) | -0.68 (-56.08, 55.89) | 22.48 (-64.74, 107.41) | 9.71 (-41.50, 63.45) | 6.54 (-57.64, 73.34) | -4.75 (-74.30, 68.54) |
| -7.99 (-96.50, 76.67) | -3.40 (-83.83, 75.63) | -5.04 (-87.39, 74.15) | -32.51 (-107.67, 42.22) | -17.01 (-99.12, 62.51) | 6.42 (-101.42, 109.67) | -6.19 (-85.16, 71.31) | -10.30 (-97.23, 77.01) | -20.72 (-111.97, 67.66) |
| 36.67 (-34.67, 105.40) | 41.42 (-18.48, 98.62) | 39.94 (-22.29, 96.48) | 12.25 (-40.49, 63.50) | 27.49 (-31.68, 84.85) | 51.12 (-38.60, 139.16) | 38.42 (-16.63, 92.95) | 34.93 (-32.98, 103.23) | 24.90 (-52.62, 99.17) |
| -1.79 (-54.19, 46.73) | 2.65 (-35.11, 39.55) | 1.30 (-41.11, 36.36) | -26.41 (-54.91, 1.26) | -11.23 (-47.35, 25.47) | 12.42 (-65.13, 86.55) | -0.59 (-24.70, 24.02) | -3.78 (-53.68, 44.44) | -14.51 (-72.64, 43.67) |
| -11.92 (-70.89, 44.60) | -7.39 (-52.40, 37.22) | -8.85 (-58.31, 34.43) | -36.62 (-74.85, 1.39) | -21.62 (-67.18, 25.03) | 1.97 (-81.20, 83.33) | -10.77 (-52.17, 31.50) | -13.82 (-70.77, 42.48) | -25.16 (-89.87, 42.26) |
| 8.70 (-56.76, 74.11) | 13.49 (-42.64, 68.04) | 11.87 (-47.10, 66.00) | -15.97 (-63.58, 33.50) | -0.82 (-55.23, 55.85) | 23.31 (-65.25, 110.56) | 10.33 (-41.22, 63.31) | 7.19 (-58.25, 71.97) | -3.81 (-75.79, 67.65) |
| Doxycycline | 4.57 (-45.18, 55.58) | 3.16 (-47.74, 51.14) | -24.55 (-67.34, 21.10) | -9.32 (-59.09, 41.92) | 13.83 (-59.86, 87.61) | 1.41 (-41.91, 47.89) | -2.31 (-60.63, 60.18) | -12.99 (-81.42, 58.31) |
| -4.57 (-55.58, 45.18) | Glycyrrhiza | -1.50 (-38.92, 30.74) | -29.07 (-54.49, -3.58) | -13.87 (-50.56, 23.48) | 9.81 (-67.43, 85.69) | -3.31 (-33.43, 28.03) | -6.61 (-55.50, 43.81) | -16.90 (-73.88, 40.25) |
| -3.16 (-51.14, 47.74) | 1.50 (-30.74, 38.92) | Laser | -27.74 (-53.44, 3.04) | -12.15 (-48.57, 28.98) | 11.28 (-63.83, 87.14) | -1.95 (-31.42, 34.44) | -4.86 (-52.98, 49.09) | -15.47 (-72.58, 45.54) |
| 24.55 (-21.10, 67.34) | 29.07 (3.58, 54.49) | 27.74 (-3.04, 53.44) | Placebo | 15.25 (-12.19, 43.58) | 39.02 (-34.93, 109.89) | 25.83 (7.91, 45.48) | 22.69 (-20.03, 66.50) | 11.96 (-39.02, 63.84) |
| 9.32 (-41.92, 59.09) | 13.87 (-23.48, 50.56) | 12.15 (-28.98, 48.57) | -15.25 (-43.58, 12.19) | Probiotics | 23.97 (-53.41, 98.41) | 10.59 (-16.93, 39.27) | 7.80 (-43.32, 57.67) | -2.98 (-62.39, 54.98) |
| -13.83 (-87.61, 59.86) | -9.81 (-85.69, 67.43) | -11.28 (-87.14, 63.83) | -39.02 (-109.89, 34.93) | -23.97 (-98.41, 53.41) | Quercetin | -13.34 (-83.61, 62.27) | -15.69 (-99.68, 67.09) | -26.73 (-114.36, 63.38) |
| -1.41 (-47.89, 41.91) | 3.31 (-28.03, 33.43) | 1.95 (-34.44, 31.42) | -25.83 (-45.48, -7.91) | -10.59 (-39.27, 16.93) | 13.34 (-62.27, 83.61) | Triamcinolone | -3.21 (-46.73, 40.25) | -14.20 (-69.92, 40.97) |
| 2.31 (-60.18, 60.63) | 6.61 (-43.81, 55.50) | 4.86 (-49.09, 52.98) | -22.69 (-66.50, 20.03) | -7.80 (-57.67, 43.32) | 15.69 (-67.09, 99.68) | 3.21 (-40.25, 46.73) | Triester Glycerol Oxide | -10.67 (-78.70, 56.04) |
| 12.99 (-58.31, 81.42) | 16.90 (-40.25, 73.88) | 15.47 (-45.54, 72.58) | -11.96 (-63.84, 39.02) | 2.98 (-54.98, 62.39) | 26.73 (-63.38, 114.36) | 14.20 (-40.97, 69.92) | 10.67 (-56.04, 78.70) | Zinc |

# Rank probability

**Rank 1 is best, rank N is worst.**

| Drug | Rank 1 | Rank 2 | Rank 3 | Rank 4 | Rank 5 | Rank 6 | Rank 7 | Rank 8 | Rank 9 | Rank 10 | Rank 11 | Rank 12 | Rank 13 | Rank 14 | Rank 15 | Rank 16 | Rank 17 | Rank 18 | Rank 19 |
|---|---|---|---|---|---|---|---|---|---|---|---|---|---|---|---|---|---|---|---|
| Allicin | 0.05 | 0.06 | 0.05 | 0.05 | 0.04 | 0.04 | 0.04 | 0.04 | 0.04 | 0.05 | 0.04 | 0.05 | 0.06 | 0.06 | 0.06 | 0.06 | 0.07 | 0.09 | 0.07 |
| Aloe | 0 | 0.01 | 0.02 | 0.03 | 0.03 | 0.04 | 0.05 | 0.06 | 0.07 | 0.08 | 0.09 | 0.09 | 0.1 | 0.1 | 0.09 | 0.07 | 0.04 | 0.03 | 0.01 |
| Amlexanox | 0.03 | 0.08 | 0.13 | 0.14 | 0.13 | 0.12 | 0.1 | 0.08 | 0.06 | 0.04 | 0.03 | 0.02 | 0.01 | 0.01 | 0 | 0 | 0 | 0 | 0 |
| Benzydamine | 0.02 | 0.09 | 0.08 | 0.06 | 0.05 | 0.05 | 0.05 | 0.05 | 0.05 | 0.05 | 0.05 | 0.05 | 0.05 | 0.06 | 0.05 | 0.05 | 0.05 | 0.05 | 0.03 |
| Berberine gelatin | 0.04 | 0.05 | 0.04 | 0.05 | 0.04 | 0.04 | 0.04 | 0.04 | 0.04 | 0.05 | 0.05 | 0.06 | 0.06 | 0.07 | 0.06 | 0.06 | 0.07 | 0.09 | 0.07 |
| Chitosan | 0.24 | 0.1 | 0.06 | 0.05 | 0.04 | 0.03 | 0.03 | 0.03 | 0.03 | 0.03 | 0.03 | 0.03 | 0.04 | 0.03 | 0.04 | 0.04 | 0.04 | 0.06 | 0.07 |
| Cryotherapy | 0 | 0.01 | 0.01 | 0.01 | 0.01 | 0.01 | 0.01 | 0.01 | 0.01 | 0.02 | 0.02 | 0.03 | 0.03 | 0.04 | 0.05 | 0.06 | 0.08 | 0.16 | 0.43 |
| Curcumin | 0.02 | 0.04 | 0.06 | 0.07 | 0.08 | 0.08 | 0.08 | 0.08 | 0.09 | 0.08 | 0.08 | 0.07 | 0.06 | 0.05 | 0.03 | 0.02 | 0.01 | 0.01 | 0 |
| Dexamethasone | 0.12 | 0.14 | 0.11 | 0.09 | 0.08 | 0.07 | 0.06 | 0.06 | 0.05 | 0.04 | 0.04 | 0.04 | 0.03 | 0.03 | 0.02 | 0.02 | 0.01 | 0.01 | 0 |
| Diosmectite | 0.04 | 0.05 | 0.04 | 0.04 | 0.04 | 0.04 | 0.04 | 0.04 | 0.05 | 0.05 | 0.05 | 0.06 | 0.05 | 0.06 | 0.07 | 0.06 | 0.07 | 0.09 | 0.07 |
| Doxycycline | 0.04 | 0.06 | 0.07 | 0.06 | 0.06 | 0.06 | 0.05 | 0.05 | 0.06 | 0.06 | 0.06 | 0.06 | 0.06 | 0.06 | 0.05 | 0.05 | 0.04 | 0.04 | 0.02 |
| Glycyrrhiza | 0.02 | 0.05 | 0.07 | 0.08 | 0.09 | 0.09 | 0.09 | 0.09 | 0.08 | 0.08 | 0.07 | 0.06 | 0.05 | 0.03 | 0.03 | 0.01 | 0.01 | 0 | 0 |
| Laser | 0.02 | 0.05 | 0.06 | 0.08 | 0.08 | 0.08 | 0.08 | 0.08 | 0.08 | 0.07 | 0.07 | 0.06 | 0.06 | 0.04 | 0.03 | 0.02 | 0.01 | 0.01 | 0 |
| Placebo | 0 | 0 | 0 | 0 | 0 | 0 | 0 | 0 | 0 | 0 | 0 | 0.01 | 0.04 | 0.08 | 0.17 | 0.25 | 0.26 | 0.15 | 0.03 |
| Probiotics | 0 | 0.01 | 0.01 | 0.02 | 0.03 | 0.03 | 0.04 | 0.05 | 0.06 | 0.07 | 0.09 | 0.1 | 0.1 | 0.11 | 0.09 | 0.08 | 0.06 | 0.03 | 0.01 |
| Quercetin | 0.27 | 0.12 | 0.07 | 0.05 | 0.04 | 0.04 | 0.03 | 0.03 | 0.03 | 0.03 | 0.03 | 0.03 | 0.03 | 0.03 | 0.03 | 0.03 | 0.03 | 0.04 | 0.04 |
| Triamcinolone | 0 | 0.01 | 0.02 | 0.04 | 0.07 | 0.1 | 0.11 | 0.13 | 0.13 | 0.13 | 0.1 | 0.08 | 0.05 | 0.03 | 0.01 | 0 | 0 | 0 | 0 |
| Triester Glycerol Oxide | 0.05 | 0.06 | 0.06 | 0.05 | 0.06 | 0.05 | 0.05 | 0.05 | 0.05 | 0.06 | 0.06 | 0.06 | 0.06 | 0.06 | 0.05 | 0.05 | 0.05 | 0.05 | 0.03 |
| Zinc | 0.04 | 0.04 | 0.04 | 0.03 | 0.03 | 0.03 | 0.03 | 0.03 | 0.03 | 0.04 | 0.04 | 0.05 | 0.05 | 0.07 | 0.07 | 0.06 | 0.08 | 0.11 | 0.11 |

## Consistency check

| Parameter | Median (95% CI) |
|---|---|
| Random Effects Standard Deviation | 23.49 (16.60, 34.97) |

## Convergence Diagnostics

Convergence is assessed using the Brooks-Gelman-Rubin method. This method compares within-chain and between-chain variance to calculate the Potential Scale Reduction Factor (PSRF). A PSRF close to one indicates approximate convergence has been reached.

| Parameter | PSRF |
|---|---|
| d.Benzydamine.Doxycycline | 1.01 |
| d.Benzydamine.Placebo | 1.06 |
| d.Benzydamine.Quercetin | 1.01 |
| d.Benzydamine.Triamcinolone | 1.08 |
| d.Placebo.Allicin | 1.00 |
| d.Placebo.Aloe | 1.01 |
| d.Placebo.Amlexanox | 1.01 |
| d.Placebo.Berberinegelatin | 1.01 |
| d.Placebo.Chitosan | 1.00 |
| d.Placebo.Cryotherapy | 1.10 |
| d.Placebo.Curcumin | 1.02 |
| d.Placebo.Dexamethasone | 1.04 |
| d.Placebo.Diosmectite | 1.00 |
| d.Placebo.Glycyrrhiza | 1.01 |
| d.Placebo.Laser | 1.33 |
| d.Placebo.Probiotics | 1.01 |
| d.Placebo.TriesterGlycerolOxide | 1.00 |
| d.Placebo.Zinc | 1.14 |
| sd.d | 1.24 |

Number of chains : 4
Tuning iterations : 20,000
Simulation iterations : 50,000
Thinning interval : 10
Inference samples : 10,000
Variance scaling factor : 2.5

## 2.Inconsistency Model

### Summary estimates for Size-reducing effect

The estimate values for size-reducing effect are given as mean difference (MD) and 95% confidence interval (CI). The table shows the pooled estimates based on the network meta-analysis during the treatment period for each study.

| | | | | | | | | | |
|---|---|---|---|---|---|---|---|---|---|
| Allicin | -0.72 (-53.32, 52.17) | 13.97 (-38.57, 67.58) | -4.00 (-82.92, 68.12) | -1.49 (-66.21, 64.92) | 13.99 (-73.57, 102.29) | -16.49 (-86.54, 53.17) | 10.11 (-46.69, 65.05) | 21.81 (-45.81, 85.28) | -2.17 (-66.45, 63.22) |
| 0.72 (-52.17, 53.32) | Aloe | 14.75 (-19.36, 49.47) | -2.22 (-68.76, 56.12) | -0.62 (-51.40, 49.27) | 15.17 (-62.27, 92.42) | -15.41 (-72.96, 40.51) | 10.90 (-25.82, 46.30) | 22.32 (-30.67, 73.20) | -0.97 (-51.84, 50.41) |
| -13.97 (-67.58, 38.57) | -14.75 (-49.47, 19.36) | Amlexanox | -16.29 (-85.45, 41.75) | -15.33 (-67.01, 34.12) | 0.20 (-79.63, 76.88) | -30.45 (-89.12, 25.55) | -4.27 (-40.04, 32.93) | 8.37 (-39.60, 50.89) | -15.77 (-67.22, 35.56) |
| 4.00 (-68.12, 82.92) | 2.22 (-56.12, 68.76) | 16.29 (-41.75, 85.45) | Benzydamine | 1.53 (-68.55, 79.55) | 18.53 (-75.86, 114.12) | -12.86 (-90.59, 68.23) | 13.25 (-42.68, 76.36) | 25.60 (-37.75, 89.40) | 2.32 (-70.73, 78.55) |
| 1.49 (-64.92, 66.21) | 0.62 (-49.27, 51.40) | 15.33 (-34.12, 67.01) | -1.53 (-79.55, 68.55) | Berberine gelatin | 15.70 (-72.21, 103.11) | -15.09 (-83.95, 52.93) | 11.09 (-42.90, 64.83) | 22.75 (-41.25, 84.49) | -0.63 (-61.79, 63.22) |
| -13.99 (-102.29, 73.57) | -15.17 (-92.42, 62.27) | -0.20 (-76.88, 79.63) | -18.53 (-114.12, 75.86) | -15.70 (-103.11, 72.21) | Chitosan | -30.59 (-123.11, 60.34) | -4.21 (-84.42, 76.10) | 7.25 (-79.95, 94.01) | -15.89 (-101.03, 69.84) |
| 16.49 (-53.17, 86.54) | 15.41 (-40.51, 72.96) | 30.45 (-25.55, 89.12) | 12.86 (-68.23, 90.59) | 15.09 (-52.93, 83.95) | 30.59 (-60.34, 123.11) | Cryotherapy | 26.62 (-32.47, 84.89) | 38.05 (-32.19, 106.23) | 14.73 (-52.32, 83.80) |
| -10.11 (-65.05, 46.69) | -10.90 (-46.30, 25.82) | 4.27 (-32.93, 40.04) | -13.25 (-76.36, 42.68) | -11.09 (-64.83, 42.90) | 4.21 (-76.10, 84.42) | -26.62 (-84.89, 32.47) | Curcumin | 12.32 (-43.12, 62.14) | -11.91 (-66.25, 43.24) |
| -21.81 (-85.28, 45.81) | -22.32 (-73.20, 30.67) | -8.37 (-50.89, 39.60) | -25.60 (-89.40, 37.75) | -22.75 (-84.49, 41.25) | -7.25 (-94.01, 79.95) | -38.05 (-106.23, 32.19) | -12.32 (-62.14, 43.12) | Dexamethasone | -23.49 (-86.16, 42.48) |
| 2.17 (-63.22, 66.45) | 0.97 (-50.41, 51.84) | 15.77 (-35.56, 67.22) | -2.32 (-78.55, 70.73) | 0.63 (-63.22, 61.79) | 15.89 (-69.84, 101.03) | -14.73 (-83.80, 52.32) | 11.91 (-43.24, 66.25) | 23.49 (-42.48, 86.16) | Diosmectite |
| -7.59 (-70.14, 58.01) | -8.23 (-56.81, 41.96) | 6.34 (-41.55, 55.82) | -10.62 (-71.71, 43.48) | -8.98 (-71.77, 54.58) | 6.85 (-79.70, 93.21) | -24.20 (-92.15, 44.32) | 2.50 (-44.16, 49.12) | 14.47 (-49.72, 75.95) | -9.09 (-71.17, 54.84) |
| -3.50 (-61.45, 53.69) | -4.99 (-44.59, 38.94) | 10.27 (-23.42, 44.26) | -6.84 (-80.34, 58.73) | -5.07 (-60.41, 51.41) | 11.13 (-71.06, 90.56) | -20.69 (-81.19, 41.15) | 5.64 (-36.57, 55.18) | 18.05 (-34.98, 71.08) | -5.35 (-60.46, 52.08) |
| -18.87 (-73.86, 37.19) | -19.41 (-56.96, 18.51) | -4.54 (-37.60, 27.47) | -21.43 (-92.77, 39.82) | -20.12 (-73.05, 32.44) | -4.36 (-84.73, 73.58) | -35.70 (-93.94, 23.44) | -9.19 (-47.36, 31.63) | 3.85 (-51.16, 54.13) | -20.15 (-73.98, 32.27) |
| 17.40 (-29.12, 63.69) | 16.73 (-8.12, 41.34) | 35.38 (14.93, 55.89) | 12.66 (-55.83, 73.59) | 16.03 (-28.51, 59.58) | 31.75 (-42.37, 105.33) | 0.99 (-51.00, 52.44) | 18.86 (-16.14, 50.56) | 37.20 (-4.07, 78.36) | 15.75 (-28.80, 60.26) |
| 1.15 (-54.11, 57.76) | 0.28 (-38.28, 38.39) | 15.54 (-24.09, 52.81) | -1.98 (-68.18, 56.45) | -0.39 (-54.70, 55.28) | 15.25 (-65.14, 95.36) | -15.40 (-76.57, 44.71) | 11.12 (-25.07, 46.82) | 23.07 (-33.56, 77.06) | -0.50 (-55.21, 54.20) |
| -11.18 (-97.67, 80.56) | -11.98 (-89.29, 66.31) | 2.71 (-75.09, 83.81) | -15.44 (-62.03, 30.84) | -12.90 (-97.73, 74.35) | 2.93 (-103.10, 109.37) | -27.28 (-119.68, 63.81) | -1.37 (-76.30, 75.72) | 11.36 (-69.59, 88.80) | -13.18 (-100.32, 76.15) |
| -7.32 (-58.14, 45.30) | -8.08 (-36.67, 22.32) | 7.08 (-22.76, 36.15) | -10.30 (-68.71, 40.01) | -8.57 (-57.37, 41.16) | 7.04 (-69.55, 84.39) | -23.58 (-79.30, 31.64) | 2.99 (-20.73, 25.98) | 15.06 (-36.04, 61.44) | -8.87 (-58.77, 41.32) |
| -7.07 (-69.48, 57.71) | -8.01 (-56.13, 41.71) | 7.12 (-41.70, 55.88) | -10.39 (-80.76, 53.48) | -8.44 (-70.35, 54.89) | 7.04 (-79.11, 92.55) | -23.28 (-91.76, 44.16) | 2.96 (-43.34, 49.10) | 15.00 (-47.54, 75.59) | -8.61 (-71.15, 53.57) |
| 8.96 (-55.01, 72.96) | 7.83 (-43.63, 57.80) | 22.42 (-28.82, 74.99) | 4.31 (-66.98, 72.63) | 7.27 (-56.29, 70.06) | 23.10 (-62.63, 109.07) | -7.44 (-77.90, 60.34) | 18.19 (-34.76, 72.21) | 29.78 (-31.86, 91.42) | 6.51 (-56.24, 70.80) |

| | | | | | | | | |
|---|---|---|---|---|---|---|---|---|
| 7.59 (-58.01, 70.14) | 3.50 (-53.69, 61.45) | 18.87 (-37.19, 73.86) | -17.40 (-63.69, 29.12) | -1.15 (-57.76, 54.11) | 11.18 (-80.56, 97.67) | 7.32 (-45.30, 58.14) | 7.07 (-57.71, 69.48) | -8.96 (-72.96, 55.01) |
| 8.23 (-41.96, 56.81) | 4.99 (-38.94, 44.59) | 19.41 (-18.51, 56.96) | -16.73 (-41.34, 8.12) | -0.28 (-38.39, 38.28) | 11.98 (-66.31, 89.29) | 8.08 (-22.32, 36.67) | 8.01 (-41.71, 56.13) | -7.83 (-57.80, 43.63) |
| -6.34 (-55.82, 41.55) | -10.27 (-44.26, 23.42) | 4.54 (-27.47, 37.60) | -35.38 (-55.89, -14.93) | -15.54 (-52.81, 24.09) | -2.71 (-83.81, 75.09) | -7.08 (-36.15, 22.76) | -7.12 (-55.88, 41.70) | -22.42 (-74.99, 28.82) |
| 10.62 (-43.48, 71.71) | 6.84 (-58.73, 80.34) | 21.43 (-39.82, 92.77) | -12.66 (-73.59, 55.83) | 1.98 (-56.45, 68.18) | 15.44 (-30.84, 62.03) | 10.30 (-40.01, 68.71) | 10.39 (-53.48, 80.76) | -4.31 (-72.63, 66.98) |
| 8.98 (-54.58, 71.77) | 5.07 (-51.41, 60.41) | 20.12 (-32.44, 73.05) | -16.03 (-59.58, 28.51) | 0.39 (-55.28, 54.70) | 12.90 (-74.35, 97.73) | 8.57 (-41.16, 57.37) | 8.44 (-54.89, 70.35) | -7.27 (-70.06, 56.29) |
| -6.85 (-93.21, 79.70) | -11.13 (-90.56, 71.06) | 4.36 (-73.58, 84.73) | -31.75 (-105.33, 42.37) | -15.25 (-95.36, 65.14) | -2.93 (-109.37, 103.10) | -7.04 (-84.39, 69.55) | -7.04 (-92.55, 79.11) | -23.10 (-109.07, 62.63) |
| 24.20 (-44.32, 92.15) | 20.69 (-41.15, 81.19) | 35.70 (-23.44, 93.94) | -0.99 (-52.44, 51.00) | 15.40 (-44.71, 76.57) | 27.28 (-63.81, 119.68) | 23.58 (-31.64, 79.30) | 23.28 (-44.16, 91.76) | 7.44 (-60.34, 77.90) |
| -2.50 (-49.12, 44.16) | -5.64 (-55.18, 36.57) | 9.19 (-31.63, 47.36) | -18.86 (-50.56, 16.14) | -11.12 (-46.82, 25.07) | 1.37 (-75.72, 76.30) | -2.99 (-25.98, 20.73) | -2.96 (-49.10, 43.34) | -18.19 (-72.21, 34.76) |
| -14.47 (-75.95, 49.72) | -18.05 (-71.08, 34.98) | -3.85 (-54.13, 51.16) | -37.20 (-78.36, 4.07) | -23.07 (-77.06, 33.56) | -11.36 (-88.80, 69.59) | -15.06 (-61.44, 36.04) | -15.00 (-75.59, 47.54) | -29.78 (-91.42, 31.86) |
| 9.09 (-54.84, 71.17) | 5.35 (-52.08, 60.46) | 20.15 (-32.27, 73.98) | -15.75 (-60.26, 28.80) | 0.50 (-54.20, 55.21) | 13.18 (-76.15, 100.32) | 8.87 (-41.32, 58.77) | 8.61 (-53.57, 71.15) | -6.51 (-70.80, 56.24) |
| Doxycycline | -3.84 (-60.83, 51.16) | 11.26 (-40.84, 62.13) | -23.54 (-76.44, 27.93) | -8.94 (-56.69, 40.59) | 3.53 (-72.08, 78.69) | -0.38 (-40.07, 39.80) | -0.26 (-55.73, 53.23) | -16.20 (-77.95, 47.11) |
| 3.84 (-51.16, 60.83) | Glycyrrhiza | 14.79 (-22.09, 52.53) | -32.90 (-55.66, -8.77) | -5.96 (-48.55, 44.75) | 7.62 (-77.50, 88.96) | 2.68 (-33.79, 46.23) | 2.91 (-51.19, 59.04) | -12.17 (-69.11, 44.90) |
| -11.26 (-62.13, 40.84) | -14.79 (-52.53, 22.09) | Laser | -28.03 (-55.09, 1.66) | -20.20 (-59.67, 22.52) | -7.33 (-90.43, 73.07) | -13.67 (-48.48, 20.01) | -12.21 (-61.63, 39.20) | -27.31 (-80.58, 26.99) |
| 23.54 (-27.93, 76.44) | 32.90 (8.77, 55.66) | 28.03 (-1.66, 55.09) | Placebo | 13.37 (-14.41, 39.67) | 28.82 (-48.71, 103.16) | 22.58 (-13.00, 61.29) | 22.62 (-27.95, 74.79) | 8.92 (-35.87, 52.90) |
| 8.94 (-40.59, 56.69) | 5.96 (-44.75, 48.55) | 20.20 (-22.52, 59.67) | -13.37 (-39.67, 14.41) | Probiotics | 12.76 (-65.58, 88.53) | 8.20 (-20.54, 37.16) | 7.88 (-40.76, 56.42) | -7.20 (-61.90, 46.09) |
| -3.53 (-78.69, 72.08) | -7.62 (-88.96, 77.50) | 7.33 (-73.07, 90.43) | -28.82 (-103.16, 48.71) | -12.76 (-88.53, 65.58) | Quercetin | -4.19 (-76.07, 69.39) | -4.33 (-84.26, 78.74) | -19.23 (-105.10, 65.19) |
| 0.38 (-39.80, 40.07) | -2.68 (-46.23, 33.79) | 13.67 (-20.01, 48.48) | -22.58 (-61.29, 13.00) | -8.20 (-37.16, 20.54) | 4.19 (-69.39, 76.07) | Triamcinolone | -0.11 (-40.90, 41.00) | -15.55 (-65.31, 33.96) |
| 0.26 (-53.23, 55.73) | -2.91 (-59.04, 51.19) | 12.21 (-39.20, 61.63) | -22.62 (-74.79, 27.95) | -7.88 (-56.42, 40.76) | 4.33 (-78.74, 84.26) | 0.11 (-41.00, 40.90) | Triester Glycerol Oxide | -15.73 (-77.69, 47.32) |
| 16.20 (-47.11, 77.95) | 12.17 (-44.90, 69.11) | 27.31 (-26.99, 80.58) | -8.92 (-52.90, 35.87) | 7.20 (-46.09, 61.90) | 19.23 (-65.19, 105.10) | 15.55 (-33.96, 65.31) | 15.73 (-47.32, 77.69) | Zinc |

## Inconsistency Factors

| Cycle | Median (95% CI) |
|---|---|
| Aloe, Amlexanox, Placebo, Triamcinolone | 2.20 (-18.83, 32.03) |
| Aloe, Curcumin, Placebo, Triamcinolone | -5.03 (-48.66, 15.75) |
| Aloe, Amlexanox, Dexamethasone, Placebo, Triamcinolone | -0.77 (-35.63, 31.63) |
| Aloe, Amlexanox, Glycyrrhiza, Placebo, Triamcinolone | 8.03 (-10.93, 48.21) |
| Aloe, Amlexanox, Laser, Placebo, Triamcinolone | -5.38 (-43.79, 16.27) |
| Aloe, Placebo, Probiotics, Triamcinolone | -1.52 (-34.62, 23.45) |
| Aloe, Placebo, Triamcinolone | -0.68 (-36.74, 31.29) |
| Aloe, Placebo, Triamcinolone | -0.68 (-36.74, 31.29) |
| Aloe, Placebo, Triamcinolone | -0.68 (-36.74, 31.29) |
| Amlexanox, Laser, Triamcinolone | -0.81 (-37.48, 25.92) |

## Variance Calculation

| Parameter | Median (95% CI) |
|---|---|
| Random Effects Standard Deviation | 21.14 (14.27, 31.66) |
| Inconsistency Standard Deviation | 12.81 (1.61, 45.29) |

## Convergence Diagnostics

Convergence is assessed using the Brooks-Gelman-Rubin method. This method compares within-chain and between-chain variance to calculate the Potential Scale Reduction Factor (PSRF). A PSRF close to one indicates approximate convergence has been reached.

| Parameter | PSRF |
|---|---|
| d.Allicin.Placebo | 1.00 |
| d.Aloe.Triamcinolone | 1.00 |
| d.Amlexanox.Dexamethasone | 1.53 |
| d.Amlexanox.Glycyrrhiza | 1.01 |
| d.Benzydamine.Doxycycline | 1.72 |
| d.Benzydamine.Quercetin | 1.00 |
| d.Glycyrrhiza.Laser | 1.01 |
| d.Placebo.Aloe | 1.00 |
| d.Placebo.Berberinegelatin | 1.00 |
| d.Placebo.Chitosan | 1.00 |
| d.Placebo.Cryotherapy | 1.00 |
| d.Placebo.Diosmectite | 1.00 |
| d.Placebo.Zinc | 103 |
| d.Triamcinolone.Amlexanox | 1.02 |
| d.Triamcinolone.Benzydamine | 1.80 |
| d.Triamcinolone.Curcumin | 1.00 |
| d.Triamcinolone.Probiotics | 1.00 |
| d.Triamcinolone.TriesterGlycerolOxide | 1.00 |
| w.Aloe.Placebo.Amlexanox.Triamcinolone | 1.01 |
| w.Aloe.Placebo.Curcumin.Triamcinolone | 1.00 |
| w.Aloe.Placebo.Dexamethasone.Amlexanox.Triamcinolone | 1.06 |
| w.Aloe.Placebo.Glycyrrhiza.Amlexanox.Triamcinolone | 1.00 |
| w.Aloe.Placebo.Laser.Amlexanox.Triamcinolone | 1.01 |
| w.Aloe.Placebo.Prpbiotics.Triamcinolone | 1.00 |
| w.Aloe.Placebo.Triamcinolone | 1.01 |
| w.Aloe.Placebo.Triamcinolone | 1.01 |
| w.Aloe.Placebo.Triamcinolone | 1.01 |
| w.Amlexanox.Laser.Triamcinolone | 1.03 |
| sd.d | 1.00 |
| sd.w | 1.01 |

Number of chains : 4
Tuning iterations : 20,000
Simulation iterations : 50,000
Thinning interval : 10
Inference samples : 10,000
Variance scaling factor : 2.5

## 3. Node-splitting analysis

### Node-splitting analysis of inconsistency

Inconsistency between direct and indirect estimates was estimated in the Node-Splitting Model. When P values is above 0.05 in a comparison, there is evidence of statistical inconsistency.

| Name | Direct Effect | Indirect Effect | Overall | P-Value |
|---|---|---|---|---|
| Aloe, Triamcinolone | 10.21 (-40.60, 60.46) | 4.55 (-31.80, 43.91) | 8.38 (-21.59, 40.18) | 0.85 |
| Amlexanox, Dexamethasone | 12.53 (-43.51, 79.80) | -3.44 (-62.35, 53.60) | 1.26 (-35.76, 38.69) | 0.67 |
| Amlexanox, Glycyrrhiza | -13.76 (-68.65, 39.83) | -4.21 (-44.62, 35.59) | -5.83 (-37.06, 23.74) | 0.76 |
| Amlexanox, Laser | 10.51 (-40.36, 60.36) | -24.58 (-72.00, 13.79) | -7.47 (-41.98, 22.72) | 0.26 |
| Amlexanox, Placebo | -43.87 (-67.14, -20.94) | -19.63 (-51.37, 13.55) | -35.29 (-54.72, -15.53) | 0.2 |
| Amlexanox, Triamcinolone | 8.63 (-39.59, 55.83) | -18.98 (-47.01, 10.06) | -9.29 (-33.32, 16.84) | 0.32 |
| Benzydamine, Placebo | -20.20 (-76.55, 34.13) | -24.22 (-78.47, 32.21) | -23.99 (-73.07, 26.19) | 0.92 |
| Benzydamine, Triamcinolone | 5.34 (-63.88, 65.60) | 2.60 (-81.48, 67.53) | 2.15 (-47.05, 54.59) | 0.85 |
| Curcumin, Placebo | -2.16 (-49.56, 44.59) | -36.72 (-69.53, -4.83) | -26.41 (-54.91, 1.26) | 0.22 |
| Curcumin, Triamcinolone | -8.02 (-35.04, 19.46) | 27.96 (-25.49, 81.28) | -0.59 (-24.70, 24.02) | 0.23 |
| Dexamethasone, Placebo | -32.35 (-87.66, 24.74) | -37.62 (-94.74, 21.31) | -36.62 (-74.85, 1.39) | 0.9 |
| Glycyrrhiza, Laser | 22.88 (-23.37, 69.23) | -18.36 (-55.30, 18.14) | -1.50 (-38.92, 30.74) | 0.16 |
| Laser, Placebo | -36.29 (-63.60, -7.19) | -34.32 (-72.05, 3.79) | -27.74 (-53.44, 3.04) | 0.92 |
| Laser, Triamcinolone | 6.70 (-52.76, 63.20) | -9.90 (-43.36, 25.94) | -1.95 (-31.42, 34.44) | 0.69 |
| Placebo, Probiotics | 10.48 (-24.65, 45.32) | 18.10 (-37.81, 65.62) | 15.25 (-12.19, 43.58) | 0.81 |
| Placebo, Triamcinolone | 30.78 (1.50, 61.86) | 26.78 (-4.03, 57.58) | 25.83 (7.91, 45.48) | 0.84 |
| Probiotics, Triamcinolone | 5.09 (-33.75, 44.69) | 9.61 (-30.68, 49.79) | 10.59 (-16.93, 39.27) | 0.87 |

## 4. Network structure

### Network structure of sensitivity analysis

37 RCTs involving 19 local interventions were included in the sensitivity analysis considering the size-reducing effect.

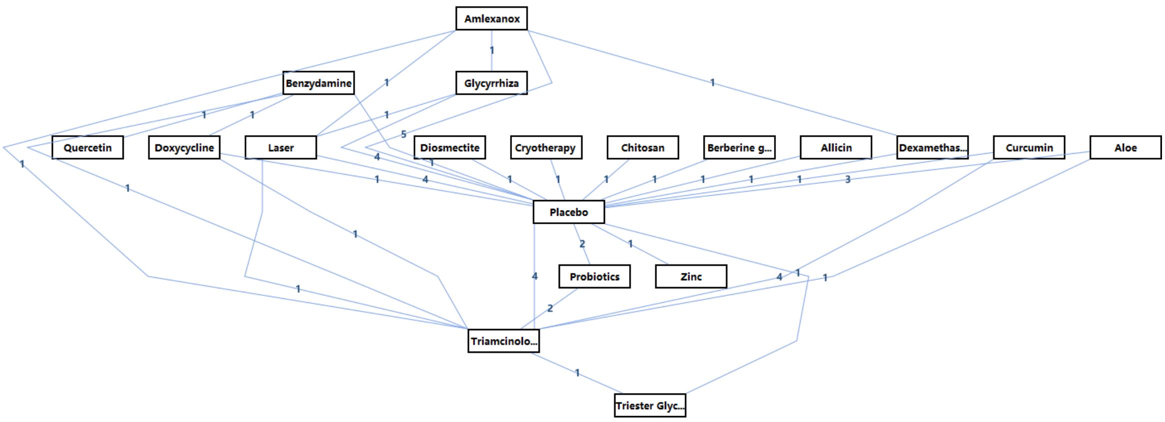

## 5. Pairwise meta-analysis

### Pairwise meta-analysis of size-reducing effect

Size-reducing effect were measured by mean difference (MD) and 95% confidence interval (CI).

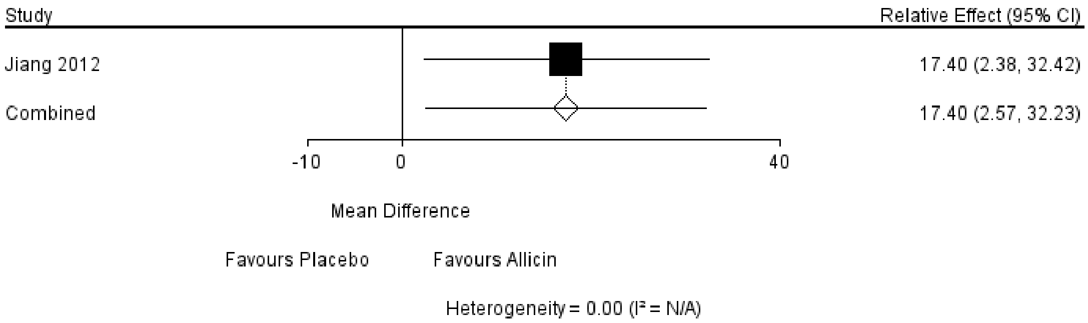


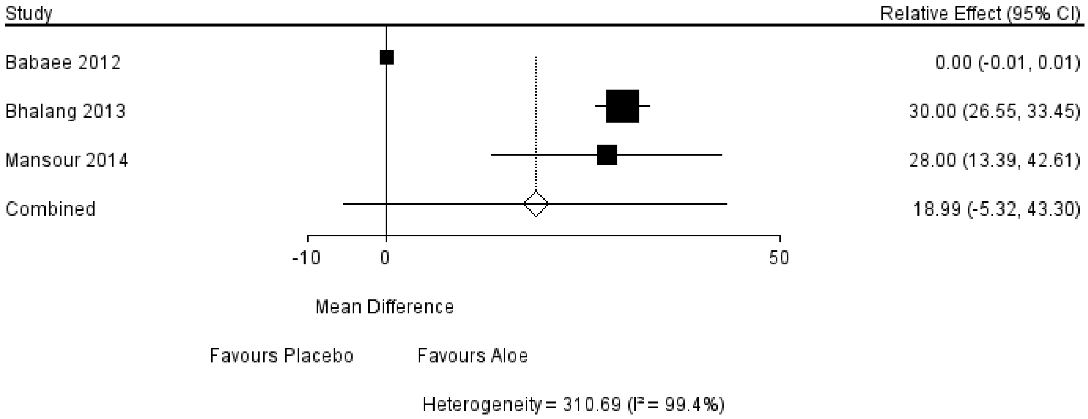


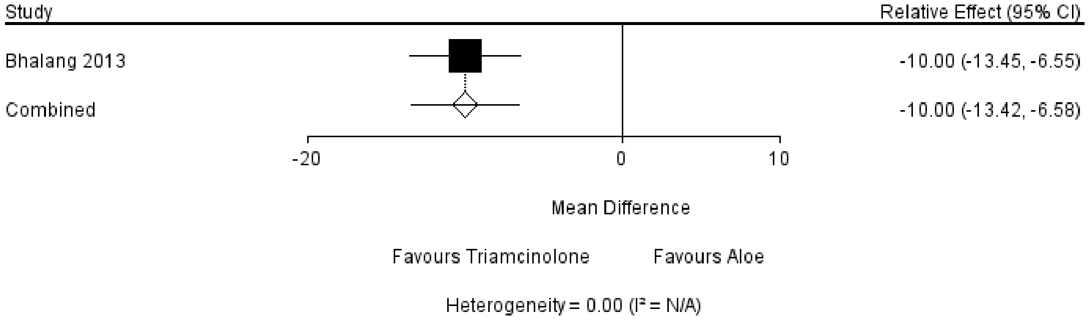


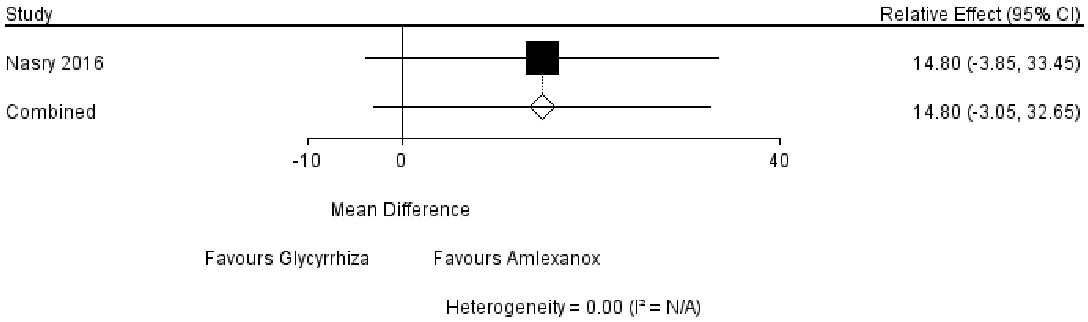

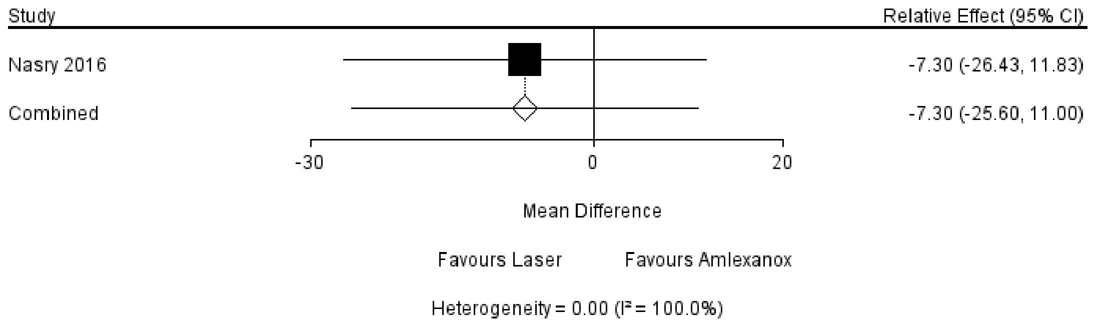
Study
Relative Effect (95% CI)
Nasry 2016
-7.30 (-26.43, 11.83)
Combined
-7.30 (-25.60, 11.00)
-30
0
20
Mean Difference
Favours Laser
Favours Amlexanox
Heterogeneity = 0.00 (I² = 100.0%)

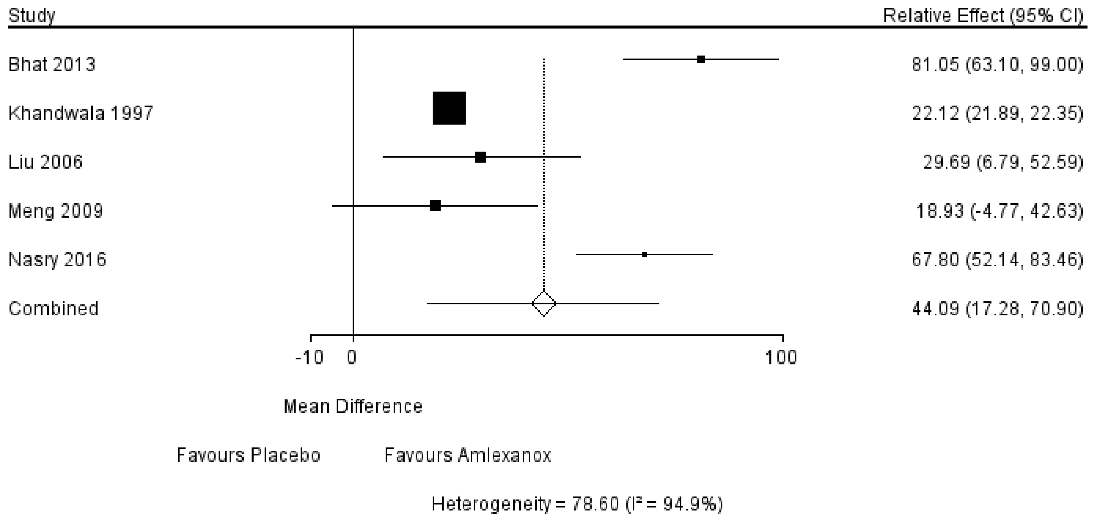
Study
Relative Effect (95% CI)
Bhat 2013
81.05 (63.10, 99.00)
Khandwala 1997
22.12 (21.89, 22.35)
Liu 2006
29.69 (6.79, 52.59)
Meng 2009
18.93 (-4.77, 42.63)
Nasry 2016
67.80 (52.14, 83.46)
Combined
44.09 (17.28, 70.90)
-10
0
100
Mean Difference
Favours Placebo
Favours Amlexanox
Heterogeneity = 78.60 (I² = 94.9%)

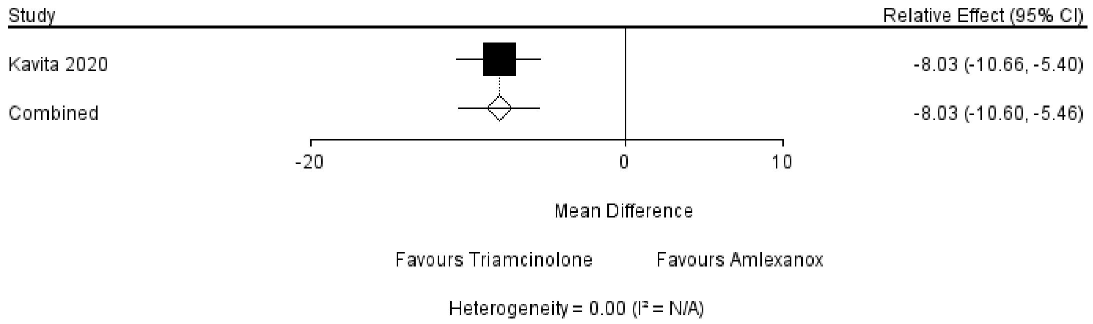
Study
Relative Effect (95% CI)
Kavita 2020
-8.03 (-10.66, -5.40)
Combined
-8.03 (-10.60, -5.46)
-20
0
10
Mean Difference
Favours Triamcinolone
Favours Amlexanox
Heterogeneity = 0.00 (I² = N/A)

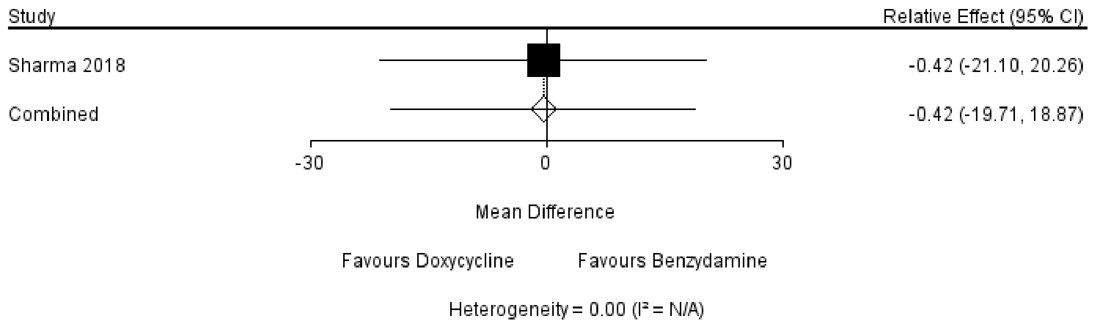
Study
Relative Effect (95% CI)
Sharma 2018
-0.42 (-21.10, 20.26)
Combined
-0.42 (-19.71, 18.87)
-30
0
30
Mean Difference
Favours Doxycycline
Favours Benzydamine
Heterogeneity = 0.00 (I² = N/A)

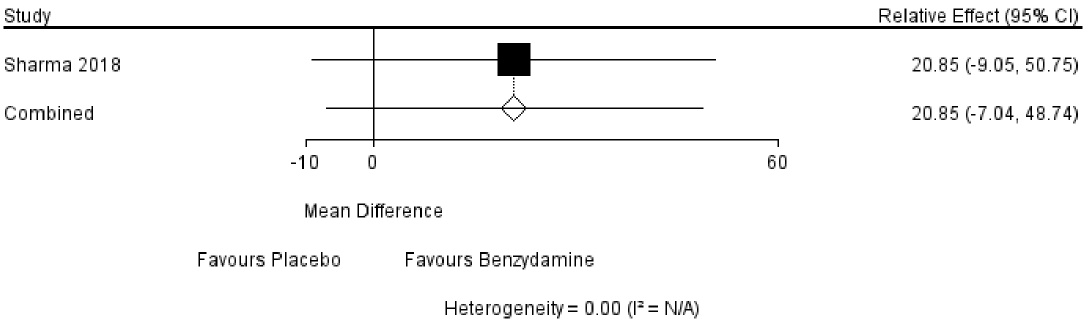
Study
Relative Effect (95% CI)
Sharma 2018
20.85 (-9.05, 50.75)
Combined
20.85 (-7.04, 48.74)
-10
0
60
Mean Difference
Favours Placebo
Favours Benzydamine
Heterogeneity = 0.00 (I² = N/A)

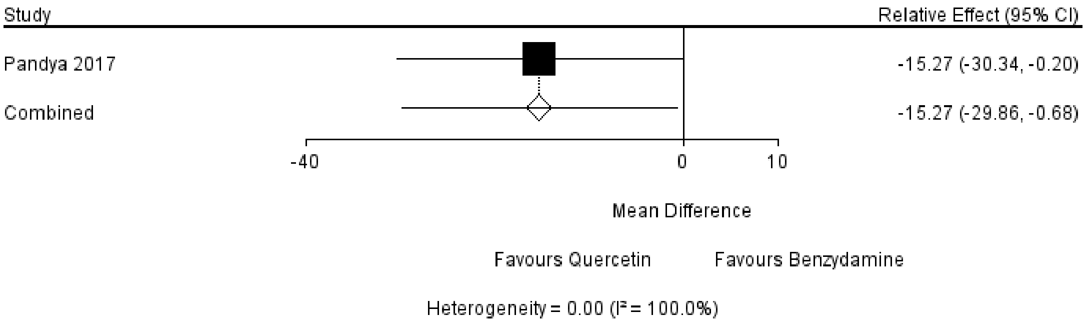
Study
Relative Effect (95% CI)
Pandya 2017
-15.27 (-30.34, -0.20)
Combined
-15.27 (-29.86, -0.68)
-40
0
10
Mean Difference
Favours Quercetin
Favours Benzydamine
Heterogeneity = 0.00 (I² = 100.0%)

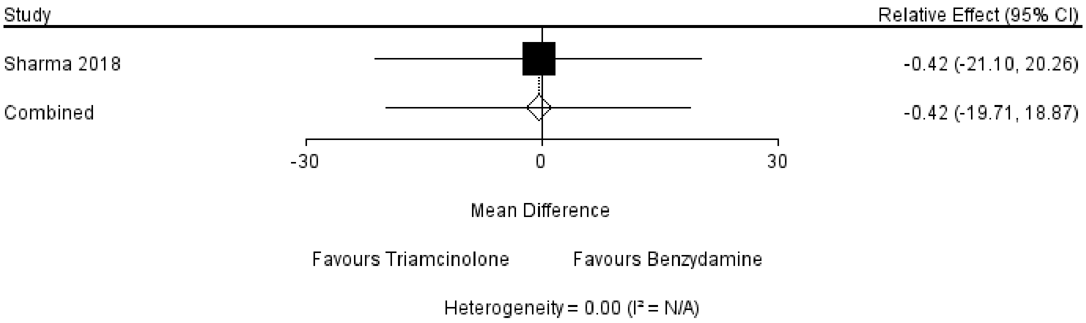
Study
Relative Effect (95% CI)
Sharma 2018
-0.42 (-21.10, 20.26)
Combined
-0.42 (-19.71, 18.87)
-30
0
30
Mean Difference
Favours Triamcinolone
Favours Benzydamine
Heterogeneity = 0.00 (I² = N/A)

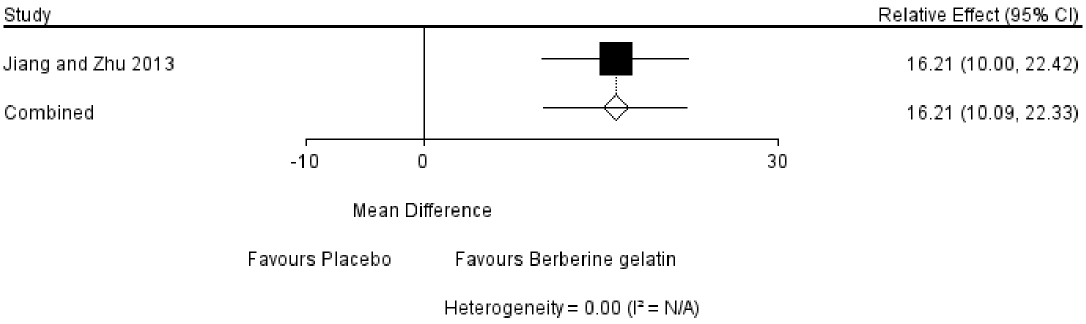
Study
Relative Effect (95% CI)
Jiang and Zhu 2013
16.21 (10.00, 22.42)
Combined
16.21 (10.09, 22.33)
-10
0
30
Mean Difference
Favours Placebo
Favours Berberine gelatin
Heterogeneity = 0.00 (I² = N/A)

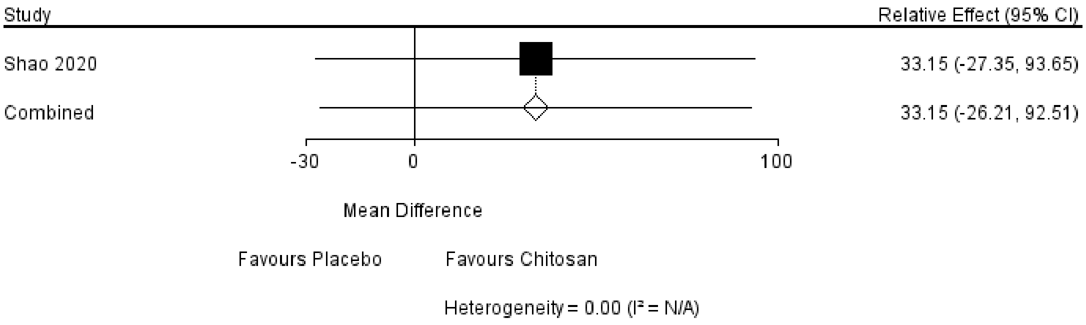
Study
Relative Effect (95% CI)
Shao 2020
33.15 (-27.35, 93.65)
Combined
33.15 (-26.21, 92.51)
-30
0
100
Mean Difference
Favours Placebo
Favours Chitosan
Heterogeneity = 0.00 (I² = N/A)

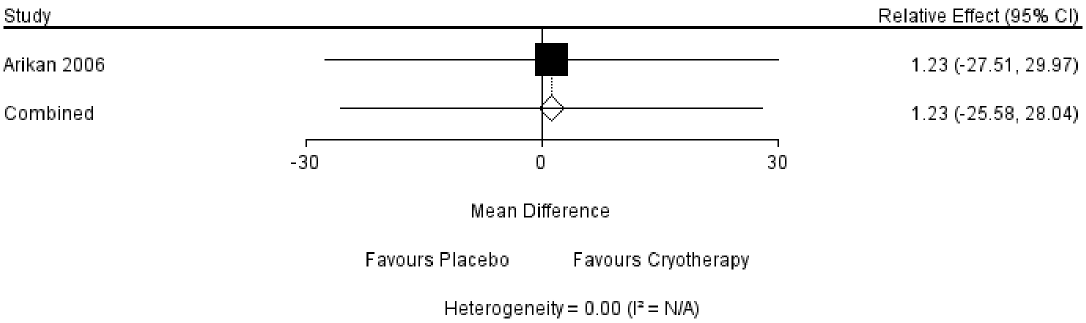
Study
Relative Effect (95% CI)
Arikan 2006
1.23 (-27.51, 29.97)
Combined
1.23 (-25.58, 28.04)
-30
0
30
Mean Difference
Favours Placebo
Favours Cryotherapy
Heterogeneity = 0.00 (I² = N/A)

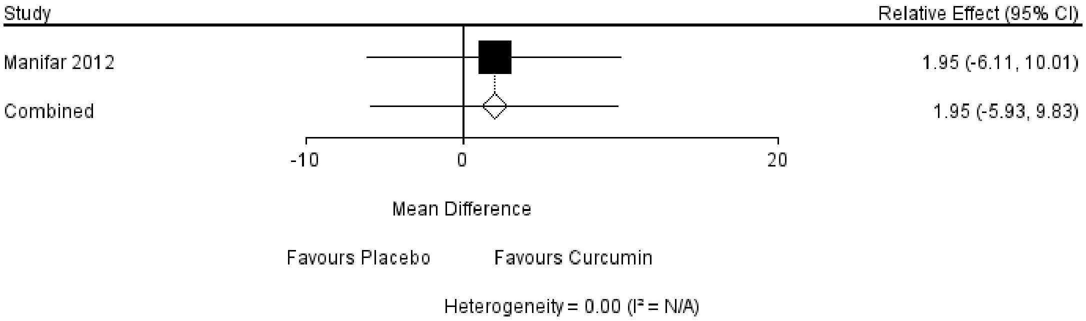
Study
Relative Effect (95% CI)
Manifar 2012
1.95 (-6.11, 10.01)
Combined
1.95 (-5.93, 9.83)
-10
0
20
Mean Difference
Favours Placebo
Favours Curcumin
Heterogeneity = 0.00 (I² = N/A)

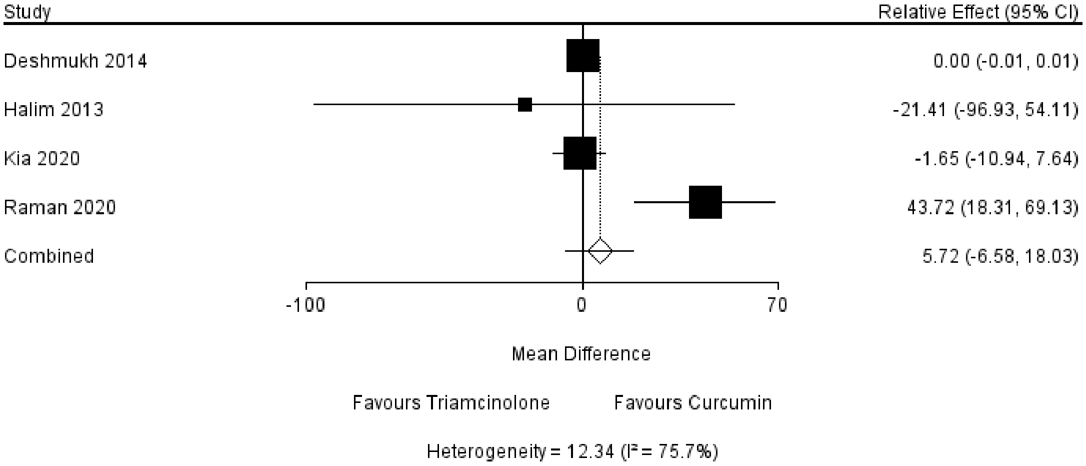
Study
Relative Effect (95% CI)
Deshmukh 2014
0.00 (-0.01, 0.01)
Halim 2013
-21.41 (-96.93, 54.11)
Kia 2020
-1.65 (-10.94, 7.64)
Raman 2020
43.72 (18.31, 69.13)
Combined
5.72 (-6.58, 18.03)
-100
0
70
Mean Difference
Favours Triamcinolone
Favours Curcumin
Heterogeneity = 12.34 (I² = 75.7%)

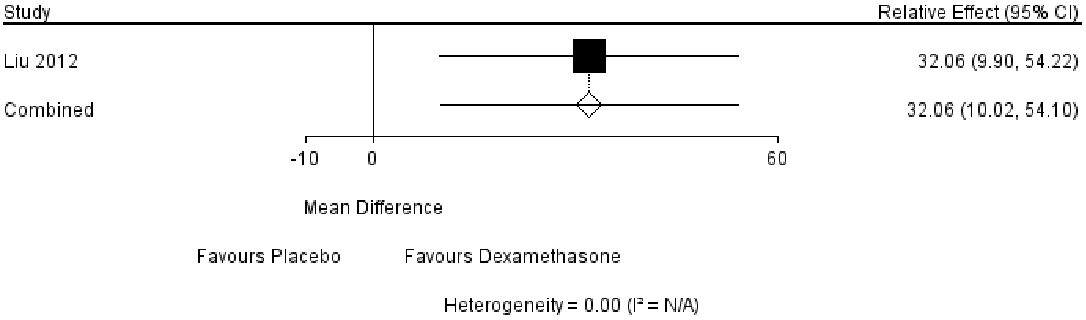
Study
Relative Effect (95% CI)
Liu 2012
32.06 (9.90, 54.22)
Combined
32.06 (10.02, 54.10)
-10
0
60
Mean Difference
Favours Placebo
Favours Dexamethasone
Heterogeneity = 0.00 (I² = N/A)

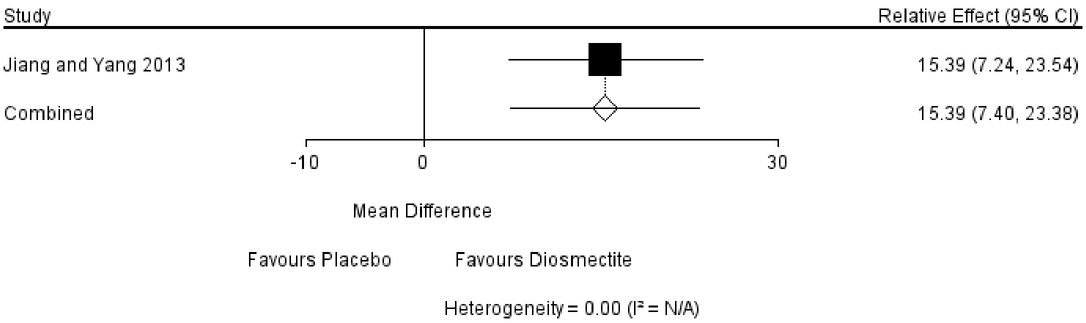
Study
Relative Effect (95% CI)
Jiang and Yang 2013
15.39 (7.24, 23.54)
Combined
15.39 (7.40, 23.38)
-10
0
30
Mean Difference
Favours Placebo
Favours Diosmectite
Heterogeneity = 0.00 (I² = N/A)

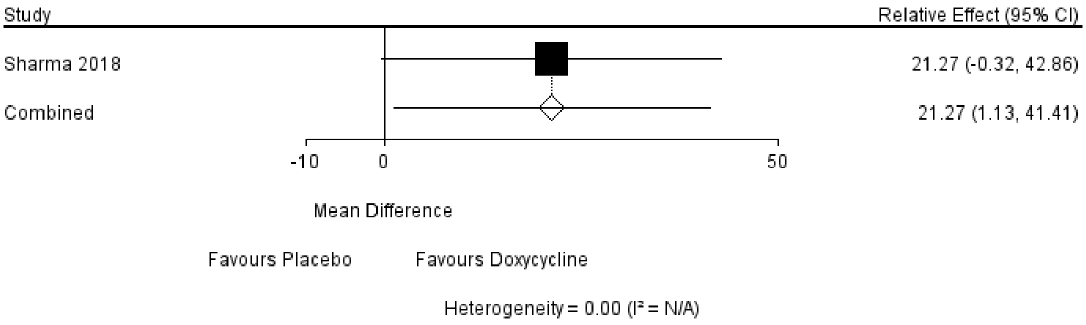
Study
Relative Effect (95% CI)
Sharma 2018
21.27 (-0.32, 42.86)
Combined
21.27 (1.13, 41.41)
-10
0
50
Mean Difference
Favours Placebo
Favours Doxycycline
Heterogeneity = 0.00 (I² = N/A)

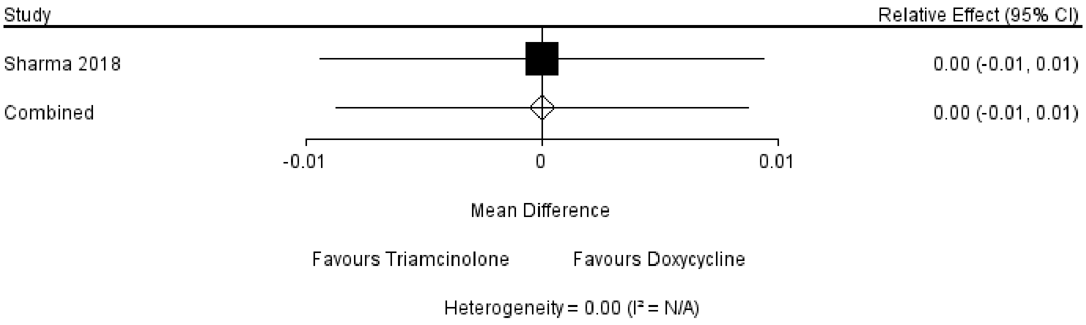
Study
Relative Effect (95% CI)
Sharma 2018
0.00 (-0.01, 0.01)
Combined
0.00 (-0.01, 0.01)
-0.01
0
0.01
Mean Difference
Favours Triamcinolone
Favours Doxycycline
Heterogeneity = 0.00 (I² = N/A)

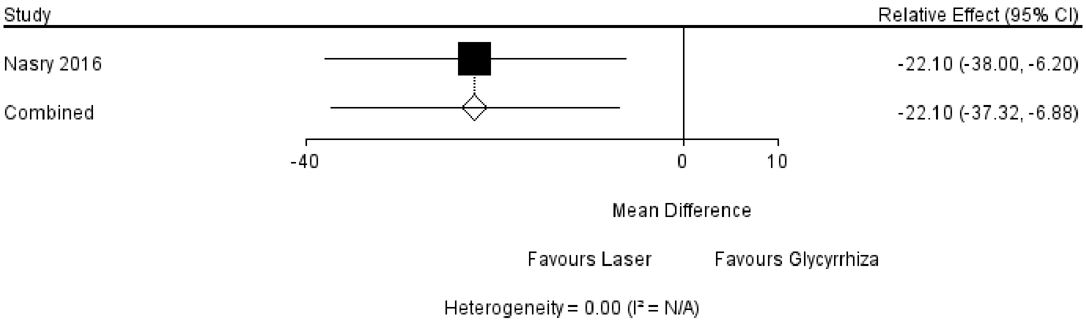
Study
Relative Effect (95% CI)
Nasry 2016
-22.10 (-38.00, -6.20)
Combined
-22.10 (-37.32, -6.88)
-40
0
10
Mean Difference
Favours Laser
Favours Glycyrrhiza
Heterogeneity = 0.00 (I² = N/A)

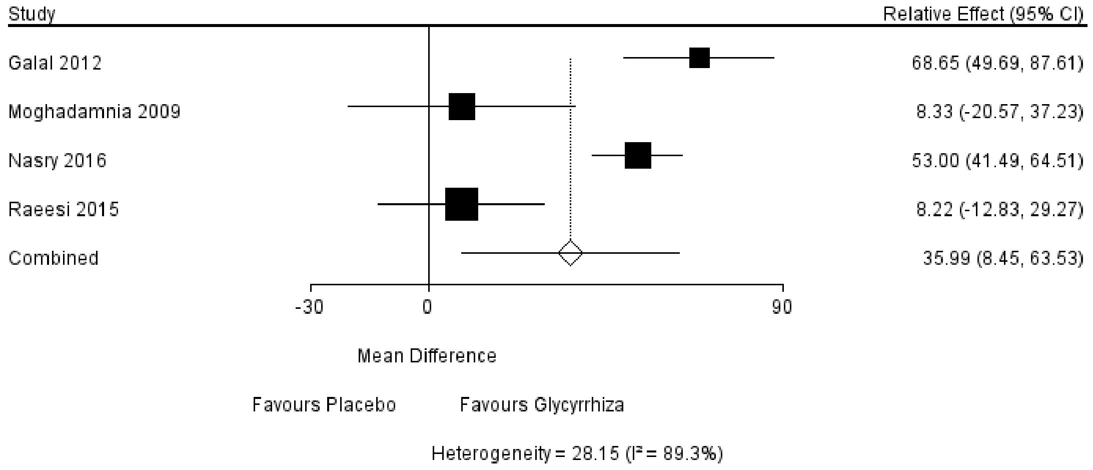
Study
Relative Effect (95% CI)
Galal 2012
68.65 (49.69, 87.61)
Moghadamnia 2009
8.33 (-20.57, 37.23)
Nasry 2016
53.00 (41.49, 64.51)
Raeesi 2015
8.22 (-12.83, 29.27)
Combined
35.99 (8.45, 63.53)
-30
0
90
Mean Difference
Favours Placebo
Favours Glycyrrhiza
Heterogeneity = 28.15 (I² = 89.3%)

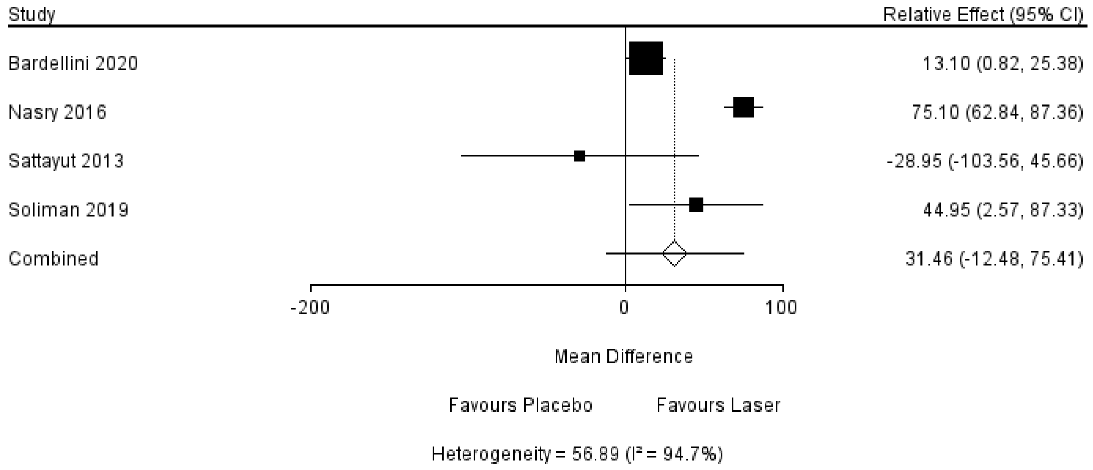
Study
Relative Effect (95% CI)
Bardellini 2020
13.10 (0.82, 25.38)
Nasry 2016
75.10 (62.84, 87.36)
Sattayut 2013
-28.95 (-103.56, 45.66)
Soliman 2019
44.95 (2.57, 87.33)
Combined
31.46 (-12.48, 75.41)
-200
0
100
Mean Difference
Favours Placebo
Favours Laser
Heterogeneity = 56.89 (I² = 94.7%)

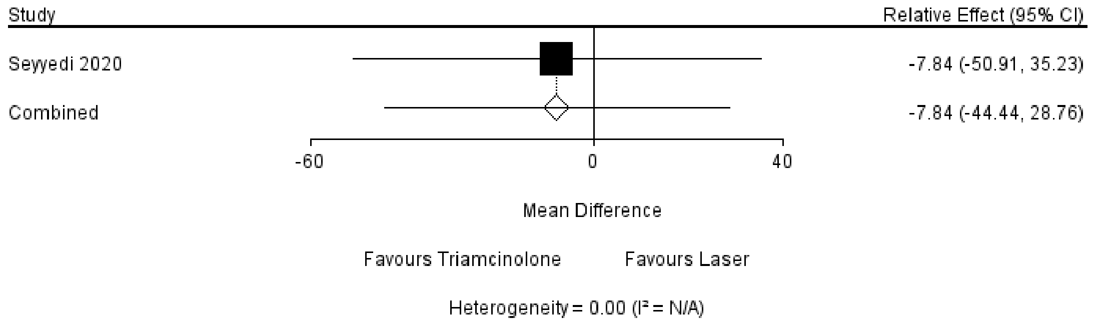
Study
Relative Effect (95% CI)
Seyyedi 2020
-7.84 (-50.91, 35.23)
Combined
-7.84 (-44.44, 28.76)
-60
0
40
Mean Difference
Favours Triamcinolone
Favours Laser
Heterogeneity = 0.00 (I² = N/A)

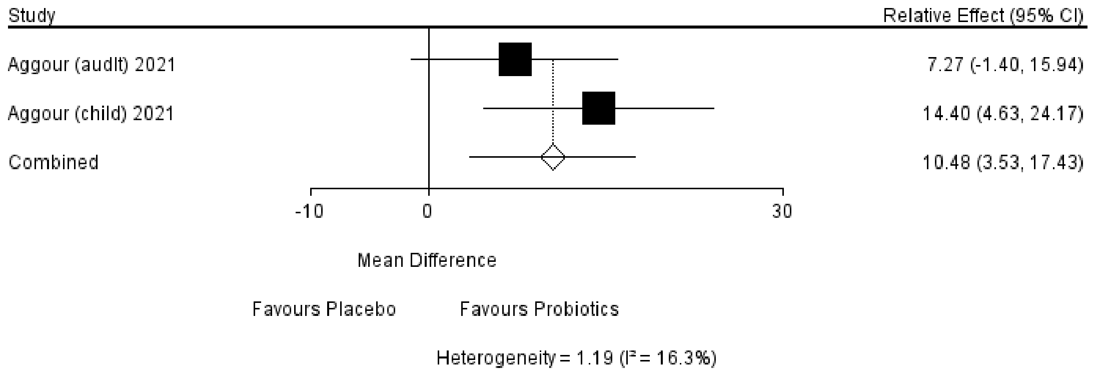
Study
Relative Effect (95% CI)
Aggour (audit) 2021
7.27 (-1.40, 15.94)
Aggour (child) 2021
14.40 (4.63, 24.17)
Combined
10.48 (3.53, 17.43)
-10
0
30
Mean Difference
Favours Placebo
Favours Probiotics
Heterogeneity = 1.19 (I² = 16.3%)

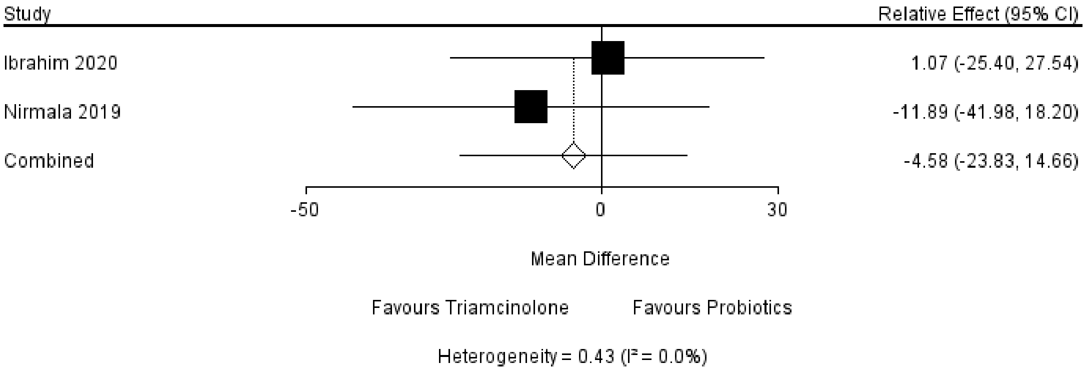
Study
Relative Effect (95% CI)
Ibrahim 2020
1.07 (-25.40, 27.54)
Nirmala 2019
-11.89 (-41.98, 18.20)
Combined
-4.58 (-23.83, 14.66)
-50
0
30
Mean Difference
Favours Triamcinolone
Favours Probiotics
Heterogeneity = 0.43 (I² = 0.0%)

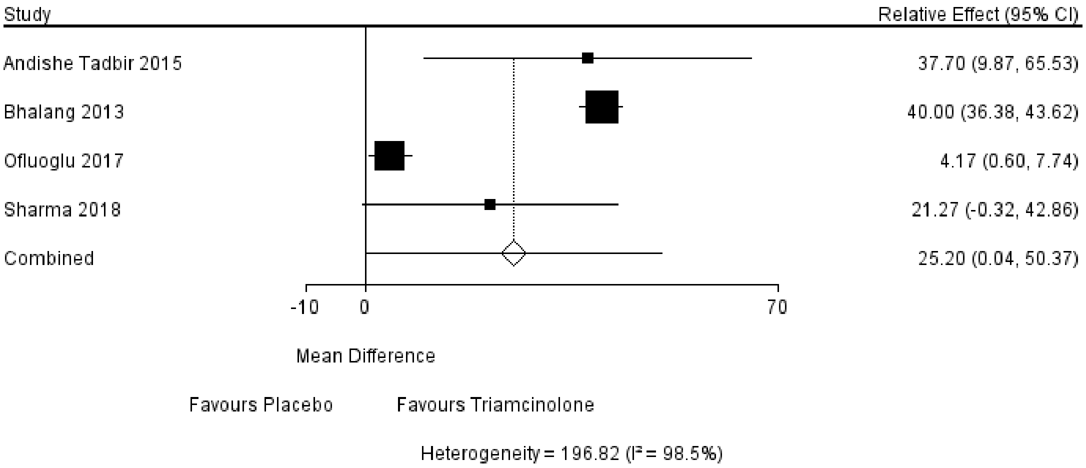
Study
Relative Effect (95% CI)
Andishe Tadbir 2015
37.70 (9.87, 65.53)
Bhalang 2013
40.00 (36.38, 43.62)
Ofluoglu 2017
4.17 (0.60, 7.74)
Sharma 2018
21.27 (-0.32, 42.86)
Combined
25.20 (0.04, 50.37)
-10
0
70
Mean Difference
Favours Placebo
Favours Triamcinolone
Heterogeneity = 196.82 (I² = 98.5%)

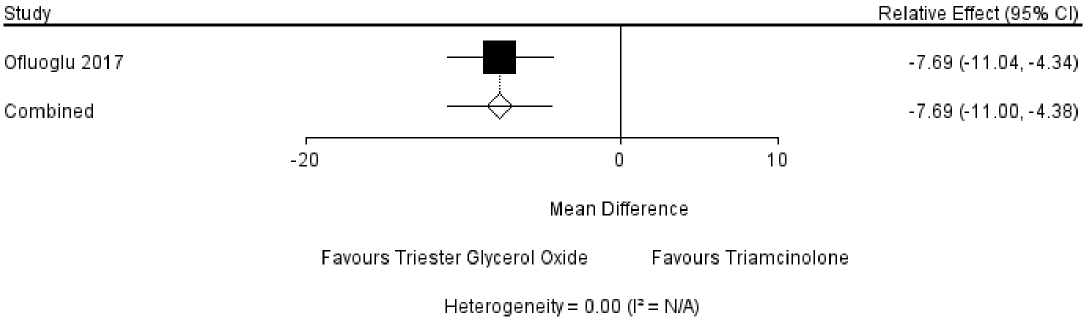
Study
Relative Effect (95% CI)
Ofluoglu 2017
-7.69 (-11.04, -4.34)
Combined
-7.69 (-11.00, -4.38)
-20
0
10
Mean Difference
Favours Triester Glycerol Oxide
Favours Triamcinolone
Heterogeneity = 0.00 (I² = N/A)

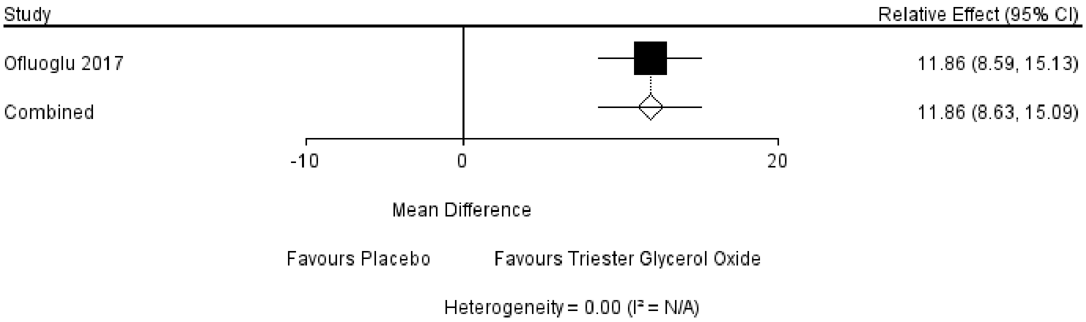
Study
Relative Effect (95% CI)
Ofluoglu 2017
11.86 (8.59, 15.13)
Combined
11.86 (8.63, 15.09)
-10
0
20
Mean Difference
Favours Placebo
Favours Triester Glycerol Oxide
Heterogeneity = 0.00 (I² = N/A)

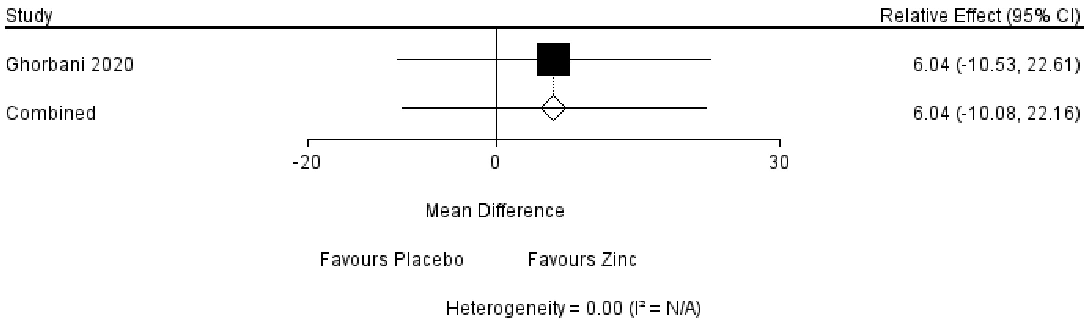
Study
Relative Effect (95% CI)
Ghorbani 2020
6.04 (-10.53, 22.61)
Combined
6.04 (-10.08, 22.16)
-20
0
30
Mean Difference
Favours Placebo
Favours Zinc
Heterogeneity = 0.00 (I² = N/A)

| Comparison | NO. of included study | Heterogeneity ($I^2$) | Combined effect [MD (95%CI)] |
|---|---|---|---|
| Allicin vs | | | |
| Placebo | 1 | NA | 17.40 (2.57, 32.23) |
| Aloe vs | | | |
| Placebo | 3 | 99.4% | 18.99 (-5.32, 43.30) |
| Triamcinolone | 1 | NA | -10.00 (-13.42, -6.58) |
| Amlexanox vs | | | |
| Glycyrrhiza | 1 | NA | 14.80 (-3.05, 32.65) |
| Laser | 1 | NA | -7.30 (-25.60, 11.00) |
| Placebo | 5 | 95% | 44.09 (17.28, 70.90) |
| Triamcinolone | 1 | NA | -8.03 (-10.60, -5.46) |
| Benzydamine vs | | | |
| Placebo | 1 | NA | 20.85 (-7.04, 48.74) |
| Triamcinolone | 1 | NA | -0.42 (-19.71, 18.87) |
| Quercetin | 1 | NA | -15.27 (-29.86, -0.68) |
| Doxycycline | 1 | NA | -0.42 (-19.71, 18.87) |
| Berberine gelatin vs | | | |
| Placebo | 1 | NA | 16.21 (10.09, 22.33) |
| Chitosan vs | | | |
| Placebo | 1 | NA | 33.15 (-26.21, 92.51) |
| Cryotherapy vs | | | |
| Placebo | 1 | NA | 1.23 (-25.58, 28.04) |
| Curcumin vs | | | |
| Placebo | 1 | NA | 1.95 (-5.93, 9.83) |
| Triamcinolone | 4 | 75.7% | 5.72 (-6.58, 18.03) |
| Dexamethasone vs | | | |
| Placebo | 1 | NA | 32.06 (10.02, 54.10) |
| Diosmectite vs | | | |
| Placebo | 1 | NA | 15.39 (7.40, 23.38) |
| Doxycycline vs | | | |
| Placebo | 1 | NA | 21.27 (1.13, 41.41) |
| Triamcinolone | 1 | NA | 0 (-0.01, 0.01) |
| Glycyrrhiza vs | | | |
| Laser | 1 | NA | -22.10 (-37.32, -6.88) |
| Placebo | 4 | 89.3% | 35.99 (8.45, 63.53) |
| Laser vs | | | |
| Placebo | 4 | 94.7% | 31.46 (-12.48, 75.41) |
| Triamcinolone | 1 | NA | -7.84 (-44.44, 28.76) |
| Probiotics vs | | | |
| Triamcinolone | 2 | 0% | -4.58 (-23.83, 14.66) |
| Placebo | 2 | 16.3% | 10.48 (3.53, 17.43) |
| Triamcinolone vs | | | |
| Triester Glycerol Oxide | 1 | NA | -7.69 (-11.00, -4.38) |
| Placebo | 4 | 98.5% | 25.20 (0.04, 50.37) |
| Triester Glycerol Oxide vs | | | |
| Placebo | 1 | NA | 11.86 (8.63, 15.09) |
| Zinc vs | | | |
| Placebo | 1 | NA | 6.04 (-10.08, 22.16) |

MD, mean difference; CI, confidence interval; NA, not applicable.

**6. Subgroup Discussion**

The 51 RCTs studied for the size-reducing effect had different durations of local intervention and were examined at different times during the trial. The size-reducing effect is discussed separately according to the different examination times.

**Day 1**

**1. Consistency Model**

1.1 Summary estimates

| | | |
|---|---|---|
| Glycyrrhiza | -14.16 (-176.75, 151.03) | -30.05 (-70.88, 13.06) |
| 14.16 (-151.03, 176.75) | Laser | -16.19 (-175.48, 143.23) |
| 30.05 (-13.06, 70.88) | 16.19 (-143.23, 175.48) | Placebo |

1.2 Rank probability(Rank 1 is best, rank N is worst)

| Drug | Rank 1 | Rank 2 | Rank 3 |
|---|---|---|---|
| Glycyrrhiza | 0.54 | 0.42 | 0.04 |
| Laser | 0.44 | 0.14 | 0.42 |
| Placebo | 0.03 | 0.44 | 0.53 |

1.3 Consistency check

| Parameter | Median (95% CI) |
|---|---|
| Random Effects Standard Deviation | 14.87 (0.80, 28.99) |

1.4 Convergence Diagnostics

| Parameter | PSRF |
|---|---|
| d.Placebo.Glycyrrhiza | 1.00 |
| d.Placebo.Laser | 1.00 |
| sd.d | 1.00 |

Number of chains : 4
Tuning iterations : 20,000
Simulation iterations : 50,000
Thinning interval : 10
Inference samples : 10,000
Variance scaling factor: 2.5

**2. Inconsistency Model**

2.1 Summary estimates

| | | |
|---|---|---|
| Glycyrrhiza | -20.64 (-189.90, 145.55) | -30.03 (-70.13, 10.42) |
| 20.64 (-145.55, 189.90) | Laser | -8.58 (-173.98, 152.25) |
| 30.03 (-10.42, 70.13) | 8.58 (-152.25, 173.98) | Placebo |

2.2Variance Calculation

| Parameter | Median (95% CI) |
|---|---|
| Random Effects Standard Deviation | 14.83 (1.06, 29.05) |
| Inconsistency Standard Deviation | 14.67 (0.65, 29.11) |

2.3Convergence Diagnostics

| Parameter | PSRF |
|---|---|
| d.Glycyrrhiza.Placebo | 1.00 |
| d.Placebo.Laser | 1.00 |
| sd.d | 1.00 |
| sd.w | 1.00 |

Number of chains : 4
Tuning iterations : 20,000
Simulation iterations : 50,000
Thinning interval : 10
Inference samples : 10,000
Variance scaling factor: 2.5

## 3. Network structure

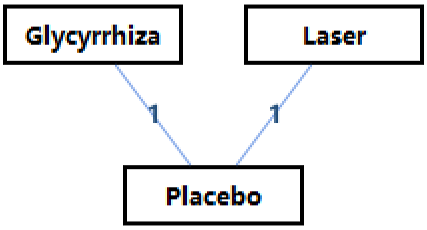


**Day 2**

## 1. Consistency Model

### 1.1 Summary estimates

| | | | | | |
|---|---|---|---|---|---|
| Allicin | -13.70 (-63.05, 33.47) | 24.39 (-24.56, 76.19) | 3.46 (-50.45, 52.71) | -3.42 (-74.82, 66.63) | -19.44 (-77.92, 41.05) |
| 13.70 (-33.47, 63.05) | Aloe | 38.04 (-7.69, 86.27) | 17.32 (-33.58, 66.82) | 11.16 (-58.87, 78.60) | -5.23 (-58.69, 47.65) |
| -24.39 (-76.19, 24.56) | -38.04 (-86.27, 7.69) | Amlexanox | -20.67 (-74.21, 25.80) | -27.46 (-99.55, 39.81) | -43.54 (-105.04, 13.31) |
| -3.46 (-52.71, 50.45) | -17.32 (-66.82, 33.58) | 20.67 (-25.80, 74.21) | Berberine gelatin | -6.51 (-75.23, 62.83) | -22.27 (-80.93, 39.89) |
| 3.42 (-66.63, 74.82) | -11.16 (-78.60, 58.87) | 27.46 (-39.81, 99.55) | 6.51 (-62.83, 75.23) | Chitosan | -15.23 (-96.40, 64.14) |
| 19.44 (-41.05, 77.92) | 5.23 (-47.65, 58.69) | 43.54 (-13.31, 105.04) | 22.27 (-39.89, 80.93) | 15.23 (-64.14, 96.40) | Curcumin |
| 20.43 (-29.62, 72.96) | 7.17 (-41.92, 55.98) | 45.37 (-3.54, 98.77) | 24.13 (-29.14, 74.88) | 18.08 (-53.83, 86.75) | 2.04 (-59.84, 62.81) |
| -21.65 (-68.28, 20.45) | -35.11 (-81.73, 3.81) | 2.64 (-32.71, 36.02) | -17.98 (-68.83, 23.22) | -25.38 (-93.64, 40.83) | -41.14 (-101.38, 13.73) |
| -28.90 (-75.72, 22.05) | -42.53 (-89.37, 6.20) | -4.37 (-38.90, 35.59) | -25.72 (-75.87, 23.78) | -31.30 (-102.46, 37.53) | -48.00 (-106.99, 12.56) |
| 17.72 (-16.34, 53.85) | 4.05 (-29.23, 37.89) | 42.46 (9.55, 77.85) | 21.24 (-15.04, 56.95) | 14.57 (-46.97, 77.06) | -0.93 (-50.49, 47.71) |
| 14.27 (-30.08, 60.43) | 0.78 (-33.48, 34.08) | 38.95 (-1.04, 82.97) | 17.93 (-27.47, 60.98) | 11.33 (-55.84, 77.97) | -4.35 (-47.24, 37.64) |
| 12.96 (-36.22, 62.11) | -0.75 (-44.57, 43.22) | 37.34 (-8.80, 86.25) | 16.58 (-32.09, 63.26) | 10.08 (-59.02, 78.82) | -5.51 (-62.29, 46.12) |

| | | | | | |
|---|---|---|---|---|---|
| -20.43 (-72.96, 29.62) | 21.65 (-20.45, 68.28) | 28.90 (-22.05, 75.72) | -17.72 (-53.85, 16.34) | -14.27 (-60.43, 30.08) | -12.96 (-62.11, 36.22) |
| -7.17 (-55.98, 41.92) | 35.11 (-3.81, 81.73) | 42.53 (-6.20, 89.37) | -4.05 (-37.89, 29.23) | -0.78 (-34.08, 33.48) | 0.75 (-43.22, 44.57) |
| -45.37 (-98.77, 3.54) | -2.64 (-36.02, 32.71) | 4.37 (-35.59, 38.90) | -42.46 (-77.85, -9.55) | -38.95 (-82.97, 1.04) | -37.34 (-86.25, 8.80) |
| -24.13 (-74.88, 29.14) | 17.98 (-23.22, 68.83) | 25.72 (-23.78, 75.87) | -21.24 (-56.95, 15.04) | -17.93 (-60.98, 27.47) | -16.58 (-63.26, 32.09) |
| -18.08 (-86.75, 53.83) | 25.38 (-40.83, 93.64) | 31.30 (-37.53, 102.46) | -14.57 (-77.06, 46.97) | -11.33 (-77.97, 55.84) | -10.08 (-78.82, 59.02) |
| -2.04 (-62.81, 59.84) | 41.14 (-13.73, 101.38) | 48.00 (-12.56, 106.99) | 0.93 (-47.71, 50.49) | 4.35 (-37.64, 47.24) | 5.51 (-46.12, 62.29) |
| Diosmectite | 42.32 (-0.88, 90.17) | 49.22 (-1.65, 99.57) | 3.04 (-34.10, 39.16) | 6.30 (-38.01, 50.00) | 7.81 (-42.61, 58.57) |
| -42.32 (-90.17, 0.88) | Glycyrrhiza | 7.31 (-29.69, 37.57) | -39.43 (-69.27, -16.29) | -35.99 (-75.90, -1.89) | -34.27 (-78.67, 5.74) |
| -49.22 (-99.57, 1.65) | -7.31 (-37.57, 29.69) | Laser | -46.76 (-79.70, -13.87) | -43.37 (-85.77, 0.68) | -41.94 (-88.73, 7.21) |
| -3.04 (-39.16, 34.10) | 39.43 (16.29, 69.27) | 46.76 (13.87, 79.70) | Placebo | 3.16 (-22.34, 29.02) | 4.63 (-27.46, 38.14) |
| -6.30 (-50.00, 38.01) | 35.99 (1.89, 75.90) | 43.37 (-0.68, 85.77) | -3.16 (-29.02, 22.34) | Triamcinolone | 1.39 (-31.20, 36.15) |
| -7.81 (-58.57, 42.61) | 34.27 (-5.74, 78.67) | 41.94 (-7.21, 88.73) | -4.63 (-38.14, 27.46) | -1.39 (-36.15, 31.20) | Triester Glycerol Oxide |

1.2 Rank probability(Rank 1 is best, rank N is worst)

| Drug | Rank 1 | Rank 2 | Rank 3 | Rank 4 | Rank 5 | Rank 6 | Rank 7 | Rank 8 | Rank 9 | Rank 10 | Rank 11 | Rank 12 |
|---|---|---|---|---|---|---|---|---|---|---|---|---|
| Allicin | 0.02 | 0.03 | 0.04 | 0.16 | 0.29 | 0.23 | 0.08 | 0.04 | 0.04 | 0.03 | 0.03 | 0.02 |
| Aloe | 0.01 | 0.01 | 0.01 | 0.02 | 0.05 | 0.11 | 0.18 | 0.17 | 0.15 | 0.13 | 0.09 | 0.06 |
| Amlexanox | 0.24 | 0.32 | 0.27 | 0.1 | 0.03 | 0.02 | 0.01 | 0.01 | 0 | 0 | 0 | 0 |
| Berberine gelatin | 0.03 | 0.03 | 0.05 | 0.28 | 0.28 | 0.16 | 0.06 | 0.03 | 0.03 | 0.02 | 0.02 | 0.01 |
| Chitosan | 0.11 | 0.05 | 0.05 | 0.15 | 0.1 | 0.09 | 0.06 | 0.05 | 0.04 | 0.05 | 0.08 | 0.17 |
| Curcumin | 0.01 | 0.01 | 0.02 | 0.04 | 0.06 | 0.09 | 0.1 | 0.07 | 0.07 | 0.09 | 0.14 | 0.31 |
| Diosmectite | 0 | 0.01 | 0.01 | 0.02 | 0.03 | 0.05 | 0.07 | 0.08 | 0.08 | 0.12 | 0.24 | 0.29 |
| Glycyrrhiza | 0.1 | 0.28 | 0.4 | 0.14 | 0.04 | 0.02 | 0.01 | 0 | 0 | 0 | 0 | 0 |
| Laser | 0.47 | 0.26 | 0.14 | 0.06 | 0.03 | 0.01 | 0.01 | 0.01 | 0 | 0 | 0 | 0 |
| Placebo | 0 | 0 | 0 | 0 | 0 | 0.02 | 0.06 | 0.12 | 0.19 | 0.29 | 0.25 | 0.05 |
| Triamcinolone | 0 | 0 | 0.01 | 0.01 | 0.03 | 0.07 | 0.16 | 0.23 | 0.24 | 0.15 | 0.08 | 0.02 |
| Triester Glycerol Oxide | 0.01 | 0.01 | 0.01 | 0.02 | 0.06 | 0.13 | 0.21 | 0.19 | 0.15 | 0.1 | 0.08 | 0.05 |

1.3 Consistency check

| Parameter | Median (95% CI) |
|---|---|
| Random Effects Standard Deviation | 8.88 (0.88, 42.96) |

1.4 Convergence Diagnostics

| Parameter | PSRF |
|---|---|
| d.Placebo.Allicin | 1.00 |
| d.Placebo.Aloe | 1.01 |
| d.Placebo.Amlexanox | 1.00 |
| d.Placebo.Berberinegelatin | 1.00 |
| d.Placebo.Chitosan | 1.00 |
| d.Placebo.Diosmectite | 1.00 |
| d.Placebo.Glycyrrhiza | 1.00 |
| d.Placebo.Laser | 1.00 |
| d.Placebo.Triamcinolone | 1.00 |
| d.Placebo.TriesterGlycerolOxide | 1.01 |
| d.Triamcinolone.Curcumin | 1.01 |
| sd.d | 1.01 |

Number of chains : 4
Tuning iterations : 20,000
Simulation iterations : 50,000
Thinning interval : 10
Inference samples : 10,000
Variance scaling factor: 2.5

**2.Inconsistency Model**

2.1 Summary estimates

| | | | |
|---|---|---|---|
| Allicin | -14.52 (-62.92, 38.79) | 23.84 (-23.31, 76.80) | 2.89 (-48.30, 60.30) |
| 14.52 (-38.79, 62.92) | Aloe | 37.87 (-9.07, 87.91) | 17.62 (-31.40, 69.32) |
| -23.84 (-76.80, 23.31) | -37.87 (-87.91, 9.07) | Amlexanox | -20.51 (-72.43, 29.88) |
| -2.89 (-60.30, 48.30) | -17.62 (-69.32, 31.40) | 20.51 (-29.88, 72.43) | Berberine gelatin |
| 1.81 (-66.75, 79.70) | -10.56 (-79.25, 65.86) | 28.38 (-43.66, 104.36) | 7.07 (-64.79, 82.59) |
| 21.34 (-42.59, 83.72) | 8.21 (-46.95, 61.72) | 46.16 (-14.98, 106.23) | 25.54 (-35.99, 83.81) |
| 22.04 (-31.43, 72.79) | 8.36 (-43.22, 57.57) | 46.24 (-4.53, 97.81) | 26.01 (-27.89, 80.44) |
| -20.82 (-71.40, 24.25) | -34.83 (-81.42, 6.86) | 2.90 (-33.08, 36.66) | -17.47 (-65.13, 26.99) |
| -27.79 (-80.26, 21.85) | -42.59 (-88.51, 5.36) | -4.11 (-40.39, 35.84) | -24.55 (-74.80, 26.47) |
| 18.75 (-21.71, 56.13) | 4.40 (-27.39, 38.15) | 42.19 (10.29, 77.91) | 21.90 (-13.70, 60.12) |
| 15.52 (-32.61, 61.13) | 1.24 (-32.11, 34.54) | 38.90 (-3.08, 83.54) | 18.96 (-26.42, 63.12) |
| 14.29 (-39.25, 61.57) | -0.30 (-44.82, 44.46) | 37.57 (-8.46, 87.06) | 17.43 (-34.09, 66.44) |

| | | | | | | | |
|---|---|---|---|---|---|---|---|
| -1.81 (-79.70, 66.75) | -21.34 (-83.72, 42.59) | -22.04 (-72.79, 31.43) | 20.82 (-24.25, 71.40) | 27.79 (-21.85, 80.26) | -18.75 (-56.13, 21.71) | -15.52 (-61.13, 32.61) | -14.29 (-61.57, 39.25) |
| 10.56 (-65.86, 79.25) | -8.21 (-61.72, 46.95) | -8.36 (-57.57, 43.22) | 34.83 (-6.86, 81.42) | 42.59 (-5.36, 88.51) | -4.40 (-38.15, 27.39) | -1.24 (-34.54, 32.11) | 0.30 (-44.46, 44.82) |
| -28.38 (-104.36, 43.66) | -46.16 (-106.23, 14.98) | -46.24 (-97.81, 4.53) | -2.90 (-36.66, 33.08) | 4.11 (-35.84, 40.39) | -42.19 (-77.91, -10.29) | -38.90 (-83.54, 3.08) | -37.57 (-87.06, 8.46) |
| -7.07 (-82.59, 64.79) | -25.54 (-83.81, 35.99) | -26.01 (-80.44, 27.89) | 17.47 (-26.99, 65.13) | 24.55 (-26.47, 74.80) | -21.90 (-60.12, 13.70) | -18.96 (-63.12, 26.42) | -17.43 (-66.44, 34.09) |
| Chitosan | -18.52 (-92.08, 65.99) | -18.28 (-89.71, 57.87) | 25.42 (-41.81, 97.91) | 31.98 (-38.75, 107.17) | -14.66 (-76.69, 50.02) | -11.48 (-78.29, 58.89) | -10.05 (-80.46, 65.66) |
| 18.52 (-65.99, 92.08) | Curcumin | 0.31 (-62.10, 62.49) | 43.64 (-12.19, 103.03) | 50.26 (-11.97, 109.73) | 3.80 (-47.81, 53.48) | 7.18 (-36.84, 49.05) | 8.45 (-47.99, 63.86) |
| 18.28 (-57.87, 89.71) | -0.31 (-62.49, 62.10) | Diosmectite | 42.93 (-1.42, 91.64) | 50.78 (-0.41, 99.02) | 3.99 (-34.48, 40.79) | 7.33 (-38.93, 52.39) | 8.68 (-42.37, 58.83) |
| -25.42 (-97.91, 41.81) | -43.64 (-103.03, 12.19) | -42.93 (-91.64, 1.42) | Glycyrrhiza | 6.58 (-29.44, 41.29) | -39.51 (-69.01, -13.13) | -36.01 (-76.48, 0.60) | -34.38 (-81.70, 6.40) |
| -31.98 (-107.17, 38.75) | -50.26 (-109.73, 11.97) | -50.78 (-99.02, 0.41) | -6.58 (-41.29, 29.44) | Laser | -41.02 (-94.48, 23.19) | -43.58 (-87.08, 0.39) | -42.35 (-90.66, 6.73) |
| 14.66 (-50.02, 76.69) | -3.80 (-53.48, 47.81) | -3.99 (-40.79, 34.48) | 39.51 (13.13, 69.01) | 41.02 (-23.19, 94.48) | Placebo | 3.10 (-23.20, 30.04) | 4.54 (-29.74, 40.26) |
| 11.48 (-58.89, 78.29) | -7.18 (-49.05, 36.84) | -7.33 (-52.39, 38.93) | 36.01 (-0.60, 76.48) | 43.58 (-0.39, 87.08) | -3.10 (-30.04, 23.20) | Triamcinolone | 1.46 (-33.76, 35.92) |
| 10.05 (-65.66, 80.46) | -8.45 (-63.86, 47.99) | -8.68 (-58.83, 42.37) | 34.38 (-6.40, 81.70) | 42.35 (-6.73, 90.66) | -4.54 (-40.26, 29.74) | -1.46 (-35.92, 33.76) | Triester Glycerol Oxide |

## 2.2 Inconsistency Factors

| Cycle | Median (95% CI) |
|---|---|
| Glycyrrhiza, Laser, Placebo | 3.60 (-41.97, 68.36) |

## 2.3 Variance Calculation

| Parameter | Median (95% CI) |
|---|---|
| Random Effects Standard Deviation | 8.57 (0.49, 43.70) |
| Inconsistency Standard Deviation | 25.73 (1.12, 51.05) |

### 2.4 Convergence Diagnostics

| Parameter | PSRF |
|---|---|
| d.Allicin.Placebo | 1.00 |
| d.Aloe.Triamcinolone | 1.00 |
| d.Glycyrrhiza.Laser | 1.01 |
| d.Placebo.Aloe | 1.00 |
| d.Placebo.Amlexanox | 1.00 |
| d.Placebo.Berberinegelatin | 1.00 |
| d.Placebo.Chitosan | 1.04 |
| d.Placebo.Diosmectite | 1.00 |
| d.Placebo.Glycyrrhiza | 1.00 |
| d.Triamcinolone.Curcumin | 1.00 |
| d.Triamcinolone.TriesterGlycerol... | 1.00 |
| w.Glycyrrhiza.Laser.Placebo | 1.01 |
| sd.d | 1.01 |
| sd.w | 1.00 |

Number of chains : 4
Tuning iterations : 20,000
Simulation iterations : 50,000
Thinning interval : 10
Inference samples : 10,000
Variance scaling factor: 2.5

## 3. Node-splitting analysis

| Name | Direct Effect | Indirect Effect | Overall | P-Value |
|---|---|---|---|---|
| Glycyrrhiza, Laser | 9.29 (-28.30, 48.97) | -54.27 (-204.92, 99.93) | 7.31 (-29.69, 37.57) | 0.39 |

## 4. Network structure

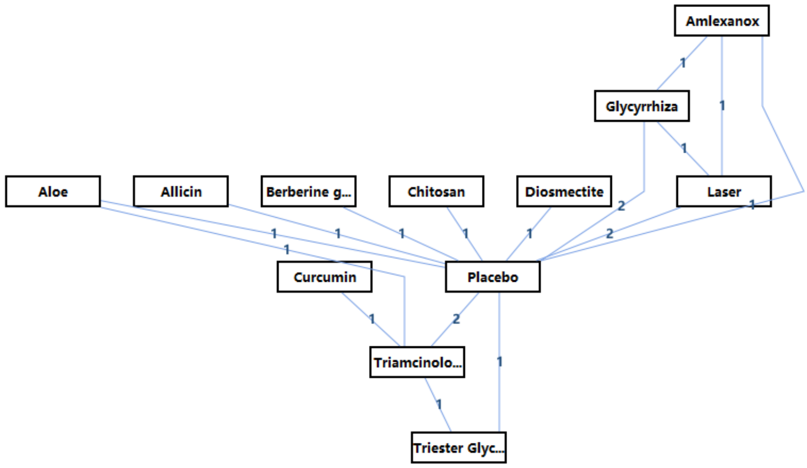


# Day 3

## 1. Consistency Model

### 1.1 Summary estimates

| | | | |
|---|---|---|---|
| Aloe | 38.19 (-85.41, 157.33) | 36.97 (-100.17, 171.75) | 64.36 (-79.42, 193.82) |
| -38.19 (-157.33, 85.41) | Amlexanox | -0.88 (-120.99, 122.12) | 25.24 (-75.82, 124.28) |
| -36.97 (-171.75, 100.17) | 0.88 (-122.12, 120.99) | Cryotherapy | 28.02 (-116.85, 159.94) |
| -64.36 (-193.82, 79.42) | -25.24 (-124.28, 75.82) | -28.02 (-159.94, 116.85) | Curcumin |
| -43.30 (-197.22, 114.54) | -5.34 (-105.16, 87.24) | -6.60 (-161.55, 148.07) | 20.01 (-125.54, 157.17) |
| -28.19 (-139.91, 88.75) | 9.91 (-89.38, 108.56) | 8.51 (-108.21, 128.42) | 36.27 (-86.66, 150.99) |
| -13.08 (-135.08, 132.49) | 26.54 (-72.57, 141.46) | 23.92 (-103.51, 170.48) | 51.46 (-54.38, 168.26) |
| -14.19 (-106.28, 80.00) | 23.84 (-52.09, 96.88) | 22.38 (-72.48, 120.71) | 49.71 (-54.05, 141.86) |
| -26.69 (-138.12, 86.20) | 11.10 (-91.32, 108.98) | 10.18 (-105.20, 130.41) | 37.40 (-84.90, 148.55) |
| -69.57 (-183.04, 57.74) | -31.33 (-102.97, 45.89) | -32.55 (-146.25, 92.68) | -5.06 (-75.40, 63.26) |
| -99.84 (-236.93, 37.79) | -61.77 (-183.72, 58.04) | -63.09 (-201.00, 73.68) | -35.93 (-177.50, 99.41) |

| | | | | | | |
|---|---|---|---|---|---|---|
| 43.30 (-114.54, 197.22) | 28.19 (-88.75, 139.91) | 13.08 (-132.49, 135.08) | 14.19 (-80.00, 106.28) | 26.69 (-86.20, 138.12) | 69.57 (-57.74, 183.04) | 99.84 (-37.79, 236.93) |
| 5.34 (-87.24, 105.16) | -9.91 (-108.56, 89.38) | -26.54 (-141.46, 72.57) | -23.84 (-96.88, 52.09) | -11.10 (-108.98, 91.32) | 31.33 (-45.89, 102.97) | 61.77 (-58.04, 183.72) |
| 6.60 (-148.07, 161.55) | -8.51 (-128.42, 108.21) | -23.92 (-170.48, 103.51) | -22.38 (-120.71, 72.48) | -10.18 (-130.41, 105.20) | 32.55 (-92.68, 146.25) | 63.09 (-73.68, 201.00) |
| -20.01 (-157.17, 125.54) | -36.27 (-150.99, 86.66) | -51.46 (-168.26, 54.38) | -49.71 (-141.86, 54.05) | -37.40 (-148.55, 84.90) | 5.06 (-63.26, 75.40) | 35.93 (-99.41, 177.50) |
| Dexamethasone | -14.88 (-153.05, 122.53) | -31.32 (-189.69, 101.88) | -28.72 (-150.52, 92.26) | -17.33 (-157.99, 121.40) | 25.63 (-100.98, 143.49) | 56.33 (-94.05, 212.52) |
| 14.88 (-122.53, 153.05) | Glycyrrhiza | -15.72 (-139.37, 90.66) | -14.01 (-81.13, 54.60) | -1.21 (-96.48, 92.59) | 41.18 (-59.82, 134.15) | 71.17 (-45.35, 191.41) |
| 31.32 (-101.88, 189.69) | 15.72 (-90.66, 139.37) | Laser | 2.30 (-82.61, 106.35) | 14.46 (-93.73, 139.12) | 57.09 (-24.93, 148.69) | 87.14 (-35.77, 230.43) |
| 28.72 (-92.26, 150.52) | 14.01 (-54.60, 81.13) | -2.30 (-106.35, 82.61) | Placebo | 12.63 (-55.50, 78.63) | 54.85 (-22.59, 119.89) | 85.02 (-10.24, 181.86) |
| 17.33 (-121.40, 157.99) | 1.21 (-92.59, 96.48) | -14.46 (-139.12, 93.73) | -12.63 (-78.63, 55.50) | Probiotics | 42.73 (-60.68, 133.84) | 72.26 (-41.47, 189.09) |
| -25.63 (-143.49, 100.98) | -41.18 (-134.15, 59.82) | -57.09 (-148.69, 24.93) | -54.85 (-119.89, 22.59) | -42.73 (-133.84, 60.68) | Triamcinolone | 30.50 (-85.10, 154.44) |
| -56.33 (-212.52, 94.05) | -71.17 (-191.41, 45.35) | -87.14 (-230.43, 35.77) | -85.02 (-181.86, 10.24) | -72.26 (-189.09, 41.47) | -30.50 (-154.44, 85.10) | Zinc |

## 1.2 Rank probability(Rank 1 is best, rank N is worst)

| Drug | Rank 1 | Rank 2 | Rank 3 | Rank 4 | Rank 5 | Rank 6 | Rank 7 | Rank 8 | Rank 9 | Rank 10 | Rank 11 |
|---|---|---|---|---|---|---|---|---|---|---|---|
| Aloe | 0.01 | 0.03 | 0.03 | 0.04 | 0.05 | 0.05 | 0.07 | 0.08 | 0.1 | 0.17 | 0.37 |
| Amlexanox | 0.01 | 0.04 | 0.07 | 0.13 | 0.19 | 0.17 | 0.13 | 0.1 | 0.08 | 0.06 | 0.02 |
| Cryotherapy | 0.06 | 0.11 | 0.08 | 0.1 | 0.1 | 0.1 | 0.1 | 0.08 | 0.08 | 0.1 | 0.09 |
| Curcumin | 0.14 | 0.19 | 0.18 | 0.13 | 0.1 | 0.07 | 0.05 | 0.04 | 0.04 | 0.04 | 0.02 |
| Dexamethasone | 0.09 | 0.11 | 0.1 | 0.12 | 0.11 | 0.1 | 0.07 | 0.06 | 0.07 | 0.08 | 0.09 |
| Glycyrrhiza | 0.02 | 0.04 | 0.06 | 0.08 | 0.11 | 0.13 | 0.14 | 0.13 | 0.11 | 0.1 | 0.06 |
| Laser | 0.01 | 0.03 | 0.04 | 0.07 | 0.07 | 0.09 | 0.1 | 0.09 | 0.11 | 0.16 | 0.24 |
| Placebo | 0 | 0 | 0 | 0.02 | 0.04 | 0.08 | 0.15 | 0.24 | 0.26 | 0.17 | 0.04 |
| Probiotics | 0.01 | 0.04 | 0.06 | 0.09 | 0.11 | 0.13 | 0.14 | 0.13 | 0.13 | 0.11 | 0.06 |
| Triamcinolone | 0.08 | 0.27 | 0.27 | 0.16 | 0.09 | 0.05 | 0.03 | 0.02 | 0.01 | 0.01 | 0 |
| Zinc | 0.57 | 0.14 | 0.09 | 0.06 | 0.04 | 0.03 | 0.02 | 0.02 | 0.01 | 0.01 | 0.01 |

1.3 Consistency check

| Parameter | Median (95% CI) |
|---|---|
| Random Effects Standard Deviation | 36.53 (16.67, 87.26) |

1.4 Convergence Diagnostics

| Parameter | PSRF |
|---|---|
| d.Amlexanox.Dexamethasone | 1.02 |
| d.Placebo.Aloe | 1.00 |
| d.Placebo.Amlexanox | 1.00 |
| d.Placebo.Cryotherapy | 1.00 |
| d.Placebo.Glycyrrhiza | 1.00 |
| d.Placebo.Laser | 1.00 |
| d.Placebo.Probiotics | 1.00 |
| d.Placebo.Triamcinolone | 1.00 |
| d.Placebo.Zinc | 1.00 |
| d.Triamcinolone.Curcumin | 1.00 |
| sd.d | 1.00 |

Number of chains : 4
Tuning iterations : 20,000
Simulation iterations : 50,000
Thinning interval : 10
Inference samples : 10,000
Variance scaling factor: 2.5

## 2.Inconsistency Model

2.1 Summary estimates

| | | | | | | | |
|---|---|---|---|---|---|---|---|
| Aloe | 19.11 (-35.30, 91.12) | 39.55 (-33.87, 107.84) | 29.68 (-40.14, 117.89) | 20.20 (-51.27, 106.48) | 29.39 (-31.65, 86.17) | -3.75 (-82.85, 81.56) | 14.37 (-34.17, 61.82) |
| -19.11 (-91.12, 35.30) | Amlexanox | 17.96 (-59.29, 78.09) | 10.16 (-43.43, 63.44) | -0.31 (-48.39, 49.87) | 10.37 (-52.03, 55.44) | -24.44 (-90.95, 38.32) | -4.08 (-54.95, 28.91) |
| -39.55 (-107.84, 33.87) | -17.96 (-78.09, 59.29) | Cryotherapy | -7.56 (-81.75, 85.25) | -17.42 (-93.31, 73.94) | -8.81 (-71.59, 55.86) | -41.25 (-124.12, 49.05) | -23.64 (-75.70, 31.76) |
| -29.68 (-117.89, 40.14) | -10.16 (-63.44, 43.43) | 7.56 (-85.25, 81.75) | Curcumin | -8.80 (-84.11, 62.07) | 0.56 (-81.18, 63.09) | -34.33 (-103.13, 30.06) | -14.88 (-88.12, 40.82) |
| -20.20 (-106.48, 51.27) | 0.31 (-49.87, 48.39) | 17.42 (-73.94, 93.31) | 8.80 (-62.07, 84.11) | Dexamethasone | 10.19 (-74.14, 73.76) | -25.44 (-107.76, 52.52) | -4.97 (-77.45, 51.45) |
| -29.39 (-86.17, 31.65) | -10.37 (-55.44, 52.03) | 8.81 (-55.86, 71.59) | -0.56 (-63.09, 81.18) | -10.19 (-73.76, 74.14) | Glycyrrhiza | -33.85 (-105.55, 45.70) | -15.31 (-48.43, 21.18) |
| 3.75 (-81.56, 82.85) | 24.44 (-38.32, 90.95) | 41.25 (-49.05, 124.12) | 34.33 (-30.06, 103.13) | 25.44 (-52.52, 107.76) | 33.85 (-45.70, 105.55) | Laser | 46.21 (-46.38, 140.89) |
| -14.37 (-61.82, 34.17) | 4.08 (-28.91, 54.95) | 23.64 (-31.76, 75.70) | 14.88 (-40.82, 88.12) | 4.97 (-51.45, 77.45) | 15.31 (-21.18, 48.43) | -46.21 (-140.89, 46.38) | Placebo |
| -28.58 (-84.35, 30.06) | -9.20 (-54.16, 52.98) | 10.11 (-53.25, 71.11) | 0.92 (-61.16, 81.71) | -8.59 (-72.27, 71.65) | 0.86 (-46.33, 47.52) | -32.63 (-103.51, 45.91) | -13.96 (-46.30, 18.86) |
| -31.19 (-113.33, 25.73) | -11.65 (-58.33, 22.23) | 5.31 (-85.60, 68.04) | -2.72 (-43.98, 30.16) | -12.04 (-81.30, 45.68) | -1.54 (-78.01, 45.30) | -37.45 (-98.37, 14.71) | -94.02 (-133.75, -30.18) |
| -100.09 (-166.94, -34.05) | -80.12 (-136.76, -8.59) | -61.70 (-136.33, 8.67) | -70.77 (-143.61, 16.48) | -78.98 (-152.83, 7.37) | -70.65 (-132.69, -12.11) | -105.03 (-186.21, -19.66) | -85.26 (-134.62, -36.60) |

| | | |
|---|---|---|
| 28.58 (-30.06, 84.35) | 31.19 (-25.73, 113.33) | 100.09 (34.05, 166.94) |
| 9.20 (-52.98, 54.16) | 11.65 (-22.23, 58.33) | 80.12 (8.59, 136.76) |
| -10.11 (-71.11, 53.25) | -5.31 (-68.04, 85.60) | 61.70 (-8.67, 136.33) |
| -0.92 (-81.71, 61.16) | 2.72 (-30.16, 43.98) | 70.77 (-16.48, 143.61) |
| 8.59 (-71.65, 72.27) | 12.04 (-45.68, 81.30) | 78.98 (-7.37, 152.83) |
| -0.86 (-47.52, 46.33) | 1.54 (-45.30, 78.01) | 70.65 (12.11, 132.69) |
| 32.63 (-45.91, 103.51) | 37.45 (-14.71, 98.37) | 105.03 (19.66, 186.21) |
| 13.96 (-18.86, 46.30) | 94.02 (30.18, 133.75) | 85.26 (36.60, 134.62) |
| Probiotics | 2.78 (-44.62, 78.82) | 71.86 (12.05, 132.34) |
| -2.78 (-78.82, 44.62) | Triamcinolone | 67.76 (-15.47, 130.03) |
| -71.86 (-132.34, -12.05) | -67.76 (-130.03, 15.47) | Zinc |

2.2 Inconsistency Factors

| Cycle | Median (95% CI) |
|---|---|
| Amlexanox, Laser, Placebo, Triamcinolone | -27.88 (-122.82, 59.06) |
| Amlexanox, Placebo, Triamcinolone | 76.53 (-8.08, 123.31) |

2.3 Variance Calculation

| Parameter | Median (95% CI) |
|---|---|
| Random Effects Standard Deviation | 11.67 (0.98, 55.76) |
| Inconsistency Standard Deviation | 67.12 (16.68, 100.02) |

2.4 Convergence Diagnostics

| Parameter | PSRF |
|---|---|
| d.Aloe.Placebo | 1.00 |
| d.Amlexanox.Dexamethasone | 1.19 |
| d.Amlexanox.Triamcinolone | 1.01 |
| d.Placebo.Amlexanox | 1.00 |
| d.Placebo.Cryotherapy | 1.01 |
| d.Placebo.Glycyrrhiza | 1.00 |
| d.Placebo.Probiotics | 1.01 |
| d.Placebo.Zinc | 1.01 |
| d.Triamcinolone.Curcumin | 1.01 |
| d.Triamcinolone.Laser | 1.00 |
| w.Amlexanox.Placebo.Laser.Tria... | 1.00 |
| w.Amlexanox.Placebo.Triamcinol... | 1.00 |
| sd.d | 1.01 |
| sd.w | 1.00 |

| | | |
|---|---|---|
| Number of chains | : | 4 |
| Tuning iterations | : | 20,000 |
| Simulation iterations | : | 50,000 |
| Thinning interval | : | 10 |
| Inference samples | : | 10,000 |
| Variance scaling factor: | | 2.5 |

## 3. Node-splitting analysis

| Name | Direct Effect | Indirect Effect | Overall | P-Value |
|---|---|---|---|---|
| Amlexanox, Placebo | -3.24 (-73.90, 61.95) | -80.03 (-150.79, 36.17) | -23.84 (-96.88, 52.09) | 0.17 |
| Amlexanox, Triamcinolone | 10.14 (-53.70, 74.47) | 89.12 (-22.73, 152.94) | 31.33 (-45.89, 102.97) | 0.14 |
| Laser, Placebo | 82.29 (-60.65, 226.22) | -38.33 (-165.77, 80.37) | 2.30 (-82.61, 106.35) | 0.18 |
| Laser, Triamcinolone | 29.42 (-68.30, 126.55) | 150.30 (-8.90, 309.08) | 57.09 (-24.93, 148.69) | 0.19 |
| Placebo, Triamcinolone | 100.84 (55.85, 147.26) | 11.02 (-53.67, 49.37) | 54.85 (-22.59, 119.89) | 0.01 |

## 4. Network structure

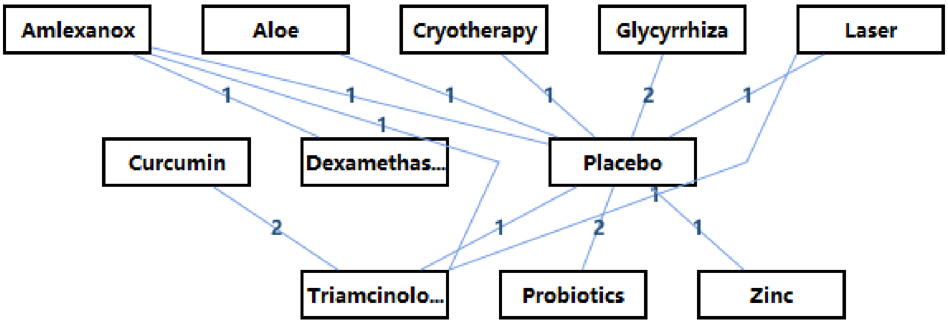


**Day 4**

## 1. Consistency Model

### 1.1 Summary estimates

| | | | | | | | | |
|---|---|---|---|---|---|---|---|---|
| Allicin | 14.63 (-108.29, 133.33) | 25.41 (-115.31, 172.60) | -5.19 (-151.54, 147.72) | -2.32 (-165.35, 158.56) | -13.68 (-145.68, 114.23) | -18.56 (-172.65, 129.70) | 46.95 (-94.16, 197.67) | 19.40 (-114.60, 154.81) |
| -14.63 (-133.33, 108.29) | Amlexanox | 11.37 (-103.17, 127.86) | -19.08 (-136.15, 103.57) | -16.99 (-148.05, 113.56) | -27.47 (-124.29, 64.81) | -32.59 (-154.28, 86.84) | 33.47 (-78.72, 151.35) | 5.60 (-89.76, 101.33) |
| -25.41 (-172.60, 115.31) | -11.37 (-127.86, 103.17) | Benzydamine | -29.89 (-177.77, 117.81) | -28.69 (-187.29, 123.14) | -38.73 (-159.67, 69.28) | -43.70 (-194.77, 95.94) | 22.18 (-86.46, 131.08) | -5.81 (-133.09, 116.87) |
| 5.19 (-147.72, 151.54) | 19.08 (-103.57, 136.15) | 29.89 (-117.81, 177.77) | Berberine gelatin | 2.21 (-157.49, 158.60) | -8.41 (-146.26, 117.13) | -14.45 (-169.09, 138.11) | 52.02 (-94.71, 202.76) | 24.27 (-107.59, 156.61) |
| 2.32 (-158.56, 165.35) | 16.99 (-113.56, 148.05) | 28.69 (-123.14, 187.29) | -2.21 (-158.60, 157.49) | Chitosan | -11.65 (-157.21, 132.40) | -16.01 (-180.53, 146.44) | 51.18 (-105.34, 210.60) | 22.31 (-120.39, 165.57) |
| 13.68 (-114.23, 145.68) | 27.47 (-64.81, 124.29) | 38.73 (-69.28, 159.67) | 8.41 (-117.13, 146.26) | 11.65 (-132.40, 157.21) | Curcumin | -5.64 (-133.96, 125.63) | 61.30 (-47.45, 179.14) | 33.16 (-74.74, 144.68) |
| 18.56 (-129.70, 172.65) | 32.59 (-86.84, 154.28) | 43.70 (-95.94, 194.77) | 14.45 (-138.11, 169.09) | 16.01 (-146.44, 180.53) | 5.64 (-125.63, 133.96) | Diosmectite | 66.63 (-79.29, 217.37) | 38.61 (-92.49, 170.16) |
| -46.95 (-197.67, 94.16) | -33.47 (-151.35, 78.72) | -22.18 (-131.08, 86.46) | -52.02 (-202.76, 94.71) | -51.18 (-210.60, 105.34) | -61.30 (-179.14, 47.45) | -66.63 (-217.37, 79.29) | Doxycycline | -27.81 (-156.47, 94.76) |
| -19.40 (-154.81, 114.60) | -5.60 (-101.33, 89.76) | 5.81 (-116.87, 133.09) | -24.27 (-156.61, 107.59) | -22.31 (-165.57, 120.39) | -33.16 (-144.68, 74.74) | -38.61 (-170.16, 92.49) | 27.81 (-94.76, 156.47) | Laser |
| 19.93 (-85.93, 130.86) | 34.68 (-20.72, 88.08) | 45.62 (-49.84, 152.46) | 15.88 (-85.74, 124.77) | 17.90 (-103.62, 138.48) | 7.03 (-70.13, 81.84) | 2.30 (-104.81, 107.15) | 67.70 (-28.64, 171.38) | 40.11 (-36.50, 118.89) |
| -47.37 (-228.94, 130.28) | -32.95 (-192.07, 118.71) | -22.39 (-125.66, 85.11) | -51.89 (-233.57, 128.13) | -49.98 (-243.51, 135.97) | -60.34 (-220.75, 90.50) | -66.27 (-248.85, 114.44) | -0.00 (-152.86, 151.93) | -27.13 (-193.59, 135.33) |
| -30.78 (-157.23, 93.17) | -16.03 (-105.31, 67.90) | -5.15 (-102.31, 97.84) | -34.70 (-157.92, 93.75) | -32.82 (-172.74, 105.59) | -43.80 (-116.47, 20.79) | -49.76 (-178.77, 74.40) | 17.51 (-80.88, 117.80) | -10.94 (-112.85, 91.77) |
| -14.09 (-161.01, 132.24) | 0.09 (-115.99, 109.55) | 10.86 (-118.03, 143.74) | -18.10 (-163.00, 127.11) | -16.32 (-173.34, 134.15) | -27.36 (-144.31, 83.50) | -33.24 (-182.47, 113.57) | 33.89 (-98.11, 168.15) | 5.16 (-122.30, 128.27) |

| | | | |
|---|---|---|---|
| -19.93 (-130.86, 85.93) | 47.37 (-130.28, 228.94) | 30.78 (-93.17, 157.23) | 14.09 (-132.24, 161.01) |
| -34.68 (-88.08, 20.72) | 32.95 (-118.71, 192.07) | 16.03 (-67.90, 105.31) | -0.09 (-109.55, 115.99) |
| -45.62 (-152.46, 49.84) | 22.39 (-85.11, 125.66) | 5.15 (-97.84, 102.31) | -10.86 (-143.74, 118.03) |
| -15.88 (-124.77, 85.74) | 51.89 (-128.13, 233.57) | 34.70 (-93.75, 157.92) | 18.10 (-127.11, 163.00) |
| -17.90 (-138.48, 103.62) | 49.98 (-135.97, 243.51) | 32.82 (-105.59, 172.74) | 16.32 (-134.15, 173.34) |
| -7.03 (-81.84, 70.13) | 60.34 (-90.50, 220.75) | 43.80 (-20.79, 116.47) | 27.36 (-83.50, 144.31) |
| -2.30 (-107.15, 104.81) | 66.27 (-114.44, 248.85) | 49.76 (-74.40, 178.77) | 33.24 (-113.57, 182.47) |
| -67.70 (-171.38, 28.64) | 0.00 (-151.93, 152.86) | -17.51 (-117.80, 80.88) | -33.89 (-168.15, 98.11) |
| -40.11 (-118.89, 36.50) | 27.13 (-135.33, 193.59) | 10.94 (-91.77, 112.85) | -5.16 (-128.27, 122.30) |
| Placebo | 67.39 (-74.27, 214.42) | 51.01 (-12.04, 118.39) | 34.50 (-60.31, 136.84) |
| -67.39 (-214.42, 74.27) | Quercetin | -17.05 (-164.83, 127.47) | -33.39 (-203.08, 134.07) |
| -51.01 (-118.39, 12.04) | 17.05 (-127.47, 164.83) | Triamcinolone | -15.83 (-115.14, 81.26) |
| -34.50 (-136.84, 60.31) | 33.39 (-134.07, 203.08) | 15.83 (-81.26, 115.14) | Triester Glycerol Oxide |

## 1.2 Rank probability(Rank 1 is best, rank N is worst)

| Drug | Rank 1 | Rank 2 | Rank 3 | Rank 4 | Rank 5 | Rank 6 | Rank 7 | Rank 8 | Rank 9 | Rank 10 | Rank 11 | Rank 12 | Rank 13 |
|---|---|---|---|---|---|---|---|---|---|---|---|---|---|
| Allicin | 0.06 | 0.06 | 0.06 | 0.06 | 0.07 | 0.08 | 0.07 | 0.08 | 0.08 | 0.08 | 0.08 | 0.1 | 0.12 |
| Amlexanox | 0.03 | 0.05 | 0.08 | 0.1 | 0.12 | 0.13 | 0.14 | 0.13 | 0.09 | 0.06 | 0.04 | 0.03 | 0.01 |
| Benzydamine | 0.04 | 0.13 | 0.13 | 0.12 | 0.11 | 0.1 | 0.08 | 0.08 | 0.06 | 0.05 | 0.05 | 0.04 | 0.02 |
| Berberine gelatin | 0.05 | 0.05 | 0.05 | 0.06 | 0.06 | 0.07 | 0.07 | 0.08 | 0.08 | 0.09 | 0.09 | 0.12 | 0.14 |
| Chitosan | 0.08 | 0.07 | 0.06 | 0.06 | 0.06 | 0.06 | 0.06 | 0.07 | 0.07 | 0.06 | 0.08 | 0.1 | 0.18 |
| Curcumin | 0 | 0.01 | 0.02 | 0.03 | 0.05 | 0.07 | 0.08 | 0.1 | 0.12 | 0.12 | 0.14 | 0.14 | 0.12 |
| Diosmectite | 0.03 | 0.04 | 0.04 | 0.04 | 0.05 | 0.05 | 0.06 | 0.07 | 0.08 | 0.08 | 0.1 | 0.14 | 0.23 |
| Doxycycline | 0.23 | 0.18 | 0.14 | 0.1 | 0.08 | 0.07 | 0.06 | 0.04 | 0.03 | 0.02 | 0.02 | 0.02 | 0.01 |
| Laser | 0.07 | 0.09 | 0.1 | 0.1 | 0.1 | 0.1 | 0.1 | 0.09 | 0.08 | 0.06 | 0.04 | 0.04 | 0.02 |
| Placebo | 0 | 0 | 0 | 0 | 0.01 | 0.01 | 0.04 | 0.08 | 0.15 | 0.24 | 0.25 | 0.17 | 0.05 |
| Quercetin | 0.31 | 0.14 | 0.09 | 0.07 | 0.06 | 0.05 | 0.05 | 0.04 | 0.04 | 0.04 | 0.03 | 0.04 | 0.04 |
| Triamcinolone | 0.05 | 0.1 | 0.15 | 0.17 | 0.16 | 0.12 | 0.1 | 0.07 | 0.04 | 0.02 | 0.01 | 0.01 | 0 |
| Triester Glycerol Oxide | 0.07 | 0.08 | 0.08 | 0.09 | 0.09 | 0.09 | 0.09 | 0.08 | 0.08 | 0.07 | 0.06 | 0.06 | 0.05 |

## 1.3 Consistency check

| Parameter | Median (95% CI) |
|---|---|
| Random Effects Standard Deviation | 46.52 (26.29, 91.77) |

## 1.4 Convergence Diagnostics

| Parameter | PSRF |
|---|---|
| d.Benzydamine.Doxycycline | 1.00 |
| d.Benzydamine.Placebo | 1.00 |
| d.Benzydamine.Quercetin | 1.00 |
| d.Benzydamine.Triamcinolone | 1.00 |
| d.Placebo.Allicin | 1.00 |
| d.Placebo.Amlexanox | 1.00 |
| d.Placebo.Berberinegelatin | 1.00 |
| d.Placebo.Chitosan | 1.00 |
| d.Placebo.Curcumin | 1.00 |
| d.Placebo.Diosmectite | 1.00 |
| d.Placebo.Laser | 1.00 |
| d.Placebo.TriesterGlycerolOxide | 1.00 |
| sd.d | 1.00 |

Number of chains : 4
Tuning iterations : 20,000
Simulation iterations : 50,000
Thinning interval : 10
Inference samples : 10,000
Variance scaling factor: 2.5

# 2.Inconsistency Model

## 2.1 Summary estimates

| | | | | | | | |
|---|---|---|---|---|---|---|---|
| Allicin | 14.24 (-111.41, 137.57) | 25.03 (-128.28, 178.16) | -4.52 (-159.50, 153.10) | -1.63 (-168.95, 162.59) | -15.19 (-156.57, 127.83) | -19.62 (-174.90, 137.87) | 48.33 (-98.34, 201.34) |
| -14.24 (-137.57, 111.41) | Amlexanox | 10.97 (-107.55, 126.90) | -18.73 (-140.09, 102.16) | -15.60 (-155.00, 116.29) | -28.96 (-136.33, 75.92) | -34.64 (-156.83, 88.02) | 34.12 (-82.90, 148.91) |
| -25.03 (-178.16, 128.28) | -10.97 (-126.90, 107.55) | Benzydamine | -29.56 (-179.21, 124.03) | -27.03 (-189.52, 136.25) | -39.58 (-161.29, 83.01) | -43.31 (-197.65, 101.71) | 23.50 (-87.26, 139.72) |
| 4.52 (-153.10, 159.50) | 18.73 (-102.16, 140.09) | 29.56 (-124.03, 179.21) | Berberine gelatin | 3.33 (-162.21, 167.87) | -10.11 (-154.66, 129.74) | -15.20 (-170.81, 139.97) | 53.00 (-95.12, 204.81) |
| 1.63 (-162.59, 168.95) | 15.60 (-116.29, 155.00) | 27.03 (-136.25, 189.52) | -3.33 (-167.87, 162.21) | Chitosan | -13.82 (-161.04, 140.34) | -17.45 (-181.10, 147.32) | 50.71 (-112.66, 218.39) |
| 15.19 (-127.83, 156.57) | 28.96 (-75.92, 136.33) | 39.58 (-83.01, 161.29) | 10.11 (-129.74, 154.66) | 13.82 (-140.34, 161.04) | Curcumin | -5.14 (-144.77, 136.31) | 62.98 (-56.74, 183.55) |
| 19.62 (-137.87, 174.90) | 34.64 (-88.02, 156.83) | 43.31 (-101.71, 197.65) | 15.20 (-139.97, 170.81) | 17.45 (-147.32, 181.10) | 5.14 (-136.31, 144.77) | Diosmectite | 67.13 (-80.15, 221.72) |
| -48.33 (-201.34, 98.34) | -34.12 (-148.91, 82.90) | -23.50 (-139.72, 87.26) | -53.00 (-204.81, 95.12) | -50.71 (-218.39, 112.66) | -62.98 (-183.55, 56.74) | -67.13 (-221.72, 80.15) | Doxycycline |
| -18.75 (-157.93, 115.94) | -4.76 (-101.83, 91.04) | 6.07 (-126.36, 134.89) | -22.56 (-160.81, 110.84) | -20.29 (-176.66, 125.29) | -33.91 (-156.94, 81.43) | -38.10 (-172.96, 98.67) | 29.21 (-100.74, 159.67) |
| 20.59 (-91.01, 133.50) | 35.19 (-19.92, 89.70) | 46.12 (-57.92, 149.35) | 17.18 (-92.99, 125.24) | 19.66 (-106.58, 142.22) | 7.88 (-80.06, 92.36) | 1.32 (-108.84, 112.57) | 68.89 (-32.28, 175.38) |
| -45.94 (-232.01, 142.21) | -32.22 (-186.67, 128.75) | -21.57 (-131.68, 86.60) | -51.58 (-236.70, 139.03) | -48.24 (-247.17, 143.13) | -61.42 (-225.25, 98.20) | -65.92 (-253.94, 115.92) | 1.57 (-150.59, 162.57) |
| -30.01 (-163.01, 98.81) | -16.42 (-108.37, 71.32) | -6.08 (-108.39, 97.44) | -35.25 (-165.69, 95.16) | -32.02 (-180.07, 108.85) | -45.35 (-118.53, 24.07) | -50.31 (-182.54, 78.40) | 18.01 (-82.84, 118.08) |
| -14.38 (-168.28, 135.69) | -0.41 (-115.64, 116.18) | 10.39 (-126.56, 144.52) | -19.91 (-165.20, 127.10) | -16.84 (-174.52, 141.34) | -28.73 (-153.31, 86.54) | -34.07 (-184.25, 113.60) | 34.27 (-99.18, 167.25) |

| | | | | |
|---|---|---|---|---|
| 18.75 (-115.94, 157.93) | -20.59 (-133.50, 91.01) | 45.94 (-142.21, 232.01) | 30.01 (-98.81, 163.01) | 14.38 (-135.69, 168.28) |
| 4.76 (-91.04, 101.83) | -35.19 (-89.70, 19.92) | 32.22 (-128.75, 186.67) | 16.42 (-71.32, 108.37) | 0.41 (-116.18, 115.64) |
| -6.07 (-134.89, 126.36) | -46.12 (-149.35, 57.92) | 21.57 (-86.60, 131.68) | 6.08 (-97.44, 108.39) | -10.39 (-144.52, 126.56) |
| 22.56 (-110.84, 160.81) | -17.18 (-125.24, 92.99) | 51.58 (-139.03, 236.70) | 35.25 (-95.16, 165.69) | 19.91 (-127.10, 165.20) |
| 20.29 (-125.29, 176.66) | -19.66 (-142.22, 106.58) | 48.24 (-143.13, 247.17) | 32.02 (-108.85, 180.07) | 16.84 (-141.34, 174.52) |
| 33.91 (-81.43, 156.94) | -7.88 (-92.36, 80.06) | 61.42 (-98.20, 225.25) | 45.35 (-24.07, 118.53) | 28.73 (-86.54, 153.31) |
| 38.10 (-98.67, 172.96) | -1.32 (-112.57, 108.84) | 65.92 (-115.92, 253.94) | 50.31 (-78.40, 182.54) | 34.07 (-113.60, 184.25) |
| -29.21 (-159.67, 100.74) | -68.89 (-175.38, 32.28) | -1.57 (-162.57, 150.59) | -18.01 (-118.08, 82.84) | -34.27 (-167.25, 99.18) |
| Laser | -40.13 (-121.23, 37.62) | 28.06 (-143.14, 194.84) | 11.78 (-94.73, 119.10) | -4.63 (-133.32, 124.61) |
| 40.13 (-37.62, 121.23) | Placebo | 67.43 (-82.80, 215.71) | 51.77 (-16.77, 125.45) | 35.39 (-65.20, 138.28) |
| -28.06 (-194.84, 143.14) | -67.43 (-215.71, 82.80) | Quercetin | -15.38 (-161.40, 132.40) | -32.23 (-203.02, 143.53) |
| -11.78 (-119.10, 94.73) | -51.77 (-125.45, 16.77) | 15.38 (-132.40, 161.40) | Triamcinolone | -16.43 (-118.37, 84.08) |
| 4.63 (-124.61, 133.32) | -35.39 (-138.28, 65.20) | 32.23 (-143.53, 203.02) | 16.43 (-84.08, 118.37) | Triester Glycerol Oxide |

## 2.2 Inconsistency Factors

| Cycle | Median (95% CI) |
|---|---|
| Curcumin, Placebo, Triamcinolone | 0.78 (-85.57, 87.20) |

## 2.3 Variance Calculation

| Parameter | Median (95% CI) |
|---|---|
| Random Effects Standard Deviation | 48.70 (27.52, 92.50) |
| Inconsistency Standard Deviation | 45.97 (2.49, 106.18) |

## 2.4 Convergence Diagnostics

| Parameter | PSRF |
|---|---|
| d.Allicin.Placebo | 1.00 |
| d.Benzydamine.Doxycycline | 1.00 |
| d.Benzydamine.Quercetin | 1.00 |
| d.Benzydamine.Triamcinolone | 1.00 |
| d.Placebo.Amlexanox | 1.00 |
| d.Placebo.Benzydamine | 1.00 |
| d.Placebo.Berberinegelatin | 1.00 |
| d.Placebo.Chitosan | 1.00 |
| d.Placebo.Diosmectite | 1.00 |
| d.Placebo.Laser | 1.00 |
| d.Triamcinolone.Curcumin | 1.00 |
| d.Triamcinolone.TriesterGlycerol... | 1.00 |
| w.Curcumin.Placebo.Triamcinolo... | 1.00 |
| sd.d | 1.00 |
| sd.w | 1.00 |

| | | |
|---|---|---|
| Number of chains | : | 4 |
| Tuning iterations | : | 20,000 |
| Simulation iterations | : | 50,000 |
| Thinning interval | : | 10 |
| Inference samples | : | 10,000 |
| Variance scaling factor: | | 2.5 |

## 3. Node-splitting analysis

| Name | Direct Effect | Indirect Effect | Overall | P-Value |
|---|---|---|---|---|
| Benzydamine, Placebo | -72.90 (-176.97, 26.31) | 10.29 (-114.57, 130.70) | -45.62 (-152.46, 49.84) | 0.23 |
| Benzydamine, Triamcinolone | 31.20 (-69.80, 130.93) | -51.46 (-176.19, 82.66) | 5.15 (-97.84, 102.31) | 0.27 |
| Curcumin, Placebo | -10.02 (-130.15, 112.82) | -5.08 (-120.90, 122.67) | -7.03 (-81.84, 70.13) | 0.94 |
| Curcumin, Triamcinolone | 46.37 (-36.96, 135.10) | 41.15 (-103.00, 185.63) | 43.80 (-20.79, 116.47) | 0.95 |
| Placebo, Triamcinolone | 51.33 (-31.39, 135.71) | 56.45 (-90.55, 210.36) | 51.01 (-12.04, 118.39) | 0.94 |

## 4. Network structure

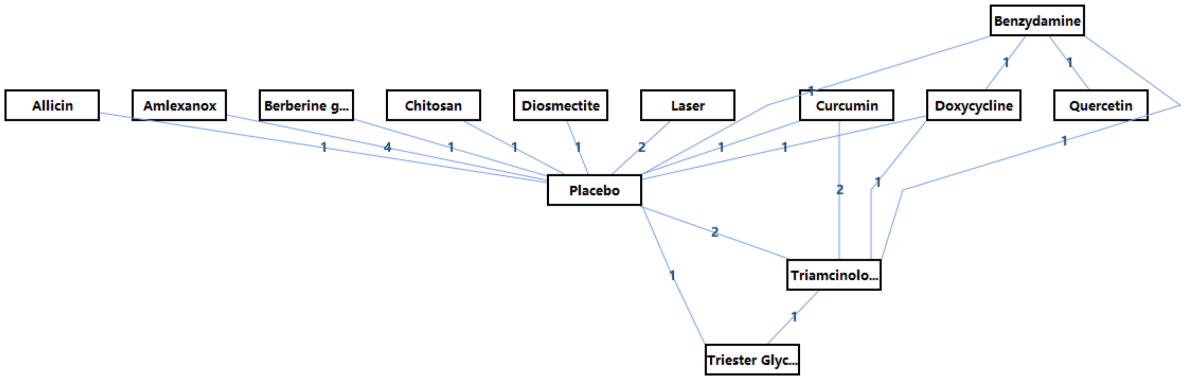


# Day 5

## 1. Consistency Model

### 1.1 Summary estimates

| | | | | | | |
|---|---|---|---|---|---|---|
| Aloe | 13.05 (-64.92, 88.43) | -16.56 (-106.20, 67.88) | 21.20 (-88.41, 131.48) | 9.27 (-66.32, 84.95) | 6.15 (-77.48, 81.28) | -18.85 (-86.31, 50.32) |
| -13.05 (-88.43, 64.92) | Amlexanox | -28.84 (-102.36, 38.42) | 9.21 (-68.07, 87.94) | -3.72 (-56.13, 49.81) | -6.39 (-67.85, 45.74) | -32.02 (-74.58, 14.28) |
| 16.56 (-67.88, 106.20) | 28.84 (-38.42, 102.36) | Curcumin | 38.25 (-64.60, 148.31) | 24.91 (-47.08, 103.01) | 21.93 (-53.63, 95.21) | -2.51 (-67.22, 70.56) |
| -21.20 (-131.48, 88.41) | -9.21 (-87.94, 68.07) | -38.25 (-148.31, 64.60) | Dexamethasone | -13.04 (-107.16, 79.97) | -15.79 (-116.22, 75.37) | -41.05 (-130.22, 49.79) |
| -9.27 (-84.95, 66.32) | 3.72 (-49.81, 56.13) | -24.91 (-103.01, 47.08) | 13.04 (-79.97, 107.16) | Glycyrrhiza | -2.80 (-61.98, 48.35) | -27.79 (-64.32, 10.89) |
| -6.15 (-81.28, 77.48) | 6.39 (-45.74, 67.85) | -21.93 (-95.21, 53.63) | 15.79 (-75.37, 116.22) | 2.80 (-48.35, 61.98) | Laser | -24.96 (-70.08, 29.55) |
| 18.85 (-50.32, 86.31) | 32.02 (-14.28, 74.58) | 2.51 (-70.56, 67.22) | 41.05 (-49.79, 130.22) | 27.79 (-10.89, 64.32) | 24.96 (-29.55, 70.08) | Placebo |
| -3.45 (-81.59, 75.66) | 9.37 (-51.75, 67.45) | -19.98 (-96.65, 51.45) | 19.16 (-80.36, 115.57) | 5.47 (-52.12, 62.40) | 2.57 (-66.53, 62.69) | -22.30 (-67.26, 22.82) |
| -14.27 (-81.83, 53.69) | -1.43 (-51.32, 48.83) | -30.97 (-84.24, 17.80) | 7.69 (-84.67, 99.72) | -5.43 (-61.89, 48.05) | -8.40 (-67.32, 43.48) | -33.73 (-76.79, 11.82) |
| -24.17 (-129.59, 80.23) | -11.57 (-104.64, 77.37) | -40.97 (-150.51, 58.46) | -2.38 (-125.11, 117.78) | -15.21 (-103.74, 69.05) | -18.53 (-116.88, 67.67) | -43.36 (-120.28, 33.67) |

| | | |
|---|---|---|
| 3.45 (-75.66, 81.59) | 14.27 (-53.69, 81.83) | 24.17 (-80.23, 129.59) |
| -9.37 (-67.45, 51.75) | 1.43 (-48.83, 51.32) | 11.57 (-77.37, 104.64) |
| 19.98 (-51.45, 96.65) | 30.97 (-17.80, 84.24) | 40.97 (-58.46, 150.51) |
| -19.16 (-115.57, 80.36) | -7.69 (-99.72, 84.67) | 2.38 (-117.78, 125.11) |
| -5.47 (-62.40, 52.12) | 5.43 (-48.05, 61.89) | 15.21 (-69.05, 103.74) |
| -2.57 (-62.69, 66.53) | 8.40 (-43.48, 67.32) | 18.53 (-67.67, 116.88) |
| 22.30 (-22.82, 67.26) | 33.73 (-11.82, 76.79) | 43.36 (-33.67, 120.28) |
| Probiotics | 11.33 (-42.80, 65.10) | 21.11 (-69.51, 112.74) |
| -11.33 (-65.10, 42.80) | Triamcinolone | 10.07 (-78.37, 102.61) |
| -21.11 (-112.74, 69.51) | -10.07 (-102.61, 78.37) | Zinc |

1.2 Rank probability(Rank 1 is best, rank N is worst)

| Drug | Rank 1 | Rank 2 | Rank 3 | Rank 4 | Rank 5 | Rank 6 | Rank 7 | Rank 8 | Rank 9 | Rank 10 |
|---|---|---|---|---|---|---|---|---|---|---|
| Aloe | 0.08 | 0.09 | 0.08 | 0.08 | 0.09 | 0.09 | 0.11 | 0.12 | 0.13 | 0.13 |
| Amlexanox | 0.04 | 0.14 | 0.17 | 0.17 | 0.16 | 0.13 | 0.1 | 0.06 | 0.03 | 0.01 |
| Curcumin | 0.02 | 0.03 | 0.04 | 0.05 | 0.06 | 0.08 | 0.1 | 0.12 | 0.17 | 0.33 |
| Dexamethasone | 0.31 | 0.15 | 0.09 | 0.07 | 0.06 | 0.06 | 0.06 | 0.06 | 0.06 | 0.09 |
| Glycyrrhiza | 0.05 | 0.11 | 0.13 | 0.14 | 0.14 | 0.15 | 0.12 | 0.09 | 0.05 | 0.01 |
| Laser | 0.06 | 0.1 | 0.11 | 0.12 | 0.12 | 0.13 | 0.12 | 0.11 | 0.08 | 0.05 |
| Placebo | 0 | 0 | 0 | 0.01 | 0.03 | 0.06 | 0.11 | 0.21 | 0.31 | 0.27 |
| Probiotics | 0.04 | 0.08 | 0.1 | 0.11 | 0.12 | 0.13 | 0.14 | 0.13 | 0.09 | 0.05 |
| Triamcinolone | 0.07 | 0.15 | 0.18 | 0.17 | 0.16 | 0.12 | 0.08 | 0.04 | 0.02 | 0 |
| Zinc | 0.33 | 0.15 | 0.09 | 0.07 | 0.06 | 0.06 | 0.06 | 0.06 | 0.05 | 0.06 |

1.3 Consistency check

| Parameter | Median (95% CI) |
|---|---|
| Random Effects Standard Deviation | 33.43 (19.44, 62.54) |

1.4 Convergence Diagnostics

| Parameter | PSRF |
|---|---|
| d.Amlexanox.Dexamethasone | 1.00 |
| d.Placebo.Aloe | 1.00 |
| d.Placebo.Amlexanox | 1.00 |
| d.Placebo.Glycyrrhiza | 1.00 |
| d.Placebo.Laser | 1.00 |
| d.Placebo.Probiotics | 1.00 |
| d.Placebo.Triamcinolone | 1.00 |
| d.Placebo.Zinc | 1.00 |
| d.Triamcinolone.Curcumin | 1.00 |
| sd.d | 1.00 |

Number of chains : 4
Tuning iterations : 20,000
Simulation iterations : 50,000
Thinning interval : 10
Inference samples : 10,000
Variance scaling factor: 2.5

# 2.Inconsistency Model

## 2.1 Summary estimates

| | | | | | | | | | |
|---|---|---|---|---|---|---|---|---|---|
| Aloe | 3.32 (-75.78, 83.10) | -16.74 (-110.99, 70.28) | 12.36 (-93.42, 118.62) | -13.83 (-101.68, 71.89) | -2.37 (-88.08, 76.10) | -18.45 (-83.23, 46.43) | 13.89 (-76.28, 103.91) | 14.63 (-49.73, 78.68) | 24.30 (-73.32, 118.91) |
| -3.32 (-83.10, 75.78) | Amlexanox | -21.39 (-100.44, 54.75) | 9.10 (-61.95, 81.08) | -17.91 (-70.75, 38.25) | -5.56 (-63.69, 45.34) | -22.45 (-69.31, 29.32) | 9.36 (-65.45, 90.23) | 7.35 (-47.31, 62.73) | 21.37 (-66.40, 109.00) |
| 16.74 (-70.28, 110.99) | 21.39 (-54.75, 100.44) | Curcumin | 29.82 (-72.10, 138.02) | 3.30 (-79.96, 91.75) | 14.35 (-59.68, 89.67) | -1.53 (-73.42, 75.08) | 31.04 (-42.77, 109.29) | 29.64 (-15.53, 80.58) | 41.42 (-60.57, 146.89) |
| -12.36 (-118.62, 93.42) | -9.10 (-81.08, 61.95) | -29.82 (-138.02, 72.10) | Dexamethasone | -27.22 (-115.64, 65.04) | -14.61 (-109.66, 72.74) | -30.77 (-118.32, 57.33) | 1.36 (-104.75, 106.46) | 0.06 (-93.20, 93.44) | 12.84 (-100.78, 122.83) |
| 13.83 (-71.89, 101.68) | 17.91 (-38.25, 70.75) | -3.30 (-91.75, 79.96) | 27.22 (-65.04, 115.64) | Glycyrrhiza | 12.31 (-53.09, 64.89) | -32.68 (-65.45, 3.49) | 26.90 (-54.85, 116.96) | 26.17 (-42.56, 96.38) | 38.66 (-54.81, 131.27) |
| 2.37 (-76.10, 88.08) | 5.56 (-45.34, 63.69) | -14.35 (-89.67, 59.68) | 14.61 (-72.74, 109.66) | -12.31 (-64.89, 53.09) | Laser | 4.41 (-63.29, 96.57) | 16.34 (-56.80, 96.15) | 15.14 (-39.34, 75.07) | 26.82 (-61.57, 121.17) |
| 18.45 (-46.43, 83.23) | 22.45 (-29.32, 69.31) | 1.53 (-75.08, 73.42) | 30.77 (-57.33, 118.32) | 32.68 (-3.49, 65.45) | -4.41 (-96.57, 63.29) | Placebo | 16.77 (-26.67, 63.72) | 29.87 (-72.72, 132.38) | 43.03 (-28.15, 115.81) |
| -13.89 (-103.91, 76.28) | -9.36 (-90.23, 65.45) | -31.04 (-109.29, 42.77) | -1.36 (-106.46, 104.75) | -26.90 (-116.96, 54.85) | -16.34 (-96.15, 56.80) | -16.77 (-63.72, 26.67) | Probiotics | -1.84 (-61.20, 58.73) | 9.78 (-89.72, 109.72) |
| -14.63 (-78.68, 49.73) | -7.35 (-62.73, 47.31) | -29.64 (-80.58, 15.53) | -0.06 (-93.44, 93.20) | -26.17 (-96.38, 42.56) | -15.14 (-75.07, 39.34) | -29.87 (-132.38, 72.72) | 1.84 (-58.73, 61.20) | Triamcinolone | 10.95 (-79.04, 103.77) |
| -24.30 (-118.91, 73.32) | -21.37 (-109.00, 66.40) | -41.42 (-146.89, 60.57) | -12.84 (-122.83, 100.78) | -38.66 (-131.27, 54.81) | -26.82 (-121.17, 61.57) | -43.03 (-115.81, 28.15) | -9.78 (-109.72, 89.72) | -10.95 (-103.77, 79.04) | Zinc |

2.2 Inconsistency Factors

| Cycle | Median (95% CI) |
|---|---|
| Amlexanox, Glycyrrhiza, Placebo | -24.97 (-88.02, 19.55) |
| Amlexanox, Laser, Placebo | 15.95 (-36.44, 110.28) |
| Amlexanox, Laser, Triamcinolone | 0.54 (-55.45, 64.80) |
| Amlexanox, Laser, Placebo, Triamcinolone | 1.13 (-55.54, 61.19) |
| Amlexanox, Laser, Placebo, Probiotics, Triamcinolone | 10.65 (-42.90, 89.06) |

2.3 Variance Calculation

| Parameter | Median (95% CI) |
|---|---|
| Random Effects Standard Deviation | 30.03 (14.97, 59.34) |
| Inconsistency Standard Deviation | 34.85 (3.35, 87.39) |

2.4 Convergence Diagnostics

| Parameter | PSRF |
|---|---|
| d.Aloe.Placebo | 1.00 |
| d.Amlexanox.Dexamethasone | 1.00 |
| d.Amlexanox.Glycyrrhiza | 1.00 |
| d.Glycyrrhiza.Laser | 1.00 |
| d.Laser.Triamcinolone | 1.00 |
| d.Placebo.Amlexanox | 1.00 |
| d.Placebo.Zinc | 1.00 |
| d.Triamcinolone.Curcumin | 1.00 |
| d.Triamcinolone.Probiotics | 1.00 |
| w.Amlexanox.Glycyrrhiza.Placebo | 1.00 |
| w.Amlexanox.Laser.Placebo | 1.00 |
| w.Amlexanox.Laser.Triamcinolone | 1.00 |
| w.Amlexanox.Laser.Triamcinolon... | 1.00 |
| w.Amlexanox.Laser.Triamcinolon... | 1.00 |
| sd.d | 1.00 |
| sd.w | 1.00 |

Number of chains : 4
Tuning iterations : 20,000
Simulation iterations : 50,000
Thinning interval : 10
Inference samples : 10,000
Variance scaling factor: 2.5

**3. Node-splitting analysis**

| Name | Direct Effect | Indirect Effect | Overall | P-Value |
|---|---|---|---|---|
| Amlexanox, Glycyrrhiza | -14.55 (-96.93, 66.57) | -1.23 (-69.58, 69.12) | -3.72 (-56.13, 49.81) | 0.79 |
| Amlexanox, Laser | 10.24 (-59.68, 77.49) | -56.43 (-132.79, 18.07) | -6.39 (-67.85, 45.74) | 0.17 |
| Amlexanox, Placebo | -44.02 (-98.67, 9.64) | -16.18 (-76.22, 48.61) | -32.02 (-74.58, 14.28) | 0.46 |
| Amlexanox, Triamcinolone | 8.69 (-74.93, 91.39) | -4.71 (-81.18, 69.16) | 1.43 (-48.83, 51.32) | 0.79 |
| Glycyrrhiza, Laser | 22.52 (-45.28, 91.87) | -53.33 (-132.35, 21.65) | -2.80 (-61.98, 48.35) | 0.12 |
| Laser, Placebo | -39.77 (-98.34, 36.30) | -24.69 (-87.00, 42.32) | -24.96 (-70.08, 29.55) | 0.71 |
| Laser, Triamcinolone | 29.34 (-55.66, 115.59) | -5.56 (-74.37, 75.58) | 8.40 (-43.48, 67.32) | 0.52 |
| Placebo, Probiotics | 10.55 (-43.57, 65.00) | 60.79 (-36.75, 151.55) | 22.30 (-22.82, 67.26) | 0.32 |
| Placebo, Triamcinolone | 35.39 (-47.22, 117.45) | 31.70 (-30.73, 91.02) | 33.73 (-11.82, 76.79) | 0.93 |
| Probiotics, Triamcinolone | -16.45 (-96.23, 63.34) | 32.79 (-41.72, 105.53) | 11.33 (-42.80, 65.10) | 0.32 |

## 4. Network structure

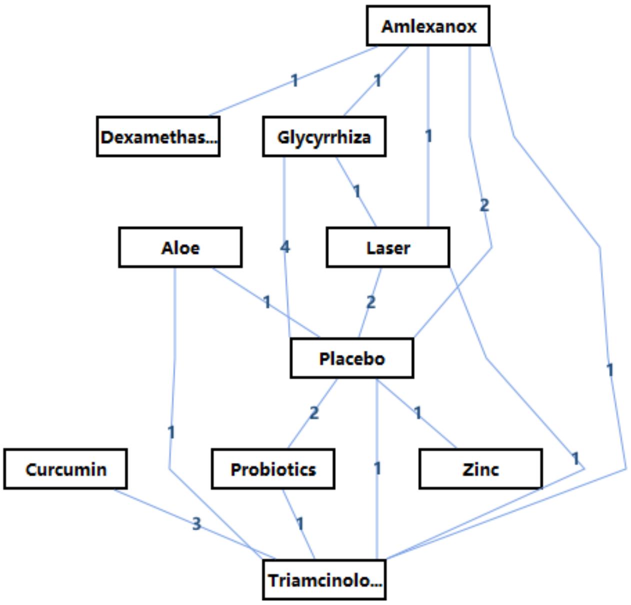


# Day 6

## 1. Consistency Model

### 1.1 Summary estimates

| | | | | | | |
|---|---|---|---|---|---|---|
| Allicin | 10.62 (-115.02, 141.93) | 27.47 (-78.94, 133.87) | -1.06 (-127.34, 128.88) | 16.04 (-123.64, 156.62) | -76.58 (-226.85, 75.07) | 7.24 (-124.72, 134.20) |
| -10.62 (-141.93, 115.02) | Aloe | 17.33 (-92.79, 122.57) | -12.28 (-142.02, 119.28) | 5.97 (-134.45, 146.99) | -87.96 (-240.46, 67.43) | -3.31 (-137.94, 126.47) |
| -27.47 (-133.87, 78.94) | -17.33 (-122.57, 92.79) | Amlexanox | -28.99 (-133.51, 76.27) | -11.59 (-129.64, 105.09) | -104.27 (-232.08, 25.46) | -20.26 (-126.51, 84.91) |
| 1.06 (-128.88, 127.34) | 12.28 (-119.28, 142.02) | 28.99 (-76.27, 133.51) | Berberine gelatin | 16.98 (-121.13, 158.36) | -75.47 (-224.60, 77.28) | 8.31 (-121.30, 133.73) |
| -16.04 (-156.62, 123.64) | -5.97 (-146.99, 134.45) | 11.59 (-105.09, 129.64) | -16.98 (-158.36, 121.13) | Chitosan | -93.20 (-251.02, 70.67) | -8.81 (-150.16, 128.52) |
| 76.58 (-75.07, 226.85) | 87.96 (-67.43, 240.46) | 104.27 (-25.46, 232.08) | 75.47 (-77.28, 224.60) | 93.20 (-70.67, 251.02) | Curcumin | 83.96 (-67.54, 230.58) |
| -7.24 (-134.20, 124.72) | 3.31 (-126.47, 137.94) | 20.26 (-84.91, 126.51) | -8.31 (-133.73, 121.30) | 8.81 (-128.52, 150.16) | -83.96 (-230.58, 67.54) | Dexamethasone |
| 2.37 (-128.74, 129.38) | 12.45 (-116.69, 141.42) | 29.28 (-79.34, 132.96) | 0.33 (-131.43, 130.79) | 18.13 (-123.93, 156.45) | -75.10 (-228.50, 77.44) | 8.05 (-122.98, 138.77) |
| -27.89 (-159.12, 104.53) | -17.56 (-146.96, 119.53) | -0.37 (-111.21, 112.61) | -29.80 (-159.05, 103.90) | -12.46 (-155.02, 133.16) | -104.20 (-254.35, 51.04) | -21.47 (-155.41, 116.26) |
| 17.28 (-74.19, 106.72) | 27.55 (-63.95, 120.59) | 44.22 (-9.12, 97.63) | 15.79 (-73.73, 105.29) | 32.72 (-74.19, 136.98) | -59.55 (-177.74, 61.18) | 23.83 (-67.01, 115.54) |
| -1.74 (-114.62, 105.10) | 8.58 (-108.35, 122.61) | 25.41 (-58.39, 107.82) | -3.05 (-114.99, 107.60) | 14.16 (-110.94, 137.64) | -78.53 (-177.30, 20.89) | 4.52 (-110.37, 117.64) |
| -2.17 (-125.08, 120.70) | 7.56 (-115.17, 136.08) | 24.95 (-76.76, 125.24) | -3.61 (-128.51, 121.62) | 14.26 (-122.55, 148.81) | -79.47 (-206.41, 53.41) | 4.87 (-122.34, 126.05) |

| | | | | |
|---|---|---|---|---|
| -2.37 (-129.38, 128.74) | 27.89 (-104.53, 159.12) | -17.28 (-106.72, 74.19) | 1.74 (-105.10, 114.62) | 2.17 (-120.70, 125.08) |
| -12.45 (-141.42, 116.69) | 17.56 (-119.53, 146.96) | -27.55 (-120.59, 63.95) | -8.58 (-122.61, 108.35) | -7.56 (-136.08, 115.17) |
| -29.28 (-132.96, 79.34) | 0.37 (-112.61, 111.21) | -44.22 (-97.63, 9.12) | -25.41 (-107.82, 58.39) | -24.95 (-125.24, 76.76) |
| -0.33 (-130.79, 131.43) | 29.80 (-103.90, 159.05) | -15.79 (-105.29, 73.73) | 3.05 (-107.60, 114.99) | 3.61 (-121.62, 128.51) |
| -18.13 (-156.45, 123.93) | 12.46 (-133.16, 155.02) | -32.72 (-136.98, 74.19) | -14.16 (-137.64, 110.94) | -14.26 (-148.81, 122.55) |
| 75.10 (-77.44, 228.50) | 104.20 (-51.04, 254.35) | 59.55 (-61.18, 177.74) | 78.53 (-20.89, 177.30) | 79.47 (-53.41, 206.41) |
| -8.05 (-138.77, 122.98) | 21.47 (-116.26, 155.41) | -23.83 (-115.54, 67.01) | -4.52 (-117.64, 110.37) | -4.87 (-126.05, 122.34) |
| Diosmectite | 30.09 (-106.17, 161.28) | -15.12 (-108.35, 76.35) | 3.98 (-111.06, 115.58) | 4.51 (-125.14, 128.72) |
| -30.09 (-161.28, 106.17) | Laser | -45.27 (-139.60, 55.49) | -26.39 (-140.75, 93.45) | -25.62 (-151.09, 105.28) |
| 15.12 (-76.35, 108.35) | 45.27 (-55.49, 139.60) | Placebo | 18.87 (-46.25, 86.53) | 19.47 (-64.48, 103.92) |
| -3.98 (-115.58, 111.06) | 26.39 (-93.45, 140.75) | -18.87 (-86.53, 46.25) | Triamcinolone | 0.91 (-85.34, 85.89) |
| -4.51 (-128.72, 125.14) | 25.62 (-105.28, 151.09) | -19.47 (-103.92, 64.48) | -0.91 (-85.89, 85.34) | Triester Glycerol Oxide |

## 1.2 Rank probability(Rank 1 is best, rank N is worst)

| Drug | Rank 1 | Rank 2 | Rank 3 | Rank 4 | Rank 5 | Rank 6 | Rank 7 | Rank 8 | Rank 9 | Rank 10 | Rank 11 | Rank 12 |
|---|---|---|---|---|---|---|---|---|---|---|---|---|
| Allicin | 0.06 | 0.07 | 0.08 | 0.08 | 0.09 | 0.1 | 0.09 | 0.08 | 0.09 | 0.09 | 0.11 | 0.05 |
| Aloe | 0.1 | 0.11 | 0.11 | 0.1 | 0.1 | 0.09 | 0.08 | 0.08 | 0.06 | 0.06 | 0.07 | 0.03 |
| Amlexanox | 0.12 | 0.19 | 0.18 | 0.16 | 0.12 | 0.09 | 0.06 | 0.03 | 0.02 | 0.01 | 0.01 | 0 |
| Berberine gelatin | 0.06 | 0.07 | 0.08 | 0.09 | 0.09 | 0.09 | 0.09 | 0.09 | 0.09 | 0.1 | 0.11 | 0.05 |
| Chitosan | 0.19 | 0.11 | 0.09 | 0.08 | 0.07 | 0.07 | 0.06 | 0.06 | 0.05 | 0.07 | 0.09 | 0.05 |
| Curcumin | 0.01 | 0.01 | 0.01 | 0.01 | 0.02 | 0.02 | 0.02 | 0.03 | 0.03 | 0.06 | 0.12 | 0.67 |
| Dexamethasone | 0.09 | 0.1 | 0.1 | 0.1 | 0.1 | 0.09 | 0.08 | 0.08 | 0.08 | 0.08 | 0.08 | 0.04 |
| Diosmectite | 0.06 | 0.07 | 0.07 | 0.08 | 0.09 | 0.09 | 0.09 | 0.09 | 0.09 | 0.1 | 0.12 | 0.05 |
| Laser | 0.24 | 0.15 | 0.12 | 0.09 | 0.08 | 0.07 | 0.06 | 0.05 | 0.04 | 0.04 | 0.04 | 0.02 |
| Placebo | 0 | 0 | 0 | 0 | 0.02 | 0.06 | 0.12 | 0.2 | 0.26 | 0.21 | 0.11 | 0.01 |
| Triamcinolone | 0.02 | 0.05 | 0.08 | 0.11 | 0.12 | 0.13 | 0.13 | 0.11 | 0.1 | 0.09 | 0.05 | 0 |
| Triester Glycerol Oxide | 0.05 | 0.07 | 0.08 | 0.1 | 0.1 | 0.11 | 0.1 | 0.1 | 0.09 | 0.09 | 0.09 | 0.03 |

## 1.3 Consistency check

| Parameter | Median (95% CI) |
|---|---|
| Random Effects Standard Deviation | 37.38 (15.59, 76.19) |

### 1.4 Convergence Diagnostics

| Parameter | PSRF |
|---|---|
| d.Placebo.Allicin | 1.00 |
| d.Placebo.Aloe | 1.00 |
| d.Placebo.Amlexanox | 1.00 |
| d.Placebo.Berberinegelatin | 1.00 |
| d.Placebo.Chitosan | 1.00 |
| d.Placebo.Dexamethasone | 1.00 |
| d.Placebo.Diosmectite | 1.00 |
| d.Placebo.Laser | 1.00 |
| d.Placebo.Triamcinolone | 1.00 |
| d.Placebo.TriesterGlycerolOxide | 1.00 |
| d.Triamcinolone.Curcumin | 1.00 |
| sd.d | 1.00 |

Number of chains : 4
Tuning iterations : 20,000
Simulation iterations : 50,000
Thinning interval : 10
Inference samples : 10,000
Variance scaling factor: 2.5

## 2.Inconsistency Model

### 2.1 Summary estimates

| | | | | | | | |
|---|---|---|---|---|---|---|---|
| Allicin | 11.02 (-116.41, 138.17) | 26.38 (-81.98, 133.32) | -0.79 (-131.28, 129.26) | 14.32 (-129.90, 156.87) | -77.66 (-228.47, 80.35) | 6.76 (-124.28, 137.60) | -2.32 (-134.97, 128.16) |
| -11.02 (-138.17, 116.41) | Aloe | 15.64 (-92.45, 118.23) | -13.02 (-140.21, 119.51) | 4.33 (-134.66, 145.26) | -88.94 (-236.22, 64.72) | -3.98 (-133.20, 125.94) | -13.14 (-142.28, 115.91) |
| -26.38 (-133.32, 81.98) | -15.64 (-118.23, 92.45) | Amlexanox | -28.21 (-131.32, 79.57) | -11.61 (-128.42, 111.64) | -104.26 (-232.83, 35.63) | -19.35 (-125.36, 89.58) | -29.13 (-132.43, 78.73) |
| 0.79 (-129.26, 131.28) | 13.02 (-119.51, 140.21) | 28.21 (-79.57, 131.32) | Berberine gelatin | 15.94 (-123.28, 156.78) | -76.58 (-223.88, 77.08) | 7.64 (-122.35, 140.77) | -0.99 (-130.73, 131.28) |
| -14.32 (-156.87, 129.90) | -4.33 (-145.26, 134.66) | 11.61 (-111.64, 128.42) | -15.94 (-156.78, 123.28) | Chitosan | -91.50 (-253.93, 71.75) | -8.93 (-150.75, 131.94) | -17.35 (-158.01, 123.59) |
| 77.66 (-80.35, 228.47) | 88.94 (-64.72, 236.22) | 104.26 (-35.63, 232.83) | 76.58 (-77.08, 223.88) | 91.50 (-71.75, 253.93) | Curcumin | 85.64 (-74.74, 236.95) | 75.67 (-77.29, 223.91) |
| -6.76 (-137.60, 124.28) | 3.98 (-125.94, 133.20) | 19.35 (-89.58, 125.36) | -7.64 (-140.77, 122.35) | 8.93 (-131.94, 150.75) | -85.64 (-236.95, 74.74) | Dexamethasone | -9.06 (-139.83, 120.66) |
| 2.32 (-128.16, 134.97) | 13.14 (-115.91, 142.28) | 29.13 (-78.73, 132.43) | 0.99 (-131.28, 130.73) | 17.35 (-123.59, 158.01) | -75.67 (-223.91, 77.29) | 9.06 (-120.66, 139.83) | Diosmectite |
| -27.16 (-165.93, 109.42) | -16.33 (-153.62, 115.27) | -0.49 (-114.89, 108.54) | -29.07 (-165.14, 107.03) | -12.50 (-156.59, 132.81) | -104.23 (-262.11, 52.96) | -20.49 (-158.08, 117.93) | -29.90 (-168.42, 104.95) |
| 17.52 (-75.60, 110.39) | 28.60 (-63.60, 120.12) | 44.26 (-12.35, 95.21) | 16.02 (-73.85, 107.74) | 32.91 (-73.57, 141.03) | -59.66 (-176.85, 63.54) | 24.42 (-67.05, 116.81) | 15.42 (-76.81, 108.33) |
| -1.07 (-117.50, 113.23) | 9.60 (-101.39, 120.61) | 25.36 (-61.67, 106.44) | -2.35 (-113.55, 109.98) | 13.77 (-112.56, 139.67) | -78.85 (-179.98, 23.50) | 5.47 (-112.01, 118.98) | -3.66 (-119.12, 110.15) |
| -1.41 (-130.92, 124.43) | 9.48 (-115.20, 132.44) | 25.13 (-78.83, 124.35) | -3.17 (-129.49, 123.52) | 13.17 (-126.36, 151.37) | -78.80 (-212.67, 55.41) | 5.12 (-122.42, 134.34) | -4.08 (-133.47, 122.16) |

| | | | |
|---|---|---|---|
| 27.16 (-109.42, 165.93) | -17.52 (-110.39, 75.60) | 1.07 (-113.23, 117.50) | 1.41 (-124.43, 130.92) |
| 16.33 (-115.27, 153.62) | -28.60 (-120.12, 63.60) | -9.60 (-120.61, 101.39) | -9.48 (-132.44, 115.20) |
| 0.49 (-108.54, 114.89) | -44.26 (-95.21, 12.35) | -25.36 (-106.44, 61.67) | -25.13 (-124.35, 78.83) |
| 29.07 (-107.03, 165.14) | -16.02 (-107.74, 73.85) | 2.35 (-109.98, 113.55) | 3.17 (-123.52, 129.49) |
| 12.50 (-132.81, 156.59) | -32.91 (-141.03, 73.57) | -13.77 (-139.67, 112.56) | -13.17 (-151.37, 126.36) |
| 104.23 (-52.96, 262.11) | 59.66 (-63.54, 176.85) | 78.85 (-23.50, 179.98) | 78.80 (-55.41, 212.67) |
| 20.49 (-117.93, 158.08) | -24.42 (-116.81, 67.05) | -5.47 (-118.98, 112.01) | -5.12 (-134.34, 122.42) |
| 29.90 (-104.95, 168.42) | -15.42 (-108.33, 76.81) | 3.66 (-110.15, 119.12) | 4.08 (-122.16, 133.47) |
| Laser | -45.02 (-145.41, 53.44) | -26.29 (-144.49, 95.25) | -25.94 (-156.16, 104.19) |
| 45.02 (-53.44, 145.41) | Placebo | 19.00 (-44.19, 85.78) | 18.82 (-66.02, 107.07) |
| 26.29 (-95.25, 144.49) | -19.00 (-85.78, 44.19) | Triamcinolone | 0.40 (-84.69, 85.98) |
| 25.94 (-104.19, 156.16) | -18.82 (-107.07, 66.02) | -0.40 (-85.98, 84.69) | Triester Glycerol Oxide |

2.2 Variance Calculation

| Parameter | Median (95% CI) |
|---|---|
| Random Effects Standard Deviation | 37.78 (15.91, 76.37) |
| Inconsistency Standard Deviation | 40.92 (1.92, 79.05) |

2.4 Convergence Diagnostics

| Parameter | PSRF |
|---|---|
| d.Allicin.Placebo | 1.00 |
| d.Placebo.Aloe | 1.00 |
| d.Placebo.Amlexanox | 1.00 |
| d.Placebo.Berberinegelatin | 1.00 |
| d.Placebo.Chitosan | 1.00 |
| d.Placebo.Dexamethasone | 1.00 |
| d.Placebo.Diosmectite | 1.00 |
| d.Placebo.Laser | 1.00 |
| d.Placebo.Triamcinolone | 1.00 |
| d.Triamcinolone.Curcumin | 1.00 |
| d.Triamcinolone.TriesterGlycerol... | 1.00 |
| sd.d | 1.00 |
| sd.w | 1.00 |

Number of chains : 4
Tuning iterations : 20,000
Simulation iterations : 50,000
Thinning interval : 10
Inference samples : 10,000
Variance scaling factor: 2.5

## 3. Network structure

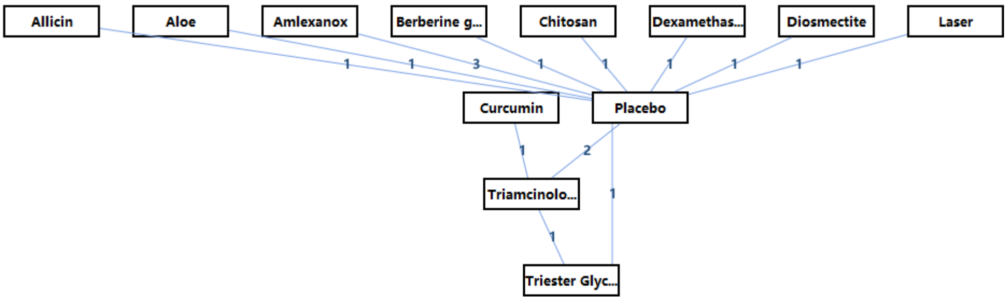

**Day 7**

## 1. Consistency Model

### 1.1 Summary estimates

| | | | | | | | | | |
|---|---|---|---|---|---|---|---|---|---|
| Aloe | 22.98 (-72.30, 116.68) | 7.61 (-67.80, 87.05) | 17.51 (-32.36, 69.90) | 30.72 (-41.26, 103.68) | 1.51 (-53.91, 54.32) | -1.49 (-40.02, 38.76) | 19.31 (-62.63, 98.64) | 11.29 (-36.97, 57.19) | 10.57 (-60.92, 82.67) |
| -22.98 (-116.68, 72.30) | Amlexanox | -14.83 (-123.11, 94.37) | -5.20 (-99.55, 92.58) | 7.85 (-52.11, 69.04) | -21.04 (-115.09, 74.25) | -24.15 (-107.89, 62.93) | -3.80 (-118.51, 113.03) | -11.90 (-106.25, 83.03) | -12.90 (-116.33, 92.88) |
| -7.61 (-87.05, 67.80) | 14.83 (-94.37, 123.11) | Cryotherapy | 9.45 (-67.42, 86.54) | 22.95 (-69.11, 113.10) | -6.19 (-83.99, 69.68) | -8.97 (-74.43, 57.58) | 11.29 (-89.63, 111.97) | 3.13 (-73.53, 78.31) | 2.09 (-85.42, 90.44) |
| -17.51 (-69.90, 32.36) | 5.20 (-92.58, 99.55) | -9.45 (-86.54, 67.42) | Curcumin | 13.29 (-62.43, 84.05) | -15.82 (-68.40, 32.76) | -18.81 (-58.53, 22.56) | 1.58 (-70.79, 73.91) | -6.45 (-38.17, 23.82) | -7.93 (-79.71, 67.69) |
| -30.72 (-103.68, 41.26) | -7.85 (-69.04, 52.11) | -22.95 (-113.10, 69.11) | -13.29 (-84.05, 62.43) | Dexamethasone | -29.81 (-102.89, 44.40) | -31.99 (-91.88, 30.21) | -12.12 (-108.89, 86.68) | -19.79 (-92.09, 53.26) | -20.93 (-105.32, 65.72) |
| -1.51 (-54.32, 53.91) | 21.04 (-74.25, 115.09) | 6.19 (-69.68, 83.99) | 15.82 (-32.76, 68.40) | 29.81 (-44.40, 102.89) | Laser | -2.75 (-42.46, 39.73) | 17.52 (-62.75, 98.25) | 9.18 (-36.24, 56.85) | 8.54 (-61.04, 82.65) |
| 1.49 (-38.76, 40.02) | 24.15 (-62.93, 107.89) | 8.97 (-57.58, 74.43) | 18.81 (-22.56, 58.53) | 31.99 (-30.21, 91.88) | 2.75 (-39.73, 42.46) | Placebo | 20.48 (-57.48, 95.11) | 12.40 (-27.35, 48.50) | 11.27 (-48.66, 71.68) |
| -19.31 (-98.64, 62.63) | 3.80 (-113.03, 118.51) | -11.29 (-111.97, 89.63) | -1.58 (-73.91, 70.79) | 12.12 (-86.68, 108.89) | -17.52 (-98.25, 62.75) | -20.48 (-95.11, 57.48) | Probiotics | -8.36 (-72.45, 57.30) | -9.85 (-103.78, 90.18) |
| -11.29 (-57.19, 36.97) | 11.90 (-83.03, 106.25) | -3.13 (-78.31, 73.53) | 6.45 (-23.82, 38.17) | 19.79 (-53.26, 92.09) | -9.18 (-56.85, 36.24) | -12.40 (-48.50, 27.35) | 8.36 (-57.30, 72.45) | Triamcinolone | -1.55 (-71.30, 72.37) |
| -10.57 (-82.67, 60.92) | 12.90 (-92.88, 116.33) | -2.09 (-90.44, 85.42) | 7.93 (-67.69, 79.71) | 20.93 (-65.72, 105.32) | -8.54 (-82.65, 61.04) | -11.27 (-71.68, 48.66) | 9.85 (-90.18, 103.78) | 1.55 (-72.37, 71.30) | Zinc |

1.2 Rank probability(Rank 1 is best, rank N is worst)

| Drug | Rank 1 | Rank 2 | Rank 3 | Rank 4 | Rank 5 | Rank 6 | Rank 7 | Rank 8 | Rank 9 | Rank 10 |
|---|---|---|---|---|---|---|---|---|---|---|
| Aloe | 0.02 | 0.03 | 0.05 | 0.08 | 0.1 | 0.12 | 0.13 | 0.14 | 0.17 | 0.15 |
| Amlexanox | 0.22 | 0.18 | 0.11 | 0.08 | 0.06 | 0.05 | 0.05 | 0.06 | 0.07 | 0.14 |
| Cryotherapy | 0.12 | 0.09 | 0.1 | 0.09 | 0.07 | 0.07 | 0.08 | 0.08 | 0.1 | 0.19 |
| Curcumin | 0.07 | 0.12 | 0.17 | 0.17 | 0.16 | 0.11 | 0.08 | 0.05 | 0.04 | 0.02 |
| Dexamethasone | 0.22 | 0.26 | 0.14 | 0.09 | 0.07 | 0.06 | 0.04 | 0.04 | 0.05 | 0.02 |
| Laser | 0.02 | 0.04 | 0.07 | 0.09 | 0.11 | 0.12 | 0.13 | 0.14 | 0.15 | 0.14 |
| Placebo | 0 | 0 | 0.01 | 0.04 | 0.08 | 0.15 | 0.22 | 0.24 | 0.18 | 0.06 |
| Probiotics | 0.22 | 0.12 | 0.12 | 0.09 | 0.07 | 0.06 | 0.06 | 0.06 | 0.08 | 0.13 |
| Triamcinolone | 0.02 | 0.06 | 0.11 | 0.16 | 0.18 | 0.16 | 0.13 | 0.1 | 0.06 | 0.02 |
| Zinc | 0.1 | 0.09 | 0.11 | 0.11 | 0.09 | 0.08 | 0.08 | 0.08 | 0.11 | 0.14 |

1.3 Consistency check

| Parameter | Median (95% CI) |
|---|---|
| Random Effects Standard Deviation | 26.48 (14.59, 42.00) |

1.4 Convergence Diagnostics

| Parameter | PSRF |
|---|---|
| d.Dexamethasone.Amlexanox | 1.00 |
| d.Placebo.Aloe | 1.00 |
| d.Placebo.Cryotherapy | 1.00 |
| d.Placebo.Curcumin | 1.00 |
| d.Placebo.Dexamethasone | 1.00 |
| d.Placebo.Laser | 1.00 |
| d.Placebo.Triamcinolone | 1.00 |
| d.Placebo.Zinc | 1.08 |
| d.Triamcinolone.Probiotics | 1.00 |
| sd.d | 1.00 |

Number of chains : 4
Tuning iterations : 20,000
Simulation iterations : 50,000
Thinning interval : 10
Inference samples : 10,000
Variance scaling factor: 2.5

## 2.Inconsistency Model

2.1 Summary estimates

| | | | | |
|---|---|---|---|---|
| Aloe | 33.62 (-71.30, 121.56) | 19.16 (-62.12, 94.32) | 25.93 (-27.75, 82.30) | 42.01 (-36.39, 112.30) |
| -33.62 (-121.56, 71.30) | Amlexanox | -13.90 (-111.30, 95.87) | -7.61 (-92.14, 99.05) | 8.79 (-45.54, 70.54) |
| -19.16 (-94.32, 62.12) | 13.90 (-95.87, 111.30) | Cryotherapy | 6.54 (-65.95, 87.87) | 22.95 (-65.80, 106.17) |
| -25.93 (-82.30, 27.75) | 7.61 (-99.05, 92.14) | -6.54 (-87.87, 65.95) | Curcumin | 16.20 (-61.83, 84.07) |
| -42.01 (-112.30, 36.39) | -8.79 (-70.54, 45.54) | -22.95 (-106.17, 65.80) | -16.20 (-84.07, 61.83) | Dexamethasone |
| 4.33 (-58.48, 76.81) | 37.87 (-62.50, 129.84) | 23.46 (-57.34, 110.14) | 29.10 (-22.76, 99.13) | 45.46 (-30.81, 131.12) |
| -9.80 (-53.15, 38.22) | 23.17 (-64.26, 102.43) | 9.08 (-54.18, 73.81) | 15.75 (-21.20, 61.04) | 31.69 (-28.40, 90.43) |
| -27.77 (-113.34, 54.29) | 5.55 (-122.60, 109.33) | -8.91 (-114.44, 86.43) | -2.27 (-72.44, 67.74) | 14.03 (-88.97, 104.05) |
| -11.68 (-59.38, 33.13) | 13.71 (-93.74, 98.95) | -0.71 (-81.00, 72.53) | 5.83 (-24.00, 36.04) | 21.97 (-56.72, 89.39) |
| -20.29 (-110.20, 56.75) | 15.54 (-120.92, 110.18) | -1.49 (-104.86, 84.59) | 5.09 (-76.86, 76.53) | 21.77 (-81.36, 103.23) |

| | | | | |
|---|---|---|---|---|
| -4.33 (-76.81, 58.48) | 9.80 (-38.22, 53.15) | 27.77 (-54.29, 113.34) | 11.68 (-33.13, 59.38) | 20.29 (-56.75, 110.20) |
| -37.87 (-129.84, 62.50) | -23.17 (-102.43, 64.26) | -5.55 (-109.33, 122.60) | -13.71 (-98.95, 93.74) | -15.54 (-110.18, 120.92) |
| -23.46 (-110.14, 57.34) | -9.08 (-73.81, 54.18) | 8.91 (-86.43, 114.44) | 0.71 (-72.53, 81.00) | 1.49 (-84.59, 104.86) |
| -29.10 (-99.13, 22.76) | -15.75 (-61.04, 21.20) | 2.27 (-67.74, 72.44) | -5.83 (-36.04, 24.00) | -5.09 (-76.53, 76.86) |
| -45.46 (-131.12, 30.81) | -31.69 (-90.43, 28.40) | -14.03 (-104.05, 88.97) | -21.97 (-89.39, 56.72) | -21.77 (-103.23, 81.36) |
| Laser | 2.31 (-34.42, 51.17) | 32.76 (-47.72, 124.01) | 22.53 (-24.80, 90.69) | 21.50 (-61.85, 142.39) |
| -2.31 (-51.17, 34.42) | Placebo | 18.04 (-53.35, 97.69) | 28.55 (-17.58, 69.69) | 8.90 (-49.65, 90.80) |
| -32.76 (-124.01, 47.72) | -18.04 (-97.69, 53.35) | Probiotics | -8.36 (-71.91, 55.53) | -7.36 (-103.84, 95.96) |
| -22.53 (-90.69, 24.80) | -28.55 (-69.69, 17.58) | 8.36 (-55.53, 71.91) | Triamcinolone | 1.04 (-68.99, 79.48) |
| -21.50 (-142.39, 61.85) | -8.90 (-90.80, 49.65) | 7.36 (-95.96, 103.84) | -1.04 (-79.48, 68.99) | Zinc |

## 2.2 Inconsistency Factors

| Cycle | Median (95% CI) |
|---|---|
| Aloe, Curcumin, Placebo, Triamcinolone | 5.55 (-30.60, 51.07) |
| Curcumin, Laser, Placebo, Triamcinolone | 6.31 (-29.65, 67.59) |
| Curcumin, Placebo, Triamcinolone | 14.09 (-19.30, 61.60) |

## 2.3 Variance Calculation

| Parameter | Median (95% CI) |
|---|---|
| Random Effects Standard Deviation | 24.40 (7.05, 41.92) |
| Inconsistency Standard Deviation | 23.30 (1.93, 42.57) |

## 2.4 Convergence Diagnostics

| Parameter | PSRF |
|---|---|
| d.Aloe.Placebo | 1.01 |
| d.Curcumin.Triamcinolone | 1.01 |
| d.Dexamethasone.Amlexanox | 1.19 |
| d.Placebo.Cryotherapy | 1.00 |
| d.Placebo.Curcumin | 1.06 |
| d.Placebo.Dexamethasone | 1.00 |
| d.Placebo.Zinc | 1.68 |
| d.Triamcinolone.Laser | 1.76 |
| d.Triamcinolone.Probiotics | 1.00 |
| w.Aloe.Placebo.Curcumin.Triamc... | 1.01 |
| w.Curcumin.Placebo.Laser.Triam... | 1.12 |
| w.Curcumin.Placebo.Triamcinolo... | 1.01 |
| sd.d | 1.06 |
| sd.w | 1.01 |

Number of chains : 4

Tuning iterations : 20,000

Simulation iterations : 50,000

Thinning interval : 10

Inference samples : 10,000

Variance scaling factor: 2.5

## 3. Node-splitting analysis

| Name | Direct Effect | Indirect Effect | Overall | P-Value |
|---|---|---|---|---|
| Aloe, Triamcinolone | 10.03 (-46.07, 70.52) | -9.03 (-74.33, 57.86) | 11.29 (-36.97, 57.19) | 0.61 |
| Curcumin, Placebo | -2.09 (-60.34, 56.28) | -35.17 (-91.21, 21.28) | -18.81 (-58.53, 22.56) | 0.37 |
| Curcumin, Triamcinolone | -11.53 (-47.40, 20.19) | 21.25 (-54.91, 92.39) | -6.45 (-38.17, 23.82) | 0.37 |
| Laser, Placebo | -1.56 (-49.22, 50.99) | -4.35 (-85.85, 79.11) | -2.75 (-42.46, 39.73) | 0.95 |
| Laser, Triamcinolone | 7.43 (-63.13, 77.77) | 11.55 (-52.61, 77.03) | 9.18 (-36.24, 56.85) | 0.94 |
| Placebo, Triamcinolone | 39.96 (-19.36, 97.87) | -2.85 (-56.38, 47.59) | 12.40 (-27.35, 48.50) | 0.24 |

## 4. Network structure

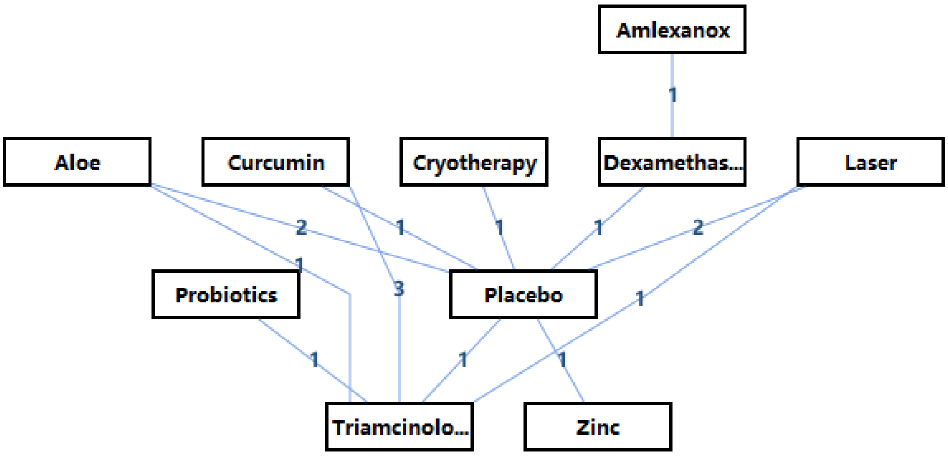


**Day 10**

## 1. Consistency Model

### 1.1 Summary estimates

| | | | | | | | |
|---|---|---|---|---|---|---|---|
| Aloe | 13.96 (-29.65, 55.90) | 3.25 (-36.87, 47.86) | 12.45 (-36.52, 60.32) | 13.85 (-25.91, 53.36) | 0.00 (-26.04, 26.61) | 1.69 (-51.49, 55.64) | 13.79 (-25.69, 53.29) |
| -13.96 (-55.90, 29.65) | Benzydamine | -9.56 (-50.22, 33.82) | -2.12 (-43.76, 40.48) | -0.37 (-30.60, 32.31) | -13.98 (-47.18, 20.09) | -10.43 (-60.34, 38.18) | -0.57 (-30.48, 32.00) |
| -3.25 (-47.86, 36.87) | 9.56 (-33.82, 50.22) | Cryotherapy | 8.31 (-40.40, 54.07) | 10.44 (-29.11, 47.49) | -3.15 (-38.60, 26.33) | -2.21 (-55.58, 50.46) | 10.42 (-29.48, 47.90) |
| -12.45 (-60.32, 36.52) | 2.12 (-40.48, 43.76) | -8.31 (-54.07, 40.40) | Curcumin | 1.42 (-34.98, 40.13) | -12.39 (-52.12, 27.94) | -9.03 (-56.31, 35.82) | 1.50 (-26.29, 29.96) |
| -13.85 (-53.36, 25.91) | 0.37 (-32.31, 30.60) | -10.44 (-47.49, 29.11) | -1.42 (-40.13, 34.98) | Doxycycline | -13.86 (-42.66, 14.13) | -11.12 (-57.56, 32.92) | 0.03 (-26.40, 25.94) |
| -0.00 (-26.61, 26.04) | 13.98 (-20.09, 47.18) | 3.15 (-26.33, 38.60) | 12.39 (-27.94, 52.12) | 13.86 (-14.13, 42.66) | Placebo | 1.72 (-43.99, 50.06) | 13.79 (-14.33, 42.93) |
| -1.69 (-55.64, 51.49) | 10.43 (-38.18, 60.34) | 2.21 (-50.46, 55.58) | 9.03 (-35.82, 56.31) | 11.12 (-32.92, 57.56) | -1.72 (-50.06, 43.99) | Probiotics | 11.45 (-25.77, 49.21) |
| -13.79 (-53.29, 25.69) | 0.57 (-32.00, 30.48) | -10.42 (-47.90, 29.48) | -1.50 (-29.96, 26.29) | -0.03 (-25.94, 26.40) | -13.79 (-42.93, 14.33) | -11.45 (-49.21, 25.77) | Triamcinolone |

### 1.2 Rank probability(Rank 1 is best, rank N is worst)

| Drug | Rank 1 | Rank 2 | Rank 3 | Rank 4 | Rank 5 | Rank 6 | Rank 7 | Rank 8 |
|---|---|---|---|---|---|---|---|---|
| Aloe | 0.05 | 0.05 | 0.06 | 0.07 | 0.11 | 0.17 | 0.25 | 0.25 |
| Benzydamine | 0.28 | 0.16 | 0.12 | 0.14 | 0.12 | 0.09 | 0.06 | 0.05 |
| Cryotherapy | 0.09 | 0.08 | 0.08 | 0.1 | 0.17 | 0.18 | 0.14 | 0.16 |
| Curcumin | 0.2 | 0.15 | 0.13 | 0.16 | 0.13 | 0.1 | 0.07 | 0.06 |
| Doxycycline | 0.15 | 0.22 | 0.23 | 0.18 | 0.11 | 0.06 | 0.03 | 0.02 |
| Placebo | 0.01 | 0.03 | 0.04 | 0.08 | 0.14 | 0.24 | 0.31 | 0.15 |
| Probiotics | 0.13 | 0.07 | 0.06 | 0.07 | 0.13 | 0.11 | 0.11 | 0.31 |
| Triamcinolone | 0.09 | 0.24 | 0.29 | 0.2 | 0.1 | 0.06 | 0.02 | 0 |

1.3 Consistency check

| Parameter | Median (95% CI) |
|---|---|
| Random Effects Standard Deviation | 10.38 (0.44, 20.77) |

1.4 Convergence Diagnostics

| Parameter | PSRF |
|---|---|
| d.Placebo.Aloe | 1.00 |
| d.Placebo.Benzydamine | 1.15 |
| d.Placebo.Cryotherapy | 1.67 |
| d.Placebo.Doxycycline | 1.27 |
| d.Placebo.Triamcinolone | 1.26 |
| d.Triamcinolone.Curcumin | 1.00 |
| d.Triamcinolone.Probiotics | 1.00 |
| sd.d | 1.01 |

Number of chains : 4
Tuning iterations : 20,000
Simulation iterations : 50,000
Thinning interval : 10
Inference samples : 10,000
Variance scaling factor: 2.5

## 2.Inconsistency Model

2.1 Summary estimates

| | | | | | | | |
|---|---|---|---|---|---|---|---|
| Aloe | 26.37 (-19.12, 69.63) | 0.52 (-41.67, 46.63) | 20.10 (-30.64, 69.96) | 21.36 (-21.09, 62.32) | 0.02 (-26.07, 27.39) | 9.32 (-46.39, 65.06) | 21.39 (-20.86, 62.61) |
| -26.37 (-69.63, 19.12) | Benzydamine | -25.64 (-77.97, 27.56) | -6.82 (-47.81, 36.07) | -5.16 (-35.60, 26.67) | -26.05 (-62.30, 11.77) | -16.63 (-64.92, 33.82) | -5.12 (-35.69, 25.80) |
| -0.52 (-46.63, 41.67) | 25.64 (-27.56, 77.97) | Cryotherapy | 19.40 (-36.75, 73.93) | 21.06 (-29.33, 67.48) | -0.54 (-37.73, 34.47) | 8.98 (-54.23, 68.23) | 21.16 (-28.45, 66.85) |
| -20.10 (-69.96, 30.64) | 6.82 (-36.07, 47.81) | -19.40 (-73.93, 36.75) | Curcumin | 1.73 (-36.30, 40.16) | -19.56 (-61.73, 23.43) | -10.75 (-57.04, 37.09) | 1.66 (-26.46, 29.11) |
| -21.36 (-62.32, 21.09) | 5.16 (-26.67, 35.60) | -21.06 (-67.48, 29.33) | -1.73 (-40.16, 36.30) | Doxycycline | -20.91 (-53.45, 12.42) | -12.71 (-56.36, 35.87) | 0.04 (-27.09, 25.96) |
| -0.02 (-27.39, 26.07) | 26.05 (-11.77, 62.30) | 0.54 (-34.47, 37.73) | 19.56 (-23.43, 61.73) | 20.91 (-12.42, 53.45) | Placebo | 9.30 (-40.48, 59.12) | 21.25 (-12.39, 53.05) |
| -9.32 (-65.06, 46.39) | 16.63 (-33.82, 64.92) | -8.98 (-68.23, 54.23) | 10.75 (-37.09, 57.04) | 12.71 (-35.87, 56.36) | -9.30 (-59.12, 40.48) | Probiotics | 12.23 (-26.64, 49.63) |
| -21.39 (-62.61, 20.86) | 5.12 (-25.80, 35.69) | -21.16 (-66.85, 28.45) | -1.66 (-29.11, 26.46) | -0.04 (-25.96, 27.09) | -21.25 (-53.05, 12.39) | -12.23 (-49.63, 26.64) | Triamcinolone |

2.2 Variance Calculation

| Parameter | Median (95% CI) |
|---|---|
| Random Effects Standard Deviation | 10.63 (0.61, 20.75) |
| Inconsistency Standard Deviation | 10.50 (0.51, 20.74) |

2. Convergence Diagnostics

| Parameter | PSRF |
|---|---|
| d.Aloe.Placebo | 1.00 |
| d.Benzydamine.Doxycycline | 1.52 |
| d.Doxycycline.Triamcinolone | 1.00 |
| d.Placebo.Benzydamine | 1.34 |
| d.Placebo.Cryotherapy | 1.00 |
| d.Triamcinolone.Curcumin | 1.00 |
| d.Triamcinolone.Probiotics | 1.00 |
| sd.d | 1.00 |
| sd.w | 1.00 |

Number of chains : 4
Tuning iterations : 20,000
Simulation iterations : 50,000
Thinning interval : 10
Inference samples : 10,000
Variance scaling factor: 2.5

## 3. Node-splitting analysis

| Name | Direct Effect | Indirect Effect | Overall | P-Value |
|---|---|---|---|---|
| Placebo, Triamcinolone | 11.40 (-33.38, 50.39) | 4.38 (-627.78, 629.87) | 13.79 (-14.33, 42.93) | 0.99 |

4. Network structure

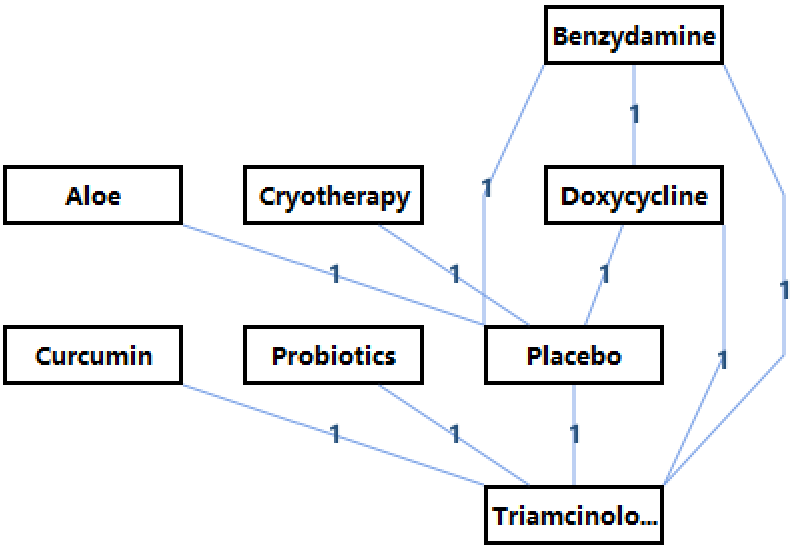

# Chapter S3 Symptom-reducing effect

## 1.Consistency Model

### Summary estimates for Symptom-reducing effect

The estimate values for symptom-reducing effect are given as mean difference (MD) and 95% confidence interval (CI). The table shows the pooled estimates based on the network meta-analysis during the treatment period for each study.

| | | | | | | | | | |
|---|---|---|---|---|---|---|---|---|---|
| Allicin | -11.13 (-69.75, 47.06) | -0.48 (-51.98, 51.58) | -6.81 (-69.49, 57.85) | -7.33 (-76.31, 59.28) | -15.21 (-87.00, 56.61) | 4.35 (-63.41, 74.46) | 8.78 (-53.20, 68.00) | -5.10 (-65.20, 52.19) | -6.24 (-74.41, 60.89) |
| 11.13 (-47.06, 69.75) | Aloe | 11.04 (-26.53, 49.29) | 4.46 (-46.64, 57.67) | 3.64 (-54.99, 64.38) | -3.78 (-66.84, 59.91) | 15.57 (-43.06, 75.11) | 19.52 (-26.95, 67.12) | 5.73 (-41.98, 54.53) | 5.17 (-53.64, 63.37) |
| 0.48 (-51.58, 51.98) | -11.04 (-49.29, 26.53) | Amlexanox | -6.04 (-49.98, 36.96) | -7.13 (-58.15, 45.02) | -15.16 (-71.55, 42.48) | 5.10 (-47.83, 58.04) | 8.19 (-30.35, 48.31) | -5.15 (-39.53, 29.62) | -5.78 (-57.63, 44.66) |
| 6.81 (-57.85, 69.49) | -4.46 (-57.67, 46.64) | 6.04 (-36.96, 49.98) | Benzydamine | -1.33 (-63.47, 63.12) | -8.29 (-76.68, 57.75) | 11.22 (-52.03, 74.46) | 14.44 (-35.37, 65.93) | 1.17 (-51.24, 53.76) | 0.60 (-63.32, 62.90) |
| 7.33 (-59.28, 76.31) | -3.64 (-64.38, 54.99) | 7.13 (-45.02, 58.15) | 1.33 (-63.12, 63.47) | Berberine gelatin | -7.91 (-78.66, 64.66) | 12.00 (-58.02, 82.88) | 15.63 (-43.16, 75.85) | 2.28 (-58.73, 61.93) | 1.47 (-67.67, 69.65) |
| 15.21 (-56.61, 87.00) | 3.78 (-59.91, 66.84) | 15.16 (-42.48, 71.55) | 8.29 (-57.75, 76.68) | 7.91 (-64.66, 78.66) | Chitosan | 19.92 (-53.93, 93.32) | 23.57 (-41.96, 86.94) | 9.74 (-55.05, 72.70) | 9.29 (-63.25, 78.93) |
| -4.35 (-74.46, 63.41) | -15.57 (-75.11, 43.06) | -5.10 (-58.04, 47.83) | -11.22 (-74.46, 52.03) | -12.00 (-82.88, 58.02) | -19.92 (-93.32, 53.93) | Chlorhexidine | 3.81 (-53.55, 60.13) | -9.78 (-72.01, 50.47) | -10.64 (-80.90, 58.14) |
| -8.78 (-68.00, 53.20) | -19.52 (-67.12, 26.95) | -8.19 (-48.31, 30.35) | -14.44 (-65.93, 35.37) | -15.63 (-75.85, 43.16) | -23.57 (-86.94, 41.96) | -3.81 (-60.13, 53.55) | Curcumin | -13.19 (-63.86, 34.96) | -14.12 (-74.81, 46.31) |
| 5.10 (-52.19, 65.20) | -5.73 (-54.53, 41.98) | 5.15 (-29.62, 39.53) | -1.17 (-53.76, 51.24) | -2.28 (-61.93, 58.73) | -9.74 (-72.70, 55.05) | 9.78 (-50.47, 72.01) | 13.19 (-34.96, 63.86) | Dexamethasone | -1.00 (-60.79, 58.99) |
| 6.24 (-60.89, 74.41) | -5.17 (-63.37, 53.64) | 5.78 (-44.66, 57.63) | -0.60 (-62.90, 63.32) | -1.47 (-69.65, 67.67) | -9.29 (-78.93, 63.25) | 10.64 (-58.14, 80.90) | 14.12 (-46.31, 74.81) | 1.00 (-58.99, 60.79) | Diosmectite |
| 13.80 (-40.31, 67.06) | 2.73 (-39.11, 42.52) | 13.41 (-16.87, 43.26) | 7.07 (-35.26, 48.88) | 6.14 (-47.12, 59.18) | -1.90 (-59.23, 56.75) | 18.51 (-36.62, 73.79) | 21.94 (-19.85, 64.81) | 8.36 (-33.93, 50.47) | 7.53 (-47.34, 60.81) |
| 2.30 (-51.28, 57.04) | -8.56 (-50.29, 31.57) | 2.07 (-26.50, 31.34) | -3.97 (-51.06, 41.89) | -5.15 (-58.03, 49.13) | -12.85 (-70.28, 45.70) | 7.03 (-48.88, 62.80) | 10.59 (-30.93, 54.27) | -2.78 (-45.77, 38.95) | -3.48 (-58.22, 50.02) |
| -22.60 (-89.40, 46.50) | -33.67 (-94.40, 27.91) | -23.17 (-74.90, 31.19) | -29.10 (-91.86, 35.12) | -30.25 (-98.02, 38.57) | -37.85 (-107.64, 34.95) | -17.90 (-87.36, 55.52) | -14.62 (-73.85, 48.19) | -27.77 (-86.91, 32.53) | -28.37 (-98.07, 40.42) |
| -8.88 (-58.79, 42.10) | -19.67 (-56.44, 16.80) | -8.95 (-31.73, 14.74) | -15.06 (-58.35, 27.47) | -16.20 (-66.31, 34.94) | -23.98 (-78.47, 32.41) | -3.98 (-55.28, 48.70) | -0.48 (-37.26, 36.66) | -14.00 (-51.54, 23.57) | -14.94 (-65.78, 36.07) |
| -13.42 (-101.20, 72.54) | -24.26 (-105.48, 53.55) | -13.26 (-88.22, 59.65) | -20.06 (-101.17, 62.00) | -21.04 (-108.15, 64.66) | -28.55 (-120.37, 61.91) | -8.90 (-61.99, 42.94) | -4.73 (-82.45, 70.35) | -18.58 (-99.83, 60.50) | -19.27 (-108.16, 67.11) |
| 23.68 (-23.63, 71.40) | 12.51 (-21.63, 46.30) | 23.26 (4.15, 42.15) | 17.16 (-24.03, 57.33) | 16.06 (-31.45, 65.31) | 8.08 (-44.73, 62.68) | 28.19 (-21.68, 80.09) | 31.75 (-3.69, 68.34) | 18.31 (-16.30, 52.81) | 17.41 (-31.22, 65.56) |
| 18.32 (-38.88, 77.09) | 7.38 (-39.47, 52.86) | 18.11 (-19.40, 54.95) | 11.77 (-38.87, 64.16) | 10.76 (-46.05, 69.73) | 3.14 (-57.41, 65.31) | 22.52 (-37.53, 84.26) | 26.48 (-21.04, 74.20) | 12.86 (-34.61, 60.46) | 12.07 (-44.86, 68.66) |
| -0.32 (-80.47, 81.21) | -11.87 (-84.22, 61.96) | -0.45 (-68.52, 67.87) | -6.41 (-59.13, 44.18) | -7.93 (-89.00, 72.93) | -15.53 (-99.54, 68.35) | 4.57 (-76.78, 85.38) | 7.54 (-63.98, 81.64) | -6.02 (-79.37, 66.86) | -6.96 (-87.12, 75.67) |
| -2.67 (-71.14, 65.26) | -13.75 (-71.36, 45.54) | -2.77 (-53.37, 48.34) | -8.83 (-72.51, 53.97) | -10.03 (-75.82, 57.53) | -18.21 (-89.10, 54.22) | 2.21 (-68.39, 73.94) | 5.57 (-52.32, 66.02) | -8.41 (-65.11, 51.65) | -8.51 (-75.81, 59.70) |
| -11.31 (-95.64, 72.67) | -22.59 (-98.43, 53.39) | -11.74 (-82.70, 61.38) | -18.00 (-96.26, 61.93) | -19.33 (-102.53, 65.83) | -27.01 (-116.27, 62.04) | -6.67 (-53.92, 41.44) | -3.32 (-77.49, 71.95) | -16.91 (-94.24, 61.68) | -17.37 (-102.73, 68.10) |
| -5.01 (-56.21, 46.14) | -16.02 (-51.47, 18.38) | -4.98 (-29.39, 18.10) | -11.21 (-52.26, 29.12) | -12.39 (-63.00, 39.53) | -20.31 (-75.42, 36.43) | -0.58 (-47.33, 47.98) | 3.11 (-27.44, 34.40) | -10.19 (-49.11, 28.52) | -11.27 (-63.29, 40.42) |
| -0.81 (-64.30, 63.96) | -12.17 (-64.93, 41.05) | -1.00 (-46.93, 44.17) | -7.18 (-64.49, 49.25) | -8.13 (-71.00, 54.56) | -16.41 (-83.29, 52.69) | 3.83 (-60.21, 66.49) | 7.29 (-44.85, 59.78) | -6.33 (-60.21, 47.23) | -7.06 (-71.51, 56.74) |
| 13.95 (-53.92, 82.30) | 2.57 (-58.36, 61.57) | 13.62 (-38.34, 65.86) | 7.00 (-55.47, 69.16) | 6.45 (-59.89, 75.09) | -1.34 (-72.17, 70.89) | 18.50 (-52.45, 89.74) | 21.82 (-37.45, 82.78) | 8.75 (-52.20, 67.58) | 7.68 (-61.58, 74.55) |

| | | | | | | | | | | | | |
|---|---|---|---|---|---|---|---|---|---|---|---|---|
| -13.80 (-67.06, 40.31) | -2.30 (-57.04, 51.28) | 22.60 (-46.50, 89.40) | 8.88 (-42.10, 58.79) | 13.42 (-72.54, 101.20) | -23.68 (-71.40, 23.63) | -18.32 (-77.09, 38.88) | 0.32 (-81.21, 80.47) | 2.67 (-65.26, 71.14) | 11.31 (-72.67, 95.64) | 5.01 (-46.14, 56.21) | 0.81 (-63.96, 64.30) | -13.95 (-82.30, 53.92) |
| -2.73 (-42.52, 39.11) | 8.56 (-31.57, 50.29) | 33.67 (-27.91, 94.40) | 19.67 (-16.80, 56.44) | 24.26 (-53.55, 105.48) | -12.51 (-46.30, 21.63) | -7.38 (-52.86, 39.47) | 11.87 (-61.96, 84.22) | 13.75 (-45.54, 71.36) | 22.59 (-53.39, 98.43) | 16.02 (-18.38, 51.47) | 12.17 (-41.05, 64.93) | -2.57 (-61.57, 58.36) |
| -13.41 (-43.26, 16.87) | -2.07 (-31.34, 26.50) | 23.17 (-31.19, 74.90) | 8.95 (-14.74, 31.73) | 13.26 (-59.65, 88.22) | -23.26 (-42.15, -4.15) | -18.11 (-54.95, 19.40) | 0.45 (-67.87, 68.52) | 2.77 (-48.34, 53.37) | 11.74 (-61.38, 82.70) | 4.98 (-18.10, 29.39) | 1.00 (-44.17, 46.93) | -13.62 (-65.86, 38.34) |
| -7.07 (-48.88, 35.26) | 3.97 (-41.89, 51.06) | 29.10 (-35.12, 91.86) | 15.06 (-27.47, 58.35) | 20.06 (-62.00, 101.17) | -17.16 (-57.33, 24.03) | -11.77 (-64.16, 38.87) | 6.41 (-44.18, 59.13) | 8.83 (-53.97, 72.51) | 18.00 (-61.93, 96.26) | 11.21 (-29.12, 52.26) | 7.18 (-49.25, 64.49) | -7.00 (-69.16, 55.47) |
| -6.14 (-59.18, 47.12) | 5.15 (-49.13, 58.03) | 30.25 (-38.57, 98.02) | 16.20 (-34.94, 66.31) | 21.04 (-64.66, 108.15) | -16.06 (-65.31, 31.45) | -10.76 (-69.73, 46.05) | 7.93 (-72.93, 89.00) | 10.03 (-57.53, 75.82) | 19.33 (-65.83, 102.53) | 12.39 (-39.53, 63.00) | 8.13 (-54.56, 71.00) | -6.45 (-75.09, 59.89) |
| 1.90 (-56.75, 59.23) | 12.85 (-45.70, 70.28) | 37.85 (-34.95, 107.64) | 23.98 (-32.41, 78.47) | 28.55 (-61.91, 120.37) | -8.08 (-62.68, 44.73) | -3.14 (-65.31, 57.41) | 15.53 (-68.35, 99.54) | 18.21 (-54.22, 89.10) | 27.01 (-62.04, 116.27) | 20.31 (-36.43, 75.42) | 16.41 (-52.69, 83.29) | 1.34 (-70.89, 72.17) |
| -18.51 (-73.79, 36.62) | -7.03 (-62.80, 48.88) | 17.90 (-55.52, 87.36) | 3.98 (-48.70, 55.28) | 8.90 (-42.94, 61.99) | -28.19 (-80.09, 21.68) | -22.52 (-84.26, 37.53) | -4.57 (-85.38, 76.78) | -2.21 (-73.94, 68.39) | 6.67 (-41.44, 53.92) | 0.58 (-47.98, 47.33) | -3.83 (-66.49, 60.21) | -18.50 (-89.74, 52.45) |
| -21.94 (-64.81, 19.85) | -10.59 (-54.27, 30.93) | 14.62 (-48.19, 73.85) | 0.48 (-36.66, 37.26) | 4.73 (-70.35, 82.45) | -31.75 (-68.34, 3.69) | -26.48 (-74.20, 21.04) | -7.54 (-81.64, 63.98) | -5.57 (-66.02, 52.32) | 3.32 (-71.95, 77.49) | -3.11 (-34.40, 27.44) | -7.29 (-59.78, 44.85) | -21.82 (-82.78, 37.45) |
| -8.36 (-50.47, 33.93) | 2.78 (-38.95, 45.77) | 27.77 (-32.53, 86.91) | 14.00 (-23.57, 51.54) | 18.58 (-60.50, 99.83) | -18.31 (-52.81, 16.30) | -12.86 (-60.46, 34.61) | 6.02 (-66.86, 79.37) | 8.41 (-51.65, 65.11) | 16.91 (-61.68, 94.24) | 10.19 (-28.52, 49.11) | 6.33 (-47.23, 60.21) | -8.75 (-67.58, 52.20) |
| -7.53 (-60.81, 47.34) | 3.48 (-50.02, 58.22) | 28.37 (-40.42, 98.07) | 14.94 (-36.07, 65.78) | 19.27 (-67.11, 108.16) | -17.41 (-65.56, 31.22) | -12.07 (-68.66, 44.86) | 6.96 (-75.67, 87.12) | 8.51 (-59.70, 75.81) | 17.37 (-68.10, 102.73) | 11.27 (-40.42, 63.29) | 7.06 (-56.74, 71.51) | -7.68 (-74.55, 61.58) |
| Doxycycline | 11.30 (-22.44, 43.57) | 36.17 (-19.34, 90.45) | 22.35 (-6.76, 50.25) | 27.17 (-48.19, 103.87) | -10.00 (-34.51, 13.65) | -4.72 (-44.91, 35.41) | 13.99 (-52.83, 79.24) | 16.02 (-37.84, 68.53) | 25.11 (-48.92, 97.55) | 18.39 (-9.26, 46.67) | 14.35 (-33.69, 61.84) | -0.71 (-53.33, 52.75) |
| -11.30 (-43.57, 22.44) | Glycyrrhiza | 25.07 (-30.21, 79.20) | 11.08 (-16.23, 38.16) | 15.88 (-59.61, 92.11) | -21.10 (-44.72, 2.27) | -16.00 (-56.40, 25.25) | 2.76 (-66.14, 71.12) | 5.25 (-49.45, 57.80) | 13.76 (-59.97, 87.11) | 7.30 (-21.39, 36.03) | 3.33 (-45.32, 50.78) | -11.45 (-65.01, 42.06) |
| -36.17 (-90.45, 19.34) | -25.07 (-79.20, 30.21) | Insulin-liposomal gel | -14.15 (-65.20, 39.06) | -9.53 (-95.34, 81.53) | -46.49 (-94.73, 3.77) | -40.82 (-98.20, 18.56) | -22.63 (-103.13, 59.67) | -20.12 (-87.61, 47.67) | -11.79 (-95.27, 74.71) | -17.95 (-69.46, 36.39) | -21.61 (-86.94, 44.52) | -36.23 (-104.82, 33.91) |
| -22.35 (-50.25, 6.76) | -11.08 (-38.16, 16.23) | 14.15 (-39.06, 65.20) | Laser | 4.53 (-67.96, 79.56) | -32.21 (-48.08, -16.39) | -26.83 (-62.81, 8.51) | -8.44 (-74.54, 58.20) | -6.07 (-56.28, 43.35) | 2.78 (-68.75, 72.64) | -3.74 (-23.22, 16.50) | -7.81 (-51.89, 35.95) | -22.46 (-73.28, 28.18) |
| -27.17 (-103.87, 48.19) | -15.88 (-92.11, 59.61) | 9.53 (-81.53, 95.34) | -4.53 (-79.56, 67.96) | N-acetylcysteine | -36.75 (-111.29, 35.20) | -31.86 (-112.07, 47.24) | -13.12 (-107.24, 82.42) | -10.61 (-99.37, 76.63) | -2.61 (-71.77, 68.59) | -8.35 (-79.41, 61.37) | -12.42 (-96.10, 69.89) | -27.04 (-117.14, 61.20) |
| 10.00 (-13.65, 34.51) | 21.10 (-2.27, 44.72) | 46.49 (-3.77, 94.73) | 32.21 (16.39, 48.08) | 36.75 (-35.20, 111.29) | Placebo | 5.25 (-26.72, 37.76) | 23.62 (-41.49, 88.72) | 26.05 (-21.91, 73.10) | 34.80 (-35.11, 104.24) | 28.45 (10.36, 46.76) | 24.69 (-17.58, 66.07) | 9.53 (-38.55, 58.13) |
| 4.72 (-35.41, 44.91) | 16.00 (-25.25, 56.40) | 40.82 (-18.56, 98.20) | 26.83 (-8.51, 62.81) | 31.86 (-47.24, 112.07) | -5.25 (-37.76, 26.72) | Probiotics | 18.81 (-52.26, 89.91) | 20.77 (-35.98, 77.43) | 30.06 (-49.02, 106.89) | 22.94 (-13.29, 60.68) | 19.27 (-33.74, 72.05) | 4.34 (-53.30, 63.20) |
| -13.99 (-79.24, 52.83) | -2.76 (-71.12, 66.14) | 22.63 (-59.67, 103.13) | 8.44 (-58.20, 74.54) | 13.12 (-82.42, 107.24) | -23.62 (-88.72, 41.49) | -18.81 (-89.91, 52.26) | Quercetin | 2.21 (-77.47, 82.40) | 10.98 (-84.23, 105.86) | 4.67 (-60.23, 70.61) | 0.14 (-76.59, 79.24) | -14.43 (-94.23, 67.61) |
| -16.02 (-68.53, 37.84) | -5.25 (-57.80, 49.45) | 20.12 (-47.67, 87.61) | 6.07 (-43.35, 56.28) | 10.61 (-76.63, 99.37) | -26.05 (-73.10, 21.91) | -20.77 (-77.43, 35.98) | -2.21 (-82.40, 77.47) | Silver nitrate | 9.20 (-76.87, 94.61) | 2.52 (-47.78, 53.88) | -1.40 (-64.41, 61.75) | -16.32 (-83.45, 51.86) |
| -25.11 (-97.55, 48.92) | -13.76 (-87.11, 59.97) | 11.79 (-74.71, 95.27) | -2.78 (-72.64, 68.75) | 2.61 (-68.59, 71.77) | -34.80 (-104.24, 35.11) | -30.06 (-106.89, 49.02) | -10.98 (-105.86, 84.23) | -9.20 (-94.61, 76.87) | Sucralfate | -6.49 (-75.01, 61.48) | -10.67 (-89.62, 69.89) | -25.44 (-110.44, 61.69) |
| -18.39 (-46.67, 9.26) | -7.30 (-36.03, 21.39) | 17.95 (-36.39, 69.46) | 3.74 (-16.50, 23.22) | 8.35 (-61.37, 79.41) | -28.45 (-46.76, -10.36) | -22.94 (-60.68, 13.29) | -4.67 (-70.61, 60.23) | -2.52 (-53.88, 47.78) | 6.49 (-61.48, 75.01) | Triamcinolone | -4.01 (-46.35, 37.87) | -18.74 (-70.98, 32.54) |
| -14.35 (-61.84, 33.69) | -3.33 (-50.78, 45.32) | 21.61 (-44.52, 86.94) | 7.81 (-35.95, 51.89) | 12.42 (-69.89, 96.10) | -24.69 (-66.07, 17.58) | -19.27 (-72.05, 33.74) | -0.14 (-79.24, 76.59) | 1.40 (-61.75, 64.41) | 10.67 (-69.89, 89.62) | 4.01 (-37.87, 46.35) | Triester Glycerol Oxide | -14.62 (-77.79, 48.99) |
| 0.71 (-52.75, 53.33) | 11.45 (-42.06, 65.01) | 36.23 (-33.91, 104.82) | 22.46 (-28.18, 73.28) | 27.04 (-61.20, 117.14) | -9.53 (-58.13, 38.55) | -4.34 (-63.20, 53.30) | 14.43 (-67.61, 94.23) | 16.32 (-51.86, 83.45) | 25.44 (-61.69, 110.44) | 18.74 (-32.54, 70.98) | 14.62 (-48.99, 77.79) | Zinc |

## Rank probability

**Rank 1 is best, rank N is worst.**

| Drug | Rank 1 | Rank 2 | Rank 3 | Rank 4 | Rank 5 | Rank 6 | Rank 7 | Rank 8 | Rank 9 | Rank 10 | Rank 11 | Rank 12 | Rank 13 | Rank 14 | Rank 15 | Rank 16 | Rank 17 |
|---|---|---|---|---|---|---|---|---|---|---|---|---|---|---|---|---|---|
| Allicin | 0.05 | 0.06 | 0.05 | 0.05 | 0.05 | 0.05 | 0.04 | 0.04 | 0.04 | 0.04 | 0.04 | 0.04 | 0.04 | 0.04 | 0.04 | 0.04 | 0.04 |
| Aloe | 0 | 0.01 | 0.01 | 0.02 | 0.02 | 0.02 | 0.03 | 0.03 | 0.04 | 0.04 | 0.04 | 0.05 | 0.06 | 0.06 | 0.07 | 0.08 | 0.07 |
| Amlexanox | 0 | 0.01 | 0.01 | 0.02 | 0.04 | 0.05 | 0.06 | 0.07 | 0.08 | 0.1 | 0.1 | 0.1 | 0.09 | 0.08 | 0.06 | 0.05 | 0.04 |
| Benzydamine | 0.01 | 0.02 | 0.03 | 0.03 | 0.04 | 0.04 | 0.04 | 0.04 | 0.04 | 0.05 | 0.05 | 0.05 | 0.05 | 0.05 | 0.06 | 0.05 | 0.06 |
| Berberine gelatin | 0.03 | 0.03 | 0.04 | 0.04 | 0.04 | 0.04 | 0.03 | 0.04 | 0.03 | 0.04 | 0.04 | 0.04 | 0.04 | 0.04 | 0.05 | 0.05 | 0.05 |
| Chitosan | 0.02 | 0.03 | 0.03 | 0.03 | 0.03 | 0.03 | 0.03 | 0.03 | 0.03 | 0.02 | 0.03 | 0.03 | 0.04 | 0.04 | 0.04 | 0.04 | 0.05 |
| Chlorhexidine | 0.01 | 0.06 | 0.1 | 0.08 | 0.07 | 0.06 | 0.04 | 0.04 | 0.04 | 0.04 | 0.04 | 0.04 | 0.04 | 0.04 | 0.04 | 0.03 | 0.04 |
| Curcumin | 0.04 | 0.07 | 0.08 | 0.09 | 0.08 | 0.07 | 0.07 | 0.06 | 0.06 | 0.05 | 0.05 | 0.05 | 0.04 | 0.04 | 0.03 | 0.03 | 0.03 |
| Dexamethasone | 0.01 | 0.02 | 0.03 | 0.04 | 0.04 | 0.04 | 0.04 | 0.04 | 0.05 | 0.05 | 0.05 | 0.06 | 0.05 | 0.06 | 0.06 | 0.06 | 0.06 |
| Diosmectite | 0.03 | 0.04 | 0.04 | 0.04 | 0.04 | 0.04 | 0.04 | 0.04 | 0.03 | 0.04 | 0.04 | 0.04 | 0.04 | 0.04 | 0.05 | 0.04 | 0.05 |
| Doxycycline | 0 | 0 | 0 | 0.01 | 0.01 | 0.01 | 0.01 | 0.02 | 0.02 | 0.03 | 0.04 | 0.05 | 0.06 | 0.07 | 0.08 | 0.09 | 0.1 |
| Glycyrrhiza | 0 | 0.01 | 0.02 | 0.03 | 0.04 | 0.05 | 0.05 | 0.06 | 0.06 | 0.07 | 0.08 | 0.08 | 0.08 | 0.08 | 0.07 | 0.07 | 0.05 |
| Insulin-liposomal gel | 0.24 | 0.15 | 0.1 | 0.08 | 0.06 | 0.04 | 0.04 | 0.04 | 0.03 | 0.03 | 0.02 | 0.02 | 0.02 | 0.02 | 0.02 | 0.02 | 0.01 |
| Laser | 0.01 | 0.03 | 0.05 | 0.09 | 0.11 | 0.12 | 0.12 | 0.11 | 0.1 | 0.08 | 0.06 | 0.05 | 0.03 | 0.02 | 0.01 | 0.01 | 0 |
| N-acetylcysteine | 0.18 | 0.12 | 0.08 | 0.06 | 0.05 | 0.04 | 0.03 | 0.03 | 0.02 | 0.03 | 0.02 | 0.02 | 0.03 | 0.03 | 0.03 | 0.03 | 0.02 |
| Placebo | 0 | 0 | 0 | 0 | 0 | 0 | 0 | 0 | 0 | 0 | 0 | 0 | 0 | 0.01 | 0.02 | 0.04 | 0.08 |
| Probiotics | 0 | 0 | 0 | 0.01 | 0.01 | 0.01 | 0.02 | 0.01 | 0.02 | 0.03 | 0.03 | 0.03 | 0.04 | 0.05 | 0.06 | 0.07 | 0.07 |
| Quercetin | 0.1 | 0.07 | 0.06 | 0.05 | 0.04 | 0.04 | 0.03 | 0.03 | 0.03 | 0.03 | 0.03 | 0.03 | 0.03 | 0.03 | 0.03 | 0.04 | 0.03 |
| Silver nitrate | 0.06 | 0.07 | 0.06 | 0.06 | 0.05 | 0.05 | 0.04 | 0.04 | 0.04 | 0.04 | 0.04 | 0.04 | 0.04 | 0.04 | 0.04 | 0.04 | 0.04 |
| Sucralfate | 0.15 | 0.12 | 0.08 | 0.06 | 0.05 | 0.04 | 0.03 | 0.03 | 0.03 | 0.03 | 0.03 | 0.03 | 0.03 | 0.03 | 0.03 | 0.03 | 0.03 |
| Triamcinolone | 0 | 0.01 | 0.02 | 0.04 | 0.06 | 0.09 | 0.12 | 0.12 | 0.12 | 0.11 | 0.1 | 0.08 | 0.05 | 0.04 | 0.02 | 0.01 | 0.01 |
| Triester Glycerol Oxide | 0.03 | 0.05 | 0.06 | 0.06 | 0.06 | 0.05 | 0.05 | 0.05 | 0.05 | 0.05 | 0.04 | 0.05 | 0.05 | 0.05 | 0.05 | 0.05 | 0.04 |
| Zinc | 0.02 | 0.02 | 0.03 | 0.03 | 0.03 | 0.03 | 0.03 | 0.03 | 0.03 | 0.03 | 0.03 | 0.04 | 0.04 | 0.04 | 0.05 | 0.05 | 0.05 |

| Rank 18 | Rank 19 | Rank 20 | Rank 21 | Rank 22 | Rank 23 |
|---|---|---|---|---|---|
| 0.04 | 0.04 | 0.04 | 0.04 | 0.04 | 0.04 |
| 0.07 | 0.06 | 0.06 | 0.06 | 0.06 | 0.05 |
| 0.02 | 0.01 | 0.01 | 0 | 0 | 0 |
| 0.05 | 0.05 | 0.04 | 0.05 | 0.05 | 0.03 |
| 0.05 | 0.05 | 0.05 | 0.05 | 0.07 | 0.08 |
| 0.05 | 0.05 | 0.06 | 0.06 | 0.09 | 0.16 |
| 0.03 | 0.03 | 0.03 | 0.04 | 0.03 | 0.01 |
| 0.02 | 0.02 | 0.01 | 0.01 | 0.01 | 0 |
| 0.05 | 0.05 | 0.04 | 0.04 | 0.04 | 0.03 |
| 0.05 | 0.05 | 0.04 | 0.05 | 0.06 | 0.07 |
| 0.1 | 0.09 | 0.08 | 0.06 | 0.04 | 0.02 |
| 0.04 | 0.03 | 0.02 | 0.01 | 0.01 | 0 |
| 0.01 | 0.01 | 0.01 | 0.01 | 0.01 | 0.01 |
| 0 | 0 | 0 | 0 | 0 | 0 |
| 0.02 | 0.02 | 0.03 | 0.03 | 0.04 | 0.05 |
| 0.13 | 0.19 | 0.21 | 0.18 | 0.1 | 0.03 |
| 0.07 | 0.08 | 0.08 | 0.09 | 0.11 | 0.09 |
| 0.03 | 0.03 | 0.03 | 0.04 | 0.06 | 0.08 |
| 0.04 | 0.03 | 0.03 | 0.03 | 0.04 | 0.04 |
| 0.02 | 0.02 | 0.03 | 0.03 | 0.04 | 0.05 |
| 0 | 0 | 0 | 0 | 0 | 0 |
| 0.04 | 0.03 | 0.03 | 0.03 | 0.03 | 0.03 |
| 0.05 | 0.05 | 0.06 | 0.07 | 0.09 | 0.13 |

## Consistency check

| Parameter | Median (95% CI) |
|---|---|
| Random Effects Standard Deviation | 23.46 (18.29, 31.60) |

## Convergence Diagnostics

Convergence is assessed using the Brooks-Gelman-Rubin method. This method compares within-chain and between-chain variance to calculate the Potential Scale Reduction Factor (PSRF). A PSRF close to one indicates approximate convergence has been reached.

| Parameter | PSRF |
|---|---|
| d.Benzydamine.Quercetin | 1.00 |
| d.Chlorhexidine.Nacetylcysteine | 1.00 |
| d.Chlorhexidine.Sucralfate | 1.00 |
| d.Placebo.Allicin | 1.00 |
| d.Placebo.Berberinegelatin | 1.00 |
| d.Placebo.Chitosan | 1.00 |
| d.Placebo.Dexamethasone | 1.00 |
| d.Placebo.Diosmectite | 1.00 |
| d.Placebo.Glycyrrhiza | 1.00 |
| d.Placebo.Insulinliposomlgel | 1.05 |
| d.Placebo.Probiotics | 1.00 |
| d.Placebo.Silvernitrate | 1.00 |
| d.Placebo.Zinc | 1.01 |
| d.Triamcinolone.Aloe | 1.00 |
| d.Triamcinolone.Amlexanox | 1.00 |
| d.Triamcinolone.Benzydamine | 1.01 |
| d.Triamcinalone.Chlorhexidine | 1.00 |
| d.Triamcinolone.Curcumin | 1.00 |
| d.Triamcinolone.Doxycycline | 1.01 |
| d.Triamcinolone.Laser | 1.01 |
| d.Triamcinolone.Placebo | 1.01 |
| d.Triamcinolone. TriesterGlycerolOxide | 1.00 |
| sd.d | 1.01 |

Number of chains : 4
Tuning iterations : 20,000
Simulation iterations : 50,000
Thinning interval : 10
Inference samples : 10,000
Variance scaling factor : 2.5

## 2.Inconsistency Model

### Summary estimates for Symptom-reducing effect

The estimate values for symptom-reducing effect are given as mean difference (MD) and 95% confidence interval (CI). The table shows the pooled estimates based on the network meta-analysis during the treatment period for each study.

| | | | | | | | | | |
|---|---|---|---|---|---|---|---|---|---|
| Allicin | -10.84 (-69.10, 50.20) | -3.21 (-57.33, 50.76) | -4.71 (-70.35, 58.91) | -7.83 (-75.89, 58.71) | -15.61 (-85.98, 57.56) | 4.96 (-68.80, 75.96) | 7.80 (-53.99, 70.81) | -6.94 (-68.99, 54.83) | -6.94 (-73.38, 61.21) |
| 10.84 (-50.20, 69.10) | Aloe | 7.65 (-36.92, 47.18) | 5.67 (-47.01, 57.73) | 2.14 (-57.67, 60.36) | -4.82 (-68.21, 57.14) | 15.53 (-46.07, 74.54) | 18.94 (-29.85, 66.80) | 3.73 (-48.70, 52.89) | 3.57 (-55.85, 61.47) |
| 3.21 (-50.76, 57.33) | -7.65 (-47.18, 36.92) | Amlexanox | -2.12 (-49.37, 48.27) | -4.95 (-57.36, 49.75) | -12.29 (-69.87, 47.60) | 7.64 (-46.74, 63.92) | 11.53 (-30.11, 54.43) | -3.87 (-41.11, 33.13) | -3.78 (-56.78, 50.54) |
| 4.71 (-58.91, 70.35) | -5.67 (-57.73, 47.01) | 2.12 (-48.27, 49.37) | Benzydamine | -3.48 (-67.16, 61.18) | -10.72 (-77.50, 58.38) | 9.37 (-54.48, 74.09) | 13.30 (-39.72, 65.46) | -2.18 (-60.05, 55.07) | -2.36 (-65.57, 62.97) |
| 7.83 (-58.71, 75.89) | -2.14 (-60.36, 57.67) | 4.95 (-49.75, 57.36) | 3.48 (-61.18, 67.16) | Berberine gelatin | -6.94 (-79.37, 64.17) | 13.35 (-60.27, 83.34) | 16.78 (-44.78, 78.27) | 1.70 (-62.33, 62.06) | 1.52 (-66.58, 69.05) |
| 15.61 (-57.56, 85.98) | 4.82 (-57.14, 68.21) | 12.29 (-47.60, 69.87) | 10.72 (-58.38, 77.50) | 6.94 (-64.17, 79.37) | Chitosan | 19.89 (-54.75, 94.54) | 23.72 (-42.32, 89.45) | 8.81 (-59.25, 72.21) | 8.34 (-63.66, 82.62) |
| -4.96 (-75.96, 68.80) | -15.53 (-74.54, 46.07) | -7.64 (-63.92, 46.74) | -9.37 (-74.09, 54.48) | -13.35 (-83.34, 60.27) | -19.89 (-94.54, 54.75) | Chlorhexidine | 3.52 (-53.88, 62.45) | -11.25 (-75.68, 53.45) | -11.44 (-82.41, 59.12) |
| -7.80 (-70.81, 53.99) | -18.94 (-66.80, 29.85) | -11.53 (-54.43, 30.11) | -13.30 (-65.46, 39.72) | -16.78 (-78.27, 44.78) | -23.72 (-89.45, 42.32) | -3.52 (-62.45, 53.88) | Curcumin | -15.11 (-68.29, 36.79) | -15.35 (-76.98, 47.37) |
| 6.94 (-54.83, 68.99) | -3.73 (-52.89, 48.70) | 3.87 (-33.13, 41.11) | 2.18 (-55.07, 60.05) | -1.70 (-62.06, 62.33) | -8.81 (-72.21, 59.25) | 11.25 (-53.45, 75.68) | 15.11 (-36.79, 68.29) | Dexamethasone | 0.31 (-60.69, 61.90) |
| 6.94 (-61.21, 73.38) | -3.57 (-61.47, 55.85) | 3.78 (-50.54, 56.78) | 2.36 (-62.97, 65.57) | -1.52 (-69.05, 66.58) | -8.34 (-82.62, 63.66) | 11.44 (-59.12, 82.41) | 15.35 (-47.37, 76.98) | -0.31 (-61.90, 60.69) | Diosmectite |
| 11.14 (-45.36, 67.45) | 0.56 (-43.38, 44.11) | 9.31 (-33.45, 43.43) | 6.61 (-36.97, 49.54) | 3.13 (-53.47, 57.95) | -3.96 (-65.10, 55.64) | 16.31 (-41.61, 72.42) | 19.72 (-25.89, 64.18) | 4.91 (-46.52, 50.90) | 4.74 (-52.57, 60.00) |
| 8.25 (-49.84, 68.77) | -3.20 (-45.49, 47.97) | 4.51 (-27.48, 37.91) | 2.58 (-50.52, 60.35) | -0.49 (-56.71, 59.60) | -7.55 (-67.96, 56.24) | 12.26 (-46.36, 74.82) | 15.81 (-31.01, 67.79) | 0.88 (-45.15, 48.58) | 1.11 (-55.05, 61.76) |
| -25.97 (-96.46, 41.84) | -36.48 (-94.79, 22.07) | -29.05 (-83.08, 23.77) | -30.76 (-95.42, 32.60) | -34.21 (-101.22, 33.48) | -41.64 (-113.55, 29.85) | -21.27 (-92.90, 51.72) | -17.27 (-78.93, 43.69) | -32.72 (-95.84, 29.13) | -32.83 (-101.93, 34.79) |
| -8.39 (-62.92, 46.32) | -19.00 (-57.23, 22.94) | -11.60 (-40.94, 15.85) | -13.50 (-59.35, 35.61) | -16.91 (-70.11, 38.30) | -24.32 (-79.39, 34.82) | -3.96 (-57.90, 53.07) | -0.09 (-40.46, 41.52) | -15.38 (-58.42, 27.29) | -15.63 (-68.57, 39.35) |
| -14.27 (-102.11, 75.83) | -24.69 (-103.55, 55.35) | -17.64 (-94.08, 59.08) | -18.96 (-102.97, 63.75) | -22.41 (-109.28, 66.61) | -29.70 (-123.46, 61.17) | -9.42 (-61.60, 41.88) | -5.90 (-84.71, 71.26) | -21.26 (-104.34, 61.11) | -21.57 (-109.36, 65.36) |
| 24.54 (-24.76, 71.69) | 13.46 (-18.86, 48.45) | 24.04 (3.93, 44.21) | 17.90 (-29.20, 65.23) | 16.10 (-31.59, 63.34) | 8.40 (-43.59, 61.76) | 28.93 (-24.99, 81.27) | 32.48 (-5.89, 71.20) | 17.31 (-21.51, 54.30) | 17.04 (-31.13, 64.24) |
| 19.02 (-39.73, 76.27) | 8.53 (-38.68, 55.04) | 15.32 (-25.26, 55.88) | 13.80 (-39.81, 67.29) | 10.58 (-47.63, 67.92) | 3.06 (-57.55, 64.65) | 23.24 (-38.89, 86.28) | 27.00 (-22.75, 77.35) | 11.56 (-40.09, 63.30) | 12.04 (-45.68, 69.86) |
| -2.65 (-84.95, 80.63) | -13.16 (-87.34, 59.57) | -5.86 (-77.99, 64.84) | -7.62 (-59.16, 42.49) | -10.88 (-94.20, 70.67) | -18.02 (-103.64, 66.29) | 1.69 (-81.37, 83.14) | 5.81 (-68.05, 78.58) | -9.81 (-89.23, 67.01) | -9.39 (-91.30, 73.81) |
| -1.67 (-69.95, 65.12) | -13.14 (-70.88, 46.15) | -5.25 (-59.15, 47.81) | -6.54 (-72.36, 56.95) | -10.52 (-77.34, 59.02) | -17.86 (-88.11, 52.92) | 2.43 (-69.77, 75.12) | 5.93 (-55.13, 67.51) | -8.92 (-70.36, 52.78) | -9.19 (-76.01, 60.16) |
| -11.24 (-96.85, 75.84) | -21.54 (-98.04, 56.24) | -13.88 (-87.25, 58.49) | -15.46 (-95.68, 64.44) | -19.33 (-104.30, 68.82) | -25.85 (-114.71, 62.04) | -6.24 (-53.97, 41.36) | -2.59 (-77.35, 72.23) | -18.07 (-99.11, 61.80) | -17.77 (-104.16, 69.26) |
| -4.99 (-59.79, 48.42) | -15.82 (-52.08, 21.21) | -8.02 (-38.22, 19.06) | -10.28 (-52.12, 31.53) | -13.19 (-66.27, 38.73) | -20.89 (-77.56, 37.36) | -0.59 (-48.34, 47.98) | 3.10 (-28.65, 33.83) | -11.86 (-55.67, 29.06) | -12.05 (-64.43, 41.15) |
| -2.60 (-69.77, 62.74) | -13.53 (-67.14, 43.22) | -5.99 (-57.32, 42.50) | -8.21 (-64.73, 49.53) | -11.24 (-76.87, 55.02) | -18.17 (-86.93, 51.74) | 2.22 (-64.37, 64.60) | 5.55 (-47.69, 59.17) | -9.93 (-68.83, 47.71) | -9.54 (-75.66, 56.38) |
| 8.04 (-63.65, 77.79) | -2.20 (-64.59, 58.69) | 5.22 (-54.60, 59.56) | 3.39 (-64.98, 69.26) | 0.46 (-71.04, 71.60) | -7.56 (-81.56, 65.87) | 13.26 (-61.65, 85.70) | 16.58 (-50.56, 80.51) | 1.30 (-64.86, 64.17) | 1.13 (-72.30, 72.04) |

| | | | | | | | | | | | | |
|---|---|---|---|---|---|---|---|---|---|---|---|---|
| -11.14 (-67.45, 45.36) | -8.25 (-68.77, 49.84) | 25.97 (-41.84, 96.46) | 8.39 (-46.32, 62.92) | 14.27 (-75.83, 102.11) | -24.54 (-71.69, 24.76) | -19.02 (-76.27, 39.73) | 2.65 (-80.63, 84.95) | 1.67 (-65.12, 69.95) | 11.24 (-75.84, 96.85) | 4.99 (-48.42, 59.79) | 2.60 (-62.74, 69.77) | -8.04 (-77.79, 63.65) |
| -0.56 (-44.11, 43.38) | 3.20 (-47.97, 45.49) | 36.48 (-22.07, 94.79) | 19.00 (-22.94, 57.23) | 24.69 (-55.35, 103.55) | -13.46 (-48.45, 18.86) | -8.53 (-55.04, 38.68) | 13.16 (-59.57, 87.34) | 13.14 (-46.15, 70.88) | 21.54 (-56.24, 98.04) | 15.82 (-21.21, 52.08) | 13.53 (-43.22, 67.14) | 2.20 (-58.69, 64.59) |
| -9.31 (-43.43, 33.45) | -4.51 (-37.91, 27.48) | 29.05 (-23.77, 83.08) | 11.60 (-15.85, 40.94) | 17.64 (-59.08, 94.08) | -24.04 (-44.21, -3.93) | -15.32 (-55.88, 25.26) | 5.86 (-64.84, 77.99) | 5.25 (-47.81, 59.15) | 13.88 (-58.49, 87.25) | 8.02 (-19.06, 38.22) | 5.99 (-42.50, 57.32) | -5.22 (-59.56, 54.60) |
| -6.61 (-49.54, 36.97) | -2.58 (-60.35, 50.52) | 30.76 (-32.60, 95.42) | 13.50 (-35.61, 59.35) | 18.96 (-63.75, 102.97) | -17.90 (-65.23, 29.20) | -13.80 (-67.29, 39.81) | 7.62 (-42.49, 59.16) | 6.54 (-56.95, 72.36) | 15.46 (-64.44, 95.68) | 10.28 (-31.53, 52.12) | 8.21 (-49.53, 64.73) | -3.39 (-69.26, 64.98) |
| -3.13 (-57.95, 53.47) | 0.49 (-59.60, 56.71) | 34.21 (-33.48, 101.22) | 16.91 (-38.30, 70.11) | 22.41 (-66.61, 109.28) | -16.10 (-63.34, 31.59) | -10.58 (-67.92, 47.63) | 10.88 (-70.67, 94.20) | 10.52 (-59.02, 77.34) | 19.33 (-68.82, 104.30) | 13.19 (-38.73, 66.27) | 11.24 (-55.02, 76.87) | -0.46 (-71.60, 71.04) |
| 3.96 (-55.64, 65.10) | 7.55 (-56.24, 67.96) | 41.64 (-29.85, 113.55) | 24.32 (-34.82, 79.39) | 29.70 (-61.17, 123.46) | -8.40 (-61.76, 43.59) | -3.06 (-64.65, 57.55) | 18.02 (-66.29, 103.64) | 17.86 (-52.92, 88.11) | 25.85 (-62.04, 114.71) | 20.89 (-37.36, 77.56) | 18.17 (-51.74, 86.93) | 7.56 (-65.87, 81.56) |
| -16.31 (-72.42, 41.61) | -12.26 (-74.82, 46.36) | 21.27 (-51.72, 92.90) | 3.96 (-53.07, 57.90) | 9.42 (-41.88, 61.60) | -28.93 (-81.27, 24.99) | -23.24 (-86.28, 38.89) | -1.69 (-83.14, 81.37) | -2.43 (-75.12, 69.77) | 6.24 (-41.36, 53.97) | 0.59 (-47.98, 48.34) | -2.22 (-64.60, 64.37) | -13.26 (-85.70, 61.65) |
| -19.72 (-64.18, 25.89) | -15.81 (-67.79, 31.01) | 17.27 (-43.69, 78.93) | 0.09 (-41.52, 40.46) | 5.90 (-71.26, 84.71) | -32.48 (-71.20, 5.89) | -27.00 (-77.35, 22.75) | -5.81 (-78.58, 68.05) | -5.93 (-67.51, 55.13) | 2.59 (-72.23, 77.35) | -3.10 (-33.83, 28.65) | -5.55 (-59.17, 47.69) | -16.58 (-80.51, 50.56) |
| -4.91 (-50.90, 46.52) | -0.88 (-48.58, 45.15) | 32.72 (-29.13, 95.84) | 15.38 (-27.29, 58.42) | 21.26 (-61.11, 104.34) | -17.31 (-54.30, 21.51) | -11.56 (-63.30, 40.09) | 9.81 (-67.01, 89.23) | 8.92 (-52.78, 70.36) | 18.07 (-61.80, 99.11) | 11.86 (-29.06, 55.67) | 9.93 (-47.71, 68.83) | -1.30 (-64.17, 64.86) |
| -4.74 (-60.00, 52.57) | -1.11 (-61.76, 55.05) | 32.83 (-34.79, 101.93) | 15.63 (-39.35, 68.57) | 21.57 (-65.36, 109.36) | -17.04 (-64.24, 31.13) | -12.04 (-69.86, 45.68) | 9.39 (-73.81, 91.30) | 9.19 (-60.16, 76.01) | 17.77 (-69.26, 104.16) | 12.05 (-41.15, 64.43) | 9.54 (-56.38, 75.66) | -1.13 (-72.04, 72.30) |
| Doxycycline | 4.53 (-48.56, 43.16) | 37.06 (-20.12, 94.46) | 20.61 (-21.14, 53.27) | 25.50 (-52.19, 102.96) | -9.58 (-34.51, 14.95) | -6.98 (-53.10, 34.94) | 13.98 (-52.58, 79.83) | 13.47 (-43.71, 68.31) | 22.17 (-53.04, 95.71) | 16.89 (-16.44, 47.63) | 14.24 (-37.38, 63.98) | 3.37 (-55.83, 63.59) |
| -4.53 (-43.16, 48.56) | Glycyrrhiza | 33.96 (-23.04, 93.97) | 16.14 (-14.70, 51.70) | 22.03 (-58.39, 103.87) | -22.12 (-46.63, 1.73) | -11.24 (-53.89, 39.05) | 10.57 (-61.89, 87.94) | 9.70 (-45.30, 68.93) | 18.58 (-56.57, 96.99) | 12.75 (-21.26, 54.72) | 10.52 (-43.49, 67.56) | -0.24 (-58.86, 61.48) |
| -37.06 (-94.46, 20.12) | -33.96 (-93.97, 23.04) | Insulin-liposomal gel | -17.47 (-71.14, 35.45) | -12.01 (-100.86, 77.23) | -50.04 (-97.82, -1.73) | -44.34 (-101.74, 13.09) | -23.06 (-104.43, 58.88) | -23.67 (-91.87, 43.98) | -15.43 (-101.63, 71.34) | -20.70 (-73.83, 32.26) | -23.29 (-89.19, 42.18) | -33.84 (-103.21, 37.30) |
| -20.61 (-53.27, 21.14) | -16.14 (-51.70, 14.70) | 17.47 (-35.45, 71.14) | Laser | 5.75 (-69.66, 83.06) | -32.21 (-48.76, -14.99) | -27.17 (-66.13, 13.85) | -6.06 (-74.23, 64.81) | -5.97 (-59.51, 47.66) | 2.29 (-71.25, 76.19) | -3.73 (-26.00, 19.54) | -6.09 (-53.37, 43.88) | -16.42 (-72.94, 42.22) |
| -25.50 (-102.96, 52.19) | -22.03 (-103.87, 58.39) | 12.01 (-77.23, 100.86) | -5.75 (-83.06, 69.66) | N-acetylcysteine | -38.05 (-113.06, 36.44) | -32.74 (-115.57, 49.29) | -11.87 (-109.91, 85.66) | -11.70 (-102.69, 76.49) | -3.32 (-74.03, 64.08) | -8.71 (-79.51, 61.27) | -11.66 (-94.09, 72.82) | -22.19 (-111.94, 69.08) |
| 9.58 (-14.95, 34.51) | 22.12 (-1.73, 46.63) | 50.04 (1.73, 97.82) | 32.21 (14.99, 48.76) | 38.05 (-36.44, 113.06) | Placebo | 5.60 (-27.57, 37.30) | 26.64 (-39.35, 94.56) | 26.14 (-21.64, 73.85) | 34.62 (-36.58, 106.68) | 28.24 (-1.97, 58.52) | 25.78 (-24.25, 74.93) | 16.00 (-34.63, 68.34) |
| 6.98 (-34.94, 53.10) | 11.24 (-39.05, 53.89) | 44.34 (-13.09, 101.74) | 27.17 (-13.85, 66.13) | 32.74 (-49.29, 115.57) | -5.60 (-37.30, 27.57) | Probiotics | 21.27 (-50.85, 94.45) | 20.99 (-36.91, 77.64) | 28.98 (-48.15, 109.88) | 23.84 (-14.70, 63.62) | 21.50 (-34.48, 77.36) | 10.87 (-49.60, 72.12) |
| -13.98 (-79.83, 52.58) | -10.57 (-87.94, 61.89) | 23.06 (-58.88, 104.43) | 6.06 (-64.81, 74.23) | 11.87 (-85.66, 109.91) | -26.64 (-94.56, 39.35) | -21.27 (-94.45, 50.85) | Quercetin | -0.99 (-82.58, 80.53) | 7.93 (-86.56, 101.61) | 2.64 (-63.52, 67.51) | 0.24 (-76.53, 76.66) | -10.94 (-96.28, 74.37) |
| -13.47 (-68.31, 43.71) | -9.70 (-68.93, 45.30) | 23.67 (-43.98, 91.87) | 5.97 (-47.66, 59.51) | 11.70 (-76.49, 102.69) | -26.14 (-73.85, 21.64) | -20.99 (-77.64, 36.91) | 0.99 (-80.53, 82.58) | Silver nitrate | 8.95 (-77.81, 95.52) | 3.34 (-49.41, 55.96) | 0.60 (-65.13, 66.60) | -10.23 (-80.40, 60.74) |
| -22.17 (-95.71, 53.04) | -18.58 (-96.99, 56.57) | 15.43 (-71.34, 101.63) | -2.29 (-76.19, 71.25) | 3.32 (-64.08, 74.03) | -34.62 (-106.68, 36.58) | -28.98 (-109.88, 48.15) | -7.93 (-101.61, 86.56) | -8.95 (-95.52, 77.81) | Sucralfate | -5.41 (-73.59, 62.75) | -8.07 (-88.35, 71.56) | -19.04 (-108.64, 69.73) |
| -16.89 (-47.63, 16.44) | -12.75 (-54.72, 21.26) | 20.70 (-32.26, 73.83) | 3.73 (-19.54, 26.00) | 8.71 (-61.27, 79.51) | -28.24 (-58.52, 1.97) | -23.84 (-63.62, 14.70) | -2.64 (-67.51, 63.52) | -3.34 (-55.96, 49.41) | 5.41 (-62.75, 73.59) | Triamcinolone | -2.49 (-46.23, 40.75) | -13.52 (-69.10, 44.29) |
| -14.24 (-63.98, 37.38) | -10.52 (-67.56, 43.49) | 23.29 (-42.18, 89.19) | 6.09 (-43.88, 53.37) | 11.66 (-72.82, 94.09) | -25.78 (-74.93, 24.25) | -21.50 (-77.36, 34.48) | -0.24 (-76.66, 76.53) | -0.60 (-66.60, 65.13) | 8.07 (-71.56, 88.35) | 2.49 (-40.75, 46.23) | Triester Glycerol Oxide | -10.70 (-78.76, 60.83) |
| -3.37 (-63.59, 55.83) | 0.24 (-61.48, 58.86) | 33.84 (-37.30, 103.21) | 16.42 (-42.22, 72.94) | 22.19 (-69.08, 111.94) | -16.00 (-68.34, 34.63) | -10.87 (-72.12, 49.60) | 10.94 (-74.37, 96.28) | 10.23 (-60.74, 80.40) | 19.04 (-69.73, 108.64) | 13.52 (-44.29, 69.10) | 10.70 (-60.83, 78.76) | Zinc |

## Inconsistency Factors

| Cycle | Median (95% CI) |
|---|---|
| Aloe, Amlexanox, Placebo, Triamcinolone | 0.50 (-14.31, 31.47) |
| Aloe, Amlexanox, Dexamethasone, Placebo, Triamcinolone | 0.05 (-25.55, 26.18) |
| Aloe, Doxycycline, Placebo, Triamcinolone | -0.38 (-32.56, 15.35) |
| Aloe, Amlexanox, Glycyrrhiza, Placebo, Triamcinolone | 1.70 (-9.46, 41.74) |
| Aloe, Amlexanox, Laser, Placebo, Triamcinolone | -0.10 (-22.51, 22.79) |
| Aloe, Placebo, Triamcinolone | -0.13 (-27.69, 24.14) |
| Aloe, Placebo, Triamcinolone | -0.13 (-27.69, 24.14) |
| Aloe, Placebo, Triamcinolone | -0.13 (-27.69, 24.14) |
| Amlexanox, Laser, Triamcinolone | 0.01 (-25.66, 20.43) |

## Variance Calculation

| **Parameter** | **Median (95% CI)** |
|---|---|
| Random Effects Standard Deviation | 23.41 (17.97, 32.05) |
| Inconsistency Standard Deviation | 6.74 (0.15, 37.28) |

## Convergence Diagnostics

Convergence is assessed using the Brooks-Gelman-Rubin method. This method compares within-chain and between-chain variance to calculate the Potential Scale Reduction Factor (PSRF). A PSRF close to one indicates approximate convergence has been reached.

| Parameter | PSRF |
|---|---|
| d.Allicin.Placebo | 1.00 |
| d.Aloe.Triamcinolone | 1.00 |
| d.Amlexanox.Dexamethasone | 1.00 |
| d.Amlexanox.Glycyrrhiza | 1.01 |
| d.Benzydamine.Doxyeycline | 1.00 |
| d.Benzydamine.Quercetin | 1.00 |
| d.Chlorhexidine.Nacetyleysteine | 1.00 |
| d.Chlorhexidine.Sucralfate | 1.00 |
| d.Glycyrrhiza.Laser | 1.01 |
| d.Placebo.Aloe | 1.00 |
| d.Placebo.Berberinegelatin | 1.00 |
| d.Placebo.Chitosan | 1.00 |
| d.Placebo.Diosmectite | 1.00 |
| d.Placebo.Insulinliposomalgel | 1.00 |
| d.placebo.Probiotics | 1.00 |
| d.Placebo.Silvernitrate | 1.00 |
| d.Placebo.Zinc | 1.15 |
| d.Triamcinolone.Amlexanox | 1.01 |
| d.Triamcinolone.Benzydamine | 1.00 |
| d.Triamcinolone.Chlorhexidine | 1.00 |
| d.Triamcinolone.Curcumin | 1.00 |
| d.Triamcinolone.TriesterGlycerolOxide | 1.00 |
| w.Aloe.Placebo.Amlexanox.Triamcinolone | 1.01 |
| w.Aloe.Placebo.Dexamethasone.Amlexanox.Triamcinolone | 1.01 |
| w.Aloe.Placebo.Doxycycline.Triamcinolone | 1.01 |
| w.Aloe.Placebo.Glycyrrhiza.Amlexanox.Triamcinolone | 1.02 |
| w.Aloe.Placebo.Laser.Amlexanox.Triamcinolone | 1.03 |
| w.Aloe.Placebo.Triamcinolone | 1.01 |
| w.Aloe.Placebo.Triamcinolone | 1.01 |
| w.Aloe.Placebo.Triamcinolone | 1.01 |
| w.Amlexanox.Laser.Triamcinolone | 1.01 |
| sd.d | 1.00 |
| sd.w | 1.03 |

Number of chains : 4
Tuning iterations : 20,000
Simulation iterations : 50,000
Thinning interval : 10
Inference samples : 10,000
Variance scaling factor : 2.5

## 3. Node-splitting analysis

### Node-splitting analysis of inconsistency

Inconsistency between direct and indirect estimates was estimated in the Node-Splitting Model. When P values is above 0.05 in a comparison, there is evidence of statistical inconsistency.

| Name | Direct Effect | Indirect Effect | Overall | P-Value |
|---|---|---|---|---|
| Aloe, Triamcinolone | 10.75 (-40.60, 60.29) | 22.21 (-21.04, 64.30) | 16.02 (-18.38, 51.47) | 0.72 |
| Amlexanox, Dexamethasone | -0.12 (-48.39, 48.02) | -11.31 (-64.58, 41.28) | -5.15 (-39.53, 29.62) | 0.74 |
| Amlexanox, Glycyrrhiza | -16.91 (-67.12, 32.29) | -3.20 (-38.03, 31.48) | -2.07 (-31.34, 26.50) | 0.66 |
| Amlexanox, Laser | 8.18 (-43.87, 59.96) | 7.79 (-17.34, 33.33) | 8.95 (-14.74, 31.73) | 0.99 |
| Amlexanox, Placebo | -33.36 (-55.45, -12.01) | -7.72 (-39.82, 22.76) | -23.26 (-42.15, -4.15) | 0.17 |
| Amlexanox, Triamcinolone | 42.21 (-4.58, 87.65) | -7.76 (-33.96, 19.47) | 4.98 (-18.10, 29.39) | 0.07 |
| Benzydamine, Doxycycline | -0.13 (-49.50, 48.84) | -18.79 (-71.52, 36.16) | -7.07 (-48.88, 35.26) | 0.6 |
| Benzydamine, Placebo | -12.10 (-60.76, 37.64) | -20.69 (-67.17, 25.07) | -17.16 (-57.33, 24.03) | 0.8 |
| Benzydamine, Triamcinolone | -0.28 (-49.39, 48.65) | 24.33 (-23.53, 73.33) | 11.21 (-29.12, 52.26) | 0.46 |
| Dexamethasone, Placebo | -13.05 (-63.34, 36.72) | -24.48 (-77.06, 27.32) | -18.31 (-52.81, 16.30) | 0.75 |
| Doxycycline, Triamcinolone | 0.03 (-49.01, 48.53) | 23.59 (-8.72, 56.12) | 18.39 (-9.26, 46.67) | 0.4 |
| Glycyrrhiza, Laser | 24.22 (-25.07, 74.40) | 8.48 (-20.95, 38.30) | 11.08 (-16.23, 38.16) | 0.57 |
| Laser, Placebo | -34.75 (-53.20, -16.15) | -30.79 (-59.83, -1.68) | -32.21 (-48.08, -16.39) | 0.8 |
| Laser, Triamcinolone | -5.25 (-35.21, 24.96) | -7.16 (-36.01, 22.01) | -3.74 (-23.22, 16.50) | 0.92 |
| Placebo, Triamcinolone | 18.98 (-5.22, 43.91) | 44.45 (16.19, 73.39) | 28.45 (10.36, 46.76) | 0.18 |

## 4. Network structure

### Network structure of sensitivity analysis

46 RCTs involving 23 local interventions were included in the sensitivity analysis considering the symptom-reducing effect.

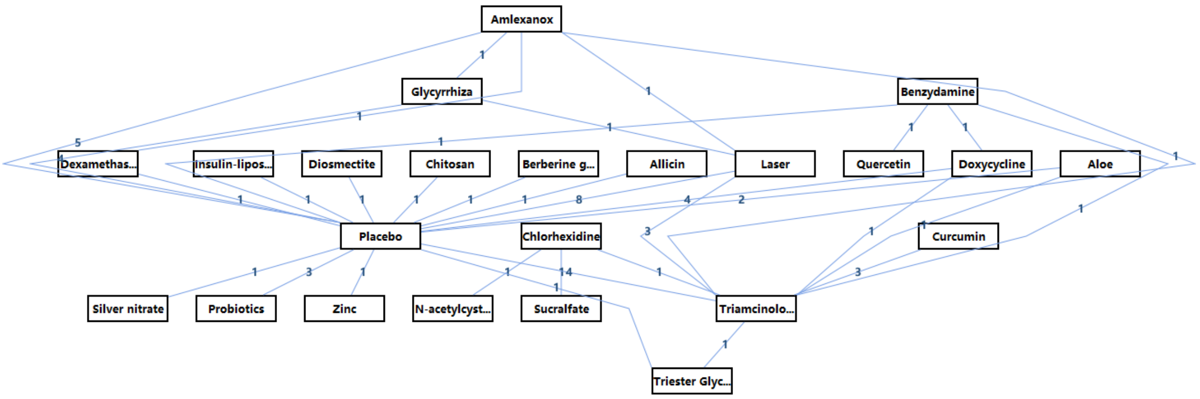

## 5. Pairwise meta-analysis

### Pairwise meta-analysis of symptom-reducing effect

Symptom-reducing effect were measured by mean difference (MD) and 95% confidence interval (CI).

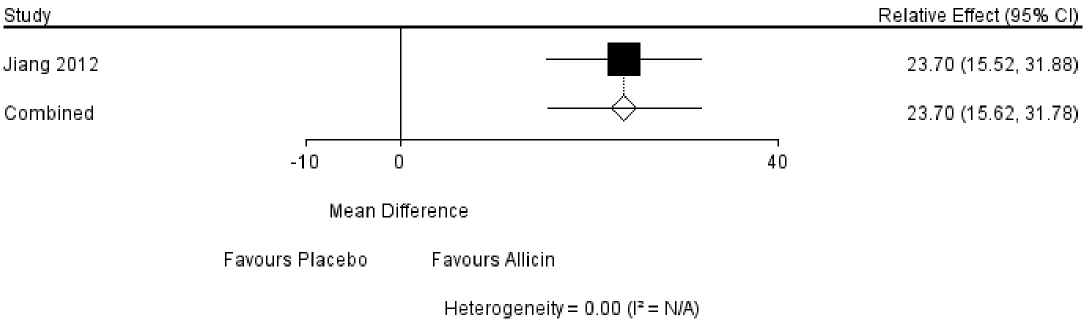


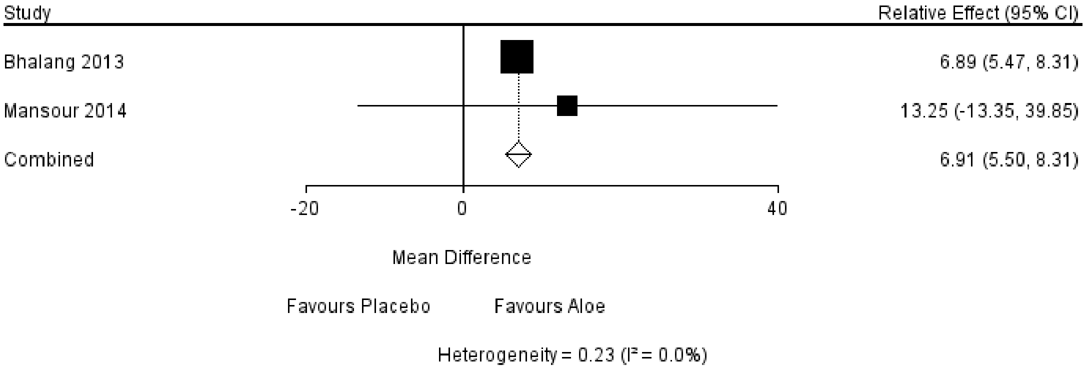


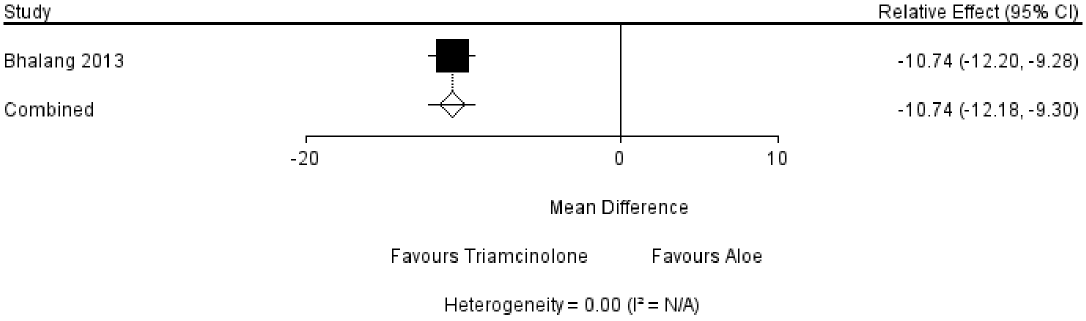

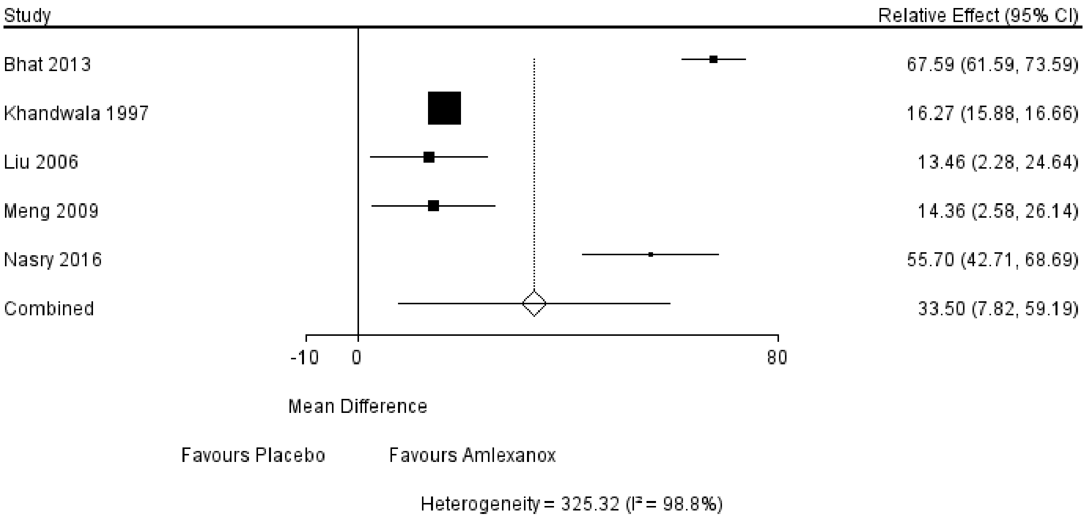
Study
Relative Effect (95% CI)
Bhat 2013
67.59 (61.59, 73.59)
Khandwala 1997
16.27 (15.88, 16.66)
Liu 2006
13.46 (2.28, 24.64)
Meng 2009
14.36 (2.58, 26.14)
Nasry 2016
55.70 (42.71, 68.69)
Combined
33.50 (7.82, 59.19)
-10
0
80
Mean Difference
Favours Placebo
Favours Amlexanox
Heterogeneity = 325.32 (I² = 98.8%)

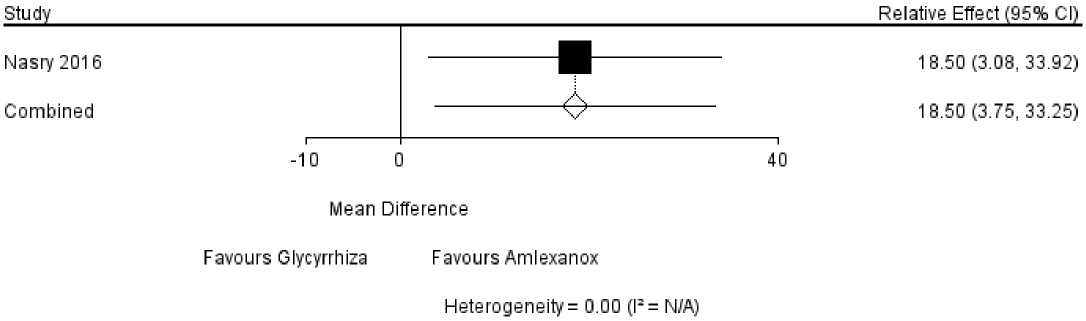
Study
Relative Effect (95% CI)
Nasry 2016
18.50 (3.08, 33.92)
Combined
18.50 (3.75, 33.25)
-10
0
40
Mean Difference
Favours Glycyrrhiza
Favours Amlexanox
Heterogeneity = 0.00 (I² = N/A)

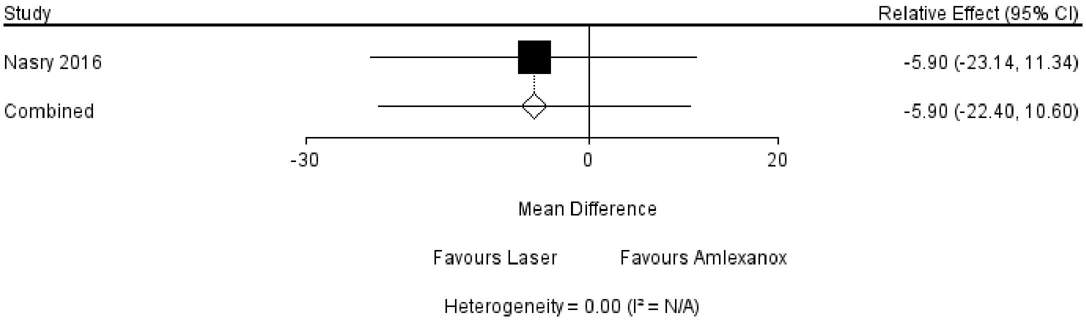
Study
Relative Effect (95% CI)
Nasry 2016
-5.90 (-23.14, 11.34)
Combined
-5.90 (-22.40, 10.60)
-30
0
20
Mean Difference
Favours Laser
Favours Amlexanox
Heterogeneity = 0.00 (I² = N/A)

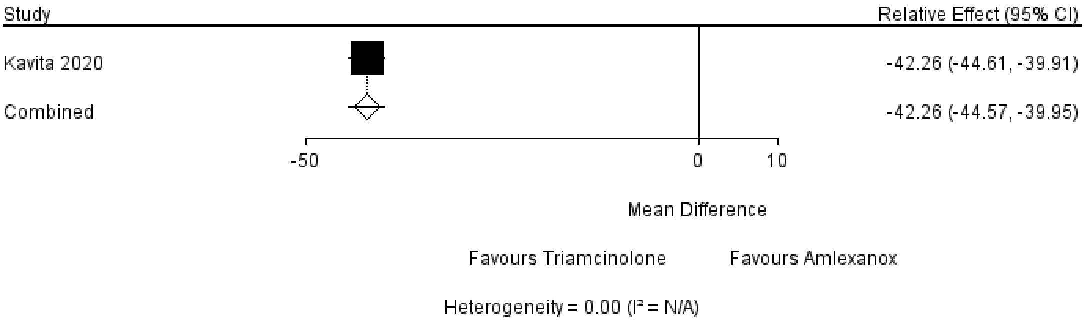
Study
Relative Effect (95% CI)
Kavita 2020
-42.26 (-44.61, -39.91)
Combined
-42.26 (-44.57, -39.95)
-50
0
10
Mean Difference
Favours Triamcinolone
Favours Amlexanox
Heterogeneity = 0.00 (I² = N/A)

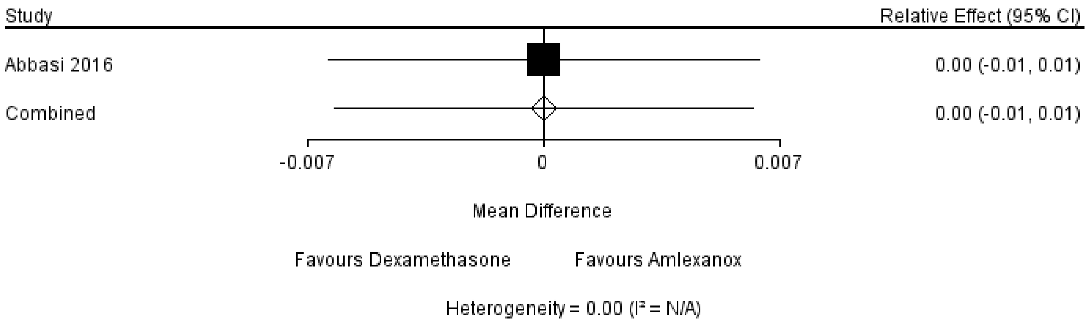
Study
Relative Effect (95% CI)
Abbasi 2016
0.00 (-0.01, 0.01)
Combined
0.00 (-0.01, 0.01)
-0.007
0
0.007
Mean Difference
Favours Dexamethasone
Favours Amlexanox
Heterogeneity = 0.00 (I² = N/A)

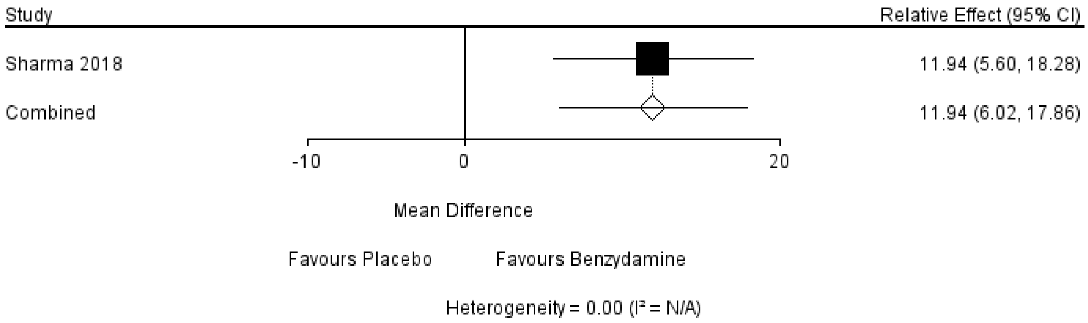
Study
Relative Effect (95% CI)
Sharma 2018
11.94 (5.60, 18.28)
Combined
11.94 (6.02, 17.86)
-10
0
20
Mean Difference
Favours Placebo
Favours Benzydamine
Heterogeneity = 0.00 (I² = N/A)

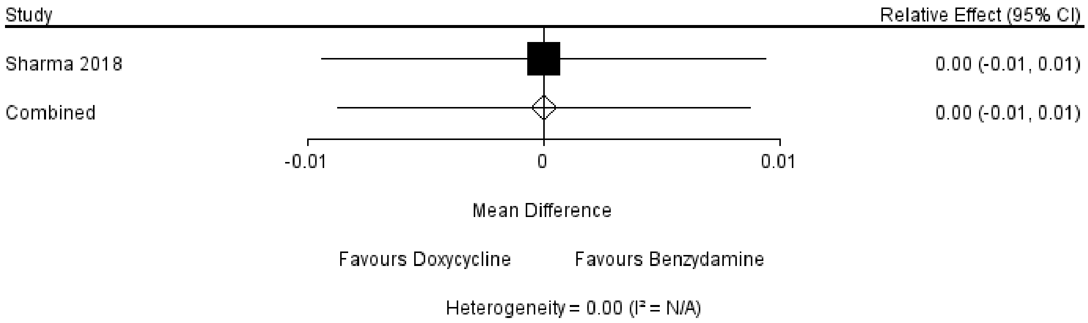
Study
Relative Effect (95% CI)
Sharma 2018
0.00 (-0.01, 0.01)
Combined
0.00 (-0.01, 0.01)
-0.01
0
0.01
Mean Difference
Favours Doxycycline
Favours Benzydamine
Heterogeneity = 0.00 (I² = N/A)

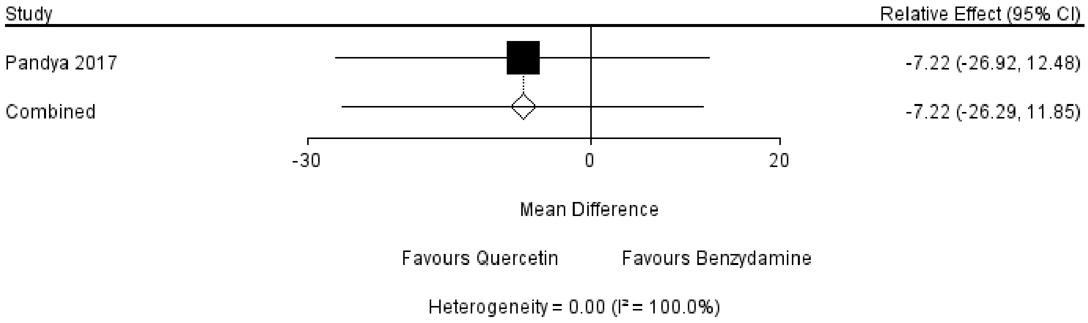
Study
Relative Effect (95% CI)
Pandya 2017
-7.22 (-26.92, 12.48)
Combined
-7.22 (-26.29, 11.85)
-30
0
20
Mean Difference
Favours Quercetin
Favours Benzydamine
Heterogeneity = 0.00 (I² = 100.0%)

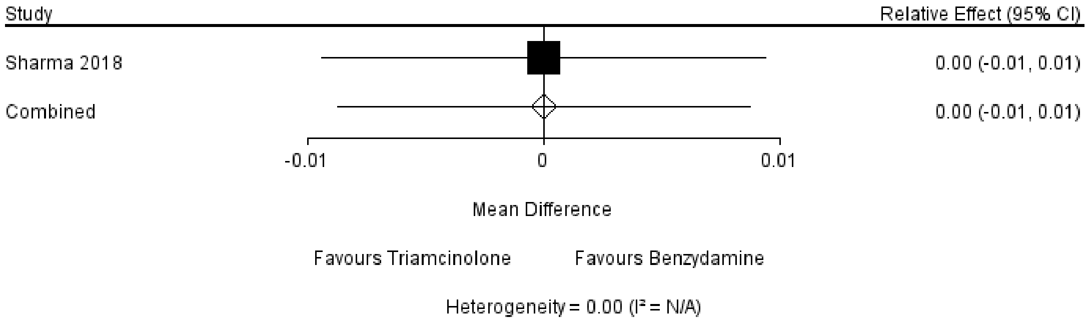
Study
Relative Effect (95% CI)
Sharma 2018
0.00 (-0.01, 0.01)
Combined
0.00 (-0.01, 0.01)
-0.01
0
0.01
Mean Difference
Favours Triamcinolone
Favours Benzydamine
Heterogeneity = 0.00 (I² = N/A)

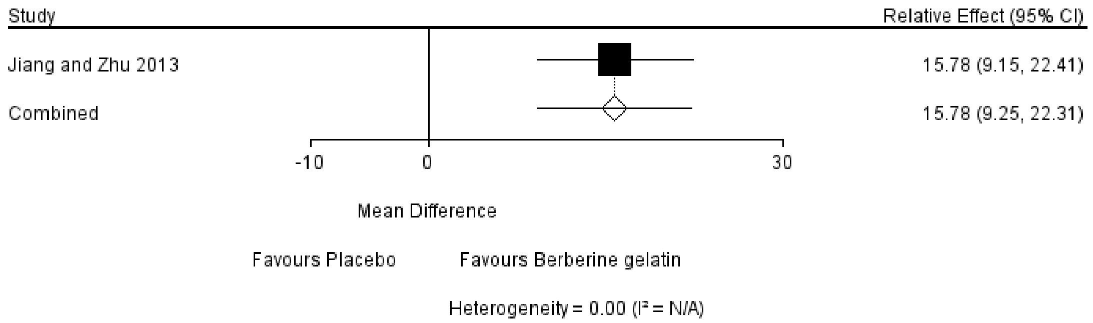
Study
Relative Effect (95% CI)
Jiang and Zhu 2013
15.78 (9.15, 22.41)
Combined
15.78 (9.25, 22.31)
-10
0
30
Mean Difference
Favours Placebo
Favours Berberine gelatin
Heterogeneity = 0.00 (I² = N/A)

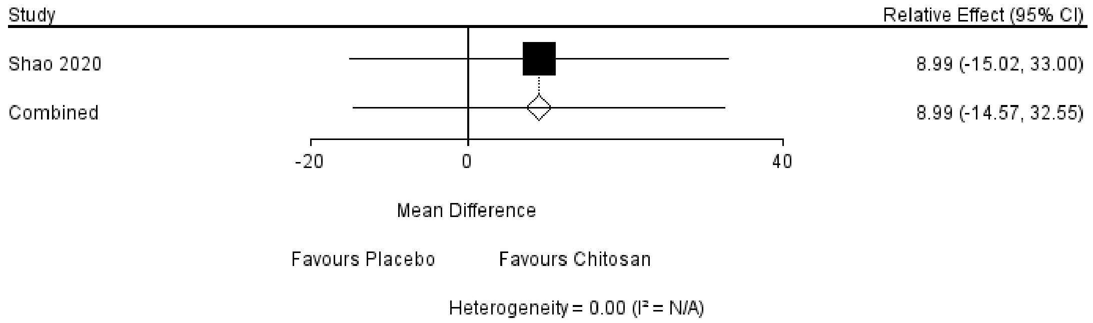
Study
Relative Effect (95% CI)
Shao 2020
8.99 (-15.02, 33.00)
Combined
8.99 (-14.57, 32.55)
-20
0
40
Mean Difference
Favours Placebo
Favours Chitosan
Heterogeneity = 0.00 (I² = N/A)

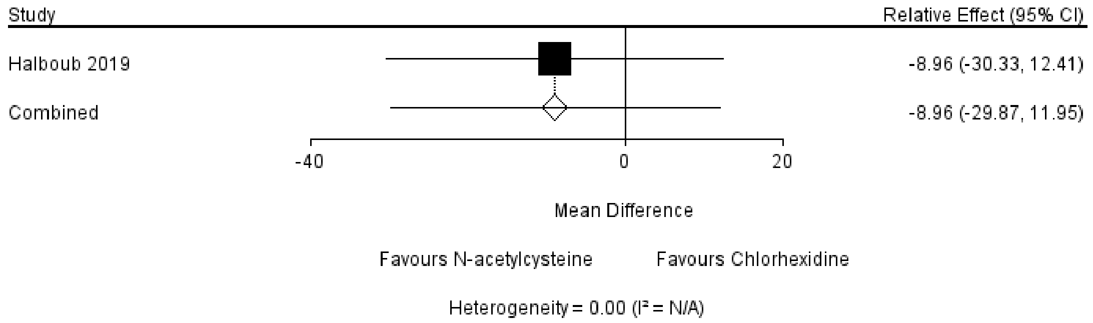
Study
Relative Effect (95% CI)
Halboub 2019
-8.96 (-30.33, 12.41)
Combined
-8.96 (-29.87, 11.95)
-40
0
20
Mean Difference
Favours N-acetylcysteine
Favours Chlorhexidine
Heterogeneity = 0.00 (I² = N/A)

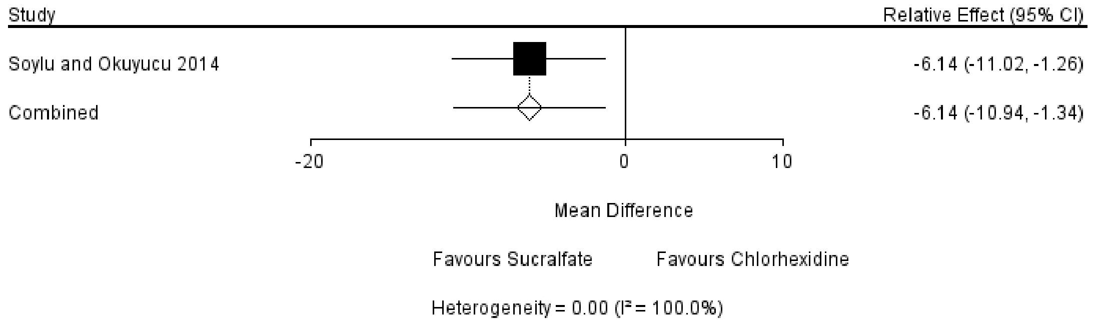
Study
Relative Effect (95% CI)
Soylu and Okuyucu 2014
-6.14 (-11.02, -1.26)
Combined
-6.14 (-10.94, -1.34)
-20
0
10
Mean Difference
Favours Sucralfate
Favours Chlorhexidine
Heterogeneity = 0.00 (I² = 100.0%)

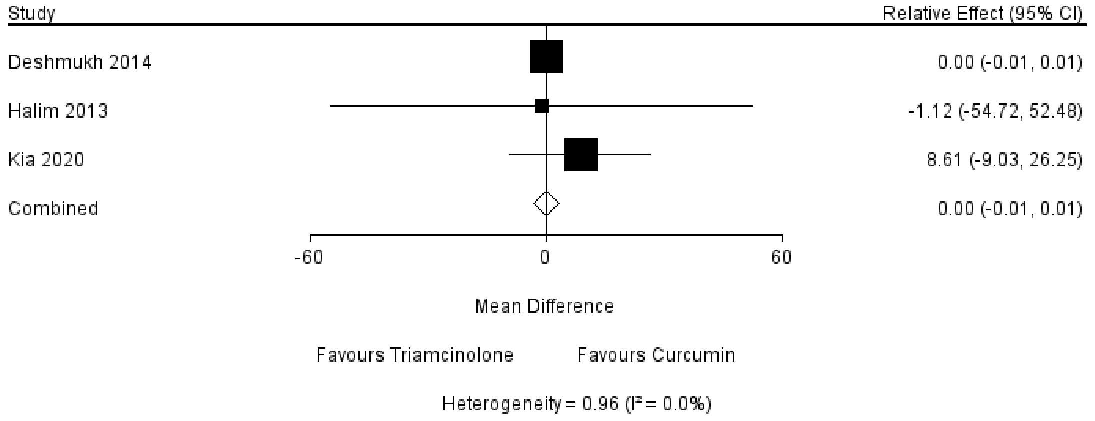
Study
Relative Effect (95% CI)
Deshmukh 2014
0.00 (-0.01, 0.01)
Halim 2013
-1.12 (-54.72, 52.48)
Kia 2020
8.61 (-9.03, 26.25)
Combined
0.00 (-0.01, 0.01)
-60
0
60
Mean Difference
Favours Triamcinolone
Favours Curcumin
Heterogeneity = 0.96 (I² = 0.0%)

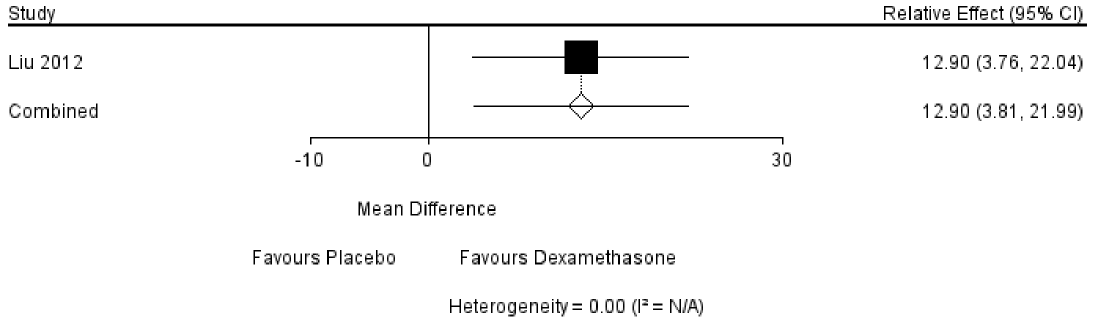
Study
Relative Effect (95% CI)
Liu 2012
12.90 (3.76, 22.04)
Combined
12.90 (3.81, 21.99)
-10
0
30
Mean Difference
Favours Placebo
Favours Dexamethasone
Heterogeneity = 0.00 (I² = N/A)

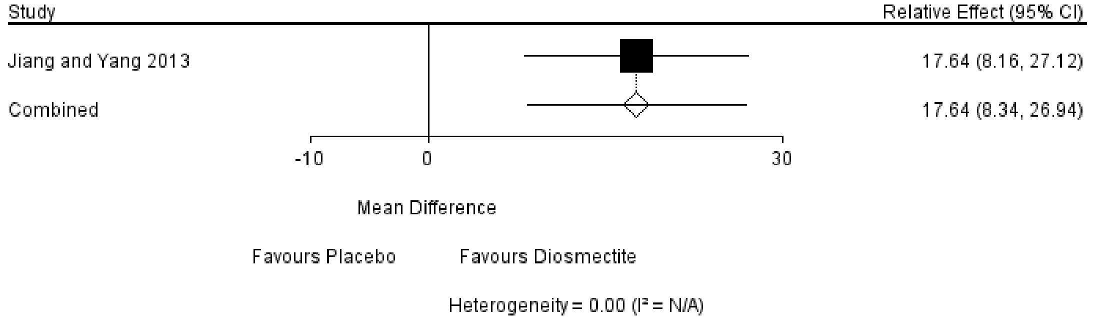
Study
Relative Effect (95% CI)
Jiang and Yang 2013
17.64 (8.16, 27.12)
Combined
17.64 (8.34, 26.94)
-10
0
30
Mean Difference
Favours Placebo
Favours Diosmectite
Heterogeneity = 0.00 (I² = N/A)

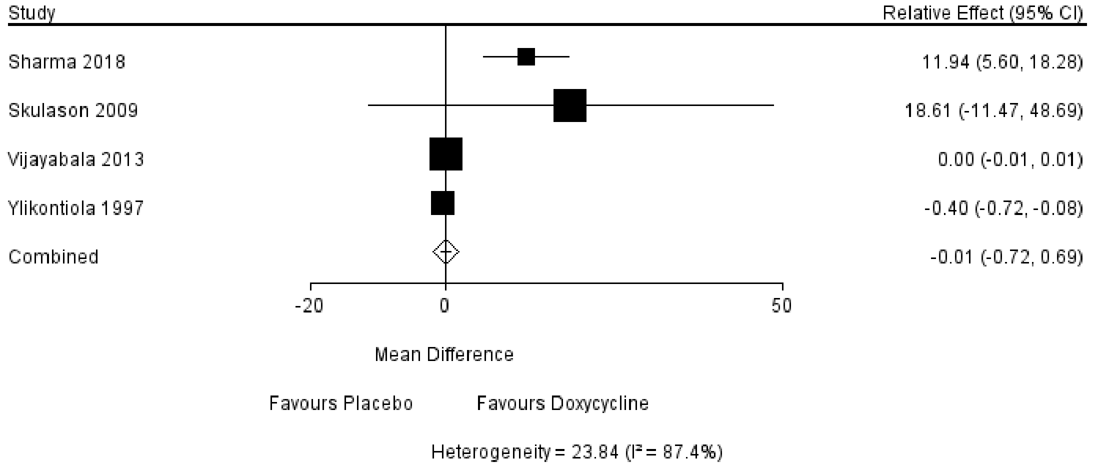
Study
Relative Effect (95% CI)
Sharma 2018
11.94 (5.60, 18.28)
Skulason 2009
18.61 (-11.47, 48.69)
Vijayabala 2013
0.00 (-0.01, 0.01)
Ylikontiola 1997
-0.40 (-0.72, -0.08)
Combined
-0.01 (-0.72, 0.69)
-20
0
50
Mean Difference
Favours Placebo
Favours Doxycycline
Heterogeneity = 23.84 (I² = 87.4%)

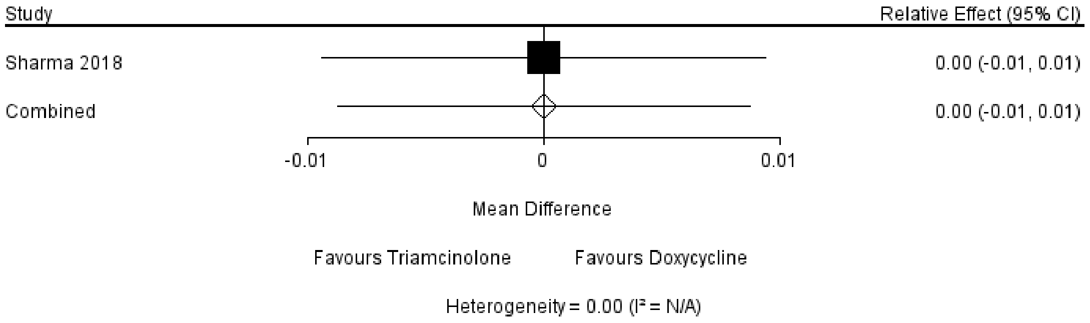

Study
Relative Effect (95% CI)
Sharma 2018
0.00 (-0.01, 0.01)
Combined
0.00 (-0.01, 0.01)
-0.01
0
0.01
Mean Difference
Favours Triamcinolone
Favours Doxycycline
Heterogeneity = 0.00 (I² = N/A)


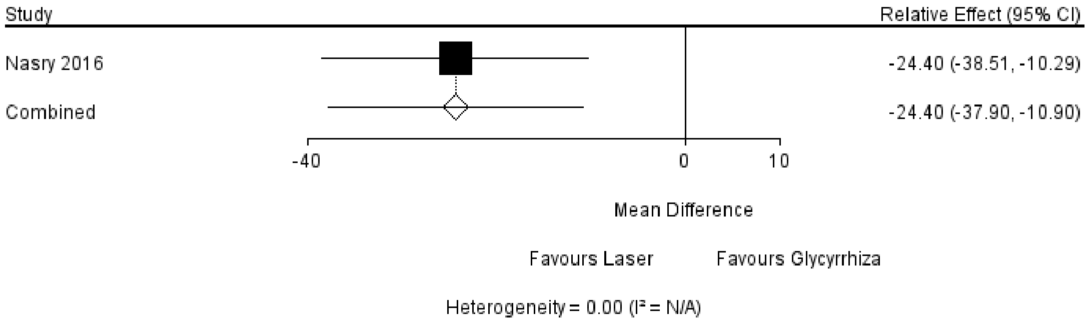

Study
Relative Effect (95% CI)
Nasry 2016
-24.40 (-38.51, -10.29)
Combined
-24.40 (-37.90, -10.90)
-40
0
10
Mean Difference
Favours Laser
Favours Glycyrrhiza
Heterogeneity = 0.00 (I² = N/A)


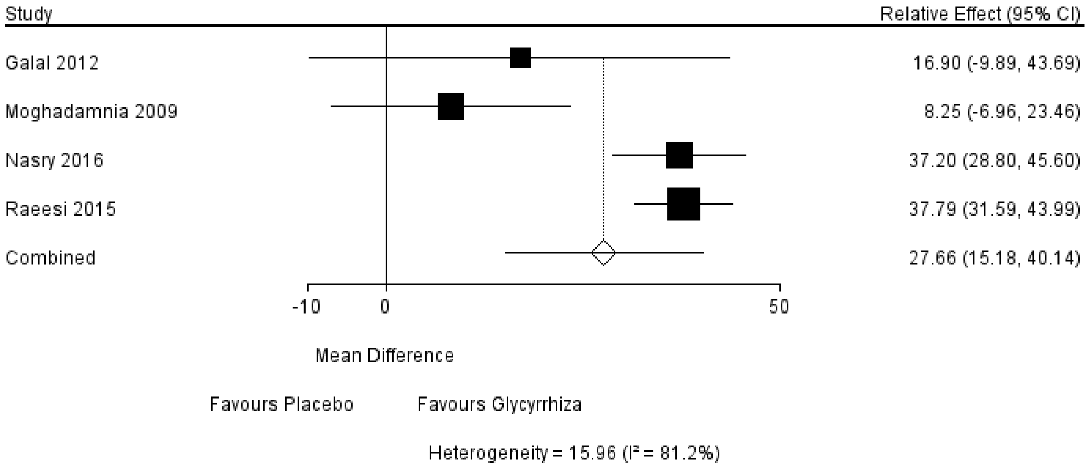

Study
Relative Effect (95% CI)
Galal 2012
16.90 (-9.89, 43.69)
Moghadamnia 2009
8.25 (-6.96, 23.46)
Nasry 2016
37.20 (28.80, 45.60)
Raeesi 2015
37.79 (31.59, 43.99)
Combined
27.66 (15.18, 40.14)
-10
0
50
Mean Difference
Favours Placebo
Favours Glycyrrhiza
Heterogeneity = 15.96 (I² = 81.2%)


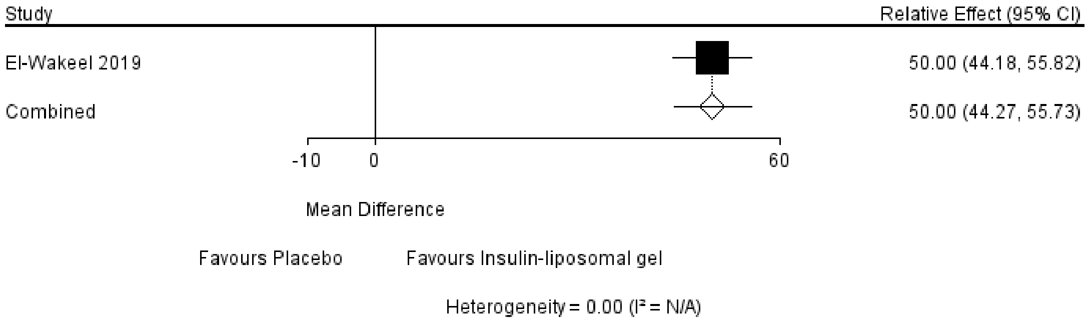

Study
Relative Effect (95% CI)
El-Wakeel 2019
50.00 (44.18, 55.82)
Combined
50.00 (44.27, 55.73)
-10
0
60
Mean Difference
Favours Placebo
Favours Insulin-liposomal gel
Heterogeneity = 0.00 (I² = N/A)

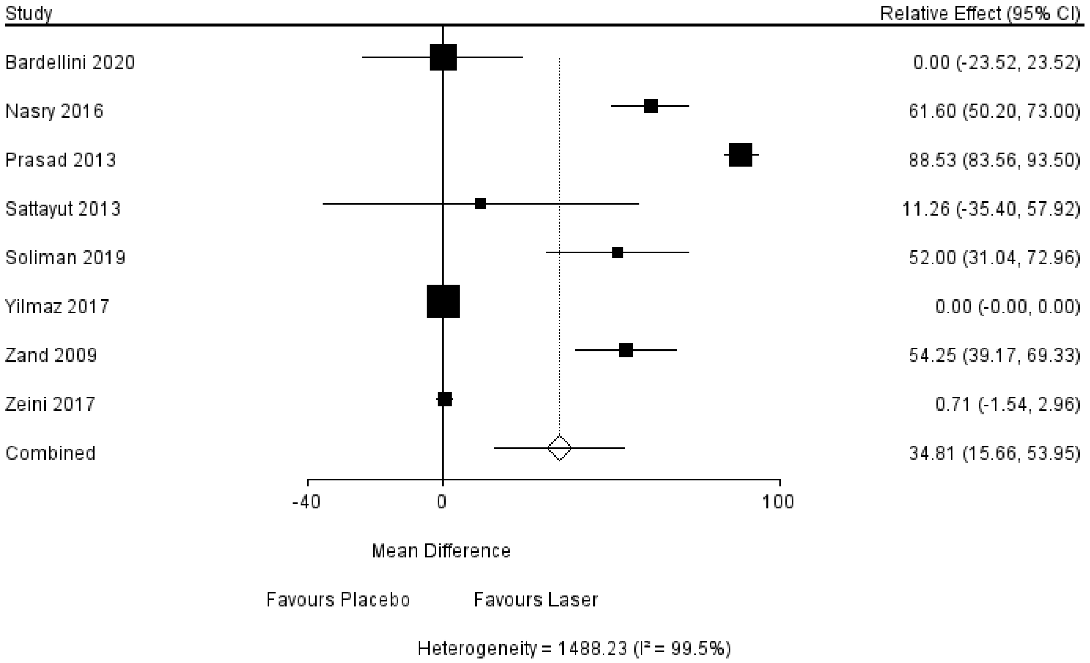
Study
Relative Effect (95% CI)
Bardellini 2020
0.00 (-23.52, 23.52)
Nasry 2016
61.60 (50.20, 73.00)
Prasad 2013
88.53 (83.56, 93.50)
Sattayut 2013
11.26 (-35.40, 57.92)
Soliman 2019
52.00 (31.04, 72.96)
Yilmaz 2017
0.00 (-0.00, 0.00)
Zand 2009
54.25 (39.17, 69.33)
Zeini 2017
0.71 (-1.54, 2.96)
Combined
34.81 (15.66, 53.95)
-40
0
100
Mean Difference
Favours Placebo
Favours Laser
Heterogeneity = 1488.23 (I² = 99.5%)

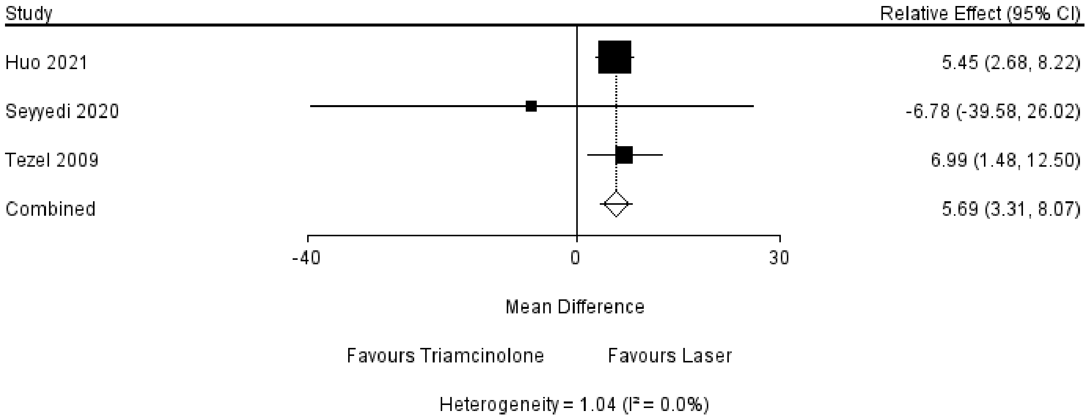
Study
Relative Effect (95% CI)
Huo 2021
5.45 (2.68, 8.22)
Seyyedi 2020
-6.78 (-39.58, 26.02)
Tezel 2009
6.99 (1.48, 12.50)
Combined
5.69 (3.31, 8.07)
-40
0
30
Mean Difference
Favours Triamcinolone
Favours Laser
Heterogeneity = 1.04 (I² = 0.0%)

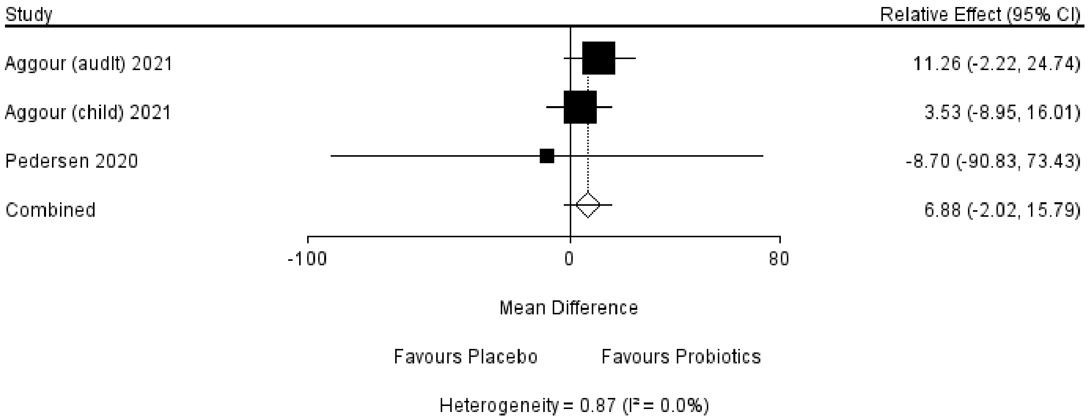
Study
Relative Effect (95% CI)
Aggour (audlt) 2021
11.26 (-2.22, 24.74)
Aggour (child) 2021
3.53 (-8.95, 16.01)
Pedersen 2020
-8.70 (-90.83, 73.43)
Combined
6.88 (-2.02, 15.79)
-100
0
80
Mean Difference
Favours Placebo
Favours Probiotics
Heterogeneity = 0.87 (I² = 0.0%)

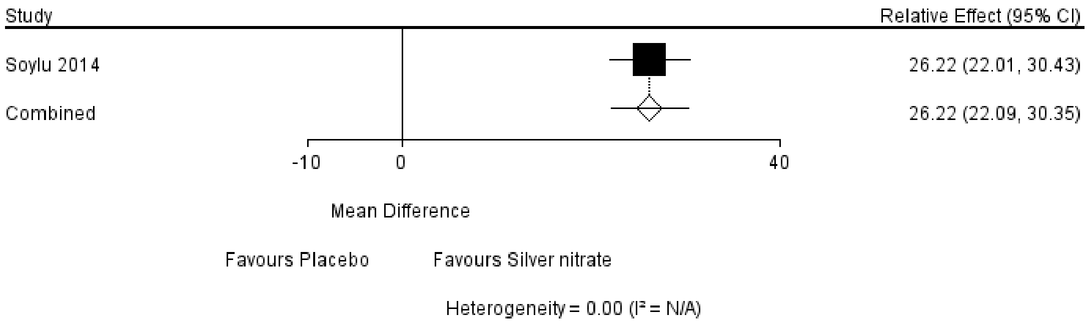
Study
Relative Effect (95% CI)
Soylu 2014
26.22 (22.01, 30.43)
Combined
26.22 (22.09, 30.35)
-10
0
40
Mean Difference
Favours Placebo
Favours Silver nitrate
Heterogeneity = 0.00 (I² = N/A)

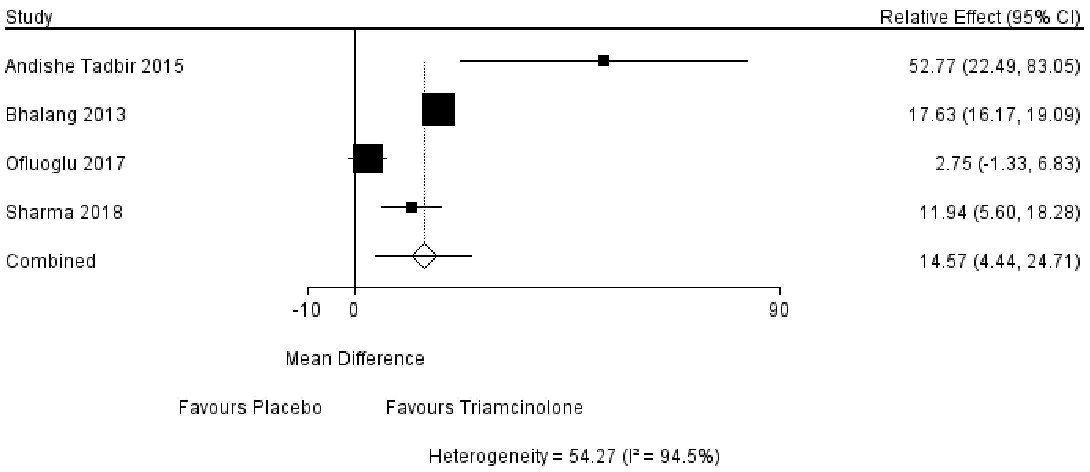
Study
Relative Effect (95% CI)
Andishe Tadbir 2015
52.77 (22.49, 83.05)
Bhalang 2013
17.63 (16.17, 19.09)
Ofluoglu 2017
2.75 (-1.33, 6.83)
Sharma 2018
11.94 (5.60, 18.28)
Combined
14.57 (4.44, 24.71)
-10
0
90
Mean Difference
Favours Placebo
Favours Triamcinolone
Heterogeneity = 54.27 (I² = 94.5%)

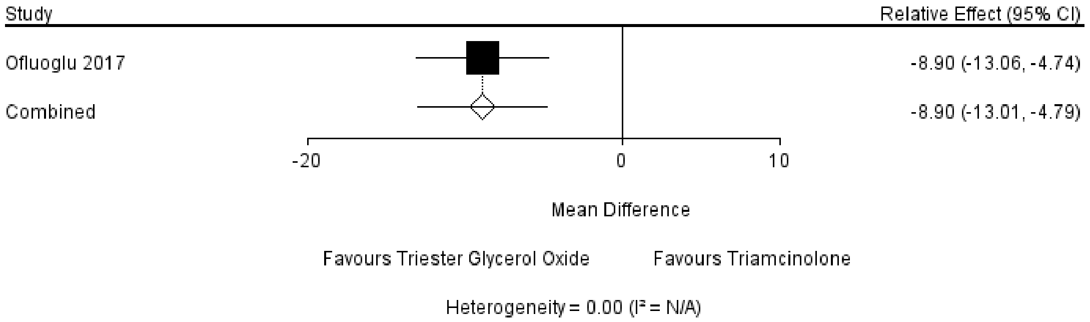
Study
Relative Effect (95% CI)
Ofluoglu 2017
-8.90 (-13.06, -4.74)
Combined
-8.90 (-13.01, -4.79)
-20
0
10
Mean Difference
Favours Triester Glycerol Oxide
Favours Triamcinolone
Heterogeneity = 0.00 (I² = N/A)

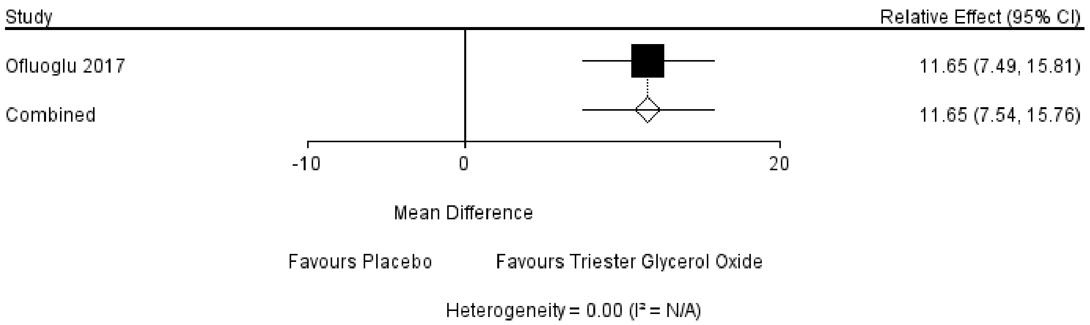
Study
Relative Effect (95% CI)
Ofluoglu 2017
11.65 (7.49, 15.81)
Combined
11.65 (7.54, 15.76)
-10
0
20
Mean Difference
Favours Placebo
Favours Triester Glycerol Oxide
Heterogeneity = 0.00 (I² = N/A)

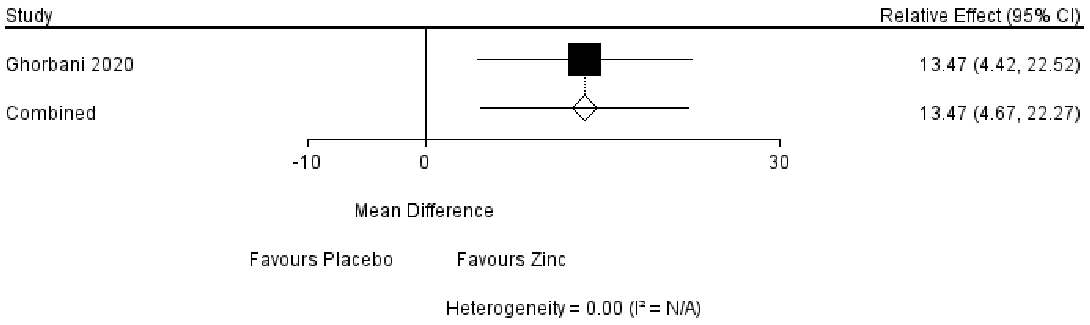
Study
Relative Effect (95% CI)
Ghorbani 2020
13.47 (4.42, 22.52)
Combined
13.47 (4.67, 22.27)
-10
0
30
Mean Difference
Favours Placebo
Favours Zinc
Heterogeneity = 0.00 (I² = N/A)

| Comparison | NO. of included study | Heterogeneity ($I^2$) | Combined effect [SMD (95%CI)] |
|---|---|---|---|
| Allicin vs | | | |
| Placebo | 1 | NA | 23.70 (15.62, 31.78) |
| Aloe vs | | | |
| Placebo | 2 | 0% | 6.91 (5.50, 8.31) |
| Triamcinolone | 1 | NA | -10.74 (-12.18, -9.30) |
| Amlexanox vs | | | |
| Placebo | 5 | 99% | 33.50 (7.82, 59.19) |
| Glycyrrhiza | 1 | NA | 18.50 (3.75, 33.25) |
| Laser | 1 | NA | -5.90 (-22.40, 10.60) |
| Triamcinolone | 1 | NA | -42.26 (-44.57, -39.95) |
| Dexamethasone | 1 | NA | 0.00 (-0.01, 0.01) |
| Benzydamine vs | | | |
| Placebo | 1 | NA | 11.94 (6.02, 17.86) |
| Doxycycline | 1 | NA | 0.00 (-0.01, 0.01) |
| Quercetin | 1 | NA | -7.22 (-26.29, 11.85) |
| Triamcinolone | 1 | NA | 0.00 (-0.01, 0.01) |
| Berberine gelatin vs | | | |
| Placebo | 1 | NA | 15.78 (9.25, 22.31) |
| Chitosan vs | | | |
| Placebo | 1 | NA | 8.99 (-14.57, 32.55) |
| Chlorhexidine vs | | | |
| N-acetylcysteine | 1 | NA | -8.96 (-29.87, 11.95) |
| Sucralfate | 1 | NA | -6.14 (-10.94, -1.34) |
| Curcumin vs | | | |
| Triamcinolone | 3 | 0% | 0.00 (-0.01, 0.01) |
| Dexamethasone vs | | | |
| Placebo | 1 | NA | 12.90 (3.81, 21.99) |
| Diosmectite vs | | | |
| Placebo | 1 | NA | 17.64 (8.34, 26.94) |
| Doxycycline vs | | | |
| Placebo | 4 | 87.4% | -0.01 (-0.72, 0.69) |
| Triamcinolone | 1 | NA | 0.00 (-0.01, 0.01) |
| Glycyrrhiza vs | | | |
| Laser | 1 | NA | -24.40 (-37.90, -10.90) |
| Placebo | 4 | 81.2% | 27.66 (15.18, 40.14) |
| Insulin-liposomal gel vs | | | |
| Placebo | 1 | NA | 50.00 (44.27, 55.73) |
| Laser vs | | | |
| Placebo | 8 | 99.5% | 34.81 (15.66, 53.95) |
| Triamcinolone | 3 | 0% | 6.99 (1.85, 12.13) |
| Probiotics vs | | | |
| Placebo | 3 | 0% | 6.88 (-2.02, 15.79) |
| Silver nitrate vs | | | |
| Placebo | 1 | NA | 26.22 (26.09, 30.35) |
| Triamcinolone vs | | | |
| Placebo | 4 | 94.5% | 14.57 (4.44, 24.71) |
| Triester Glycerol Oxide | 1 | NA | -8.90 (-13.01, -4.79) |
| Triester Glycerol Oxide vs | | | |
| Placebo | 1 | NA | 11.65 (7.54, 15.76) |
| Zinc vs | | | |
| Placebo | 1 | NA | 13.47 (4.67, 22.27) |

MD, mean difference; CI, confidence interval; NA, not applicable.

**6. Subgroup Discussion**

The 56 RCTs studied for the symptom-reducing effect had different durations of local intervention and were examined at different times during the trial. The size-reducing effect is discussed separately according to the different examination times.

**Day 0**

**1. Consistency Model**

1.1 Summary estimates

| | | | |
|---|---|---|---|
| Aloe | 60.01 (-45.60, 159.40) | -10.79 (-107.87, 83.10) | 5.38 (-94.80, 102.16) |
| -60.01 (-159.40, 45.60) | Laser | -70.96 (-115.42, -26.81) | -54.41 (-133.09, 20.80) |
| 10.79 (-83.10, 107.87) | 70.96 (26.81, 115.42) | Placebo | 16.74 (-60.78, 92.32) |
| -5.38 (-102.16, 94.80) | 54.41 (-20.80, 133.09) | -16.74 (-92.32, 60.78) | Triamcinolone |

1.2 Rank probability(Rank 1 is best, rank N is worst)

| Drug | Rank 1 | Rank 2 | Rank 3 | Rank 4 |
|---|---|---|---|---|
| Aloe | 0.1 | 0.3 | 0.28 | 0.33 |
| Laser | 0.85 | 0.12 | 0.03 | 0 |
| Placebo | 0 | 0.18 | 0.35 | 0.47 |
| Triamcinolone | 0.05 | 0.41 | 0.35 | 0.2 |

1.3 Consistency check

| Parameter | Median (95% CI) |
|---|---|
| Random Effects Standard Deviation | 44.73 (25.24, 86.33) |

1.4 Convergence Diagnostics

| Parameter | PSRF |
|---|---|
| d.Placebo.Aloe | 1.00 |
| d.Placebo.Laser | 1.00 |
| d.Placebo.Triamcinolone | 1.00 |
| sd.d | 1.00 |

Number of chains : 4
Tuning iterations : 20,000
Simulation iterations : 50,000
Thinning interval : 10
Inference samples : 10,000
Variance scaling factor: 2.5

**2.Inconsistency Model**

2.1 Summary estimates

| | | | |
|---|---|---|---|
| Aloe | 63.25 (-46.01, 172.29) | -8.69 (-111.98, 93.09) | 2.84 (-99.39, 109.45) |
| -63.25 (-172.29, 46.01) | Laser | -71.93 (-117.97, -24.56) | -51.07 (-139.54, 34.18) |
| 8.69 (-93.09, 111.98) | 71.93 (24.56, 117.97) | Placebo | 27.06 (-112.79, 179.88) |
| -2.84 (-109.45, 99.39) | 51.07 (-34.18, 139.54) | -27.06 (-179.88, 112.79) | Triamcinolone |

2.2 Inconsistency Factors

| Cycle | Median (95% CI) |
|---|---|
| Laser, Placebo, Triamcinolone | 4.62 (-68.95, 94.10) |

2.3 Variance Calculation

| Parameter | Median (95% CI) |
|---|---|
| Random Effects Standard Deviation | 46.49 (25.57, 88.78) |
| Inconsistency Standard Deviation | 41.76 (1.80, 92.60) |

## 2.4 Convergence Diagnostics

| Parameter | PSRF |
|---|---|
| d.Aloe.Placebo | 1.00 |
| d.Laser.Triamcinolone | 1.00 |
| d.Placebo.Laser | 1.00 |
| w.Laser.Placebo.Triamcinolone | 1.00 |
| sd.d | 1.00 |
| sd.w | 1.00 |

Number of chains : 4
Tuning iterations : 20,000
Simulation iterations : 50,000
Thinning interval : 10
Inference samples : 10,000
Variance scaling factor: 2.5

## 3. Node-splitting analysis

| Name | Direct Effect | Indirect Effect | Overall | P-Value |
|---|---|---|---|---|
| Laser, Placebo | -73.56 (-125.70, -22.42) | -43.34 (-201.05, 115.96) | -70.96 (-115.42, -26.81) | 0.67 |
| Laser, Triamcinolone | -40.77 (-154.72, 70.35) | -72.62 (-197.56, 52.79) | -54.41 (-133.09, 20.80) | 0.66 |
| Placebo, Triamcinolone | 2.43 (-112.79, 118.03) | 34.31 (-92.49, 158.95) | 16.74 (-60.78, 92.32) | 0.66 |

## 4. Network structure

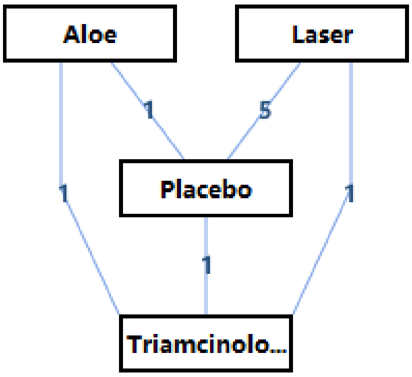


**Day 1**

## 1. Consistency Model

### 1.1 Summary estimates

| | | | | | | | |
|---|---|---|---|---|---|---|---|
| Curcumin | -0.37 (-96.16, 97.91) | -23.52 (-121.32, 77.32) | 35.77 (-75.63, 148.25) | 44.11 (-24.98, 111.65) | -26.08 (-106.28, 55.32) | -10.56 (-120.97, 103.79) | -1.24 (-56.79, 55.56) |
| 0.37 (-97.91, 96.16) | Doxycycline | -22.54 (-101.25, 51.93) | 36.79 (-58.43, 131.02) | 44.49 (-24.94, 108.29) | -25.74 (-80.55, 29.01) | -9.82 (-102.66, 83.38) | -0.72 (-83.26, 77.19) |
| 23.52 (-77.32, 121.32) | 22.54 (-51.93, 101.25) | Glycyrrhiza | 59.22 (-34.92, 152.48) | 67.71 (-1.53, 133.22) | -3.13 (-57.59, 52.06) | 13.12 (-80.52, 104.59) | 22.47 (-60.22, 99.57) |
| -35.77 (-148.25, 75.63) | -36.79 (-131.02, 58.43) | -59.22 (-152.48, 34.92) | Insulin-liposomal gel | 8.30 (-79.64, 92.53) | -62.07 (-137.87, 14.99) | -46.23 (-152.78, 61.73) | -36.81 (-135.30, 56.99) |
| -44.11 (-111.65, 24.98) | -44.49 (-108.29, 24.94) | -67.71 (-133.22, 1.53) | -8.30 (-92.53, 79.64) | Laser | -70.45 (-107.84, -28.77) | -54.50 (-137.91, 35.31) | -45.11 (-88.94, -1.39) |
| 26.08 (-55.32, 106.28) | 25.74 (-29.01, 80.55) | 3.13 (-52.06, 57.59) | 62.07 (-14.99, 137.87) | 70.45 (28.77, 107.84) | Placebo | 15.94 (-59.36, 92.86) | 25.28 (-35.00, 83.01) |
| 10.56 (-103.79, 120.97) | 9.82 (-83.38, 102.66) | -13.12 (-104.59, 80.52) | 46.23 (-61.73, 152.78) | 54.50 (-35.31, 137.91) | -15.94 (-92.86, 59.36) | Silver nitrate | 9.15 (-89.92, 103.20) |
| 1.24 (-55.56, 56.79) | 0.72 (-77.19, 83.26) | -22.47 (-99.57, 60.22) | 36.81 (-56.99, 135.30) | 45.11 (1.39, 88.94) | -25.28 (-83.01, 35.00) | -9.15 (-103.20, 89.92) | Triamcinolone |

### 1.2 Rank probability(Rank 1 is best, rank N is worst)

| Drug | Rank 1 | Rank 2 | Rank 3 | Rank 4 | Rank 5 | Rank 6 | Rank 7 | Rank 8 |
|---|---|---|---|---|---|---|---|---|
| Curcumin | 0.05 | 0.11 | 0.18 | 0.17 | 0.15 | 0.11 | 0.1 | 0.13 |
| Doxycycline | 0.04 | 0.09 | 0.19 | 0.18 | 0.2 | 0.15 | 0.09 | 0.06 |
| Glycyrrhiza | 0.01 | 0.03 | 0.06 | 0.1 | 0.13 | 0.17 | 0.2 | 0.3 |
| Insulin-liposomal gel | 0.37 | 0.27 | 0.13 | 0.09 | 0.06 | 0.04 | 0.02 | 0.02 |
| Laser | 0.47 | 0.37 | 0.11 | 0.03 | 0.01 | 0 | 0 | 0 |
| Placebo | 0 | 0 | 0.01 | 0.03 | 0.11 | 0.25 | 0.37 | 0.23 |
| Silver nitrate | 0.05 | 0.07 | 0.13 | 0.13 | 0.14 | 0.14 | 0.12 | 0.21 |
| Triamcinolone | 0.01 | 0.06 | 0.19 | 0.26 | 0.2 | 0.14 | 0.11 | 0.05 |

1.3 Consistency check

| Parameter | Median (95% CI) |
|---|---|
| Random Effects Standard Deviation | 33.57 (18.72, 66.78) |

1.4 Convergence Diagnostics

| Parameter | PSRF |
|---|---|
| d.Laser.Placebo | 1.00 |
| d.Laser.Triamcinolone | 1.00 |
| d.Placebo.Doxycycline | 1.00 |
| d.Placebo.Glycyrrhiza | 1.00 |
| d.Placebo.Insulinliposomalgel | 1.00 |
| d.Placebo.Silvernitrate | 1.00 |
| d.Triamcinolone.Curcumin | 1.00 |
| sd.d | 1.00 |

Number of chains : 4
Tuning iterations : 20,000
Simulation iterations : 50,000
Thinning interval : 10
Inference samples : 10,000
Variance scaling factor: 2.5

**2.Inconsistency Model**

2.1 Summary estimates

| | | | | | | | |
|---|---|---|---|---|---|---|---|
| Curcumin | -1.01 (-97.93, 98.18) | -23.17 (-119.67, 73.70) | 33.83 (-76.96, 146.31) | 43.94 (-27.10, 115.41) | -26.28 (-106.73, 57.30) | -10.71 (-122.07, 100.07) | -0.75 (-54.60, 55.29) |
| 1.01 (-98.18, 97.93) | Doxycycline | -21.97 (-99.46, 53.67) | 34.47 (-58.00, 130.14) | 45.24 (-22.76, 111.78) | -25.57 (-79.97, 27.68) | -9.36 (-101.74, 82.54) | 0.45 (-80.83, 80.36) |
| 23.17 (-73.70, 119.67) | 21.97 (-53.67, 99.46) | Glycyrrhiza | 57.99 (-34.90, 152.79) | 67.35 (-0.81, 131.27) | -3.43 (-55.87, 50.90) | 12.43 (-82.33, 108.22) | 22.44 (-56.96, 101.81) |
| -33.83 (-146.31, 76.96) | -34.47 (-130.14, 58.00) | -57.99 (-152.79, 34.90) | Insulin-liposomal gel | 10.23 (-75.90, 94.54) | -60.82 (-137.64, 15.77) | -44.96 (-153.51, 61.54) | -35.10 (-132.96, 60.61) |
| -43.94 (-115.41, 27.10) | -45.24 (-111.78, 22.76) | -67.35 (-131.27, 0.81) | -10.23 (-94.54, 75.90) | Laser | -70.72 (-109.05, -29.71) | -55.52 (-138.86, 33.38) | -44.83 (-88.31, 0.54) |
| 26.28 (-57.30, 106.73) | 25.57 (-27.68, 79.97) | 3.43 (-50.90, 55.87) | 60.82 (-15.77, 137.64) | 70.72 (29.71, 109.05) | Placebo | 16.19 (-60.01, 91.33) | 25.95 (-33.34, 83.79) |
| 10.71 (-100.07, 122.07) | 9.36 (-82.54, 101.74) | -12.43 (-108.22, 82.33) | 44.96 (-61.54, 153.51) | 55.52 (-33.38, 138.86) | -16.19 (-91.33, 60.01) | Silver nitrate | 10.45 (-86.44, 107.98) |
| 0.75 (-55.29, 54.60) | -0.45 (-80.36, 80.83) | -22.44 (-101.81, 56.96) | 35.10 (-60.61, 132.96) | 44.83 (-0.54, 88.31) | -25.95 (-83.79, 33.34) | -10.45 (-107.98, 86.44) | Triamcinolone |

2.2 Variance Calculation

| Parameter | Median (95% CI) |
|---|---|
| Random Effects Standard Deviation | 33.45 (18.80, 66.98) |
| Inconsistency Standard Deviation | 48.39 (2.30, 92.92) |

2.3 Convergence Diagnostics

| Parameter | PSRF |
|---|---|
| d.Curcumin.Triamcinolone | 1.00 |
| d.Laser.Placebo | 1.00 |
| d.Placebo.Doxycycline | 1.00 |
| d.Placebo.Glycyrrhiza | 1.00 |
| d.Placebo.Insulinliposomalgel | 1.02 |
| d.Placebo.Silvernitrate | 1.00 |
| d.Triamcinolone.Laser | 1.00 |
| sd.d | 1.00 |
| sd.w | 1.00 |

Number of chains : 4
Tuning iterations : 20,000
Simulation iterations : 50,000
Thinning interval : 10
Inference samples : 10,000
Variance scaling factor: 2.5

**4. Network structure**

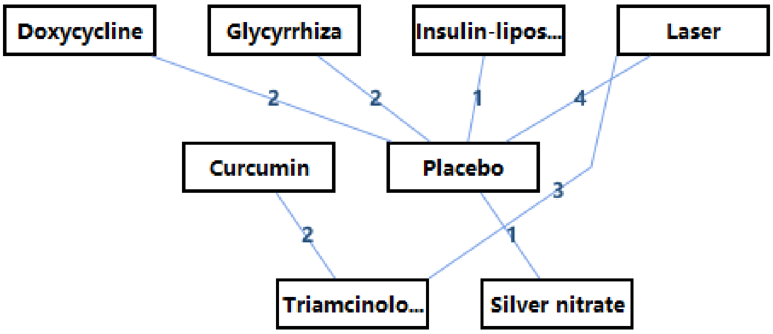

**Day 2**

## 1. Consistency Model

### 1.1 Summary estimates

| | | | | | | | | | | |
|---|---|---|---|---|---|---|---|---|---|---|
| Allicin | 3.40 (-89.94, 97.62) | 9.51 (-83.70, 101.89) | 3.74 (-93.95, 105.03) | 6.08 (-96.67, 105.08) | 19.35 (-89.84, 128.27) | 15.07 (-92.09, 125.58) | -2.61 (-98.28, 94.89) | 26.29 (-56.93, 107.58) | -12.57 (-92.44, 67.22) | 56.63 (-42.74, 155.52) |
| -3.40 (-97.62, 89.94) | Aloe | 6.20 (-81.94, 90.51) | -0.23 (-92.63, 96.77) | 2.31 (-94.38, 99.30) | 15.66 (-78.25, 110.04) | 11.39 (-85.34, 105.51) | -5.58 (-100.22, 86.05) | 22.71 (-54.15, 96.85) | -16.06 (-90.42, 54.32) | 52.59 (-41.81, 146.80) |
| -9.51 (-101.89, 83.70) | -6.20 (-90.51, 81.94) | Amlexanox | -5.76 (-95.41, 86.37) | -3.42 (-96.59, 90.19) | 9.20 (-87.72, 111.65) | 4.95 (-91.35, 106.57) | -11.60 (-103.14, 79.50) | 16.40 (-54.39, 89.18) | -22.11 (-84.57, 39.12) | 47.10 (-46.17, 139.35) |
| -3.74 (-105.03, 93.95) | 0.23 (-96.77, 92.63) | 5.76 (-86.37, 95.41) | Berberine gelatin | 1.39 (-98.18, 99.64) | 15.13 (-94.57, 124.29) | 11.00 (-96.27, 119.35) | -5.45 (-103.22, 90.01) | 22.57 (-59.82, 101.90) | -16.35 (-98.78, 61.26) | 53.13 (-50.55, 151.51) |
| -6.08 (-105.08, 96.67) | -2.31 (-99.30, 94.38) | 3.42 (-90.19, 96.59) | -1.39 (-99.64, 98.18) | Chitosan | 13.40 (-96.90, 124.19) | 9.47 (-98.86, 120.83) | -7.91 (-106.05, 89.84) | 20.58 (-62.06, 105.53) | -18.48 (-101.37, 62.23) | 50.68 (-51.85, 149.69) |
| -19.35 (-128.27, 89.84) | -15.66 (-110.04, 78.25) | -9.20 (-111.65, 87.72) | -15.13 (-124.29, 94.57) | -13.40 (-124.19, 96.90) | Chlorhexidine | -3.87 (-103.26, 93.69) | -21.28 (-129.26, 85.89) | 7.04 (-86.29, 98.46) | -31.41 (-124.57, 57.78) | 37.63 (-71.27, 144.35) |
| -15.07 (-125.58, 92.09) | -11.39 (-105.51, 85.34) | -4.95 (-106.57, 91.35) | -11.00 (-119.35, 96.27) | -9.47 (-120.83, 98.86) | 3.87 (-93.69, 103.26) | Curcumin | -17.04 (-127.45, 88.97) | 11.43 (-83.21, 102.61) | -26.85 (-119.65, 61.10) | 41.30 (-67.99, 146.83) |
| 2.61 (-94.89, 98.28) | 5.58 (-86.05, 100.22) | 11.60 (-79.50, 103.14) | 5.45 (-90.01, 103.22) | 7.91 (-89.84, 106.05) | 21.28 (-85.89, 129.26) | 17.04 (-88.97, 127.45) | Diosmectite | 28.11 (-51.30, 107.09) | -10.93 (-91.09, 70.94) | 58.58 (-40.87, 160.24) |
| -26.29 (-107.58, 56.93) | -22.71 (-96.85, 54.15) | -16.40 (-89.18, 54.39) | -22.57 (-101.90, 59.82) | -20.58 (-105.53, 62.06) | -7.04 (-98.46, 86.29) | -11.43 (-102.61, 83.21) | -28.11 (-107.09, 51.30) | Doxycycline | -38.99 (-95.11, 17.90) | 30.38 (-50.84, 109.62) |
| 12.57 (-67.22, 92.44) | 16.06 (-54.32, 90.42) | 22.11 (-39.12, 84.57) | 16.35 (-61.26, 98.78) | 18.48 (-62.23, 101.37) | 31.41 (-57.78, 124.57) | 26.85 (-61.10, 119.65) | 10.93 (-70.94, 91.09) | 38.99 (-17.90, 95.11) | Glycyrrhiza | 69.13 (-11.98, 149.48) |
| -56.63 (-155.52, 42.74) | -52.59 (-146.80, 41.81) | -47.10 (-139.35, 46.17) | -53.13 (-151.51, 50.55) | -50.68 (-149.69, 51.85) | -37.63 (-144.35, 71.27) | -41.30 (-146.83, 67.99) | -58.58 (-160.24, 40.87) | -30.38 (-109.62, 50.84) | -69.13 (-149.48, 11.98) | Insulin-liposomal gel |
| -29.49 (-105.91, 50.27) | -25.25 (-92.58, 45.15) | -19.54 (-79.89, 42.15) | -25.17 (-100.87, 55.25) | -22.69 (-103.82, 55.87) | -9.82 (-92.50, 76.63) | -14.09 (-97.34, 74.23) | -31.11 (-107.00, 45.11) | -2.86 (-54.31, 50.47) | -41.82 (-87.94, 5.75) | 27.64 (-50.14, 103.60) |
| -34.56 (-165.85, 90.71) | -31.16 (-148.57, 86.08) | -24.50 (-152.37, 94.27) | -30.67 (-160.78, 95.77) | -28.81 (-160.42, 101.28) | -16.21 (-85.68, 53.57) | -19.47 (-139.62, 99.23) | -37.14 (-166.50, 89.64) | -8.54 (-125.04, 102.10) | -47.78 (-164.05, 64.27) | 22.37 (-110.27, 145.21) |
| 6.22 (-64.16, 76.51) | 9.95 (-52.65, 74.05) | 16.28 (-43.76, 75.25) | 10.06 (-58.15, 81.57) | 12.41 (-60.34, 85.16) | 25.60 (-56.45, 108.22) | 21.33 (-59.51, 107.67) | 4.59 (-64.75, 73.85) | 32.47 (-9.40, 73.44) | -6.31 (-46.47, 32.85) | 62.85 (-6.66, 132.31) |
| -13.22 (-95.10, 67.89) | -9.42 (-70.88, 54.10) | -3.68 (-76.70, 64.27) | -9.37 (-88.99, 74.07) | -7.17 (-91.53, 74.67) | 6.23 (-64.54, 77.41) | 1.58 (-69.17, 73.02) | -14.80 (-96.80, 64.63) | 12.73 (-46.42, 69.79) | -25.60 (-83.09, 28.76) | 43.60 (-38.94, 122.50) |
| -14.46 (-108.70, 78.86) | -10.58 (-93.87, 73.83) | -4.99 (-90.65, 79.91) | -10.68 (-104.10, 83.98) | -8.83 (-106.12, 88.51) | 4.33 (-87.33, 99.20) | 0.65 (-92.87, 98.14) | -16.63 (-107.63, 76.71) | 11.50 (-63.35, 86.18) | -26.77 (-100.51, 45.24) | 42.77 (-56.01, 136.19) |
| -28.43 (-124.59, 71.60) | -25.03 (-119.07, 70.44) | -19.51 (-110.16, 73.63) | -25.38 (-123.16, 80.33) | -22.95 (-122.65, 78.43) | -9.39 (-117.58, 102.05) | -13.45 (-120.23, 98.01) | -30.48 (-128.99, 67.54) | -3.04 (-82.75, 80.78) | -41.47 (-119.63, 39.25) | 27.71 (-70.59, 129.03) |

| | | | | | |
|---|---|---|---|---|---|
| 29.49 (-50.27, 105.91) | 34.56 (-90.71, 165.85) | -6.22 (-76.51, 64.16) | 13.22 (-67.89, 95.10) | 14.46 (-78.86, 108.70) | 28.43 (-71.60, 124.59) |
| 25.25 (-45.15, 92.58) | 31.16 (-86.08, 148.57) | -9.95 (-74.05, 52.65) | 9.42 (-54.10, 70.88) | 10.58 (-73.83, 93.87) | 25.03 (-70.44, 119.07) |
| 19.54 (-42.15, 79.89) | 24.50 (-94.27, 152.37) | -16.28 (-75.25, 43.76) | 3.68 (-64.27, 76.70) | 4.99 (-79.91, 90.65) | 19.51 (-73.63, 110.16) |
| 25.17 (-55.25, 100.87) | 30.67 (-95.77, 160.78) | -10.06 (-81.57, 58.15) | 9.37 (-74.07, 88.99) | 10.68 (-83.98, 104.10) | 25.38 (-80.33, 123.16) |
| 22.69 (-55.87, 103.82) | 28.81 (-101.28, 160.42) | -12.41 (-85.16, 60.34) | 7.17 (-74.67, 91.53) | 8.83 (-88.51, 106.12) | 22.95 (-78.43, 122.65) |
| 9.82 (-76.63, 92.50) | 16.21 (-53.57, 85.68) | -25.60 (-108.22, 56.45) | -6.23 (-77.41, 64.54) | -4.33 (-99.20, 87.33) | 9.39 (-102.05, 117.58) |
| 14.09 (-74.23, 97.34) | 19.47 (-99.23, 139.62) | -21.33 (-107.67, 59.51) | -1.58 (-73.02, 69.17) | -0.65 (-98.14, 92.87) | 13.45 (-98.01, 120.23) |
| 31.11 (-45.11, 107.00) | 37.14 (-89.64, 166.50) | -4.59 (-73.85, 64.75) | 14.80 (-64.63, 96.80) | 16.63 (-76.71, 107.63) | 30.48 (-67.54, 128.99) |
| 2.86 (-50.47, 54.31) | 8.54 (-102.10, 125.04) | -32.47 (-73.44, 9.40) | -12.73 (-69.79, 46.42) | -11.50 (-86.18, 63.35) | 3.04 (-80.78, 82.75) |
| 41.82 (-5.75, 87.94) | 47.78 (-64.27, 164.05) | 6.31 (-32.85, 46.47) | 25.60 (-28.76, 83.09) | 26.77 (-45.24, 100.51) | 41.47 (-39.25, 119.63) |
| -27.64 (-103.60, 50.14) | -22.37 (-145.21, 110.27) | -62.85 (-132.31, 6.66) | -43.60 (-122.50, 38.94) | -42.77 (-136.19, 56.01) | -27.71 (-129.03, 70.59) |
| Laser | 5.89 (-101.97, 117.71) | -35.33 (-68.12, -0.74) | -16.00 (-60.86, 31.63) | -14.36 (-81.77, 55.86) | -0.64 (-78.36, 76.07) |
| -5.89 (-117.71, 101.97) | N-acetylcysteine | -40.81 (-149.04, 65.00) | -21.80 (-121.29, 76.19) | -19.88 (-138.34, 97.45) | -6.16 (-136.72, 120.53) |
| 35.33 (0.74, 68.12) | 40.81 (-65.00, 149.04) | Placebo | 19.69 (-21.03, 61.57) | 21.17 (-41.69, 83.57) | 35.36 (-36.44, 104.58) |
| 16.00 (-31.63, 60.86) | 21.80 (-76.19, 121.29) | -19.69 (-61.57, 21.03) | Triamcinolone | 1.62 (-62.58, 63.40) | 15.26 (-67.19, 97.45) |
| 14.36 (-55.86, 81.77) | 19.88 (-97.45, 138.34) | -21.17 (-83.57, 41.69) | -1.62 (-63.40, 62.58) | Triester Glycerol Oxide | 14.37 (-80.76, 107.03) |
| 0.64 (-76.07, 78.36) | 6.16 (-120.53, 136.72) | -35.36 (-104.58, 36.44) | -15.26 (-97.45, 67.19) | -14.37 (-107.03, 80.76) | Zinc |

## 1.2 Rank probability(Rank 1 is best, rank N is worst)

| Drug | Rank 1 | Rank 2 | Rank 3 | Rank 4 | Rank 5 | Rank 6 | Rank 7 | Rank 8 | Rank 9 | Rank 10 | Rank 11 | Rank 12 | Rank 13 | Rank 14 | Rank 15 | Rank 16 | Rank 17 |
|---|---|---|---|---|---|---|---|---|---|---|---|---|---|---|---|---|---|
| Allicin | 0.02 | 0.03 | 0.04 | 0.04 | 0.04 | 0.05 | 0.05 | 0.05 | 0.06 | 0.06 | 0.06 | 0.06 | 0.06 | 0.07 | 0.08 | 0.1 | 0.13 |
| Aloe | 0.01 | 0.03 | 0.03 | 0.04 | 0.04 | 0.05 | 0.06 | 0.06 | 0.07 | 0.07 | 0.07 | 0.07 | 0.07 | 0.07 | 0.08 | 0.08 | 0.08 |
| Amlexanox | 0.02 | 0.04 | 0.05 | 0.05 | 0.06 | 0.07 | 0.07 | 0.07 | 0.07 | 0.08 | 0.07 | 0.07 | 0.06 | 0.06 | 0.07 | 0.06 | 0.05 |
| Berberine gelatin | 0.03 | 0.03 | 0.04 | 0.05 | 0.05 | 0.05 | 0.05 | 0.05 | 0.06 | 0.06 | 0.06 | 0.06 | 0.06 | 0.07 | 0.07 | 0.09 | 0.1 |
| Chitosan | 0.03 | 0.05 | 0.05 | 0.05 | 0.05 | 0.05 | 0.05 | 0.05 | 0.06 | 0.06 | 0.06 | 0.06 | 0.06 | 0.06 | 0.07 | 0.08 | 0.11 |
| Chlorhexidine | 0.03 | 0.09 | 0.1 | 0.08 | 0.07 | 0.06 | 0.06 | 0.06 | 0.05 | 0.05 | 0.05 | 0.04 | 0.04 | 0.05 | 0.05 | 0.06 | 0.05 |
| Curcumin | 0.06 | 0.07 | 0.07 | 0.07 | 0.06 | 0.06 | 0.05 | 0.06 | 0.05 | 0.05 | 0.05 | 0.05 | 0.05 | 0.05 | 0.05 | 0.06 | 0.08 |
| Diosmectite | 0.02 | 0.03 | 0.03 | 0.04 | 0.04 | 0.04 | 0.05 | 0.05 | 0.05 | 0.06 | 0.06 | 0.06 | 0.06 | 0.07 | 0.08 | 0.11 | 0.14 |
| Doxycycline | 0.03 | 0.08 | 0.1 | 0.11 | 0.11 | 0.11 | 0.1 | 0.09 | 0.07 | 0.06 | 0.05 | 0.03 | 0.03 | 0.02 | 0.01 | 0.01 | 0 |
| Glycyrrhiza | 0 | 0 | 0 | 0 | 0.01 | 0.01 | 0.02 | 0.03 | 0.04 | 0.05 | 0.07 | 0.08 | 0.11 | 0.12 | 0.16 | 0.17 | 0.13 |
| Insulin-liposomal gel | 0.38 | 0.18 | 0.11 | 0.07 | 0.05 | 0.04 | 0.03 | 0.02 | 0.02 | 0.02 | 0.02 | 0.01 | 0.01 | 0.01 | 0.01 | 0.01 | 0.01 |
| Laser | 0.02 | 0.07 | 0.11 | 0.14 | 0.14 | 0.13 | 0.11 | 0.09 | 0.07 | 0.05 | 0.03 | 0.02 | 0.01 | 0.01 | 0 | 0 | 0 |
| N-acetylcysteine | 0.22 | 0.13 | 0.08 | 0.06 | 0.05 | 0.05 | 0.04 | 0.04 | 0.04 | 0.03 | 0.03 | 0.03 | 0.03 | 0.03 | 0.03 | 0.04 | 0.05 |
| Placebo | 0 | 0 | 0 | 0 | 0 | 0 | 0.01 | 0.02 | 0.04 | 0.07 | 0.11 | 0.16 | 0.2 | 0.2 | 0.13 | 0.06 | 0.01 |
| Triamcinolone | 0 | 0.01 | 0.02 | 0.04 | 0.07 | 0.09 | 0.11 | 0.12 | 0.13 | 0.12 | 0.1 | 0.07 | 0.06 | 0.04 | 0.02 | 0.01 | 0 |
| Triester Glycerol Oxide | 0.03 | 0.05 | 0.06 | 0.07 | 0.07 | 0.07 | 0.08 | 0.08 | 0.07 | 0.07 | 0.06 | 0.06 | 0.05 | 0.05 | 0.05 | 0.05 | 0.04 |
| Zinc | 0.1 | 0.12 | 0.1 | 0.08 | 0.08 | 0.07 | 0.06 | 0.05 | 0.05 | 0.05 | 0.04 | 0.04 | 0.03 | 0.03 | 0.03 | 0.03 | 0.03 |

### 1.3 Consistency check

| Parameter | Median (95% CI) |
|---|---|
| Random Effects Standard Deviation | 30.83 (18.70, 56.88) |

### 1.4 Convergence Diagnostics

| Parameter | PSRF |
|---|---|
| d.Chlorhexidine.Nacetylcysteine | 1.00 |
| d.Placebo.Allicin | 1.00 |
| d.Placebo.Amlexanox | 1.00 |
| d.Placebo.Berberinegelatin | 1.00 |
| d.Placebo.Chitosan | 1.00 |
| d.Placebo.Diosmectite | 1.00 |
| d.Placebo.Doxycycline | 1.00 |
| d.Placebo.Glycyrrhiza | 1.00 |
| d.Placebo.Insulinliposomalgel | 1.00 |
| d.Placebo.Zinc | 1.00 |
| d.Triamcinolone.Aloe | 1.00 |
| d.Triamcinolone.Chlorhexidine | 1.00 |
| d.Triamcinolone.Curcumin | 1.00 |
| d.Triamcinolone.Laser | 1.00 |
| d.Triamcinolone.Placebo | 1.00 |
| d.Triamcinolone.TriesterGlycerol... | 1.00 |
| sd.d | 1.00 |

Number of chains : 4
Tuning iterations : 20,000
Simulation iterations : 50,000
Thinning interval : 10
Inference samples : 10,000
Variance scaling factor: 2.5

## 2.Inconsistency Model

### 2.1 Summary estimates

| | | | | | | |
|---|---|---|---|---|---|---|
| Allicin | 0.57 (-91.04, 92.65) | -8.17 (-107.31, 95.14) | 3.67 (-92.49, 97.91) | 4.88 (-95.09, 102.50) | 12.05 (-94.00, 122.61) | 8.28 (-98.60, 112.96) |
| -0.57 (-92.65, 91.04) | Aloe | -8.52 (-102.22, 85.71) | 3.12 (-89.10, 93.14) | 5.19 (-89.16, 96.18) | 11.50 (-83.21, 105.19) | 7.28 (-87.59, 98.59) |
| 8.17 (-95.14, 107.31) | 8.52 (-85.71, 102.22) | Amlexanox | 11.42 (-89.14, 110.47) | 12.40 (-90.88, 113.48) | 20.47 (-89.62, 124.61) | 15.78 (-90.52, 119.68) |
| -3.67 (-97.91, 92.49) | -3.12 (-93.14, 89.10) | -11.42 (-110.47, 89.14) | Berberine gelatin | 1.90 (-96.14, 98.24) | 9.03 (-97.27, 115.03) | 4.14 (-102.24, 111.37) |
| -4.88 (-102.50, 95.09) | -5.19 (-96.18, 89.16) | -12.40 (-113.48, 90.88) | -1.90 (-98.24, 96.14) | Chitosan | 6.86 (-101.00, 115.22) | 3.20 (-103.89, 109.15) |
| -12.05 (-122.61, 94.00) | -11.50 (-105.19, 83.21) | -20.47 (-124.61, 89.62) | -9.03 (-115.03, 97.27) | -6.86 (-115.22, 101.00) | Chlorhexidine | -3.78 (-106.82, 91.86) |
| -8.28 (-112.96, 98.60) | -7.28 (-98.59, 87.59) | -15.78 (-119.68, 90.52) | -4.14 (-111.37, 102.24) | -3.20 (-109.15, 103.89) | 3.78 (-91.86, 106.82) | Curcumin |
| 1.87 (-94.40, 99.94) | 2.00 (-88.75, 97.54) | -5.75 (-105.31, 96.63) | 4.90 (-88.79, 100.25) | 6.86 (-91.45, 105.30) | 13.80 (-91.54, 124.83) | 9.68 (-96.24, 115.92) |
| -25.84 (-103.01, 54.72) | -25.44 (-95.41, 49.10) | -33.39 (-115.76, 53.63) | -22.38 (-99.36, 58.52) | -20.54 (-101.78, 61.47) | -13.57 (-104.97, 80.29) | -17.45 (-106.93, 72.45) |
| 19.94 (-69.38, 110.60) | 20.74 (-60.02, 106.27) | 11.95 (-50.41, 79.42) | 23.50 (-65.79, 113.16) | 24.72 (-67.47, 116.87) | 32.52 (-64.35, 130.35) | 27.84 (-66.00, 125.10) |
| -56.54 (-151.60, 41.49) | -56.17 (-145.55, 34.45) | -64.62 (-162.01, 37.48) | -52.67 (-149.39, 43.14) | -51.33 (-149.55, 46.18) | -44.70 (-151.02, 63.74) | -48.96 (-154.57, 58.09) |
| -8.69 (-98.69, 82.35) | -7.93 (-85.31, 73.10) | -16.09 (-76.53, 45.77) | -5.21 (-94.18, 83.88) | -3.27 (-96.98, 87.18) | 4.11 (-90.90, 95.73) | 0.15 (-90.18, 87.26) |
| -28.04 (-157.85, 101.79) | -27.13 (-148.34, 93.20) | -36.02 (-160.43, 93.54) | -24.07 (-153.77, 106.64) | -23.13 (-152.42, 109.46) | -16.03 (-85.50, 52.07) | -19.75 (-142.65, 99.46) |
| 6.05 (-59.80, 75.65) | 6.70 (-52.44, 70.69) | 12.46 (-70.77, 94.50) | 9.75 (-57.83, 78.11) | 11.54 (-59.63, 82.46) | 18.62 (-64.01, 102.47) | 13.99 (-67.11, 95.92) |
| -5.80 (-88.67, 76.05) | -5.28 (-65.43, 55.58) | -14.31 (-89.91, 64.68) | -2.33 (-84.44, 75.62) | -0.97 (-84.76, 80.90) | 5.34 (-64.41, 77.90) | 2.01 (-69.58, 69.78) |
| -11.85 (-106.97, 81.91) | -11.58 (-92.55, 70.57) | -19.35 (-112.88, 77.14) | -8.22 (-104.89, 83.80) | -6.58 (-103.77, 85.99) | 0.05 (-93.31, 94.59) | -3.89 (-99.75, 88.40) |
| -28.21 (-123.86, 67.75) | -28.13 (-118.20, 64.25) | -36.44 (-133.41, 65.67) | -24.71 (-121.42, 69.38) | -22.70 (-123.42, 75.87) | -16.57 (-121.28, 92.96) | -20.91 (-125.09, 86.47) |

| | | | | | | | | | |
|---|---|---|---|---|---|---|---|---|---|
| -1.87 (-99.94, 94.40) | 25.84 (-54.72, 103.01) | -19.94 (-110.60, 69.38) | 56.54 (-41.49, 151.60) | 8.69 (-82.35, 98.69) | 28.04 (-101.79, 157.85) | -6.05 (-75.65, 59.80) | 5.80 (-76.05, 88.67) | 11.85 (-81.91, 106.97) | 28.21 (-67.75, 123.86) |
| -2.00 (-97.54, 88.75) | 25.44 (-49.10, 95.41) | -20.74 (-106.27, 60.02) | 56.17 (-34.45, 145.55) | 7.93 (-73.10, 85.31) | 27.13 (-93.20, 148.34) | -6.70 (-70.69, 52.44) | 5.28 (-55.58, 65.43) | 11.58 (-70.57, 92.55) | 28.13 (-64.25, 118.20) |
| 5.75 (-96.63, 105.31) | 33.39 (-53.63, 115.76) | -11.95 (-79.42, 50.41) | 64.62 (-37.48, 162.01) | 16.09 (-45.77, 76.53) | 36.02 (-93.54, 160.43) | -12.46 (-94.50, 70.77) | 14.31 (-64.68, 89.91) | 19.35 (-77.14, 112.88) | 36.44 (-65.67, 133.41) |
| -4.90 (-100.25, 88.79) | 22.38 (-58.52, 99.36) | -23.50 (-113.16, 65.79) | 52.67 (-43.14, 149.39) | 5.21 (-83.88, 94.18) | 24.07 (-106.64, 153.77) | -9.75 (-78.11, 57.83) | 2.33 (-75.62, 84.44) | 8.22 (-83.80, 104.89) | 24.71 (-69.38, 121.42) |
| -6.86 (-105.30, 91.45) | 20.54 (-61.47, 101.78) | -24.72 (-116.87, 67.47) | 51.33 (-46.18, 149.55) | 3.27 (-87.18, 96.98) | 23.13 (-109.46, 152.42) | -11.54 (-82.46, 59.63) | 0.97 (-80.90, 84.76) | 6.58 (-85.99, 103.77) | 22.70 (-75.87, 123.42) |
| -13.80 (-124.83, 91.54) | 13.57 (-80.29, 104.97) | -32.52 (-130.35, 64.35) | 44.70 (-63.74, 151.02) | -4.11 (-95.73, 90.90) | 16.03 (-52.07, 85.50) | -18.62 (-102.47, 64.01) | -5.34 (-77.90, 64.41) | -0.05 (-94.59, 93.31) | 16.57 (-92.96, 121.28) |
| -9.68 (-115.92, 96.24) | 17.45 (-72.45, 106.93) | -27.84 (-125.10, 66.00) | 48.96 (-58.09, 154.57) | -0.15 (-87.26, 90.18) | 19.75 (-99.46, 142.65) | -13.99 (-95.92, 67.11) | -2.01 (-69.78, 69.58) | 3.89 (-88.40, 99.75) | 20.91 (-86.47, 125.09) |
| Diosmectite | 27.44 (-52.00, 106.47) | -18.22 (-108.64, 71.45) | 58.73 (-35.87, 153.82) | 10.18 (-76.43, 99.96) | 29.54 (-102.57, 163.99) | -4.78 (-73.08, 63.50) | 7.82 (-71.26, 90.56) | 13.19 (-76.00, 108.44) | 30.27 (-66.63, 125.15) |
| -27.44 (-106.47, 52.00) | Doxycycline | -45.06 (-119.85, 25.08) | 30.71 (-47.09, 108.95) | -17.02 (-86.86, 51.87) | 2.23 (-114.31, 120.71) | -32.08 (-74.10, 7.39) | -19.57 (-77.20, 42.34) | -13.61 (-88.15, 62.31) | 2.95 (-77.09, 80.75) |
| 18.22 (-71.45, 108.64) | 45.06 (-25.08, 119.85) | Glycyrrhiza | 77.22 (-15.05, 168.39) | 28.80 (-26.28, 84.53) | 48.38 (-73.96, 170.94) | 17.31 (-28.07, 61.19) | 26.09 (-37.52, 94.50) | 31.66 (-54.47, 118.29) | 48.20 (-42.08, 141.04) |
| -58.73 (-153.82, 35.87) | -30.71 (-108.95, 47.09) | -77.22 (-168.39, 15.05) | Insulin-liposomal gel | -48.29 (-137.98, 42.27) | -28.13 (-157.14, 100.25) | -62.97 (-131.39, 5.72) | -50.82 (-128.96, 30.35) | -44.46 (-137.86, 48.74) | -28.57 (-124.37, 69.17) |
| -10.18 (-99.96, 76.43) | 17.02 (-51.87, 86.86) | -28.80 (-84.53, 26.28) | 48.29 (-42.27, 137.98) | Laser | 20.35 (-98.03, 136.11) | -28.53 (-97.33, 43.83) | -2.44 (-58.54, 56.23) | 3.56 (-76.77, 82.97) | 19.57 (-67.55, 110.40) |
| -29.54 (-163.99, 102.57) | -2.23 (-120.71, 114.31) | -48.38 (-170.94, 73.96) | 28.13 (-100.25, 157.14) | -20.35 (-136.11, 98.03) | N-acetylcysteine | -34.42 (-145.38, 75.47) | -21.71 (-123.43, 80.01) | -15.49 (-139.26, 105.04) | 0.63 (-132.36, 128.30) |
| 4.78 (-63.50, 73.08) | 32.08 (-7.39, 74.10) | -17.31 (-61.19, 28.07) | 62.97 (-5.72, 131.39) | 28.53 (-43.83, 97.33) | 34.42 (-75.47, 145.38) | Placebo | 12.29 (-29.47, 58.66) | 18.50 (-42.97, 82.61) | 34.99 (-33.54, 101.57) |
| -7.82 (-90.56, 71.26) | 19.57 (-42.34, 77.20) | -26.09 (-94.50, 37.52) | 50.82 (-30.35, 128.96) | 2.44 (-56.23, 58.54) | 21.71 (-80.01, 123.43) | -12.29 (-58.66, 29.47) | Triamcinolone | 5.57 (-57.38, 69.33) | 22.09 (-60.36, 104.20) |
| -13.19 (-108.44, 76.00) | 13.61 (-62.31, 88.15) | -31.66 (-118.29, 54.47) | 44.46 (-48.74, 137.86) | -3.56 (-82.97, 76.77) | 15.49 (-105.04, 139.26) | -18.50 (-82.61, 42.97) | -5.57 (-69.33, 57.38) | Triester Glycerol Oxide | 16.90 (-78.33, 106.91) |
| -30.27 (-125.15, 66.63) | -2.95 (-80.75, 77.09) | -48.20 (-141.04, 42.08) | 28.57 (-69.17, 124.37) | -19.57 (-110.40, 67.55) | -0.63 (-128.30, 132.36) | -34.99 (-101.57, 33.54) | -22.09 (-104.20, 60.36) | -16.90 (-106.91, 78.33) | Zinc |

## 2.2 Inconsistency Factors

| Cycle | Median (95% CI) |
|---|---|
| Glycyrrhiza, Laser, Placebo, Triamcinolone | 2.55 (-55.03, 60.50) |
| Laser, Placebo, Triamcinolone | 9.41 (-48.09, 83.17) |
| Laser, Placebo, Triamcinolone | 9.41 (-48.09, 83.17) |

2.3 Variance Calculation

| Parameter | Median (95% CI) |
|---|---|
| Random Effects Standard Deviation | 30.07 (17.32, 57.49) |
| Inconsistency Standard Deviation | 31.46 (2.78, 74.72) |

2.4 Convergence Diagnostics

| Parameter | PSRF |
|---|---|
| d.Allicin.Placebo | 1.00 |
| d.Aloe.Triamcinolone | 1.01 |
| d.Amlexanox.Glycyrrhiza | 1.00 |
| d.Chlorhexidine.Nacetylcysteine | 1.00 |
| d.Laser.Amlexanox | 1.03 |
| d.Placebo.Aloe | 1.00 |
| d.Placebo.Berberinegelatin | 1.00 |
| d.Placebo.Chitosan | 1.00 |
| d.Placebo.Diosmectite | 1.00 |
| d.Placebo.Doxycycline | 1.00 |
| d.Placebo.Insulinliposomalgel | 1.00 |
| d.Placebo.Zinc | 1.00 |
| d.Triamcinolone.Chlorhexidine | 1.00 |
| d.Triamcinolone.Curcumin | 1.00 |
| d.Triamcinolone.Laser | 1.01 |
| d.Triamcinolone.TriesterGlycerol... | 1.00 |
| w.Glycyrrhiza.Laser.Triamcinolon... | 1.00 |
| w.Laser.Placebo.Triamcinolone | 1.01 |
| w.Laser.Placebo.Triamcinolone | 1.01 |
| sd.d | 1.01 |
| sd.w | 1.01 |

Number of chains : 4
Tuning iterations : 20,000
Simulation iterations : 50,000
Thinning interval : 10
Inference samples : 10,000
Variance scaling factor: 2.5

### 3. Node-splitting analysis

| Name | Direct Effect | Indirect Effect | Overall | P-Value |
|---|---|---|---|---|
| Glycyrrhiza, Laser | 22.17 (-50.35, 96.69) | 47.47 (-14.03, 106.87) | 41.82 (-5.75, 87.94) | 0.55 |
| Laser, Placebo | -48.05 (-80.34, -14.42) | 1.37 (-53.56, 56.74) | -35.33 (-68.12, -0.74) | 0.11 |
| Laser, Triamcinolone | 12.25 (-61.23, 86.57) | -34.09 (-94.67, 28.70) | -16.00 (-60.86, 31.63) | 0.29 |
| Placebo, Triamcinolone | 7.80 (-41.33, 55.90) | 55.55 (-27.48, 139.67) | 19.69 (-21.03, 61.57) | 0.29 |

### 4. Network structure

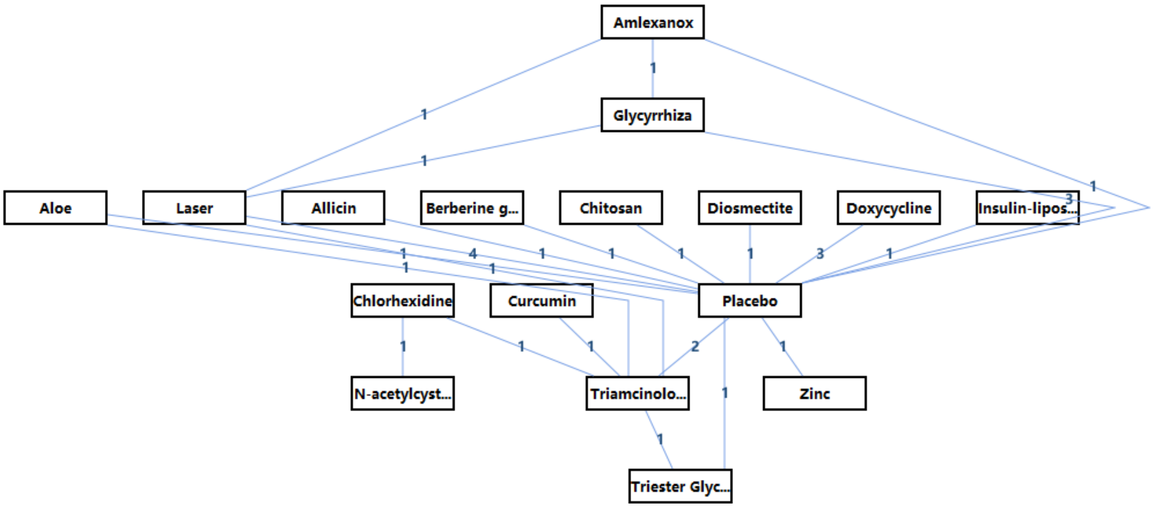

**Day 3**

## 1. Consistency Model

### 1.1 Summary estimates

| | | | | | | | | | | |
|---|---|---|---|---|---|---|---|---|---|---|
| Aloe | -9.13 (-73.82, 57.44) | 58.24 (-24.84, 139.41) | 29.60 (-52.29, 110.36) | -13.20 (-101.11, 78.74) | -3.36 (-83.68, 76.37) | 22.86 (-43.10, 85.01) | 0.27 (-69.07, 69.50) | 61.60 (-19.68, 140.69) | 46.86 (-13.22, 105.60) | -14.13 (-68.67, 39.93) |
| 9.13 (-57.44, 73.82) | Amlexanox | 66.83 (-8.92, 143.52) | 38.96 (-35.34, 114.71) | -4.16 (-66.53, 59.51) | 5.40 (-71.43, 78.71) | 31.59 (-29.19, 87.67) | 9.23 (-54.96, 69.92) | 70.28 (-6.97, 142.83) | 56.14 (4.09, 104.54) | -4.96 (-50.43, 37.96) |
| -58.24 (-139.41, 24.84) | -66.83 (-143.52, 8.92) | Chlorhexidine | -28.43 (-117.18, 59.80) | -70.95 (-168.23, 28.69) | -61.12 (-155.95, 30.59) | -35.39 (-114.68, 41.83) | -57.86 (-143.61, 23.86) | 3.43 (-90.19, 92.85) | -10.93 (-82.32, 59.07) | -71.92 (-142.56, -1.15) |
| -29.60 (-110.36, 52.29) | -38.96 (-114.71, 35.34) | 28.43 (-59.80, 117.18) | Curcumin | -42.28 (-139.10, 56.86) | -32.80 (-125.26, 57.32) | -6.75 (-85.53, 68.69) | -29.10 (-109.56, 49.50) | 31.91 (-60.27, 122.99) | 17.39 (-51.96, 86.16) | -43.73 (-112.15, 23.90) |
| 13.20 (-78.74, 101.11) | 4.16 (-59.51, 66.53) | 70.95 (-28.69, 168.23) | 42.28 (-56.86, 139.10) | Dexamethasone | 10.06 (-89.53, 104.54) | 35.63 (-50.29, 118.63) | 13.62 (-78.11, 98.67) | 74.78 (-25.07, 168.47) | 59.96 (-19.85, 137.81) | -1.15 (-79.00, 73.21) |
| 3.36 (-76.37, 83.68) | -5.40 (-78.71, 71.43) | 61.12 (-30.59, 155.95) | 32.80 (-57.32, 125.26) | -10.06 (-104.54, 89.53) | Diosmectite | 26.04 (-44.12, 95.62) | 3.72 (-71.43, 78.24) | 64.56 (-19.38, 147.67) | 50.19 (-16.83, 118.73) | -10.83 (-69.15, 49.83) |
| -22.86 (-85.01, 43.10) | -31.59 (-87.67, 29.19) | 35.39 (-41.83, 114.68) | 6.75 (-68.69, 85.53) | -35.63 (-118.63, 50.29) | -26.04 (-95.62, 44.12) | Doxycycline | -22.80 (-77.75, 34.90) | 38.74 (-29.50, 108.94) | 24.10 (-23.43, 72.37) | -36.76 (-71.84, 0.37) |
| -0.27 (-69.50, 69.07) | -9.23 (-69.92, 54.96) | 57.86 (-23.86, 143.61) | 29.10 (-49.50, 109.56) | -13.62 (-98.67, 78.11) | -3.72 (-78.24, 71.43) | 22.80 (-34.90, 77.75) | Glycyrrhiza | 60.89 (-10.83, 134.76) | 46.81 (-6.24, 101.02) | -14.03 (-57.80, 29.48) |
| -61.60 (-140.69, 19.68) | -70.28 (-142.83, 6.97) | -3.43 (-92.85, 90.19) | -31.91 (-122.99, 60.27) | -74.78 (-168.47, 25.07) | -64.56 (-147.67, 19.38) | -38.74 (-108.94, 29.50) | -60.89 (-134.76, 10.83) | Insulin-liposomal gel | -14.35 (-82.01, 53.90) | -75.51 (-134.42, -14.96) |
| -46.86 (-105.60, 13.22) | -56.14 (-104.54, -4.09) | 10.93 (-59.07, 82.32) | -17.39 (-86.16, 51.96) | -59.96 (-137.81, 19.85) | -50.19 (-118.73, 16.83) | -24.10 (-72.37, 23.43) | -46.81 (-101.02, 6.24) | 14.35 (-53.90, 82.01) | Laser | -61.05 (-92.61, -29.60) |
| 14.13 (-39.93, 68.67) | 4.96 (-37.96, 50.43) | 71.92 (1.15, 142.56) | 43.73 (-23.90, 112.15) | 1.15 (-73.21, 79.00) | 10.83 (-49.83, 69.15) | 36.76 (-0.37, 71.84) | 14.03 (-29.48, 57.80) | 75.51 (14.96, 134.42) | 61.05 (29.60, 92.61) | Placebo |
| 5.98 (-61.32, 73.53) | -2.75 (-64.12, 57.58) | 63.64 (-15.11, 144.57) | 36.06 (-42.88, 115.66) | -6.69 (-93.03, 79.84) | 2.82 (-69.87, 75.09) | 28.73 (-26.37, 83.25) | 6.20 (-54.98, 65.87) | 67.46 (-5.40, 140.36) | 52.91 (1.21, 104.12) | -8.11 (-49.17, 34.11) |
| -6.45 (-85.81, 72.96) | -15.38 (-88.67, 57.50) | 51.08 (-36.22, 142.43) | 22.62 (-67.11, 111.76) | -19.19 (-116.97, 76.94) | -10.22 (-95.10, 76.12) | 16.00 (-54.33, 84.00) | -6.85 (-78.39, 66.46) | 54.63 (-29.53, 138.81) | 40.64 (-26.14, 107.37) | -20.63 (-79.71, 37.56) |
| -77.39 (-178.97, 23.86) | -86.61 (-182.37, 7.55) | -19.81 (-78.46, 39.13) | -47.86 (-152.07, 54.57) | -90.71 (-203.98, 23.26) | -81.19 (-189.04, 25.72) | -55.15 (-152.31, 39.83) | -77.80 (-179.72, 20.96) | -16.38 (-125.12, 89.41) | -30.82 (-122.74, 57.69) | -92.29 (-183.49, -3.94) |
| -28.36 (-83.09, 23.71) | -37.42 (-82.40, 6.48) | 29.49 (-33.83, 91.32) | 1.27 (-60.03, 62.00) | -41.79 (-118.90, 36.15) | -31.54 (-100.98, 34.36) | -5.90 (-55.14, 40.25) | -28.11 (-83.69, 24.95) | 33.05 (-35.86, 100.94) | 18.51 (-15.44, 50.02) | -42.65 (-74.68, -12.28) |
| -47.12 (-126.67, 32.70) | -56.04 (-130.32, 18.89) | 10.73 (-81.42, 104.71) | -17.53 (-106.36, 74.00) | -60.03 (-155.83, 39.79) | -50.40 (-138.56, 35.55) | -24.30 (-94.77, 45.56) | -46.96 (-122.90, 25.52) | 14.70 (-71.00, 97.87) | 0.22 (-68.49, 66.46) | -61.36 (-121.02, -0.88) |

| | | | | |
|---|---|---|---|---|
| -5.98 (-73.53, 61.32) | 6.45 (-72.96, 85.81) | 77.39 (-23.86, 178.97) | 28.36 (-23.71, 83.09) | 47.12 (-32.70, 126.67) |
| 2.75 (-57.58, 64.12) | 15.38 (-57.50, 88.67) | 86.61 (-7.55, 182.37) | 37.42 (-6.48, 82.40) | 56.04 (-18.89, 130.32) |
| -63.64 (-144.57, 15.11) | -51.08 (-142.43, 36.22) | 19.81 (-39.13, 78.46) | -29.49 (-91.32, 33.83) | -10.73 (-104.71, 81.42) |
| -36.06 (-115.66, 42.88) | -22.62 (-111.76, 67.11) | 47.86 (-54.57, 152.07) | -1.27 (-62.00, 60.03) | 17.53 (-74.00, 106.36) |
| 6.69 (-79.84, 93.03) | 19.19 (-76.94, 116.97) | 90.71 (-23.26, 203.98) | 41.79 (-36.15, 118.90) | 60.03 (-39.79, 155.83) |
| -2.82 (-75.09, 69.87) | 10.22 (-76.12, 95.10) | 81.19 (-25.72, 189.04) | 31.54 (-34.36, 100.98) | 50.40 (-35.55, 138.56) |
| -28.73 (-83.25, 26.37) | -16.00 (-84.00, 54.33) | 55.15 (-39.83, 152.31) | 5.90 (-40.25, 55.14) | 24.30 (-45.56, 94.77) |
| -6.20 (-65.87, 54.98) | 6.85 (-66.46, 78.39) | 77.80 (-20.96, 179.72) | 28.11 (-24.95, 83.69) | 46.96 (-25.52, 122.90) |
| -67.46 (-140.36, 5.40) | -54.63 (-138.81, 29.53) | 16.38 (-89.41, 125.12) | -33.05 (-100.94, 35.86) | -14.70 (-97.87, 71.00) |
| -52.91 (-104.12, -1.21) | -40.64 (-107.37, 26.14) | 30.82 (-57.69, 122.74) | -18.51 (-50.02, 15.44) | -0.22 (-66.46, 68.49) |
| 8.11 (-34.11, 49.17) | 20.63 (-37.56, 79.71) | 92.29 (3.94, 183.49) | 42.65 (12.28, 74.68) | 61.36 (0.88, 121.02) |
| Probiotics | 12.44 (-57.90, 84.39) | 83.66 (-11.37, 181.98) | 34.61 (-16.07, 86.79) | 53.05 (-19.85, 127.95) |
| -12.44 (-84.39, 57.90) | Silver nitrate | 71.34 (-33.85, 176.85) | 21.78 (-43.40, 88.72) | 39.93 (-42.00, 123.78) |
| -83.66 (-181.98, 11.37) | -71.34 (-176.85, 33.85) | Sucralfate | -49.17 (-135.75, 35.44) | -30.63 (-139.82, 76.64) |
| -34.61 (-86.79, 16.07) | -21.78 (-88.72, 43.40) | 49.17 (-35.44, 135.75) | Triamcinolone | 18.72 (-50.10, 86.48) |
| -53.05 (-127.95, 19.85) | -39.93 (-123.78, 42.00) | 30.63 (-76.64, 139.82) | -18.72 (-86.48, 50.10) | Zinc |

## 1.2 Rank probability(Rank 1 is best, rank N is worst)

| Drug | Rank 1 | Rank 2 | Rank 3 | Rank 4 | Rank 5 | Rank 6 | Rank 7 | Rank 8 | Rank 9 | Rank 10 | Rank 11 | Rank 12 | Rank 13 | Rank 14 | Rank 15 | Rank 16 |
|---|---|---|---|---|---|---|---|---|---|---|---|---|---|---|---|---|
| Aloe | 0 | 0 | 0.01 | 0.02 | 0.02 | 0.03 | 0.05 | 0.08 | 0.09 | 0.11 | 0.11 | 0.11 | 0.09 | 0.09 | 0.09 | 0.1 |
| Amlexanox | 0 | 0 | 0 | 0 | 0.01 | 0.01 | 0.02 | 0.04 | 0.06 | 0.09 | 0.11 | 0.13 | 0.14 | 0.14 | 0.17 | 0.08 |
| Chlorhexidine | 0.07 | 0.29 | 0.19 | 0.13 | 0.09 | 0.06 | 0.05 | 0.03 | 0.03 | 0.02 | 0.01 | 0.01 | 0.01 | 0.01 | 0.01 | 0 |
| Curcumin | 0.05 | 0.06 | 0.09 | 0.09 | 0.1 | 0.1 | 0.09 | 0.09 | 0.08 | 0.06 | 0.05 | 0.04 | 0.03 | 0.03 | 0.03 | 0.02 |
| Dexamethasone | 0.01 | 0.01 | 0.01 | 0.02 | 0.02 | 0.03 | 0.03 | 0.05 | 0.05 | 0.06 | 0.07 | 0.07 | 0.07 | 0.08 | 0.12 | 0.3 |
| Diosmectite | 0 | 0.01 | 0.01 | 0.02 | 0.03 | 0.03 | 0.05 | 0.05 | 0.08 | 0.09 | 0.1 | 0.09 | 0.09 | 0.08 | 0.11 | 0.16 |
| Doxycycline | 0 | 0.01 | 0.03 | 0.06 | 0.08 | 0.12 | 0.14 | 0.16 | 0.13 | 0.1 | 0.07 | 0.04 | 0.02 | 0.01 | 0.01 | 0 |
| Glycyrrhiza | 0 | 0 | 0.01 | 0.01 | 0.02 | 0.03 | 0.04 | 0.07 | 0.1 | 0.12 | 0.13 | 0.12 | 0.11 | 0.09 | 0.08 | 0.07 |
| Insulin-liposomal gel | 0.24 | 0.19 | 0.18 | 0.12 | 0.08 | 0.05 | 0.04 | 0.03 | 0.02 | 0.02 | 0.01 | 0.01 | 0 | 0 | 0 | 0 |
| Laser | 0.03 | 0.1 | 0.18 | 0.23 | 0.21 | 0.12 | 0.06 | 0.03 | 0.02 | 0.01 | 0 | 0 | 0 | 0 | 0 | 0 |
| Placebo | 0 | 0 | 0 | 0 | 0 | 0 | 0 | 0 | 0.01 | 0.02 | 0.06 | 0.15 | 0.23 | 0.27 | 0.2 | 0.07 |
| Probiotics | 0 | 0 | 0 | 0.01 | 0.01 | 0.02 | 0.03 | 0.05 | 0.07 | 0.1 | 0.12 | 0.12 | 0.12 | 0.12 | 0.11 | 0.11 |
| Silver nitrate | 0.01 | 0.01 | 0.03 | 0.03 | 0.05 | 0.06 | 0.07 | 0.08 | 0.1 | 0.1 | 0.1 | 0.08 | 0.07 | 0.07 | 0.07 | 0.08 |
| Sucralfate | 0.47 | 0.18 | 0.1 | 0.06 | 0.05 | 0.03 | 0.02 | 0.02 | 0.02 | 0.01 | 0.01 | 0.01 | 0.01 | 0 | 0.01 | 0.01 |
| Triamcinolone | 0 | 0 | 0.02 | 0.05 | 0.13 | 0.21 | 0.23 | 0.17 | 0.1 | 0.05 | 0.02 | 0.01 | 0 | 0 | 0 | 0 |
| Zinc | 0.12 | 0.13 | 0.14 | 0.14 | 0.12 | 0.09 | 0.07 | 0.06 | 0.05 | 0.03 | 0.02 | 0.02 | 0.01 | 0.01 | 0.01 | 0.01 |

## 1.3 Consistency check

| Parameter | Median (95% CI) |
|---|---|
| Random Effects Standard Deviation | 26.72 (16.53, 48.07) |

## 1.4 Convergence Diagnostics

| Parameter | PSRF |
|---|---|
| d.Amlexanox.Dexamethasone | 1.00 |
| d.Chlorhexidine.Sucralfate | 1.00 |
| d.Placebo.Diosmectite | 1.00 |
| d.Placebo.Doxycycline | 1.00 |
| d.Placebo.Glycyrrhiza | 1.00 |
| d.Placebo.Insulinliposomalgel | 1.03 |
| d.Placebo.Probiotics | 1.00 |
| d.Placebo.Silvernitrate | 1.00 |
| d.Placebo.Zinc | 1.00 |
| d.Triamcinolone.Aloe | 1.00 |
| d.Triamcinolone.Amlexanox | 1.00 |
| d.Triamcinolone.Chlorhexidine | 1.00 |
| d.Triamcinolone.Curcumin | 1.00 |
| d.Triamcinolone.Laser | 1.00 |
| d.Triamcinolone.Placebo | 1.00 |
| sd.d | 1.00 |

Number of chains : 4
Tuning iterations : 20,000
Simulation iterations : 50,000
Thinning interval : 10
Inference samples : 10,000
Variance scaling factor: 2.5

## 2.Inconsistency Model

### 2.1 Summary estimates

| | | | | | | | |
|---|---|---|---|---|---|---|---|
| Aloe | -0.63 (-67.67, 65.06) | 66.97 (-17.22, 147.56) | 39.50 (-43.13, 120.18) | -4.82 (-98.01, 85.74) | 3.40 (-76.52, 84.82) | 30.21 (-35.25, 92.99) | 7.73 (-61.80, 74.76) |
| 0.63 (-65.06, 67.67) | Amlexanox | 67.86 (-8.74, 142.50) | 40.34 (-36.09, 111.87) | -4.18 (-65.17, 55.25) | 4.30 (-68.23, 76.45) | 31.08 (-27.05, 86.75) | 8.86 (-52.78, 67.51) |
| -66.97 (-147.56, 17.22) | -67.86 (-142.50, 8.74) | Chlorhexidine | -27.67 (-112.30, 60.21) | -71.94 (-169.01, 24.81) | -63.81 (-152.29, 30.14) | -36.49 (-116.24, 42.30) | -59.56 (-140.81, 23.44) |
| -39.50 (-120.18, 43.13) | -40.34 (-111.87, 36.09) | 27.67 (-60.21, 112.30) | Curcumin | -44.12 (-139.47, 53.31) | -35.67 (-124.35, 56.12) | -8.58 (-85.57, 66.74) | -31.44 (-110.59, 47.74) |
| 4.82 (-85.74, 98.01) | 4.18 (-55.25, 65.17) | 71.94 (-24.81, 169.01) | 44.12 (-53.31, 139.47) | Dexamethasone | 8.68 (-85.34, 105.12) | 35.31 (-46.23, 117.62) | 12.66 (-72.21, 99.27) |
| -3.40 (-84.82, 76.52) | -4.30 (-76.45, 68.23) | 63.81 (-30.14, 152.29) | 35.67 (-56.12, 124.35) | -8.68 (-105.12, 85.34) | Diosmectite | 26.30 (-41.97, 95.18) | 3.80 (-69.68, 74.13) |
| -30.21 (-92.99, 35.25) | -31.08 (-86.75, 27.05) | 36.49 (-42.30, 116.24) | 8.58 (-66.74, 85.57) | -35.31 (-117.62, 46.23) | -26.30 (-95.18, 41.97) | Doxycycline | -22.18 (-77.04, 30.93) |
| -7.73 (-74.76, 61.80) | -8.86 (-67.51, 52.78) | 59.56 (-23.44, 140.81) | 31.44 (-47.74, 110.59) | -12.66 (-99.27, 72.21) | -3.80 (-74.13, 69.68) | 22.18 (-30.93, 77.04) | Glycyrrhiza |
| -68.09 (-148.03, 14.06) | -69.15 (-140.56, 6.32) | -1.61 (-92.20, 90.99) | -29.01 (-116.69, 61.39) | -72.77 (-170.57, 23.04) | -64.40 (-146.90, 18.77) | -37.92 (-107.53, 31.35) | -60.24 (-131.93, 10.31) |
| -56.19 (-121.55, 5.93) | -57.62 (-111.06, -4.94) | 9.46 (-59.55, 81.48) | -18.16 (-86.50, 52.88) | -61.64 (-142.46, 18.88) | -52.76 (-126.30, 19.28) | -26.94 (-81.16, 26.86) | -49.26 (-107.56, 9.15) |
| 7.19 (-47.63, 62.64) | 6.30 (-37.77, 50.34) | 74.14 (2.95, 143.66) | 46.01 (-21.72, 112.86) | 1.80 (-76.17, 75.51) | 10.71 (-47.99, 69.36) | 37.11 (1.42, 70.89) | 14.57 (-27.09, 56.69) |
| -0.52 (-70.85, 68.74) | -1.21 (-61.07, 59.34) | 66.98 (-16.54, 148.52) | 38.15 (-39.03, 117.37) | -5.55 (-92.52, 79.78) | 3.16 (-68.52, 76.23) | 29.25 (-24.32, 84.27) | 6.94 (-52.20, 66.32) |
| -13.42 (-92.52, 64.99) | -14.46 (-85.06, 56.69) | 53.91 (-35.58, 141.59) | 25.24 (-62.55, 112.88) | -18.06 (-114.96, 75.32) | -9.63 (-92.23, 73.42) | 16.59 (-51.53, 83.04) | -6.01 (-79.02, 64.22) |
| -86.89 (-186.08, 19.65) | -86.45 (-181.35, 9.78) | -19.22 (-76.88, 37.90) | -47.01 (-150.14, 58.02) | -90.98 (-204.87, 23.22) | -82.53 (-187.00, 28.07) | -55.62 (-152.33, 43.26) | -78.25 (-179.68, 22.81) |
| -28.75 (-100.57, 45.93) | -38.35 (-83.15, 5.33) | 28.66 (-32.53, 89.86) | 1.36 (-58.07, 59.09) | -42.56 (-120.26, 31.70) | -34.38 (-101.87, 33.95) | -7.60 (-57.06, 40.60) | -30.15 (-84.06, 22.74) |
| -54.30 (-135.63, 26.92) | -55.02 (-130.82, 20.30) | 12.07 (-82.66, 103.71) | -15.38 (-106.34, 77.14) | -59.30 (-158.03, 36.77) | -50.59 (-135.09, 34.03) | -24.09 (-93.93, 44.29) | -46.86 (-120.18, 25.69) |

| | | | | | | | |
|---|---|---|---|---|---|---|---|
| 68.09 (-14.06, 148.03) | 56.19 (-5.93, 121.55) | -7.19 (-62.64, 47.63) | 0.52 (-68.74, 70.85) | 13.42 (-64.99, 92.52) | 86.89 (-19.65, 186.08) | 28.75 (-45.93, 100.57) | 54.30 (-26.92, 135.63) |
| 69.15 (-6.32, 140.56) | 57.62 (4.94, 111.06) | -6.30 (-50.34, 37.77) | 1.21 (-59.34, 61.07) | 14.46 (-56.69, 85.06) | 86.45 (-9.78, 181.35) | 38.35 (-5.33, 83.15) | 55.02 (-20.30, 130.82) |
| 1.61 (-90.99, 92.20) | -9.46 (-81.48, 59.55) | -74.14 (-143.66, -2.95) | -66.98 (-148.52, 16.54) | -53.91 (-141.59, 35.58) | 19.22 (-37.90, 76.88) | -28.66 (-89.86, 32.53) | -12.07 (-103.71, 82.66) |
| 29.01 (-61.39, 116.69) | 18.16 (-52.88, 86.50) | -46.01 (-112.86, 21.72) | -38.15 (-117.37, 39.03) | -25.24 (-112.88, 62.55) | 47.01 (-58.02, 150.14) | -1.36 (-59.09, 58.07) | 15.38 (-77.14, 106.34) |
| 72.77 (-23.04, 170.57) | 61.64 (-18.88, 142.46) | -1.80 (-75.51, 76.17) | 5.55 (-79.78, 92.52) | 18.06 (-75.32, 114.96) | 90.98 (-23.22, 204.87) | 42.56 (-31.70, 120.26) | 59.30 (-36.77, 158.03) |
| 64.40 (-18.77, 146.90) | 52.76 (-19.28, 126.30) | -10.71 (-69.36, 47.99) | -3.16 (-76.23, 68.52) | 9.63 (-73.42, 92.23) | 82.53 (-28.07, 187.00) | 34.38 (-33.95, 101.87) | 50.59 (-34.03, 135.09) |
| 37.92 (-31.35, 107.53) | 26.94 (-26.86, 81.16) | -37.11 (-70.89, -1.42) | -29.25 (-84.27, 24.32) | -16.59 (-83.04, 51.53) | 55.62 (-43.26, 152.33) | 7.60 (-40.60, 57.06) | 24.09 (-44.29, 93.93) |
| 60.24 (-10.31, 131.93) | 49.26 (-9.15, 107.56) | -14.57 (-56.69, 27.09) | -6.94 (-66.32, 52.20) | 6.01 (-64.22, 79.02) | 78.25 (-22.81, 179.68) | 30.15 (-22.74, 84.06) | 46.86 (-25.69, 120.18) |
| Insulin-liposomal gel | -11.34 (-81.86, 59.06) | -75.22 (-132.46, -15.52) | -66.84 (-137.71, 3.49) | -54.53 (-134.33, 28.68) | 18.39 (-91.13, 126.04) | -30.83 (-96.73, 39.62) | -13.35 (-97.20, 71.51) |
| 11.34 (-59.06, 81.86) | Laser | -61.25 (-93.02, -28.02) | -56.02 (-113.39, 1.52) | -43.14 (-114.11, 27.67) | 29.36 (-63.98, 120.81) | -19.26 (-54.50, 18.24) | -2.32 (-75.25, 71.60) |
| 75.22 (15.52, 132.46) | 61.25 (28.02, 93.02) | Placebo | 7.78 (-34.66, 48.39) | 20.41 (-36.01, 78.14) | 93.05 (-0.58, 182.72) | 51.67 (-5.55, 126.24) | 61.51 (1.09, 121.53) |
| 66.84 (-3.49, 137.71) | 56.02 (-1.52, 113.39) | -7.78 (-48.39, 34.66) | Probiotics | 12.95 (-58.15, 84.04) | 85.89 (-16.53, 184.04) | 36.89 (-14.58, 90.96) | 53.38 (-19.57, 126.79) |
| 54.53 (-28.68, 134.33) | 43.14 (-27.67, 114.11) | -20.41 (-78.14, 36.01) | -12.95 (-84.04, 58.15) | Silver nitrate | 72.50 (-33.83, 176.63) | 24.37 (-42.72, 89.91) | 40.73 (-42.03, 124.10) |
| -18.39 (-126.04, 91.13) | -29.36 (-120.81, 63.98) | -93.05 (-182.72, 0.58) | -85.89 (-184.04, 16.53) | -72.50 (-176.63, 33.83) | Sucralfate | -48.24 (-132.24, 39.58) | -31.83 (-136.29, 80.66) |
| 30.83 (-39.62, 96.73) | 19.26 (-18.24, 54.50) | -51.67 (-126.24, 5.55) | -36.89 (-90.96, 14.58) | -24.37 (-89.91, 42.72) | 48.24 (-39.58, 132.24) | Triamcinolone | 16.64 (-52.41, 85.48) |
| 13.35 (-71.51, 97.20) | 2.32 (-71.60, 75.25) | -61.51 (-121.53, -1.09) | -53.38 (-126.79, 19.57) | -40.73 (-124.10, 42.03) | 31.83 (-80.66, 136.29) | -16.64 (-85.48, 52.41) | Zinc |

2.2 Inconsistency Factors

| Cycle | Median (95% CI) |
|---|---|
| Amlexanox, Laser, Placebo, Triamcinolone | -1.00 (-44.82, 37.53) |
| Amlexanox, Placebo, Triamcinolone | 5.22 (-40.96, 72.30) |
| Amlexanox, Placebo, Triamcinolone | 5.22 (-40.96, 72.30) |

2.3 Variance Calculation

| Parameter | Median (95% CI) |
|---|---|
| Random Effects Standard Deviation | 25.89 (15.63, 48.04) |
| Inconsistency Standard Deviation | 22.79 (0.64, 71.08) |

## 2.4 Convergence Diagnostics

| Parameter | PSRF |
|---|---|
| d.Aloe.Placebo | 1.00 |
| d.Amlexanox.Dexamethasone | 1.00 |
| d.Amlexanox.Triamcinolone | 1.00 |
| d.Chlorhexidine.Sucralfate | 1.00 |
| d.Placebo.Amlexanox | 1.00 |
| d.Placebo.Diosmectite | 1.00 |
| d.Placebo.Doxycycline | 1.00 |
| d.Placebo.Glycyrrhiza | 1.00 |
| d.Placebo.Insulinliposomalgel | 1.00 |
| d.Placebo.Probiotics | 1.00 |
| d.Placebo.Silvernitrate | 1.00 |
| d.Placebo.Zinc | 1.00 |
| d.Triamcinolone.Chlorhexidine | 1.00 |
| d.Triamcinolone.Curcumin | 1.00 |
| d.Triamcinolone.Laser | 1.00 |
| w.Amlexanox.Placebo.Laser.Tria... | 1.00 |
| w.Amlexanox.Placebo.Triamcinol... | 1.00 |
| w.Amlexanox.Placebo.Triamcinol... | 1.00 |
| sd.d | 1.00 |
| sd.w | 1.00 |

Number of chains : 4
Tuning iterations : 20,000
Simulation iterations : 50,000
Thinning interval : 10
Inference samples : 10,000
Variance scaling factor: 2.5

## 3. Node-splitting analysis

| Name | Direct Effect | Indirect Effect | Overall | P-Value |
|---|---|---|---|---|
| Amlexanox, Placebo | -5.71 (-70.72, 60.86) | -5.20 (-79.45, 69.55) | -4.96 (-50.43, 37.96) | 1 |
| Amlexanox, Triamcinolone | 37.74 (-28.49, 101.58) | 37.23 (-37.02, 111.53) | 37.42 (-6.48, 82.40) | 0.99 |
| Laser, Placebo | -59.48 (-100.64, -19.03) | -63.88 (-128.46, -1.40) | -61.05 (-92.61, -29.60) | 0.9 |
| Laser, Triamcinolone | -20.04 (-66.88, 27.58) | -15.76 (-73.03, 42.86) | -18.51 (-50.02, 15.44) | 0.9 |
| Placebo, Triamcinolone | 43.93 (-1.45, 90.98) | 40.57 (-9.38, 91.52) | 42.65 (12.28, 74.68) | 0.93 |

## 4. Network structure

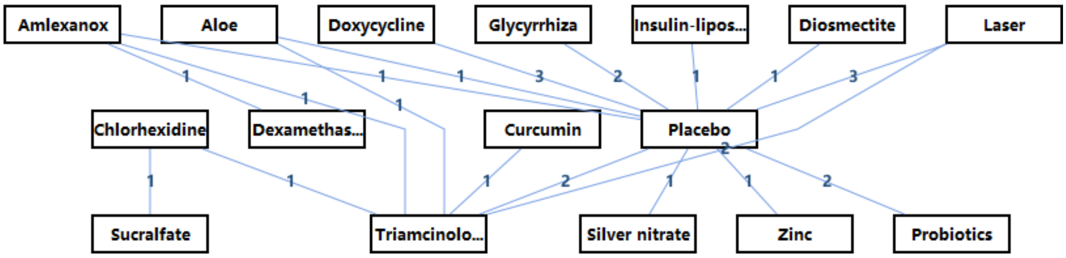

## Day 4

# 1. Consistency Model

## 1.1 Summary estimates

| | | | | | | | | | | |
|---|---|---|---|---|---|---|---|---|---|---|
| Allicin | -21.91 (-64.39, 21.05) | 2.67 (-33.50, 38.84) | -11.32 (-56.24, 33.22) | -0.63 (-46.97, 45.48) | -13.03 (-65.68, 39.89) | 5.56 (-45.85, 58.64) | -3.44 (-46.21, 41.16) | -4.90 (-50.44, 42.18) | 8.71 (-27.46, 45.95) | -29.43 (-77.43, 18.97) |
| 21.91 (-21.05, 64.39) | Aloe | 24.59 (-6.92, 57.22) | 10.56 (-28.69, 50.20) | 21.07 (-21.44, 63.86) | 8.96 (-39.61, 59.14) | 27.57 (-18.55, 75.49) | 18.70 (-18.25, 55.75) | 17.55 (-26.59, 61.14) | 30.93 (-2.94, 63.19) | -7.57 (-51.79, 37.65) |
| -2.67 (-38.84, 33.50) | -24.59 (-57.22, 6.92) | Amlexanox | -14.13 (-47.84, 19.93) | -3.69 (-38.63, 32.46) | -15.71 (-58.36, 28.48) | 3.08 (-40.60, 47.36) | -6.14 (-39.10, 27.24) | -7.31 (-43.76, 29.78) | 6.05 (-17.93, 31.09) | -32.45 (-70.10, 6.20) |
| 11.32 (-33.22, 56.24) | -10.56 (-50.20, 28.69) | 14.13 (-19.93, 47.84) | Benzydamine | 10.62 (-32.91, 55.13) | -1.23 (-52.67, 49.07) | 16.89 (-29.84, 66.66) | 7.83 (-30.63, 47.06) | 6.66 (-38.05, 51.60) | 20.15 (-11.58, 51.53) | -18.19 (-64.25, 28.09) |
| 0.63 (-45.48, 46.97) | -21.07 (-63.86, 21.44) | 3.69 (-32.46, 38.63) | -10.62 (-55.13, 32.91) | Berberine gelatin | -11.99 (-62.17, 40.30) | 6.45 (-46.37, 60.05) | -2.83 (-45.20, 40.91) | -3.74 (-50.45, 41.33) | 9.85 (-28.21, 46.99) | -28.66 (-76.29, 18.62) |
| 13.03 (-39.89, 65.68) | -8.96 (-59.14, 39.61) | 15.71 (-28.48, 58.36) | 1.23 (-49.07, 52.67) | 11.99 (-40.30, 62.17) | Chitosan | 18.69 (-38.67, 77.00) | 9.33 (-40.60, 59.29) | 8.39 (-44.65, 60.76) | 21.78 (-24.08, 67.12) | -16.83 (-71.68, 36.52) |
| -5.56 (-58.64, 45.85) | -27.57 (-75.49, 18.55) | -3.08 (-47.36, 40.60) | -16.89 (-66.66, 29.84) | -6.45 (-60.05, 46.37) | -18.69 (-77.00, 38.67) | Chlorhexidine | -9.34 (-55.05, 35.30) | -9.80 (-64.85, 41.30) | 3.23 (-42.28, 46.57) | -34.92 (-89.92, 17.73) |
| 3.44 (-41.16, 46.21) | -18.70 (-55.75, 18.25) | 6.14 (-27.24, 39.10) | -7.83 (-47.06, 30.63) | 2.83 (-40.91, 45.20) | -9.33 (-59.29, 40.60) | 9.34 (-35.30, 55.05) | Curcumin | -0.78 (-46.11, 42.12) | 12.42 (-21.04, 44.95) | -25.95 (-72.22, 18.54) |
| 4.90 (-42.18, 50.44) | -17.55 (-61.14, 26.59) | 7.31 (-29.78, 43.76) | -6.66 (-51.60, 38.05) | 3.74 (-41.33, 50.45) | -8.39 (-60.76, 44.65) | 9.80 (-41.30, 64.85) | 0.78 (-42.12, 46.11) | Diosmectite | 13.28 (-25.37, 51.55) | -25.08 (-73.70, 22.82) |
| -8.71 (-45.95, 27.46) | -30.93 (-63.19, 2.94) | -6.05 (-31.09, 17.93) | -20.15 (-51.53, 11.58) | -9.85 (-46.99, 28.21) | -21.78 (-67.12, 24.08) | -3.23 (-46.57, 42.28) | -12.42 (-44.95, 21.04) | -13.28 (-51.55, 25.37) | Doxycycline | -38.74 (-77.82, 1.67) |
| 29.43 (-18.97, 77.43) | 7.57 (-37.65, 51.79) | 32.45 (-6.20, 70.10) | 18.19 (-28.09, 64.25) | 28.66 (-18.62, 76.29) | 16.83 (-36.52, 71.68) | 34.92 (-17.73, 89.92) | 25.95 (-18.54, 72.22) | 25.08 (-22.82, 73.70) | 38.74 (-1.67, 77.82) | Glycyrrhiza |
| -13.09 (-60.69, 34.52) | -35.23 (-80.14, 10.16) | -10.84 (-47.51, 27.20) | -24.47 (-70.01, 20.60) | -13.90 (-59.56, 32.08) | -26.05 (-78.39, 27.00) | -7.75 (-60.59, 46.08) | -16.96 (-61.16, 27.38) | -17.87 (-65.52, 30.10) | -4.34 (-43.37, 34.69) | -42.86 (-90.94, 6.70) |
| -40.64 (-77.33, -3.55) | -63.17 (-94.25, -30.52) | -38.30 (-62.16, -14.39) | -52.51 (-86.33, -18.71) | -41.65 (-78.72, -4.50) | -54.09 (-97.44, -8.87) | -35.02 (-77.87, 8.95) | -44.31 (-76.15, -12.45) | -45.42 (-83.03, -7.56) | -32.03 (-58.17, -6.34) | -70.48 (-109.65, -32.13) |
| -21.33 (-85.93, 43.61) | -43.12 (-106.37, 18.38) | -18.46 (-78.02, 40.84) | -32.40 (-94.97, 29.36) | -22.25 (-88.47, 43.28) | -33.82 (-105.18, 35.68) | -15.39 (-55.18, 24.86) | -24.59 (-84.86, 34.91) | -25.25 (-92.68, 39.69) | -12.17 (-72.83, 46.50) | -50.78 (-117.62, 15.63) |
| 22.84 (-9.96, 55.31) | 0.89 (-27.33, 28.22) | 25.47 (9.44, 41.54) | 11.22 (-18.37, 41.40) | 21.88 (-9.92, 54.54) | 9.82 (-30.90, 50.48) | 28.64 (-12.32, 70.15) | 19.33 (-9.52, 48.42) | 18.29 (-15.43, 50.91) | 31.78 (12.33, 50.54) | -6.94 (-41.97, 27.14) |
| -6.72 (-66.09, 51.72) | -29.06 (-84.58, 25.10) | -4.29 (-56.05, 46.44) | -18.32 (-56.56, 19.80) | -8.00 (-66.79, 50.64) | -20.19 (-84.19, 44.82) | -1.17 (-63.71, 61.25) | -10.46 (-64.44, 43.95) | -11.62 (-70.12, 46.43) | 1.73 (-47.93, 51.10) | -36.57 (-95.38, 22.15) |
| 4.93 (-32.43, 40.86) | -17.32 (-46.41, 10.77) | 7.51 (-16.21, 30.10) | -6.55 (-36.73, 23.90) | 4.02 (-32.53, 40.47) | -8.29 (-51.93, 36.21) | 10.72 (-26.37, 48.76) | 1.54 (-22.95, 25.03) | 0.35 (-37.15, 36.55) | 13.66 (-9.54, 36.56) | -24.70 (-63.31, 13.07) |
| 1.41 (-41.86, 44.83) | -20.40 (-59.58, 17.43) | 4.51 (-29.26, 37.01) | -9.59 (-50.08, 30.69) | 0.85 (-41.75, 43.97) | -11.10 (-60.17, 38.39) | 7.40 (-39.51, 55.54) | -1.93 (-39.32, 36.16) | -2.98 (-47.52, 40.75) | 10.51 (-23.78, 43.37) | -28.10 (-72.01, 17.05) |
| -41.74 (-89.11, 5.29) | -63.52 (-107.61, -19.79) | -39.01 (-76.71, -1.31) | -53.08 (-98.66, -8.12) | -42.57 (-88.81, 4.15) | -55.15 (-106.93, -0.50) | -36.14 (-88.05, 17.05) | -45.14 (-90.19, -0.19) | -46.43 (-94.18, 1.77) | -32.89 (-72.49, 5.56) | -71.57 (-119.24, -21.15) |

| | | | | | | | |
|---|---|---|---|---|---|---|---|
| 13.09 (-34.52, 60.69) | 40.64 (3.55, 77.33) | 21.33 (-43.61, 85.93) | -22.84 (-55.31, 9.96) | 6.72 (-51.72, 66.09) | -4.93 (-40.86, 32.43) | -1.41 (-44.83, 41.86) | 41.74 (-5.29, 89.11) |
| 35.23 (-10.16, 80.14) | 63.17 (30.52, 94.25) | 43.12 (-18.38, 106.37) | -0.89 (-28.22, 27.33) | 29.06 (-25.10, 84.58) | 17.32 (-10.77, 46.41) | 20.40 (-17.43, 59.58) | 63.52 (19.79, 107.61) |
| 10.84 (-27.20, 47.51) | 38.30 (14.39, 62.16) | 18.46 (-40.84, 78.02) | -25.47 (-41.54, -9.44) | 4.29 (-46.44, 56.05) | -7.51 (-30.10, 16.21) | -4.51 (-37.01, 29.26) | 39.01 (1.31, 76.71) |
| 24.47 (-20.60, 70.01) | 52.51 (18.71, 86.33) | 32.40 (-29.36, 94.97) | -11.22 (-41.40, 18.37) | 18.32 (-19.80, 56.56) | 6.55 (-23.90, 36.73) | 9.59 (-30.69, 50.08) | 53.08 (8.12, 98.66) |
| 13.90 (-32.08, 59.56) | 41.65 (4.50, 78.72) | 22.25 (-43.28, 88.47) | -21.88 (-54.54, 9.92) | 8.00 (-50.64, 66.79) | -4.02 (-40.47, 32.53) | -0.85 (-43.97, 41.75) | 42.57 (-4.15, 88.81) |
| 26.05 (-27.00, 78.39) | 54.09 (8.87, 97.44) | 33.82 (-35.68, 105.18) | -9.82 (-50.48, 30.90) | 20.19 (-44.82, 84.19) | 8.29 (-36.21, 51.93) | 11.10 (-38.39, 60.17) | 55.15 (0.50, 106.93) |
| 7.75 (-46.08, 60.59) | 35.02 (-8.95, 77.87) | 15.39 (-24.86, 55.18) | -28.64 (-70.15, 12.32) | 1.17 (-61.25, 63.71) | -10.72 (-48.76, 26.37) | -7.40 (-55.54, 39.51) | 36.14 (-17.05, 88.05) |
| 16.96 (-27.38, 61.16) | 44.31 (12.45, 76.15) | 24.59 (-34.91, 84.86) | -19.33 (-48.42, 9.52) | 10.46 (-43.95, 64.44) | -1.54 (-25.03, 22.95) | 1.93 (-36.16, 39.32) | 45.14 (0.19, 90.19) |
| 17.87 (-30.10, 65.52) | 45.42 (7.56, 83.03) | 25.25 (-39.69, 92.68) | -18.29 (-50.91, 15.43) | 11.62 (-46.43, 70.12) | -0.35 (-36.55, 37.15) | 2.98 (-40.75, 47.52) | 46.43 (-1.77, 94.18) |
| 4.34 (-34.69, 43.37) | 32.03 (6.34, 58.17) | 12.17 (-46.50, 72.83) | -31.78 (-50.54, -12.33) | -1.73 (-51.10, 47.93) | -13.66 (-36.56, 9.54) | -10.51 (-43.37, 23.78) | 32.89 (-5.56, 72.49) |
| 42.86 (-6.70, 90.94) | 70.48 (32.13, 109.65) | 50.78 (-15.63, 117.62) | 6.94 (-27.14, 41.97) | 36.57 (-22.15, 95.38) | 24.70 (-13.07, 63.31) | 28.10 (-17.05, 72.01) | 71.57 (21.15, 119.24) |
| Insulin-liposomal gel | 27.69 (-10.27, 65.70) | 8.00 (-58.30, 74.17) | -36.15 (-69.20, -1.47) | -6.09 (-65.20, 53.38) | -18.17 (-54.54, 20.47) | -14.82 (-58.67, 30.10) | 28.81 (-19.86, 75.89) |
| -27.69 (-65.70, 10.27) | Laser | -19.85 (-78.34, 38.84) | -63.68 (-81.37, -45.97) | -33.93 (-84.04, 17.12) | -45.82 (-66.42, -24.87) | -42.67 (-74.78, -10.63) | 1.12 (-37.79, 39.08) |
| -8.00 (-74.17, 58.30) | 19.85 (-38.84, 78.34) | N-acetylcysteine | -43.60 (-101.04, 13.45) | -14.16 (-88.53, 58.87) | -26.28 (-81.03, 28.45) | -22.65 (-83.93, 38.51) | 20.43 (-46.14, 85.44) |
| 36.15 (1.47, 69.20) | 63.68 (45.97, 81.37) | 43.60 (-13.45, 101.04) | Placebo | 29.69 (-18.27, 77.88) | 17.85 (1.80, 34.39) | 21.04 (-7.20, 49.83) | 64.55 (30.62, 98.58) |
| 6.09 (-53.38, 65.20) | 33.93 (-17.12, 84.04) | 14.16 (-58.87, 88.53) | -29.69 (-77.88, 18.27) | Quercetin | -11.67 (-60.27, 37.36) | -8.73 (-64.65, 46.73) | 34.87 (-25.26, 94.47) |
| 18.17 (-20.47, 54.54) | 45.82 (24.87, 66.42) | 26.28 (-28.45, 81.03) | -17.85 (-34.39, -1.80) | 11.67 (-37.36, 60.27) | Triamcinolone | 3.11 (-25.44, 31.10) | 46.71 (8.08, 83.72) |
| 14.82 (-30.10, 58.67) | 42.67 (10.63, 74.78) | 22.65 (-38.51, 83.93) | -21.04 (-49.83, 7.20) | 8.73 (-46.73, 64.65) | -3.11 (-31.10, 25.44) | Triester Glycerol Oxide | 43.35 (-1.15, 87.07) |
| -28.81 (-75.89, 19.86) | -1.12 (-39.08, 37.79) | -20.43 (-85.44, 46.14) | -64.55 (-98.58, -30.62) | -34.87 (-94.47, 25.26) | -46.71 (-83.72, -8.08) | -43.35 (-87.07, 1.15) | Zinc |

## 1.2 Rank probability(Rank 1 is best, rank N is worst)

| Drug | Rank 1 | Rank 2 | Rank 3 | Rank 4 | Rank 5 | Rank 6 | Rank 7 | Rank 8 | Rank 9 | Rank 10 | Rank 11 | Rank 12 | Rank 13 | Rank 14 | Rank 15 | Rank 16 | Rank 17 | Rank 18 | Rank 19 |
|---|---|---|---|---|---|---|---|---|---|---|---|---|---|---|---|---|---|---|---|
| Allicin | 0 | 0.01 | 0.04 | 0.06 | 0.07 | 0.07 | 0.08 | 0.07 | 0.07 | 0.07 | 0.07 | 0.06 | 0.06 | 0.06 | 0.06 | 0.05 | 0.03 | 0.03 | 0.02 |
| Aloe | 0 | 0 | 0 | 0 | 0 | 0 | 0.01 | 0.01 | 0.01 | 0.02 | 0.03 | 0.04 | 0.05 | 0.08 | 0.1 | 0.13 | 0.15 | 0.19 | 0.17 |
| Amlexanox | 0 | 0 | 0.01 | 0.03 | 0.07 | 0.1 | 0.12 | 0.13 | 0.13 | 0.12 | 0.09 | 0.07 | 0.05 | 0.04 | 0.02 | 0.01 | 0 | 0 | 0 |
| Benzydamine | 0 | 0 | 0 | 0.01 | 0.02 | 0.02 | 0.03 | 0.05 | 0.05 | 0.06 | 0.06 | 0.08 | 0.09 | 0.11 | 0.11 | 0.1 | 0.09 | 0.08 | 0.05 |
| Berberine gelatin | 0 | 0.01 | 0.03 | 0.05 | 0.06 | 0.07 | 0.08 | 0.07 | 0.07 | 0.07 | 0.07 | 0.07 | 0.07 | 0.07 | 0.06 | 0.05 | 0.03 | 0.03 | 0.02 |
| Chitosan | 0 | 0.01 | 0.02 | 0.03 | 0.03 | 0.04 | 0.04 | 0.04 | 0.04 | 0.04 | 0.05 | 0.05 | 0.05 | 0.07 | 0.08 | 0.08 | 0.08 | 0.11 | 0.14 |
| Chlorhexidine | 0.01 | 0.03 | 0.06 | 0.12 | 0.11 | 0.09 | 0.07 | 0.07 | 0.06 | 0.06 | 0.05 | 0.05 | 0.05 | 0.05 | 0.04 | 0.03 | 0.03 | 0.03 | 0.01 |
| Curcumin | 0 | 0 | 0.02 | 0.03 | 0.04 | 0.06 | 0.07 | 0.08 | 0.08 | 0.08 | 0.09 | 0.09 | 0.09 | 0.08 | 0.07 | 0.05 | 0.03 | 0.02 | 0.02 |
| Diosmectite | 0 | 0.01 | 0.02 | 0.04 | 0.05 | 0.06 | 0.06 | 0.07 | 0.07 | 0.06 | 0.07 | 0.07 | 0.07 | 0.08 | 0.08 | 0.06 | 0.05 | 0.05 | 0.03 |
| Doxycycline | 0 | 0.01 | 0.04 | 0.1 | 0.15 | 0.16 | 0.14 | 0.11 | 0.09 | 0.07 | 0.05 | 0.03 | 0.02 | 0.01 | 0.01 | 0 | 0 | 0 | 0 |
| Glycyrrhiza | 0 | 0 | 0 | 0 | 0 | 0.01 | 0.01 | 0.01 | 0.01 | 0.02 | 0.02 | 0.03 | 0.03 | 0.04 | 0.06 | 0.07 | 0.1 | 0.17 | 0.42 |
| Insulin-liposomal | 0.02 | 0.05 | 0.14 | 0.15 | 0.13 | 0.09 | 0.08 | 0.07 | 0.05 | 0.04 | 0.04 | 0.03 | 0.03 | 0.02 | 0.02 | 0.01 | 0.01 | 0.01 | 0 |

| | | | | | | | | | | | | | | | | | | | |
|---|---|---|---|---|---|---|---|---|---|---|---|---|---|---|---|---|---|---|---|
| Laser | 0.32 | 0.42 | 0.17 | 0.06 | 0.02 | 0.01 | 0 | 0 | 0 | 0 | 0 | 0 | 0 | 0 | 0 | 0 | 0 | 0 | 0 |
| N-acetylcysteine | 0.16 | 0.12 | 0.16 | 0.11 | 0.07 | 0.05 | 0.04 | 0.04 | 0.03 | 0.03 | 0.03 | 0.03 | 0.02 | 0.02 | 0.02 | 0.02 | 0.02 | 0.02 | 0.02 |
| Placebo | 0 | 0 | 0 | 0 | 0 | 0 | 0 | 0 | 0 | 0 | 0 | 0.01 | 0.02 | 0.05 | 0.12 | 0.22 | 0.31 | 0.22 | 0.06 |
| Quercetin | 0.04 | 0.06 | 0.1 | 0.1 | 0.08 | 0.07 | 0.06 | 0.05 | 0.05 | 0.05 | 0.04 | 0.04 | 0.04 | 0.04 | 0.04 | 0.03 | 0.03 | 0.03 | 0.03 |
| Triamcinolone | 0 | 0 | 0 | 0 | 0.01 | 0.01 | 0.03 | 0.05 | 0.09 | 0.13 | 0.16 | 0.17 | 0.15 | 0.1 | 0.06 | 0.02 | 0.01 | 0 | 0 |
| TGO | 0 | 0.01 | 0.02 | 0.04 | 0.05 | 0.07 | 0.08 | 0.08 | 0.08 | 0.08 | 0.09 | 0.08 | 0.09 | 0.07 | 0.06 | 0.04 | 0.02 | 0.02 | 0.01 |
| Zinc | 0.42 | 0.27 | 0.15 | 0.07 | 0.04 | 0.02 | 0.01 | 0.01 | 0.01 | 0 | 0 | 0 | 0 | 0 | 0 | 0 | 0 | 0 | 0 |

## 1.3 Consistency check

| Parameter | Median (95% CI) |
|---|---|
| Random Effects Standard Deviation | 14.34 (9.10, 25.48) |

## 1.4 Convergence Diagnostics

| Parameter | PSRF |
|---|---|
| d.Benzydamine.Quercetin | 1.00 |
| d.Chlorhexidine.Nacetylcysteine | 1.00 |
| d.Placebo.Allicin | 1.00 |
| d.Placebo.Amlexanox | 1.00 |
| d.Placebo.Berberinegelatin | 1.00 |
| d.Placebo.Chitosan | 1.00 |
| d.Placebo.Diosmectite | 1.00 |
| d.Placebo.Glycyrrhiza | 1.00 |
| d.Placebo.Insulinliposomalgel | 1.15 |
| d.Placebo.Zinc | 1.00 |
| d.Triamcinolone.Aloe | 1.00 |
| d.Triamcinolone.Benzydamine | 1.00 |
| d.Triamcinolone.Chlorhexidine | 1.00 |
| d.Triamcinolone.Curcumin | 1.00 |
| d.Triamcinolone.Doxycycline | 1.00 |
| d.Triamcinolone.Laser | 1.00 |
| d.Triamcinolone.Placebo | 1.00 |
| d.Triamcinolone.TriesterGlycerol... | 1.00 |
| sd.d | 1.00 |

Number of chains : 4
Tuning iterations : 20,000
Simulation iterations : 50,000
Thinning interval : 10
Inference samples : 10,000
Variance scaling factor: 2.5

# 2.Inconsistency Model

## 2.1 Summary estimates

| | | | |
|---|---|---|---|
| Allicin | -18.78 (-65.21, 26.69) | 2.95 (-35.40, 40.16) | -9.16 (-55.76, 37.33) |
| 18.78 (-26.69, 65.21) | Aloe | 21.25 (-13.20, 56.35) | 9.63 (-32.58, 50.71) |
| -2.95 (-40.16, 35.40) | -21.25 (-56.35, 13.20) | Amlexanox | -12.14 (-48.43, 24.38) |
| 9.16 (-37.33, 55.76) | -9.63 (-50.71, 32.58) | 12.14 (-24.38, 48.43) | Benzydamine |
| 0.72 (-45.93, 48.71) | -17.81 (-62.99, 26.57) | 3.43 (-33.40, 40.18) | -8.38 (-55.11, 36.65) |
| 12.57 (-40.65, 66.41) | -6.27 (-56.75, 45.40) | 15.15 (-28.18, 59.58) | 3.27 (-47.76, 53.80) |
| -6.41 (-59.41, 49.52) | -25.15 (-72.45, 24.04) | -3.65 (-48.92, 42.11) | -15.48 (-66.16, 36.37) |
| 2.58 (-44.47, 48.98) | -16.31 (-55.22, 22.78) | 5.21 (-30.24, 40.89) | -6.89 (-47.79, 34.27) |
| 4.17 (-44.26, 52.80) | -14.53 (-60.83, 30.65) | 6.92 (-32.08, 44.26) | -4.88 (-52.04, 40.44) |
| -9.62 (-48.04, 30.78) | -28.20 (-62.59, 6.77) | -6.78 (-32.30, 19.09) | -18.73 (-51.87, 13.62) |
| 29.41 (-20.34, 79.17) | 10.89 (-35.88, 57.07) | 32.22 (-6.22, 71.81) | 20.64 (-28.03, 66.86) |
| -14.46 (-61.62, 34.91) | -32.99 (-79.02, 12.30) | -11.71 (-49.03, 25.86) | -23.57 (-69.96, 23.00) |
| -41.57 (-84.60, 1.83) | -60.40 (-97.69, -21.30) | -38.86 (-70.17, -7.41) | -50.45 (-90.57, -10.36) |
| -22.16 (-89.02, 45.73) | -40.71 (-103.56, 21.16) | -19.54 (-81.09, 40.52) | -31.75 (-95.96, 33.91) |
| 22.76 (-11.68, 57.37) | 3.97 (-26.84, 34.22) | 25.46 (8.72, 41.99) | 13.61 (-18.83, 45.00) |
| -9.27 (-69.85, 51.86) | -27.72 (-85.93, 30.32) | -6.05 (-61.02, 46.51) | -18.36 (-57.79, 20.98) |
| 3.83 (-35.14, 42.71) | -16.90 (-68.65, 38.60) | 6.73 (-17.68, 30.91) | -5.08 (-38.03, 26.88) |
| -1.72 (-47.34, 43.93) | -20.49 (-59.97, 20.08) | 1.09 (-33.65, 35.95) | -10.72 (-53.09, 31.13) |
| -41.38 (-91.44, 7.63) | -60.14 (-107.37, -14.12) | -38.66 (-79.13, -0.21) | -50.72 (-98.04, -3.96) |

| | | | | | | | | | | | | |
|---|---|---|---|---|---|---|---|---|---|---|---|---|
| -0.72 (-48.71, 45.93) | -12.57 (-66.41, 40.65) | 6.41 (-49.52, 59.41) | -2.58 (-48.98, 44.47) | -4.17 (-52.80, 44.26) | 9.62 (-30.78, 48.04) | -29.41 (-79.17, 20.34) | 14.46 (-34.91, 61.62) | 41.57 (-1.83, 84.60) | 22.16 (-45.73, 89.02) | -22.76 (-57.37, 11.68) | 9.27 (-51.86, 69.85) | -3.83 (-42.71, 35.14) |
| 17.81 (-26.57, 62.99) | 6.27 (-45.40, 56.75) | 25.15 (-24.04, 72.45) | 16.31 (-22.78, 55.22) | 14.53 (-30.65, 60.83) | 28.20 (-6.77, 62.59) | -10.89 (-57.07, 35.88) | 32.99 (-12.30, 79.02) | 60.40 (21.30, 97.69) | 40.71 (-21.16, 103.56) | -3.97 (-34.22, 26.84) | 27.72 (-30.32, 85.93) | 16.90 (-38.60, 68.65) |
| -3.43 (-40.18, 33.40) | -15.15 (-59.58, 28.18) | 3.65 (-42.11, 48.92) | -5.21 (-40.89, 30.24) | -6.92 (-44.26, 32.08) | 6.78 (-19.09, 32.30) | -32.22 (-71.81, 6.22) | 11.71 (-25.86, 49.03) | 38.86 (7.41, 70.17) | 19.54 (-40.52, 81.09) | -25.46 (-41.99, -8.72) | 6.05 (-46.51, 61.02) | -6.73 (-30.91, 17.68) |
| 8.38 (-36.65, 55.11) | -3.27 (-53.80, 47.76) | 15.48 (-36.37, 66.16) | 6.89 (-34.27, 47.79) | 4.88 (-40.44, 52.04) | 18.73 (-13.62, 51.87) | -20.64 (-66.86, 28.03) | 23.57 (-23.00, 69.96) | 50.45 (10.36, 90.57) | 31.75 (-33.91, 95.96) | -13.61 (-45.00, 18.83) | 18.36 (-20.98, 57.79) | 5.08 (-26.88, 38.03) |
| Berberine gelatin | -11.66 (-64.28, 40.48) | 7.23 (-46.80, 60.00) | -1.59 (-47.52, 43.14) | -3.38 (-50.05, 43.86) | 10.51 (-28.47, 47.58) | -28.68 (-77.02, 19.66) | 15.53 (-32.13, 61.06) | 42.20 (0.57, 84.64) | 22.84 (-44.08, 90.71) | -21.86 (-54.39, 10.44) | 9.79 (-49.71, 70.99) | -3.33 (-39.97, 33.91) |
| 11.66 (-40.48, 64.28) | Chitosan | 18.89 (-41.23, 77.45) | 10.19 (-40.76, 60.18) | 8.33 (-44.28, 60.85) | 22.13 (-22.08, 66.53) | -17.10 (-71.74, 36.68) | 26.88 (-24.02, 80.19) | 53.90 (5.59, 101.93) | 34.71 (-37.43, 107.40) | -10.21 (-49.40, 31.04) | 21.59 (-43.26, 86.73) | 8.52 (-35.55, 52.63) |
| -7.23 (-60.00, 46.80) | -18.89 (-77.45, 41.23) | Chlorhexidine | -8.88 (-54.69, 38.01) | -10.63 (-63.88, 44.87) | 2.79 (-41.72, 49.92) | -35.77 (-90.50, 19.83) | 7.90 (-45.58, 62.18) | 35.20 (-12.10, 81.24) | 15.95 (-24.72, 56.45) | -29.33 (-70.81, 13.95) | 2.72 (-60.08, 66.99) | -10.21 (-47.88, 29.56) |
| 1.59 (-43.14, 47.52) | -10.19 (-60.18, 40.76) | 8.88 (-38.01, 54.69) | Curcumin | -2.06 (-47.03, 44.56) | 12.03 (-23.28, 46.83) | -27.23 (-74.62, 20.50) | 16.92 (-28.25, 62.71) | 43.77 (8.58, 80.13) | 24.88 (-36.31, 86.79) | -20.30 (-50.81, 11.29) | 11.52 (-45.11, 67.67) | -1.67 (-26.54, 23.57) |
| 3.38 (-43.86, 50.05) | -8.33 (-60.85, 44.28) | 10.63 (-44.87, 63.88) | 2.06 (-44.56, 47.03) | Diosmectite | 13.86 (-26.32, 52.45) | -25.40 (-74.10, 22.90) | 18.84 (-29.95, 65.12) | 45.54 (2.93, 88.33) | 26.81 (-41.49, 92.99) | -18.46 (-52.80, 15.38) | 13.39 (-47.39, 72.76) | 0.30 (-38.71, 38.67) |
| -10.51 (-47.58, 28.47) | -22.13 (-66.53, 22.08) | -2.79 (-49.92, 41.72) | -12.03 (-46.83, 23.28) | -13.86 (-52.45, 26.32) | Doxycycline | -39.18 (-78.49, 1.16) | 5.00 (-34.35, 44.31) | 31.88 (0.43, 64.11) | 12.83 (-47.43, 74.05) | -32.39 (-51.35, -12.47) | -0.29 (-50.48, 50.52) | -13.51 (-37.09, 11.36) |
| 28.68 (-19.66, 77.02) | 17.10 (-36.68, 71.74) | 35.77 (-19.83, 90.50) | 27.23 (-20.50, 74.62) | 25.40 (-22.90, 74.10) | 39.18 (-1.16, 78.49) | Glycyrrhiza | 43.66 (-4.16, 91.84) | 70.96 (27.05, 115.51) | 51.63 (-17.37, 120.45) | 6.74 (-27.23, 41.96) | 38.63 (-22.05, 100.18) | 25.62 (-12.61, 65.95) |
| -15.53 (-61.06, 32.13) | -26.88 (-80.19, 24.02) | -7.90 (-62.18, 45.58) | -16.92 (-62.71, 28.25) | -18.84 (-65.12, 29.95) | -5.00 (-44.31, 34.35) | -43.66 (-91.84, 4.16) | Insulin-liposomal gel | 26.77 (-15.79, 70.98) | 7.66 (-59.79, 75.48) | -37.40 (-70.22, -3.12) | -5.15 (-65.78, 56.75) | -18.52 (-56.32, 20.38) |
| -42.20 (-84.64, -0.57) | -53.90 (-101.93, -5.59) | -35.20 (-81.24, 12.10) | -43.77 (-80.13, -8.58) | -45.54 (-88.33, -2.93) | -31.88 (-64.11, -0.43) | -70.96 (-115.51, -27.05) | -26.77 (-70.98, 15.79) | Laser | -19.17 (-80.25, 42.48) | -63.64 (-82.60, -44.84) | -32.53 (-87.20, 23.80) | -45.42 (-71.49, -18.92) |
| -22.84 (-90.71, 44.08) | -34.71 (-107.40, 37.43) | -15.95 (-56.45, 24.72) | -24.88 (-86.79, 36.31) | -26.81 (-92.99, 41.49) | -12.83 (-74.05, 47.43) | -51.63 (-120.45, 17.37) | -7.66 (-75.48, 59.79) | 19.17 (-42.48, 80.25) | N-acetylcysteine | -45.10 (-102.97, 13.82) | -13.57 (-89.02, 62.07) | -26.19 (-83.00, 29.52) |
| 21.86 (-10.44, 54.39) | 10.21 (-31.04, 49.40) | 29.33 (-13.95, 70.81) | 20.30 (-11.29, 50.81) | 18.46 (-15.38, 52.80) | 32.39 (12.47, 51.35) | -6.74 (-41.96, 27.23) | 37.40 (3.12, 70.22) | 63.64 (44.84, 82.60) | 45.10 (-13.82, 102.97) | Placebo | 31.66 (-18.93, 82.55) | 16.82 (-31.92, 67.53) |
| -9.79 (-70.99, 49.71) | -21.59 (-86.73, 43.26) | -2.72 (-66.99, 60.08) | -11.52 (-67.67, 45.11) | -13.39 (-72.76, 47.39) | 0.29 (-50.52, 50.48) | -38.63 (-100.18, 22.05) | 5.15 (-56.75, 65.78) | 32.53 (-23.80, 87.20) | 13.57 (-62.07, 89.02) | -31.66 (-82.55, 18.93) | Quercetin | -13.02 (-64.04, 38.10) |
| 3.33 (-33.91, 39.97) | -8.52 (-52.63, 35.55) | 10.21 (-29.56, 47.88) | 1.67 (-23.57, 26.54) | -0.30 (-38.67, 38.71) | 13.51 (-11.36, 37.09) | -25.62 (-65.95, 12.61) | 18.52 (-20.38, 56.32) | 45.42 (18.92, 71.49) | 26.19 (-29.52, 83.00) | -16.82 (-67.53, 31.92) | 13.02 (-38.10, 64.04) | Triamcinolone |
| -2.38 (-47.34, 41.74) | -14.19 (-65.59, 37.09) | 4.91 (-44.46, 52.11) | -3.92 (-43.52, 35.22) | -5.88 (-51.82, 39.28) | 8.02 (-26.74, 41.63) | -31.18 (-78.29, 15.15) | 12.79 (-32.48, 57.74) | 39.84 (2.68, 77.59) | 20.86 (-43.28, 85.22) | -22.01 (-78.08, 29.99) | 7.40 (-49.63, 64.43) | -5.64 (-35.54, 24.86) |
| -42.50 (-89.36, 4.75) | -54.03 (-108.12, -0.53) | -35.16 (-91.24, 19.08) | -43.84 (-91.67, 2.50) | -45.55 (-95.58, 2.58) | -31.78 (-73.13, 7.35) | -71.11 (-120.78, -20.91) | -26.94 (-76.39, 20.48) | -0.01 (-44.06, 43.51) | -19.29 (-87.32, 49.67) | -64.24 (-99.51, -29.05) | -32.09 (-94.98, 29.22) | -45.39 (-84.43, -6.61) |

| | |
|---|---|
| 1.72 (-43.93, 47.34) | 41.38 (-7.63, 91.44) |
| 20.49 (-20.08, 59.97) | 60.14 (14.12, 107.37) |
| -1.09 (-35.95, 33.65) | 38.66 (0.21, 79.13) |
| 10.72 (-31.13, 53.09) | 50.72 (3.96, 98.04) |
| 2.38 (-41.74, 47.34) | 42.50 (-4.75, 89.36) |
| 14.19 (-37.09, 65.59) | 54.03 (0.53, 108.12) |
| -4.91 (-52.11, 44.46) | 35.16 (-19.08, 91.24) |
| 3.92 (-35.22, 43.52) | 43.84 (-2.50, 91.67) |
| 5.88 (-39.28, 51.82) | 45.55 (-2.58, 95.58) |
| -8.02 (-41.63, 26.74) | 31.78 (-7.35, 73.13) |
| 31.18 (-15.15, 78.29) | 71.11 (20.91, 120.78) |
| -12.79 (-57.74, 32.48) | 26.94 (-20.48, 76.39) |
| -39.84 (-77.59, -2.68) | 0.01 (-43.51, 44.06) |
| -20.86 (-85.22, 43.28) | 19.29 (-49.67, 87.32) |
| 22.01 (-29.99, 78.08) | 64.24 (29.05, 99.51) |
| -7.40 (-64.43, 49.63) | 32.09 (-29.22, 94.98) |
| 5.64 (-24.86, 35.54) | 45.39 (6.61, 84.43) |
| Triester Glycerol Oxide | 40.24 (-6.34, 85.81) |
| -40.24 (-85.81, 6.34) | Zinc |

2.3 Variance Calculation

| Cycle | Median (95% CI) |
|---|---|
| Doxycycline, Laser, Placebo, Triamcinolone | -0.11 (-28.97, 26.24) |
| Doxycycline, Placebo, Triamcinolone | -1.01 (-48.44, 46.21) |
| Doxycycline, Placebo, Triamcinolone | -1.01 (-48.44, 46.21) |
| Doxycycline, Placebo, Triamcinolone | -1.01 (-48.44, 46.21) |

2.4 Convergence Diagnostics

| Parameter | PSRF |
|---|---|
| d.Benzydamine.Quercetin | 1.00 |
| d.Chlorhexidine.Nacetylcysteine | 1.00 |
| d.Placebo.Allicin | 1.00 |
| d.Placebo.Amlexanox | 1.00 |
| d.Placebo.Berberinegelatin | 1.00 |
| d.Placebo.Chitosan | 1.00 |
| d.Placebo.Diosmectite | 1.00 |
| d.Placebo.Glycyrrhiza | 1.00 |
| d.Placebo.Insulinliposomalgel | 1.15 |
| d.Placebo.Zinc | 1.00 |
| d.Triamcinolone.Aloe | 1.00 |
| d.Triamcinolone.Benzydamine | 1.00 |
| d.Triamcinolone.Chlorhexidine | 1.00 |
| d.Triamcinolone.Curcumin | 1.00 |
| d.Triamcinolone.Doxycycline | 1.00 |
| d.Triamcinolone.Laser | 1.00 |
| d.Triamcinolone.Placebo | 1.00 |
| d.Triamcinolone.TriesterGlycerol... | 1.00 |
| sd.d | 1.00 |

Number of chains : 4
Tuning iterations : 20,000
Simulation iterations : 50,000
Thinning interval : 10
Inference samples : 10,000
Variance scaling factor: 2.5

## 3. Node-splitting analysis

| Parameter | Median (95% CI) |
|---|---|
| Random Effects Standard Deviation | 14.66 (8.97, 26.74) |
| Inconsistency Standard Deviation | 11.28 (0.65, 69.10) |

## 4. Network structure

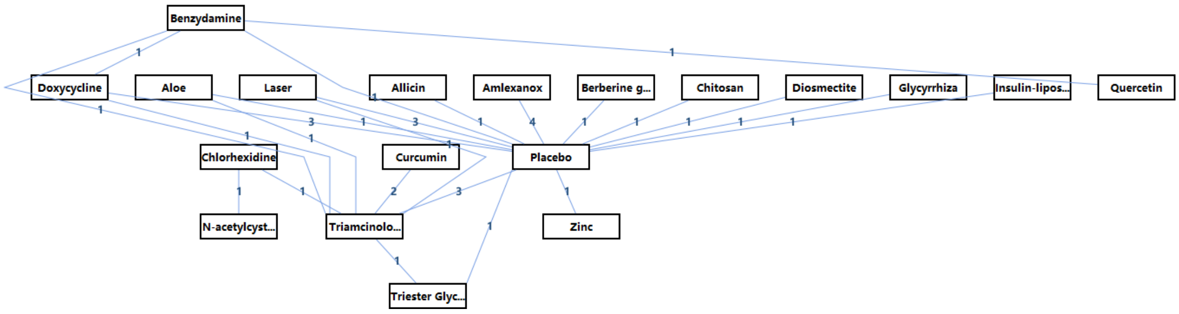

**Day 5**

## 1. Consistency Model

### 1.1 Summary estimates

| | | | | | | | | | | | | |
|---|---|---|---|---|---|---|---|---|---|---|---|---|
| Aloe | 8.86 (-34.03, 54.31) | 40.15 (-21.48, 103.51) | 33.83 (-21.19, 89.33) | 16.53 (-48.51, 82.12) | 0.65 (-59.35, 59.48) | 6.78 (-45.80, 57.36) | 5.35 (-41.26, 51.84) | 31.93 (-23.22, 87.73) | -14.62 (-55.44, 26.91) | -7.62 (-58.41, 44.39) | 31.29 (-8.86, 72.31) | 37.71 (-22.07, 100.28) |
| -8.86 (-54.31, 34.03) | Amlexanox | 30.70 (-26.60, 90.64) | 24.78 (-25.34, 73.95) | 7.64 (-40.60, 56.12) | -8.22 (-59.91, 42.26) | -2.21 (-44.11, 38.08) | -3.32 (-35.40, 27.19) | 23.19 (-19.68, 64.48) | -23.48 (-50.39, 2.53) | -15.94 (-57.81, 24.04) | 22.39 (-11.79, 55.08) | 29.32 (-22.30, 81.54) |
| -40.15 (-103.51, 21.48) | -30.70 (-90.64, 26.60) | Chlorhexidine | -6.54 (-69.60, 52.82) | -23.66 (-99.03, 50.89) | -39.41 (-115.39, 31.93) | -33.64 (-102.48, 32.39) | -34.69 (-98.69, 25.04) | -8.20 (-78.63, 60.39) | -54.53 (-115.42, 1.51) | -47.45 (-114.95, 17.31) | -8.55 (-57.85, 38.29) | -2.52 (-76.59, 71.65) |
| -33.83 (-89.33, 21.19) | -24.78 (-73.95, 25.34) | 6.54 (-52.82, 69.60) | Curcumin | -17.33 (-85.54, 51.75) | -33.07 (-98.34, 33.88) | -26.98 (-85.87, 31.60) | -28.54 (-81.91, 25.78) | -1.63 (-64.20, 60.28) | -48.26 (-97.21, 3.09) | -40.99 (-99.36, 18.63) | -2.32 (-39.13, 34.99) | 4.60 (-62.42, 72.38) |
| -16.53 (-82.12, 48.51) | -7.64 (-56.12, 40.60) | 23.66 (-50.89, 99.03) | 17.33 (-51.75, 85.54) | Dexamethasone | -15.66 (-86.31, 53.23) | -9.65 (-76.10, 52.55) | -10.62 (-69.76, 45.65) | 15.85 (-50.92, 78.16) | -30.66 (-86.97, 22.81) | -23.97 (-87.52, 39.27) | 15.06 (-42.91, 72.14) | 21.50 (-51.36, 93.60) |
| -0.65 (-59.48, 59.35) | 8.22 (-42.26, 59.91) | 39.41 (-31.93, 115.39) | 33.07 (-33.88, 98.34) | 15.66 (-53.23, 86.31) | Diosmectite | 5.93 (-48.04, 59.81) | 4.94 (-44.54, 54.21) | 31.68 (-29.95, 90.27) | -15.01 (-59.17, 28.45) | -7.90 (-61.36, 46.38) | 30.67 (-23.00, 86.79) | 37.54 (-25.09, 101.16) |
| -6.78 (-57.36, 45.80) | 2.21 (-38.08, 44.11) | 33.64 (-32.39, 102.48) | 26.98 (-31.60, 85.87) | 9.65 (-52.55, 76.10) | -5.93 (-59.81, 48.04) | Doxycycline | -1.01 (-40.02, 37.66) | 25.19 (-23.74, 76.13) | -21.07 (-52.64, 10.92) | -13.80 (-58.49, 31.97) | 24.90 (-20.66, 71.09) | 31.58 (-21.76, 86.47) |
| -5.35 (-51.84, 41.26) | 3.32 (-27.19, 35.40) | 34.69 (-25.04, 98.69) | 28.54 (-25.78, 81.91) | 10.62 (-45.65, 69.76) | -4.94 (-54.21, 44.54) | 1.01 (-37.66, 40.02) | Glycyrrhiza | 26.88 (-14.92, 67.45) | -20.08 (-41.31, 2.53) | -12.65 (-51.28, 25.92) | 25.77 (-12.07, 64.73) | 32.65 (-16.17, 83.53) |
| -31.93 (-87.73, 23.22) | -23.19 (-64.48, 19.68) | 8.20 (-60.39, 78.63) | 1.63 (-60.28, 64.20) | -15.85 (-78.16, 50.92) | -31.68 (-90.27, 29.95) | -25.19 (-76.13, 23.74) | -26.88 (-67.45, 14.92) | Laser | -46.59 (-85.72, -6.63) | -39.21 (-89.55, 12.84) | -0.36 (-49.94, 49.34) | 5.92 (-53.31, 67.63) |
| 14.62 (-26.91, 55.44) | 23.48 (-2.53, 50.39) | 54.53 (-1.51, 115.42) | 48.26 (-3.09, 97.21) | 30.66 (-22.81, 86.97) | 15.01 (-28.45, 59.17) | 21.07 (-10.92, 52.64) | 20.08 (-2.53, 41.31) | 46.59 (6.63, 85.72) | Placebo | 7.18 (-24.35, 38.85) | 45.80 (12.78, 79.09) | 52.81 (9.11, 98.47) |
| 7.62 (-44.39, 58.41) | 15.94 (-24.04, 57.81) | 47.45 (-17.31, 114.95) | 40.99 (-18.63, 99.36) | 23.97 (-39.27, 87.52) | 7.90 (-46.38, 61.36) | 13.80 (-31.97, 58.49) | 12.65 (-25.92, 51.28) | 39.21 (-12.84, 89.55) | -7.18 (-38.85, 24.35) | Probiotics | 38.73 (-6.15, 84.22) | 45.70 (-9.48, 99.63) |
| -31.29 (-72.31, 8.86) | -22.39 (-55.08, 11.79) | 8.55 (-38.29, 57.85) | 2.32 (-34.99, 39.13) | -15.06 (-72.14, 42.91) | -30.67 (-86.79, 23.00) | -24.90 (-71.09, 20.66) | -25.77 (-64.73, 12.07) | 0.36 (-49.34, 49.94) | -45.80 (-79.09, -12.78) | -38.73 (-84.22, 6.15) | Triamcinolone | 6.61 (-48.82, 63.15) |
| -37.71 (-100.28, 22.07) | -29.32 (-81.54, 22.30) | 2.52 (-71.65, 76.59) | -4.60 (-72.38, 62.42) | -21.50 (-93.60, 51.36) | -37.54 (-101.16, 25.09) | -31.58 (-86.47, 21.76) | -32.65 (-83.53, 16.17) | -5.92 (-67.63, 53.31) | -52.81 (-98.47, -9.11) | -45.70 (-99.63, 9.48) | -6.61 (-63.15, 48.82) | Zinc |

## 1.2 Rank probability(Rank 1 is best, rank N is worst)

| Drug | Rank 1 | Rank 2 | Rank 3 | Rank 4 | Rank 5 | Rank 6 | Rank 7 | Rank 8 | Rank 9 | Rank 10 | Rank 11 | Rank 12 | Rank 13 |
|---|---|---|---|---|---|---|---|---|---|---|---|---|---|
| Aloe | 0 | 0.01 | 0.02 | 0.03 | 0.05 | 0.08 | 0.09 | 0.1 | 0.12 | 0.13 | 0.13 | 0.11 | 0.14 |
| Amlexanox | 0 | 0 | 0.01 | 0.03 | 0.07 | 0.13 | 0.2 | 0.2 | 0.16 | 0.11 | 0.06 | 0.02 | 0.01 |
| Chlorhexidine | 0.31 | 0.18 | 0.13 | 0.1 | 0.07 | 0.05 | 0.04 | 0.03 | 0.02 | 0.02 | 0.01 | 0.01 | 0.01 |
| Curcumin | 0.16 | 0.19 | 0.16 | 0.13 | 0.11 | 0.08 | 0.05 | 0.04 | 0.03 | 0.02 | 0.02 | 0.01 | 0.01 |
| Dexamethasone | 0.07 | 0.08 | 0.08 | 0.08 | 0.1 | 0.11 | 0.09 | 0.08 | 0.07 | 0.06 | 0.06 | 0.05 | 0.07 |
| Diosmectite | 0.01 | 0.02 | 0.03 | 0.04 | 0.05 | 0.07 | 0.08 | 0.09 | 0.11 | 0.12 | 0.12 | 0.11 | 0.15 |
| Doxycycline | 0.01 | 0.02 | 0.03 | 0.05 | 0.07 | 0.11 | 0.12 | 0.13 | 0.13 | 0.13 | 0.1 | 0.06 | 0.04 |
| Glycyrrhiza | 0 | 0 | 0.01 | 0.03 | 0.05 | 0.09 | 0.14 | 0.17 | 0.19 | 0.17 | 0.1 | 0.04 | 0.01 |
| Laser | 0.14 | 0.18 | 0.14 | 0.14 | 0.14 | 0.09 | 0.06 | 0.04 | 0.03 | 0.02 | 0.01 | 0.01 | 0 |
| Placebo | 0 | 0 | 0 | 0 | 0 | 0 | 0 | 0.01 | 0.02 | 0.07 | 0.2 | 0.37 | 0.34 |
| Probiotics | 0 | 0 | 0.01 | 0.01 | 0.02 | 0.04 | 0.05 | 0.07 | 0.09 | 0.13 | 0.17 | 0.19 | 0.21 |
| Triamcinolone | 0.03 | 0.13 | 0.25 | 0.24 | 0.18 | 0.08 | 0.04 | 0.02 | 0.01 | 0.01 | 0 | 0 | 0 |
| Zinc | 0.27 | 0.18 | 0.13 | 0.11 | 0.1 | 0.07 | 0.05 | 0.03 | 0.02 | 0.02 | 0.01 | 0.01 | 0.01 |

## 1.3 Consistency check

| Parameter | Median (95% CI) |
|---|---|
| Random Effects Standard Deviation | 18.93 (11.29, 37.41) |

## 1.4 Convergence Diagnostics

| Parameter | PSRF |
|---|---|
| d.Amlexanox.Dexamethasone | 1.00 |
| d.Placebo.Aloe | 1.00 |
| d.Placebo.Amlexanox | 1.00 |
| d.Placebo.Diosmectite | 1.00 |
| d.Placebo.Doxycycline | 1.00 |
| d.Placebo.Glycyrrhiza | 1.01 |
| d.Placebo.Laser | 1.00 |
| d.Placebo.Probiotics | 1.00 |
| d.Placebo.Triamcinolone | 1.00 |
| d.Placebo.Zinc | 1.00 |
| d.Triamcinolone.Chlorhexidine | 1.00 |
| d.Triamcinolone.Curcumin | 1.00 |
| sd.d | 1.00 |

Number of chains : 4
Tuning iterations : 20,000
Simulation iterations : 50,000
Thinning interval : 10
Inference samples : 10,000
Variance scaling factor: 2.5

## 2.Inconsistency Model

### 2.1 Summary estimates

| | | |
|---|---|---|
| Aloe | -2.13 (-53.22, 53.94) | 36.70 (-26.63, 103.43) |
| 2.13 (-53.94, 53.22) | Amlexanox | 39.07 (-24.02, 99.89) |
| -36.70 (-103.43, 26.63) | -39.07 (-99.89, 24.02) | Chlorhexidine |
| -29.95 (-89.24, 25.51) | -31.87 (-83.62, 23.28) | 7.30 (-54.10, 68.97) |
| -5.49 (-82.50, 64.65) | -7.98 (-55.72, 40.00) | 31.58 (-48.75, 108.07) |
| -4.61 (-64.36, 60.71) | -6.19 (-65.21, 59.06) | 32.95 (-40.04, 111.49) |
| -10.05 (-61.46, 45.43) | -11.29 (-63.57, 43.59) | 26.59 (-40.02, 99.42) |
| -10.37 (-55.15, 40.47) | 0.25 (-35.80, 37.60) | 26.86 (-34.44, 95.64) |
| -19.27 (-86.67, 44.52) | -21.38 (-63.26, 22.80) | 18.04 (-55.32, 90.10) |
| 10.85 (-29.96, 55.36) | 20.38 (-37.20, 70.85) | 48.25 (-10.69, 112.01) |
| 4.00 (-48.26, 59.07) | 2.61 (-49.78, 57.98) | 41.24 (-26.46, 112.04) |
| -27.94 (-74.25, 14.52) | -30.21 (-67.81, 8.74) | 9.32 (-39.56, 56.63) |
| -41.82 (-102.81, 20.11) | -43.30 (-105.33, 20.96) | -4.43 (-81.27, 73.48) |

| | | | | | | | | | |
|---|---|---|---|---|---|---|---|---|---|
| 29.95 (-25.51, 89.24) | 5.49 (-64.65, 82.50) | 4.61 (-60.71, 64.36) | 10.05 (-45.43, 61.46) | 10.37 (-40.47, 55.15) | 19.27 (-44.52, 86.67) | -10.85 (-55.36, 29.96) | -4.00 (-59.07, 48.26) | 27.94 (-14.52, 74.25) | 41.82 (-20.11, 102.81) |
| 31.87 (-23.28, 83.62) | 7.98 (-40.00, 55.72) | 6.19 (-59.06, 65.21) | 11.29 (-43.59, 63.57) | -0.25 (-37.60, 35.80) | 21.38 (-22.80, 63.26) | -20.38 (-70.85, 37.20) | -2.61 (-57.98, 49.78) | 30.21 (-8.74, 67.81) | 43.30 (-20.96, 105.33) |
| -7.30 (-68.97, 54.10) | -31.58 (-108.07, 48.75) | -32.95 (-111.49, 40.04) | -26.59 (-99.42, 40.02) | -26.86 (-95.64, 34.44) | -18.04 (-90.10, 55.32) | -48.25 (-112.01, 10.69) | -41.24 (-112.04, 26.46) | -9.32 (-56.63, 39.56) | 4.43 (-73.48, 81.27) |
| Curcumin | -24.36 (-94.70, 49.58) | -25.14 (-97.05, 40.50) | -19.49 (-84.31, 39.15) | -19.91 (-78.89, 34.00) | -10.75 (-75.68, 56.73) | -40.72 (-96.72, 10.25) | -33.69 (-98.24, 25.81) | -1.57 (-38.69, 35.21) | 11.88 (-59.52, 80.21) |
| 24.36 (-49.58, 94.70) | Dexamethasone | -1.95 (-85.80, 73.73) | 3.95 (-73.53, 73.58) | 3.84 (-65.84, 67.97) | 13.51 (-52.28, 75.90) | -17.17 (-86.10, 45.65) | -9.77 (-85.99, 59.48) | 22.60 (-40.71, 82.25) | 36.17 (-47.11, 112.12) |
| 25.14 (-40.50, 97.05) | 1.95 (-73.73, 85.80) | Diosmectite | 5.77 (-49.67, 62.07) | 5.56 (-46.14, 56.84) | 15.23 (-54.82, 88.27) | -15.68 (-60.10, 30.20) | -8.23 (-63.55, 48.60) | 23.72 (-32.06, 86.88) | 37.40 (-27.23, 103.36) |
| 19.49 (-39.15, 84.31) | -3.95 (-73.58, 73.53) | -5.77 (-62.07, 49.67) | Doxycycline | 0.23 (-42.68, 40.31) | 9.89 (-53.96, 74.72) | -21.09 (-53.81, 10.84) | -13.97 (-58.97, 31.71) | 18.04 (-29.01, 71.84) | 31.55 (-24.44, 88.99) |
| 19.91 (-34.00, 78.89) | -3.84 (-67.97, 65.84) | -5.56 (-56.84, 46.14) | -0.23 (-40.31, 42.68) | Glycyrrhiza | 21.85 (-22.79, 64.44) | -21.34 (-45.65, 4.02) | -13.93 (-55.00, 27.11) | 18.08 (-21.20, 63.73) | 31.48 (-21.04, 86.36) |
| 10.75 (-56.73, 75.68) | -13.51 (-75.90, 52.28) | -15.23 (-88.27, 54.82) | -9.89 (-74.72, 53.96) | -21.85 (-64.44, 22.79) | Laser | -41.82 (-98.70, 25.67) | -24.30 (-89.28, 41.17) | 8.97 (-44.02, 63.20) | 21.85 (-50.03, 92.80) |
| 40.72 (-10.25, 96.72) | 17.17 (-45.65, 86.10) | 15.68 (-30.20, 60.10) | 21.09 (-10.84, 53.81) | 21.34 (-4.02, 45.65) | 41.82 (-25.67, 98.70) | Placebo | 7.21 (-24.85, 39.58) | 39.41 (3.28, 80.37) | 52.66 (8.03, 99.50) |
| 33.69 (-25.81, 98.24) | 9.77 (-59.48, 85.99) | 8.23 (-48.60, 63.55) | 13.97 (-31.71, 58.97) | 13.93 (-27.11, 55.00) | 24.30 (-41.17, 89.28) | -7.21 (-39.58, 24.85) | Probiotics | 32.17 (-15.07, 84.84) | 45.68 (-11.20, 102.41) |
| 1.57 (-35.21, 38.69) | -22.60 (-82.25, 40.71) | -23.72 (-86.88, 32.06) | -18.04 (-71.84, 29.01) | -18.08 (-63.73, 21.20) | -8.97 (-63.20, 44.02) | -39.41 (-80.37, -3.28) | -32.17 (-84.84, 15.07) | Triamcinolone | 13.72 (-48.70, 70.60) |
| -11.88 (-80.21, 59.52) | -36.17 (-112.12, 47.11) | -37.40 (-103.36, 27.23) | -31.55 (-88.99, 24.44) | -31.48 (-86.36, 21.04) | -21.85 (-92.80, 50.03) | -52.66 (-99.50, -8.03) | -45.68 (-102.41, 11.20) | -13.72 (-70.60, 48.70) | Zinc |

2.2 Inconsistency Factors

| Cycle | Median (95% CI) |
|---|---|
| Amlexanox, Glycyrrhiza, Placebo, Triamcinolone | -6.32 (-61.36, 25.69) |
| Amlexanox, Placebo, Triamcinolone | -4.90 (-66.70, 35.23) |
| Amlexanox, Placebo, Triamcinolone | -4.90 (-66.70, 35.23) |

## 2.3 Variance Calculation

| Parameter | Median (95% CI) |
| --- | --- |
| Random Effects Standard Deviation | 18.78 (10.27, 39.91) |
| Inconsistency Standard Deviation | 22.19 (0.97, 58.63) |

## 2.4 Convergence Diagnostics

| Parameter | PSRF |
| --- | --- |
| d.Aloe.Placebo | 1.00 |
| d.Amlexanox.Dexamethasone | 1.00 |
| d.Amlexanox.Laser | 1.00 |
| d.Placebo.Diosmectite | 1.00 |
| d.Placebo.Doxycycline | 1.00 |
| d.Placebo.Glycyrrhiza | 1.01 |
| d.Placebo.Probiotics | 1.00 |
| d.Placebo.Triamcinolone | 1.00 |
| d.Placebo.Zinc | 1.00 |
| d.Triamcinolone.Amlexanox | 1.00 |
| d.Triamcinolone.Chlorhexidine | 1.00 |
| d.Triamcinolone.Curcumin | 1.00 |
| w.Amlexanox.Glycyrrhiza.Placeb... | 1.00 |
| w.Amlexanox.Placebo.Triamcinol... | 1.00 |
| w.Amlexanox.Placebo.Triamcinol... | 1.00 |
| sd.d | 1.01 |
| sd.w | 1.00 |

Number of chains : 4
Tuning iterations : 20,000
Simulation iterations : 50,000
Thinning interval : 10
Inference samples : 10,000
Variance scaling factor: 2.5

## 3. Node-splitting analysis

| Name | Direct Effect | Indirect Effect | Overall | P-Value |
| --- | --- | --- | --- | --- |
| Amlexanox, Glycyrrhiza | -15.42 (-69.31, 33.74) | -2.94 (-48.68, 41.88) | -3.32 (-35.40, 27.19) | 0.67 |
| Amlexanox, Placebo | -33.20 (-68.93, -0.68) | -14.02 (-57.49, 29.16) | -23.48 (-50.39, 2.53) | 0.41 |
| Amlexanox, Triamcinolone | 42.12 (2.87, 82.51) | -6.95 (-57.71, 41.05) | 22.39 (-11.79, 55.08) | 0.1 |
| Placebo, Triamcinolone | 26.83 (-14.03, 66.31) | 76.67 (29.75, 125.97) | 45.80 (12.78, 79.09) | 0.1 |

## 4. Network structure

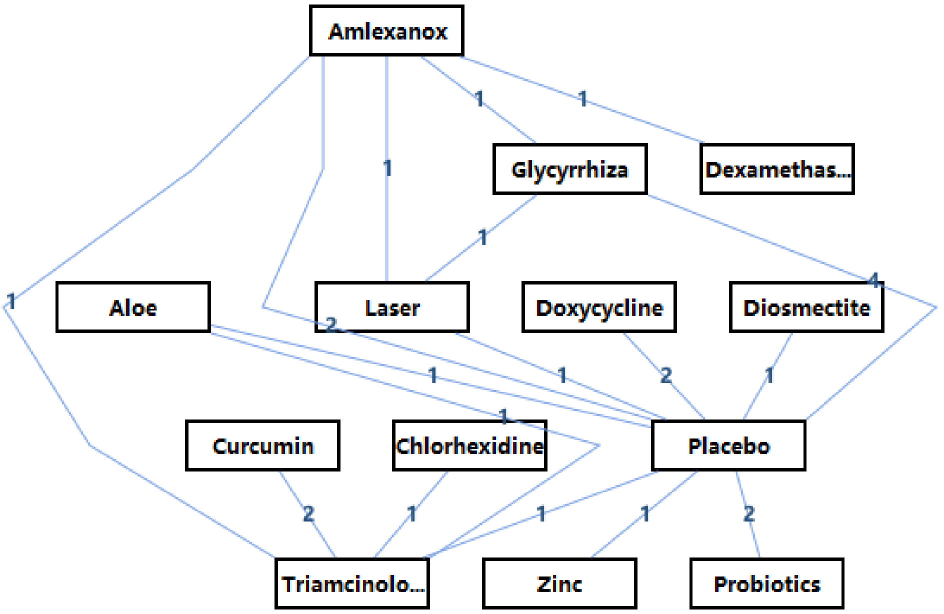

**Day 6**

## 1. Consistency Model

1.1 Summary estimates

| | | | | | | | | | | |
|---|---|---|---|---|---|---|---|---|---|---|
| Allicin | -15.58 (-93.18, 61.40) | 8.41 (-66.14, 81.71) | -8.29 (-96.30, 80.70) | -14.51 (-106.44, 77.58) | 4.75 (-91.21, 103.46) | 10.21 (-86.53, 109.21) | -10.46 (-100.81, 79.82) | -6.46 (-98.92, 81.03) | -13.27 (-90.17, 65.60) | 27.12 (-64.78, 115.94) |
| 15.58 (-61.40, 93.18) | Aloe | 24.03 (-34.90, 78.37) | 7.28 (-69.27, 82.73) | 1.60 (-79.51, 78.74) | 20.66 (-61.48, 103.64) | 25.66 (-53.05, 105.99) | 4.74 (-72.51, 80.50) | 8.84 (-69.61, 84.87) | 2.61 (-60.65, 63.29) | 42.21 (-36.21, 120.26) |
| -8.41 (-81.71, 66.14) | -24.03 (-78.37, 34.90) | Amlexanox | -16.38 (-89.11, 56.13) | -23.24 (-98.40, 54.31) | -3.40 (-85.77, 81.70) | 1.64 (-77.22, 85.69) | -19.23 (-90.68, 54.33) | -15.14 (-87.83, 56.75) | -21.30 (-78.45, 37.75) | 18.53 (-54.93, 91.28) |
| 8.29 (-80.70, 96.30) | -7.28 (-82.73, 69.27) | 16.38 (-56.13, 89.11) | Berberine gelatin | -6.80 (-97.61, 86.22) | 12.74 (-81.43, 111.82) | 18.40 (-75.37, 113.49) | -2.51 (-90.75, 87.60) | 1.21 (-86.13, 90.11) | -4.89 (-79.65, 73.32) | 35.27 (-53.42, 124.28) |
| 14.51 (-77.58, 106.44) | -1.60 (-78.74, 79.51) | 23.24 (-54.31, 98.40) | 6.80 (-86.22, 97.61) | Chitosan | 19.40 (-77.48, 119.93) | 25.30 (-73.34, 122.65) | 3.83 (-87.99, 96.18) | 8.07 (-86.29, 99.80) | 2.13 (-77.93, 82.40) | 40.79 (-49.35, 133.70) |
| -4.75 (-103.46, 91.21) | -20.66 (-103.64, 61.48) | 3.40 (-81.70, 85.77) | -12.74 (-111.82, 81.43) | -19.40 (-119.93, 77.48) | Chlorhexidine | 5.55 (-86.71, 94.49) | -15.87 (-113.41, 80.81) | -10.96 (-109.31, 84.04) | -18.10 (-107.72, 68.36) | 22.00 (-80.99, 118.36) |
| -10.21 (-109.21, 86.53) | -25.66 (-105.99, 53.05) | -1.64 (-85.69, 77.22) | -18.40 (-113.49, 75.37) | -25.30 (-122.65, 73.34) | -5.55 (-94.49, 86.71) | Curcumin | -21.46 (-119.23, 75.18) | -17.18 (-114.44, 78.34) | -22.70 (-109.22, 61.74) | 16.78 (-83.06, 112.80) |
| 10.46 (-79.82, 100.81) | -4.74 (-80.50, 72.51) | 19.23 (-54.33, 90.68) | 2.51 (-87.60, 90.75) | -3.83 (-96.18, 87.99) | 15.87 (-80.81, 113.41) | 21.46 (-75.18, 119.23) | Dexamethasone | 4.31 (-87.54, 91.84) | -1.93 (-78.82, 76.31) | 37.66 (-52.21, 126.29) |
| 6.46 (-81.03, 98.92) | -8.84 (-84.87, 69.61) | 15.14 (-56.75, 87.83) | -1.21 (-90.11, 86.13) | -8.07 (-99.80, 86.29) | 10.96 (-84.04, 109.31) | 17.18 (-78.34, 114.44) | -4.31 (-91.84, 87.54) | Diosmectite | -6.40 (-84.60, 71.14) | 33.90 (-55.81, 124.48) |
| 13.27 (-65.60, 90.17) | -2.61 (-63.29, 60.65) | 21.30 (-37.75, 78.45) | 4.89 (-73.32, 79.65) | -2.13 (-82.40, 77.93) | 18.10 (-68.36, 107.72) | 22.70 (-61.74, 109.22) | 1.93 (-76.31, 78.82) | 6.40 (-71.14, 84.60) | Doxycycline | 39.81 (-40.48, 116.70) |
| -27.12 (-115.94, 64.78) | -42.21 (-120.26, 36.21) | -18.53 (-91.28, 54.93) | -35.27 (-124.28, 53.42) | -40.79 (-133.70, 49.35) | -22.00 (-118.36, 80.99) | -16.78 (-112.80, 83.06) | -37.66 (-126.29, 52.21) | -33.90 (-124.48, 55.81) | -39.81 (-116.70, 40.48) | Insulin-liposomal gel |
| -35.60 (-132.39, 61.32) | -51.62 (-134.64, 35.77) | -28.00 (-107.42, 54.12) | -44.06 (-139.04, 51.53) | -50.21 (-146.46, 47.82) | -31.12 (-131.89, 72.43) | -25.29 (-126.48, 77.54) | -46.33 (-143.35, 48.23) | -43.05 (-137.10, 54.32) | -48.46 (-133.45, 36.68) | -8.96 (-103.55, 87.15) |
| -14.08 (-132.61, 104.84) | -30.00 (-134.03, 75.84) | -5.66 (-113.05, 100.00) | -22.22 (-141.78, 96.81) | -28.62 (-146.50, 91.85) | -8.61 (-73.78, 59.27) | -3.65 (-114.73, 108.86) | -24.53 (-143.77, 93.83) | -20.45 (-139.96, 96.71) | -26.56 (-134.95, 83.91) | 12.97 (-107.16, 132.41) |
| 23.71 (-40.14, 87.24) | 8.17 (-34.62, 52.18) | 32.31 (-5.64, 67.60) | 15.60 (-47.75, 78.00) | 9.14 (-57.49, 74.02) | 28.75 (-44.93, 104.56) | 33.85 (-37.82, 107.48) | 12.92 (-50.08, 75.18) | 17.41 (-46.89, 81.44) | 11.34 (-33.04, 55.41) | 50.76 (-13.42, 114.19) |
| -0.87 (-76.85, 72.28) | -16.41 (-65.70, 32.38) | 7.55 (-46.15, 58.10) | -9.07 (-83.63, 60.35) | -15.20 (-92.40, 59.29) | 4.32 (-61.10, 70.10) | 9.29 (-55.34, 72.46) | -11.95 (-86.16, 61.60) | -7.40 (-81.85, 64.47) | -13.87 (-73.35, 42.34) | 25.94 (-49.24, 98.32) |
| 1.72 (-88.56, 86.47) | -14.31 (-82.89, 54.41) | 9.83 (-58.52, 78.28) | -6.73 (-94.71, 75.92) | -13.08 (-101.58, 73.17) | 6.09 (-80.50, 91.48) | 11.71 (-74.17, 97.63) | -8.94 (-95.91, 75.52) | -5.51 (-91.16, 79.79) | -11.48 (-85.88, 61.08) | 28.02 (-59.38, 112.80) |
| -15.54 (-107.60, 74.92) | -31.28 (-108.12, 48.65) | -7.07 (-82.47, 68.66) | -24.22 (-111.61, 65.79) | -30.42 (-123.04, 63.84) | -10.76 (-106.82, 92.51) | -5.67 (-103.32, 95.25) | -26.39 (-118.61, 65.73) | -21.79 (-116.15, 67.91) | -28.87 (-107.49, 50.82) | 11.23 (-79.45, 101.12) |

| | | | | | |
|---|---|---|---|---|---|
| 35.60 (-61.32, 132.39) | 14.08 (-104.84, 132.61) | -23.71 (-87.24, 40.14) | 0.87 (-72.28, 76.85) | -1.72 (-86.47, 88.56) | 15.54 (-74.92, 107.60) |
| 51.62 (-35.77, 134.64) | 30.00 (-75.84, 134.03) | -8.17 (-52.18, 34.62) | 16.41 (-32.38, 65.70) | 14.31 (-54.41, 82.89) | 31.28 (-48.65, 108.12) |
| 28.00 (-54.12, 107.42) | 5.66 (-100.00, 113.05) | -32.31 (-67.60, 5.64) | -7.55 (-58.10, 46.15) | -9.83 (-78.28, 58.52) | 7.07 (-68.66, 82.47) |
| 44.06 (-51.53, 139.04) | 22.22 (-96.81, 141.78) | -15.60 (-78.00, 47.75) | 9.07 (-60.35, 83.63) | 6.73 (-75.92, 94.71) | 24.22 (-65.79, 111.61) |
| 50.21 (-47.82, 146.46) | 28.62 (-91.85, 146.50) | -9.14 (-74.02, 57.49) | 15.20 (-59.29, 92.40) | 13.08 (-73.17, 101.58) | 30.42 (-63.84, 123.04) |
| 31.12 (-72.43, 131.89) | 8.61 (-59.27, 73.78) | -28.75 (-104.56, 44.93) | -4.32 (-70.10, 61.10) | -6.09 (-91.48, 80.50) | 10.76 (-92.51, 106.82) |
| 25.29 (-77.54, 126.48) | 3.65 (-108.86, 114.73) | -33.85 (-107.48, 37.82) | -9.29 (-72.46, 55.34) | -11.71 (-97.63, 74.17) | 5.67 (-95.25, 103.32) |
| 46.33 (-48.23, 143.35) | 24.53 (-93.83, 143.77) | -12.92 (-75.18, 50.08) | 11.95 (-61.60, 86.16) | 8.94 (-75.52, 95.91) | 26.39 (-65.73, 118.61) |
| 43.05 (-54.32, 137.10) | 20.45 (-96.71, 139.96) | -17.41 (-81.44, 46.89) | 7.40 (-64.47, 81.85) | 5.51 (-79.79, 91.16) | 21.79 (-67.91, 116.15) |
| 48.46 (-36.68, 133.45) | 26.56 (-83.91, 134.95) | -11.34 (-55.41, 33.04) | 13.87 (-42.34, 73.35) | 11.48 (-61.08, 85.88) | 28.87 (-50.82, 107.49) |
| 8.96 (-87.15, 103.55) | -12.97 (-132.41, 107.16) | -50.76 (-114.19, 13.42) | -25.94 (-98.32, 49.24) | -28.02 (-112.80, 59.38) | -11.23 (-101.12, 79.45) |
| Laser | -21.94 (-146.26, 102.10) | -59.65 (-130.10, 12.85) | -35.02 (-113.22, 48.18) | -36.93 (-127.00, 55.47) | -21.08 (-115.42, 72.78) |
| 21.94 (-102.10, 146.26) | N-acetylcysteine | -38.07 (-138.00, 62.75) | -12.81 (-105.96, 80.60) | -15.72 (-123.50, 95.09) | 2.18 (-118.18, 119.62) |
| 59.65 (-12.85, 130.10) | 38.07 (-62.75, 138.00) | Placebo | 24.71 (-11.06, 61.67) | 22.40 (-34.44, 80.55) | 39.68 (-26.60, 104.99) |
| 35.02 (-48.18, 113.22) | 12.81 (-80.60, 105.96) | -24.71 (-61.67, 11.06) | Triamcinolone | -2.35 (-58.83, 55.39) | 14.76 (-62.20, 87.93) |
| 36.93 (-55.47, 127.00) | 15.72 (-95.09, 123.50) | -22.40 (-80.55, 34.44) | 2.35 (-55.39, 58.83) | Triester Glycerol Oxide | 17.27 (-72.68, 101.91) |
| 21.08 (-72.78, 115.42) | -2.18 (-119.62, 118.18) | -39.68 (-104.99, 26.60) | -14.76 (-87.93, 62.20) | -17.27 (-101.91, 72.68) | Zinc |

## 1.2 Rank probability(Rank 1 is best, rank N is worst)

| Drug | Rank 1 | Rank 2 | Rank 3 | Rank 4 | Rank 5 | Rank 6 | Rank 7 | Rank 8 | Rank 9 | Rank 10 | Rank 11 | Rank 12 | Rank 13 | Rank 14 | Rank 15 | Rank 16 | Rank 17 |
|---|---|---|---|---|---|---|---|---|---|---|---|---|---|---|---|---|---|
| Allicin | 0.03 | 0.05 | 0.06 | 0.06 | 0.07 | 0.07 | 0.06 | 0.07 | 0.07 | 0.06 | 0.06 | 0.06 | 0.06 | 0.05 | 0.05 | 0.06 | 0.06 |
| Aloe | 0 | 0.01 | 0.01 | 0.02 | 0.02 | 0.03 | 0.04 | 0.05 | 0.06 | 0.08 | 0.09 | 0.1 | 0.1 | 0.1 | 0.1 | 0.1 | 0.08 |
| Amlexanox | 0.01 | 0.04 | 0.07 | 0.1 | 0.12 | 0.11 | 0.11 | 0.1 | 0.08 | 0.08 | 0.06 | 0.05 | 0.03 | 0.02 | 0.01 | 0.01 | 0 |
| Berberine gelatin | 0.02 | 0.03 | 0.04 | 0.05 | 0.05 | 0.06 | 0.06 | 0.06 | 0.06 | 0.07 | 0.07 | 0.07 | 0.07 | 0.07 | 0.07 | 0.08 | 0.1 |
| Chitosan | 0.02 | 0.03 | 0.03 | 0.04 | 0.04 | 0.04 | 0.04 | 0.05 | 0.05 | 0.05 | 0.06 | 0.06 | 0.06 | 0.07 | 0.07 | 0.1 | 0.17 |
| Chlorhexidine | 0.03 | 0.08 | 0.09 | 0.08 | 0.07 | 0.07 | 0.06 | 0.06 | 0.06 | 0.06 | 0.05 | 0.05 | 0.05 | 0.04 | 0.05 | 0.06 | 0.04 |
| Curcumin | 0.09 | 0.09 | 0.09 | 0.08 | 0.08 | 0.07 | 0.06 | 0.06 | 0.05 | 0.05 | 0.05 | 0.04 | 0.04 | 0.03 | 0.04 | 0.03 | 0.04 |
| Dexamethasone | 0.02 | 0.03 | 0.04 | 0.05 | 0.04 | 0.05 | 0.05 | 0.05 | 0.06 | 0.06 | 0.06 | 0.07 | 0.07 | 0.07 | 0.08 | 0.09 | 0.12 |
| Diosmectite | 0.02 | 0.03 | 0.04 | 0.05 | 0.05 | 0.06 | 0.05 | 0.06 | 0.06 | 0.06 | 0.06 | 0.07 | 0.07 | 0.06 | 0.06 | 0.08 | 0.09 |
| Doxycycline | 0 | 0.01 | 0.01 | 0.02 | 0.03 | 0.04 | 0.05 | 0.06 | 0.06 | 0.08 | 0.09 | 0.09 | 0.1 | 0.09 | 0.09 | 0.08 | 0.07 |
| Insulin-liposomal gel | 0.17 | 0.18 | 0.13 | 0.1 | 0.08 | 0.06 | 0.05 | 0.04 | 0.03 | 0.03 | 0.03 | 0.02 | 0.02 | 0.02 | 0.01 | 0.01 | 0.01 |
| Laser | 0.32 | 0.15 | 0.11 | 0.07 | 0.06 | 0.05 | 0.04 | 0.04 | 0.03 | 0.02 | 0.02 | 0.02 | 0.02 | 0.02 | 0.01 | 0.01 | 0.01 |
| N-acetylcysteine | 0.16 | 0.11 | 0.09 | 0.07 | 0.06 | 0.05 | 0.05 | 0.04 | 0.04 | 0.04 | 0.04 | 0.03 | 0.03 | 0.03 | 0.04 | 0.05 | 0.08 |
| Placebo | 0 | 0 | 0 | 0 | 0 | 0 | 0 | 0.01 | 0.01 | 0.03 | 0.06 | 0.1 | 0.15 | 0.21 | 0.22 | 0.16 | 0.05 |
| Triamcinolone | 0 | 0.01 | 0.02 | 0.04 | 0.07 | 0.1 | 0.12 | 0.13 | 0.13 | 0.12 | 0.1 | 0.07 | 0.05 | 0.03 | 0.02 | 0.01 | 0 |
| Triester Glycerol Oxide | 0.02 | 0.04 | 0.05 | 0.06 | 0.07 | 0.07 | 0.08 | 0.07 | 0.07 | 0.07 | 0.07 | 0.07 | 0.06 | 0.06 | 0.05 | 0.05 | 0.04 |
| Zinc | 0.09 | 0.12 | 0.12 | 0.1 | 0.08 | 0.07 | 0.06 | 0.06 | 0.05 | 0.04 | 0.04 | 0.03 | 0.04 | 0.03 | 0.03 | 0.03 | 0.02 |

1.3 Consistency check

| Parameter | Median (95% CI) |
|---|---|
| Random Effects Standard Deviation | 26.71 (14.77, 55.74) |

1.4 Convergence Diagnostics

| Parameter | PSRF |
|---|---|
| d.Chlorhexidine.Nacetylcysteine | 1.00 |
| d.Placebo.Allicin | 1.00 |
| d.Placebo.Amlexanox | 1.00 |
| d.Placebo.Berberinegelatin | 1.00 |
| d.Placebo.Chitosan | 1.00 |
| d.Placebo.Dexamethasone | 1.00 |
| d.Placebo.Diosmectite | 1.00 |
| d.Placebo.Doxycycline | 1.00 |
| d.Placebo.Insulinliposomalgel | 1.03 |
| d.Placebo.Laser | 1.34 |
| d.Placebo.Zinc | 1.09 |
| d.Triamcinolone.Aloe | 1.00 |
| d.Triamcinolone.Chlorhexidine | 1.00 |
| d.Triamcinolone.Curcumin | 1.03 |
| d.Triamcinolone.Placebo | 1.00 |
| d.Triamcinolone.TriesterGlycerol... | 1.00 |
| sd.d | 1.00 |

| | | |
|---|---|---|
| Number of chains | : | 4 |
| Tuning iterations | : | 20,000 |
| Simulation iterations | : | 50,000 |
| Thinning interval | : | 10 |
| Inference samples | : | 10,000 |
| Variance scaling factor: | | 2.5 |

## 2.Inconsistency Model

2.1 Summary estimates

| | | | | | | |
|---|---|---|---|---|---|---|
| Allicin | -14.08 (-95.32, 67.04) | 8.69 (-68.34, 83.56) | -7.54 (-103.78, 85.71) | -14.29 (-107.91, 78.80) | 4.96 (-101.91, 112.38) | 2.87 (-99.44, 108.30) |
| 14.08 (-67.04, 95.32) | Aloe | 23.47 (-37.86, 80.91) | 6.21 (-74.61, 87.21) | -0.01 (-81.41, 83.30) | 18.99 (-68.93, 109.86) | 18.02 (-70.72, 104.76) |
| -8.69 (-83.56, 68.34) | -23.47 (-80.91, 37.86) | Amlexanox | -16.88 (-93.25, 64.40) | -23.08 (-99.07, 55.12) | -3.22 (-96.55, 90.17) | -5.48 (-95.56, 86.51) |
| 7.54 (-85.71, 103.78) | -6.21 (-87.21, 74.61) | 16.88 (-64.40, 93.25) | Berberine gelatin | -6.66 (-103.75, 91.42) | 13.07 (-94.65, 124.66) | 10.82 (-96.06, 117.50) |
| 14.29 (-78.80, 107.91) | 0.01 (-83.30, 81.41) | 23.08 (-55.12, 99.07) | 6.66 (-91.42, 103.75) | Chitosan | 19.50 (-86.09, 128.51) | 17.20 (-92.31, 123.71) |
| -4.96 (-112.38, 101.91) | -18.99 (-109.86, 68.93) | 3.22 (-90.17, 96.55) | -13.07 (-124.66, 94.65) | -19.50 (-128.51, 86.09) | Chlorhexidine | -2.42 (-97.53, 93.75) |
| -2.87 (-108.30, 99.44) | -18.02 (-104.76, 70.72) | 5.48 (-86.51, 95.56) | -10.82 (-117.50, 96.06) | -17.20 (-123.71, 92.31) | 2.42 (-93.75, 97.53) | Curcumin |
| 11.22 (-82.66, 105.09) | -3.75 (-81.34, 79.15) | 19.00 (-55.56, 97.55) | 3.20 (-87.85, 95.53) | -3.32 (-97.43, 92.10) | 15.49 (-88.72, 126.38) | 14.50 (-92.08, 121.24) |
| 6.45 (-85.19, 100.02) | -7.22 (-89.25, 74.27) | 15.61 (-60.47, 90.16) | -1.52 (-93.98, 92.41) | -8.16 (-101.35, 89.24) | 11.40 (-95.64, 120.35) | 10.09 (-96.08, 115.71) |
| 12.63 (-69.71, 95.93) | -1.77 (-67.00, 66.52) | 21.26 (-40.45, 83.25) | 4.33 (-75.25, 86.51) | -1.39 (-84.46, 82.92) | 17.67 (-79.02, 116.32) | 16.05 (-79.37, 116.06) |
| -25.86 (-117.48, 69.58) | -39.59 (-119.66, 42.47) | -17.10 (-92.71, 62.09) | -33.77 (-123.69, 63.61) | -39.79 (-136.03, 56.08) | -20.63 (-125.50, 88.72) | -22.26 (-126.80, 85.81) |
| -28.13 (-122.94, 67.79) | -42.77 (-124.77, 43.57) | -19.77 (-96.31, 59.43) | -36.73 (-130.11, 61.32) | -42.71 (-138.23, 55.65) | -23.50 (-130.94, 87.75) | -24.91 (-130.06, 83.63) |
| -13.98 (-142.04, 112.43) | -28.80 (-139.63, 84.96) | -6.13 (-119.00, 111.24) | -22.88 (-149.38, 106.85) | -28.94 (-156.27, 103.68) | -9.57 (-78.01, 58.26) | -11.94 (-131.88, 111.76) |
| 23.59 (-41.52, 88.34) | 9.39 (-36.64, 56.49) | 32.39 (-5.70, 70.03) | 15.82 (-50.68, 81.70) | 9.30 (-59.04, 78.27) | 28.74 (-56.58, 115.21) | 26.96 (-57.76, 111.75) |
| -1.45 (-84.65, 79.32) | -15.89 (-73.04, 41.06) | 7.20 (-55.62, 67.98) | -9.17 (-93.38, 71.45) | -16.02 (-99.76, 68.50) | 3.28 (-64.23, 72.01) | 1.39 (-64.84, 66.58) |
| 0.50 (-96.59, 100.90) | -13.52 (-89.83, 68.01) | 9.00 (-71.46, 93.91) | -7.18 (-105.74, 91.09) | -14.15 (-112.35, 91.12) | 5.52 (-84.75, 98.34) | 3.34 (-85.78, 96.31) |
| -7.58 (-100.25, 85.88) | -21.89 (-99.98, 59.46) | 1.20 (-74.05, 79.02) | -14.60 (-111.62, 76.55) | -21.64 (-117.49, 75.82) | -2.37 (-111.47, 106.56) | -4.30 (-108.49, 102.70) |

| | | | | | | | | | |
|---|---|---|---|---|---|---|---|---|---|
| -11.22 (-105.09, 82.66) | -6.45 (-100.02, 85.19) | -12.63 (-95.93, 69.71) | 25.86 (-69.58, 117.48) | 28.13 (-67.79, 122.94) | 13.98 (-112.43, 142.04) | -23.59 (-88.34, 41.52) | 1.45 (-79.32, 84.65) | -0.50 (-100.90, 96.59) | 7.58 (-85.88, 100.25) |
| 3.75 (-79.15, 81.34) | 7.22 (-74.27, 89.25) | 1.77 (-66.52, 67.00) | 39.59 (-42.47, 119.66) | 42.77 (-43.57, 124.77) | 28.80 (-84.96, 139.63) | -9.39 (-56.49, 36.64) | 15.89 (-41.06, 73.04) | 13.52 (-68.01, 89.83) | 21.89 (-59.46, 99.98) |
| -19.00 (-97.55, 55.56) | -15.61 (-90.16, 60.47) | -21.26 (-83.25, 40.45) | 17.10 (-62.09, 92.71) | 19.77 (-59.43, 96.31) | 6.13 (-111.24, 119.00) | -32.39 (-70.03, 5.70) | -7.20 (-67.98, 55.62) | -9.00 (-93.91, 71.46) | -1.20 (-79.02, 74.05) |
| -3.20 (-95.53, 87.85) | 1.52 (-92.41, 93.98) | -4.33 (-86.51, 75.25) | 33.77 (-63.61, 123.69) | 36.73 (-61.32, 130.11) | 22.88 (-106.85, 149.38) | -15.82 (-81.70, 50.68) | 9.17 (-71.45, 93.38) | 7.18 (-91.09, 105.74) | 14.60 (-76.55, 111.62) |
| 3.32 (-92.10, 97.43) | 8.16 (-89.24, 101.35) | 1.39 (-82.92, 84.46) | 39.79 (-56.08, 136.03) | 42.71 (-55.65, 138.23) | 28.94 (-103.68, 156.27) | -9.30 (-78.27, 59.04) | 16.02 (-68.50, 99.76) | 14.15 (-91.12, 112.35) | 21.64 (-75.82, 107.49) |
| -15.49 (-126.38, 88.72) | -11.40 (-120.35, 95.64) | -17.67 (-116.32, 79.02) | 20.63 (-88.72, 125.50) | 23.50 (-87.75, 130.94) | 9.57 (-58.26, 78.01) | -28.74 (-115.21, 56.58) | -3.28 (-72.01, 64.23) | -5.52 (-98.34, 84.75) | 2.37 (-106.56, 111.47) |
| -14.50 (-121.24, 92.08) | -10.09 (-115.71, 96.08) | -16.05 (-116.06, 79.37) | 22.26 (-85.81, 126.80) | 24.91 (-83.63, 130.06) | 11.94 (-111.76, 131.88) | -26.96 (-111.75, 57.76) | -1.39 (-66.58, 64.84) | -3.34 (-96.31, 85.78) | 4.30 (-102.70, 108.49) |
| Dexamethasone | 4.42 (-88.79, 98.54) | -1.59 (-83.62, 80.04) | 35.87 (-58.22, 129.90) | 39.48 (-57.54, 134.78) | 25.67 (-101.89, 154.11) | -12.94 (-78.31, 54.69) | 12.55 (-67.61, 95.57) | 10.18 (-89.79, 108.52) | 18.39 (-75.08, 111.54) |
| -4.42 (-98.54, 88.79) | Diosmectite | -6.38 (-86.45, 73.82) | 32.13 (-61.34, 124.16) | 35.22 (-58.85, 127.94) | 21.20 (-107.55, 151.85) | -16.84 (-83.71, 49.34) | 8.52 (-72.55, 91.40) | 5.95 (-93.56, 103.76) | 14.27 (-81.64, 106.77) |
| 1.59 (-80.04, 83.62) | 6.38 (-73.82, 86.45) | Doxycycline | 38.57 (-43.96, 118.96) | 40.71 (-41.87, 124.88) | 27.63 (-95.51, 145.26) | -10.85 (-57.93, 37.99) | 14.19 (-54.04, 83.71) | 12.19 (-76.43, 98.78) | 20.42 (-60.22, 102.60) |
| -35.87 (-129.90, 58.22) | -32.13 (-124.16, 61.34) | -38.57 (-118.96, 43.96) | Insulin-liposomal gel | 2.71 (-91.47, 95.97) | -11.88 (-141.55, 117.83) | -49.20 (-114.29, 19.44) | -23.92 (-105.62, 61.19) | -26.40 (-124.73, 73.36) | 18.36 (-111.32, 76.78) |
| -39.48 (-134.78, 57.54) | -35.22 (-127.94, 58.85) | -40.71 (-124.88, 41.87) | -2.71 (-95.97, 91.47) | Laser | -13.51 (-143.80, 115.83) | -52.02 (-117.86, 17.43) | -26.63 (-109.11, 58.73) | -28.94 (-127.95, 72.96) | 21.17 (-112.61, 75.16) |
| -25.67 (-154.11, 101.89) | -21.20 (-151.85, 107.55) | -27.63 (-145.26, 95.51) | 11.88 (-117.83, 141.55) | 13.51 (-115.83, 143.80) | N-acetylcysteine | -38.46 (-145.73, 71.89) | -12.89 (-109.08, 85.78) | -15.65 (-130.59, 101.52) | 7.72 (-134.12, 122.39) |
| 12.94 (-54.69, 78.31) | 16.84 (-49.34, 83.71) | 10.85 (-37.99, 57.93) | 49.20 (-19.44, 114.29) | 52.02 (-17.43, 117.86) | 38.46 (-71.89, 145.73) | Placebo | 24.18 (-38.05, 86.74) | 21.75 (-57.98, 101.60) | 31.52 (-35.42, 97.32) |
| -12.55 (-95.57, 67.61) | -8.52 (-91.40, 72.55) | -14.19 (-83.71, 54.04) | 23.92 (-61.19, 105.62) | 26.63 (-58.73, 109.11) | 12.89 (-85.78, 109.08) | -24.18 (-86.74, 38.05) | Triamcinolone | -1.68 (-66.71, 57.89) | 5.64 (-75.69, 85.22) |
| -10.18 (-108.52, 89.79) | -5.95 (-103.76, 93.56) | -12.19 (-98.78, 76.43) | 26.40 (-73.36, 124.73) | 28.94 (-72.96, 127.95) | 15.65 (-101.52, 130.59) | -21.75 (-101.60, 57.98) | 1.68 (-57.89, 66.71) | Triester Glycerol Oxide | 8.37 (-90.99, 106.09) |
| -18.39 (-111.54, 75.08) | -14.27 (-106.77, 81.64) | -20.42 (-102.60, 60.22) | 18.36 (-76.78, 111.32) | 21.17 (-75.16, 112.61) | 7.72 (-122.39, 134.12) | -31.52 (-97.32, 35.42) | -5.64 (-85.22, 75.69) | -8.37 (-106.09, 90.99) | Zinc |

2.2 Inconsistency Factors

| Cycle | Median (95% CI) |
|---|---|
| Aloe, Placebo, Triamcinolone | -0.38 (-58.99, 54.34) |
| Aloe, Placebo, Triamcinolone | -0.38 (-58.99, 54.34) |

2.3 Variance Calculation

| Parameter | Median (95% CI) |
|---|---|
| Random Effects Standard Deviation | 28.06 (15.11, 58.19) |
| Inconsistency Standard Deviation | 24.41 (1.30, 64.80) |

## 2.4 Convergence Diagnostics

| Parameter | PSRF |
|---|---|
| d.Allicin.Placebo | 1.00 |
| d.Aloe.Triamcinolone | 1.00 |
| d.Chlorhexidine.Nacetylcysteine | 1.00 |
| d.Placebo.Aloe | 1.00 |
| d.Placebo.Amlexanox | 1.00 |
| d.Placebo.Berberinegelatin | 1.00 |
| d.Placebo.Chitosan | 1.00 |
| d.Placebo.Dexamethasone | 1.00 |
| d.Placebo.Diosmectite | 1.00 |
| d.Placebo.Doxycycline | 1.00 |
| d.Placebo.Insulinliposomalgel | 1.00 |
| d.Placebo.Laser | 1.00 |
| d.Placebo.Zinc | 1.01 |
| d.Triamcinolone.Chlorhexidine | 1.00 |
| d.Triamcinolone.Curcumin | 1.00 |
| d.Triamcinolone.TriesterGlycerol... | 1.00 |
| w.Aloe.Placebo.Triamcinolone | 1.00 |
| w.Aloe.Placebo.Triamcinolone | 1.00 |
| sd.d | 1.00 |
| sd.w | 1.00 |

Number of chains : 4
Tuning iterations : 20,000
Simulation iterations : 50,000
Thinning interval : 10
Inference samples : 10,000
Variance scaling factor: 2.5

## 3. Node-splitting analysis

| Name | Direct Effect | Indirect Effect | Overall | P-Value |
|---|---|---|---|---|
| Aloe, Triamcinolone | 18.64 (-51.20, 89.82) | 15.88 (-56.56, 89.69) | 16.41 (-32.38, 65.70) | 0.94 |

## 4. Network structure

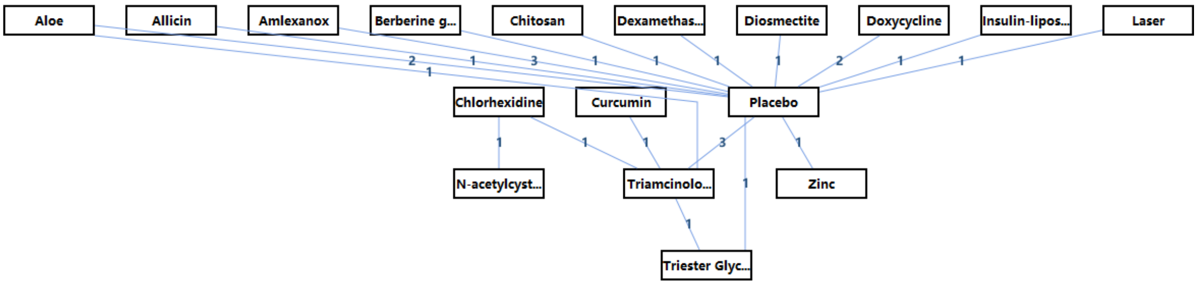

**Day 7**

## 1. Consistency Model

| | | | | | | | | | | | |
|---|---|---|---|---|---|---|---|---|---|---|---|
| Aloe | 8.28 (-29.91, 47.09) | 3.27 (-34.44, 41.10) | 11.63 (-14.23, 39.64) | 8.42 (-22.49, 41.28) | 4.91 (-22.35, 31.30) | 10.31 (-13.72, 32.75) | -4.44 (-24.12, 17.39) | 21.94 (-7.04, 53.69) | 9.44 (-35.79, 52.40) | 8.16 (-13.71, 28.52) | 5.53 (-27.75, 40.21) |
| -8.28 (-47.09, 29.91) | Amlexanox | -5.77 (-53.42, 42.30) | 3.40 (-36.37, 44.23) | -0.05 (-20.69, 22.41) | -3.30 (-40.40, 31.63) | 1.89 (-35.24, 36.01) | -12.73 (-43.87, 19.53) | 13.56 (-23.55, 53.30) | 0.59 (-53.02, 53.15) | -0.07 (-37.96, 35.16) | -3.00 (-43.99, 39.13) |
| -3.27 (-41.10, 34.44) | 5.77 (-42.30, 53.42) | Chlorhexidine | 8.66 (-26.42, 45.14) | 5.64 (-36.60, 50.04) | 1.90 (-37.99, 40.35) | 6.88 (-27.84, 40.74) | -7.29 (-42.37, 28.76) | 18.81 (-21.20, 62.36) | 6.14 (-15.99, 28.40) | 4.88 (-27.16, 35.94) | 2.78 (-41.64, 48.22) |
| -11.63 (-39.64, 14.23) | -3.40 (-44.23, 36.37) | -8.66 (-45.14, 26.42) | Curcumin | -3.42 (-37.76, 30.93) | -6.80 (-36.04, 20.42) | -1.43 (-25.33, 19.24) | -16.05 (-39.17, 7.77) | 10.17 (-21.23, 44.08) | -2.59 (-45.24, 39.06) | -3.51 (-21.80, 11.68) | -6.29 (-40.77, 29.25) |
| -8.42 (-41.28, 22.49) | 0.05 (-22.41, 20.69) | -5.64 (-50.04, 36.60) | 3.42 (-30.93, 37.76) | Dexamethasone | -3.49 (-34.40, 23.95) | 1.76 (-28.88, 28.24) | -12.74 (-36.19, 11.15) | 13.31 (-19.18, 47.12) | 0.42 (-49.75, 49.38) | -0.26 (-31.61, 27.63) | -3.05 (-37.44, 33.26) |
| -4.91 (-31.30, 22.35) | 3.30 (-31.63, 40.40) | -1.90 (-40.35, 37.99) | 6.80 (-20.42, 36.04) | 3.49 (-23.95, 34.40) | Doxycycline | 5.33 (-18.04, 27.48) | -9.23 (-24.43, 9.32) | 16.97 (-9.50, 47.52) | 4.33 (-41.47, 50.55) | 3.30 (-20.56, 26.16) | 0.84 (-28.70, 34.47) |
| -10.31 (-32.75, 13.72) | -1.89 (-36.01, 35.24) | -6.88 (-40.74, 27.84) | 1.43 (-19.24, 25.33) | -1.76 (-28.24, 28.88) | -5.33 (-27.48, 18.04) | Laser | -14.63 (-28.41, 3.42) | 11.53 (-13.52, 42.18) | -0.99 (-41.39, 40.23) | -2.08 (-15.95, 12.27) | -4.22 (-33.75, 28.28) |
| 4.44 (-17.39, 24.12) | 12.73 (-19.53, 43.87) | 7.29 (-28.76, 42.37) | 16.05 (-7.77, 39.17) | 12.74 (-11.15, 36.19) | 9.23 (-9.32, 24.43) | 14.63 (-3.42, 28.41) | Placebo | 26.25 (4.00, 49.84) | 13.50 (-30.51, 54.46) | 12.56 (-6.58, 27.34) | 9.74 (-16.41, 37.01) |
| -21.94 (-53.69, 7.04) | -13.56 (-53.30, 23.55) | -18.81 (-62.36, 21.20) | -10.17 (-44.08, 21.23) | -13.31 (-47.12, 19.18) | -16.97 (-47.52, 9.50) | -11.53 (-42.18, 13.52) | -26.25 (-49.84, -4.00) | Silver nitrate | -12.79 (-63.70, 33.39) | -13.61 (-44.71, 12.11) | -16.50 (-52.07, 18.61) |
| -9.44 (-52.40, 35.79) | -0.59 (-53.15, 53.02) | -6.14 (-28.40, 15.99) | 2.59 (-39.06, 45.24) | -0.42 (-49.38, 49.75) | -4.33 (-50.55, 41.47) | 0.99 (-40.23, 41.39) | -13.50 (-54.46, 30.51) | 12.79 (-33.39, 63.70) | Sucralfate | -1.06 (-39.83, 37.01) | -3.52 (-53.57, 47.73) |
| -8.16 (-28.52, 13.71) | 0.07 (-35.16, 37.96) | -4.88 (-35.94, 27.16) | 3.51 (-11.68, 21.80) | 0.26 (-27.63, 31.61) | -3.30 (-26.16, 20.56) | 2.08 (-12.27, 15.95) | -12.56 (-27.34, 6.58) | 13.61 (-12.11, 44.71) | 1.06 (-37.01, 39.83) | Triamcinolone | -2.79 (-31.72, 30.24) |
| -5.53 (-40.21, 27.75) | 3.00 (-39.13, 43.99) | -2.78 (-48.22, 41.64) | 6.29 (-29.25, 40.77) | 3.05 (-33.26, 37.44) | -0.84 (-34.47, 28.70) | 4.22 (-28.28, 33.75) | -9.74 (-37.01, 16.41) | 16.50 (-18.61, 52.07) | 3.52 (-47.73, 53.57) | 2.79 (-30.24, 31.72) | Zinc |

## 1.2 Rank probability(Rank 1 is best, rank N is worst)

| Drug | Rank 1 | Rank 2 | Rank 3 | Rank 4 | Rank 5 | Rank 6 | Rank 7 | Rank 8 | Rank 9 | Rank 10 | Rank 11 | Rank 12 |
|---|---|---|---|---|---|---|---|---|---|---|---|---|
| Aloe | 0.01 | 0.01 | 0.02 | 0.03 | 0.04 | 0.06 | 0.08 | 0.12 | 0.15 | 0.18 | 0.16 | 0.14 |
| Amlexanox | 0.09 | 0.11 | 0.1 | 0.08 | 0.08 | 0.07 | 0.08 | 0.08 | 0.08 | 0.08 | 0.06 | 0.08 |
| Chlorhexidine | 0.02 | 0.09 | 0.08 | 0.06 | 0.06 | 0.07 | 0.07 | 0.07 | 0.08 | 0.09 | 0.13 | 0.19 |
| Curcumin | 0.08 | 0.14 | 0.14 | 0.14 | 0.12 | 0.11 | 0.09 | 0.07 | 0.05 | 0.04 | 0.02 | 0.01 |
| Dexamethasone | 0.03 | 0.1 | 0.12 | 0.11 | 0.1 | 0.09 | 0.1 | 0.1 | 0.09 | 0.07 | 0.05 | 0.03 |
| Doxycycline | 0.01 | 0.04 | 0.06 | 0.08 | 0.09 | 0.1 | 0.12 | 0.14 | 0.12 | 0.11 | 0.07 | 0.04 |
| Laser | 0.02 | 0.09 | 0.12 | 0.17 | 0.15 | 0.14 | 0.12 | 0.09 | 0.05 | 0.03 | 0.02 | 0.01 |
| Placebo | 0 | 0 | 0 | 0 | 0.01 | 0.02 | 0.04 | 0.07 | 0.13 | 0.2 | 0.26 | 0.28 |
| Silver nitrate | 0.48 | 0.18 | 0.13 | 0.07 | 0.05 | 0.03 | 0.02 | 0.02 | 0.01 | 0.01 | 0.01 | 0 |
| Sucralfate | 0.18 | 0.12 | 0.08 | 0.07 | 0.06 | 0.06 | 0.06 | 0.06 | 0.06 | 0.07 | 0.11 | 0.09 |
| Triamcinolone | 0 | 0.02 | 0.07 | 0.12 | 0.18 | 0.19 | 0.16 | 0.12 | 0.08 | 0.04 | 0.02 | 0 |
| Zinc | 0.07 | 0.1 | 0.08 | 0.07 | 0.07 | 0.06 | 0.07 | 0.08 | 0.08 | 0.09 | 0.1 | 0.13 |

## 1.3 Consistency check

| Parameter | Median (95% CI) |
|---|---|
| Random Effects Standard Deviation | 8.57 (3.03, 21.72) |

## 1.4 Convergence Diagnostics

| Parameter | PSRF |
|---|---|
| d.Chlorhexidine.Sucralfate | 1.00 |
| d.Dexamethasone.Amlexanox | 1.00 |
| d.Placebo.Aloe | 1.00 |
| d.Placebo.Dexamethasone | 1.00 |
| d.Placebo.Doxycycline | 1.00 |
| d.Placebo.Laser | 1.01 |
| d.Placebo.Silvernitrate | 1.00 |
| d.Placebo.Triamcinolone | 1.01 |
| d.Placebo.Zinc | 1.59 |
| d.Triamcinolone.Chlorhexidine | 1.00 |
| d.Triamcinolone.Curcumin | 1.00 |
| sd.d | 1.01 |

Number of chains : 4

Tuning iterations : 20,000

Simulation iterations : 50,000

Thinning interval : 10

Inference samples : 10,000

Variance scaling factor: 2.5

## 2.Inconsistency Model

### 2.1 Summary estimates

| | | | | | | | | | | | |
|---|---|---|---|---|---|---|---|---|---|---|---|
| Aloe | 7.22 (-35.81, 52.84) | -2.18 (-44.89, 42.62) | 5.35 (-26.17, 42.91) | 7.29 (-29.01, 45.57) | 2.68 (-36.77, 27.63) | 6.79 (-21.79, 35.22) | -5.79 (-28.86, 20.75) | 20.61 (-14.82, 58.39) | 4.19 (-47.24, 56.79) | 9.62 (-17.50, 32.86) | 6.17 (-35.93, 39.95) |
| -7.22 (-52.84, 35.81) | Amlexanox | -9.22 (-64.91, 44.78) | -2.22 (-46.74, 48.16) | -0.00 (-25.89, 26.29) | -4.87 (-54.04, 29.90) | -0.60 (-42.81, 39.92) | -12.84 (-49.54, 24.41) | 13.38 (-33.09, 59.79) | -3.14 (-63.84, 57.81) | -5.34 (-48.39, 38.44) | -0.51 (-53.40, 40.25) |
| 2.18 (-42.62, 44.89) | 9.22 (-44.78, 64.91) | Chlorhexidine | 8.10 (-29.11, 49.08) | 9.42 (-38.15, 57.97) | 2.91 (-47.50, 43.54) | 8.67 (-29.64, 45.45) | -3.12 (-42.98, 36.53) | 23.20 (-23.50, 71.45) | 6.26 (-19.82, 32.20) | 4.40 (-28.58, 39.02) | 8.61 (-44.68, 53.66) |
| -5.35 (-42.91, 26.17) | 2.22 (-48.16, 46.74) | -8.10 (-49.08, 29.11) | Curcumin | 2.04 (-39.40, 38.91) | -3.38 (-49.24, 24.64) | 1.88 (-27.16, 22.35) | -10.90 (-42.26, 16.17) | 15.61 (-26.19, 51.53) | -1.66 (-50.95, 43.38) | -2.93 (-24.12, 13.87) | 1.42 (-47.93, 35.10) |
| -7.29 (-45.57, 29.01) | 0.00 (-26.29, 25.89) | -9.42 (-57.97, 38.15) | -2.04 (-38.91, 39.40) | Dexamethasone | -5.25 (-46.22, 22.84) | -0.67 (-35.26, 31.39) | -12.79 (-40.44, 14.81) | 13.32 (-25.03, 51.20) | -3.17 (-58.23, 52.03) | -5.30 (-40.65, 30.00) | -0.50 (-46.96, 33.59) |
| -2.68 (-27.63, 36.77) | 4.87 (-29.90, 54.04) | -2.91 (-43.54, 47.50) | 3.38 (-24.64, 49.24) | 5.25 (-22.84, 46.22) | Doxycycline | 4.66 (-17.93, 38.89) | -8.18 (-22.16, 21.23) | 17.99 (-7.98, 60.09) | 2.97 (-43.71, 61.25) | 0.35 (-24.06, 37.80) | 5.18 (-25.52, 38.90) |
| -6.79 (-35.22, 21.79) | 0.60 (-39.92, 42.81) | -8.67 (-45.45, 29.64) | -1.88 (-22.35, 27.16) | 0.67 (-31.39, 35.26) | -4.66 (-38.89, 17.93) | Laser | -12.00 (-29.93, 7.52) | 14.34 (-17.53, 47.33) | -2.57 (-47.88, 43.83) | -4.69 (-20.15, 13.56) | -0.08 (-39.34, 30.35) |
| 5.79 (-20.75, 28.86) | 12.84 (-24.41, 49.54) | 3.12 (-36.53, 42.98) | 10.90 (-16.17, 42.26) | 12.79 (-14.81, 40.44) | 8.18 (-21.23, 22.16) | 12.00 (-7.52, 29.93) | Placebo | 26.27 (-0.56, 52.70) | 9.67 (-38.34, 57.39) | 1.90 (-36.27, 37.25) | 11.53 (-23.02, 35.05) |
| -20.61 (-58.39, 14.82) | -13.38 (-59.79, 33.09) | -23.20 (-71.45, 23.50) | -15.61 (-51.53, 26.19) | -13.32 (-51.20, 25.03) | -17.99 (-60.09, 7.98) | -14.34 (-47.33, 17.53) | -26.27 (-52.70, 0.56) | Silver nitrate | -16.68 (-71.62, 37.56) | -18.68 (-52.96, 16.61) | -14.11 (-60.49, 19.06) |
| -4.19 (-56.79, 47.24) | 3.14 (-57.81, 63.84) | -6.26 (-32.20, 19.82) | 1.66 (-43.38, 50.95) | 3.17 (-52.03, 58.23) | -2.97 (-61.25, 43.71) | 2.57 (-43.83, 47.88) | -9.67 (-57.39, 38.34) | 16.68 (-37.56, 71.62) | Sucralfate | -1.84 (-44.42, 41.42) | 2.42 (-58.09, 54.27) |
| -9.62 (-32.86, 17.50) | 5.34 (-38.44, 48.39) | -4.40 (-39.02, 28.58) | 2.93 (-13.87, 24.12) | 5.30 (-30.00, 40.65) | -0.35 (-37.80, 24.06) | 4.69 (-13.56, 20.15) | -1.90 (-37.25, 36.27) | 18.68 (-16.61, 52.96) | 1.84 (-41.42, 44.42) | Triamcinolone | 4.30 (-38.46, 35.32) |
| -6.17 (-39.95, 35.93) | 0.51 (-40.25, 53.40) | -8.61 (-53.66, 44.68) | -1.42 (-35.10, 47.93) | 0.50 (-33.59, 46.96) | -5.18 (-38.90, 25.52) | 0.08 (-30.35, 39.34) | -11.53 (-35.05, 23.02) | 14.11 (-19.06, 60.49) | -2.42 (-54.27, 58.09) | -4.30 (-35.32, 38.46) | Zinc |

2.2 Inconsistency Factors

| Cycle | Median (95% CI) |
|---|---|
| Laser, Placebo, Triamcinolone | -5.32 (-28.08, 12.93) |

2.3 Variance Calculation

| Parameter | Median (95% CI) |
|---|---|
| Random Effects Standard Deviation | 9.36 (1.27, 23.84) |
| Inconsistency Standard Deviation | 12.65 (0.78, 25.43) |

2.4 Convergence Diagnostics

| Parameter | PSRF |
|---|---|
| d.Aloe.Placebo | 1.08 |
| d.Chlorhexidine.Sucralfate | 1.07 |
| d.Dexamethasone.Amlexanox | 1.08 |
| d.Laser.Triamcinolone | 1.07 |
| d.Placebo.Dexamethasone | 1.06 |
| d.Placebo.Doxycycline | 1.60 |
| d.Placebo.Laser | 1.04 |
| d.Placebo.Silvernitrate | 1.08 |
| d.Placebo.Zinc | 1.46 |
| d.Triamcinolone.Chlorhexidine | 1.02 |
| d.Triamcinolone.Curcumin | 1.08 |
| w.Laser.Placebo.Triamcinolone | 1.04 |
| sd.d | 2.00 |
| sd.w | 1.00 |

Number of chains : 4
Tuning iterations : 20,000
Simulation iterations : 50,000
Thinning interval : 10
Inference samples : 10,000
Variance scaling factor: 2.5

**3. Node-splitting analysis**

| Name | Direct Effect | Indirect Effect | Overall | P-Value |
|---|---|---|---|---|
| Laser, Placebo | -9.19 (-26.50, 11.28) | -23.95 (-49.70, 4.00) | -14.63 (-28.41, 3.42) | 0.24 |
| Laser, Triamcinolone | -6.77 (-25.31, 12.97) | 9.13 (-21.39, 43.63) | -2.08 (-15.95, 12.27) | 0.31 |
| Placebo, Triamcinolone | 17.68 (-4.24, 40.87) | 2.27 (-24.74, 26.04) | 12.56 (-6.58, 27.34) | 0.23 |

**4. Network structure**

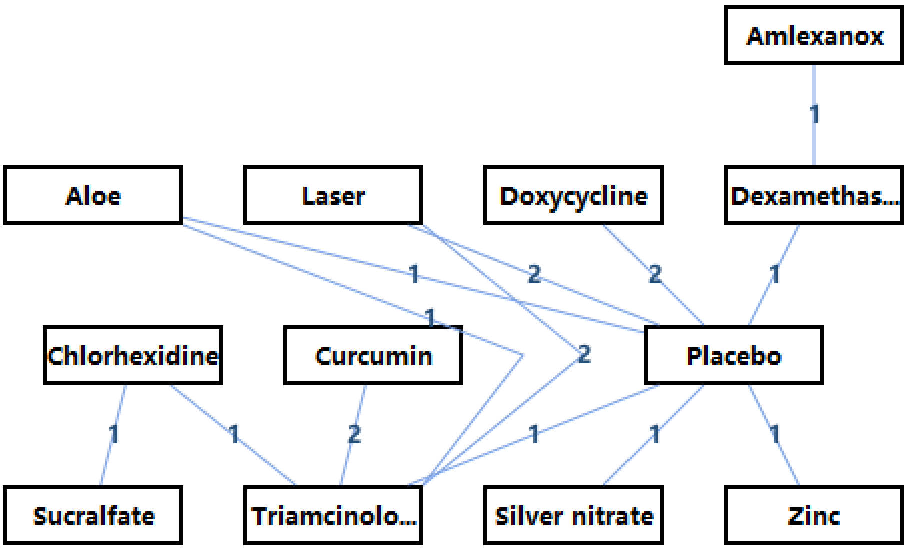

**Day 8**

## 1. Consistency Model

### 1.1 Summary estimates

| | | | | | |
|---|---|---|---|---|---|
| Benzydamine | -12.99 (-32.92, 12.62) | 13.14 (-2.11, 28.07) | -0.11 (-10.10, 10.43) | -0.24 (-10.39, 10.39) | -11.47 (-22.19, 0.55) |
| 12.99 (-12.62, 32.92) | Chlorhexidine | 25.39 (-1.50, 48.30) | 13.02 (-11.37, 31.84) | 12.83 (-11.63, 31.84) | 1.88 (-19.09, 17.00) |
| -13.14 (-28.07, 2.11) | -25.39 (-48.30, 1.50) | Dexamethasone | -12.86 (-24.79, -0.78) | -12.95 (-24.20, -1.97) | -24.18 (-38.72, -9.63) |
| 0.11 (-10.43, 10.10) | -13.02 (-31.84, 11.37) | 12.86 (0.78, 24.79) | Doxycycline | -0.09 (-5.05, 4.62) | -11.12 (-20.78, -1.93) |
| 0.24 (-10.39, 10.39) | -12.83 (-31.84, 11.63) | 12.95 (1.97, 24.20) | 0.09 (-4.62, 5.05) | Placebo | -11.10 (-20.96, -1.34) |
| 11.47 (-0.55, 22.19) | -1.88 (-17.00, 19.09) | 24.18 (9.63, 38.72) | 11.12 (1.93, 20.78) | 11.10 (1.34, 20.96) | Triamcinolone |

### 1.2 Rank probability(Rank 1 is best, rank N is worst)

| Drug | Rank 1 | Rank 2 | Rank 3 | Rank 4 | Rank 5 | Rank 6 |
|---|---|---|---|---|---|---|
| Benzydamine | 0.03 | 0.37 | 0.15 | 0.34 | 0.1 | 0.02 |
| Chlorhexidine | 0.03 | 0.14 | 0.05 | 0.03 | 0.21 | 0.54 |
| Dexamethasone | 0.93 | 0.05 | 0.01 | 0.01 | 0 | 0 |
| Doxycycline | 0.01 | 0.23 | 0.4 | 0.29 | 0.07 | 0 |
| Placebo | 0 | 0.21 | 0.39 | 0.31 | 0.08 | 0.01 |
| Triamcinolone | 0 | 0 | 0.01 | 0.02 | 0.54 | 0.43 |

### 1.3 Consistency check

| Parameter | Median (95% CI) |
|---|---|
| Random Effects Standard Deviation | 1.56 (0.10, 10.03) |

### 1.4 Convergence Diagnostics

| Parameter | PSRF |
|---|---|
| d.Placebo.Benzydamine | 1.00 |
| d.Placebo.Dexamethasone | 1.01 |
| d.Placebo.Doxycycline | 1.00 |
| d.Placebo.Triamcinolone | 1.00 |
| d.Triamcinolone.Chlorhexidine | 4.64 |
| sd.d | 1.01 |

Number of chains : 4
Tuning iterations : 20,000
Simulation iterations : 50,000
Thinning interval : 10
Inference samples : 10,000
Variance scaling factor: 2.5

## 2.Inconsistency Model

### 2.1 Summary estimates

| | | | | | |
|---|---|---|---|---|---|
| Benzydamine | -9.51 (-30.86, 13.71) | 12.49 (-4.10, 30.96) | 0.51 (-10.65, 12.45) | -0.52 (-11.49, 11.30) | -10.30 (-23.01, 1.17) |
| 9.51 (-13.71, 30.86) | Chlorhexidine | 22.44 (-3.96, 46.40) | 10.50 (-12.93, 30.73) | 9.72 (-13.84, 30.26) | -0.85 (-21.51, 16.68) |
| -12.49 (-30.96, 4.10) | -22.44 (-46.40, 3.96) | Dexamethasone | -11.98 (-27.24, 1.26) | -12.74 (-26.59, -0.65) | -22.92 (-42.43, -7.14) |
| -0.51 (-12.45, 10.65) | -10.50 (-30.73, 12.93) | 11.98 (-1.26, 27.24) | Doxycycline | -0.73 (-6.76, 4.72) | -11.08 (-22.32, -0.92) |
| 0.52 (-11.30, 11.49) | -9.72 (-30.26, 13.84) | 12.74 (0.65, 26.59) | 0.73 (-4.72, 6.76) | Placebo | -10.24 (-21.33, 0.32) |
| 10.30 (-1.17, 23.01) | 0.85 (-16.68, 21.51) | 22.92 (7.14, 42.43) | 11.08 (0.92, 22.32) | 10.24 (-0.32, 21.33) | Triamcinolone |

### 2.2 Variance Calculation

| Parameter | Median (95% CI) |
|---|---|
| Random Effects Standard Deviation | 2.06 (0.09, 10.83) |
| Inconsistency Standard Deviation | 6.48 (0.26, 12.58) |

## 2.3 Convergence Diagnostics

| Parameter | PSRF |
|---|---|
| d.Benzydamine.Doxycycline | 1.02 |
| d.Doxycycline.Placebo | 1.11 |
| d.Placebo.Dexamethasone | 1.01 |
| d.Placebo.Triamcinolone | 1.01 |
| d.Triamcinolone.Chlorhexidine | 1.00 |
| sd.d | 1.02 |
| sd.w | 1.00 |

Number of chains : 4
Tuning iterations : 20,000
Simulation iterations : 50,000
Thinning interval : 10
Inference samples : 10,000
Variance scaling factor: 2.5

## 3. Node-splitting analysis

| Name | Direct Effect | Indirect Effect | Overall | P-Value |
|---|---|---|---|---|
| Doxycycline, Triamcinolone | -10.89 (-22.76, -0.56) | -0.64 (-383.98, 380.63) | -11.12 (-20.78, -1.93) | 0.96 |
| Placebo, Triamcinolone | -11.15 (-22.38, 0.27) | 1.43 (-381.59, 374.20) | -11.10 (-20.96, -1.34) | 0.95 |

## 4. Network structure

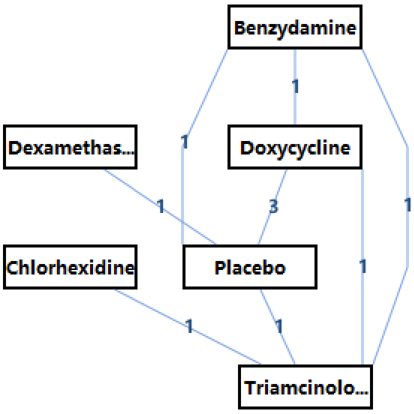


# Day 10

## 1. Consistency Model

### 1.1 Summary estimates

| | | | | | | |
|---|---|---|---|---|---|---|
| Benzydamine | -0.13 (-24.02, 23.63) | 8.58 (-20.45, 36.99) | -3.88 (-21.34, 10.69) | -7.56 (-34.90, 13.44) | -7.97 (-27.63, 6.47) | -0.02 (-16.65, 16.99) |
| 0.13 (-23.63, 24.02) | Chlorhexidine | 8.63 (-21.61, 37.92) | -3.80 (-27.45, 17.84) | -7.44 (-39.68, 18.42) | -7.87 (-33.97, 13.21) | 0.09 (-16.85, 17.08) |
| -8.58 (-36.99, 20.45) | -8.63 (-37.92, 21.61) | Curcumin | -12.66 (-41.78, 15.00) | -16.80 (-51.89, 14.80) | -16.97 (-47.99, 10.54) | -8.55 (-31.87, 15.78) |
| 3.88 (-10.69, 21.34) | 3.80 (-17.84, 27.45) | 12.66 (-15.00, 41.78) | Doxycycline | -3.64 (-25.14, 14.67) | -3.91 (-15.61, 5.00) | 3.97 (-10.57, 21.29) |
| 7.56 (-13.44, 34.90) | 7.44 (-18.42, 39.68) | 16.80 (-14.80, 51.89) | 3.64 (-14.67, 25.14) | Laser | -0.13 (-16.70, 16.64) | 7.58 (-12.92, 34.99) |
| 7.97 (-6.47, 27.63) | 7.87 (-13.21, 33.97) | 16.97 (-10.54, 47.99) | 3.91 (-5.00, 15.61) | 0.13 (-16.64, 16.70) | Placebo | 8.09 (-6.64, 27.60) |
| 0.02 (-16.99, 16.65) | -0.09 (-17.08, 16.85) | 8.55 (-15.78, 31.87) | -3.97 (-21.29, 10.57) | -7.58 (-34.99, 12.92) | -8.09 (-27.60, 6.64) | Triamcinolone |

### 1.2 Rank probability(Rank 1 is best, rank N is worst)

| Drug | Rank 1 | Rank 2 | Rank 3 | Rank 4 | Rank 5 | Rank 6 | Rank 7 |
|---|---|---|---|---|---|---|---|
| Benzydamine | 0.14 | 0.23 | 0.2 | 0.21 | 0.11 | 0.07 | 0.05 |
| Chlorhexidine | 0.14 | 0.24 | 0.17 | 0.14 | 0.11 | 0.09 | 0.11 |
| Curcumin | 0.6 | 0.12 | 0.08 | 0.06 | 0.04 | 0.04 | 0.06 |
| Doxycycline | 0.03 | 0.07 | 0.13 | 0.22 | 0.33 | 0.16 | 0.06 |
| Laser | 0.04 | 0.07 | 0.07 | 0.09 | 0.14 | 0.19 | 0.4 |
| Placebo | 0 | 0.02 | 0.04 | 0.08 | 0.16 | 0.4 | 0.3 |
| Triamcinolone | 0.05 | 0.24 | 0.33 | 0.2 | 0.1 | 0.06 | 0.02 |

1.3 Consistency check

| Parameter | Median (95% CI) |
|---|---|
| Random Effects Standard Deviation | 8.08 (2.39, 11.75) |

1.4 Convergence Diagnostics

| Parameter | PSRF |
|---|---|
| d.Placebo.Benzydamine | 1.29 |
| d.Placebo.Doxycycline | 1.21 |
| d.Placebo.Laser | 1.02 |
| d.Placebo.Triamcinolone | 1.30 |
| d.Triamcinolone.Chlorhexidine | 1.02 |
| d.Triamcinolone.Curcumin | 1.01 |
| sd.d | 1.51 |

Number of chains : 4
Tuning iterations : 20,000
Simulation iterations : 50,000
Thinning interval : 10
Inference samples : 10,000
Variance scaling factor: 2.5

## 2.Inconsistency Model

2.1 Summary estimates

| | | | | | | |
|---|---|---|---|---|---|---|
| Benzydamine | 0.12 (-22.25, 22.64) | 8.86 (-19.00, 36.44) | -3.23 (-18.42, 10.84) | -6.51 (-28.92, 14.31) | -6.50 (-21.76, 7.57) | -0.10 (-15.59, 15.98) |
| -0.12 (-22.64, 22.25) | Chlorhexidine | 8.66 (-18.53, 35.77) | -3.35 (-25.29, 18.10) | -6.55 (-34.20, 19.66) | -6.58 (-29.19, 14.60) | -0.05 (-15.86, 15.76) |
| -8.86 (-36.44, 19.00) | -8.66 (-35.77, 18.53) | Curcumin | -12.08 (-39.38, 14.82) | -15.60 (-46.80, 15.51) | -15.40 (-42.92, 11.22) | -8.75 (-31.44, 14.23) |
| 3.23 (-10.84, 18.42) | 3.35 (-18.10, 25.29) | 12.08 (-14.82, 39.38) | Doxycycline | -3.25 (-21.62, 14.38) | -3.17 (-12.83, 5.82) | 3.23 (-11.22, 18.76) |
| 6.51 (-14.31, 28.92) | 6.55 (-19.66, 34.20) | 15.60 (-15.51, 46.80) | 3.25 (-14.38, 21.62) | Laser | 0.16 (-15.89, 15.61) | 6.64 (-14.49, 28.81) |
| 6.50 (-7.57, 21.76) | 6.58 (-14.60, 29.19) | 15.40 (-11.22, 42.92) | 3.17 (-5.82, 12.83) | -0.16 (-15.61, 15.89) | Placebo | 6.50 (-7.74, 22.38) |
| 0.10 (-15.98, 15.59) | 0.05 (-15.76, 15.86) | 8.75 (-14.23, 31.44) | -3.23 (-18.76, 11.22) | -6.64 (-28.81, 14.49) | -6.50 (-22.38, 7.74) | Triamcinolone |

2.2 Variance Calculation

| Parameter | Median (95% CI) |
|---|---|
| Random Effects Standard Deviation | 7.15 (2.76, 11.57) |
| Inconsistency Standard Deviation | 6.08 (0.30, 11.66) |

2.3 Convergence Diagnostics

| Parameter | PSRF |
|---|---|
| d.Benzydamine.Doxycycline | 1.00 |
| d.Doxycycline.Placebo | 1.00 |
| d.Placebo.Laser | 1.00 |
| d.Placebo.Triamcinolone | 1.00 |
| d.Triamcinolone.Chlorhexidine | 1.00 |
| d.Triamcinolone.Curcumin | 1.00 |
| sd.d | 1.00 |
| sd.w | 1.00 |

Number of chains : 4
Tuning iterations : 20,000
Simulation iterations : 50,000
Thinning interval : 10
Inference samples : 10,000
Variance scaling factor: 2.5

## 3. Node-splitting analysis

| Name | Direct Effect | Indirect Effect | Overall | P-Value |
|---|---|---|---|---|
| Doxycycline, Triamcinolone | 0.07 (-16.35, 16.57) | -3.87 (-355.67, 356.11) | 3.97 (-10.57, 21.29) | 0.98 |
| Placebo, Triamcinolone | 9.71 (-4.44, 28.47) | 8.71 (-337.74, 358.78) | 8.09 (-6.64, 27.60) | 1 |

## 4. Network structure

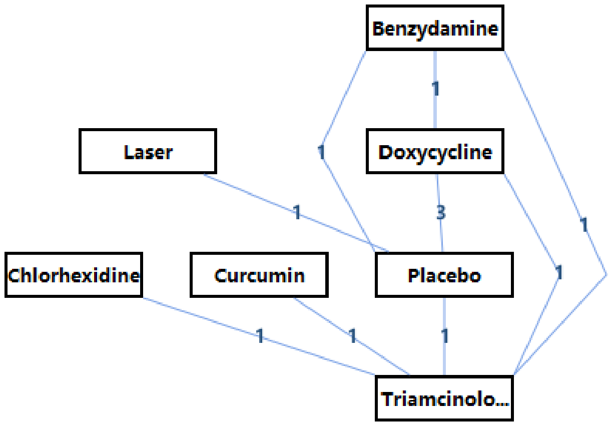


### Chart S1 "Time-Rank 1 probability" folding line chart

In order to clearly demonstrate the effect of the different local interventions on ulcer size reduction and symptom reduction at the various stages of the trial, we plot line graphs on time-rank 1 probability. The solid points represent the real results obtained and the hollow points are only dummy values introduced to represent the trend in the effect of the local intervention according to a linear equation.

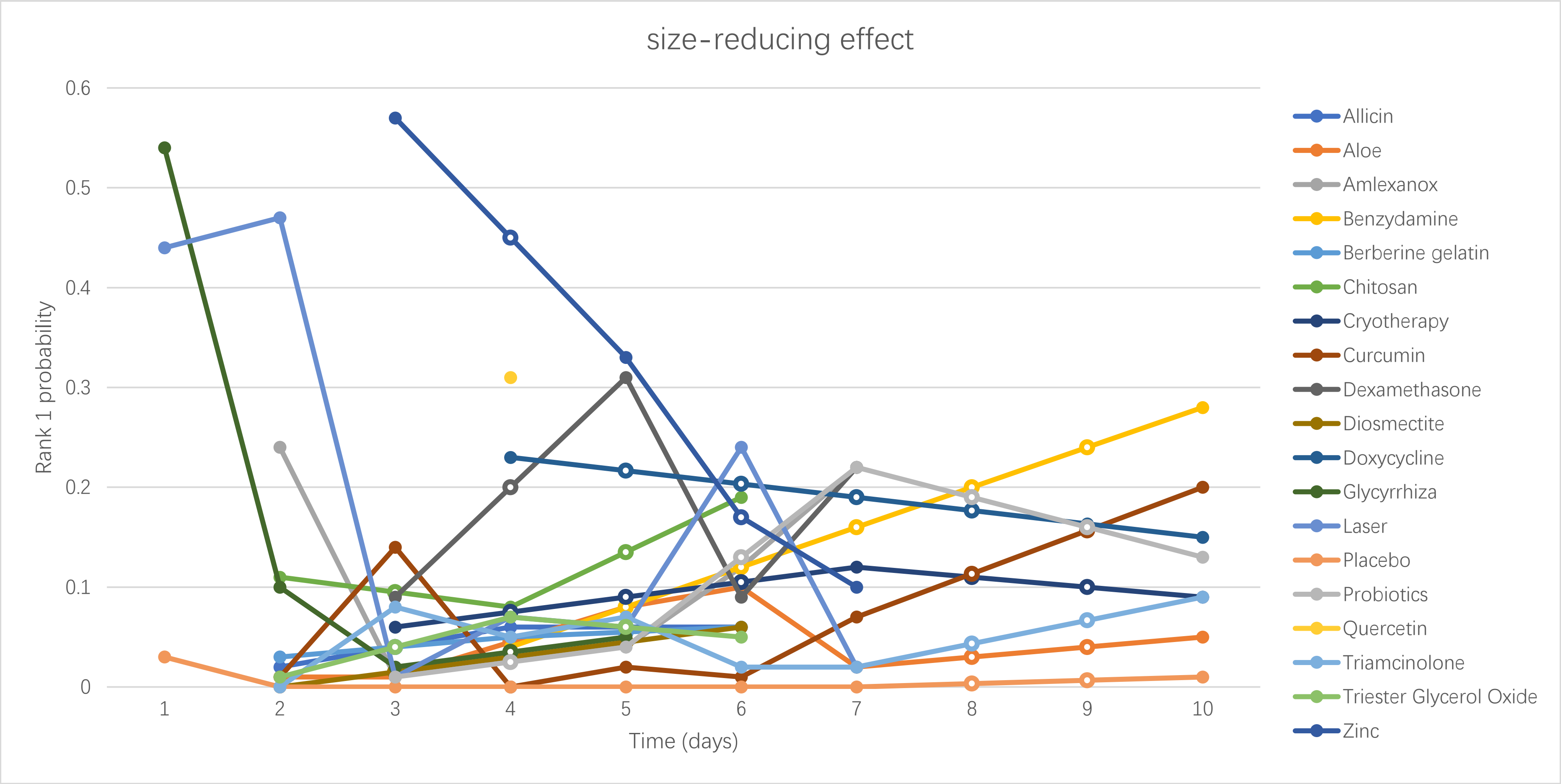
size-reducing effect
0.6
0.5
0.4
0.3
0.2
0.1
0
Rank 1 probability
1
2
3
4
5
6
7
8
9
10
Time (days)
Allicin
Aloe
Amlexanox
Benzydamine
Berberine gelatin
Chitosan
Cryotherapy
Curcumin
Dexamethasone
Diosmectite
Doxycycline
Glycyrrhiza
Laser
Placebo
Probiotics
Quercetin
Triamcinolone
Triester Glycerol Oxide
Zinc

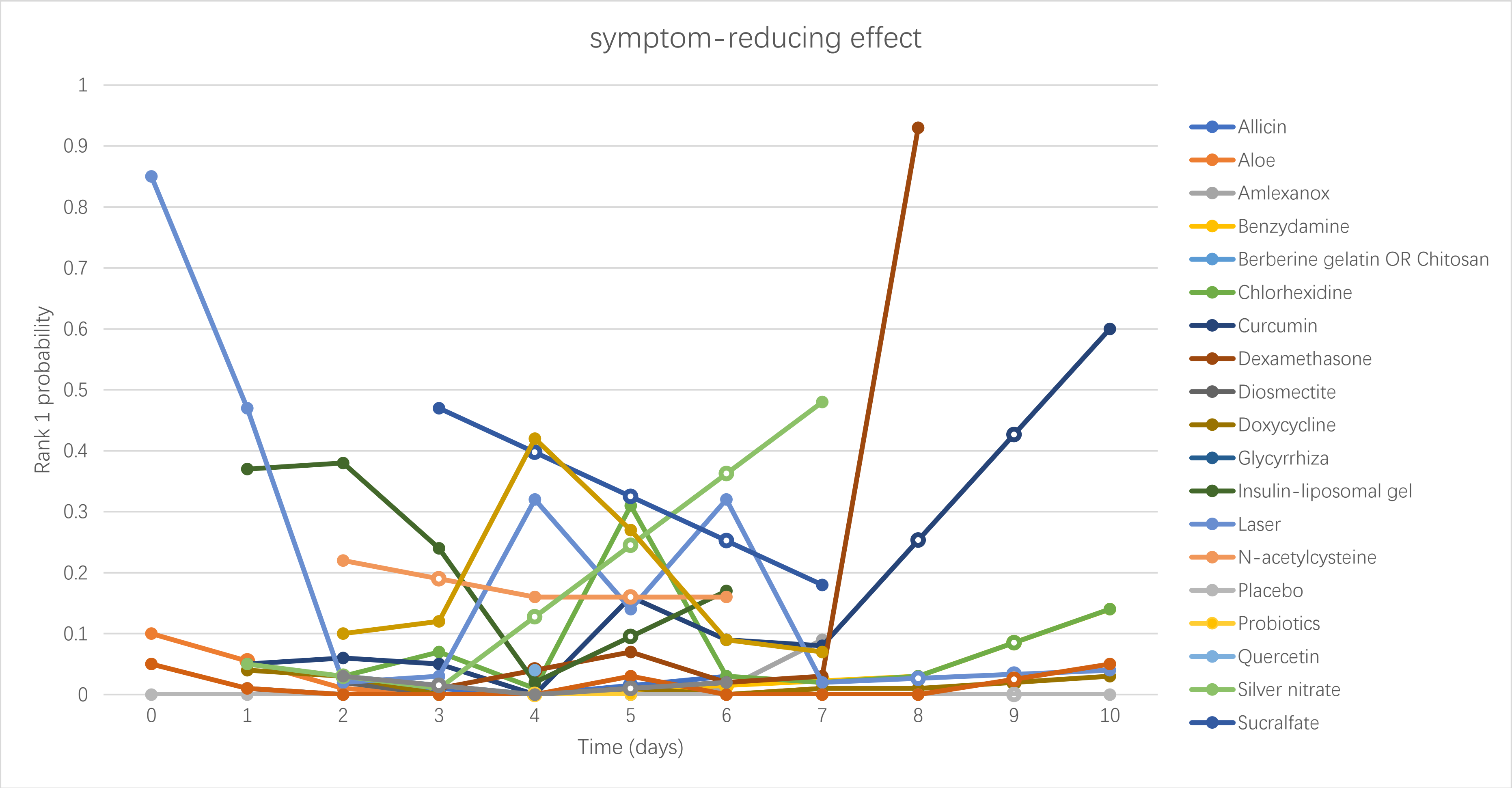
symptom-reducing effect
Rank 1 probability
Time (days)
1
0.9
0.8
0.7
0.6
0.5
0.4
0.3
0.2
0.1
0
0 1 2 3 4 5 6 7 8 9 10
Allicin
Aloe
Amlexanox
Benzydamine
Berberine gelatin OR Chitosan
Chlorhexidine
Curcumin
Dexamethasone
Diosmectite
Doxycycline
Glycyrrhiza
Insulin-liposomal gel
Laser
N-acetylcysteine
Placebo
Probiotics
Quercetin
Silver nitrate
Sucralfate

## Chapter S4 Safety outcomes

Adverse effect

36 RCTs included in the study reported on the side effects of the topical interventions.

| Interventions | RCTs | Events | Total |
|---|---|---|---|
| Allicin | 1 | 0 | 48 |
| Aloe | 1 | 0 | 60 |
| Amlexanox | 5 | 9 (4 report local, transient “stinging”, 3 report "cooling" sensation and 3 report metallic taste) | 328 |
| Benzydamine | 1 | 0 | 10 |
| Berberine gelatin | 1 | 0 | 48 |
| Chitosan | 2 | 1 (mild throat sore and bad taste) | 54 |
| Chlorhexidine | 1 | 0 | 35 |
| Curcumin | 4 | 0 | 117 |
| Dexamethasone | 1 | 4 (slight rash surrounding the mouth, a burning sensation in the larynx, or a stimulant pain at the medication site) | 120 |
| Diosmectite | 1 | 0 | 35 |
| Doxycycline | 3 | 9(1 report short burning sensation, 8 report transient bitter sensation ) | 60 |
| Glycyrrhiza | 1 | 0 | 20 |
| Honey | 2 | 0 | 92 |
| Laser | 5 | 0 | 95 |
| Penicillin | 1 | 3 (minor burning sensation) | 25 |
| Probiotics | 2 | 0 | 25 |
| Silver nitrate | 2 | 0 | 60 |
| Sucralfate | 1 | 0 | 35 |
| Triamcinolone | 9 | 0 | 259 |
| Triester Glycerol Oxide | 1 | 0 | 56 |
| Zinc | 1 | 0 | 23 |

## 1.Consistency Model

# Summary estimates for averse effect

The estimate values for adverse effect are given as risk difference (RD) and 95% confidence interval (CI). The table shows the pooled estimates based on the network meta-analysis during the treatment period for each study.

| | | | | | | | | |
|---|---|---|---|---|---|---|---|---|
| Allicin | 0.00 (0.00, 67795612 | 29743559.73 (0.00, 1 | 95.50 (0.00, 5855834 | 0.10 (0.00, 31074703 | 488139.21 (0.00, 388 | 0.23 (0.00, 51724186 | 30463.40 (0.00, 4235 | 971619.18 (0.00, 330 |
| 1779.43 (0.00, 12801 | Aloe | 15406588244.49 (0.0 | 358636.95 (0.00, 444 | 353.77 (0.00, 619917 | 2375935428.17 (0.00 | 42.89 (0.00, 2590922 | 4255653.14 (0.00, 17 | 625925929.84 (0.00, |
| 0.00 (0.00, 98262861 | 0.00 (0.00, 27443474 | Amlexanox | 0.00 (0.00, 35039437 | 0.00 (0.00, 16464214 | 0.00 (0.00, 24032678 | 0.00 (0.00, 15711136 | 0.00 (0.00, 18410647 | 0.03 (0.00, 12.88) |
| 0.01 (0.00, 29259572 | 0.00 (0.00, 24059808 | 91091.27 (0.00, 3948 | Benzydamine | 0.01 (0.00, 85083806 | 178.26 (0.00, 154837 | 0.01 (0.00, 27660634 | 20.51 (0.00, 1992265 | 3019.29 (0.00, 10586 |
| 9.76 (0.00, 19271667 | 0.00 (0.00, 15599742 | 86904344.51 (0.00, 3 | 151.75 (0.00, 352718 | Berberine gelatin | 2052733.92 (0.00, 55 | 0.20 (0.00, 49595304 | 10236.77 (0.00, 1389 | 3690931.68 (0.00, 16 |
| 0.00 (0.00, 11342492 | 0.00 (0.00, 63626914 | 299.48 (0.00, 188077 | 0.01 (0.00, 30363564 | 0.00 (0.00, 98707875 | Chitosan | 0.00 (0.00, 17287171 | 0.00 (0.00, 43748785 | 11.22 (0.00, 7559230 |
| 4.37 (0.00, 13881182 | 0.02 (0.00, 44161219 | 84775814.80 (0.00, 1 | 92.96 (0.00, 1236731 | 4.93 (0.00, 14399524 | 2035525.09 (0.00, 64 | Clobetasol | 14891.99 (0.00, 2269 | 2610072.73 (0.00, 71 |
| 0.00 (0.00, 15581864 | 0.00 (0.00, 27721300 | 17259.99 (0.00, 6682 | 0.05 (0.00, 24395746 | 0.00 (0.00, 50818455 | 222.26 (0.00, 366016 | 0.00 (0.00, 10637383 | Curcumin | 645.06 (0.00, 243441 |
| 0.00 (0.00, 27013289 | 0.00 (0.00, 72925201 | 29.91 (0.08, 18750.26 | 0.00 (0.00, 12955323 | 0.00 (0.00, 58074401 | 0.09 (0.00, 64455000 | 0.00 (0.00, 68097053 | 0.00 (0.00, 61445532 | Dexamethasone |
| 0.12 (0.00, 52192537 | 0.00 (0.00, 10390338 | 4240298.60 (0.00, 75 | 22.84 (0.00, 1024881 | 0.01 (0.00, 69936172 | 73377.77 (0.00, 5592 | 0.02 (0.00, 21374551 | 541.06 (0.00, 235778 | 133596.25 (0.00, 306 |
| 0.00 (0.00, 10925525 | 0.00 (0.00, 5004320.9 | 0.00 (0.00, 1.53) | 0.00 (0.00, 36427266 | 0.00 (0.00, 21895129 | 0.00 (0.00, 81447246 | 0.00 (0.00, 34147359 | 0.00 (0.00, 16502032 | 0.00 (0.00, 0.06) |
| 13.67 (0.00, 1771286 | 0.00 (0.00, 19226614 | 286314486.75 (0.00, | 7742.05 (0.00, 65204 | 1.77 (0.00, 51396439 | 9486779.95 (0.00, 29 | 5.81 (0.00, 66181361 | 68947.82 (0.00, 1899 | 8338551.59 (0.00, 22 |
| 510558.19 (0.00, 112 | 241.04 (0.00, 475799 | 2357303542236.30 ( | 289254382.66 (0.00, | 9579.77 (0.00, 19191 | 3111833965.72 (0.00 | 8353.42 (0.00, 23398 | 114289569.02 (0.00, | 83222103031.52 (0.0 |
| 41.55 (0.00, 8980422 | 0.28 (0.00, 85242948 | 1562196143.41 (0.00 | 323830.39 (0.00, 148 | 1553.83 (0.00, 42529 | 165292229.76 (0.00, | 179.19 (0.00, 186861 | 317660.64 (0.00, 578 | 48425776.88 (0.00, 1 |
| 1.19 (0.00, 34653363 | 0.00 (0.00, 18056717 | 1147369.31 (0.00, 21 | 197.87 (0.00, 383900 | 0.02 (0.00, 12256261 | 10309.06 (0.00, 5317 | 0.02 (0.00, 45655144 | 597.27 (0.00, 575993 | 45903.10 (0.00, 3949 |
| 0.00 (0.00, 12424267 | 0.00 (0.00, 3490516.2 | 0.00 (0.00, 3.45) | 0.00 (0.00, 19470081 | 0.00 (0.00, 10179858 | 0.00 (0.00, 43915386 | 0.00 (0.00, 84338449 | 0.00 (0.00, 23105133 | 0.00 (0.00, 0.10) |
| 0.00 (0.00, 98107911 | 0.00 (0.00, 31435817 | 11.87 (0.28, 1221.84) | 0.00 (0.00, 39291446 | 0.00 (0.00, 28271294 | 0.04 (0.00, 25336157 | 0.00 (0.00, 28099223 | 0.00 (0.00, 29768411 | 0.44 (0.00, 39.68) |
| 14217.21 (0.00, 2848 | 6.32 (0.00, 23909266 | 18560648162.68 (0.0 | 1903891.94 (0.00, 46 | 96.44 (0.00, 1725994 | 1631705442.78 (0.00 | 985.08 (0.00, 204170 | 10744120.37 (0.00, 2 | 685908650.27 (0.00, |
| 9.53 (0.00, 65559000 | 0.03 (0.00, 43838884 | 634303158.67 (0.00, | 12978.14 (0.00, 4375 | 78.64 (0.00, 6700159 | 77513643.34 (0.00, 1 | 398.10 (0.00, 692762 | 236305.11 (0.00, 167 | 22182114.23 (0.00, 6 |
| 97756864232858512 | 405677176194523.2 | 558742857301789 20 | 435531054083613 13 | 4630196115201825.( | 1861881510852974 3 | 656791543828444 56 | 734107903963077 30 | 205706513027168 12 |
| 6.75 (0.00, 16274194 | 0.00 (0.00, 23290053 | 248042441.84 (0.00, | 1968.29 (0.00, 35089 | 0.50 (0.00, 23620006 | 1303905.50 (0.00, 57 | 1.01 (0.00, 18551345 | 819.39 (0.00, 115557 | 8362648.73 (0.00, 93 |
| 25.84 (0.00, 1738817 | 0.03 (0.00, 17928084 | 304885285.84 (0.00, | 252.70 (0.00, 481991 | 0.96 (0.00, 40952882 | 8737844.55 (0.00, 45 | 0.53 (0.00, 12379849 | 111743.09 (0.00, 272 | 10359459.65 (0.00, 6 |

| | | | | | | | | |
|---|---|---|---|---|---|---|---|---|
| 8.07 (0.00, 14245820 | 179159184019466.6 | 0.07 (0.00, 74417800 | 0.00 (0.00, 67673897 | 0.02 (0.00, 91598076 | 0.84 (0.00, 14473074 | 11995410744985560 | 2081706.29 (0.00, 62 | 0.00 (0.00, 49806243 |
| 26577.60 (0.00, 8983 | 16762833642392473 | 236.82 (0.00, 510091 | 0.00 (0.00, 18211922 | 3.62 (0.00, 76243181 | 3847.17 (0.00, 52303 | 14291063434929485 | 1338904140.46 (0.00 | 0.16 (0.00, 31799259 |
| 0.00 (0.00, 81521727 | 1395278.26 (0.65, 26 | 0.00 (0.00, 47915409 | 0.00 (0.00, 42037465 | 0.00 (0.00, 39903372 | 0.00 (0.00, 31774052 | 7789711215.21 (0.29 | 0.08 (0.00, 3.52) | 0.00 (0.00, 71629218 |
| 0.04 (0.00, 44096871 | 524088819802.66 (0. | 0.00 (0.00, 14235591 | 0.00 (0.00, 24955402 | 0.00 (0.00, 14645864 | 0.01 (0.00, 11460975 | 13028662979938912 | 6996.16 (0.00, 24614 | 0.00 (0.00, 95606285 |
| 82.04 (0.00, 8928644 | 3254276114437173. | 0.56 (0.00, 37165177 | 0.00 (0.00, 39308743 | 0.00 (0.00, 45789221 | 65.90 (0.00, 1020745 | 14084693526390493 | 7939707.38 (0.00, 30 | 0.01 (0.00, 46881564 |
| 0.00 (0.00, 86014641 | 3560728226.33 (0.00 | 0.00 (0.00, 14984363 | 0.00 (0.00, 98007152 | 0.00 (0.00, 11598082 | 0.00 (0.00, 10970948 | 12411002727300.38 | 23.86 (0.00, 1372103 | 0.00 (0.00, 26401373 |
| 54.19 (0.00, 5285005 | 367785632764922.5 | 0.17 (0.00, 11575467 | 0.00 (0.00, 12274737 | 0.01 (0.00, 24799032 | 45.64 (0.00, 5659900 | 25737532503420314 | 6100256.94 (0.00, 13 | 0.00 (0.00, 58430438 |
| 0.00 (0.00, 28850985 | 90780915358.26 (0.0 | 0.00 (0.00, 18164450 | 0.00 (0.00, 74339734 | 0.00 (0.00, 11261167 | 0.00 (0.00, 25822676 | 1869763179827788. | 1350.55 (0.00, 40061 | 0.00 (0.00, 11177273 |
| 0.00 (0.00, 22294281 | 40345058.39 (17.51, | 0.00 (0.00, 13733717 | 0.00 (0.00, 14795078 | 0.00 (0.00, 12333577 | 0.00 (0.00, 88681834 | 219021826291.38 (9. | 2.26 (0.03, 202.63) | 0.00 (0.00, 28340018 |
| Diosmectite | 20134650118030.25 | 0.00 (0.00, 79541098 | 0.00 (0.00, 25718931 | 0.00 (0.00, 13535773 | 1.15 (0.00, 18908035 | 64532567030743680 | 297291.60 (0.00, 497 | 0.00 (0.00, 26028447 |
| 0.00 (0.00, 30066570 | Doxycycline | 0.00 (0.00, 15788394 | 0.00 (0.00, 59841474 | 0.00 (0.00, 875.10) | 0.00 (0.00, 13756889 | 3885.43 (0.00, 61929 | 0.00 (0.00, 0.06) | 0.00 (0.00, 30368085 |
| 367.00 (0.00, 119978 | 6660105593931289. | Glycyrrhiza | 0.00 (0.00, 28768137 | 0.01 (0.00, 41191073 | 72.89 (0.00, 8926664 | 22434624791409250 | 18198051.83 (0.00, 5 | 0.00 (0.00, 15112021 |
| 6628256.69 (0.00, 14 | 15765004434093180 | 14188.85 (0.00, 1878 | Honey | 163.62 (0.00, 150275 | 804045.86 (0.00, 508 | 42442200157852845 | 181239811725.30 (0. | 8.25 (0.00, 25762060 |
| 1600.59 (0.00, 20545 | 58233478189134896 | 121.76 (0.00, 132871 | 0.01 (0.00, 59423727 | Laser | 10535.23 (0.00, 2965 | 11553708204429963 | 105101772.45 (0.00, | 0.03 (0.00, 10741497 |
| 0.87 (0.00, 14206879 | 21648679651076.24 | 0.01 (0.00, 29198886 | 0.00 (0.00, 28574717 | 0.00 (0.00, 14165912 | Minocycline | 64805737992517645 | 99627.97 (0.00, 8707 | 0.00 (0.00, 18022840 |
| 0.00 (0.00, 10510171 | 0.00 (0.00, 24012917 | 0.00 (0.00, 66271843 | 0.00 (0.00, 55858630 | 0.00 (0.00, 106.57) | 0.00 (0.00, 23654218 | Penicillin | 0.00 (0.00, 0.14) | 0.00 (0.00, 566069.82 |
| 0.00 (0.00, 88297629 | 17629126.31 (17.46, | 0.00 (0.00, 66378286 | 0.00 (0.00, 58563467 | 0.00 (0.00, 38948662 | 0.00 (0.00, 29595640 | 103133737876.48 (7. | Placebo | 0.00 (0.00, 11003059 |
| 3388.91 (0.00, 58273 | 22662818563877283 | 386.08 (0.00, 392880 | 0.12 (0.00, 64789700 | 30.10 (0.00, 4900062 | 180809.91 (0.00, 133 | 12705884877122506 | 1554787148.86 (0.00 | Probiotics |
| 205.04 (0.00, 675817 | 14326490070467732 | 4.20 (0.00, 47879380 | 0.00 (0.00, 85040573 | 0.92 (0.00, 76564274 | 3745.88 (0.00, 23124 | 91231175311989960 | 43876646.48 (0.00, 1 | 0.00 (0.00, 10656641 |
| 48583172399791750 | 90339117659138750 | 786123725181457.0 | 75281414437.06 (0.0 | 40782394449006.22 | 21091022864645209 | 23278843419680943 | 43120130982564420 | 5409036028181.45 ( |
| 111.40 (0.00, 626963 | 3304812767729931. | 1.45 (0.00, 13809747 | 0.00 (0.00, 44072515 | 0.02 (0.00, 79103293 | 17.56 (0.00, 5484954 | 15866992607524266 | 19087254.90 (0.00, 1 | 0.00 (0.00, 62186403 |
| 132.85 (0.00, 399774 | 9069683347239406. | 0.03 (0.00, 36923980 | 0.00 (0.00, 21246026 | 0.01 (0.00, 11271375 | 135.87 (0.00, 380184 | 96423371705792150 | 20780188.25 (0.00, 1 | 0.00 (0.00, 18154334 |

| | | | |
|---|---|---|---|
| 0.10 (0.00, 16291917 | 0.00 (0.00, 53658820 | 0.15 (0.00, 74026898 | 0.04 (0.00, 58222375 |
| 29.03 (0.00, 5602728 | 0.00 (0.00, 20060255 | 365.43 (0.00, 640568 | 31.15 (0.00, 2447007 |
| 0.00 (0.00, 35941342 | 0.00 (0.00, 0.00) | 0.00 (0.00, 52421976 | 0.00 (0.00, 20572351 |
| 0.00 (0.00, 22415412 | 0.00 (0.00, 38722108 | 0.00 (0.00, 32023617 | 0.00 (0.00, 53928468 |
| 0.01 (0.00, 10614015 | 0.00 (0.00, 99593565 | 2.02 (0.00, 51661781 | 1.05 (0.00, 18710743 |
| 0.00 (0.00, 15794748 | 0.00 (0.00, 0.08) | 0.00 (0.00, 99300653 | 0.00 (0.00, 85609614 |
| 0.00 (0.00, 11828327 | 0.00 (0.00, 11667577 | 0.99 (0.00, 25878136 | 1.89 (0.00, 13455355 |
| 0.00 (0.00, 20098081 | 0.00 (0.00, 70453969 | 0.00 (0.00, 27431359 | 0.00 (0.00, 22469827 |
| 0.00 (0.00, 11416080 | 0.00 (0.00, 0.00) | 0.00 (0.00, 16705726 | 0.00 (0.00, 59804123 |
| 0.00 (0.00, 18643620 | 0.00 (0.00, 20061798 | 0.01 (0.00, 15725986 | 0.01 (0.00, 36048854 |
| 0.00 (0.00, 46650966 | 0.00 (0.00, 0.00) | 0.00 (0.00, 11113104 | 0.00 (0.00, 32200723 |
| 0.24 (0.00, 84524037 | 0.00 (0.00, 40907691 | 0.69 (0.00, 38703867 | 29.65 (0.00, 4083894 |
| 435.09 (0.00, 914316 | 0.00 (0.00, 15799893 | 22675.55 (0.00, 5095 | 27664.93 (0.00, 1636 |
| 1.09 (0.00, 85720831 | 0.00 (0.00, 80493267 | 48.64 (0.00, 4955122 | 109.18 (0.00, 109589 |
| 0.00 (0.00, 34802356 | 0.00 (0.00, 95466242 | 0.06 (0.00, 68283635 | 0.01 (0.00, 11419142 |
| 0.00 (0.00, 436115.83 | 0.00 (0.00, 0.00) | 0.00 (0.00, 17159074 | 0.00 (0.00, 88662838 |
| 0.00 (0.00, 52018465 | 0.00 (0.00, 0.00) | 0.00 (0.00, 63331872 | 0.00 (0.00, 20611439 |
| 2184.43 (0.00, 12867 | 0.00 (0.00, 16694787 | 503.22 (0.00, 353615 | 251.83 (0.00, 133121 |
| Silver nitrate | 0.00 (0.00, 23044968 | 11.42 (0.00, 3784319 | 3.00 (0.00, 33153874 |
| 1267023415621751.5 | Triamcinolone | 1243638458607581.2 | 8561321338436 12.1 |
| 0.09 (0.00, 60903990 | 0.00 (0.00, 18456638 | Triester Glycerol Oxid | 1.18 (0.00, 18011035 |
| 0.33 (0.00, 13853399 | 0.00 (0.00, 71797328 | 0.85 (0.00, 36947522 | Zinc |

## Rank probability

**Rank 1 is worst, rank N is best.**

| Drug | Rank 1 | Rank 2 | Rank 3 | Rank 4 | Rank 5 | Rank 6 | Rank 7 | Rank 8 | Rank 9 | Rank 10 | Rank 11 |
|---|---|---|---|---|---|---|---|---|---|---|---|
| Allicin | 0.04 | 0.05 | 0.04 | 0.04 | 0.04 | 0.04 | 0.03 | 0.03 | 0.02 | 0.03 | 0.04 |
| Aloe | 0.01 | 0.02 | 0.03 | 0.03 | 0.03 | 0.03 | 0.03 | 0.03 | 0.03 | 0.03 | 0.04 |
| Amlexanox | 0 | 0 | 0 | 0.03 | 0.08 | 0.16 | 0.2 | 0.2 | 0.14 | 0.09 | 0.05 |
| Benzydamine | 0.07 | 0.06 | 0.06 | 0.05 | 0.05 | 0.05 | 0.03 | 0.02 | 0.03 | 0.03 | 0.04 |
| Berberine gelatin | 0.05 | 0.04 | 0.04 | 0.04 | 0.04 | 0.03 | 0.03 | 0.02 | 0.03 | 0.03 | 0.04 |
| Chitosan | 0.07 | 0.07 | 0.07 | 0.07 | 0.06 | 0.04 | 0.04 | 0.03 | 0.03 | 0.04 | 0.05 |
| Clobetasol | 0.05 | 0.05 | 0.05 | 0.04 | 0.04 | 0.04 | 0.03 | 0.03 | 0.03 | 0.03 | 0.03 |
| Curcumin | 0.06 | 0.05 | 0.05 | 0.05 | 0.05 | 0.04 | 0.05 | 0.02 | 0.03 | 0.04 | 0.05 |
| Dexamethasone | 0 | 0 | 0 | 0 | 0.01 | 0.04 | 0.08 | 0.13 | 0.18 | 0.2 | 0.15 |
| Diosmectite | 0.05 | 0.05 | 0.05 | 0.05 | 0.04 | 0.03 | 0.03 | 0.02 | 0.02 | 0.03 | 0.05 |
| Doxycycline | 0.07 | 0.13 | 0.19 | 0.2 | 0.16 | 0.13 | 0.06 | 0.03 | 0.01 | 0.01 | 0 |
| Glycyrrhiza | 0.05 | 0.05 | 0.04 | 0.04 | 0.04 | 0.03 | 0.03 | 0.02 | 0.02 | 0.03 | 0.03 |
| Honey | 0.03 | 0.02 | 0.02 | 0.03 | 0.03 | 0.03 | 0.03 | 0.02 | 0.02 | 0.03 | 0.04 |
| Laser | 0 | 0.01 | 0.01 | 0.03 | 0.03 | 0.03 | 0.03 | 0.03 | 0.03 | 0.04 | 0.07 |
| Minocycline | 0.05 | 0.05 | 0.05 | 0.04 | 0.06 | 0.04 | 0.03 | 0.03 | 0.03 | 0.03 | 0.04 |
| Penicillin | 0.27 | 0.2 | 0.14 | 0.11 | 0.1 | 0.07 | 0.05 | 0.03 | 0.02 | 0.01 | 0 |
| Placebo | 0 | 0 | 0 | 0 | 0.01 | 0.05 | 0.11 | 0.18 | 0.21 | 0.17 | 0.13 |
| Probiotics | 0.01 | 0.02 | 0.02 | 0.02 | 0.02 | 0.03 | 0.02 | 0.03 | 0.03 | 0.03 | 0.04 |
| Silver nitrate | 0.01 | 0.03 | 0.03 | 0.03 | 0.04 | 0.03 | 0.03 | 0.03 | 0.03 | 0.04 | 0.04 |
| Triamcinolone | 0 | 0 | 0 | 0 | 0 | 0 | 0 | 0 | 0 | 0 | 0 |
| Triester Glycerol Oxide | 0.06 | 0.05 | 0.06 | 0.03 | 0.04 | 0.03 | 0.03 | 0.02 | 0.03 | 0.03 | 0.03 |
| Zinc | 0.06 | 0.04 | 0.06 | 0.04 | 0.03 | 0.03 | 0.03 | 0.02 | 0.02 | 0.03 | 0.03 |

| Rank 12 | Rank 13 | Rank 14 | Rank 15 | Rank 16 | Rank 17 | Rank 18 | Rank 19 | Rank 20 | Rank 21 | Rank 22 |
|---|---|---|---|---|---|---|---|---|---|---|
| 0.04 | 0.07 | 0.07 | 0.07 | 0.06 | 0.06 | 0.05 | 0.05 | 0.05 | 0.05 | 0.04 |
| 0.05 | 0.06 | 0.06 | 0.06 | 0.07 | 0.07 | 0.07 | 0.06 | 0.06 | 0.06 | 0.06 |
| 0.02 | 0.01 | 0 | 0 | 0 | 0 | 0 | 0 | 0 | 0 | 0 |
| 0.04 | 0.05 | 0.04 | 0.04 | 0.05 | 0.04 | 0.05 | 0.04 | 0.05 | 0.05 | 0.04 |
| 0.04 | 0.05 | 0.05 | 0.07 | 0.06 | 0.06 | 0.06 | 0.05 | 0.05 | 0.08 | 0.04 |
| 0.05 | 0.07 | 0.06 | 0.06 | 0.05 | 0.05 | 0.04 | 0.03 | 0.02 | 0.01 | 0 |
| 0.04 | 0.06 | 0.05 | 0.06 | 0.05 | 0.05 | 0.05 | 0.05 | 0.06 | 0.05 | 0.07 |
| 0.05 | 0.06 | 0.06 | 0.06 | 0.06 | 0.05 | 0.06 | 0.05 | 0.04 | 0.02 | 0 |
| 0.11 | 0.06 | 0.03 | 0.01 | 0 | 0 | 0 | 0 | 0 | 0 | 0 |
| 0.05 | 0.05 | 0.07 | 0.06 | 0.06 | 0.06 | 0.04 | 0.05 | 0.05 | 0.06 | 0.04 |
| 0 | 0 | 0 | 0 | 0 | 0 | 0 | 0 | 0 | 0 | 0 |
| 0.05 | 0.05 | 0.05 | 0.05 | 0.05 | 0.05 | 0.06 | 0.06 | 0.07 | 0.06 | 0.08 |
| 0.04 | 0.05 | 0.05 | 0.06 | 0.06 | 0.07 | 0.06 | 0.06 | 0.05 | 0.07 | 0.12 |
| 0.07 | 0.07 | 0.06 | 0.06 | 0.06 | 0.06 | 0.06 | 0.07 | 0.07 | 0.06 | 0.03 |
| 0.05 | 0.05 | 0.05 | 0.05 | 0.05 | 0.05 | 0.05 | 0.05 | 0.05 | 0.05 | 0.06 |
| 0 | 0 | 0 | 0 | 0 | 0 | 0 | 0 | 0 | 0 | 0 |
| 0.08 | 0.04 | 0.01 | 0 | 0 | 0 | 0 | 0 | 0 | 0 | 0 |
| 0.06 | 0.06 | 0.07 | 0.09 | 0.07 | 0.07 | 0.07 | 0.06 | 0.06 | 0.06 | 0.05 |
| 0.06 | 0.05 | 0.07 | 0.07 | 0.07 | 0.07 | 0.06 | 0.05 | 0.05 | 0.04 | 0.07 |
| 0.01 | 0.01 | 0.03 | 0.04 | 0.06 | 0.08 | 0.1 | 0.16 | 0.16 | 0.18 | 0.15 |
| 0.04 | 0.05 | 0.06 | 0.06 | 0.05 | 0.06 | 0.06 | 0.05 | 0.06 | 0.05 | 0.07 |
| 0.04 | 0.05 | 0.05 | 0.05 | 0.07 | 0.05 | 0.06 | 0.05 | 0.05 | 0.04 | 0.08 |

## Consistency check

| Parameter | Median (95% CI) |
|---|---|
| Random Effects Standard Deviation | 1.96 (0.12, 3.15) |

## Convergence Diagnostics

Convergence is assessed using the Brooks-Gelman-Rubin method. This method compares within-chain and between-chain variance to calculate the Potential Scale Reduction Factor (PSRF). A PSRF close to one indicates approximate convergence has been reached.

| Parameter | PSRF |
|---|---|
| d.Amlexanox.Clobetasold.Placebo.Aloe | 1.01 |
| d.Amlexanox.Placebo | 1.00 |
| d.Placebo.Allicin | 1.01 |
| d.Placebo.Aloe | 1.01 |
| d.Placebo.Benzydamine | 1.04 |
| d.Placebo.Berberinegelatin | 1.03 |
| d.Placebo.Chitosan | 1.03 |
| d.placebo.Curcumin | 1.04 |
| d.Placebo.Dexamethasoned.Placebo.Laser | 1.00 |
| d.Placebo.Diosmectite | 1.01 |
| d.Placebo.Doxycycline | 1.07 |
| d.Placebo.Glycyrrhiza | 1.02 |
| d.Placebo.Honey | 1.01 |
| d.Placebo.Laser | 1.03 |
| d.Placebo.Minocycline | 1.03 |
| d.Placebo.Penicillin | 1.07 |
| d.Placebo.Probiotics | 1.01 |
| d.Placebo.Silvernitrate | 1.04 |
| d.Placebo.Triamcinolone | 1.08 |
| d.Placebo.TriesterGlycerolOxide | 1.02 |
| d.Placebo.Zinc | 1.04 |
| sd.d | 1.00 |

Number of chains : 4
Tuning iterations : 20,000
Simulation iterations : 400,000
Thinning interval : 10
Inference samples : 80,000
Variance scaling factor : 2.5

## 2.Inconsistency Model

# Summary estimates for adverse effect

The estimate values for adverse effect are given as risk difference (RD) and 95% confidence interval (CI). The table shows the pooled estimates based on the network meta-analysis during the treatment period for each study.

| | | | | | | | | |
|---|---|---|---|---|---|---|---|---|
| Allicin | 422265.84 (0.00, 158 | 0.00 (0.00, 85516421 | 352322334030296.5( | 7.93 (0.00, 24350761 | 83192542590376346 | 0.00 (0.00, 31049907 | 910467984585.53 (0. | 0.00 (0.00, 98940324 |
| 0.00 (0.00, 35169296 | Aloe | 0.00 (0.00, 46947781 | 22713750037.61 (0.0 | 0.00 (0.00, 52523810 | 1276407829341.87 (( | 0.00 (0.00, 17986047 | 3283788.08 (0.00, 72 | 0.00 (0.00, 14341411 |
| 94650.94 (0.00, 8876 | 169041810429.87 (0. | Amlexanox | 62863198160201270 | 987824.54 (0.00, 974 | 30857461484745650 | 0.00 (0.00, 12062518 | 13854291570738086 | 0.03 (0.00, 9.76) |
| 0.00 (0.00, 68538205 | 0.00 (0.00, 14929798 | 0.00 (0.00, 47912728 | Benzydamine | 0.00 (0.00, 10188422 | 64.84 (0.00, 1733834 | 0.00 (0.00, 19104736 | 0.00 (0.00, 90876574 | 0.00 (0.00, 10714682 |
| 0.13 (0.00, 10640292 | 232495554.70 (0.00, | 0.00 (0.00, 19267313 | 58852321262523699 | Berberine gelatin | 33032267293880488 | 0.00 (0.00, 43822414 | 594041022656500.4( | 0.00 (0.00, 64478781 |
| 0.00 (0.00, 15767149 | 0.00 (0.00, 90153895 | 0.00 (0.00, 15.19) | 0.02 (0.00, 12528143 | 0.00 (0.00, 18664025 | Chitosan | 0.00 (0.00, 85933399 | 0.00 (0.00, 58970523 | 0.00 (0.00, 0.40) |
| 128313640154.88 (0. | 70296956814351980 | 151110011.76 (0.00, | 36109862853880890 | 577825229757310.9( | 19749517812388528 | Clobetasol | 27144701463504880 | 5665357.22 (0.00, 12 |
| 0.00 (0.00, 18529853 | 0.00 (0.00, 33088175 | 0.00 (0.00, 47993683 | 8459.06 (0.00, 16504 | 0.00 (0.00, 13774533 | 593840.28 (0.00, 103 | 0.00 (0.00, 38549881 | Curcumin | 0.00 (0.00, 1423358.5 |
| 3593403.97 (0.00, 41 | 6065040763170.86 (( | 31.43 (0.10, 21450.83 | 26601751751452710 | 36984516.01 (0.00, 2 | 12237882876578776 | 0.00 (0.00, 80397817 | 52922940155503080 | Dexamethasone |
| 0.01 (0.00, 30456428 | 4674069.71 (0.00, 22 | 0.00 (0.00, 42645192 | 1598421728222236.( | 0.03 (0.00, 41091319 | 16422662015050498 | 0.00 (0.00, 77447509 | 11734841593834.45 | 0.00 (0.00, 13651660 |
| 0.00 (0.00, 15667396 | 0.00 (0.00, 64388183 | 0.00 (0.00, 0.07) | 0.01 (0.00, 17712484 | 0.00 (0.00, 91148672 | 7.70 (0.00, 31876142 | 0.00 (0.00, 75465437 | 0.00 (0.00, 63302478 | 0.00 (0.00, 0.00) |
| 0.00 (0.00, 60830700 | 6155116.67 (0.00, 23 | 0.00 (0.00, 36805384 | 1350666592352402.8 | 2.24 (0.00, 29028825 | 27741575729352348 | 0.00 (0.00, 32286278 | 3986507223495.19 (( | 0.00 (0.00, 10493601 |
| 15871.16 (0.00, 2412 | 481153680.27 (0.00, | 0.01 (0.00, 20908061 | 78215749904620450 | 99.78 (0.00, 1937091 | 10558439862186722 | 0.00 (0.00, 11213947 | 8618743338451650.( | 0.00 (0.00, 70567479 |
| 0.00 (0.00, 71385981 | 0.00 (0.00, 62953482 | 0.00 (0.00, 386081.09 | 4273.99 (0.00, 28098 | 0.00 (0.00, 46374906 | 1035.85 (0.00, 35388 | 0.00 (0.00, 10788499 | 0.02 (0.00, 17171565 | 0.00 (0.00, 10191.69) |
| 0.02 (0.00, 74985556 | 47591.94 (0.00, 1760 | 0.00 (0.00, 13745429 | 16824892997318467 | 0.00 (0.00, 65371725 | 77385943693983322 | 0.00 (0.00, 22520979 | 71251198136.22 (0.0 | 0.00 (0.00, 27569399 |
| 0.00 (0.00, 40057517 | 0.00 (0.00, 78356944 | 0.00 (0.00, 2.53) | 236627369.95 (0.00, | 0.00 (0.00, 81190738 | 186837268.63 (0.00, | 0.00 (0.00, 48025456 | 3567.16 (0.00, 25224 | 0.00 (0.00, 0.06) |
| 1679696.94 (0.00, 17 | 2752512854747.20 (( | 16.22 (0.38, 1524.01) | 11318806839505591 | 17241818.13 (0.00, 1 | 46649515322083217 | 0.00 (0.00, 29655350 | 19914636765761920 | 0.47 (0.01, 38.86) |
| 69532.69 (0.00, 2920 | 498696883304.45 (0. | 16.68 (0.00, 2635201 | 15333360552885422 | 18197313.67 (0.00, 1 | 24229502765351440 | 0.00 (0.00, 20060009 | 73755720129443930 | 0.36 (0.00, 58818271 |
| 3108811350585493.( | 42902134952894750 | 1065744093.79 (0.00 | 87746055000639840 | 1994128090329077.8 | 10485202603346870 | 94.99 (0.00, 8737841 | 29390190331685585 | 25812665.84 (0.00, 2 |
| 0.01 (0.00, 63000346 | 828.65 (0.00, 151351 | 0.00 (0.00, 31323559 | 137512123533496.6 | 0.00 (0.00, 12831286 | 498551080354059.3( | 0.00 (0.00, 75976960 | 1038707174.14 (0.00 | 0.00 (0.00, 63412111 |
| 0.00 (0.00, 33590025 | 0.00 (0.00, 50490538 | 0.00 (0.00, 14361159 | 407933.33 (0.00, 322 | 0.00 (0.00, 30855035 | 4264218.97 (0.00, 95 | 0.00 (0.00, 31550031 | 4.76 (0.00, 45703897 | 0.00 (0.00, 14230969 |
| 14316349579508516 | 78563561659500100 | 5432267777.05 (0.00 | 35801671111418930 | 72577669483211377 | 12795263427931641 | 1950.60 (0.00, 29213 | 33414012786374590 | 141940971.11 (0.00, |

| | | | | | | | | |
|---|---|---|---|---|---|---|---|---|
| 107.84 (0.00, 499235 | 2525997735344041.( | 215.94 (0.00, 107200 | 0.00 (0.00, 26700287 | 92575671482862.42 | 58.99 (0.00, 3721806 | 648418690.41 (0.00, | 0.00 (0.00, 20214264 | 0.00 (0.00, 86961249 |
| 0.00 (0.00, 21485820 | 72339264193.05 (0.0 | 0.00 (0.00, 62241819 | 0.00 (0.00, 18457147 | 17361060466.26 (0.0 | 0.00 (0.00, 22966770 | 3142.31 (0.00, 21042 | 0.00 (0.00, 30176530 | 0.00 (0.00, 45807097 |
| 255690.79 (0.00, 234 | 927434120572631100 | 3567619.48 (0.00, 18 | 71.56 (0.00, 1089817 | 534521948047311000 | 29931781.79 (0.00, 2 | 3898944829886.85 ( | 0.06 (0.00, 2.62) | 0.06 (0.00, 10246570 |
| 0.00 (0.00, 57627105 | 102.85 (0.00, 148140 | 0.00 (0.00, 33743220 | 0.00 (0.00, 22728826 | 0.00 (0.00, 10161871 | 0.00 (0.00, 10410254 | 0.00 (0.00, 17917877 | 0.00 (0.00, 29394322 | 0.00 (0.00, 13629937 |
| 36.24 (0.00, 3497684 | 104052576995611530 | 0.45 (0.00, 15478135 | 0.01 (0.00, 15339951 | 3509952562443721.5 | 3670.84 (0.00, 43765 | 23263501269.31 (0.0 | 0.00 (0.00, 10690893 | 0.00 (0.00, 39028659 |
| 0.00 (0.00, 10421091 | 0.13 (0.00, 34716064 | 0.00 (0.00, 37443659 | 0.00 (0.00, 45056964 | 0.00 (0.00, 28510690 | 0.00 (0.00, 18011418 | 0.00 (0.00, 40591800 | 0.00 (0.00, 0.73) | 0.00 (0.00, 18672.42) |
| 11608982839196.32 | 272057155773148850 | 452367009828209.5( | 166475077131.28 (0. | 201582146252067840 | 184044196190705120 | 105805882205543980 | 7684937.76 (0.00, 28 | 9480048.15 (0.00, 10 |
| 0.00 (0.00, 55255439 | 111307.02 (0.00, 467 | 0.00 (0.00, 79829125 | 0.00 (0.00, 38146187 | 62.13 (0.00, 3327582 | 0.00 (0.00, 54681853 | 0.00 (0.00, 28385228 | 0.00 (0.00, 2800430.2 | 0.00 (0.00, 11830219 |
| 10046297.76 (0.00, 8 | 297313332235961170 | 137177321.58 (0.00, | 2580.25 (0.00, 30286 | 192931333975109850 | 1037278703.89 (0.00 | 171547339584864.6( | 2.11 (0.03, 188.98) | 2.78 (0.00, 38246120 |
| Diosmectite | 590242036700167360 | 5.42 (0.00, 28203600 | 0.01 (0.00, 57728881 | 702925166393233.6( | 2769.58 (0.00, 26058 | 2374673024.05 (0.00 | 0.00 (0.00, 25799331 | 0.00 (0.00, 20603297 |
| 0.00 (0.00, 38737556 | Doxycycline | 0.00 (0.00, 53831891 | 0.00 (0.00, 21534360 | 0.00 (0.00, 15421582 | 0.00 (0.00, 12985025 | 0.00 (0.00, 12241276 | 0.00 (0.00, 0.00) | 0.00 (0.00, 26435484 |
| 0.18 (0.00, 21691037 | 209525220142840080 | Glycyrrhiza | 0.00 (0.00, 87711740 | 81030406889873.20 | 56.74 (0.00, 6207405 | 234976443.60 (0.00, | 0.00 (0.00, 17721672 | 0.00 (0.00, 24081971 |
| 78.14 (0.00, 1110167 | 296257361417367140 | 4468.12 (0.00, 14782 | Honey | 637755382519710400 | 81577.96 (0.00, 3356 | 6825972172324.43 ( | 0.00 (0.00, 12641805 | 0.00 (0.00, 14547716 |
| 0.00 (0.00, 15206544 | 2285.60 (0.00, 22022 | 0.00 (0.00, 49785358 | 0.00 (0.00, 11277612 | Laser | 0.00 (0.00, 36815139 | 0.00 (0.00, 19510622 | 0.00 (0.00, 9951.34) | 0.00 (0.00, 21112247 |
| 0.00 (0.00, 22055068 | 391949153997821360 | 0.02 (0.00, 22290805 | 0.00 (0.00, 53211533 | 115049584131465.19 | Minocycline | 720562279.42 (0.00, | 0.00 (0.00, 61741669 | 0.00 (0.00, 10344326 |
| 0.00 (0.00, 40702659 | 473469603.85 (0.00, | 0.00 (0.00, 11914176 | 0.00 (0.00, 32360757 | 385996.09 (0.00, 330 | 0.00 (0.00, 33791706 | Penicillin | 0.00 (0.00, 0.09) | 0.00 (0.00, 30548293 |
| 4644117.45 (0.00, 39 | 125304457334284240 | 66217780.24 (0.00, 3 | 1210.79 (0.00, 15158 | 874619607439387400 | 450965706.27 (0.00, | 69903980918295.03 | Placebo | 1.16 (0.00, 17422983 |
| 19392642.18 (0.00, 1 | 417322800483722700 | 1699347270.69 (0.00 | 374.98 (0.00, 948011 | 335488496831960870 | 3281786041.93 (0.00 | 1743295870329441.8 | 0.86 (0.00, 13943935 | Probiotics |
| 846290194869256.40 | 263966948949634900 | 1984214794461827.5 | 4414807211254.01 ( | 154054238317522700 | 278473588183832630 | 494465214585289730 | 58129989.22 (0.00, 4 | 82639354.19 (0.00, 5 |
| 0.01 (0.00, 59169732 | 143243715429094.06 | 0.06 (0.00, 35781368 | 0.00 (0.00, 11775026 | 1942652597739.67 ( | 0.00 (0.00, 67477018 | 2667555.10 (0.00, 53 | 0.00 (0.00, 11870547 | 0.00 (0.00, 14991748 |
| 0.00 (0.00, 98369996 | 6265418.14 (0.00, 49 | 0.00 (0.00, 75143991 | 0.00 (0.00, 11328819 | 184.34 (0.00, 115343 | 0.00 (0.00, 31104613 | 0.00 (0.00, 30363112 | 0.00 (0.00, 50507170 | 0.00 (0.00, 12275404 |
| 283624755706686570 | 166096846210316620 | 229539135888113050 | 82520788628524.17 | 361264913522821540 | 306706974031233480 | 395340964378205200 | 300661221.92 (0.00, | 31593602259.83 (0.0 |

| | | | |
|---|---|---|---|
| 0.00 (0.00, 58465612 | 124.87 (0.00, 780648 | 19306951629180.37 | 0.00 (0.00, 70672695 |
| 0.00 (0.00, 54321569 | 0.00 (0.00, 10658523 | 2442336.80 (0.00, 23 | 0.00 (0.00, 13156973 |
| 0.00 (0.00, 15290664 | 9264651.40 (0.00, 10 | 32489790475993466 | 0.00 (0.00, 55686587 |
| 0.00 (0.00, 12004464 | 0.00 (0.00, 11565255 | 0.00 (0.00, 13979627 | 0.00 (0.00, 16161415 |
| 0.00 (0.00, 60206393 | 2953.64 (0.00, 11963 | 34485730974180.96 | 0.00 (0.00, 15977221 |
| 0.00 (0.00, 1169.44) | 0.00 (0.00, 1.35) | 0.00 (0.00, 90855809 | 0.00 (0.00, 31654437 |
| 0.01 (0.00, 51096823 | 1025608602205931.4 | 40367988301283230 | 0.00 (0.00, 24485696 |
| 0.00 (0.00, 35734027 | 0.00 (0.00, 20741168 | 0.21 (0.00, 56993921 | 0.00 (0.00, 11558009 |
| 0.00 (0.00, 58448364 | 367378735.54 (0.00, | 12571685100086012 | 0.00 (0.00, 16838941 |
| 0.00 (0.00, 23331217 | 133.92 (0.00, 170904 | 8316068628224.52 ( | 0.00 (0.00, 51679929 |
| 0.00 (0.00, 1005094.1 | 0.00 (0.00, 65942244 | 0.00 (0.00, 16230468 | 0.00 (0.00, 2622231.3 |
| 0.00 (0.00, 71890506 | 16.26 (0.00, 1585500 | 3920557116019.13 ( | 0.00 (0.00, 47051059 |
| 0.00 (0.00, 52436850 | 1119936.10 (0.00, 77 | 22002927893239337 | 0.00 (0.00, 11415736 |
| 0.00 (0.00, 26149242 | 0.00 (0.00, 14409162 | 0.01 (0.00, 24906544 | 0.00 (0.00, 31748075 |
| 0.00 (0.00, 61925783 | 200.51 (0.00, 254773 | 2684234756549.01 ( | 0.00 (0.00, 72872962 |
| 0.00 (0.00, 13318730 | 0.00 (0.00, 41217800 | 53414.33 (0.00, 8599 | 0.00 (0.00, 24680416 |
| 0.00 (0.00, 25750197 | 160532814.00 (0.00, | 52287722753296660 | 0.00 (0.00, 82319368 |
| 0.00 (0.00, 27581590 | 68756544.56 (0.00, 4 | 22310219200917148 | 0.00 (0.00, 92490932 |
| Silver nitrate | 47353672292945664 | 38467196309905150 | 0.02 (0.00, 27759999 |
| 0.00 (0.00, 27502052 | Triamcinolone | 3574761030.36 (0.00 | 0.00 (0.00, 47059547 |
| 0.00 (0.00, 19258440 | 0.00 (0.00, 25484350 | Triester Glycerol Oxid | 0.00 (0.00, 27100439 |
| 49.39 (0.00, 4101602 | 43865511321584940 | 85928419480467830 | Zinc |

**Inconsistency Factors**

| Cycle | Median (95% CI) |
|---|---|
| Aloe , Chitosan , Placebo , Triamcinolone | 0.06 (-3.99, 3.92) |
| Aloe , Curcumin , Placebo , Triamcinolone | -0.04 (-3.96, 3.89) |
| Aloe , Doxycycline , Placebo , Triamcinolone | -0.04 (-3.80, 3.90) |
| Aloe , Honey , Placebo , Triamcinolone | 0.03 (-3.92, 3.97) |
| Aloe , Laser , Placebo , Triamcinolone | -0.02 (-4.02, 4.01) |
| Aloe , Placebo , Probiotics , Triamcinolone | -0.06 (-4.11, 3.80) |
| Aloe , Honey , Placebo , Silver nitrate , Triamcinolone | -0.06 (-4.01, 3.89) |
| Aloe , Placebo , Triamcinolone | -0.01 (-6.14, 3.81) |
| Aloe , Placebo , Triamcinolone | -0.01 (-6.14, 3.81) |
| Aloe , Placebo , Triamcinolone | -0.01 (-6.14, 3.81) |

**Variance Calculation**

| Parameter | Median (95% CI) |
|---|---|
| Random Effects Standard Deviation | 1.89 (0.04, 3.15) |
| Inconsistency Standard Deviation | 1.62 (0.11, 3.13) |

## Convergence Diagnostics

Convergence is assessed using the Brooks-Gelman-Rubin method. This method compares within-chain and between-chain variance to calculate the Potential Scale Reduction Factor (PSRF). A PSRF close to one indicates approximate convergence has been reached.

| Parameter | PSRF |
|---|---|
| d.Allicin.Placebo | 1.05 |
| d.Aloe.Triamcinolone | 1.46 |
| d.Amlexanox.Clobetasol | 1.01 |
| d.Benzydamine.Doxycycline | 1.05 |
| d.Honey.Silvernitrate | 1.03 |
| d.Placebo.Aloe | 1.21 |
| d.Placebo.Amlexanox | 1.00 |
| d.Placebo.Berberinegelatin | 1.01 |
| d.Placebo.Dexamethasone | 1.00 |
| d.Placebo.Diosmectite | 1.01 |
| d.Placebo.Glycyrrhiza | 1.06 |
| d.Placebo.Minocycline | 1.03 |
| d.Placebo.Penicillin | 1.12 |
| d.Placebo.Zinc | 1.03 |
| d.Triamcinolone.Benzydamine | 1.06 |
| d.Triamcinolone.Chitosan | 1.00 |
| d.Triamcinolone.Curcumin | 1.06 |
| d.Triamcinolone.Honey | 1.02 |
| d.Triamcinolone.Laser | 1.02 |
| d.Triamcinolone.Probiotics | 1.21 |
| d.Triamcinolone.TriesterGlycerolOxide | 1.02 |
| w.Aloe.Placebo.Chitosan.Triamcinolone | 1.01 |
| w.Aloe.Placebo.Curcumin.Triamcinolone | 1.01 |
| w.Aloe.Placebo.Doxycycline.Triamcinolone | 1.00 |
| w.Aloe.Placebo.Honey.Triamcinolone | 1.01 |
| w.Aloe.Placebo.Laser.Triamcinolone | 1.00 |
| w.Aloe.Placebo.Probiotics.Triamcinolone | 1.00 |
| w.Aloe.Placebo.Silvernitrate.Honey.Triamcinolone | 1.00 |
| w.Aloe.Placebo.Triamcinolone | 1.04 |
| w.Aloe.Placebo.Triamcinolone | 1.04 |
| w.Aloe.Placebo.Triamcinolone | 1.04 |
| sd.d | 1.08 |
| sd.w | 1.00 |

Number of chains : 4
Tuning iterations : 20,000
Simulation iterations : 400,000
Thinning interval : 10
Inference samples : 80,000
Variance scaling factor : 2.5

## 3. Node-splitting analysis

### Node-splitting analysis of inconsistency

Inconsistency between direct and indirect estimates was estimated in the Node-Splitting Model. When P values is above 0.05 in a comparison, there is evidence of statistical inconsistency.

| Name | Direct Effect | Indirect Effect | Overall | P-Value |
|---|---|---|---|---|
| Aloe , Triamcinolone | -7.97 (-98.59, 62.61) | -29.79 (-102.07, 29.72) | -33.64 (-111.19, 51.35) | 0.68 |
| Chitosan , Placebo | 15.27 (-37.06, 90.03) | -23.90 (-88.20, 19.82) | 3.17 (-58.49, 57.88) | 0.29 |
| Chitosan , Triamcinolone | -28.60 (-100.11, -1.28) | -8.02 (-115.88, 79.01) | -48.98 (-129.63, -2.47) | 0.64 |
| Curcumin , Placebo | 10.33 (-57.81, 126.83) | 67.85 (2.94, 136.18) | 7.21 (-58.66, 63.56) | 0.39 |
| Curcumin , Triamcinolone | -23.70 (-109.95, 28.63) | -37.13 (-103.98, 99.51) | -45.74 (-126.10, 18.07) | 0.98 |
| Doxycycline , Triamcinolone | -12.94 (-79.78, 55.58) | -77.96 (-142.00, -23.35) | -71.28 (-146.09, -24.16) | 0.11 |
| Honey , Silver nitrate | 9.92 (-69.05, 66.54) | 10.79 (-60.43, 97.38) | 6.08 (-89.64, 105.83) | 0.99 |
| Honey , Triamcinolone | -12.53 (-85.19, 71.54) | -33.08 (-122.74, 53.40) | -25.04 (-124.83, 74.14) | 0.65 |
| Laser , Placebo | 10.72 (-28.23, 76.36) | 17.62 (-63.93, 99.16) | 18.47 (-19.78, 81.38) | 0.98 |
| Laser , Triamcinolone | -16.10 (-79.47, 40.59) | -27.12 (-100.02, 37.27) | -31.34 (-108.31, 38.93) | 0.78 |
| Placebo , Probiotics | -27.06 (-84.79, 27.66) | -48.86 (-134.73, 48.69) | -21.16 (-89.50, 30.03) | 0.7 |
| Placebo , Triamcinolone | -16.95 (-125.56, 40.66) | -56.37 (-155.35, -1.52) | -52.12 (-117.84, -7.89) | 0.57 |
| Probiotics , Triamcinolone | -15.04 (-104.14, 55.00) | -29.65 (-98.36, 100.12) | -29.32 (-112.50, 35.05) | 0.94 |

## 4. Network structure

### Network structure of sensitivity analysis

36 RCTs involving 22 local interventions were included in the sensitivity analysis considering the adverse effect.

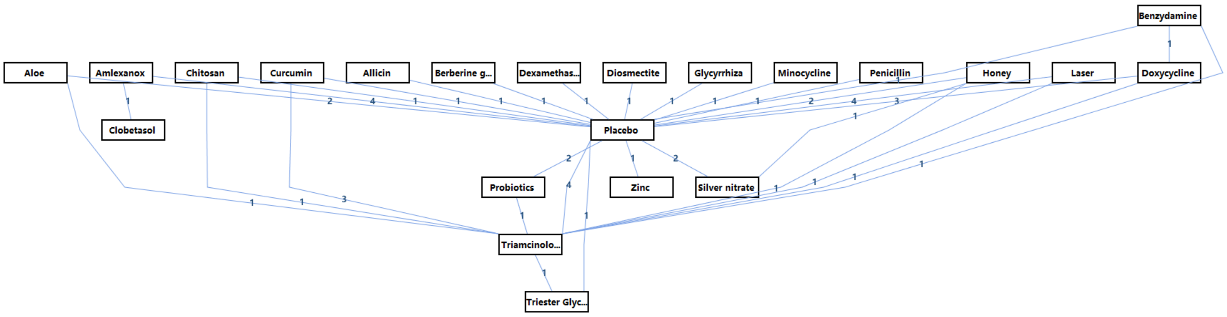

## 5. Pairwise meta-analysis

### Pairwise meta-analysis of adverse effect

Adverse effect were measured by risk difference (RD) and 95% confidence interval (CI).

| Study or Subgroup | Triamcinolone Events | Triamcinolone Total | Aloe Events | Aloe Total | Weight | Risk Difference M-H, Fixed, 95% CI |
|---|---|---|---|---|---|---|
| Bhalang 2013 | 0 | 60 | 0 | 60 | 100.0% | 0.00 [-0.03, 0.03] |
| **Total (95% CI)** | | **60** | | **60** | **100.0%** | **0.00 [-0.03, 0.03]** |
| Total events | 0 | | 0 | | | |

Heterogeneity: Not applicable
Test for overall effect: Z = 0.00 (P = 1.00)

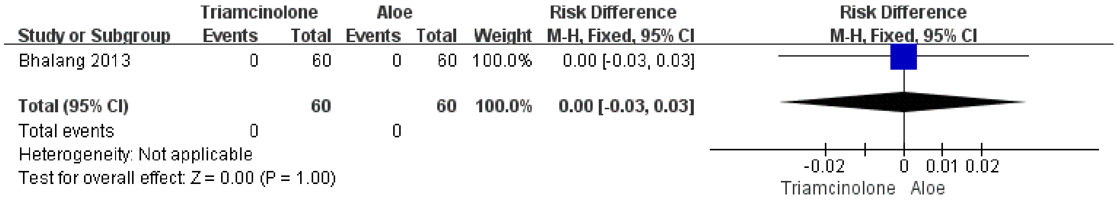


| Study or Subgroup | Placebo Events | Placebo Total | Zinc Events | Zinc Total | Weight | Risk Difference M-H, Fixed, 95% CI |
|---|---|---|---|---|---|---|
| Ghorbani 2020 | 0 | 23 | 0 | 23 | 100.0% | 0.00 [-0.08, 0.08] |
| **Total (95% CI)** | | **23** | | **23** | **100.0%** | **0.00 [-0.08, 0.08]** |
| Total events | 0 | | 0 | | | |

Heterogeneity: Not applicable
Test for overall effect: Z = 0.00 (P = 1.00)

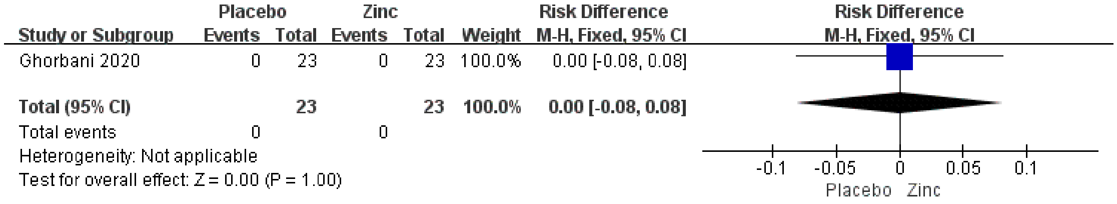


| Study or Subgroup | Placebo Events | Placebo Total | Aloe Events | Aloe Total | Weight | Risk Difference M-H, Fixed, 95% CI |
|---|---|---|---|---|---|---|
| Bhalang 2013 | 0 | 60 | 0 | 60 | 66.7% | 0.00 [-0.03, 0.03] |
| Mansour 2014 | 0 | 30 | 0 | 30 | 33.3% | 0.00 [-0.06, 0.06] |
| **Total (95% CI)** | | **90** | | **90** | **100.0%** | **0.00 [-0.03, 0.03]** |
| Total events | 0 | | 0 | | | |

Heterogeneity: Chi² = 0.00, df = 1 (P = 1.00); I² = 0%
Test for overall effect: Z = 0.00 (P = 1.00)

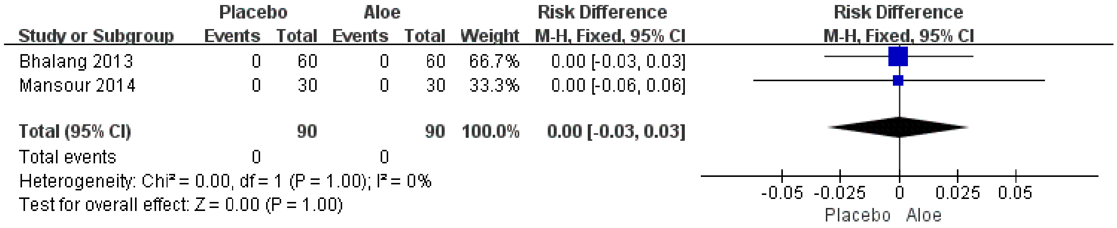


| Study or Subgroup | Placebo Events | Placebo Total | Amlexanox Events | Amlexanox Total | Weight | Risk Difference M-H, Fixed, 95% CI |
|---|---|---|---|---|---|---|
| Bhat 2013 | 0 | 50 | 8 | 50 | 18.0% | -0.16 [-0.27, -0.05] |
| Garnick 1998 | 0 | 14 | 0 | 18 | 5.7% | 0.00 [-0.12, 0.12] |
| Liu 2006 | 1 | 108 | 1 | 104 | 38.1% | -0.00 [-0.03, 0.03] |
| Meng 2009 | 0 | 105 | 0 | 108 | 38.3% | 0.00 [-0.02, 0.02] |
| **Total (95% CI)** | | **277** | | **280** | **100.0%** | **-0.03 [-0.05, -0.00]** |
| Total events | 1 | | 9 | | | |

Heterogeneity: Chi² = 20.44, df = 3 (P = 0.0001); I² = 85%
Test for overall effect: Z = 2.37 (P = 0.02)

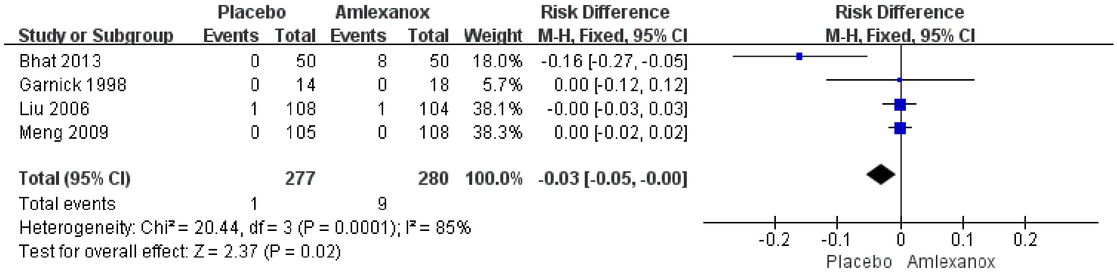


| Study or Subgroup | Triamcinolone Events | Triamcinolone Total | Curcumin Events | Curcumin Total | Weight | Risk Difference M-H, Fixed, 95% CI |
|---|---|---|---|---|---|---|
| Deshmukh 2014 | 0 | 30 | 0 | 30 | 33.7% | 0.00 [-0.06, 0.06] |
| Kia 2020 | 0 | 29 | 0 | 29 | 32.6% | 0.00 [-0.06, 0.06] |
| Raman 2020 | 0 | 30 | 0 | 30 | 33.7% | 0.00 [-0.06, 0.06] |
| **Total (95% CI)** | | **89** | | **89** | **100.0%** | **0.00 [-0.04, 0.04]** |
| Total events | 0 | | 0 | | | |

Heterogeneity: Chi² = 0.00, df = 2 (P = 1.00); I² = 0%
Test for overall effect: Z = 0.00 (P = 1.00)

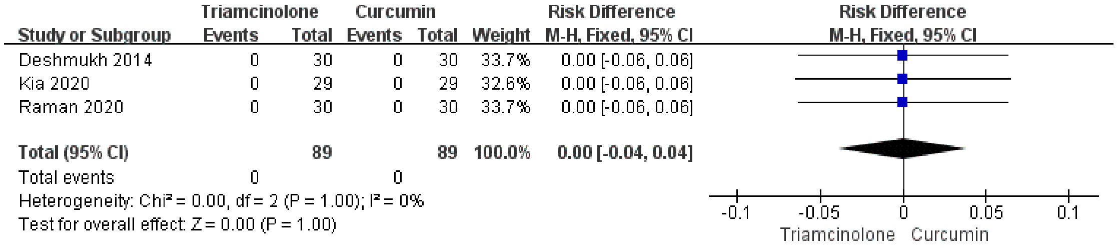


| Study or Subgroup | Placebo Events | Placebo Total | Doxycycline Events | Doxycycline Total | Weight | Risk Difference M-H, Random, 95% CI |
|---|---|---|---|---|---|---|
| Sharma 2018 | 0 | 10 | 0 | 10 | 31.8% | 0.00 [-0.17, 0.17] |
| Skulason 2009 | 0 | 24 | 1 | 25 | 37.5% | -0.04 [-0.15, 0.07] |
| Vijayabala 2013 | 0 | 25 | 8 | 25 | 30.6% | -0.32 [-0.51, -0.13] |
| **Total (95% CI)** | | **59** | | **60** | **100.0%** | **-0.11 [-0.31, 0.09]** |
| Total events | 0 | | 9 | | | |

Heterogeneity: Tau² = 0.03; Chi² = 10.28, df = 2 (P = 0.006); I² = 81%
Test for overall effect: Z = 1.10 (P = 0.27)

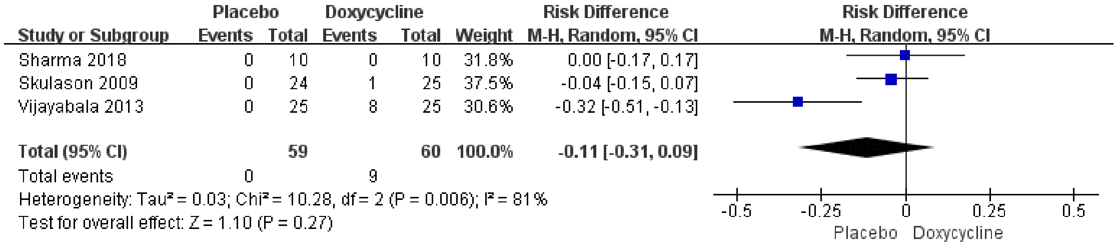

| Study or Subgroup | Placebo Events | Placebo Total | Honey Events | Honey Total | Weight | Risk Difference M-H, Fixed, 95% CI |
|---|---|---|---|---|---|---|
| El-Haddad 2014 | 0 | 56 | 0 | 67 | 70.9% | 0.00 [-0.03, 0.03] |
| Rodríguez-Archilla 2017 | 0 | 25 | 0 | 25 | 29.1% | 0.00 [-0.07, 0.07] |
| **Total (95% CI)** | | **81** | | **92** | **100.0%** | **0.00 [-0.03, 0.03]** |
| Total events | 0 | | 0 | | | |

Heterogeneity: Chi² = 0.00, df = 1 (P = 1.00); I² = 0%
Test for overall effect: Z = 0.00 (P = 1.00)

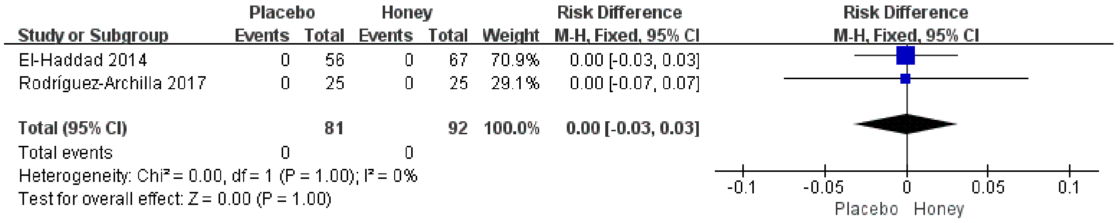


| Study or Subgroup | Placebo Events | Placebo Total | Laser Events | Laser Total | Weight | Risk Difference M-H, Fixed, 95% CI |
|---|---|---|---|---|---|---|
| Albrektson 2014 | 0 | 20 | 0 | 20 | 23.5% | 0.00 [-0.09, 0.09] |
| Yilmaz 2017 | 0 | 40 | 0 | 40 | 47.1% | 0.00 [-0.05, 0.05] |
| Zand 2009 | 0 | 15 | 0 | 15 | 17.6% | 0.00 [-0.12, 0.12] |
| Zand 2012 | 0 | 10 | 0 | 10 | 11.8% | 0.00 [-0.17, 0.17] |
| **Total (95% CI)** | | **85** | | **85** | **100.0%** | **0.00 [-0.05, 0.05]** |
| Total events | 0 | | 0 | | | |

Heterogeneity: Chi² = 0.00, df = 3 (P = 1.00); I² = 0%
Test for overall effect: Z = 0.00 (P = 1.00)

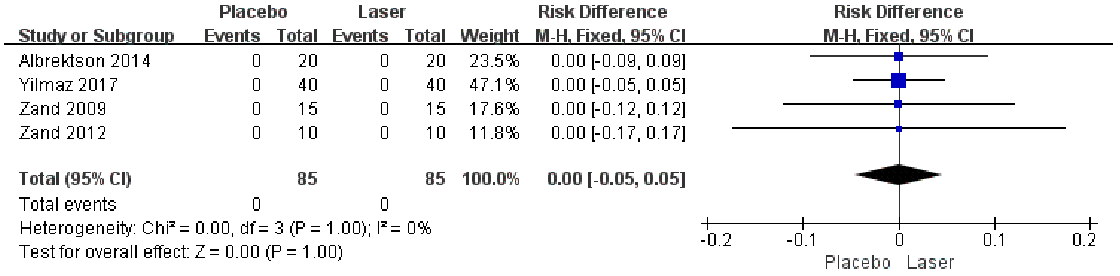


| Study or Subgroup | Placebo Events | Placebo Total | Probiotics Events | Probiotics Total | Weight | Risk Difference M-H, Fixed, 95% CI |
|---|---|---|---|---|---|---|
| Pedersen 2020 | 0 | 10 | 0 | 10 | 40.0% | 0.00 [-0.17, 0.17] |
| Trinchieri 2011 | 0 | 15 | 0 | 15 | 60.0% | 0.00 [-0.12, 0.12] |
| **Total (95% CI)** | | **25** | | **25** | **100.0%** | **0.00 [-0.11, 0.11]** |
| Total events | 0 | | 0 | | | |

Heterogeneity: Chi² = 0.00, df = 1 (P = 1.00); I² = 0%
Test for overall effect: Z = 0.00 (P = 1.00)

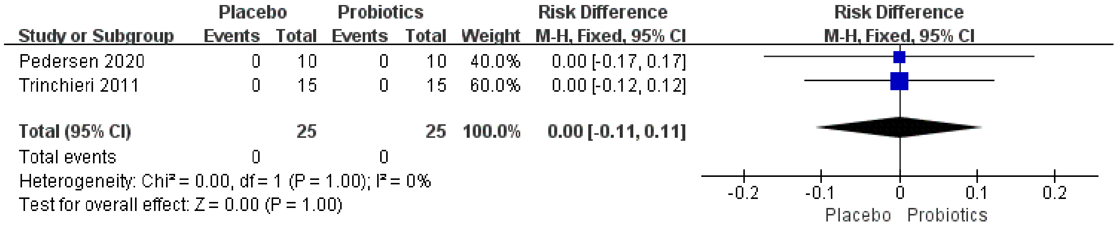


| Study or Subgroup | Placebo Events | Placebo Total | Silver nitrate Events | Silver nitrate Total | Weight | Risk Difference M-H, Fixed, 95% CI |
|---|---|---|---|---|---|---|
| Rodríguez-Archilla 2017 | 0 | 25 | 0 | 25 | 43.6% | 0.00 [-0.07, 0.07] |
| Soylu 2014 | 0 | 30 | 0 | 35 | 56.4% | 0.00 [-0.06, 0.06] |
| **Total (95% CI)** | | **55** | | **60** | **100.0%** | **0.00 [-0.05, 0.05]** |
| Total events | 0 | | 0 | | | |

Heterogeneity: Chi² = 0.00, df = 1 (P = 1.00); I² = 0%
Test for overall effect: Z = 0.00 (P = 1.00)

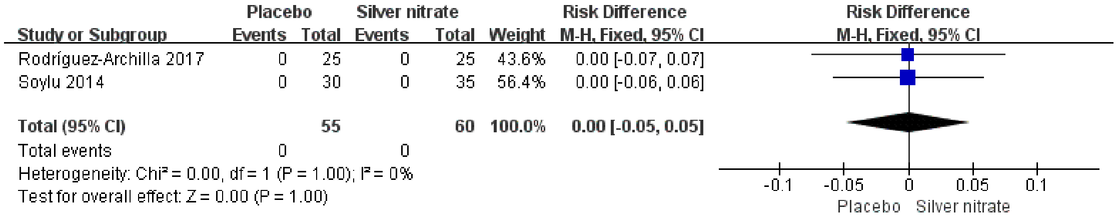


| Study or Subgroup | Placebo Events | Placebo Total | Triamcinolone Events | Triamcinolone Total | Weight | Risk Difference M-H, Fixed, 95% CI |
|---|---|---|---|---|---|---|
| Bhalang 2013 | 0 | 60 | 0 | 60 | 33.6% | 0.00 [-0.03, 0.03] |
| El-Haddad 2014 | 0 | 56 | 0 | 57 | 31.7% | 0.00 [-0.03, 0.03] |
| Ofluoglu 2017 | 0 | 51 | 0 | 53 | 29.1% | 0.00 [-0.04, 0.04] |
| Sharma 2018 | 0 | 10 | 0 | 10 | 5.6% | 0.00 [-0.17, 0.17] |
| **Total (95% CI)** | | **177** | | **180** | **100.0%** | **0.00 [-0.02, 0.02]** |
| Total events | 0 | | 0 | | | |

Heterogeneity: Chi² = 0.00, df = 3 (P = 1.00); I² = 0%
Test for overall effect: Z = 0.00 (P = 1.00)

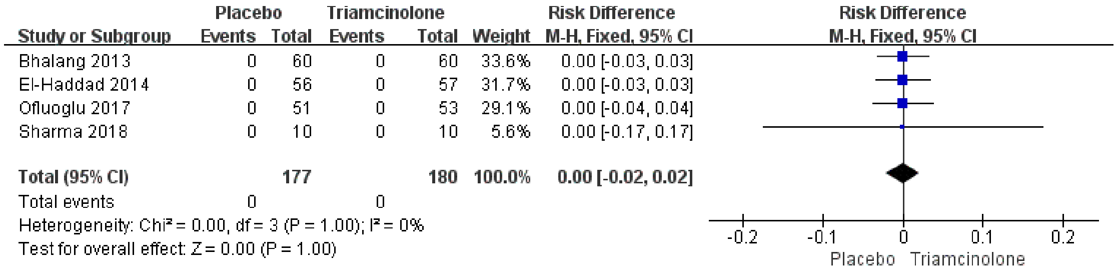


| Study or Subgroup | Placebo Events | Placebo Total | Allicin Events | Allicin Total | Weight | Risk Difference M-H, Fixed, 95% CI |
|---|---|---|---|---|---|---|
| Jiang 2012 | 0 | 48 | 0 | 48 | 100.0% | 0.00 [-0.04, 0.04] |
| **Total (95% CI)** | | **48** | | **48** | **100.0%** | **0.00 [-0.04, 0.04]** |
| Total events | 0 | | 0 | | | |

Heterogeneity: Not applicable
Test for overall effect: Z = 0.00 (P = 1.00)

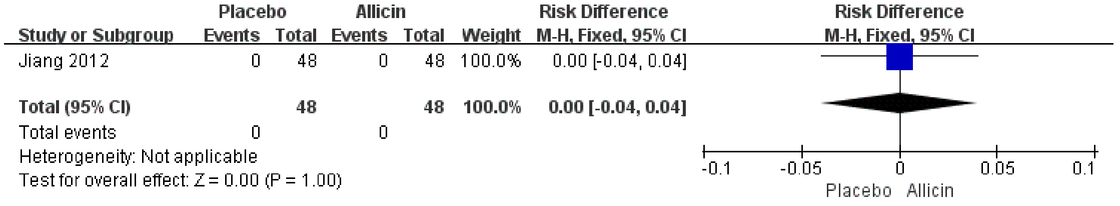


| Study or Subgroup | Clobetasol Events | Clobetasol Total | Amlexanox Events | Amlexanox Total | Weight | Risk Difference M-H, Fixed, 95% CI |
|---|---|---|---|---|---|---|
| Rodríguez 2007 | 0 | 48 | 0 | 48 | 100.0% | 0.00 [-0.04, 0.04] |
| **Total (95% CI)** | | **48** | | **48** | **100.0%** | **0.00 [-0.04, 0.04]** |
| Total events | 0 | | 0 | | | |

Heterogeneity: Not applicable
Test for overall effect: Z = 0.00 (P = 1.00)

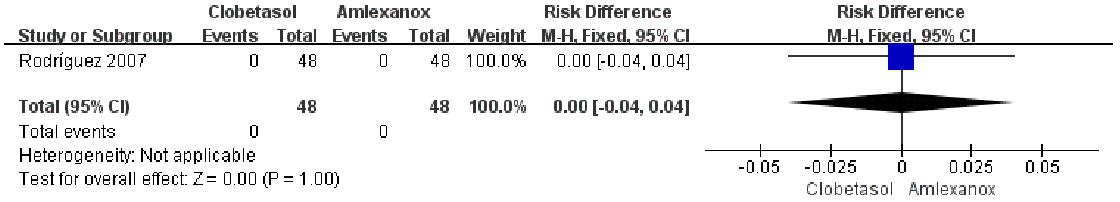

| Study or Subgroup | Placebo Events | Placebo Total | Benzydamine Events | Benzydamine Total | Weight | Risk Difference M-H, Fixed, 95% CI |
|---|---|---|---|---|---|---|
| Sharma 2018 | 0 | 10 | 0 | 10 | 100.0% | 0.00 [-0.17, 0.17] |
| **Total (95% CI)** | | **10** | | **10** | **100.0%** | **0.00 [-0.17, 0.17]** |
| Total events | 0 | | 0 | | | |
| Heterogeneity: Not applicable | | | | | | |
| Test for overall effect: Z = 0.00 (P = 1.00) | | | | | | |

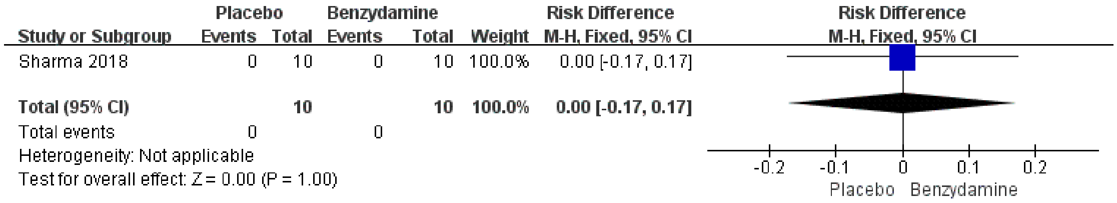


| Study or Subgroup | Triamcinolone Events | Triamcinolone Total | Benzydamine Events | Benzydamine Total | Weight | Risk Difference M-H, Fixed, 95% CI |
|---|---|---|---|---|---|---|
| Sharma 2018 | 0 | 10 | 0 | 10 | 100.0% | 0.00 [-0.17, 0.17] |
| **Total (95% CI)** | | **10** | | **10** | **100.0%** | **0.00 [-0.17, 0.17]** |
| Total events | 0 | | 0 | | | |
| Heterogeneity: Not applicable | | | | | | |
| Test for overall effect: Z = 0.00 (P = 1.00) | | | | | | |

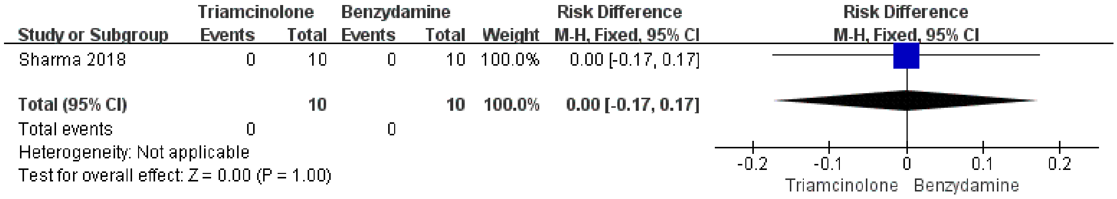


| Study or Subgroup | Doxycycline Events | Doxycycline Total | Benzydamine Events | Benzydamine Total | Weight | Risk Difference M-H, Fixed, 95% CI |
|---|---|---|---|---|---|---|
| Sharma 2018 | 0 | 10 | 0 | 10 | 100.0% | 0.00 [-0.17, 0.17] |
| **Total (95% CI)** | | **10** | | **10** | **100.0%** | **0.00 [-0.17, 0.17]** |
| Total events | 0 | | 0 | | | |
| Heterogeneity: Not applicable | | | | | | |
| Test for overall effect: Z = 0.00 (P = 1.00) | | | | | | |

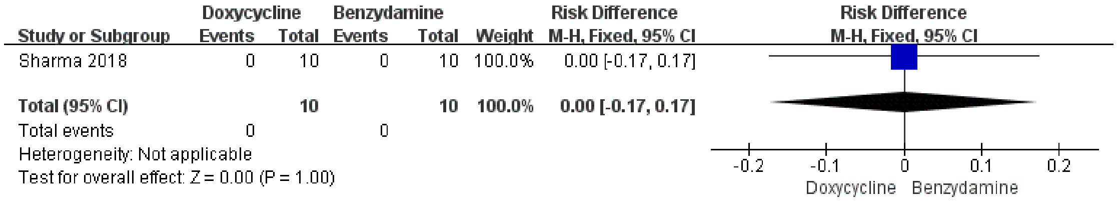


| Study or Subgroup | Placebo Events | Placebo Total | Berberine gelatin Events | Berberine gelatin Total | Weight | Risk Difference M-H, Fixed, 95% CI |
|---|---|---|---|---|---|---|
| Jiang and Zhu 2013 | 0 | 42 | 0 | 42 | 100.0% | 0.00 [-0.05, 0.05] |
| **Total (95% CI)** | | **42** | | **42** | **100.0%** | **0.00 [-0.05, 0.05]** |
| Total events | 0 | | 0 | | | |
| Heterogeneity: Not applicable | | | | | | |
| Test for overall effect: Z = 0.00 (P = 1.00) | | | | | | |

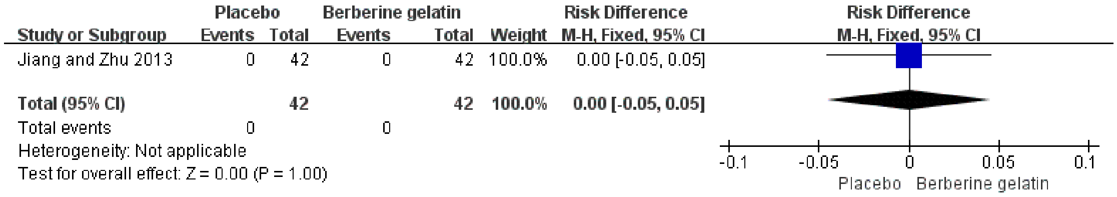


| Study or Subgroup | Placebo Events | Placebo Total | Chitosan Events | Chitosan Total | Weight | Risk Difference M-H, Fixed, 95% CI |
|---|---|---|---|---|---|---|
| Shao 2020 | 0 | 32 | 0 | 34 | 100.0% | 0.00 [-0.06, 0.06] |
| **Total (95% CI)** | | **32** | | **34** | **100.0%** | **0.00 [-0.06, 0.06]** |
| Total events | 0 | | 0 | | | |
| Heterogeneity: Not applicable | | | | | | |
| Test for overall effect: Z = 0.00 (P = 1.00) | | | | | | |

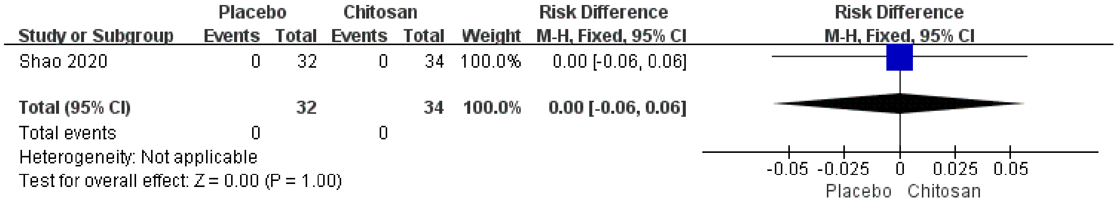


| Study or Subgroup | Triamcinolone Events | Triamcinolone Total | Chitosan Events | Chitosan Total | Weight | Risk Difference M-H, Fixed, 95% CI |
|---|---|---|---|---|---|---|
| Rahmani 2018 | 0 | 20 | 1 | 20 | 100.0% | -0.05 [-0.18, 0.08] |
| **Total (95% CI)** | | **20** | | **20** | **100.0%** | **-0.05 [-0.18, 0.08]** |
| Total events | 0 | | 1 | | | |
| Heterogeneity: Not applicable | | | | | | |
| Test for overall effect: Z = 0.77 (P = 0.44) | | | | | | |

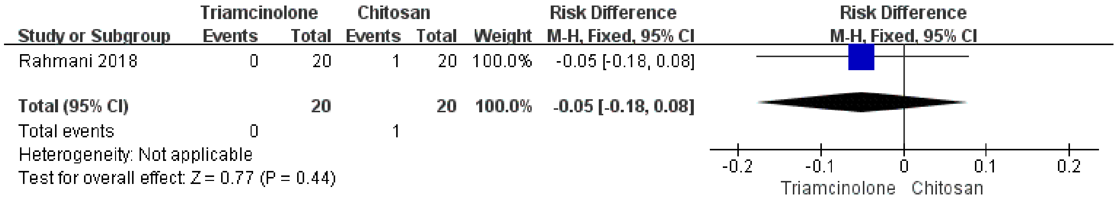


| Study or Subgroup | Placebo Events | Placebo Total | Curcumin Events | Curcumin Total | Weight | Risk Difference M-H, Fixed, 95% CI |
|---|---|---|---|---|---|---|
| Manifar 2012 | 0 | 29 | 0 | 28 | 100.0% | 0.00 [-0.07, 0.07] |
| **Total (95% CI)** | | **29** | | **28** | **100.0%** | **0.00 [-0.07, 0.07]** |
| Total events | 0 | | 0 | | | |
| Heterogeneity: Not applicable | | | | | | |
| Test for overall effect: Z = 0.00 (P = 1.00) | | | | | | |

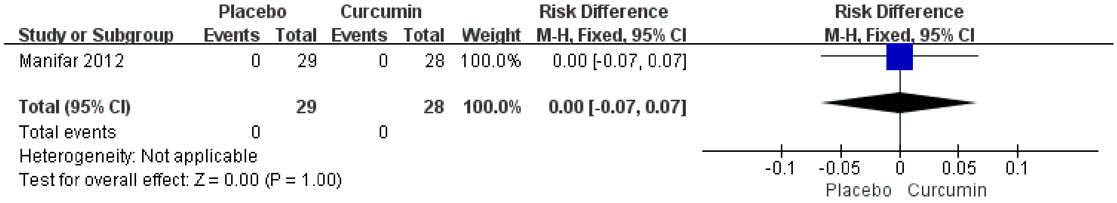

| Study or Subgroup | Placebo Events | Placebo Total | Dexamethasone Events | Dexamethasone Total | Weight | Risk Difference M-H, Fixed, 95% CI |
|---|---|---|---|---|---|---|
| Liu 2012 | 8 | 120 | 4 | 120 | 100.0% | 0.03 [-0.02, 0.09] |
| **Total (95% CI)** | | **120** | | **120** | **100.0%** | **0.03 [-0.02, 0.09]** |
| Total events | 8 | | 4 | | | |

Heterogeneity: Not applicable
Test for overall effect: Z = 1.19 (P = 0.23)

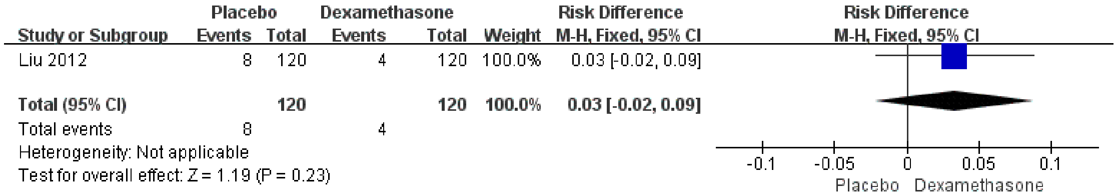


| Study or Subgroup | Placebo Events | Placebo Total | Diosmectite Events | Diosmectite Total | Weight | Risk Difference M-H, Fixed, 95% CI |
|---|---|---|---|---|---|---|
| Jiang and Yang 2013 | 0 | 30 | 0 | 35 | 100.0% | 0.00 [-0.06, 0.06] |
| **Total (95% CI)** | | **30** | | **35** | **100.0%** | **0.00 [-0.06, 0.06]** |
| Total events | 0 | | 0 | | | |

Heterogeneity: Not applicable
Test for overall effect: Z = 0.00 (P = 1.00)

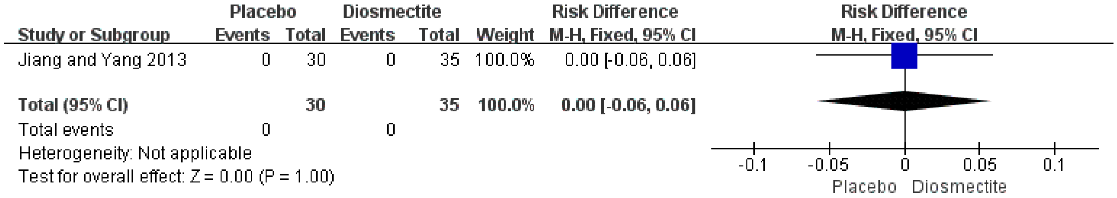


| Study or Subgroup | Triamcinolone Events | Triamcinolone Total | Doxycycline Events | Doxycycline Total | Weight | Risk Difference M-H, Fixed, 95% CI |
|---|---|---|---|---|---|---|
| Sharma 2018 | 0 | 10 | 0 | 10 | 100.0% | 0.00 [-0.17, 0.17] |
| **Total (95% CI)** | | **10** | | **10** | **100.0%** | **0.00 [-0.17, 0.17]** |
| Total events | 0 | | 0 | | | |

Heterogeneity: Not applicable
Test for overall effect: Z = 0.00 (P = 1.00)

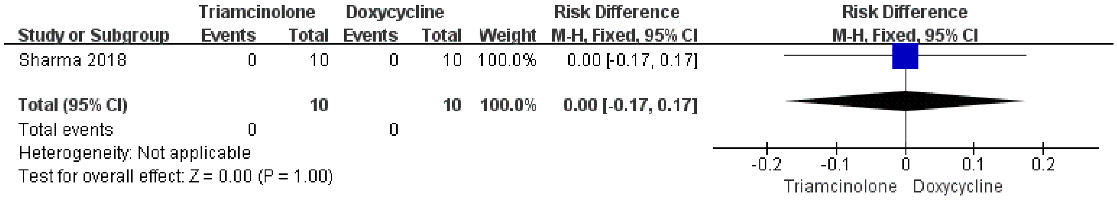


| Study or Subgroup | Placebo Events | Placebo Total | Glycyrrhiza Events | Glycyrrhiza Total | Weight | Risk Difference M-H, Fixed, 95% CI |
|---|---|---|---|---|---|---|
| Raeesi 2015 | 0 | 20 | 0 | 20 | 100.0% | 0.00 [-0.09, 0.09] |
| **Total (95% CI)** | | **20** | | **20** | **100.0%** | **0.00 [-0.09, 0.09]** |
| Total events | 0 | | 0 | | | |

Heterogeneity: Not applicable
Test for overall effect: Z = 0.00 (P = 1.00)

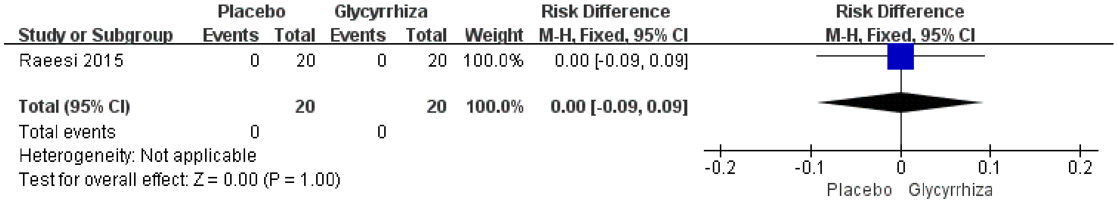


| Study or Subgroup | Silver nitrate Events | Silver nitrate Total | Honey Events | Honey Total | Weight | Risk Difference M-H, Fixed, 95% CI |
|---|---|---|---|---|---|---|
| Rodríguez-Archilla 2017 | 0 | 25 | 0 | 25 | 100.0% | 0.00 [-0.07, 0.07] |
| **Total (95% CI)** | | **25** | | **25** | **100.0%** | **0.00 [-0.07, 0.07]** |
| Total events | 0 | | 0 | | | |

Heterogeneity: Not applicable
Test for overall effect: Z = 0.00 (P = 1.00)

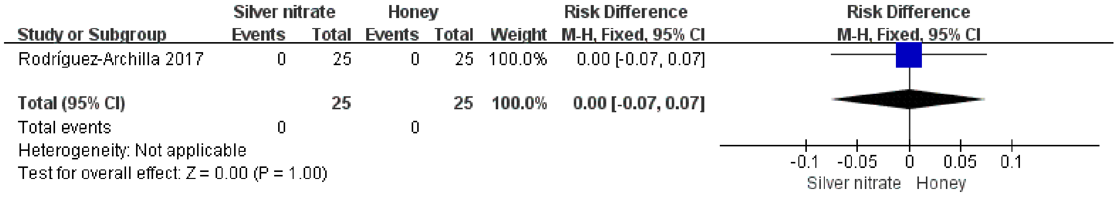


| Study or Subgroup | Triamcinolone Events | Triamcinolone Total | Honey Events | Honey Total | Weight | Risk Difference M-H, Fixed, 95% CI |
|---|---|---|---|---|---|---|
| El-Haddad 2014 | 0 | 57 | 0 | 67 | 100.0% | 0.00 [-0.03, 0.03] |
| **Total (95% CI)** | | **57** | | **67** | **100.0%** | **0.00 [-0.03, 0.03]** |
| Total events | 0 | | 0 | | | |

Heterogeneity: Not applicable
Test for overall effect: Z = 0.00 (P = 1.00)

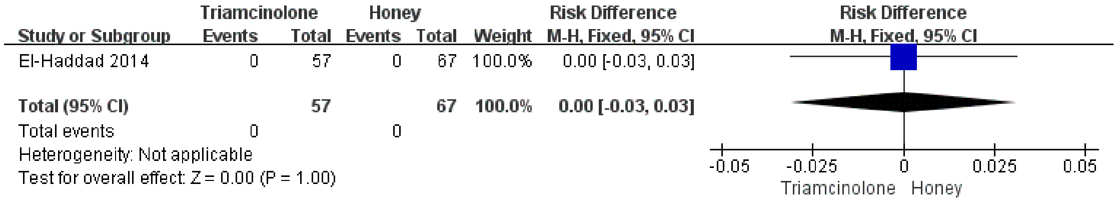


| Study or Subgroup | Triamcinolone Events | Triamcinolone Total | Laser Events | Laser Total | Weight | Risk Difference M-H, Fixed, 95% CI |
|---|---|---|---|---|---|---|
| Tezel 2009 | 0 | 10 | 0 | 10 | 100.0% | 0.00 [-0.17, 0.17] |
| **Total (95% CI)** | | **10** | | **10** | **100.0%** | **0.00 [-0.17, 0.17]** |
| Total events | 0 | | 0 | | | |

Heterogeneity: Not applicable
Test for overall effect: Z = 0.00 (P = 1.00)

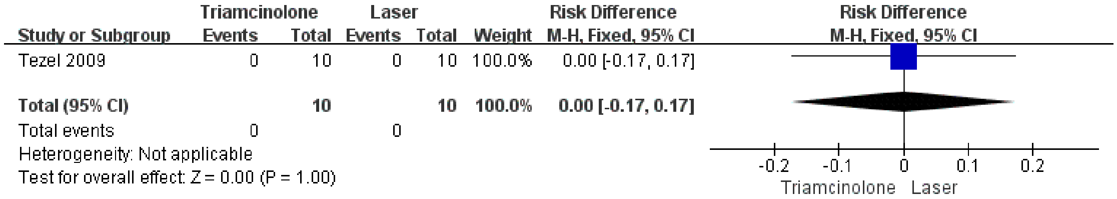

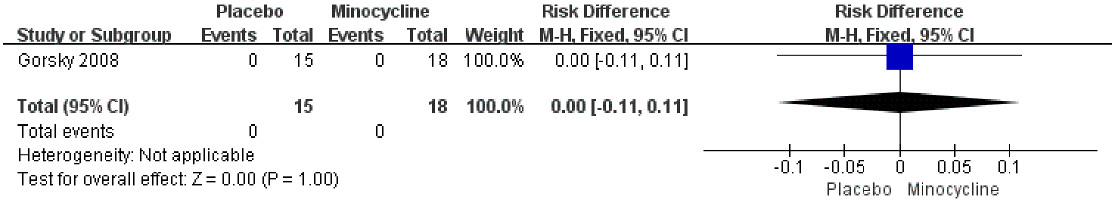


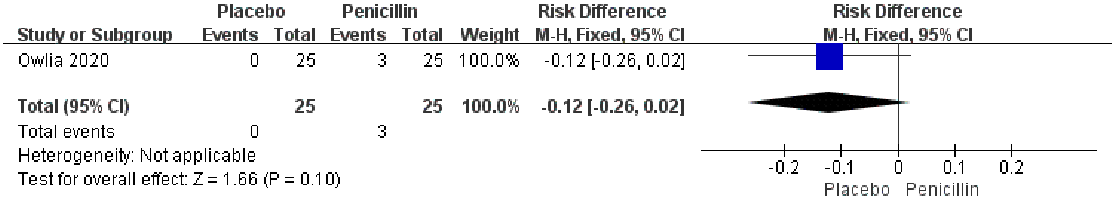


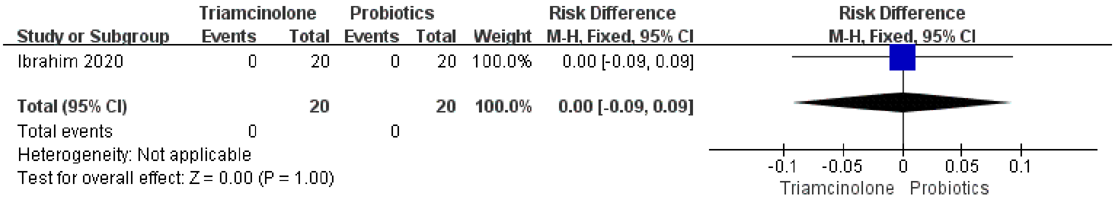


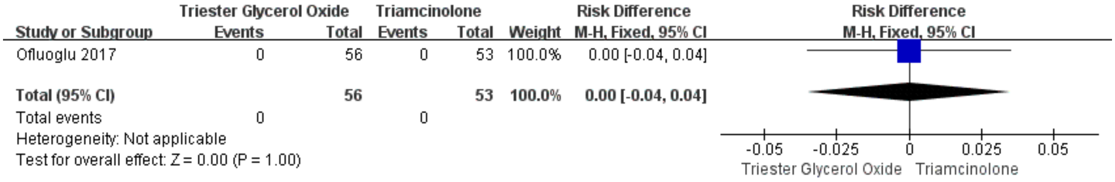


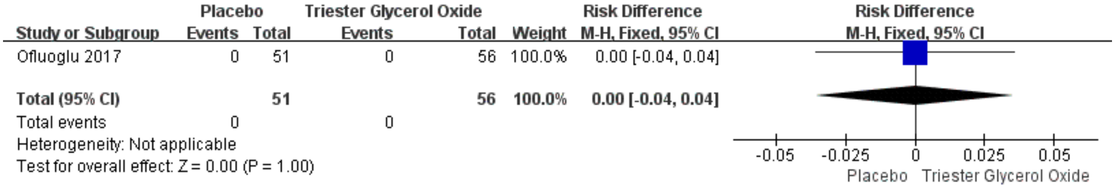


| Comparison | NO. of included study | Heterogeneity ($I^2$) | Combined effect [RD (95%CI)] |
|---|---|---|---|
| Allicin vs | | | |
| Placebo | 1 | NA | 0.00 (-0.04, 0.04) |
| Aloe vs | | | |
| Placebo | 2 | 0% | 0.00 (-0.03,0.03) |
| Triamcinolone | 1 | NA | 0.00 (-0.03, 0.03) |
| Amlexanox vs | | | |
| Placebo | 4 | 85% | -0.03 (-0.05, -0.00) |
| Clobetasol | 1 | NA | 0.00 (-0.04, 0.04) |
| Benzydamine vs | | | |
| Placebo | 1 | NA | 0.00 (-0.17, 0.17) |
| Doxycycline | 1 | NA | 0.00 (-0.17, 0.17) |
| Triamcinolone | 1 | NA | 0.00 (-0.17, 0.17) |
| Berberine gelatin vs | | | |
| Placebo | 1 | NA | 0.00 (-0.05, 0.05) |
| Chitosan vs | | | |

| | | | |
|---|---|---|---|
| Placebo | 1 | NA | 0.00 (-0.06, 0.06) |
| Triamcinolone | 1 | NA | -0.05 (-0.18, 0.08) |
| Curcumin vs | | | |
| Triamcinolone | 3 | 0% | 0.00 (-0.04,0.04) |
| Placebo | 1 | NA | 0.00 (-0.07, 0.07) |
| Dexamethasone vs | | | |
| Placebo | 1 | NA | 0.03 (-0.02, 0.09) |
| Diosmectite vs | | | |
| Placebo | 1 | NA | 0.00 (-0.06, 0.06) |
| Doxycycline vs | | | |
| Placebo | 3 | 81% | -0.11 (-0.31, 0.09) |
| Triamcinolone | 1 | NA | 0.00 (-0.17, 0.17) |
| Glycyrrhiza vs | | | |
| Placebo | 1 | NA | 0.00 (-0.09, 0.09) |
| Honey | | | |
| Placebo | 2 | 0% | 0.00 (-0.03, 0.03) |
| Silver nitrate | 1 | NA | 0.00 (-0.07, 0.07) |
| Triamcinolone | 1 | NA | 0.00 (-0.03, 0.03) |
| Laser vs | | | |
| Placebo | 4 | 0% | 0.00 (-0.05, 0.05) |
| Triamcinolone | 1 | NA | 0.00 (-0.17, 0.17) |
| Minocycline | | | |
| Placebo | 1 | NA | 0.00 (-0.11, 0.11) |
| Penicillin | | | |
| Placebo | 1 | NA | -0.12 (-0.26, 0.02) |
| Probiotics vs | | | |
| Placebo | 2 | 0% | 0.00 (-0.11, 0.11) |
| Triamcinolone | 1 | NA | 0.00 (-0.09, 0.09) |
| Silver nitrate vs | | | |
| Placebo | 2 | 0% | 0.00 (-0.05, 0.05) |
| Triamcinolone vs | | | |
| Placebo | 4 | 0% | 0.00 (-0.02, 0.02) |
| Triester Glycerol Oxide | 1 | NA | 0.00 (-0.04, 0.04) |
| Triester Glycerol Oxide vs | | | |
| Placebo | 1 | NA | 0.00 (-0.04, 0.04) |
| Zinc vs | | | |
| Placebo | 1 | NA | 0.00 (-0.08, 0.08) |

RD, risk difference; CI, confidence interval; NA, not applicable.

Hematologic values

4 RCTs included in the study reported on the blood levels of the intervention drug or blood laboratory findings.

| Interventions | Total | Hematologic values |
|---|---|---|
| Dexamethasone | 114 | Blood level<0.502 ng/mL |
| Aloe | 60 | No significant differences between the blood test values before and after 7 days of application |
| Allicin | 48 | None of the hematologic values at day 6 were considered clinically abnormal |
| Amlexanox | 108 | None of the hematologic values were considered clinically abnormal |

**Chapter S5 Relapse**

4 included articles described the role of interventions on RAS recurrence. Because different criteria were used for evaluation, only descriptive studies were performed.

| Interventions | Total | Relapse |
|---|---|---|
| Probiotics | Adult Group: 30<br>Children group :30 | Adult Group (Outbreak frequency/6 months)<br>Probiotics: 3.33 (0.64)<br>Placebo: 3.65 (0.32)<br>Children group (Outbreak frequency/6 months)<br>Probiotics: 2.65 (0.54)<br>Placebo: 3.65 (0.62) |
| Chlorhexidine | 38 | Total ulcer numbers (6 weeks):<br>Chlorhexidine: 7.54±6.52<br>Placebo: 8.32±5.52<br>Interval between ulcers (6 weeks):<br>Chlorhexidine: 7.26±8.61<br>Placebo:3.86±2.05 |
| Benzydamine | 18 | Number of new ulcers (3 months)<br>Benzydamine: 7 (2-33)<br>Placebo: 8 (2-20) |
| Chlorhexidine | 18 | Number of new ulcers (3 months)<br>Chlorhexidine: 6.5 (3-20)<br>Placebo: 8 (2-20) |
| Triamcinolone | 26 | No. of new ulcers (8 months)<br>Placebo: 7.81<br>Triamcinolone acetonide in orabase : 7.00<br>Triamcinolone acetonide in watery base : 6.42 |

**FigureS1 Risk of bias graph**

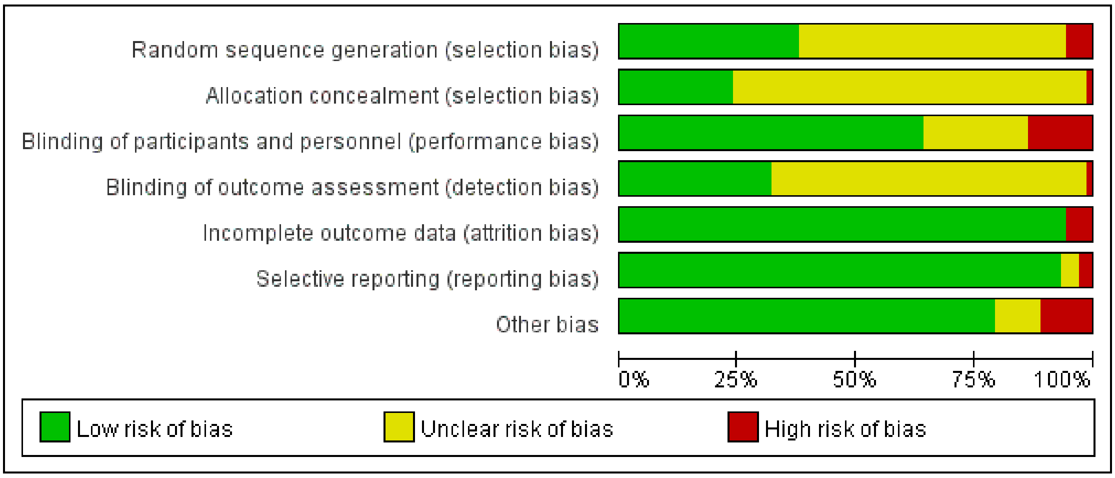

# Figure S2 Risk of bias summary for individual studies

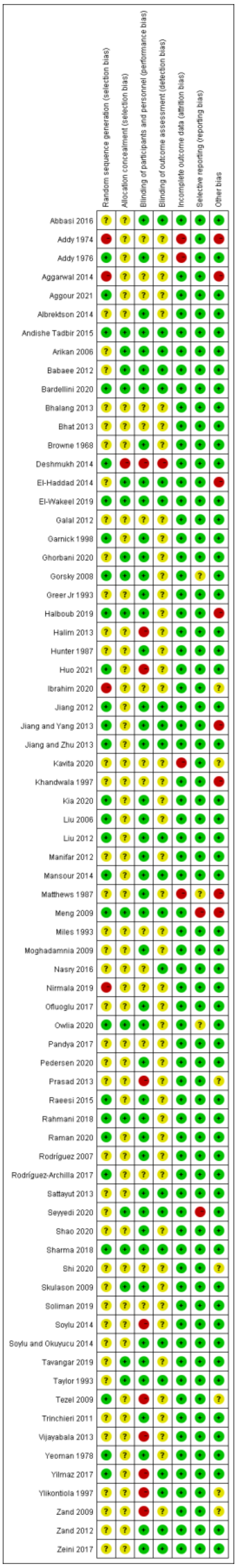

# Funnel plots

## Figure S3 Funnel plot for healing effect

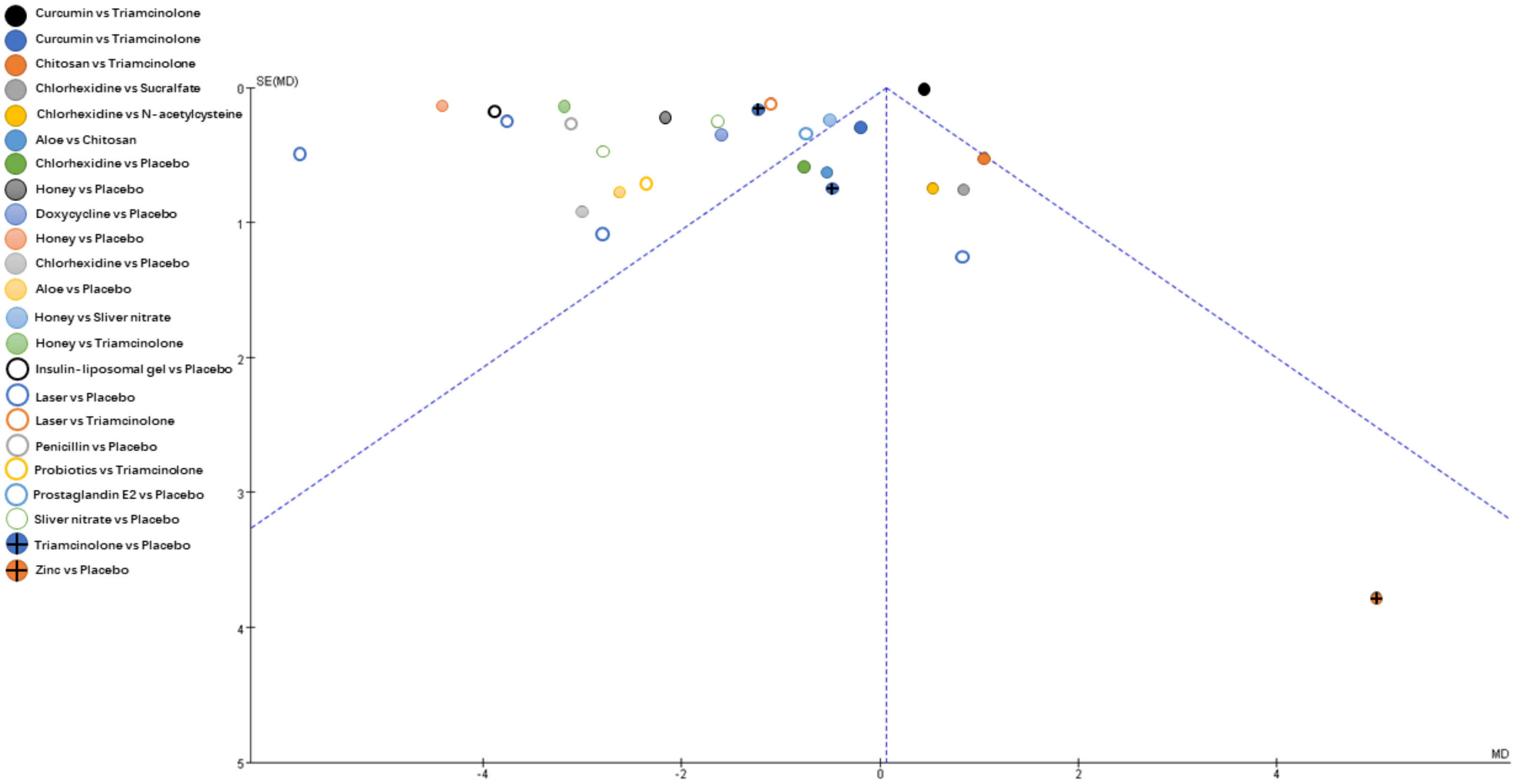

Figure S4 Funnel plot for size-reducing effect

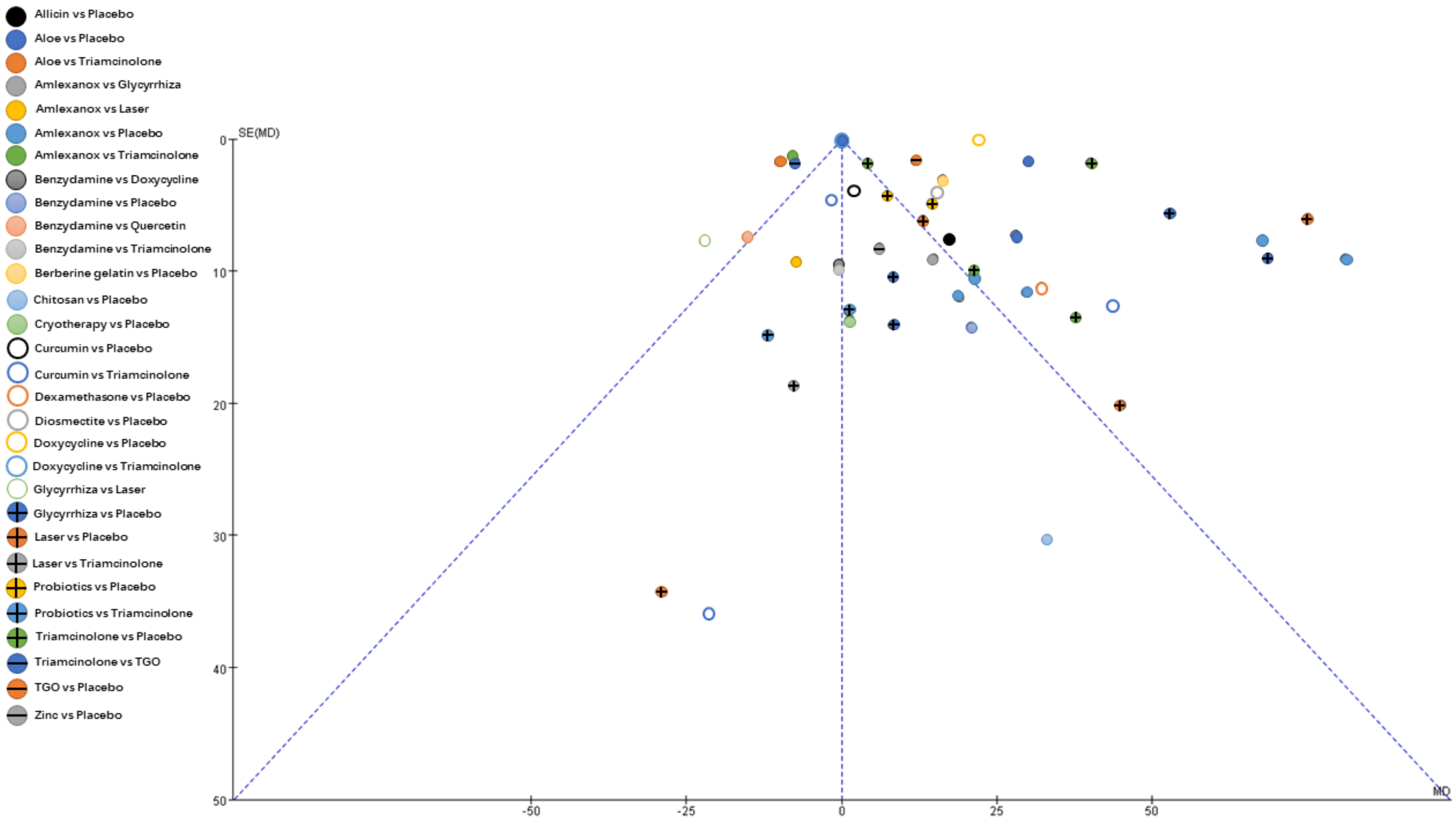

Figure S5 Funnel plot for symptom-reducing effect

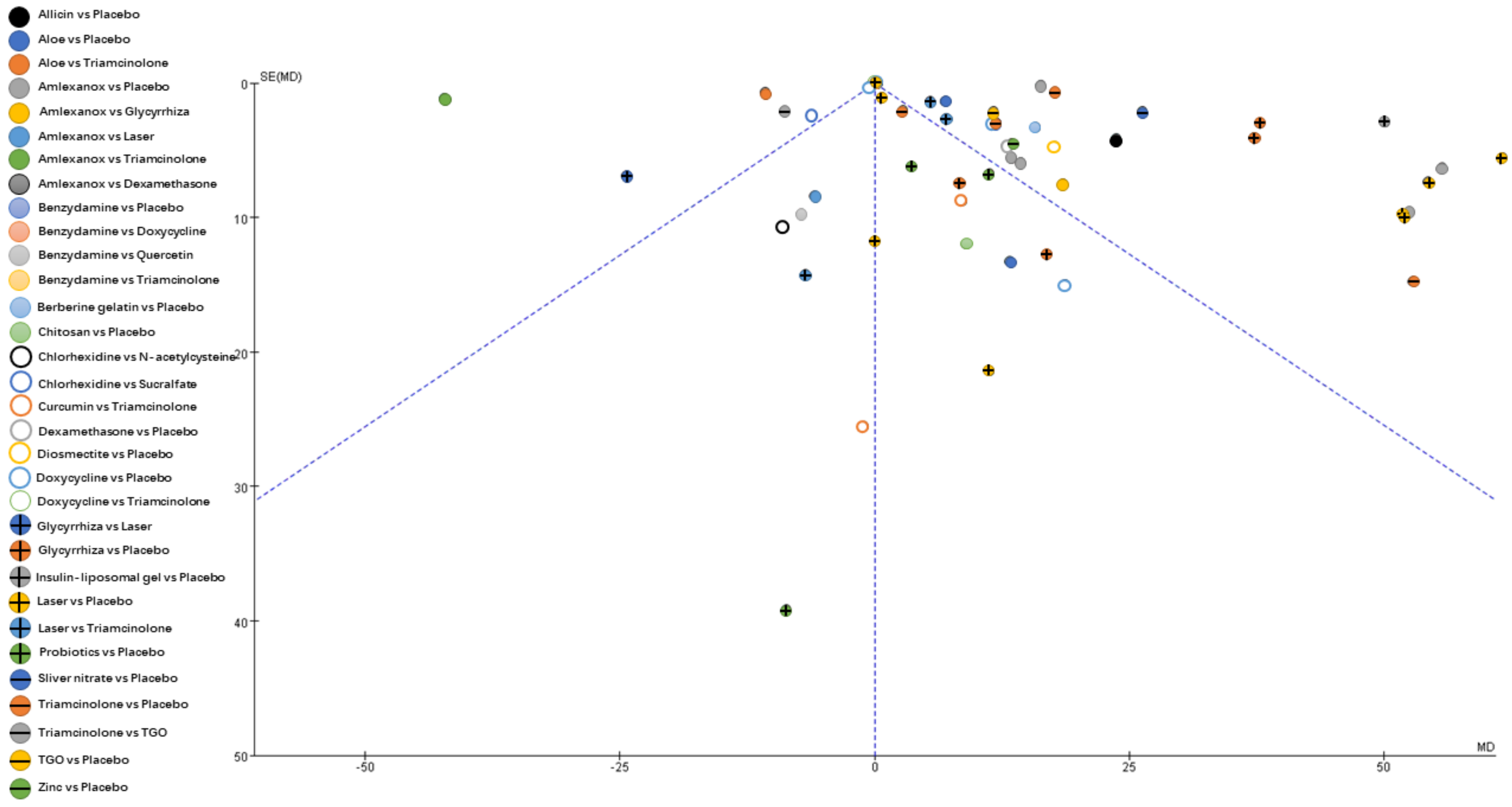

Figure S6 Funnel plot for adverse effect

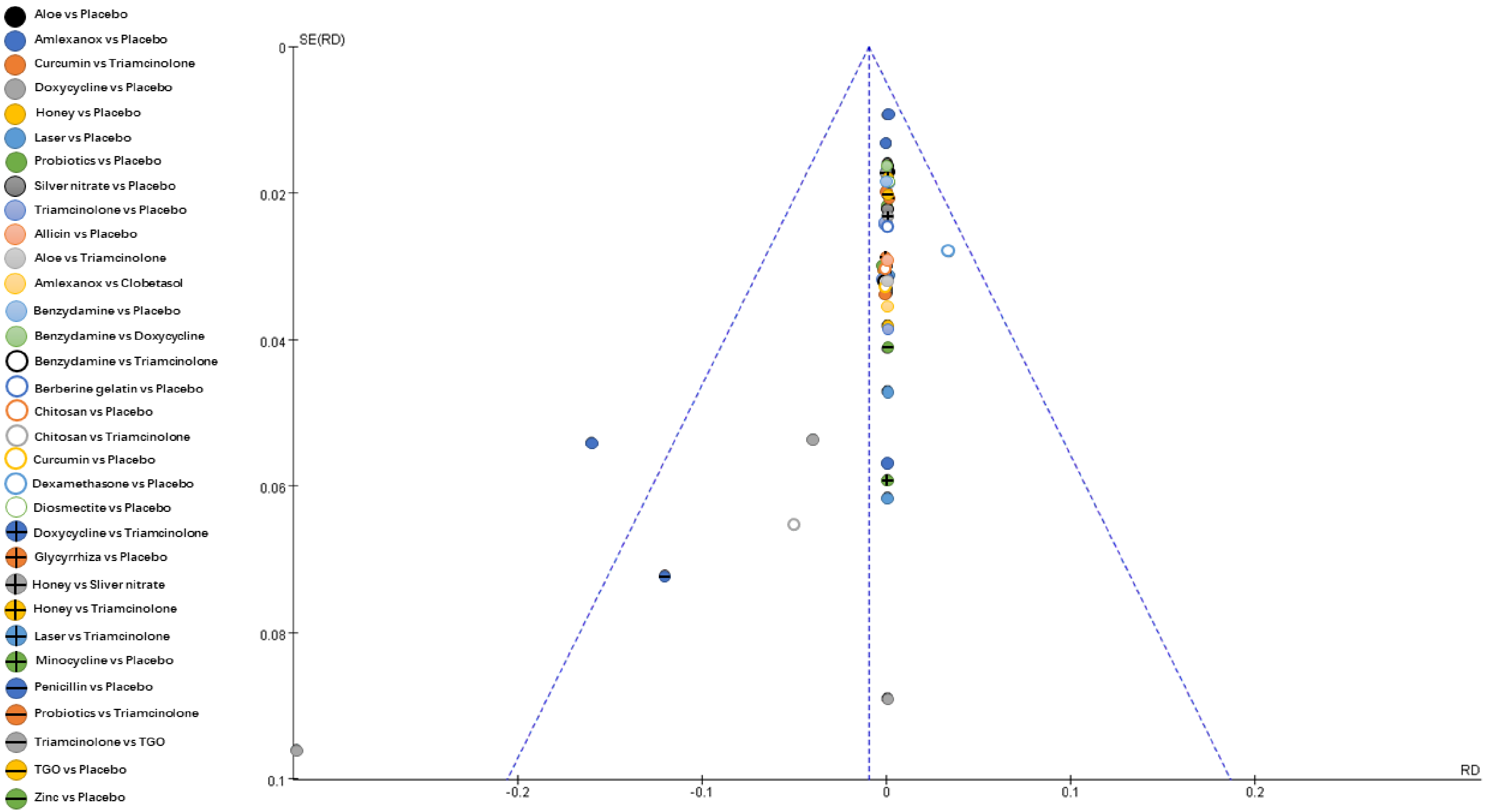

**Figure S7** **Network structure of sensitivity analysis**

Network structure of sensitivity analysis. 69 RCTs were considered in sensitivity analysis for the four outcomes. (a) Healing effect, data from 26 RCTs (1306 participants) with 20 pairwise comparisons among 18 interventions were pooled. (b) Size-reducing effect, data from 37 RCTs (3587 participants) with 30 pairwise comparisons among 19 interventions were pooled. (c) Symptom-reducing effect, data from 46 RCTs (4020 participants) with 32 pairwise comparisons among 23 interventions were pooled. (d) Adverse effect, data from 36 RCTs (2787 participants) with 32 pairwise comparisons among 22 interventions were pooled. Nodes and edges are weighted according to volume of studies including that treatment or comparison.

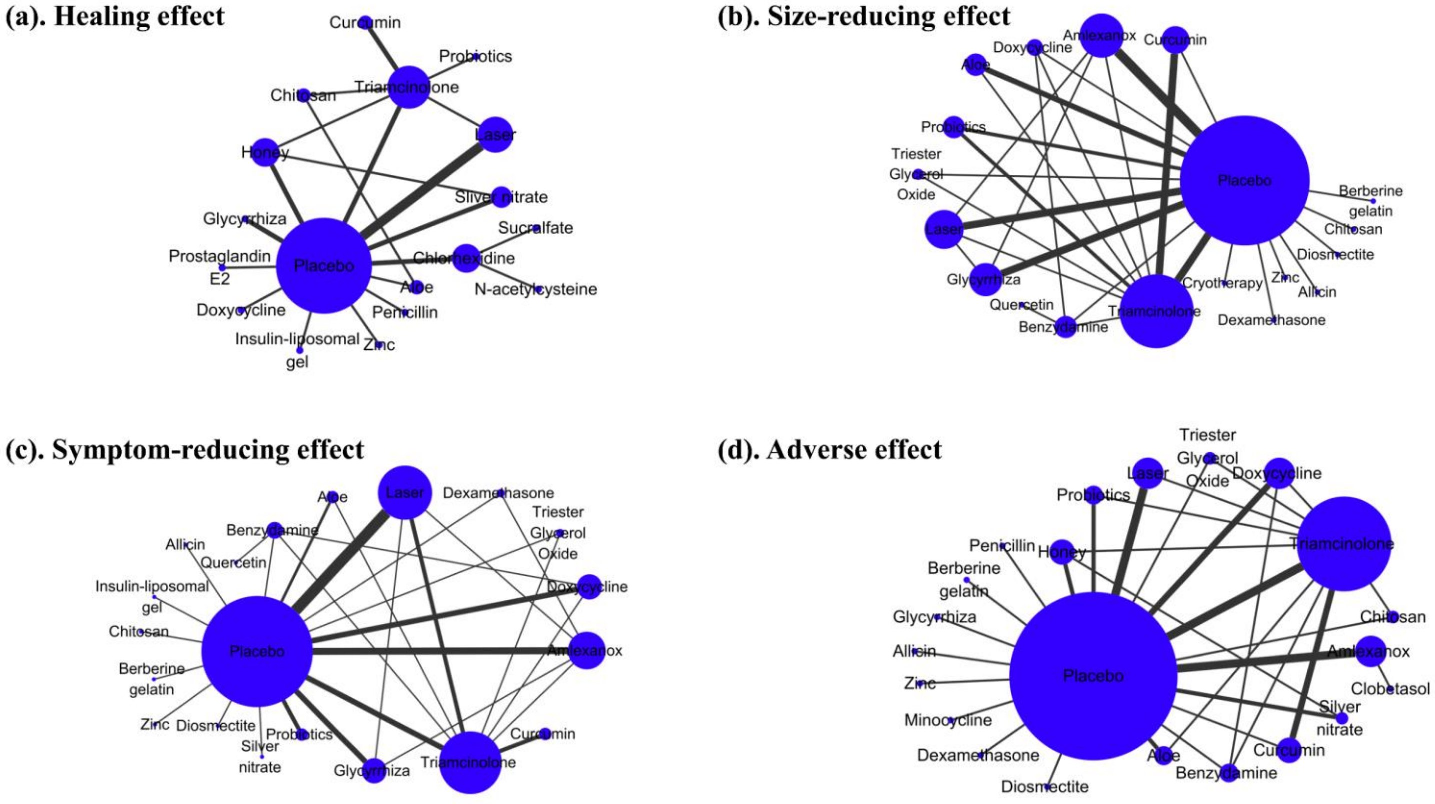

**Table S11 PRISMA NMA Checklist of Items**

| Section/Topic | Item # | Checklist Item | Reported on Page # |
|---|---|---|---|
| **TITLE** | | | |
| Title | 1 | Identify the report as a systematic review *incorporating a network meta-analysis (or related form of meta-analysis).* | 1 |
| **ABSTRACT** | | | |
| Structured summary | 2 | Provide a structured summary including: **Background, Methods, Results, Discussion/Conclusions, and Other.** | 2 |
| **INTRODUCTION** | | | |
| Rationale | 3 | Describe the rationale for the review in the context of what is already known*, including mention of why a network meta-analysis has been conducted.* | 3-4 |
| Objectives | 4 | Provide an explicit statement of questions being addressed, with reference to participants, interventions, comparisons, outcomes, and study design (PICOS). | 3-4 |
| **METHODS** | | | |
| Protocol and registration | 5 | Indicate whether a review protocol exists and if and where it can be accessed (e.g., Web address); and, if available, provide registration information, including registration number. | 4 |
| Eligibility criteria | 6 | Specify study characteristics (e.g., PICOS, length of follow-up) and report characteristics (e.g., years considered, language, publication status) used as criteria for eligibility, giving rationale. | 4 |
| Information sources | 7 | Describe all information sources (e.g., databases with dates of coverage, contact with study authors to identify additional studies) in the search and date last searched. | 4 |
| Search | 8 | Present full electronic search strategy for at least one database, including any limits used, such that it could be repeated. | Table S1-S5 |
| Study selection | 9 | State the process for selecting studies (i.e., screening, eligibility, included in systematic review, and, if applicable, included in the meta-analysis). | Fig. 1 |
| Data collection process | 10 | Describe method of data extraction from reports (e.g., piloted forms, independently, in duplicate) | 4-6 |

| | | | |
|---|---|---|---|
| | | and any processes for obtaining and confirming data from investigators. | |
| Data items | 11 | List and define all variables for which data were sought (e.g., PICOS, funding sources) and any assumptions and simplifications made. | 4-6 |
| **Geometry of the network** | **S1** | Describe methods used to explore the geometry of the treatment network under study and potential biases related to it. This should include how the evidence base has been graphically summarized for presentation, and what characteristics were compiled and used to describe the evidence base to readers. | 5 |
| Risk of bias within individual studies | 12 | Describe methods used for assessing risk of bias of individual studies (including specification of whether this was done at the study or outcome level), and how this information is to be used in any data synthesis. | 5 |
| Summary measures | 13 | State the principal summary measures (e.g., risk ratio, difference in means). | 5 |
| Planned methods of analysis | 14 | Describe the methods of handling data and combining results of studies for each network meta-analysis. This should include, but not be limited to:<br>• *Handling of multi-arm trials;*<br>• *Selection of variance structure;*<br>• *Selection of prior distributions in Bayesian analyses; and*<br>• *Assessment of model fit.* | 5 |
| **Assessment of Inconsistency** | **S2** | Describe the statistical methods used to evaluate the agreement of direct and indirect evidence in the treatment network(s) studied. Describe efforts taken to address its presence when found. | 5 |
| Risk of bias across studies | 15 | Specify any assessment of risk of bias that may affect the cumulative evidence (e.g., publication bias, selective reporting within studies). | 5 |
| Additional analyses | 16 | Describe methods of additional analyses if done, indicating which were pre-specified. This may include, but not be limited to, the following:<br>• Sensitivity or subgroup analyses;<br>• Meta-regression analyses;<br>• *Alternative formulations of the treatment network; and*<br>• *Use of alternative prior distributions for Bayesian analyses (if applicable).* | 5 |

| | | | |
|---|---|---|---|
| **RESULTS†** | | | |
| Study selection | 17 | Give numbers of studies screened, assessed for eligibility, and included in the review, with reasons for exclusions at each stage, ideally with a flow diagram. | 6 |
| **Presentation of network structure** | **S3** | Provide a network graph of the included studies to enable visualization of the geometry of the treatment network. | Fig. 2 |
| **Summary of network geometry** | **S4** | Provide a brief overview of characteristics of the treatment network. This may include commentary on the abundance of trials and randomized patients for the different interventions and pairwise comparisons in the network, gaps of evidence in the treatment network, and potential biases reflected by the network structure. | 7-9 |
| Study characteristics | 18 | For each study, present characteristics for which data were extracted (e.g., study size, PICOS, follow-up period) and provide the citations. | Table S4 |
| Risk of bias within studies | 19 | Present data on risk of bias of each study and, if available, any outcome level assessment. | Figure S1-S2 |
| Results of individual studies | 20 | For all outcomes considered (benefits or harms), present, for each study: 1) simple summary data for each intervention group, and 2) effect estimates and confidence intervals. | Table S6 |
| Synthesis of results | 21 | Present results of each meta-analysis done, including confidence/credible intervals. If additional summary measures were explored (such as treatment rankings), these should also be presented. | 7-8 |
| **Exploration for inconsistency** | **S5** | Describe results from investigations of inconsistency. This may include such information as measures of model fit to compare consistency and inconsistency models, *P* values from statistical tests, or summary of inconsistency estimates from different parts of the treatment network. | 11 |
| Risk of bias across studies | 22 | Present results of any assessment of risk of bias across studies for the evidence base being studied. | 8 |
| Results of additional analyses | 23 | Give results of additional analyses, if done (e.g., sensitivity or subgroup analyses, meta-regression analyses, *alternative network geometries studied, alternative choice of prior distributions for* | 8-9 |

| | | | |
|---|---|---|---|
| | | *Bayesian analyses,* and so forth). | |
| **DISCUSSION** | | | |
| Summary of evidence | 24 | Summarize the main findings, including the strength of evidence for each main outcome; consider their relevance to key groups (e.g., healthcare providers, users, and policy-makers). | 15-17 |
| Limitations | 25 | Discuss limitations at study and outcome level (e.g., risk of bias), and at review level (e.g., incomplete retrieval of identified research, reporting bias). *Comment on the validity of the assumptions, such as transitivity and consistency. Comment on any concerns regarding network geometry (e.g., avoidance of certain comparisons).* | 17 |
| Conclusions | 26 | Provide a general interpretation of the results in the context of other evidence, and implications for future research. | 17 |
| **FUNDING** | | | |
| Funding | 27 | Describe sources of funding for the systematic review and other support (e.g., supply of data); role of funders for the systematic review. This should also include information regarding whether funding has been received from manufacturers of treatments in the network and/or whether some of the authors are content experts with professional conflicts of interest that could affect use of treatments in the network. | 17 |
